# Effectiveness and Safety of Ayurvedic Medicines in Type 2 Diabetes Mellitus Management: A Systematic Review and Meta-Analysis

**DOI:** 10.3389/fphar.2022.821810

**Published:** 2022-06-08

**Authors:** Kaushik Chattopadhyay, Haiquan Wang, Jaspreet Kaur, Gamze Nalbant, Abdullah Almaqhawi, Burak Kundakci, Jeemon Panniyammakal, Michael Heinrich, Sarah Anne Lewis, Sheila Margaret Greenfield, Nikhil Tandon, Tuhin Kanti Biswas, Sanjay Kinra, Jo Leonardi-Bee

**Affiliations:** ^1^ Division of Epidemiology and Public Health, School of Medicine, University of Nottingham, Nottingham, United Kingdom; ^2^ The Nottingham Centre for Evidence-Based Healthcare: A JBI Centre of Excellence, Nottingham, United Kingdom; ^3^ Department of Family and Community Medicine, College of Medicine, King Faisal University, Alahsa, Saudi Arabia; ^4^ Sree Chitra Tirunal Institute for Medical Sciences and Technology, Thiruvananthapuram, India; ^5^ Centre for Pharmacognosy and Phytotherapy, School of Pharmacy, University College London, London, United Kingdom; ^6^ Institute of Applied Health Research, University of Birmingham, Birmingham, United Kingdom; ^7^ Department of Endocrinology, Metabolism and Diabetes, All India Institute of Medical Sciences, New Delhi, India; ^8^ Department of Kayachikitsa, J B Roy State Ayurvedic Medical College and Hospital, Kolkata, India; ^9^ Department of Non-communicable Disease Epidemiology, London School of Hygiene and Tropical Medicine, London, United Kingdom

**Keywords:** ayurveda, effectiveness, meta-analysis, safety, systematic review, type 2 diabetes mellitus

## Abstract

**Introduction:** Many Ayurvedic medicines have the potential for managing type 2 diabetes mellitus (T2DM), with previous systematic reviews demonstrating effectiveness and safety for specific Ayurvedic medicines. However, many of the reviews need updating and none provide a comprehensive summary of all the Ayurvedic medicines evaluated for managing T2DM.

**Objective:** The objective of this systematic review was to evaluate and synthesize evidence on the effectiveness and safety of Ayurvedic medicines for managing T2DM.

**Inclusion criteria:** Published and unpublished RCTs assessing the effectiveness and safety of Ayurvedic medicines for managing T2DM in adults.

**Methods:** The JBI systematic review methodology was followed. A comprehensive search of sources (including 18 electronic databases) from inception to 16 January 2021 was made. No language restrictions were applied. Data synthesis was conducted using narrative synthesis and random effects meta-analyses, where appropriate. Pooled results are reported as mean differences (MD) with 95% confidence intervals (CI).

**Results:** Out of 32,519 records identified from the searches, 219 articles were included in the systematic review representing 199 RCTs (21,191 participants) of 98 Ayurvedic medicines. Overall, in the studies reviewed the methodology was not adequately reported, resulting in poorer methodological quality scoring. Glycated hemoglobin (HbA1c) was reduced using *Aegle marmelos* (L.) Corrêa (MD -1.6%; 95% CI −3 to −0.3), *Boswellia serrata* Roxb. (−0.5; −0.7 to −0.4), *Gynostemma pentaphyllum* (Thunb.) Makino (−1; −1.5 to −0.6), *Momordica charantia* L. (−0.3; −0.4 to −0.1), *Nigella sativa* L. (−0.4; −0.6 to −0.1), *Plantago ovata* Forssk. (−0.9; −1.4 to −0.3), *Tinospora cordifolia* (Willd.) Hook.f. and Thomson (−0.5; −0.6 to −0.5), *Trigonella foenum-graecum* L. (−0.6; −0.9 to −0.4), and *Urtica dioica* L. (−1.3; −2.4 to −0.2) compared to control. Similarly, fasting blood glucose (FBG) was reduced by 4–56 mg/dl for a range of Ayurvedic medicines. Very few studies assessed health-related quality of life (HRQoL). Adverse events were not reported in many studies, and if reported, these were mostly none to mild and predominately related to the gastrointestinal tract.

**Conclusion:** The current evidence suggests the benefit of a range of Ayurvedic medicines in improving glycemic control in T2DM patients. Given the limitations of the available evidence and to strengthen the evidence base, high-quality RCTs should be conducted and reported.

## 1 Introduction

Type 2 diabetes mellitus (T2DM) is a complex disorder that has major health, social, and economic consequences. (European Medicines Agency, 2018; International Diabetes Federation, 2019) Chronic hyperglycemia is associated with macro- and micro-vascular complications and even death. (European Medicines Agency, 2018; International Diabetes Federation, 2019) Ayurveda is a dominant traditional medical system that has been used for thousands of years in many South Asian countries. (Sharma et al., 2007) In Ayurveda, the corresponding term for diabetes mellitus is madhumeha (madhu means ‘‘sweetness’’ and meha means ‘‘excessive urination’’). (Ministry of Ayush, 2016; Central Council for Research in Ayurvedic Sciences, 2017) Classical Ayurvedic texts, written in Sanskrit, have described this condition and its management in detail. (Ministry of Ayush, 2016; Central Council for Research in Ayurvedic Sciences, 2017) Briefly, a multi-pronged and individualized approach is used to manage the condition such as through lifestyle modification (including diet), Ayurvedic detoxifying and purifying therapies (e.g., Panchakarma), and Ayurvedic medicines (containing plant-, animal- or mineral-origin ingredients–single or in combination). It is hypothesized that many of these medicines work through pancreatic as well as extrapancreatic effects. (Ministry of Ayush, 2016; Central Council for Research in Ayurvedic Sciences, 2017) T2DM is one of the main diseases for which patients consult Ayurvedic practitioners and use Ayurvedic medicines, often continuously from the point of diagnosis. (Mehrotra et al., 2004; Kumar et al., 2006; Priya and Shweta, 2010; Chandra, 2011; Bhalerao et al., 2013; Chandra, 2013) Ayurveda is commonly used by patients as it fits with their health beliefs and culture; thus, its acceptability, satisfaction, and perceived relief are usually high, especially among rural, poor, older, and indigenous/minority populations. (Chacko, 2003; Bhalerao et al., 2013) Many T2DM patients prefer not to use Western medicines due to the associated side effects, cost, and mode of administration (e.g., injections). (Chandra, 2011; Bhalerao et al., 2013; Chandra, 2013)

Previous systematic reviews of clinical trials suggest beneficial effects of several Ayurvedic medicines on T2DM-related outcomes, including improvement in blood glucose, with no major safety issues. (Hardy et al., 2001; Shekelle et al., 2005; Sridharan et al., 2011) However, they are now outdated, and one was limited in scope in terms of the Ayurvedic medicines considered. The evidence base over the past 10 years has grown substantially, thereby highlighting the need to refocus and update the reviews to provide contemporary estimates of effect and safety for all the Ayurvedic medicines for the management of T2DM. Additionally, the review findings will be used to guide the development of a clinical guideline for managing T2DM using Ayurvedic medicines.

## 2 Review Questions

i) Are Ayurvedic medicines effective in controlling blood glucose levels in T2DM patients?
ii) Are Ayurvedic medicines effective in improving health-related quality of life (HRQoL) for T2DM patients?
iii) Are Ayurvedic medicines safe for use by T2DM patients?

## 3 Materials and Methods

The systematic review process adhered to the JBI Systematic Reviews of Effectiveness guidance and was reported following the Preferred Reporting Items for Systematic Reviews and Meta-Analyses (PRISMA) guidelines. (Moher et al., 2009; Tufanaru et al., 2017) This review was conducted according to *a priori* published protocol, (Chattopadhyay et al., 2020a) and registered with PROSPERO (CRD42018118285).

### 3.1 Inclusion Criteria

#### 3.1.1 Participants

The systematic review included studies conducted among adults (≥18 years) with T2DM, irrespective of associated comorbidities (e.g., obesity, hypertension, dyslipidemia) or T2DM complications (such as macro- or micro-vascular). Both newly diagnosed T2DM/treatment naïve, as well as existing cases/on treatment, were eligible. Studies with mixed populations, e.g., adults and children, were included if the mean age of the participants was ≥18 years or the study findings were reported separately for adults. Studies that included participants with type 1 diabetes were excluded unless it was possible to extract the data on T2DM participants.

#### 3.1.2 Interventions

Studies were included if they assessed any classical or proprietary Ayurvedic medicine (such as containing plant- or mineral-origin ingredients–single or in combination) in any form (e.g., tablets, capsules, powder, decoction) and administered for at least 8 weeks. Cross-checking of the eligibility of Ayurvedic medicine (to distinguish it from traditional Chinese and Western medicines) was performed by Ayurveda experts in the team *via* searching the Indian Medicinal Plants Database (http://medicinalplants.in), Encyclopedia on Indian Medicinal Plants (http://envis.frlht.org/implad), Traditional Knowledge Digital Library (http://www.tkdl.res.in), Ayurvedic Pharmacopoeia of India, and Ayurvedic Formulary of India. Studies on multi-modal interventions that included Ayurvedic medicine were included if it was possible to extract data relating to Ayurvedic medicine. To distinguish between Ayurvedic medicines and dietary ingredients, studies were excluded where medicines were used as dietary ingredients or foods. In addition, Ayurvedic detoxifying and purifying therapies (e.g., Panchakarma) were beyond the scope of this review.

#### 3.1.3 Comparators

Studies comparing Ayurvedic medicines with no intervention, placebo (as defined by the study authors), non-pharmaceutical intervention (e.g., yoga), or pharmaceutical intervention (i.e., Western oral antidiabetic drug [OAD] or head-to-head comparison with another Ayurvedic medicine) were eligible for inclusion in the review. Co-intervention was allowed if all the eligible study arms received the same co-intervention. If a study had multiple treatment arms (multi-arms), the authors only included the arms that met the review inclusion criteria. Studies comparing two or more drug manufacturing processes, forms or timings of administration, doses, or Anupans (Ayurvedic medicines are usually taken with an Anupan, a carrier substance such as a liquid drink) of the same Ayurvedic medicine without any other comparator were excluded.

#### 3.1.4 Outcomes

The following outcomes were included:

• Primary outcomes: blood glucose (i.e., glycated hemoglobin [HbA1c], fasting blood glucose [FBG]), HRQoL, and adverse events.
• Secondary outcomes: postprandial blood glucose (PPBG), fasting and stimulated insulin, fasting and stimulated C-peptide, insulin resistance (homeostasis model assessment of insulin resistance [HOMA-IR]), body weight, body mass index (BMI), waist circumference, systolic blood pressure (SBP), diastolic blood pressure (DBP), heart rate, and serum lipid (i.e., total cholesterol [TC], high-density lipoprotein cholesterol [HDL-C], low-density lipoprotein cholesterol [LDL-C], triglycerides [TG]).

The timing of outcome measurement had to be at least 8 weeks from randomization since this is the recommended minimum time for T2DM management studies. (Sridharan et al., 2011; European Medicines Agency, 2018) The management of complications of T2DM was beyond the scope of this review.

#### 3.1.5 Types of Studies

The review included RCTs, of any design. Only the first phase of cross-over trials was included to avoid any carry-over of treatment effect.

### 3.2 Search Strategy

The authors searched for both published and unpublished studies via the following electronic databases and gray literature sources: MEDLINE (Ovid; from 1946), Embase (Ovid; from 1974), CINAHL (EBSCOhost; from 1937), PsycINFO (Ovid; from 1806), Web of Science (from 1900), Cochrane Central Register of Controlled Trials (CENTRAL; from 1996), Allied and Complementary Medicine Database (AMED; Ovid; from 1985), International Pharmaceutical Abstracts (Ovid; from 1970), Turning Research Into Practice (TRIP; from 1997), AYUSH Research Portal (a database of Ayurveda and other research articles; http://ayushportal.nic.in), Digital Helpline for Ayurveda Research Articles (DHARA; http://dharaonline.org), A Bibliography of Indian Medicine (ABIM; http://indianmedicine.eldoc.ub.rug.nl), CAM-QUEST (a database of complementary and alternative medicine research articles; https://www.cam-quest.org/en), Directory of Open Access Journals, EthOS, OpenGrey, and ProQuest Dissertations and Theses. The databases were searched on 16th January 2021. The search strategies are detailed in Supplementary Appendix S1, which were developed based on the previous systematic reviews and clinical guidelines on this topic (Hardy et al., 2001; Shekelle et al., 2005; Central Council for Research in Ayurvedic Sciences, 2011; Sridharan et al., 2011; National Institute for Health and Care Excellence, 2015; Ministry of Ayush, 2016; Ministry of Health and Family Welfare, 2016; Central Council for Research in Ayurvedic Sciences, 2017; Central Council for Research in Ayurvedic Sciences and Directorate General of Health Services, 2018) and in consultation with an experienced information specialist. A combination of search terms and index terms was used. No language restrictions were applied, and an external company was hired for professional translations. Similarly, no date restrictions were applied. Furthermore, Researches in Ayurveda and Ayurvedic Research Database (ARD; databases of Ayurveda-related dissertations and theses; seventh edition; 2001–2008; https://ayurvedahealthcare.info) was searched for additional studies. The reference list of relevant previous systematic reviews and included studies and the tables of content of 48 Ayurveda journals were screened till 16th January 2021 for additional studies. Relevant experts in the field were contacted (at least twice through email), including the Central Council for Research in Ayurvedic Sciences (Ministry of Ayush, India) and authors of the included studies (and manufacturers of the included Ayurvedic medicines), to locate additional studies.

### 3.3 Study Selection

Following the searches, the identified citations were collated and uploaded into EndNote v8.2 (Clarivate, Philadelphia, United States), and duplicate citations were removed. The remaining citations were uploaded into Rayyan [Qatar Computing Research Institute (Data Analytics), Doha, Qatar], and titles and abstracts were screened for eligibility by two independent reviewers. Studies identified as potentially eligible or those without an abstract had their full text retrieved. In case the full text of an article was not available even through the interlibrary loan service/British Library, the author and journal editor were approached (at least twice through email). Potentially eligible ongoing RCTs were contacted (at least twice through email) by the authors to access the study results. Full texts of the studies were assessed for eligibility by two independent reviewers. Any disagreements that arose between the two reviewers were resolved through discussion and in consultation with a third reviewer. Full texts of the studies that did not meet the inclusion criteria were excluded, and reasons for exclusion were recorded. In the case of multiple publications of the same study, the article having the most complete data was included. If partial data were provided in each article, then all such articles were included.

### 3.4 Assessment of Methodological Quality

Included studies were critically assessed using the standardized JBI critical appraisal checklist for RCTs independently by two reviews, assigning a score as met (yes), not met (no), or unclear. (Tufanaru et al., 2017) A third reviewer compared their work and highlighted the disagreements between the two reviewers. Any such disagreements were resolved through discussion between the three reviewers. All studies, regardless of their methodological quality, were included in the review.

### 3.5 Data Extraction

Two reviewers independently extracted data from the included studies, using a pre-developed and pre-tested data extraction tool. A third reviewer compared their work and highlighted the disagreements between the two reviewers. Any such disagreements were resolved through discussion between the three reviewers. Data extraction included details about the population, intervention, comparator, and outcomes. For biochemical, anthropometric, and physiological parameters and HRQoL data, the authors extracted the 8-week time point data. Where this time point was not reported or multiple time points were reported, data from the time point closest to 8 weeks were extracted. For adverse events, the authors extracted the end of study data. Intention-to-treat (ITT) data were preferred compared to per-protocol data. Post-intervention data were extracted in preference to change from baseline data (i.e., post-intervention score–baseline score). Percentage change from baseline was not extracted due to the sensitivity to changes in variance and failure to protect from baseline imbalances, thus leading to non-normally distributed outcome data. (Vickers, 2001) For each outcome, the data were converted into one standard unit of measurement. In multi-arm studies, where two or more drug manufacturing processes, forms or timings of administration, doses, or Anupans of the same Ayurvedic medicine were compared with another comparator, the two or more study arms were combined together before pooling with other studies. (Hedges and Olkin, 1985; Cohen, 1988) This was also performed for multi-arm studies when the interventions and comparators were provided with co-interventions (e.g., Ayurvedic medicine arm pooled with Ayurvedic medicine and exercise arm versus placebo arm pooled with placebo and exercise arm).

Multiple strategies were used to obtain the relevant missing data. The corresponding author of the included study was contacted by email (at least two times per author) to obtain the missing data. If the standard deviation (SD) was missing (in a small number of studies), SD was imputed from a similar study (in terms of intervention, comparator, sample size, and numerical outcome data). If only a median and interquartile range (IQR) was reported (in a small number of studies), the mean was assumed to be equal to the median and the SD was calculated using a standard formula (=IQR/1.35). (Higgins et al., 2019)

### 3.6 Data Synthesis

Initially, narrative syntheses were conducted to describe the studies. For each outcome, where at least two studies on an Ayurvedic medicine were included, random-effects meta-analyses were conducted to provide a weighted measure of treatment effect. For continuous outcomes, mean differences (MD) with 95% confidence intervals (CI) were reported where the same scale was used across studies. Where different scales were used across studies, standardized mean differences (SMD) with 95% CIs were reported. Where necessary, post-intervention data were pooled with change from baseline data and only for MDs but not SMDs. For the purpose of analysis, the following comparators were combined together: no medicine, no additional medicine, and placebo. For studies with more than one comparator group (e.g., mentioned above, OAD), the comparisons were included in separate meta-analysis models to avoid the issue of double-counting of the comparator group. Statistical heterogeneity was quantified using the *I* ^2^ statistic. Analyses were conducted using STATA v16 for Windows (STATACorp, College Station, Texas, United States). The findings are interpreted from the clinical point of view.

### 3.7 Assessment of Publication Bias

Funnel plots were used to assess publication bias, where there were at least 10 studies included in the meta-analysis.

### 3.8 Sensitivity Analyses

Where a significant pooled association was found between the intervention and primary outcome, sensitivity analysis was performed to assess the robustness of the result by excluding studies that were not journal publications (i.e., not peer-reviewed). After excluding these studies, at least two eligible studies were needed for the sensitivity analysis.

### 3.9 Subgroup Analyses

Where a significant pooled association was found between the intervention and primary outcome, subgroup analysis was performed to explore the influence of the following factors on the result:

• Country: South Asia (i.e., Afghanistan, Bangladesh, Bhutan, India, Maldives, Nepal, Pakistan, or Sri Lanka) versus others.
• Comparator: no medicine or no additional medicine versus placebo.

For this purpose, at least two eligible studies per subgroup were needed.

## 4 Assessing Certainty in the Findings

The Grading of Recommendations, Assessment, Development and Evaluation (GRADE) method was used to assess the certainty of the findings (i.e., the primary outcomes). (Schünemann et al., 2013) Only those Ayurvedic medicines were included where the meta-analysis (at least two studies were needed) was possible on at least one primary outcome. Two reviewers were involved in the process, and the findings were initially ranked as high and were downgraded to moderate, low, or very low if there was evidence of the following: risk of bias, inconsistency of results, indirectness of evidence, imprecision, and/or publication bias. More specifically, in the risk of bias domain, the following were considered for downgrading: no allocation concealment (no to Q2 in the JBI critical appraisal checklist for RCTs), lack of blinding (no to Q4,5,6), attrition bias (no to Q9), and selective outcome reporting. If an issue out of these four was present in the majority of studies (i.e., >50%), then it was downgraded by one level. If more than one issue was present, then it was downgraded by two levels. In the inconsistency of results domain, if the statistical heterogeneity (i.e., *I* ^2^ statistic) was 75%–89%, then it was downgraded by one level. If the *I* ^2^ statistic was ≥90%, then it was downgraded by two levels. In the imprecision domain, if the total sample size was 100 to <400, then it was downgraded by one level. If the total sample size was <100, then it was downgraded by two levels. If the total sample size was ≥400 with wide CI and not earlier downgraded for inconsistency, then it was downgraded by one level. If publication bias was detected, then it was downgraded by one level. (See Summary of Findings table [Supplementary Material]).

## 5 Results

### 5.1 Inclusion of Studies

**FIGURE 1:**
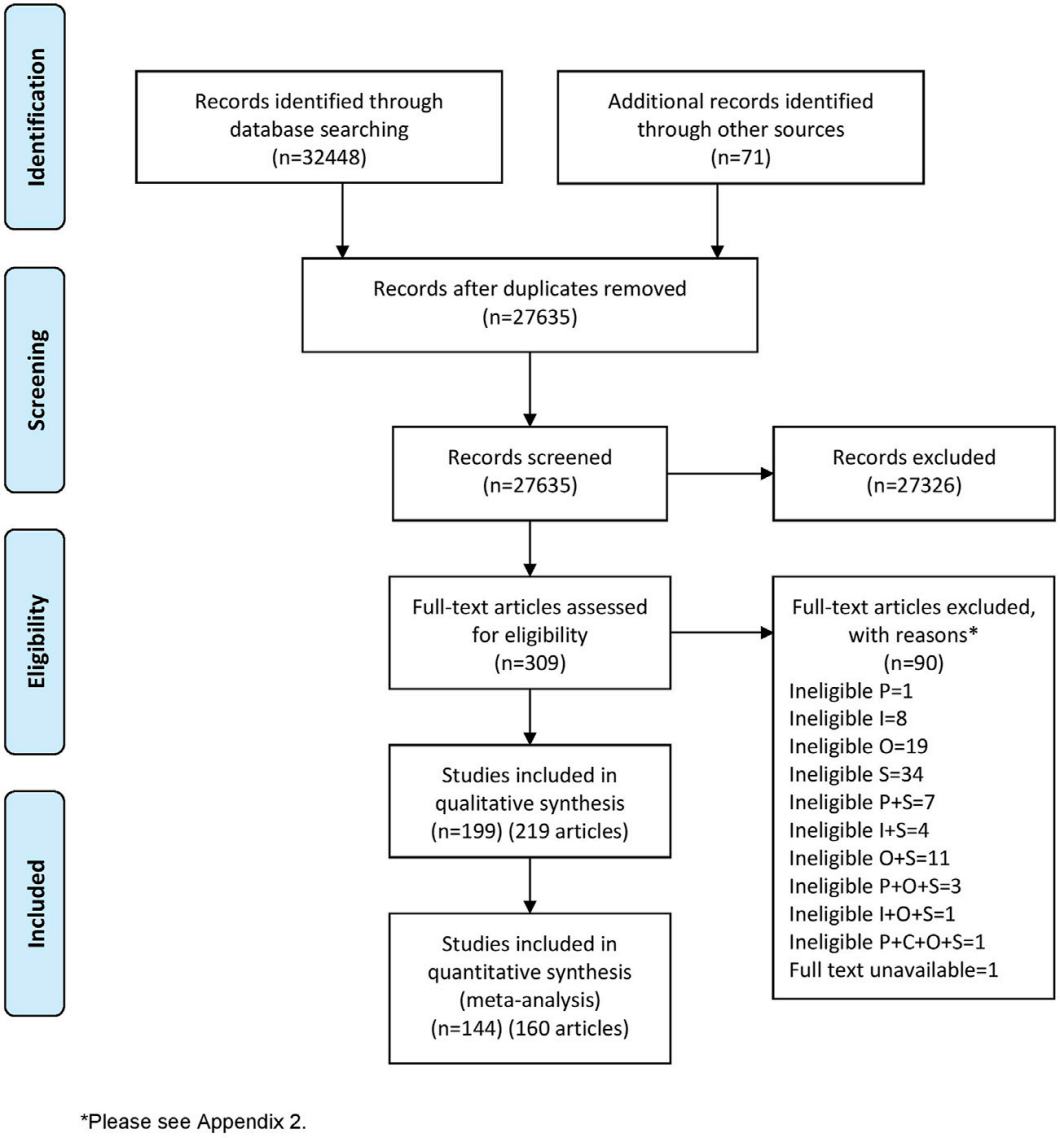
PRISMA flow diagram (study selection and inclusion process).

The literature search identified 32,519 records. After removing duplicate records, 27,635 titles and abstracts were screened. Full texts of 309 records were sought, and their eligibility for inclusion was assessed. 219 records were included in this systematic review representing 199 RCTs and including 21,191 participants (in the eligible study arms) (Figure 1). Data from 144 RCTs (160 records) were included in the meta-analyses. A further 28 ongoing RCTs were included, which are reported in Supplementary Appendix S2. The reasons for the exclusion of 90 records excluded at the full text screening stage are reported in Supplementary Appendix S3.

### 5.2 Characteristics of Included Studies

Characteristics of 199 included studies are reported in Supplementary Appendix S4. 82 studies (41%) were conducted in South Asia (one in Bangladesh, 72 in India, eight in Pakistan, and one in Sri Lanka), and 72 studies (36%) in Iran. 26 RCTs (13%) recruited newly diagnosed/treatment naïve T2DM patients, 146 (73%) recruited existing cases/on OAD, and 22 (11%) recruited both; it was unclear in the remaining five RCTs. The mean duration of T2DM in existing cases ranged from 2.5 to 12.8 years. 28 RCTs recruited participants with physical or mental health comorbidities or complications of T2DM. The sample size of the studies ranged from 12 to 6,114 (only eligible study arms were considered). The mean age of participants ranged from 37.5 to 65.4 years. The percentage of female participants ranged from 0% to 100%. At baseline, the mean HbA1c and FBG ranged from 6.4 to 11.8% and 118–360 mg/dl, respectively. 42 RCTs (21%) received commercial funding or other support; however, it was unclear in 86 RCTs (43%).

### 5.3 Interventions

98 Ayurvedic medicines were included in the systematic review. The details of the interventions are provided in Supplementary Appendix S5. 56 Ayurvedic medicines were of single plant-origin (i.e., single herbs), two were of single mineral-origin (namely, Swarnamakshika Bhasma [ash obtained through incineration] and Yashad Bhasma), and Shilajit (a blackish-brown powder or an exudate from high mountain rocks). 31 Ayurvedic medicines contained ≥2 plant-origin ingredients, and one contained ≥2 mineral-origin ingredients (namely, Trivanga Bhasma). Seven Ayurvedic medicines were herbo-mineral formulations (namely, Hyponidd, Inolter, Madhumeha Nashini Gutika, Naga Bhasma and Nishamalaki combination, Nishamalaki and Shilajit combination, Salasaradi Kashaya, and Tejashiladi Vati). An oral route of administration was used for all of the Ayurvedic medicines, using a range of administration forms (including tablets, capsules, and powder) and timings of administration (ranging from one to four times daily). The daily doses of Ayurvedic medicines varied, depending on their type and form and timing of administration. Similarly, a range of Anupan of Ayurvedic medicines were reported. The treatment duration (and trial follow-up) ranged from 8 (i.e., based on the systematic review inclusion criteria) to 78 weeks (six RCTs reported 26 weeks, and one each reported 36, 39, 52, and 78 weeks).

### 5.4 Methodological Quality

Overall, in the studies reviewed the methodology was not adequately reported, resulting in poorer methodological quality scoring (Supplementary Appendix S6). The major issues in these studies were: 1) inadequately reporting the randomization process that was used to assign participants to study arms; 2) inadequately reporting the allocation concealment process; 3) inadequately reporting who was blinded to intervention assignment and how blinding was performed; 4) inadequate reporting of the placebo and on further interrogation some placebos were actually Ayurvedic medicines, and it was unclear if placebos were identical especially when other forms of administration like powder or liquid were used which are easy to differentiate taste and/or smell wise; 5) inadequately reporting whether the study arms were treated identically other than the intervention of interest and particularly, in existing cases/on OAD, it was not always clear whether OAD was continued for the rest of the trial; 6) inadequately reporting the ascertainment of outcomes (including adverse events); 7) errors/issues in the sample size calculation and reporting (e.g., not using the primary outcome or using an inappropriate outcome as primary for the sample size calculation, unclear *minimum* clinically important difference or using an inappropriate *minimum* clinically important difference for the sample size calculation; 8) errors/issues in data analysis and reporting (e.g., not performing ITT analysis or doing pre-post analysis of outcomes within study arms but no comparative analysis between study arms); and 9) in terms of the follow-up, not analyzing the differences between study arms, such as no analysis of the patterns of loss to follow-up and the impact of the loss to follow-up on results.

### 5.5 Effects of Interventions

Meta-analyses were conducted on 33 Ayurvedic medicines (32 single herbs and Shilajit). The majority of the Ayurvedic medicines were compared to no medicine or no additional medicine or placebo; however, two were compared to both no medicine or no additional medicine or placebo and OAD (*Momordica charantia* L. and *Trigonella foenum-graecum* L.) and two were compared to OAD (*Enicostemma axillare* (Lam.) Raynal and *Pterocarpus marsupium* Roxb.). (See ZIP file for forest and funnel plots [Supplementary Material]).

#### 5.5.1 *Aegle marmelos* (L.) Corrêa

**FIGURE 2:**
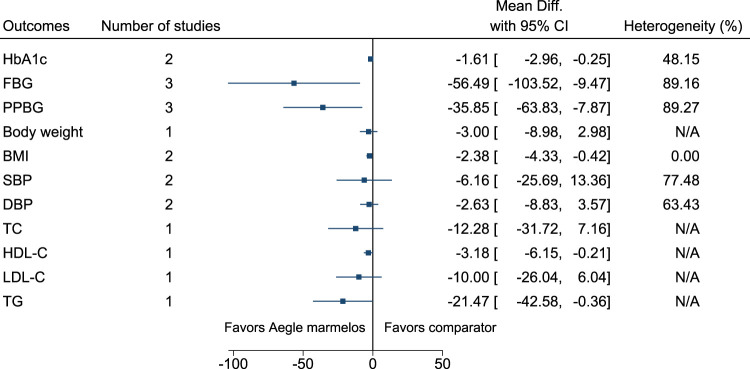
*Aegle marmelos*—Summary forest plot.

*Aegle marmelos* significantly reduced HbA1c (MD −1.6%; 95% CI −3 to −0.3), FBG (−56 mg/dl; −104 to −9), PPBG (−36 mg/dl; −64 to −8), and BMI (−2.4 kg/m^2^; −4.3 to −0.4) (see Figure 2). (Sankhla et al., 2009; Yaheya and Ismail, 2009; Sharma and Sharma, 2013; Nigam and Nambiar, 2019).

#### 5.5.2 *Allium sativum* L.

**FIGURE 3:**
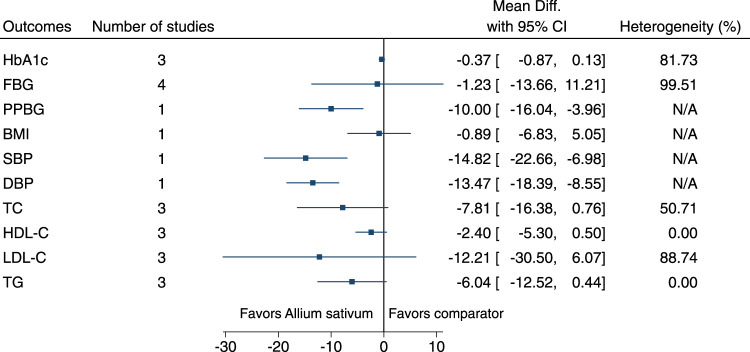
*Allium sativum*—Summary forest plot.

*Allium sativum* significantly reduced PPBG (−10 mg/dl; −16 to −4), SBP (−15 mmHg; −23 to −7), and DBP (−13 mmHg; −18 to −9) (see Figure 3). (Ashraf et al., 2005; Balasubramaniam et al., 2010; Ashraf et al., 2011a; Ashraf et al., 2011b; Kumar et al., 2013; Mansouri et al., 2018).

#### 5.5.3 *Aloe vera* L.

**FIGURE 4:**
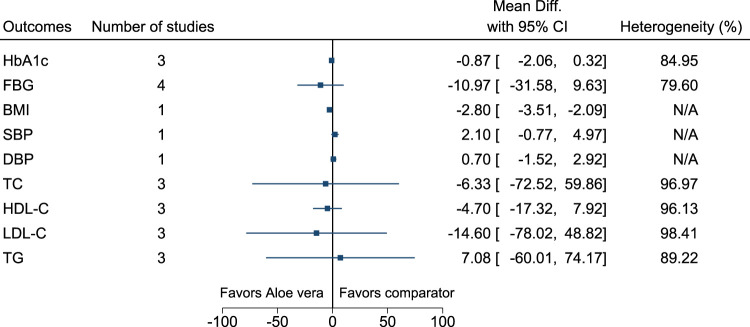
*Aloe vera*—Summary forest plot.

*Aloe vera* significantly reduced BMI (−2.8 kg/m^2^; −3.5 to −2.1) (see Figure 4). (Arora et al., 2009; Huseini et al., 2012a; Huseini et al., 2012b; Zarrintan et al., 2015; Maurya et al., 2017).

#### 5.5.4 *Anethum graveolens* L.

**FIGURE 5:**
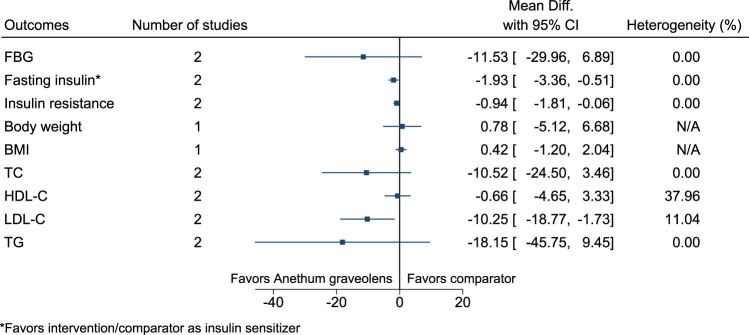
*Anethum graveolens*—Summary forest plot.

*Anethum graveolens* significantly reduced fasting insulin (as insulin sensitizer; −2 mIU/L; −3 to −1), insulin resistance (−0.9; −1.8 to −0.1), and LDL-C (−10 mg/dl; −19 to −2) (see Figure 5). (Mobasseri et al., 2014; Haidari et al., 2020).

#### 5.5.5 *Azadirachta indica* A.Juss.

**FIGURE 6:**
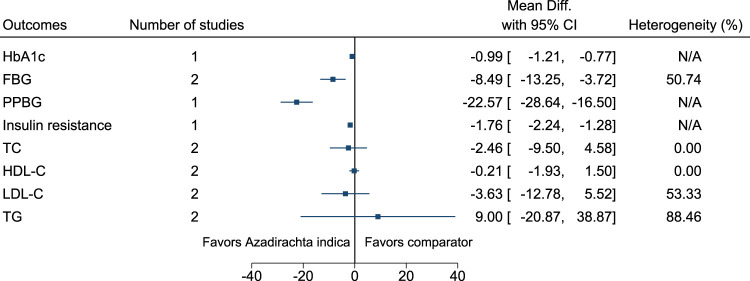
*Azadirachta indica* - Summary forest plot.

*Azadirachta indica* significantly reduced HbA1c (−1%; −1.2 to −0.8), FBG (−8 mg/dl; −13 to −4), PPBG (−23 mg/dl; −29 to −17), and insulin resistance (−1.8; −2.2 to −1.3) (see Figure 6). (Balasubramaniam et al., 2010; Pingali et al., 2020).

#### 5.5.6 *Boswellia serrata* Roxb.

**FIGURE 7:**
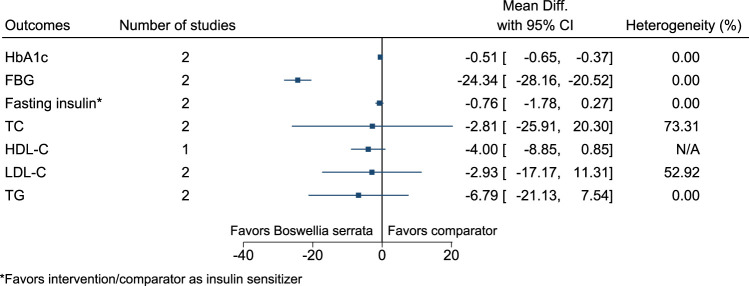
*Boswellia serrata*—Summary forest plot.

*Boswellia serrata* significantly reduced HbA1c (−0.5%; −0.7 to −0.4) and FBG (−24 mg/dl; −28 to −21) (see Figure 7). (Azadmehr et al., 2014; Mehrzadi et al., 2018).

#### 5.5.7 *Camellia sinensis* (L.) Kuntze

**FIGURE 8:**
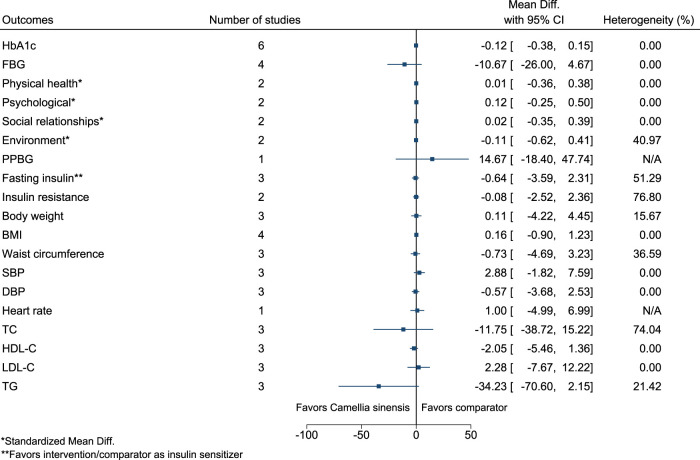
*Camellia sinensis*—Summary forest plot.

Figure 8 shows the summary forest plot for *Camellia sinensis*. (MacKenzie et al., 2007; Mirzaei et al., 2009; Hsu et al., 2011; Lasaite et al., 2014; Liu et al., 2014; Quezada-Fernández et al., 2019).

#### 5.5.8 *Cinnamomum aromaticum* Nees

**FIGURE 9:**
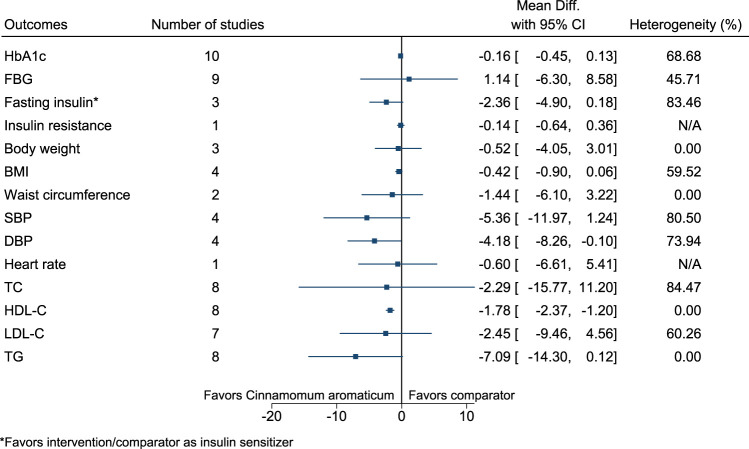
*Cinnamomum aromaticum*—Summary forest plot.

*Cinnamomum aromaticum* significantly increased HDL-C (2 mg/dl; 1–2) (see Figure 9). (Mang et al., 2006; Suppapitiporn et al., 2006; Blevins et al., 2007; Crawford, 2009; Akilen et al., 2010; Wainstein et al., 2011; Lu et al., 2012; Sharma et al., 2012; Hasanzade et al., 2013; Tangvarasittichai et al., 2015; Sengsuk et al., 2016).

#### 5.5.9 *Cinnamomum verum* J.Presl

**FIGURE 10:**
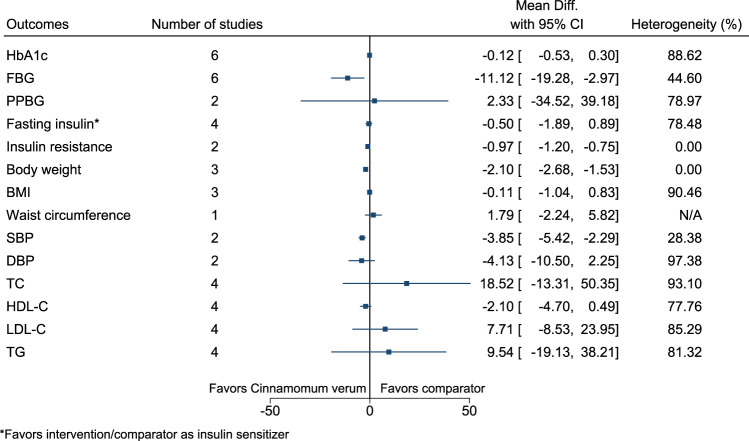
*Cinnamomum verum*—Summary forest plot.

*Cinnamomum verum* significantly reduced FBG (−11 mg/dl; −19 to −3), insulin resistance (−1; −1.2 to −0.8), body weight (−2.1 kg; −2.7 to −1.5), and SBP (−4 mmHg; −5 to −2) (see Figure 10). (Vafa et al., 2012; Zahmatkesh et al., 2012; Azimi et al., 2014; Azimi et al., 2016; Talaei et al., 2017a; Zare et al., 2019; Mirmiranpour et al., 2020).

#### 5.5.10 *Citrullus colocynthis* (L.) Schrad.

**FIGURE 11:**
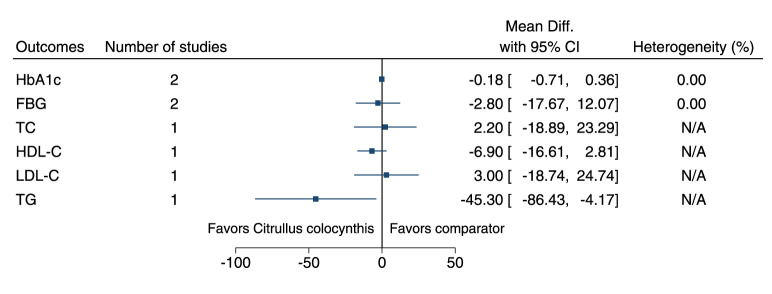
*Citrullus colocynthis* - Summary forest plot.

*Citrullus colocynthis* significantly reduced TG (−45 mg/dl; −86 to −4) (see Figure 11). (Huseini et al., 2009; Barghamdi et al., 2016).

#### 5.5.11 *Coccinia grandis* (L.) Voigt

**FIGURE 12:**
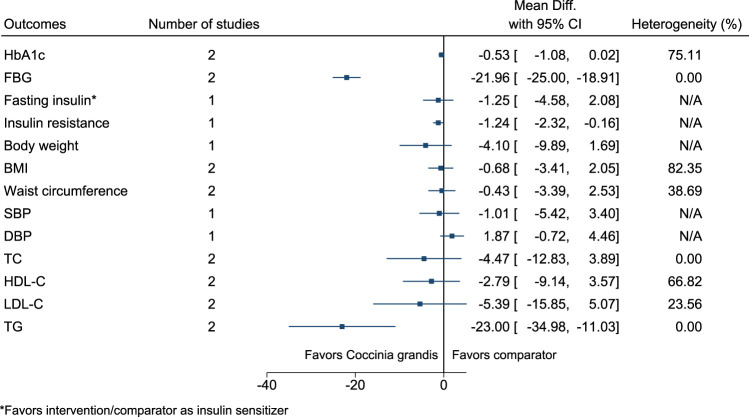
*Coccinia grandis*—Summary forest plot.

*Coccinia grandis* significantly reduced FBG (−22 mg/dl; −25 to −19), insulin resistance (−1.2; −2.3 to −0.2), and TG (−23 mg/dl; −35 to −11) (see Figure 12). (Kuriyan et al., 2008; Kurpad and Raj, 2008; Wasana et al., 2021).

#### 5.5.12 *Crocus sativus* L.

**FIGURE 13:**
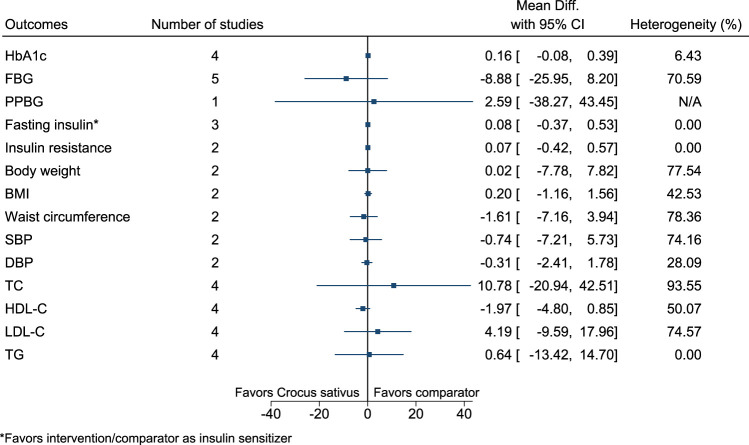
*Crocus sativus*—Summary forest plot.

Figure 13 shows the summary forest plot for *Crocus sativus*. (Azimi et al., 2014; Azimi et al., 2016; Milajerdi et al., 2018; Ebrahimi et al., 2019; Moravej Aleali et al., 2019; Mobasseri et al., 2020).

#### 5.5.13 *Cuminum cyminum* L.

**FIGURE 14:**
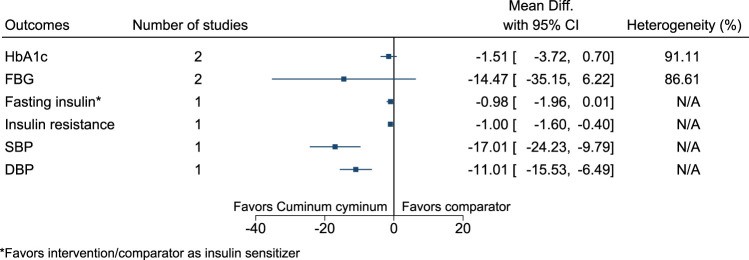
*Cuminum cyminum*—Summary forest plot.

*Cuminum cyminum* significantly reduced insulin resistance (−1; −1.6 to −0.4), SBP (−17 mmHg; −24 to −10), and DBP (−11 mmHg; −16 to −6) (see Figure 14). (Jafari et al., 2017; Mansouri et al., 2018; Hendre et al., 2020).

#### 5.5.14 *Curcuma longa* L.

**FIGURE 15:**
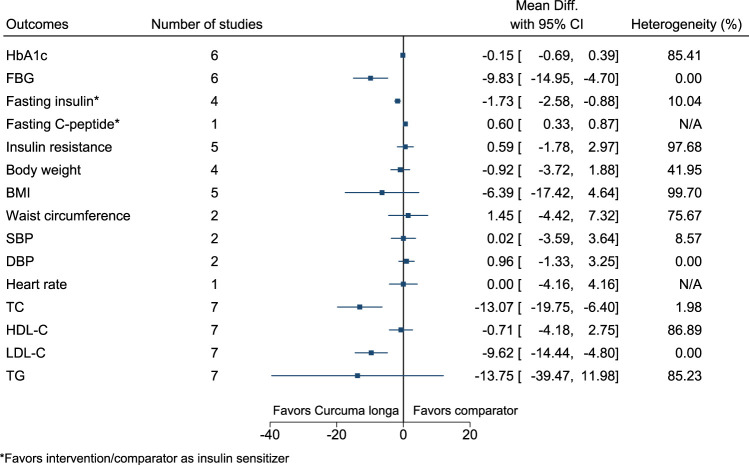
*Curcuma longa*—Summary forest plot.

*Curcuma longa* significantly reduced FBG (−10 mg/dl; −15 to −5), fasting insulin (as insulin sensitizer; −2 mIU/L; −3 to −1), TC (−13 mg/dl; −20 to −6), and LDL-C (−10 mg/dl; −14 to −5), and increased fasting C-peptide (as insulin sensitizer; 0.6 ng/ml; 0.33–0.87) (see Figure 15). (Usharani et al., 2008; Na et al., 2013; Chuengsamarn et al., 2014; Panahi et al., 2017; Panahi et al., 2018; Adab et al., 2019; Adibian et al., 2019; Srinivasan et al., 2019; de Sousa et al., 2020).

#### 5.5.15 *Cyamopsis tetragonoloba* (L.) Taub.

**FIGURE 16:**
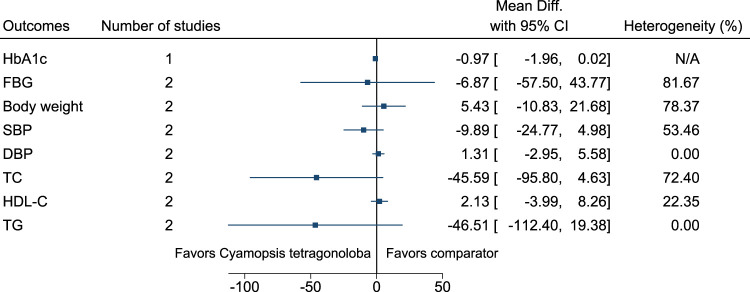
*Cyamopsis tetragonoloba*—Summary forest plot.

Figure 16 shows the summary forest plot for *Cyamopsis tetragonoloba*. (Uusitupa et al., 1984; Uusitupa et al., 1989).

#### 5.5.16 *Elettaria cardamomum* (L.) Maton

**FIGURE 17:**
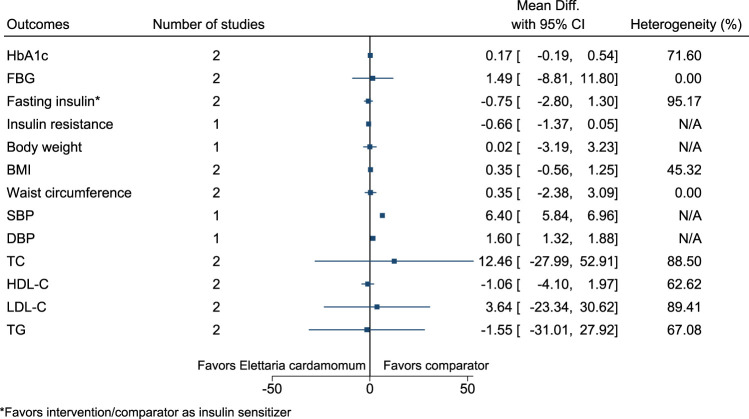
*Elettaria cardamomum* - Summary forest plot.

*Elettaria cardamomum* significantly increased SBP (6 mmHg; 6–7) and DBP (2 mmHg; 1–2) (see Figure 17). (Azimi et al., 2014; Azimi et al., 2016; Aghasi et al., 2019).

#### 5.5.17 *Enicostemma axillare* (Lam.) Raynal (versus OAD)

**FIGURE 18:**
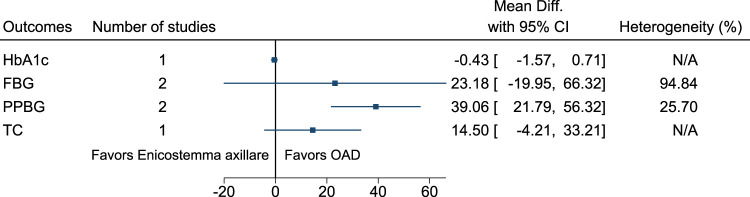
*Enicostemma axillare* (versus OAD)—Summary forest plot.

Compared to *Enicostemma axillare*, OAD significantly reduced PPBG (−39 mg/dl; −56 to −22) (see Figure 18). (Kumar et al., 2014; Shankarrao et al., 2017).

#### 5.5.18 *Gynostemma pentaphyllum* (Thunb.) Makino

**FIGURE 19:**
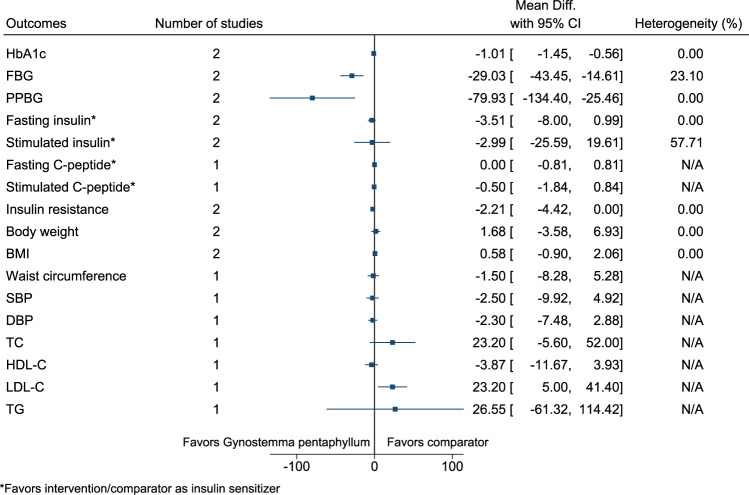
*Gynostemma pentaphyllum*—Summary forest plot.

*Gynostemma pentaphyllum* significantly reduced HbA1c (−1%; −1.5 to −0.6), FBG (−29 mg/dl; −43 to −15), and PPBG (−80 mg/dl; −134 to −25), and increased LDL-C (23 mg/dl; 5–41) (see Figure 19). (Huyen et al., 2010; Huyen et al., 2012).

#### 5.5.19 *Ipomoea batatas* (L.) Lam.

**FIGURE 20:**
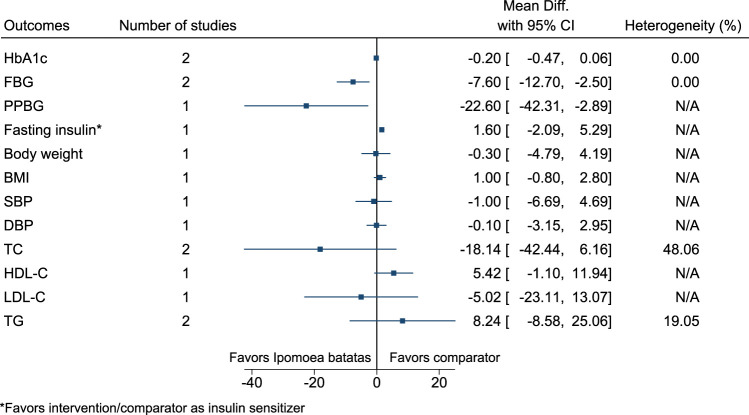
*Ipomoea batatas*—Summary forest plot.

*Ipomoea batatas* significantly reduced FBG (−8 mg/dl; −13 to −3) and PPBG (−23 mg/dl; −42 to −3) (see Figure 20). (Ludvik et al., 2004; Ludvik et al., 2008).

#### 5.5.20 *Juglans regia* L.

**FIGURE 21:**
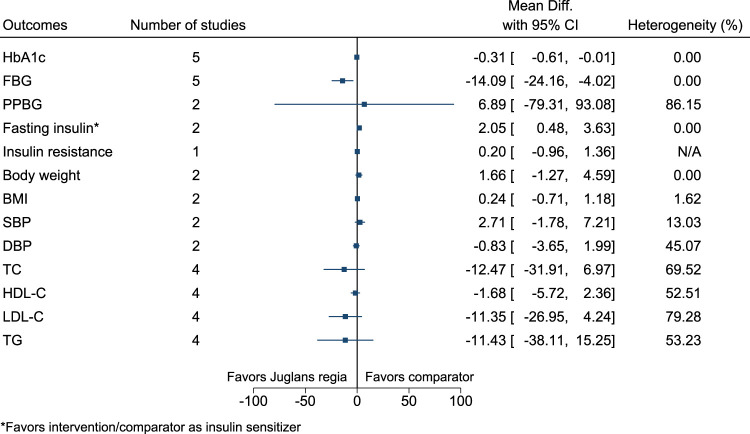
*Juglans regia*—Summary forest plot.

*Juglans regia* significantly reduced FBG (−14 mg/dl; −24 to −4) (see Figure 21). (Hosseini et al., 2014a; Hosseini et al., 2014b; Zibaeenezhad et al., 2016; Abdoli et al., 2017; Zibaeenezhad et al., 2017; Rabiei et al., 2018).

#### 5.5.21 *Momordica charantia* L.

**FIGURE 22:**
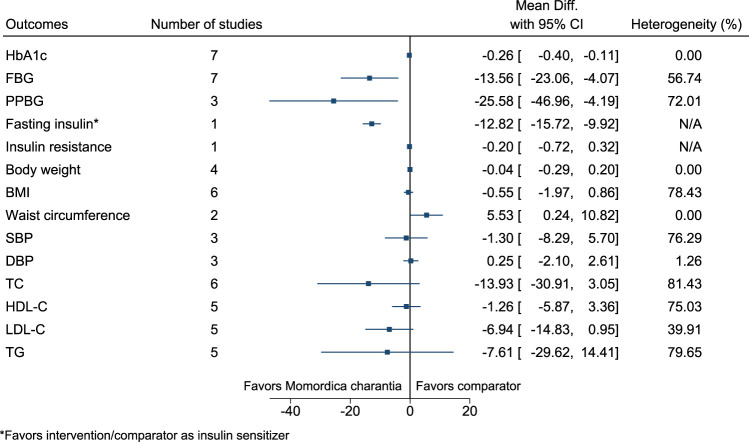
*Momordica charantia*—Summary forest plot.

*Momordica charantia* significantly reduced HbA1c (−0.3%; −0.4 to −0.1), FBG (−14 mg/dl; −23 to −4), PPBG (−26 mg/dl; −47 to −4), and fasting insulin (as insulin sensitizer; −13 mIU/L; −16 to −10) (see Figure 22). (Dans et al., 2007; Zänker et al., 2012; Trakoon-osot et al., 2013; Suthar et al., 2016a; Cortez-Navarrete et al., 2018; Kumari et al., 2018; Amini et al., 2020; Kim et al., 2020) In the country subgroup analysis for HbA1c and FBG, no statistically significant difference was found between subgroups (*p* = 0.25 and *p* = 0.14, respectively).

#### 5.5.22 *Momordica charantia* L. (versus OAD)

**FIGURE 23:**
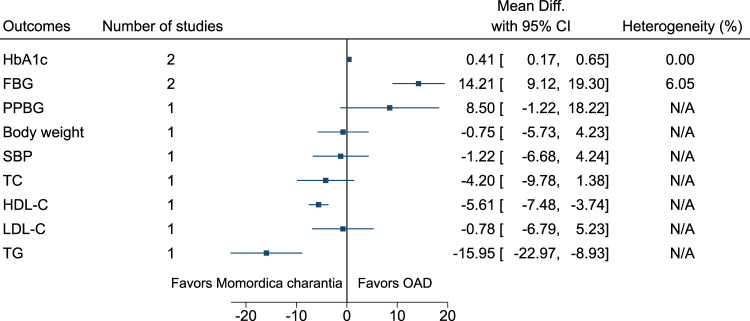
*Momordica charantia* (versus OAD)—Summary forest plot.

Compared to *Momordica charantia*, OAD significantly reduced HbA1c (−0.4%; −0.7 to −0.2) and FBG (−14 mg/dl; −19 to −9). Compared to OAD, *Momordica charantia* significantly increased HDL-C (6 mg/dl; 4–7) and reduced TG (−16 mg/dl; −23 to −9) (see Figure 23). (Inayat U Rahman et al., 2015; Suthar et al., 2016b).

#### 5.5.23 *Nigella sativa* L.

**FIGURE 24:**
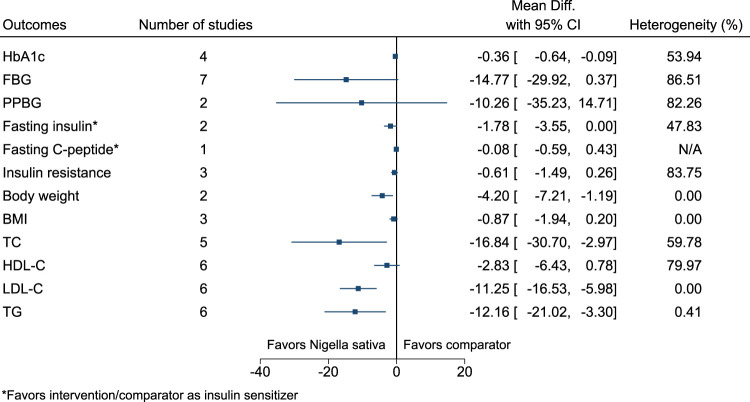
*Nigella sativa*—Summary forest plot.

*Nigella sativa* significantly reduced HbA1c (−0.4%; −0.6 to −0.1), body weight (−4.2 kg; −7.2 to −1.2), TC (−17 mg/dl; −31 to −3), LDL-C (−11 mg/dl; −17 to −6), and TG (−12 mg/dl; −21 to −3) (see Figure 24). (Najmi et al., 2012; Hosseini et al., 2013; Hadi et al., 2015; Heshmati et al., 2015; Kaatabi et al., 2015; Kooshki et al., 2020; Jangjo-Borazjani et al., 2021).

#### 5.5.24 *Plantago ovata* Forssk.

**FIGURE 25:**
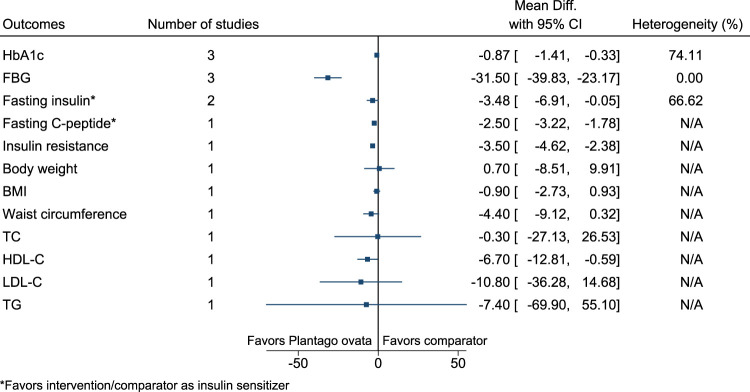
*Plantago ovata*—Summary forest plot.

*Plantago ovata* significantly reduced HbA1c (−0.9%; −1.4 to −0.3), FBG (−32 mg/dl; −40 to −23), fasting C-peptide (as insulin sensitizer; −2.5 ng/ml; −3.22 to −1.78), and insulin resistance (−3.5; −4.6 to −2.4), and increased HDL-C (7 mg/dl; 1–13) (see Figure 25). (Ziai et al., 2005; Feinglos et al., 2013; Abutair et al., 2016).

#### 5.5.25 *Portulaca oleracea* L.

**FIGURE 26:**
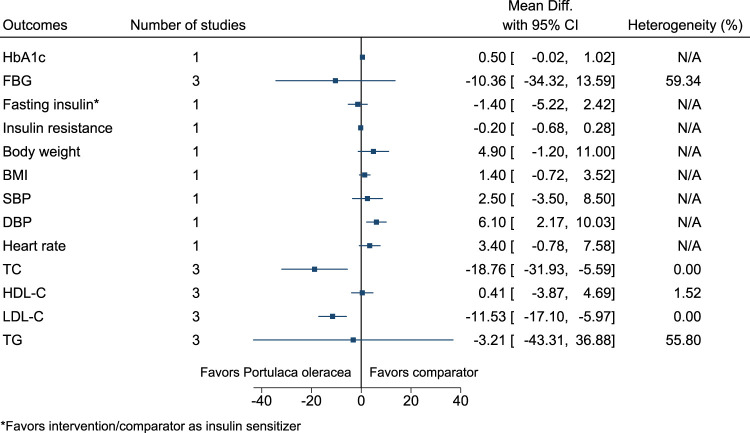
*Portulaca oleracea*—Summary forest plot.

*Portulaca oleracea* significantly reduced TC (−19 mg/dl; −32 to −6) and LDL-C (−12 mg/dl; −17 to −6), and increased DBP (6 mmHg; 2–10) (see Figure 26). (Farzanegi, 2014; Dehghan et al., 2016; Wainstein et al., 2016).

#### 5.5.26 *Pterocarpus marsupium* Roxb. (versus OAD)

**FIGURE 27:**
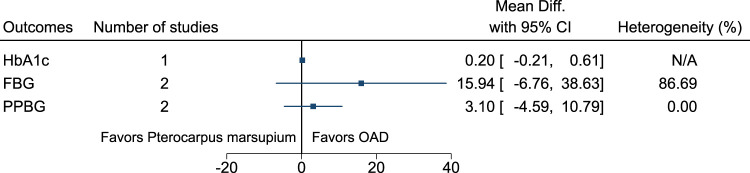
*Pterocarpus marsupium* (versus OAD)—Summary forest plot.

Figure 27 shows the summary forest plot for *Pterocarpus marsupium* (versus OAD). (Hariharan et al., 2005; Maurya et al., 2017).

#### 5.5.27 *Punica granatum* L.

**FIGURE 28:**
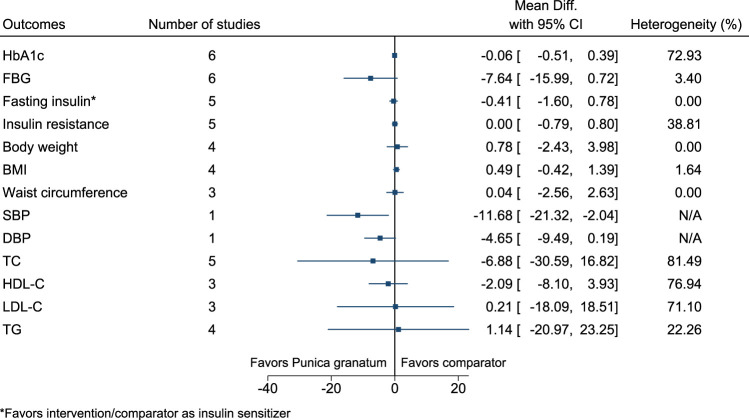
*Punica granatum*—Summary forest plot.

*Punica granatum* significantly reduced SBP (−12 mmHg; −21 to −2) (see Figure 28). (Babaeian et al., 2013; Sohrab et al., 2014; Sohrab et al., 2015; Faghihimani et al., 2016; Khajebishak et al., 2019a; Khajebishak et al., 2019b; Grabež et al., 2020; Hashemi et al., 2020).

#### 5.5.28 *Sesamum indicum* L.

**FIGURE 29:**
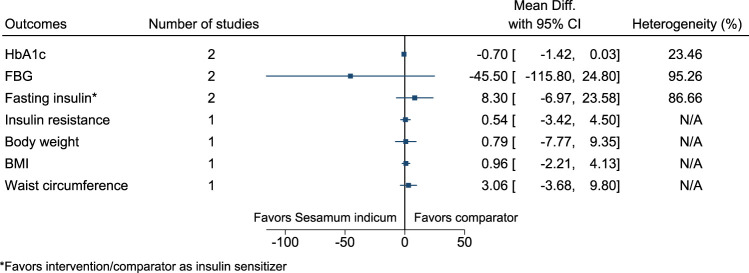
*Sesamum indicum*—Summary forest plot.

Figure 29 shows the summary forest plot for *Sesamum indicum*. (Mohammad Shahi et al., 2017; Aslam et al., 2018).

#### 5.5.29 Shilajit

**FIGURE 30:**
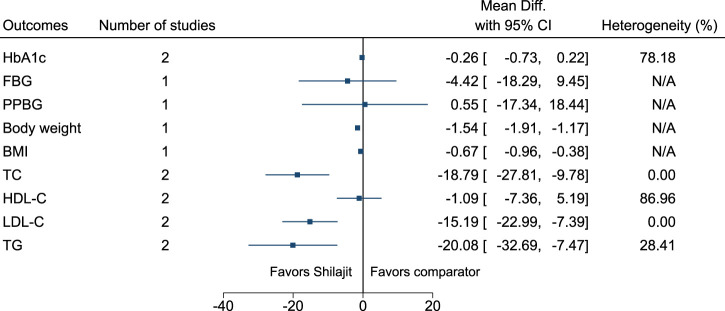
Shilajit—Summary forest plot.

Shilajit significantly reduced body weight (−1.5 kg; −1.9 to −1.2), BMI (−0.7 kg/m^2^; −1 to −0.4), TC (−19 mg/dl; −28 to −10), LDL-C (−15 mg/dl; −23 to −7), and TG (−20 mg/dl; −33 to −7) (see Figure 30). (Narasimha Raju and Sharma, 2016; Niranjan et al., 2016).

#### 5.5.30 *Syzygium cumini* (L.) Skeels

**FIGURE 31:**
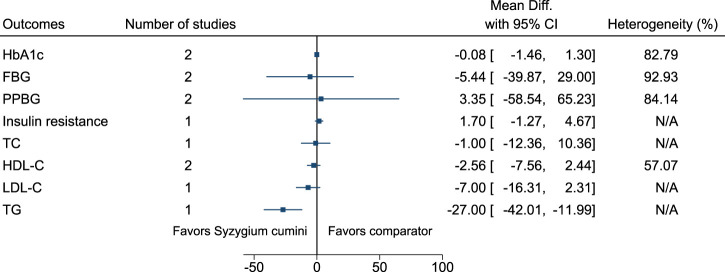
*Syzygium cumini*—Summary forest plot.

*Syzygium cumini* significantly reduced TG (−27 mg/dl; −42 to −12) (see Figure 31). (Sahana et al., 2010; Sidana et al., 2016; Sidana et al., 2017).

#### 5.5.31 *Tinospora cordifolia* (Willd.) Hook.f. & Thomson

**FIGURE 32:**
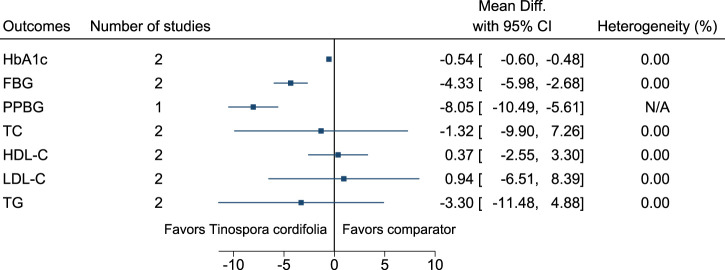
*Tinospora cordifolia*—Summary forest plot.

*Tinospora cordifolia* significantly reduced HbA1c (−0.5%; −0.6 to −0.5), FBG (−4 mg/dl; −6 to −3), and PPBG (−8 mg/dl; −10 to −6) (see Figure 32). (Balasubramaniam et al., 2010; Mishra et al., 2015; Roy et al., 2015).

#### 5.5.32 *Trigonella foenum-graecum* L.

**FIGURE 33:**
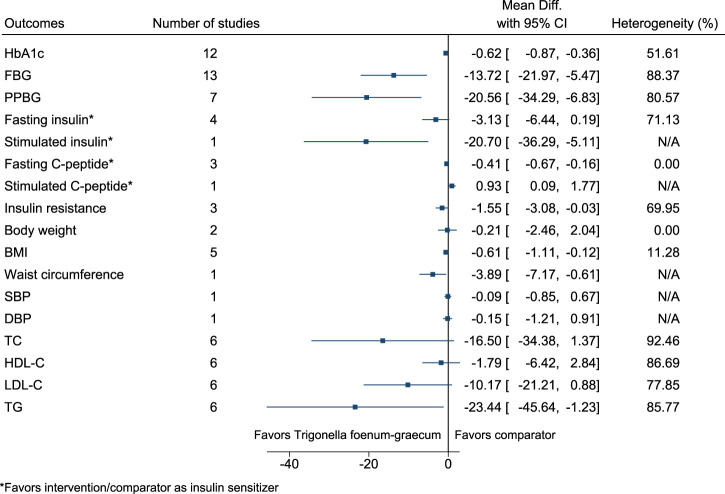
*Trigonella foenum-graecum*—Summary forest plot.

*Trigonella foenum-graecum* significantly reduced HbA1c (−0.6%; −0.9 to −0.4), FBG (−14 mg/dl; −22 to −5), PPBG (−21 mg/dl; −34 to −7), stimulated insulin (as insulin sensitizer; −21 mIU/L; −36 to −5), fasting C-peptide (as insulin sensitizer; −0.41 ng/ml; −0.67 to −0.16), BMI (−0.6 kg/m^2^; −1.1 to −0.1), waist circumference (−4 cm; −7 to −1), and TG (−23 mg/dl; −46 to −1), and increased stimulated C-peptide (as insulin sensitizer; 0.93 ng/ml; 0.09–1.77) (see Figure 33). (Gupta et al., 2001; Lu et al., 2008; Yaheya and Ismail, 2009; Ansari et al., 2011; Rafraf et al., 2014; Suchitra and Parthasarathy, 2015; Kaur et al., 2016; Singh et al., 2016; Verma et al., 2016; Ranade and Mudgalkar, 2017; Gholaman and Gholami, 2018; Kandhare et al., 2018; Hassani et al., 2019; Hota et al., 2019; Rashid et al., 2019; Hadi et al., 2020) Publication bias was detected in the funnel plot for HbA1c but not for FBG. In the funnel plot for HbA1c, there was an absence of smaller sized studies showing a larger positive effect, which could imply the effect may be larger than that reported from the meta-analysis. In the sensitivity analysis, *Trigonella foenum-graecum* significantly reduced HbA1c and FBG even after excluding a publication that was not peer-reviewed. In the country subgroup analysis for HbA1c, no statistically significant difference was found between subgroups (*p* = 0.44). However, for FBG, a statistically significant difference was found between subgroups (*p* < 0.001). In the comparator subgroup analysis for HbA1c and FBG, a statistically significant difference was found between subgroups (*p* = 0.04 and *p* < 0.001, respectively).

#### 5.5.33 *Trigonella foenum-graecum* L. (versus OAD)

**FIGURE 34:**
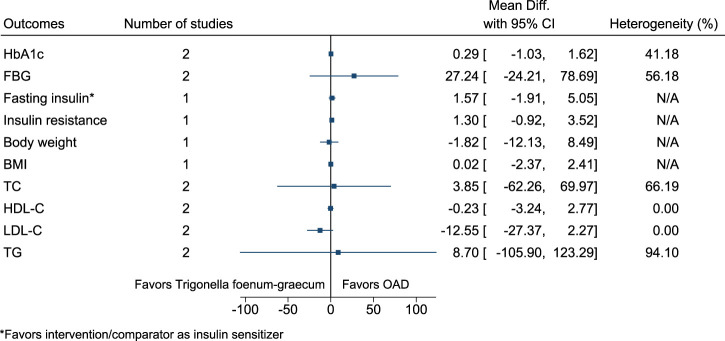
*Trigonella foenum-graecum* (versus OAD)—Summary forest plot.

Figure 34 shows the summary forest plot for *Trigonella foenum-graecum* (versus OAD). (Singh et al., 2016; Najdi et al., 2019).

#### 5.5.34 *Urtica dioica* L.

**FIGURE 35:**
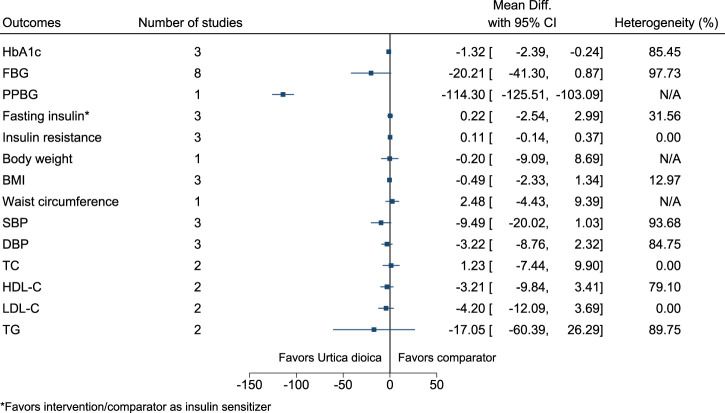
*Urtica dioica*—Summary forest plot.

*Urtica dioica* significantly reduced HbA1c (−1.3%; −2.4 to −0.2) and PPBG (−114 mg/dl; −126 to −103) (see Figure 35). (Namazi et al., 2011; Esfanjani et al., 2012a; Esfanjani et al., 2012b; Kianbakht et al., 2013; Khajeh-Mehrizi et al., 2014; Dabagh and Nikbakht, 2016; Hassani et al., 2016; Dadvar et al., 2017; Ghalavand et al., 2017; Korani et al., 2017; Mohammadnia and Hassani, 2017).

#### 5.5.35 *Zingiber officinale* Roscoe

**FIGURE 36:**
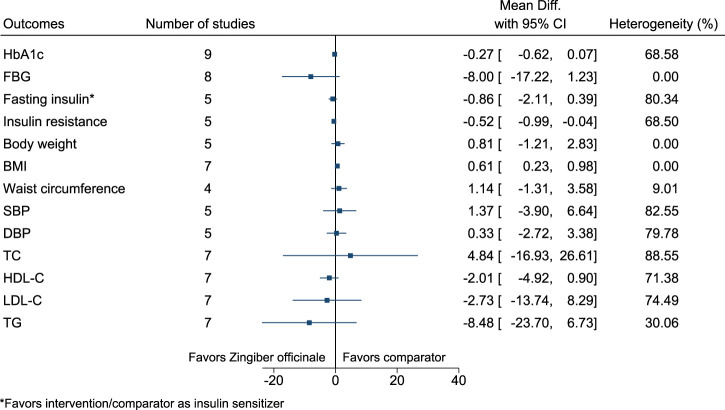
*Zingiber officinale*—Summary forest plot.

*Zingiber officinale* significantly increased BMI (0.6 kg/m^2^; 0.2–1) (see Figure 36). (Mahluji et al., 2013; Arablou et al., 2014a; Arablou et al., 2014b; Azimi et al., 2014; Mozaffari-Khosravi et al., 2014; Shidfar et al., 2015; Azimi et al., 2016; Arzati et al., 2017; Talaei et al., 2017b; Mohammadi et al., 2017; Talaei et al., 2018; Zarezadeh et al., 2018; Mohammadi and Avandi, 2019; Carvalho et al., 2020; Gholinezhad et al., 2020).

The following 65 Ayurvedic medicines, administered either as a single medicine or in combination with other Ayurvedic medicines, could not be included in any meta-analyses due to being assessed in single studies: *Abelmoschus esculentus* (L.) Moench, *Acacia Senegal* (L.) Willd., *Acalypha indica* L., *Allium cepa* L., Asanadi Ghana Vati, AYUBES, *Berberis aristata* DC, BGR-34, Bilvadi Churna, *Capparis spinosa* L., CardiPro, *Cichorium intybus* L., Cogent db, *Convolvulus prostratus* Forssk., Darvyadi Kwatha, DCBT 2345, Diabetea tea, *Eclipta prostrata* (L.) L., *Emblica officinalis* Gaertn., combination of *Emblica officinalis* and *Withania somnifera* (L.) Dunal, Herbal combination, *Hibiscus sabdariffa* L., Hyponidd, Inolter, Kalpit, Khadira-Kramuka Kashaya Ghanavati, Kiratadi Churna, *Linum usitatissimum* L., Lodhradi Kashaya Ghana Vati, Madhumeha Nashini Gutika, Madhumehari Vati, Mamajjaka Ghana Vati, *Mangifera indica* L., Mehagni, *Murraya koenigii* (L.) Spreng., *Musa sapientum* O.Kuntze, Mustadi Kwatha Ghana Vati, combination of Naga Bhasma and Nishamalaki, combination of *Nigella sativa* and *Trigonella foenum-graecum*, Nisha Katakadi Kashaya, Nishamalaki, combination of Nishamalaki and *Hordeum vulgare* L., combination of Nishamalaki and Shilajit, *Ocimum tenuiflorum* L., Pancreas tonic, *Phyllanthus amarus* Schumach. & Thonn., Polyherbal formulation, Salasaradi Kashaya, Swarnamakshika Bhasma, Talapotaka Churna, Tejashiladi Vati, *Terminalia chebula* Retz., combination of *Tinospora cordifolia* and *Azadirachta indica*, *Tribulus terrestris* L., combination of *Trigonella foenum-graecum* and *Ocimum tenuiflorum*, Trikatu Gutika, *Triticum aestivum* L., Trivanga Bhasma, *Vernonia cinerea* (L.) Less., Vidangadi Yoga, Vijaysaradi Ghana Vati, *Withania coagulans* (Stocks) Dunal, *Withania somnifera*, Yashad Bhasma, and *Ziziphus mauritiana* Lam. (See Supplementary Appendix S7 for 89 comparisons of Ayurvedic medicines which could not be included in meta-analyses).

### 5.6 Safety of Interventions

Supplementary Appendix S8 reports the adverse events and dropouts/withdrawals/discontinued interventions due to adverse events in the included studies. Adverse events were not reported in many studies. If reported, these were mostly none to mild and predominately related to the gastrointestinal tract. However, in the majority of cases, the relationship between the intervention and adverse events was not provided.

## 6 Discussions

Beneficial effects of several Ayurvedic medicines on T2DM-related outcomes, including blood glucose, were found. The reduction in HbA1c of at least 0.3% or 0.4% is considered to be clinically meaningful, (US Food and Drug Administration, 2008) and a number of Ayurvedic medicines were found to bring such reduction such as *Aegle marmelos* (L.) Corrêa, *Boswellia serrata* Roxb., *Gynostemma pentaphyllum* (Thunb.) Makino, *Momordica charantia* L., *Nigella sativa* L., *Plantago ovata* Forssk., *Tinospora cordifolia* (Willd.) Hook.f. & Thomson, *Trigonella foenum-graecum* L., and *Urtica dioica* L. compared to control. Similarly, FBG was reduced by 4–56 mg/dl for a range of Ayurvedic medicines. However, the majority of studies did not assess HRQoL, an important patient-reported outcome. Adverse events were not reported in many studies. If reported, these were mostly none to mild and predominantly related to the gastrointestinal tract. The findings are consistent with several systematic reviews conducted on single herbs as well as Ayurveda as a whole system. (Hardy et al., 2001; Yeh et al., 2003; Shekelle et al., 2005; Nahas and Moher, 2009; Shojaii et al., 2011; Sridharan et al., 2011; Suksomboon et al., 2011; Akilen et al., 2012; Allen et al., 2013; Gibb et al., 2015; Namazi et al., 2019; Peter et al., 2019; Jalali et al., 2020; Jamali et al., 2020) It should be noted that the majority of these Ayurvedic medicines are already in use in many countries, and many are used as dietary ingredients such as spices or foods. In many countries, Ayurvedic medicines are available over-the-counter (which includes online shopping) and are considered as dietary supplements. (Chattopadhyay et al., 2020b) Ayurveda is now recognized in 17 countries, including in and beyond South Asia. (Press Information Bureau, 2021) The integration of Ayurveda and Western medicine has been done in India. (Priya and Shweta, 2010) Many single herbs included in this review are not restricted to Ayurveda but are also used in other traditional therapies around the world such as Iranian traditional medicine and traditional Chinese medicine. Similarly, many traditional therapies use multi-ingredient medicines such as Unani (Graeco-Arabic), Siddha (from Southern part of India), traditional Chinese medicine, and Russian traditional medicine. (Chattopadhyay and Bochenek, 2008; Li et al., 2014; Shikov et al., 2021).

The Cochrane systematic review was conducted a decade ago and focused on multiherbal formulations and Ayurveda as a whole system and excluded single herbs and their extracts. (Sridharan et al., 2011) In this review, classical and proprietary Ayurvedic medicines in any form were included (containing plant- as well as mineral-origin ingredients–single or in combination). Many Ayurveda experts and Ayurvedic practitioners may view the inclusion of herb extracts and proprietary Ayurvedic medicines in this review as a deviation from the classical style of management. However, in reality, many Ayurvedic practitioners prescribe, and many people consume these types of medicines. Similarly, Ayurveda experts and Ayurvedic practitioners may view the exclusion of Ayurvedic detoxifying and purifying therapies (e.g., Panchakarma) in this review as a deviation from the classical style of management. However, considering the feasibility and practicality of the review work, these were beyond the scope of this review. The focus of this review was on Ayurvedic medicines, as these are commonly prescribed and consumed. Having said that, the future review work should consider synthesizing evidence on the effectiveness and safety of such complex interventions.

Overall, the methodology was not adequately reported in the studies, and this resulted in poor methodological quality scoring. The assessment of methodological quality is subjective to a large extent, and the reviewers were strict. For example, other systematic reviewers might be satisfied if the differences between study arms in terms of their follow-up are described. However, the reviewers went a step further and were expecting these to be analyzed. The strictness is one of the reasons for poor methodological quality scoring. In addition, if the funding statement was provided, it was mostly brief and difficult to determine the level of support received from pharmaceutical companies. For example, it was not always clear if a pharmaceutical company provided the trial medicines for free or these were purchased. Therefore, it was difficult to determine the funding bias i.e., the tendency of a study to support the interests of the study’s financial sponsor.

This systematic review has several strengths and weaknesses. To the best of our knowledge, this was the first comprehensive systematic review on any traditional medicine including Ayurveda, and which included a wide range of classical and proprietary Ayurvedic medicines in any form (containing plant- as well as mineral-origin ingredients–single or in combination). A large number of sources and databases were searched, without any date or language restrictions. An extensively robust methodology was followed to conduct this review. Although the information provided in the studies was at times confusing, the reviewers tried their best to extract the correct information. Two independent reviewers were involved throughout the process, and a third reviewer cross-checked everything. The kappa statistic was within the acceptable range i.e., 0.67 and 0.57 for the title and abstract screening and full text screening, respectively. A multi-disciplinary team was involved in the review, with expertise in Ayurveda, medicinal plants, diabetes, systematic reviewing, and statistics. The initial plan was to perform a range of sensitivity and subgroup analyses. However, many of these could not be performed. For example, complete information on commercial funding or other support was needed to correctly conduct the sensitivity analysis by excluding commercially funded studies. However, it was unclear in 43% of RCTs. Some of the issues were outside the scope of this review, and the evidence should be synthesized in future reviews to decide the optimal option. For example, comparison of two or more drug manufacturing processes, forms or timings of administration, doses, and Anupans of the same Ayurvedic medicine. Similarly, many other factors, such as patients’ age, sex, ethnicity, lifestyle (e.g., diet and physical activity), chronicity and severity of T2DM, and comorbidities, can influence the outcomes. However, due to limited data for some comparison, we were not able to conduct separate subgroup analyses to explore the potential influence of these factors. Apart from the issues highlighted in this review, there are some basic issues which were beyond its scope and need addressing as well. For example, standardization and quality control of Ayurvedic medicines. (Chattopadhyay and Bochenek, 2008)

## 7 Conclusion

The current evidence suggests the benefit of a range of Ayurvedic medicines in improving glycemic control in T2DM patients. This evidence base and more specifically the Summary of Findings table will be used to develop a clinical guideline for managing T2DM by Ayurvedic practitioners. Given the limitations of the available evidence and to strengthen the evidence base, high-quality RCTs should be conducted and reported.

## 8 Recommendations for Practice

There is a need to develop, evaluate, and implement need-sensitive, evidence-based interventions to manage T2DM among different population groups. (Chattopadhyay and Leonardi-Bee, 2021) Based on the best available evidence as found in this systematic review and more specifically the Summary of Findings table, a clinical guideline for managing T2DM by Ayurvedic practitioners will now be systematically developed. The next steps will be guided by the GRADE approach, the United Kingdom’s National Institute for Health and Care Excellence (NICE) manual for developing guidelines and the Appraisal of Guidelines for Research and Evaluation (AGREE) II instrument. (Brouwers et al., 2010; Schünemann et al., 2013; National Institute for Health and Care Excellence, 2014) Clinical guidelines for managing T2DM by Ayurvedic practitioners exist. (Central Council for Research in Ayurvedic Sciences, 2011; Ministry of Ayush, 2016; Ministry of Health and Family Welfare, 2016; Central Council for Research in Ayurvedic Sciences, 2017; Central Council for Research in Ayurvedic Sciences and Directorate General of Health Services, 2018) However, their quality is questionable due to several factors, including whether the best available evidence was considered. Low-quality clinical guidelines can lead to the use of ineffective interventions, inefficient use of scarce resources, and most importantly, harm to patients. (Institute of Medicine, 2011) The goal is to deter the usage of Ayurvedic medicines of no, minimal, or questionable value and promote the usage of effective and safe Ayurvedic medicines.

## 9 Recommendations for Research

In terms of recommendation for research, the future RCTs must address the following issues to strengthen the evidence base: 1) true randomization should be used to assign participants to study arms; 2) allocation concealment should be done to conceal the allocation to study arms; 3) adequate blinding should be done after considering who will be blinded and how blinding will be performed; 4) if placebo is used then it should be identical to the intervention, not only look and texture wise but also taste and smell wise, so that it is hard to differentiate from the intervention; 5) study arms should be treated identically other than the intervention of interest; 6) outcomes should be measured in the same way for study arms; 7) the sample size should be calculated based on appropriate components, such as primary outcome and *minimum* clinically important difference, and the trial should be adequately powered; 8) data should be analysed properly (including ITT analysis and post-intervention difference in outcomes between study arms); and 9) differences between study arms in terms of their follow-up should be described as well as analyzed. It should be noted that comprehensive and transparent reporting of the trial methods, funding, and results is important after designing and conducting a high-quality RCT. Clinical trial registration and prior publication of the trial protocol are also important. In order to update the clinical guideline in the future, robust RCTs should be conducted not only on those Ayurvedic medicines that were included in meta-analyses but also on those on which meta-analysis was not possible. It would be beneficial if the comparison is also made with the standard treatment (i.e., OAD). T2DM is a chronic condition, and so, long-term studies are needed to determine the effectiveness and safety of these Ayurvedic medicines, especially in terms of preventing macro- or micro-vascular complications of T2DM and death. In addition, a sufficiently detailed description of the botanical and phytochemical aspects of the study material should be provided.

## Data Availability

The original contributions presented in the study are included in the article/Supplementary Material, further inquiries can be directed to the corresponding author.
