# Supplementary material for: Effectiveness and Safety of Ayurvedic Medicines in Type 2 Diabetes Mellitus Management: A Systematic Review and Meta-Analysis
Source: Front Pharmacol. 2022 Jun 8;13:821810. doi: 10.3389/fphar.2022.821810 (PMC9213670; doi:10.3389/fphar.2022.821810)
Supplement: Supplementary file 1 [file DataSheet1.zip › Supplementary Material/Summary of Findings Table.pdf]

Summary of Findings

***Aegle marmelos* (L.) Corrêa compared to comparator for T2DM management**

Patient or population: T2DM management
Intervention: *Aegle marmelos*
Comparison: comparator (i.e., no medicine, no additional medicine, or placebo)

Outcomes	Absolute effect (95% CI)	№ of participants (studies)	Certainty of the evidence (GRADE)	Comments
HbA1c	MD **1.6 % lower** (3 lower to 0.3 lower)	210 (2 RCTs)	⊕⊕⊕◯ MODERATE [a]	
FBG	MD **56 mg/dL lower** (104 lower to 9 lower)	228 (3 RCTs)	⊕◯◯◯ VERY LOW [a,b,c]	
HRQoL - not reported	-	-	-	
Adverse events	1 study reported 0 adverse event. Exact details were not provided in the other 2 studies.	(3 RCTs)	⊕⊕◯◯ LOW [a,d]	

CI: Confidence interval; **MD:** Mean difference

GRADE Working Group grades of evidence
High certainty: We are very confident that the true effect lies close to that of the estimate of the effect
Moderate certainty: We are moderately confident in the effect estimate: The true effect is likely to be close to the estimate of the effect, but there is a possibility that it is substantially different
Low certainty: Our confidence in the effect estimate is limited: The true effect may be substantially different from the estimate of the effect
Very low certainty: We have very little confidence in the effect estimate: The true effect is likely to be substantially different from the estimate of effect

Explanations
a. Small sample size.
b. Attrition bias.
c. Statistical heterogeneity.
d. Selective outcome reporting.

***Allium sativum* L. compared to comparator for T2DM management**

Patient or population: T2DM management
Intervention: *Allium sativum*
Comparison: comparator (i.e., no medicine, no additional medicine, or placebo)

Outcomes	Absolute effect (95% CI)	№ of participants (studies)	Certainty of the evidence (GRADE)	Comments
HbA1c	MD **0.4 % lower** (0.9 lower to 0.1 higher)	272 (3 RCTs)	⊕⊕◯◯ LOW [a,b]	
FBG	MD **1 mg/dL lower** (14 lower to 11 higher)	316 (4 RCTs)	⊕◯◯◯ VERY LOW [a,b]	
HRQoL - not reported	-	-	-	
Adverse events	1 study reported 0 adverse event in the intervention arm. 2 studies reported gastrointestinal tract related adverse event.	(3 RCTs)	⊕⊕◯◯ LOW [b,c]	

CI: Confidence interval; **MD:** Mean difference

GRADE Working Group grades of evidence
High certainty: We are very confident that the true effect lies close to that of the estimate of the effect
Moderate certainty: We are moderately confident in the effect estimate: The true effect is likely to be close to the estimate of the effect, but there is a possibility that it is substantially different
Low certainty: Our confidence in the effect estimate is limited: The true effect may be substantially different from the estimate of the effect
Very low certainty: We have very little confidence in the effect estimate: The true effect is likely to be substantially different from the estimate of effect

Explanations
a. Statistical heterogeneity.
b. Small sample size.
c. Selective outcome reporting.

***Aloe vera* L. compared to comparator for T2DM management**

Patient or population: T2DM management
Intervention: *Aloe vera*
Comparison: comparator (i.e., no medicine, no additional medicine, or placebo)

Outcomes	Absolute effect (95% CI)	№ of participants (studies)	Certainty of the evidence (GRADE)	Comments
HbA1c	MD **0.9 % lower** (2.1 lower to 0.3 higher)	137 (3 RCTs)	⊕◯◯◯ VERY LOW [a,b,c]	
FBG	MD **11 mg/dL lower** (32 lower to 10 higher)	197 (4 RCTs)	⊕◯◯◯ VERY LOW [a,b,c]	
HRQoL - not reported	-	-	-	
Adverse events	1 study reported 0 adverse event. The other study reported 0 adverse event in the intervention arm and gastrointestinal tract related adverse event in the comparator arm.	(2 RCTs)	⊕⊕◯◯ LOW [c,d]	

CI: Confidence interval; **MD:** Mean difference

GRADE Working Group grades of evidence
High certainty: We are very confident that the true effect lies close to that of the estimate of the effect
Moderate certainty: We are moderately confident in the effect estimate: The true effect is likely to be close to the estimate of the effect, but there is a possibility that it is substantially different
Low certainty: Our confidence in the effect estimate is limited: The true effect may be substantially different from the estimate of the effect
Very low certainty: We have very little confidence in the effect estimate: The true effect is likely to be substantially different from the estimate of effect

Explanations
a. Attrition bias.
b. Statistical heterogeneity.
c. Small sample size.
d. Selective outcome reporting.

***Anethum graveolens* L. compared to comparator for T2DM management**				
**Patient or population**: T2DM management<br>**Intervention**: *Anethum graveolens*<br>**Comparison**: comparator (i.e., no medicine, no additional medicine, or placebo)				
Outcomes	Absolute effect<br>(95% CI)	№ of participants<br>(studies)	Certainty of the evidence<br>(GRADE)	Comments
HbA1c - not reported	-	-	-	
FBG	MD **12 mg/dL lower**<br>(30 lower to 7 higher)	94<br>(2 RCTs)	⊕◯◯◯<br>VERY LOW [a,b]	
HRQoL - not reported	-	-	-	
Adverse events - not reported	-	-	-	
**CI:** Confidence interval; **MD:** Mean difference				
**GRADE Working Group grades of evidence**<br>**High certainty:** We are very confident that the true effect lies close to that of the estimate of the effect<br>**Moderate certainty:** We are moderately confident in the effect estimate: The true effect is likely to be close to the estimate of the effect, but there is a possibility that it is substantially different<br>**Low certainty:** Our confidence in the effect estimate is limited: The true effect may be substantially different from the estimate of the effect<br>**Very low certainty:** We have very little confidence in the effect estimate: The true effect is likely to be substantially different from the estimate of effect				

Explanations
a. Attrition bias.
b. Small sample size.

***Azadirachta indica* A.Juss. compared to comparator for T2DM management**

Patient or population: T2DM management
Intervention: *Azadirachta indica*
Comparison: comparator (i.e., no medicine, no additional medicine, or placebo)

Outcomes	Absolute effect (95% CI)	№ of participants (studies)	Certainty of the evidence (GRADE)	Comments
HbA1c	MD **1 % lower** (1.2 lower to 0.8 lower)	78 (1 RCT)	⊕◯◯◯ VERY LOW [a,b]	
FBG	MD **8 mg/dL lower** (13 lower to 4 lower)	118 (2 RCTs)	⊕⊕⊕◯ MODERATE [b]	
HRQoL - not reported	-	-	-	
Adverse events	1 study reported gastrointestinal tract related adverse event.	(1 RCT)	⊕◯◯◯ VERY LOW [b,c]	

CI: Confidence interval; **MD:** Mean difference

GRADE Working Group grades of evidence
High certainty: We are very confident that the true effect lies close to that of the estimate of the effect
Moderate certainty: We are moderately confident in the effect estimate: The true effect is likely to be close to the estimate of the effect, but there is a possibility that it is substantially different
Low certainty: Our confidence in the effect estimate is limited: The true effect may be substantially different from the estimate of the effect
Very low certainty: We have very little confidence in the effect estimate: The true effect is likely to be substantially different from the estimate of effect

Explanations
a. Attrition bias.
b. Small sample size.
c. Selective outcome reporting.

***Boswellia serrata* Roxb. compared to comparator for T2DM management**

Patient or population: T2DM management
Intervention: *Boswellia serrata*
Comparison: comparator (i.e., no medicine, no additional medicine, or placebo)

Outcomes	Absolute effect (95% CI)	№ of participants (studies)	Certainty of the evidence (GRADE)	Comments
HbA1c	MD **0.5 % lower** (0.7 lower to 0.4 lower)	127 (2 RCTs)	⊕⊕⊕◯ MODERATE [a]	
FBG	MD **24 mg/dL lower** (28 lower to 21 lower)	127 (2 RCTs)	⊕⊕⊕◯ MODERATE [a]	
HRQoL - not reported	-	-	-	
Adverse events	2 studies reported 0 adverse event (this includes 1 study which reported 0 adverse event in the intervention arm).	(2 RCTs)	⊕⊕◯◯ LOW [a,b]	

CI: Confidence interval; **MD:** Mean difference

GRADE Working Group grades of evidence
High certainty: We are very confident that the true effect lies close to that of the estimate of the effect
Moderate certainty: We are moderately confident in the effect estimate: The true effect is likely to be close to the estimate of the effect, but there is a possibility that it is substantially different
Low certainty: Our confidence in the effect estimate is limited: The true effect may be substantially different from the estimate of the effect
Very low certainty: We have very little confidence in the effect estimate: The true effect is likely to be substantially different from the estimate of effect

Explanations
a. Small sample size.
b. Selective outcome reporting.

***Camellia sinensis* (L.) Kuntze compared to comparator for T2DM management**				
**Patient or population**: T2DM management<br>**Intervention**: *Camellia sinensis*<br>**Comparison**: comparator (i.e., no medicine, no additional medicine, or placebo)				
Outcomes	Absolute effect (95% CI)	№ of participants (studies)	Certainty of the evidence (GRADE)	Comments
HbA1c	MD **0.1 % lower** (0.4 lower to 0.2 higher)	317 (6 RCTs)	⊕⊕◯◯ LOW [a,b]	
FBG	MD **11 mg/dL lower** (26 lower to 5 higher)	237 (4 RCTs)	⊕⊕◯◯ LOW [a,b]	
HRQoL - Physical health	SMD **0.01 SD higher** (0.36 lower to 0.38 higher)	108 (2 RCTs)	⊕⊕◯◯ LOW [a,b]	
HRQoL - Psychological	SMD **0.12 SD higher** (0.25 lower to 0.5 higher)	108 (2 RCTs)	⊕⊕◯◯ LOW [a,b]	
HRQoL - Social relationships	SMD **0.02 SD higher** (0.35 lower to 0.39 higher)	108 (2 RCTs)	⊕⊕◯◯ LOW [a,b]	
HRQoL - Environment	SMD **0.11 SD lower** (0.62 lower to 0.41 higher)	108 (2 RCTs)	⊕⊕◯◯ LOW [a,b]	
Adverse events	3 studies mainly reported gastrointestinal tract related adverse events and hypoglycemia (this includes 1 study which reported 0 adverse event in the comparator arm).	(3 RCTs)	⊕⊕◯◯ LOW [b,c]	
**CI:** Confidence interval; **MD:** Mean difference; **SMD:** Standardised mean difference				
**GRADE Working Group grades of evidence**<br>**High certainty:** We are very confident that the true effect lies close to that of the estimate of the effect<br>**Moderate certainty:** We are moderately confident in the effect estimate: The true effect is likely to be close to the estimate of the effect, but there is a possibility that it is substantially different<br>**Low certainty:** Our confidence in the effect estimate is limited: The true effect may be substantially different from the estimate of the effect<br>**Very low certainty:** We have very little confidence in the effect estimate: The true effect is likely to be substantially different from the estimate of effect				

Explanations

a. Attrition bias.
b. Small sample size.
c. Selective outcome reporting.

Cinnamomum aromaticum Nees compared to comparator for T2DM management				
**Patient or population**: T2DM management<br>**Intervention**: *Cinnamomum aromaticum*<br>**Comparison**: comparator (i.e., no medicine, no additional medicine, or placebo)				
Outcomes	Absolute effect (95% CI)	№ of participants (studies)	Certainty of the evidence (GRADE)	Comments
HbA1c	MD **0.2 % lower** (0.5 lower to 0.1 higher)	794 (10 RCTs)	⊕⊕⊕◯ MODERATE [a]	
FBG	MD **1 mg/dL higher** (6 lower to 9 higher)	692 (9 RCTs)	⊕⊕⊕⊕ HIGH	
HRQoL - not reported	-	-	-	
Adverse events	3 studies reported 0 adverse event (this includes 1 study which reported 0 adverse event in the intervention arm). 2 studies reported gastrointestinal tract and skin related adverse events (this includes 1 study which reported 0 adverse event in the intervention arm and 1 study which reported 0 adverse event in the comparator arm).	(5 RCTs)	⊕⊕⊕◯ MODERATE [b]	
**CI:** Confidence interval; **MD:** Mean difference				
**GRADE Working Group grades of evidence**<br>**High certainty:** We are very confident that the true effect lies close to that of the estimate of the effect<br>**Moderate certainty:** We are moderately confident in the effect estimate: The true effect is likely to be close to the estimate of the effect, but there is a possibility that it is substantially different<br>**Low certainty:** Our confidence in the effect estimate is limited: The true effect may be substantially different from the estimate of the effect<br>**Very low certainty:** We have very little confidence in the effect estimate: The true effect is likely to be substantially different from the estimate of effect				

Explanations
a. Publication bias detected in the funnel plot.
b. Selective outcome reporting.

***Cinnamomum verum* J.Presl compared to comparator for T2DM management**

Patient or population: T2DM management
Intervention: *Cinnamomum verum*
Comparison: comparator (i.e., no medicine, no additional medicine, or placebo)

Outcomes	Absolute effect (95% CI)	№ of participants (studies)	Certainty of the evidence (GRADE)	Comments
HbA1c	MD **0.1 % lower** (0.5 lower to 0.3 higher)	403 (6 RCTs)	⊕⊕◯◯ LOW [a,b]	
FBG	MD **11 mg/dL lower** (19 lower to 3 lower)	403 (6 RCTs)	⊕⊕⊕◯ MODERATE [a]	
HRQoL - not reported	-	-	-	
Adverse events	4 studies reported 0 adverse event (this includes 1 study which reported 0 adverse event in the intervention arm). 1 study reported an adverse event (this study reported 0 adverse event in the intervention arm).	(5 RCTs)	⊕⊕◯◯ LOW [c,d]	

CI: Confidence interval; **MD:** Mean difference

GRADE Working Group grades of evidence
High certainty: We are very confident that the true effect lies close to that of the estimate of the effect
Moderate certainty: We are moderately confident in the effect estimate: The true effect is likely to be close to the estimate of the effect, but there is a possibility that it is substantially different
Low certainty: Our confidence in the effect estimate is limited: The true effect may be substantially different from the estimate of the effect
Very low certainty: We have very little confidence in the effect estimate: The true effect is likely to be substantially different from the estimate of effect

Explanations
a. Attrition bias.
b. Statistical heterogeneity.
c. Selective outcome reporting.
d. Small sample size.

***Citrullus colocynthis* (L.) Schrad. compared to comparator for T2DM management**				
**Patient or population**: T2DM management<br>**Intervention**: *Citrullus colocynthis*<br>**Comparison**: comparator (i.e., no medicine, no additional medicine, or placebo)				
Outcomes	Absolute effect (95% CI)	№ of participants (studies)	Certainty of the evidence (GRADE)	Comments
HbA1c	MD **0.2 % lower** (0.7 lower to 0.4 higher)	120 (2 RCTs)	⨁⨁⨁◯ MODERATE [a]	
FBG	MD **3 mg/dL lower** (18 lower to 12 higher)	120 (2 RCTs)	⨁⨁⨁◯ MODERATE [a]	
HRQoL - not reported	-	-	-	
Adverse events	1 study reported 0 adverse event in the intervention arm. The other study reported gastrointestinal tract related adverse event.	(2 RCTs)	⨁⨁◯◯ LOW [a,b]	
**CI:** Confidence interval; **MD:** Mean difference				

GRADE Working Group grades of evidence
High certainty: We are very confident that the true effect lies close to that of the estimate of the effect
Moderate certainty: We are moderately confident in the effect estimate: The true effect is likely to be close to the estimate of the effect, but there is a possibility that it is substantially different
Low certainty: Our confidence in the effect estimate is limited: The true effect may be substantially different from the estimate of the effect
Very low certainty: We have very little confidence in the effect estimate: The true effect is likely to be substantially different from the estimate of effect

Explanations
a. Small sample size.
b. Selective outcome reporting.

Coccinia grandis **(L.) Voigt compared to comparator for T2DM management**				
**Patient or population**: T2DM management<br>**Intervention**: *Coccinia grandis*<br>**Comparison**: comparator (i.e., no medicine, no additional medicine, or placebo)				
Outcomes	Absolute effect (95% CI)	№ of participants (studies)	Certainty of the evidence (GRADE)	Comments
HbA1c	MD **0.5 % lower** (1.1 lower to 0)	217 (2 RCTs)	⨁⨁◯◯ LOW [a,b]	
FBG	MD **22 mg/dL lower** (25 lower to 19 lower)	217 (2 RCTs)	⨁⨁⨁◯ MODERATE [b]	
HRQoL - not reported	-	-	-	
Adverse events	1 study reported 0 adverse event. The other study mainly reported gastrointestinal tract related adverse events and hypoglycemia.	(2 RCTs)	⨁⨁◯◯ LOW [b,c]	
**CI:** Confidence interval; **MD:** Mean difference				

GRADE Working Group grades of evidence
High certainty: We are very confident that the true effect lies close to that of the estimate of the effect
Moderate certainty: We are moderately confident in the effect estimate: The true effect is likely to be close to the estimate of the effect, but there is a possibility that it is substantially different
Low certainty: Our confidence in the effect estimate is limited: The true effect may be substantially different from the estimate of the effect
Very low certainty: We have very little confidence in the effect estimate: The true effect is likely to be substantially different from the estimate of effect

Explanations
a. Statistical heterogeneity.
b. Small sample size.
c. Selective outcome reporting.

Crocus sativus L. compared to comparator for T2DM management

Patient or population: T2DM management
Intervention: _Crocus sativus_
Comparison: comparator (i.e., no medicine, no additional medicine, or placebo)

Outcomes	Absolute effect (95% CI)	№ of participants (studies)	Certainty of the evidence (GRADE)	Comments
HbA1c	MD **0.2 % higher** (0.1 lower to 0.4 higher)	277 (4 RCTs)	⊕⊕◯◯ LOW [a,b]	
FBG	MD **9 mg/dL lower** (26 lower to 8 higher)	334 (5 RCTs)	⊕⊕◯◯ LOW [a,b]	
HRQoL - not reported	-	-	-	
Adverse events	1 study reported 0 adverse event in the intervention arm.	(1 RCT)	⊕◯◯◯ VERY LOW [b,c]	

CI: Confidence interval; **MD:** Mean difference

GRADE Working Group grades of evidence
High certainty: We are very confident that the true effect lies close to that of the estimate of the effect
Moderate certainty: We are moderately confident in the effect estimate: The true effect is likely to be close to the estimate of the effect, but there is a possibility that it is substantially different
Low certainty: Our confidence in the effect estimate is limited: The true effect may be substantially different from the estimate of the effect
Very low certainty: We have very little confidence in the effect estimate: The true effect is likely to be substantially different from the estimate of effect

Explanations
a. Attrition bias.
b. Small sample size.
c. Selective outcome reporting.

***Cuminum cyminum* L. compared to comparator for T2DM management**				
**Patient or population**: T2DM management<br>**Intervention**: *Cuminum cyminum*<br>**Comparison**: comparator (i.e., no medicine, no additional medicine, or placebo)				
Outcomes	Absolute effect (95% CI)	№ of participants (studies)	Certainty of the evidence (GRADE)	Comments
HbA1c	MD **1.5 % lower** (3.7 lower to 0.7 higher)	140 (2 RCTs)	⊕◯◯◯ VERY LOW [a,b]	
FBG	MD **14 mg/dL lower** (35 lower to 6 higher)	290 (2 RCTs)	⊕⊕◯◯ LOW [a,b]	
HRQoL - not reported	-	-	-	
Adverse events	1 study reported 0 adverse event in the intervention arm.	(1 RCT)	⊕◯◯◯ VERY LOW [b,c]	

CI: Confidence interval; **MD:** Mean difference

GRADE Working Group grades of evidence
High certainty: We are very confident that the true effect lies close to that of the estimate of the effect
Moderate certainty: We are moderately confident in the effect estimate: The true effect is likely to be close to the estimate of the effect, but there is a possibility that it is substantially different
Low certainty: Our confidence in the effect estimate is limited: The true effect may be substantially different from the estimate of the effect
Very low certainty: We have very little confidence in the effect estimate: The true effect is likely to be substantially different from the estimate of effect

Explanations
a. Statistical heterogeneity.
b. Small sample size.
c. Selective outcome reporting.

***Curcuma longa* L. compared to comparator for T2DM management**

Patient or population: T2DM management
Intervention: *Curcuma longa*
Comparison: comparator (i.e., no medicine, no additional medicine, or placebo)

Outcomes	Absolute effect (95% CI)	№ of participants (studies)	Certainty of the evidence (GRADE)	Comments
HbA1c	MD **0.2 % lower** (0.7 lower to 0.4 higher)	593 (6 RCTs)	⊕⊕◯◯ LOW [a,b]	
FBG	MD **10 mg/dL lower** (15 lower to 5 lower)	593 (6 RCTs)	⊕⊕⊕◯ MODERATE [a]	
HRQoL - not reported	-	-	-	
Adverse events	3 studies reported 0 adverse event (this includes 1 study which reported 0 adverse event in the comparator arm). 3 studies mainly reported gastrointestinal tract related adverse events.	(6 RCTs)	⊕⊕⊕◯ MODERATE [c]	

CI: Confidence interval; **MD:** Mean difference

GRADE Working Group grades of evidence
High certainty: We are very confident that the true effect lies close to that of the estimate of the effect
Moderate certainty: We are moderately confident in the effect estimate: The true effect is likely to be close to the estimate of the effect, but there is a possibility that it is substantially different
Low certainty: Our confidence in the effect estimate is limited: The true effect may be substantially different from the estimate of the effect
Very low certainty: We have very little confidence in the effect estimate: The true effect is likely to be substantially different from the estimate of effect

Explanations
a. Attrition bias.
b. Statistical heterogeneity.
c. Selective outcome reporting.

***Cyamopsis tetragonoloba* (L.) Taub. compared to comparator for T2DM management**

Patient or population: T2DM management
Intervention: *Cyamopsis tetragonoloba*
Comparison: comparator (i.e., no medicine, no additional medicine, or placebo)

Outcomes	Absolute effect (95% CI)	№ of participants (studies)	Certainty of the evidence (GRADE)	Comments
HbA1c	MD **1 % lower** (2 lower to 0)	39 (1 RCT)	⨁⨁◯◯ LOW [a]	
FBG	MD **7 mg/dL lower** (58 lower to 44 higher)	56 (2 RCTs)	⨁◯◯◯ VERY LOW [a,b]	
HRQoL - not reported	-	-	-	
Adverse events	1 study reported gastrointestinal tract related adverse events. Exact details were not provided in the other study.	(2 RCTs)	⨁◯◯◯ VERY LOW [a,c]	

CI: Confidence interval; **MD:** Mean difference

GRADE Working Group grades of evidence
High certainty: We are very confident that the true effect lies close to that of the estimate of the effect
Moderate certainty: We are moderately confident in the effect estimate: The true effect is likely to be close to the estimate of the effect, but there is a possibility that it is substantially different
Low certainty: Our confidence in the effect estimate is limited: The true effect may be substantially different from the estimate of the effect
Very low certainty: We have very little confidence in the effect estimate: The true effect is likely to be substantially different from the estimate of effect

Explanations
a. Small sample size.
b. Statistical heterogeneity.
c. Selective outcome reporting.

***Elettaria cardamomum* (L.) Maton compared to comparator for T2DM management**

Patient or population: T2DM management
Intervention: *Elettaria cardamomum*
Comparison: comparator (i.e., no medicine, no additional medicine, or placebo)

Outcomes	Absolute effect (95% CI)	№ of participants (studies)	Certainty of the evidence (GRADE)	Comments
HbA1c	MD **0.2 % higher** (0.2 lower to 0.5 higher)	164 (2 RCTs)	⊕⊕⊕◯ MODERATE [a]	
FBG	MD **1 mg/dL higher** (9 lower to 12 higher)	164 (2 RCTs)	⊕⊕⊕◯ MODERATE [a]	
HRQoL - not reported	-	-	-	
Adverse events	1 study reported 0 adverse event in the intervention arm. The other study reported adverse events (this study reported 0 adverse event in the comparator arm).	(2 RCTs)	⊕⊕⊕◯ MODERATE [a]	

CI: Confidence interval; **MD:** Mean difference

GRADE Working Group grades of evidence
High certainty: We are very confident that the true effect lies close to that of the estimate of the effect
Moderate certainty: We are moderately confident in the effect estimate: The true effect is likely to be close to the estimate of the effect, but there is a possibility that it is substantially different
Low certainty: Our confidence in the effect estimate is limited: The true effect may be substantially different from the estimate of the effect
Very low certainty: We have very little confidence in the effect estimate: The true effect is likely to be substantially different from the estimate of effect

Explanations
a. Small sample size.

***Enicostemma axillare* (Lam.) Raynal compared to OAD for T2DM management**

Patient or population: T2DM management
Intervention: *Enicostemma axillare*
Comparison: OAD

Outcomes	Absolute effect (95% CI)	№ of participants (studies)	Certainty of the evidence (GRADE)	Comments
HbA1c	MD **0.4 % lower** (1.6 lower to 0.7 higher)	32 (1 RCT)	⊕◯◯◯ VERY LOW [a,b]	
FBG	MD **23 mg/dL higher** (20 lower to 66 higher)	101 (2 RCTs)	⊕◯◯◯ VERY LOW [b,c]	
HRQoL - not reported	-	-	-	
Adverse events	1 study reported 0 adverse event in the intervention arm.	(1 RCT)	⊕◯◯◯ VERY LOW [b,d]	

CI: Confidence interval; **MD:** Mean difference

GRADE Working Group grades of evidence
High certainty: We are very confident that the true effect lies close to that of the estimate of the effect
Moderate certainty: We are moderately confident in the effect estimate: The true effect is likely to be close to the estimate of the effect, but there is a possibility that it is substantially different
Low certainty: Our confidence in the effect estimate is limited: The true effect may be substantially different from the estimate of the effect
Very low certainty: We have very little confidence in the effect estimate: The true effect is likely to be substantially different from the estimate of effect

Explanations
a. Attrition bias.
b. Small sample size.
c. Statistical heterogeneity.
d. Selective outcome reporting.

***Gynostemma pentaphyllum* (Thunb.) Makino compared to comparator for T2DM management**

Patient or population: T2DM management
Intervention: *Gynostemma pentaphyllum*
Comparison: comparator (i.e., no medicine, no additional medicine, or placebo)

Outcomes	Absolute effect (95% CI)	№ of participants (studies)	Certainty of the evidence (GRADE)	Comments
HbA1c	MD **1 % lower** (1.5 lower to 0.6 lower)	49 (2 RCTs)	⊕⊕◯◯ LOW [a]	
FBG	MD **29 mg/dL lower** (43 lower to 15 lower)	49 (2 RCTs)	⊕⊕◯◯ LOW [a]	
HRQoL - not reported	-	-	-	
Adverse events	2 studies reported 0 adverse event.	(2 RCTs)	⊕⊕◯◯ LOW [a]	

CI: Confidence interval; **MD:** Mean difference

GRADE Working Group grades of evidence
High certainty: We are very confident that the true effect lies close to that of the estimate of the effect
Moderate certainty: We are moderately confident in the effect estimate: The true effect is likely to be close to the estimate of the effect, but there is a possibility that it is substantially different
Low certainty: Our confidence in the effect estimate is limited: The true effect may be substantially different from the estimate of the effect
Very low certainty: We have very little confidence in the effect estimate: The true effect is likely to be substantially different from the estimate of effect

Explanations
a. Small sample size.

***Ipomoea batatas* (L.) Lam. compared to comparator for T2DM management**

Patient or population: T2DM management
Intervention: *Ipomoea batatas*
Comparison: comparator (i.e., no medicine, no additional medicine, or placebo)

Outcomes	Absolute effect (95% CI)	№ of participants (studies)	Certainty of the evidence (GRADE)	Comments
HbA1c	MD **0.2 % lower** (0.5 lower to 0.1 higher)	122 (2 RCTs)	⊕⊕⊕◯ MODERATE [a]	
FBG	MD **8 mg/dL lower** (13 lower to 3 lower)	122 (2 RCTs)	⊕⊕⊕◯ MODERATE [a]	
HRQoL - not reported	-	-	-	
Adverse events	Exact details were not provided in 2 studies but mainly reported gastrointestinal tract related adverse events.	(2 RCTs)	⊕⊕◯◯ LOW [a,b]	

CI: Confidence interval; **MD:** Mean difference

GRADE Working Group grades of evidence
High certainty: We are very confident that the true effect lies close to that of the estimate of the effect
Moderate certainty: We are moderately confident in the effect estimate: The true effect is likely to be close to the estimate of the effect, but there is a possibility that it is substantially different
Low certainty: Our confidence in the effect estimate is limited: The true effect may be substantially different from the estimate of the effect
Very low certainty: We have very little confidence in the effect estimate: The true effect is likely to be substantially different from the estimate of effect

Explanations
a. Small sample size.
b. Selective outcome reporting.

***Juglans regia* L. compared to comparator for T2DM management**				

Patient or population: T2DM management
Intervention: *Juglans regia*
Comparison: comparator (i.e., no medicine, no additional medicine, or placebo)

Outcomes	Absolute effect (95% CI)	№ of participants (studies)	Certainty of the evidence (GRADE)	Comments
HbA1c	MD **0.3 % lower** (0.6 lower to 0)	285 (5 RCTs)	⊕⊕◯◯ LOW [a,b]	
FBG	MD **14 mg/dL lower** (24 lower to 4 lower)	285 (5 RCTs)	⊕⊕◯◯ LOW [a,b]	
HRQoL - not reported	-	-	-	
Adverse events	2 studies reported 0 adverse event (this includes 1 study which reported 0 adverse event in the intervention arm). 2 studies mainly reported gastrointestinal tract related adverse events (this includes 1 study which reported 0 adverse event in the comparator arm).	(4 RCTs)	⊕⊕◯◯ LOW [b,c]	

CI: Confidence interval; **MD:** Mean difference

GRADE Working Group grades of evidence
High certainty: We are very confident that the true effect lies close to that of the estimate of the effect
Moderate certainty: We are moderately confident in the effect estimate: The true effect is likely to be close to the estimate of the effect, but there is a possibility that it is substantially different
Low certainty: Our confidence in the effect estimate is limited: The true effect may be substantially different from the estimate of the effect
Very low certainty: We have very little confidence in the effect estimate: The true effect is likely to be substantially different from the estimate of effect

Explanations
a. Attrition bias.
b. Small sample size.
c. Selective outcome reporting.

***Momordica charantia* L. compared to comparator for T2DM management**				
**Patient or population**: T2DM management<br>**Intervention**: *Momordica charantia*<br>**Comparison**: comparator (i.e., no medicine, no additional medicine, or placebo)				
Outcomes	Absolute effect (95% CI)	№ of participants (studies)	Certainty of the evidence (GRADE)	Comments
HbA1c	MD **0.3 % lower** (0.4 lower to 0.1 lower)	404 (7 RCTs)	⊕⊕⊕◯ MODERATE [a]	
FBG	MD **14 mg/dL lower** (23 lower to 4 lower)	360 (7 RCTs)	⊕⊕◯◯ LOW [a,b]	
HRQoL - not reported	-	-	-	
Adverse events	1 study reported 0 adverse event. 5 studies mainly reported gastrointestinal tract related adverse events (this includes 1 study which reported 0 adverse event in the comparator arm).	(6 RCTs)	⊕⊕◯◯ LOW [b,c]	
**CI:** Confidence interval; **MD:** Mean difference				

GRADE Working Group grades of evidence
High certainty: We are very confident that the true effect lies close to that of the estimate of the effect
Moderate certainty: We are moderately confident in the effect estimate: The true effect is likely to be close to the estimate of the effect, but there is a possibility that it is substantially different
Low certainty: Our confidence in the effect estimate is limited: The true effect may be substantially different from the estimate of the effect
Very low certainty: We have very little confidence in the effect estimate: The true effect is likely to be substantially different from the estimate of effect

Explanations
a. Attrition bias.
b. Small sample size.
c. Selective outcome reporting.

***Momordica charantia* L. compared to OAD for T2DM management**

Patient or population: T2DM management
Intervention: *Momordica charantia*
Comparison: OAD

Outcomes	Absolute effect (95% CI)	№ of participants (studies)	Certainty of the evidence (GRADE)	Comments
HbA1c	MD **0.4 % higher** (0.2 higher to 0.7 higher)	201 (2 RCTs)	⊕⊕◯◯ LOW [a,b]	
FBG	MD **14 mg/dL higher** (9 higher to 19 higher)	198 (2 RCTs)	⊕⊕◯◯ LOW [a,b]	
HRQoL - not reported	-	-	-	
Adverse events	1 study mainly reported gastrointestinal tract related adverse events. Exact details were not provided in the other study.	(2 RCTs)	⊕⊕◯◯ LOW [b,c]	

CI: Confidence interval; **MD:** Mean difference

GRADE Working Group grades of evidence
High certainty: We are very confident that the true effect lies close to that of the estimate of the effect
Moderate certainty: We are moderately confident in the effect estimate: The true effect is likely to be close to the estimate of the effect, but there is a possibility that it is substantially different
Low certainty: Our confidence in the effect estimate is limited: The true effect may be substantially different from the estimate of the effect
Very low certainty: We have very little confidence in the effect estimate: The true effect is likely to be substantially different from the estimate of effect

Explanations
a. Attrition bias.
b. Small sample size.
c. Selective outcome reporting.

***Nigella sativa* L. compared to comparator for T2DM management**

Patient or population: T2DM management
Intervention: *Nigella sativa*
Comparison: comparator (i.e., no medicine, no additional medicine, or placebo)

Outcomes	Absolute effect (95% CI)	№ of participants (studies)	Certainty of the evidence (GRADE)	Comments
HbA1c	MD **0.4 % lower** (0.6 lower to 0.1 lower)	325 (4 RCTs)	⊕⊕⊕◯ MODERATE [a]	
FBG	MD **15 mg/dL lower** (30 lower to 0)	458 (7 RCTs)	⊕⊕⊕◯ MODERATE [b]	
HRQoL - not reported	-	-	-	
Adverse events	3 studies reported 0 adverse event (this includes 1 study which reported 0 adverse event in the intervention arm). 2 studies reported gastrointestinal tract related adverse events (this includes 1 study which reported 0 adverse event in the comparator arm and exact details were not provided in the other study).	(5 RCTs)	⊕⊕◯◯ LOW [a,c]	

CI: Confidence interval; **MD:** Mean difference

GRADE Working Group grades of evidence
High certainty: We are very confident that the true effect lies close to that of the estimate of the effect
Moderate certainty: We are moderately confident in the effect estimate: The true effect is likely to be close to the estimate of the effect, but there is a possibility that it is substantially different
Low certainty: Our confidence in the effect estimate is limited: The true effect may be substantially different from the estimate of the effect
Very low certainty: We have very little confidence in the effect estimate: The true effect is likely to be substantially different from the estimate of effect

Explanations
a. Small sample size.
b. Statistical heterogeneity.
c. Selective outcome reporting.

Plantago ovata Forssk. compared to comparator for T2DM management

Patient or population: T2DM management
Intervention: *Plantago ovata*
Comparison: comparator (i.e., no medicine, no additional medicine, or placebo)

Outcomes	Absolute effect (95% CI)	№ of participants (studies)	Certainty of the evidence (GRADE)	Comments
HbA1c	MD **0.9 % lower** (1.4 lower to 0.3 lower)	106 (3 RCTs)	⊕⊕◯◯ LOW [a,b]	
FBG	MD **32 mg/dL lower** (40 lower to 23 lower)	106 (3 RCTs)	⊕⊕◯◯ LOW [a,b]	
HRQoL - not reported	-	-	-	
Adverse events	1 study mainly reported gastrointestinal tract related adverse events. Exact details were not provided in the other study but hypoglycemia was reported.	(2 RCTs)	⊕◯◯◯ VERY LOW [b,c]	

CI: Confidence interval; **MD:** Mean difference

GRADE Working Group grades of evidence
High certainty: We are very confident that the true effect lies close to that of the estimate of the effect
Moderate certainty: We are moderately confident in the effect estimate: The true effect is likely to be close to the estimate of the effect, but there is a possibility that it is substantially different
Low certainty: Our confidence in the effect estimate is limited: The true effect may be substantially different from the estimate of the effect
Very low certainty: We have very little confidence in the effect estimate: The true effect is likely to be substantially different from the estimate of effect

Explanations
a. Attrition bias.
b. Small sample size.
c. Selective outcome reporting.

***Portulaca oleracea* L. compared to comparator for T2DM management**

Patient or population: T2DM management
Intervention: *Portulaca oleracea*
Comparison: comparator (i.e., no medicine, no additional medicine, or placebo)

Outcomes	Absolute effect (95% CI)	№ of participants (studies)	Certainty of the evidence (GRADE)	Comments
HbA1c	MD **0.5 % higher** (0 to 1 higher)	63 (1 RCT)	⊕⊕◯◯ LOW [a]	
FBG	MD **10 mg/dL lower** (34 lower to 14 higher)	123 (3 RCTs)	⊕⊕⊕◯ MODERATE [a]	
HRQoL - not reported	-	-	-	
Adverse events	1 study reported adverse events.	(1 RCT)	⊕◯◯◯ VERY LOW [a,b]	

CI: Confidence interval; **MD:** Mean difference

GRADE Working Group grades of evidence
High certainty: We are very confident that the true effect lies close to that of the estimate of the effect
Moderate certainty: We are moderately confident in the effect estimate: The true effect is likely to be close to the estimate of the effect, but there is a possibility that it is substantially different
Low certainty: Our confidence in the effect estimate is limited: The true effect may be substantially different from the estimate of the effect
Very low certainty: We have very little confidence in the effect estimate: The true effect is likely to be substantially different from the estimate of effect

Explanations
a. Small sample size.
b. Selective outcome reporting.

***Pterocarpus marsupium* Roxb. compared to OAD for T2DM management**

Patient or population: T2DM management
Intervention: *Pterocarpus marsupium*
Comparison: OAD

Outcomes	Absolute effect (95% CI)	№ of participants (studies)	Certainty of the evidence (GRADE)	Comments
HbA1c	MD **0.2 % higher** (0.2 lower to 0.6 higher)	90 (1 RCT)	⨁◯◯◯ VERY LOW [a,b]	
FBG	MD **16 mg/dL higher** (7 lower to 39 higher)	409 (2 RCTs)	⨁⨁◯◯ LOW [a,c]	
HRQoL - not reported	-	-	-	
Adverse events	1 study reported 0 adverse event in the intervention arm. The other study reported gastrointestinal tract related adverse event.	(2 RCTs)	⨁⨁⨁◯ MODERATE [d]	

CI: Confidence interval; **MD:** Mean difference

GRADE Working Group grades of evidence
High certainty: We are very confident that the true effect lies close to that of the estimate of the effect
Moderate certainty: We are moderately confident in the effect estimate: The true effect is likely to be close to the estimate of the effect, but there is a possibility that it is substantially different
Low certainty: Our confidence in the effect estimate is limited: The true effect may be substantially different from the estimate of the effect
Very low certainty: We have very little confidence in the effect estimate: The true effect is likely to be substantially different from the estimate of effect

Explanations
a. Attrition bias.
b. Small sample size.
c. Statistical heterogeneity.
d. Selective outcome reporting.

Punica granatum L. compared to comparator for T2DM management				
**Patient or population**: T2DM management<br>**Intervention**: *Punica granatum*<br>**Comparison**: comparator (i.e., no medicine, no additional medicine, or placebo)				
Outcomes	Absolute effect (95% CI)	№ of participants (studies)	Certainty of the evidence (GRADE)	Comments
HbA1c	MD **0.1 % lower** (0.5 lower to 0.4 higher)	312 (6 RCTs)	⊕⊕◯◯ LOW [a,b]	
FBG	MD **8 mg/dL lower** (16 lower to 1 higher)	312 (6 RCTs)	⊕⊕◯◯ LOW [a,b]	
HRQoL - not reported	-	-	-	
Adverse events	2 studies reported 0 adverse event (this includes 1 study which reported 0 adverse event in the intervention arm). 1 study mainly reported gastrointestinal tract related adverse events.	(3 RCTs)	⊕⊕◯◯ LOW [b,c]	
**CI:** Confidence interval; **MD:** Mean difference				

GRADE Working Group grades of evidence
High certainty: We are very confident that the true effect lies close to that of the estimate of the effect
Moderate certainty: We are moderately confident in the effect estimate: The true effect is likely to be close to the estimate of the effect, but there is a possibility that it is substantially different
Low certainty: Our confidence in the effect estimate is limited: The true effect may be substantially different from the estimate of the effect
Very low certainty: We have very little confidence in the effect estimate: The true effect is likely to be substantially different from the estimate of effect

Explanations
a. Attrition bias.
b. Small sample size.
c. Selective outcome reporting.

***Sesamum indicum* L. compared to comparator for T2DM management**

Patient or population: T2DM management
Intervention: *Sesamum indicum*
Comparison: comparator (i.e., no medicine, no additional medicine, or placebo)

Outcomes	Absolute effect (95% CI)	№ of participants (studies)	Certainty of the evidence (GRADE)	Comments
HbA1c	MD **0.7 % lower** (1.4 lower to 0)	90 (2 RCTs)	⨁◯◯◯ VERY LOW [a,b]	
FBG	MD **46 mg/dL lower** (116 lower to 25 higher)	90 (2 RCTs)	⨁◯◯◯ VERY LOW [a,b,c]	
HRQoL - not reported	-	-	-	
Adverse events - not reported	-	-	-	

CI: Confidence interval; **MD:** Mean difference

GRADE Working Group grades of evidence
High certainty: We are very confident that the true effect lies close to that of the estimate of the effect
Moderate certainty: We are moderately confident in the effect estimate: The true effect is likely to be close to the estimate of the effect, but there is a possibility that it is substantially different
Low certainty: Our confidence in the effect estimate is limited: The true effect may be substantially different from the estimate of the effect
Very low certainty: We have very little confidence in the effect estimate: The true effect is likely to be substantially different from the estimate of effect

Explanations
a. Attrition bias.
b. Small sample size.
c. Statistical heterogeneity.

Shilajit compared to comparator for T2DM management

Patient or population: T2DM management
Intervention: Shilajit
Comparison: comparator (i.e., no medicine, no additional medicine, or placebo)

Outcomes	Absolute effect (95% CI)	№ of participants (studies)	Certainty of the evidence (GRADE)	Comments
HbA1c	MD **0.3 % lower** (0.7 lower to 0.2 higher)	132 (2 RCTs)	⨁⨁◯◯ LOW [a,b]	
FBG	MD **4 mg/dL lower** (18 lower to 9 higher)	92 (1 RCT)	⨁◯◯◯ VERY LOW [b,c]	
HRQoL - not reported	-	-	-	
Adverse events	1 study reported adverse events.	(1 RCT)	⨁◯◯◯ VERY LOW [b,d]	

CI: Confidence interval; **MD:** Mean difference

GRADE Working Group grades of evidence
High certainty: We are very confident that the true effect lies close to that of the estimate of the effect
Moderate certainty: We are moderately confident in the effect estimate: The true effect is likely to be close to the estimate of the effect, but there is a possibility that it is substantially different
Low certainty: Our confidence in the effect estimate is limited: The true effect may be substantially different from the estimate of the effect
Very low certainty: We have very little confidence in the effect estimate: The true effect is likely to be substantially different from the estimate of effect

Explanations
a. Statistical heterogeneity.
b. Small sample size.
c. Attrition bias.
d. Selective outcome reporting.

***Syzygium cumini* (L.) Skeels compared to comparator for T2DM management**

Patient or population: T2DM management
Intervention: *Syzygium cumini*
Comparison: comparator (i.e., no medicine, no additional medicine, or placebo)

Outcomes	Absolute effect (95% CI)	№ of participants (studies)	Certainty of the evidence (GRADE)	Comments
HbA1c	MD **0.1 % lower** (1.5 lower to 1.3 higher)	124 (2 RCTs)	⨁⨁◯◯ LOW [a,b]	
FBG	MD **5 mg/dL lower** (40 lower to 29 higher)	124 (2 RCTs)	⨁◯◯◯ VERY LOW [a,b]	
HRQoL - not reported	-	-	-	
Adverse events - not reported	-	-	-	

CI: Confidence interval; **MD:** Mean difference

GRADE Working Group grades of evidence
High certainty: We are very confident that the true effect lies close to that of the estimate of the effect
Moderate certainty: We are moderately confident in the effect estimate: The true effect is likely to be close to the estimate of the effect, but there is a possibility that it is substantially different
Low certainty: Our confidence in the effect estimate is limited: The true effect may be substantially different from the estimate of the effect
Very low certainty: We have very little confidence in the effect estimate: The true effect is likely to be substantially different from the estimate of effect

Explanations
a. Statistical heterogeneity.
b. Small sample size.

Tinospora cordifolia (Willd.) Hook.f. & Thomson compared to comparator for T2DM management

Patient or population: T2DM management
Intervention: *Tinospora cordifolia*
Comparison: comparator (i.e., no medicine, no additional medicine, or placebo)

Outcomes	Absolute effect (95% CI)	№ of participants (studies)	Certainty of the evidence (GRADE)	Comments
HbA1c	MD **0.5 % lower** (0.6 lower to 0.5 lower)	159 (2 RCTs)	⊕⊕⊕◯ MODERATE [a]	
FBG	MD **4 mg/dL lower** (6 lower to 3 lower)	140 (2 RCTs)	⊕⊕⊕◯ MODERATE [a]	
HRQoL - not reported	-	-	-	
Adverse events	2 studies reported 0 adverse event (this includes 1 study which reported 0 adverse event in the intervention arm).	(2 RCTs)	⊕⊕⊕◯ MODERATE [a]	

CI: Confidence interval; **MD:** Mean difference

GRADE Working Group grades of evidence
High certainty: We are very confident that the true effect lies close to that of the estimate of the effect
Moderate certainty: We are moderately confident in the effect estimate: The true effect is likely to be close to the estimate of the effect, but there is a possibility that it is substantially different
Low certainty: Our confidence in the effect estimate is limited: The true effect may be substantially different from the estimate of the effect
Very low certainty: We have very little confidence in the effect estimate: The true effect is likely to be substantially different from the estimate of effect

Explanations
a. Small sample size.

***Trigonella foenum-graecum* L. compared to comparator for T2DM management**

Patient or population: T2DM management
Intervention: *Trigonella foenum-graecum*
Comparison: comparator (i.e., no medicine, no additional medicine, or placebo)

Outcomes	Absolute effect (95% CI)	№ of participants (studies)	Certainty of the evidence (GRADE)	Comments
HbA1c	MD **0.6 % lower** (0.9 lower to 0.4 lower)	1095 (12 RCTs)	⊕⊕⊕◯ MODERATE [a]	
FBG	MD **14 mg/dL lower** (22 lower to 5 lower)	1069 (13 RCTs)	⊕⊕⊕◯ MODERATE [a,b]	
HRQoL - not reported	-	-	-	
Adverse events	5 studies reported 0 adverse event (this includes 2 studies which reported 0 adverse event in the intervention arm). 5 studies reported different body systems related adverse events but mainly gastrointestinal tract related and hypoglycemia (this includes 1 study which reported 0 adverse event in the comparator arm). Exact details were not provided in 1 study.	(11 RCTs)	⊕⊕⊕◯ MODERATE [c]	

CI: Confidence interval; **MD:** Mean difference

GRADE Working Group grades of evidence
High certainty: We are very confident that the true effect lies close to that of the estimate of the effect
Moderate certainty: We are moderately confident in the effect estimate: The true effect is likely to be close to the estimate of the effect, but there is a possibility that it is substantially different
Low certainty: Our confidence in the effect estimate is limited: The true effect may be substantially different from the estimate of the effect
Very low certainty: We have very little confidence in the effect estimate: The true effect is likely to be substantially different from the estimate of effect

Explanations
a. Publication bias.
b. Statistical heterogeneity.
c. Selective outcome reporting.

***Trigonella foenum-graecum* L. compared to OAD for T2DM management**

Patient or population: T2DM management
Intervention: *Trigonella foenum-graecum*
Comparison: OAD

Outcomes	Absolute effect (95% CI)	№ of participants (studies)	Certainty of the evidence (GRADE)	Comments
HbA1c	MD **0.3 % higher** (1 lower to 1.6 higher)	49 (2 RCTs)	⊕◯◯◯ VERY LOW [a,b]	
FBG	MD **27 mg/dL higher** (24 lower to 79 higher)	49 (2 RCTs)	⊕◯◯◯ VERY LOW [a,b]	
HRQoL - not reported	-	-	-	
Adverse events	2 studies mainly reported gastrointestinal tract related adverse events and hypoglycemia (this includes 1 study which reported 0 adverse event in the comparator arm).	(2 RCTs)	⊕⊕◯◯ LOW [b]	

CI: Confidence interval; **MD:** Mean difference

GRADE Working Group grades of evidence
High certainty: We are very confident that the true effect lies close to that of the estimate of the effect
Moderate certainty: We are moderately confident in the effect estimate: The true effect is likely to be close to the estimate of the effect, but there is a possibility that it is substantially different
Low certainty: Our confidence in the effect estimate is limited: The true effect may be substantially different from the estimate of the effect
Very low certainty: We have very little confidence in the effect estimate: The true effect is likely to be substantially different from the estimate of effect

Explanations
a. Lack of blinding.
b. Small sample size.

Urtica dioica L. compared to comparator for T2DM management				
**Patient or population**: T2DM management<br>**Intervention**: *Urtica dioica*<br>**Comparison**: comparator (i.e., no medicine, no additional medicine, or placebo)				
Outcomes	Absolute effect (95% CI)	№ of participants (studies)	Certainty of the evidence (GRADE)	Comments
HbA1c	MD **1.3 % lower** (2.4 lower to 0.2 lower)	181 (3 RCTs)	⊕◯◯◯ VERY LOW [a,b,c]	
FBG	MD **20 mg/dL lower** (41 lower to 1 higher)	376 (8 RCTs)	⊕◯◯◯ VERY LOW [a,b,c]	
HRQoL - not reported	-	-	-	
Adverse events	1 study reported 0 adverse event. The other study reported skin related adverse event (this study reported 0 adverse event in the comparator arm).	(2 RCTs)	⊕⊕◯◯ LOW [c,d]	
**CI:** Confidence interval; **MD:** Mean difference				
**GRADE Working Group grades of evidence**<br>**High certainty:** We are very confident that the true effect lies close to that of the estimate of the effect<br>**Moderate certainty:** We are moderately confident in the effect estimate: The true effect is likely to be close to the estimate of the effect, but there is a possibility that it is substantially different<br>**Low certainty:** Our confidence in the effect estimate is limited: The true effect may be substantially different from the estimate of the effect<br>**Very low certainty:** We have very little confidence in the effect estimate: The true effect is likely to be substantially different from the estimate of effect				

Explanations
a. Attrition bias.
b. Statistical heterogeneity.
c. Small sample size.
d. Selective outcome reporting.

***Zingiber officinale* Roscoe compared to comparator for T2DM management**

Patient or population: T2DM management
Intervention: *Zingiber officinale*
Comparison: comparator (i.e., no medicine, no additional medicine, or placebo)

Outcomes	Absolute effect (95% CI)	№ of participants (studies)	Certainty of the evidence (GRADE)	Comments
HbA1c	MD **0.3 % lower** (0.6 lower to 0.1 higher)	561 (9 RCTs)	⊕⊕⊕◯ MODERATE [a]	
FBG	MD **8 mg/dL lower** (17 lower to 1 higher)	517 (8 RCTs)	⊕⊕⊕◯ MODERATE [a]	
HRQoL - not reported	-	-	-	
Adverse events	3 studies reported 0 adverse event (this includes 2 studies which reported 0 adverse event in the intervention arm). 2 studies reported gastrointestinal tract related adverse events.	(5 RCTs)	⊕⊕⊕◯ MODERATE [b]	

CI: Confidence interval; **MD:** Mean difference

GRADE Working Group grades of evidence
High certainty: We are very confident that the true effect lies close to that of the estimate of the effect
Moderate certainty: We are moderately confident in the effect estimate: The true effect is likely to be close to the estimate of the effect, but there is a possibility that it is substantially different
Low certainty: Our confidence in the effect estimate is limited: The true effect may be substantially different from the estimate of the effect
Very low certainty: We have very little confidence in the effect estimate: The true effect is likely to be substantially different from the estimate of effect

Explanations
a. Attrition bias.
b. Selective outcome reporting.
