# Supplementary material for: Effectiveness and Safety of Ayurvedic Medicines in Type 2 Diabetes Mellitus Management: A Systematic Review and Meta-Analysis
Source: Front Pharmacol. 2022 Jun 8;13:821810. doi: 10.3389/fphar.2022.821810 (PMC9213670; doi:10.3389/fphar.2022.821810)
Supplement: Supplementary file 1 [file DataSheet1.zip › Supplementary Material/Appendices.pdf]

Appendix S1: Search strategies.

MEDLINE (Ovid) (1946-16 January 2021): 2042 records

1. exp Diabetes mellitus, type 2/
2. (Type* adj3 ("2" or "II" or two*) adj3 (diabete* or diabetic*)).tw.
3. ((Late or maturit* or adult* or slow*) adj3 onset* adj3 (diabete* or diabetic*)).tw.
4. ((Ketosis-resistant* or stable*) adj3 (diabete* or diabetic*)).tw.
5. ((Non-insulin* or non insulin* or noninsulin*) adj3 depend* adj3 (diabete* or diabetic*)).tw.
6. (NIDDM or T2DM or T2D).tw.
7. Prameha.mp. [mp=title, abstract, original title, name of substance word, subject heading word, floating sub-heading word, keyword heading word, organism supplementary concept word, protocol supplementary concept word, rare disease supplementary concept word, unique identifier, synonyms]
8. Madhumeha.mp. [mp=title, abstract, original title, name of substance word, subject heading word, floating sub-heading word, keyword heading word, organism supplementary concept word, protocol supplementary concept word, rare disease supplementary concept word, unique identifier, synonyms]
9. or/1-8
10. exp Medicine, Ayurvedic/
11. Ayurved*.tw,ot.
12. *Medicine, traditional/
13. exp Complementary medicine/tu [Therapeutic use]
14. ((Plant* or herb* or medicin* or drug* or therap* or intervention* or extract* or formulation* or preparation* or supplement*) adj6 (Ayurved* or Hindu or Indian)).tw,ot.
15. exp Plants, medicinal/tu [Therapeutic use]
16. exp Plant extracts/tu [Therapeutic use]
17. exp Plants/tu [Therapeutic use]
18. ((Plant* or herb*) adj6 (medicin* or drug* or therap* or intervention* or extract* or formulation* or preparation* or supplement*)).tw,ot.
19. exp Ethnobotany/
20. exp Ethnopharmacology/
21. (Ethnobotan* or ethno botan* or ethnopharmacolog* or ethno pharmacolog*).tw,ot.
22. *Phytotherapy/
23. (Phytotherap* or phyto therap*).tw,ot.
24. exp Acanthaceae/
25. exp Aegle/
26. exp Aloe/
27. exp Artocarpus/
28. exp Azadirachta/
29. exp Butea/
30. exp Cassia/
31. exp Catharanthus/
32. exp Cinnamomum/
33. exp Cinnamomum aromaticum/
34. exp Cinnamomum zeylanicum/
35. exp Clerodendrum/
36. exp Cucurbitaceae/
37. exp Curcuma/
38. exp Cyamopsis/
39. exp Ficus/
40. exp Garlic/
41. exp Ginger/
42. exp Gymnema/
43. exp Gymnema sylvestre/

44. exp Ipomoea batatas/
45. exp Momordica charantia/
46. exp Murraya/
47. exp Nigella sativa/
48. exp Ocimum sanctum/
49. exp Onions/
50. exp Phyllanthus/
51. exp Phyllanthus emblica/
52. exp Piper/
53. exp Plantago/
54. exp Psyllium/
55. exp Pterocarpus/
56. exp Punicaceae/
57. exp Salacia/
58. exp Solanum/
59. exp Syzygium/
60. exp Tamarindus/
61. exp Tinospora/
62. exp Trigonella/
63. exp Lead/
64. exp Tin/
65. (Aegle marmelos or Crateva marmelos or Allium cepa or Allium sativum or Aloe vera or Artocarpus heterophyllus or Azadirachta indica or Melia azadirachta or Butea monosperma or Cassia auriculata or Catharanthus roseus or Vinca rosea or Cinnamomum aromaticum or Cinnamomum cassia or Cinnamomum tamala or Cinnamomum verum or Cinnamomum zeylanicum or Clerodendrum phlomides or Coccinia grandis or Coccinia cordifolia or Coccinia indica or Curcuma longa or Cyamopsis tetragonoloba or Emblica officinalis or Phyllanthus emblica or Enicostemma axillare or Enicostemma littorale or Ficus carica or Gymnema lactiferum or Gymnema sylvestre or Hygrophila schulli or Asteracantha longifolia or Ipomoea batatas or Momordica charantia or Murraya koenigii or Nigella sativa or Ocimum tenuiflorum or Ocimum sanctum or Phyllanthus amarus or Phyllanthus nirurii or Piper longum or Plantago arenaria or Plantago psyllium or Pterocarpus marsupium or Pterocarpus santalinus or Punica granatum or Salacia chinensis or Salacia reticulate or Solanum torvum or Syzygium cumini or Eugenia jambolana or Syzygium jambolanum or Syzygium jambos or Eugenia jambos or Tamarindus indica or Tinospora cordifolia or Trigonella foenum-greacum or Zingiber officinale).tw,ot.
66. (Lead or Tin or Black bitumen or Black asphalt*).tw,ot.
67. (Arogyavardhini* or Arogyvardhini* or Ayaskriti or Chandrakant* or Chandraprabh* or Devdarvarisht* or Devdarvarist* or Dhanvantar* or Gokshuradi* or Goksuradi* or Jambudyarisht* or Jambudyarist* or Katakakhadiradi* or Kathakakhadiradi* or Kshirabal* or Ksirabal* or Lodhrasav* or Mamajjak* or Nag* or Naag* or Nimbadi* or Nimadi* or Nishamalak* or Nisamalak* or Nishamlak* or Nisamlak* or Nishakathakadi* or Nisakathakadi* or Phalatrikadi* or Falatrikadi* or Rajanyamalakadi* or Rajanyamlakadi* or Saptamrit* or Shaptamrit* or Saptargangyadi* or Shilajit* or Silajit* or Shilajat* or Silajat* or Shilajeet* or Silajeet* or Siva* or Shiva* or Somnath* or Shomnath* or Triphal* or Trifal* or Trivang* or Tribang* or Vang* or Bang* or Vasant Kusumakar* or Vashant Kushumakar* or Vasanta Kusumakar* or Vashanta Kushumakar* or Vasantkusumakar* or Vashantkushumakar* or Vasantakusumakar* or Vashantakushumakar* or Basant Kusumakar* or Bashant Kushumakar* or Basanta Kusumakar* or Bashanta Kushumakar* or Basantkusumakar* or Bashantkushumakar* or Basantakusumakar* or Bashantakushumakar* or Vijayasaradi* or Vijayasharadi* or Vijaysaradi* or Vijaysharadi* or Bijayasaradi* or Bijayasharadi* or Bijaysaradi* or Bijaysharadi* or Vyoshadi* or Vyosadi* or Byoshadi* or Byosadi*).tw,ot.
68. (Ayush 82 or Ayush-82 or Cogent DB or Diabecon or D 400 or D-400 or GS4 or Gurmar or Hyponidd or Inolter or M 93 or M-93 or MA 471 or MA-471 or Nosulin or Pancreas Tonic).tw,ot.

69. or/10-68
70. Randomized controlled trial.pt.
71. Controlled clinical trial.pt.
72. Clinical trial.pt.
73. exp Clinical trials as Topic/
74. Placebos/
75. Random allocation/
76. Double-blind method/
77. Single-blind method/
78. Cross-over studies/
79. ((Random$ or control$ or clinical$) adj3 (trial$ or stud$)).tw.
80. (Random$ adj3 allocat$).tw.
81. Placebo$.tw.
82. ((Singl$ or doubl$ or trebl$ or tripl$) adj (blind$ or mask$)).tw.
83. (Crossover$ or (cross adj over$)).tw.
84. or/70-83
85. 9 and 69 and 84

Embase (Ovid) (1974-16 January 2021): 13989 records

1. exp Diabetes mellitus, type 2/
2. (Type* adj3 ("2" or "II" or two*) adj3 (diabete* or diabetic*)).tw.
3. ((Late or maturit* or adult* or slow*) adj3 onset* adj3 (diabete* or diabetic*)).tw.
4. ((Ketosis-resistant* or stable*) adj3 (diabete* or diabetic*)).tw.
5. ((Non-insulin* or non insulin* or noninsulin*) adj3 depend* adj3 (diabete* or diabetic*)).tw.
6. (NIDDM or T2DM or T2D).tw.
7. Prameha.mp.
8. Madhumeha.mp.
9. or/1-8
10. exp Medicine, Ayurvedic/
11. Ayurved*.tw,ot.
12. *Medicine, traditional/
13. exp Complementary medicine/tu [Therapeutic use]
14. ((Plant* or herb* or medicin* or drug* or therap* or intervention* or extract* or formulation* or preparation* or supplement*) adj6 (Ayurved* or Hindu or Indian)).tw,ot.
15. exp Plants, medicinal/tu [Therapeutic use]
16. exp Plant extracts/tu [Therapeutic use]
17. exp Plants/tu [Therapeutic use]
18. ((Plant* or herb*) adj6 (medicin* or drug* or therap* or intervention* or extract* or formulation* or preparation* or supplement*)).tw,ot.
19. exp Ethnobotany/
20. exp Ethnopharmacology/
21. (Ethnobotan* or ethno botan* or ethnopharmacolog* or ethno pharmacolog*).tw,ot.
22. *Phytotherapy/
23. (Phytotherap* or phyto therap*).tw,ot.
24. exp Acanthaceae/
25. exp Aegle/
26. Aloe.mp. [mp=title, abstract, original title, name of substance word, subject heading word, floating sub-heading word, keyword heading word, organism supplementary concept word, protocol supplementary concept word, rare disease supplementary concept word, unique identifier, synonyms]
27. Artocarpus.mp. [mp=title, abstract, original title, name of substance word, subject heading word, floating sub-heading word, keyword heading word, organism supplementary concept word, protocol supplementary concept word, rare disease supplementary concept word, unique identifier, synonyms]

28. Azadirachta.mp. or exp Azadirachta indica/
29. exp Butea monosperma/ or exp Butea/ or Butea.mp.
30. Cassia.mp. or cassia extract/ or Cinnamomum cassia/ or exp Cassia/ or Cinnamomum cassia extract/
31. exp Catharanthus/
32. Cinnamomum.mp. or Cinnamomum camphora/ or exp Cinnamomum/
33. exp Cinnamomum/
34. exp Cinnamomum aromaticum/
35. exp Cinnamomum zeylanicum/
36. exp Clerodendrum/
37. exp Cucurbitaceae/
38. exp Curcuma/
39. exp Cyamopsis/
40. exp Ficus/
41. exp Garlic/
42. exp Ginger/
43. exp Gymnema/
44. exp Gymnema sylvestre/
45. exp Ipomoea batatas/
46. exp Momordica charantia/
47. exp Murraya/
48. exp Nigella sativa/
49. exp Ocimum sanctum/
50. exp Phyllanthus/
51. exp Phyllanthus emblica/
52. exp Piper/
53. exp Plantago/
54. exp Psyllium/
55. exp Pterocarpus/
56. exp Punicaceae/
57. exp Salacia/
58. exp Solanum/
59. Syzygium cumini/ or Syzygium.mp. or exp Syzygium/ or Syzygium cumini extract/
60. Tamarindus.mp. or exp Tamarind/
61. Tinospora cordifolia/ or Tinospora.mp. or exp Tinospora/ or Tinospora cordifolia extract/
62. Fenugreek/ or Trigonella.mp. or Trigonella foenum graecum extract/ or nonhuman/ or exp Trigonella/ or plant extract/ or plant seed/
63. exp Lead/ or Lead.mp.
64. Tin derivative/ orTin.mp. or exp Tin/
65. Onion extract/ or Onion.mp. or exp Onion/
66. Piper.mp. or exp "Piper (plant)"/
67. (Lead or Tin or Black bitumen or Black asphalt*).tw,ab,kw,ot.
68. (Aegle marmelos or Crateva marmelos or Allium cepa or Allium sativum or Aloe vera or Artocarpus heterophyllus or Azadirachta indica or Melia azadirachta or Butea monosperma or Cassia auriculata or Catharanthus roseus or Vinca rosea or Cinnamomum aromaticum or Cinnamomum cassia or Cinnamomum tamala or Cinnamomum verum or Cinnamomum zeylanicum or Clerodendrum phlomides or Coccinia grandis or Coccinia cordifolia or Coccinia indica or Curcuma longa or Cyamopsis tetragonoloba or Emblica officinalis or Phyllanthus emblica or Enicostemma axillare or Enicostemma littorale or Ficus carica or Gymnema lactiferum or Gymnema sylvestre or Hygrophila schulli or Asteracantha longifolia or Ipomoea batatas or Momordica charantia or Murraya koenigii or Nigella sativa or Ocimum tenuiflorum or Ocimum sanctum or Phyllanthus amarus or Phyllanthus nirurii or Piper longum or Plantago arenaria or Plantago psyllium or Pterocarpus marsupium or Pterocarpus santalinus or Punica granatum or Salacia chinensis or Salacia reticulate or Solanum torvum or Syzygium cumini or Eugenia jambolana or Syzygium jambolanum or Syzygium jambos or Eugenia jambos or

Tamarindus indica or Tinospora cordifolia or Trigonella foenum-greacum or Zingiber officinale).tw,ab,kw,ti.
69. (Arogyavardhini* or Arogyvardhini* or Ayaskriti or Chandrakant* or Chandraprabh* or Devdarvarisht* or Devdarvarist* or Dhanvantar* or Gokshuradi* or Goksuradi* or Jambudyarisht*or Jambudyarist* or Katakakhadiradi* or Kathakakhadiradi* or Kshirabal* or Ksirabal* or Lodhrasav* or Mamajjak* or Nag* or Naag* or Nimbadi* or Nimadi* or Nishamalak* or Nisamalak* or Nishamlak* or Nisamlak* or Nishakathakadi* or Nisakathakadi* or Phalatrikadi* or Falatrikadi* or Rajanyamalakadi* or Rajanyamlakadi* or Saptamrit* or Shaptamrit* or Saptargangyadi* or Shilajit* or Silajit* or Shilajat* or Silajat* or Shilajeet* or Silajeet* or Siva* or Shiva* or Somnath* or Shomnath* or Triphal* or Trifal* or Trivang* or Tribang* or Vang* or Bang* or Vasant Kusumakar* or Vashant Kushumakar* or Vasanta Kusumakar* or Vashanta Kushumakar* or Vasantkusumakar* or Vashantkushumakar* or Vasantakusumakar* or Vashantakushumakar* or Basant Kusumakar* or Bashant Kushumakar* or Basanta Kusumakar* or Bashanta Kushumakar* or Basantkusumakar* or Bashantkushumakar* or Basantakusumakar* or Bashantakushumakar* or Vijayasaradi* or Vijayasharadi* or Vijaysaradi* or Vijaysharadi* or Bijayasaradi* or Bijayasharadi* or Bijaysaradi* or Bijaysharadi* or Vyoshadi* or Vyosadi* or Byoshadi* or Byosadi*).tw,ot,ab,kw,ti.
70. or/10-69
71. Clinical trial/
72. Randomized controlled trial/
73. Randomization/
74. Single blind procedure/
75. Double blind procedure/
76. Crossover procedure/
77. Placebo/
78. Randomi?ed controlled trial$.tw.
79. RCT.tw.
80. Random allocation.tw.
81. Randomly allocated.tw.
82. Allocated randomly.tw.
83. (Allocated adj2 random).tw.
84. Single blind$.tw.
85. Double blind$.tw.
86. ((Treble or triple) adj blind$).tw.
87. Placebo$.tw.
88. Prospective study/
89. or/71-88
90. Case study/
91. Case report.tw.
92. Abstract report/ or letter/
93. or/90-92
94. 89 not 93
95. 9 and 70 and 94

CINAHL (EBSCOhost) (1937-16 January 2021): 193 records

(MH "medicine, Ayurvedic" OR Ayurved*) AND (MH "diabetes mellitus, type 2" OR diabet* OR NIDDM OR T2DM OR T2D OR Madhumeh* OR Prameh*)

PsycINFO (Ovid) (1806-16 January 2021): 595 records

1. exp Diabetes mellitus/
2. (Type* adj3 ("2" or "II" or two*) adj3 (diabete* or diabetic*)).tw.
3. ((Late or maturit* or adult* or slow*) adj3 onset* adj3 (diabete* or diabetic*)).tw.
4. ((Ketosis-resistant* or stable*) adj3 (diabete* or diabetic*)).tw.

5. ((Non-insulin* or non insulin* or noninsulin*) adj3 depend* adj3 (diabete* or diabetic*)).tw.
6. (NIDDM or T2DM or T2D).tw.
7. 1 or 2 or 3 or 4 or 5 or 6
8. Ayurvedic medicine.mp.
9. Ayurved*.tw,ot.
10. Traditional medicine.mp.
11. exp Complementary medicine/
12. ((Plant* or herb* or medicin* or drug* or therap* or intervention* or extract* or formulation* or preparation* or supplement*) adj6 (Ayurved* or Hindu or Indian)).tw,ot.
13. exp "Medicinal herbs and plants"/
14. Plant extracts.mp.
15. Plants.mp.
16. ((Plant* or herb*) adj6 (medicin* or drug* or therap* or intervention* or extract* or formulation* or preparation* or supplement*)).tw,ot.
17. Ethnobotany.mp.
18. Ethnopharmacology.mp.
19. (Ethnobotan* or ethno botan* or ethnopharmacolog* or ethno pharmacolog*).tw,ot.
20. Phytotherapy.mp.
21. (Phytotherap* or phyto therap*).tw,ot.
22. Acanthaceae.mp.
23. Aegle.mp.
24. Aloe.mp.
25. Artocarpus.mp.
26. Azadirachta.mp.
27. Butea.mp.
28. Cassia.mp.
29. Cinnamomum.mp.
30. Cinnamomum zeylanicum.mp.
31. Clerodendrum.mp.
32. Cucurbitaceae.mp.
33. Curcuma.mp.
34. Ficus.mp.
35. Garlic.mp.
36. Ginger.mp.
37. Gymnema.mp.
38. Ipomoea batatas.mp.
39. Momordica charantia.mp.
40. Murraya.mp.
41. Nigella sativa.mp.
42. Ocimum sanctum.mp.
43. Onions.mp.
44. Piper.mp.
45. Plantago.mp.
46. Psyllium.mp.
47. Pterocarpus.mp.
48. Salacia.mp.
49. Solanum.mp.
50. Syzygium.mp.
51. Tamarindus.mp.
52. Tinospora.mp.
53. Trigonella.mp.
54. Lead.mp.
55. Tin.mp.
56. (Aegle marmelos or Crateva marmelos or Allium cepa or Allium sativum or Aloe vera or Artocarpus heterophyllus or Azadirachta indica or Melia azadirachta or Butea monosperma or

Cassia auriculata or Catharanthus roseus or Vinca rosea or Cinnamomum aromaticum or Cinnamomum cassia or Cinnamomum tamala or Cinnamomum verum or Cinnamomum zeylanicum or Clerodendrum phlomides or Coccinia grandis or Coccinia cordifolia or Coccinia indica or Curcuma longa or Cyamopsis tetragonoloba or Emblica officinalis or Phyllanthus emblica or Enicostemma axillare or Enicostemma littorale or Ficus carica or Gymnema lactiferum or Gymnema sylvestre or Hygrophila schulli or Asteracantha longifolia or Ipomoea batatas or Momordica charantia or Murraya koenigii or Nigella sativa or Ocimum tenuiflorum or Ocimum sanctum or Phyllanthus amarus or Phyllanthus nirurii or Piper longum or Plantago arenaria or Plantago psyllium or Pterocarpus marsupium or Pterocarpus santalinus or Punica granatum or Salacia chinensis or Salacia reticulate or Solanum torvum or Syzygium cumini or Eugenia jambolana or Syzygium jambolanum or Syzygium jambos or Eugenia jambos or Tamarindus indica or Tinospora cordifolia or Trigonella foenum-greacum or Zingiber officinale).tw,ot.
57. (Lead or Tin or Black bitumen or Black asphalt*).tw,ot.
58. (Arogyavardhini* or Arogyvardhini* or Ayaskriti or Chandrakant* or Chandraprabh* or Devdarvarisht* or Devdarvarist* or Dhanvantar* or Gokshuradi* or Goksuradi* or Jambudyarisht*or Jambudyarist* or Katakakhadiradi* or Kathakakhadiradi* or Kshirabal* or Ksirabal* or Lodhrasav* or Mamajjak* or Nag* or Naag* or Nimbadi* or Nimadi* or Nishamalak* or Nisamalak* or Nishamlak* or Nisamlak* or Nishakathakadi* or Nisakathakadi* or Phalatrikadi* or Falatrikadi* or Rajanyamalakadi* or Rajanyamlakadi* or Saptamrit* or Shaptamrit* or Saptargangyadi* or Shilajit* or Silajit* or Shilajat* or Silajat* or Shilajeet* or Silajeet* or Siva* or Shiva* or Somnath* or Shomnath* or Triphal* or Trifal* or Trivang* or Tribang* or Vang* or Bang* or Vasant Kusumakar* or Vashant Kushumakar* or Vasanta Kusumakar* or Vashanta Kushumakar* or Vasantkusumakar* or Vashantkushumakar* or Vasantakusumakar* or Vashantakushumakar* or Basant Kusumakar* or Bashant Kushumakar* or Basanta Kusumakar* or Bashanta Kushumakar* or Basantkusumakar* or Bashantkushumakar* or Basantakusumakar* or Bashantakushumakar* or Vijayasaradi* or Vijayasharadi* or Vijaysaradi* or Vijaysharadi* or Bijayasaradi* or Bijayasharadi* or Bijaysaradi* or Bijaysharadi* or Vyoshadi* or Vyosadi* or Byoshadi* or Byosadi*).tw,ot.
59. (Ayush 82 or Ayush-82 or Cogent DB or Diabecon or D 400 or D-400 or GS4 or Gurmar or Hyponidd or Inolter or M 93 or M-93 or MA 471 or MA-471 or Nosulin or Pancreas Tonic).tw,ot.
60. 8 or 9 or 10 or 11 or 12 or 13 or 14 or 15 or 16 or 17 or 18 or 19 or 20 or 21 or 22 or 23 or 24 or 25 or 26 or 27 or 28 or 29 or 30 or 31 or 32 or 33 or 34 or 35 or 36 or 37 or 38 or 39 or 40 or 41 or 42 or 43 or 44 or 45 or 46 or 47 or 48 or 49 or 50 or 51 or 52 or 53 or 54 or 55 or 56 or 57 or 58 or 59
61. 7 and 60

Search strategy included only disease and treatment domains. Study design domain was removed.

Web of Science (1900-16 January 2021): 44 records
#1 TI= (“Type 2 diabetes mellitus” OR (type* adj3 ("2" or "II" or two*) adj3 (diabete* or diabetic*)) OR ((Late or maturit* or adult* or slow*) adj3 onset* adj3 (diabete* or diabetic*)) OR ((ketosis-resistant* or stable*) adj3 (diabete* or diabetic*)) OR ((non-insulin* or non insulin* or noninsulin*) adj3 depend* adj3 (diabete* or diabetic*)) OR (NIDDM or T2DM or T2D) OR (Prameh* OR Madhumeh*))

#2 TI= (“Ayurvedic medicine” OR Ayurved* OR “traditional medicine” OR “complementary medicine” OR ((plant* or herb* or medicin* or drug* or therap* or intervention* or extract* or formulation* or preparation* or supplement*) adj6 (Ayurved* or Hindu or Indian)) OR “medicinal plants” OR “plant extracts” OR plants OR ((plant* or herb*) adj6 (medicin* or drug* or therap* or intervention* or extract* or formulation* or preparation* or supplement*)) OR ethnobotany OR ethnopharmacology OR (ethnobotan* or ethno botan* or ethnopharmacolog*

or ethno pharmacolog*) OR phytotherapy OR (phytotherap* or phyto therap*) OR Acanthaceae OR Aegle OR Aloe OR Artocarpus OR azadirachta OR Butea OR Cassia OR Catharanthus OR Cinnamomum OR “Cinnamomum aromaticum” OR “Cinnamomum zeylanicum” OR Clerodendrum OR Cucurbitaceae OR Curcuma OR Cyamopsis OR Ficus OR Garlic OR Ginger OR Gymnema OR “Gymnema sylvestre” OR “Ipomoea batatas” OR Momordica charantia OR Murraya OR “Nigella sativa” OR “Ocimum sanctum” OR Onions OR Phyllanthus OR Phyllanthus emblica OR Piper OR Plantago OR Psyllium OR Pterocarpus OR Punicaceae OR Salacia OR Solanum OR Syzygium OR Tamarindus OR Tinospora OR Trigonella OR Lead OR Tin OR (Aegle marmelos or Crateva marmelos or Allium cepa or Allium sativum or Aloe vera or Artocarpus heterophyllus or “Azadirachta indica” or “Melia azadirachta” or “Butea monosperma” or “Cassia auriculate” or “Catharanthus roseus” or “Vinca rosea” or “Cinnamomum aromaticum” or “Cinnamomum cassia” or “Cinnamomum tamala” or “Cinnamomum verum” or “Cinnamomum zeylanicum” or “Clerodendrum phlomides” or “Coccinia grandis” or Coccinia cordifolia or Coccinia indica or “Curcuma longa” or “Cyamopsis tetragonoloba” or “Emblica officinalis” or “Phyllanthus emblica” or “Enicostemma axillare” or “Enicostemma littorale” or “Ficus carica” or “Gymnema lactiferum” or “Gymnema sylvestre” or “Hygrophila schulli” or “Asteracantha longifolia” or “Ipomoea batatas” or “Momordica charantia” or “Murraya koenigii” or “Nigella sativa” or “Ocimum tenuiflorum” or “Ocimum sanctum” or “Phyllanthus amarus” or “Phyllanthus nirurii” or “Piper longum” or “Plantago Arenaria” or “Plantago psyllium” or “Pterocarpus marsupium” or “Pterocarpus santalinus” or “Punica granatum” or “Salacia chinensis” or “Salacia reticulate” or “Solanum torvum” or “Syzygium cumini” or “Eugenia jambolana” or “Syzygium jambolanum” or “Syzygium jambos” or “Eugenia jambos” or “Tamarindus indica” or “Tinospora cordifolia” or “Trigo”) OR (Arogyavardhini* or Arogyvardhini* or Ayaskriti or Chandrakant* or Chandraprabh* or Devdarvarisht* or Devdarvarist* or Dhanvantar* or Gokshuradi* or Goksuradi* or Jambudyarisht*or Jambudyarist* or Katakakhadiradi* or Kathakakhadiradi* or Kshirabal* or Ksirabal* or Lodhrasav* or Mamajjak* or Nag* or Naag* or Nimbadi* or Nimadi* or Nishamalak* or Nisamalak* or Nishamlak* or Nisamlak* or Nishakathakadi* or Nisakathakadi* or Phalatrikadi* or Falatrikadi* or Rajanyamalakadi* or Rajanyamlakadi* or Saptamrit* or Shaptamrit* or Saptargangyadi* or Shilajit* or Silajit* or Shilajat* or Silajat* or Shilajeet* or Silajeet* or Siva* or Shiva* or Somnath* or Shomnath* or Triphal* or Trifal* or Trivang* or Tribang* or Vang* or Bang* or Vasant Kusumakar* or Vashant Kushumakar* or Vasanta Kusumakar* or Vashanta Kushumakar* or Vasantkusumakar* or Vashantkushumakar* or Vasantakusumakar* or Vashantakushumakar* or Basant Kusumakar* or Bashant Kushumakar* or Basanta Kusumakar* or Bashanta Kushumakar* or Basantkusumakar* or Bashantkushumakar* or Basantakusumakar* or Bashantakushumakar* or Vijayasaradi* or Vijayasharadi* or Vijaysaradi* or Vijaysharadi* or Bijayasaradi* or Bijayasharadi* or Bijaysaradi* or Bijaysharadi* or Vyoshadi* or Vyosadi* or Byoshadi* or Byosadi*) OR (Ayush 82 or Ayush-82 or Cogent DB or Diabecon or “D 400” or “D-400” or GS4 or Gurmar or Hyponidd or Inolter or “M 93” or “M-93” or “MA 471” or “MA-471” or Nosulin or “Pancreas Tonic”))

#3 ALL= (“Randomized controlled trial” OR “controlled clinical trial” OR “clinical trial” OR “clinical trials” OR placebos OR “random allocation” OR “double-blind method” OR “single-blind method” OR “cross-over studies” OR ((random$ or control$ or clinical$) adj3 (trial$ or stud$)) OR (random$ adj3 allocat$) OR placebo$ OR ((singl$ or doubl$ or trebl$ or tripl$) adj (blind$ or mask$)) OR (crossover$ or (cross adj over$)))

#1 AND #2 AND #3

Cochrane Central Register of Controlled Trials (CENTRAL) (1996-16 January 2021): 9442 records

#1 MeSH descriptor: [Diabetes mellitus, type 2] explode all trees and with qualifier(s): [diagnosis - DI]

#2 Diabet* in Trials
#3 (NIDDM or T2DM or T2D) in Trials
#4 Ketosis resistant diabete or ketosis resistant diabetic or stable diabete or stable diabetic in Trials
#5 ((Non-insulin* or non insulin* or noninsulin*) adj3 depend* adj3 (diabete* or diabetic*))
#6 ((Late or maturit* or adult* or slow*) adj3 onset* adj3 (diabete* or diabetic*))
#7 Prameh* in Trials
#8 Madhumeh* in Trials
#9 #1 OR #2 OR #3 OR #4 OR #5 OR #6 OR #7 OR #8 in Trials
#10 MeSH descriptor: [Diabetes mellitus, type 1] explode all trees and with qualifier(s): [diagnosis - DI]
#11 Diabet* insipidus in Trials
#12 #10 OR #11 in Trials
#13 #9 NOT #12
#14 MeSH descriptor: [Medicine, traditional] explode all trees
#15 MeSH descriptor: [Medicine, Ayurvedic] explode all trees
#16 MeSH descriptor: [Complementary therapies] explode all trees
#17 MeSH descriptor: [Plants, medicinal] explode all trees
#18 MeSH descriptor: [Phytotherapy] explode all trees
#19 MeSH descriptor: [Ethnobotany] explode all trees
#20 MeSH descriptor: [Ethnopharmacology] explode all trees
#21 (Ethnobotan*):ti,ab,kw (Word variations have been searched)
#22 (Ethnopharmacolog*):ti,ab,kw (Word variations have been searched) in Trials
#23 MeSH descriptor: [Aloe] explode all trees
#24 (Ezadirachta):ti,ab,kw (Word variations have been searched) in Trials
#25 ("Aegle"):ti,ab,kw (Word variations have been searched) in Trials
#26 ("Artocarpus"):ti,ab,kw (Word variations have been searched)
#27 (Butea*):ti,ab,kw (Word variations have been searched)
#28 MeSH descriptor: [Cassia] explode all trees
#29 ("Cassia"):ti,ab,kw (Word variations have been searched) in Trials
#30 ("Cinnamomum"):ti,ab,kw (Word variations have been searched)
#31 (Cinnamomum zeylanicum):ti,ab,kw (Word variations have been searched)
#32 ("Cucurbita"):ti,ab,kw (Word variations have been searched)
#33 (Curcuma):ti,ab,kw (Word variations have been searched)
#34 MeSH descriptor: [Trigonella] explode all trees
#35 MeSH descriptor: [Ficus] explode all trees
#36 MeSH descriptor: [Garlic] explode all trees
#37 MeSH descriptor: [Ginger] explode all trees
#38 MeSH descriptor: [Zingiberales] explode all trees
#39 MeSH descriptor: [Gymnema] explode all trees
#40 ("Gymnema sylvestre"):ti,ab,kw (Word variations have been searched)
#41 ("Ipomoea batatas"):ti,ab,kw (Word variations have been searched)
#42 ("Momordica charantia"):ti,ab,kw (Word variations have been searched)
#43 (Murraya):ti,ab,kw (Word variations have been searched)
#44 ("Nigella sativa"):ti,ab,kw (Word variations have been searched)
#45 ("Ocimum sanctum"):ti,ab,kw (Word variations have been searched)
#46 MeSH descriptor: [Onions] explode all trees
#47 MeSH descriptor: [Allium] explode all trees
#48 ("Piper"):ti,ab,kw (Word variations have been searched)
#49 MeSH descriptor: [Tinospora] explode all trees
#50 ("Tinospora"):ti,ab,kw (Word variations have been searched)
#51 ("Tamarindus"):ti,ab,kw (Word variations have been searched)
#52 ("Salacia") (Word variations have been searched)
#53 (Lead):ti,ab,kw (Word variations have been searched)
#54 (Tin):ti,ab,kw (Word variations have been searched)

#55 (Arogyavardhini* or Arogyvardhini* or Ayaskriti or Chandrakant* or Chandraprabh* or Devdarvarisht* or Devdarvarist* or Dhanvantar* or Gokshuradi* or Goksuradi* or Jambudyarisht*or Jambudyarist* or Katakakhadiradi* or Kathakakhadiradi* or Kshirabal* or Ksirabal* or Lodhrasav* or Mamajjak* or Nag* or Naag* or Nimbadi* or Nimadi* or Nishamalak* or Nisamalak* or Nishamlak* or Nisamlak* or Nishakathakadi* or Nisakathakadi* or Phalatrikadi* or Falatrikadi* or Rajanyamalakadi* or Rajanyamlakadi* or Saptamrit* or Shaptamrit* or Saptargangyadi* or Shilajit* or Silajit* or Shilajat* or Silajat* or Shilajeet* or Silajeet* or Siva* or Shiva* or Somnath* or Shomnath* or Triphal* or Trifal* or Trivang* or Tribang* or Vang* or Bang* or Vasant Kusumakar* or Vashant Kushumakar* or Vasanta Kusumakar* or Vashanta Kushumakar* or Vasantkusumakar* or Vashantkushumakar* or Vasantakusumakar* or Vashantakushumakar* or Basant Kusumakar* or Bashant Kushumakar* or Basanta Kusumakar* or Bashanta Kushumakar* or Basantkusumakar* or Bashantkushumakar* or Basantakusumakar* or Bashantakushumakar* or Vijayasaradi* or Vijayasharadi* or Vijaysaradi* or Vijaysharadi* or Bijayasaradi* or Bijayasharadi* or Bijaysaradi* or Bijaysharadi* or Vyoshadi* or Vyosadi* or Byoshadi* or Byosadi*) (Word variations have been searched)
#56 (Ayush 82 or Ayush-82 or Cogent DB or Diabecon or D 400 or D-400 or GS4 or Gurmar or Hyponidd or Inolter or M 93 or M-93 or MA 471 or MA-471 or Nosulin or Pancreas Tonic) (Word variations have been searched)
#57 #14 OR #15 OR #16 OR #17 OR #18 OR #19 OR #20 OR #21 OR #22 in Trials
#58 #23 OR #24 OR #25 OR #26 OR #27 OR #28 OR #29 OR #30 OR 29 #32
#59 #57 OR #58
#60 #33 OR #34 OR #35 OR #36 OR #37 OR #38 OR #39 OR #40 OR #41 OR #42
#61 #43 OR #44 OR #45 OR #46 OR #47 OR #48 OR #49 OR #50 OR #51 OR #52
#62 #60 OR #61
#63 #53 OR #54 OR #55 OR #56
#64 #59 OR #62 OR #63
#65 #13 AND #64

Allied and Complementary Medicine Database (AMED) (Ovid) (1985-16 January 2021): 474 records

1. exp Diabetes mellitus type 2/
2. (Type* adj3 ("2" or "II" or two*) adj3 (diabete* or diabetic*)).tw.
3. ((Late or maturit* or adult* or slow*) adj3 onset* adj3 (diabete* or diabetic*)).tw.
4. ((Ketosis-resistant* or stable*) adj3 (diabete* or diabetic*)).tw.
5. ((Non-insulin* or non insulin* or noninsulin*) adj3 depend* adj3 (diabete* or diabetic*)).tw.
6. (NIDDM or T2DM or T2D).tw.
7. Prameha.mp.
8. Madhumeh*.mp.
9. or/1-8
10. exp Ayurvedic medicine/
11. Ayurved*.tw,ti,ab.
12. exp Traditional medicine/
13. exp Complementary medicine/
14. ((Plant* or herb* or medicin* or drug* or therap* or intervention* or extract* or formulation* or preparation* or supplement*) adj6 (Ayurved* or Hindu or Indian)).tw,ti,ab.
15. exp Ethnopharmacology/
16. Ethnobotany.mp.
17. (Ethnobotan* or ethno botan* or ethnopharmacolog* or ethno pharmacolog*).tw,ti,ab.
18. exp Phytotherapy/
19. (Phytotherap* or phyto therap*).tw,ti,ab.
20. Acanthaceae.mp.
21. Aegle.mp.
22. exp Aloe/

23. exp Artocarpus/
24. exp Azadirachta/
25. Butea.mp.
26. Cassia.mp.
27. Catharanthus.mp.
28. exp Cinnamomum/
29. Cinnamomum zeylanicum.mp.
30. Clerodendrum.mp.
31. Cucurbitaceae.mp.
32. exp Curcuma/
33. exp Cyamopsis/
34. exp Ficus/
35. Garlic.mp.
36. exp Zingiber officinale/
37. exp Allium sativum/
38. Gymnema.mp.
39. Gymnema sylvestre.mp.
40. exp Ipomoea/
41. exp Ipomoea/ or Ipomoea batatas.mp.
42. exp Momordica charantia/
43. exp Momordica/
44. Curry.mp.
45. Murraya koenigii.mp.
46. exp Nigella sativa/
47. Ocimum sanctum.mp.
48. Onions.mp.
49. exp Allium cepa/
50. exp Phyllanthus/
51. Emblic.mp.
52. Amla.mp.
53. Myrobalan.mp.
54. exp Piper/
55. Piper longum.mp.
56. Plantago.mp.
57. Psyllium.mp.
58. Pterocarpus.mp.
59. exp Punicaceae/
60. Salacia.mp.
61. Syzgium.mp.
62. Tamarindus.mp.
63. Tinospora.mp.
64. Tinospora cordifolia.mp.
65. Guduchi.mp.
66. exp Trigonella/
67. Fenugreek.mp.
68. exp Lead/
69. exp Tin/
70. (Lead or Tin or Black bitumen or Black asphalt*).tw,ab,ti.
71. (Aegle marmelos or Crateva marmelos or Allium cepa or Allium sativum or Aloe vera or Artocarpus heterophyllus or Azadirachta indica or Melia azadirachta or Butea monosperma or Cassia auriculata or Catharanthus roseus or Vinca rosea or Cinnamomum aromaticum or Cinnamomum cassia or Cinnamomum tamala or Cinnamomum verum or Cinnamomum zeylanicum or Clerodendrum phlomides or Coccinia grandis or Coccinia cordifolia or Coccinia indica or Curcuma longa or Cyamopsis tetragonoloba or Emblica officinalis or Phyllanthus emblica or Enicostemma axillare or Enicostemma littorale or Ficus carica or Gymnema

lactiferum or Gymnema sylvestre or Hygrophila schulli or Asteracantha longifolia or Ipomoea batatas or Momordica charantia or Murraya koenigii or Nigella sativa or Ocimum tenuiflorum or Ocimum sanctum or Phyllanthus amarus or Phyllanthus nirurii or Piper longum or Plantago arenaria or Plantago psyllium or Pterocarpus marsupium or Pterocarpus santalinus or Punica granatum or Salacia chinensis or Salacia reticulate or Solanum torvum or Syzygium cumini or Eugenia jambolana or Syzygium jambolanum or Syzygium jambos or Eugenia jambos or Tamarindus indica or Tinospora cordifolia or Trigonella foenum-greacum or Zingiber officinale).tw,ti,ab.
72. (Arogyavardhini* or Arogyvardhini* or Ayaskriti or Chandrakant* or Chandraprabh* or Devdarvarisht* or Devdarvarist* or Dhanvantar* or Gokshuradi* or Goksuradi* or Jambudyarisht*or Jambudyarist* or Katakakhadiradi* or Kathakakhadiradi* or Kshirabal* or Ksirabal* or Lodhrasav* or Mamajjak* or Nag* or Naag* or Nimbadi* or Nimadi* or Nishamalak* or Nisamalak* or Nishamlak* or Nisamlak* or Nishakathakadi* or Nisakathakadi* or Phalatrikadi* or Falatrikadi* or Rajanyamalakadi* or Rajanyamlakadi* or Saptamrit* or Shaptamrit* or Saptargangyadi* or Shilajit* or Silajit* or Shilajat* or Silajat* or Shilajeet* or Silajeet* or Siva* or Shiva* or Somnath* or Shomnath* or Triphal* or Trifal* or Trivang* or Tribang* or Vang* or Bang* or Vasant Kusumakar* or Vashant Kushumakar* or Vasanta Kusumakar* or Vashanta Kushumakar* or Vasantkusumakar* or Vashantkushumakar* or Vasantakusumakar* or Vashantakushumakar* or Basant Kusumakar* or Bashant Kushumakar* or Basanta Kusumakar* or Bashanta Kushumakar* or Basantkusumakar* or Bashantkushumakar* or Basantakusumakar* or Bashantakushumakar* or Vijayasaradi* or Vijayasharadi* or Vijaysaradi* or Vijaysharadi* or Bijayasaradi* or Bijayasharadi* or Bijaysaradi* or Bijaysharadi* or Vyoshadi* or Vyosadi* or Byoshadi* or Byosadi*).tw,ti,ab.
73. (Ayush 82 or Ayush-82 or Cogent DB or Diabecon or D 400 or D-400 or GS4 or Gurmar or Hyponidd or Inolter or M 93 or M-93 or MA 471 or MA-471 or Nosulin or Pancreas Tonic).tw,ti,ab.
74. or/10-73
75. 9 and 74

Search strategy included only disease and treatment domains. Study design domain was removed.

International Pharmaceutical Abstracts (Ovid) (1970-16 January 2021): 286 records
1. Diabetes mellitus.mp. [mp=title, subject heading word, registry word, abstract, trade name/generic name]
2. (Type* adj3 ("2" or "II" or two*) adj3 (diabete* or diabetic*)).tw.
3. ((Late or maturit* or adult* or slow*) adj3 onset* adj3 (diabete* or diabetic*)).tw.
4. ((Ketosis-resistant* or stable*) adj3 (diabete* or diabetic*)).tw.
5. ((Non-insulin* or non insulin* or noninsulin*) adj3 depend* adj3 (diabete* or diabetic*)).tw.
6. (NIDDM or T2DM or T2D).tw.
7. Prameh*.mp. [mp=title, subject heading word, registry word, abstract, trade name/generic name]
8. Madhumeh*.mp. [mp=title, subject heading word, registry word, abstract, trade name/generic name]
9. 1 or 2 or 3 or 4 or 5 or 6 or 7 or 8
10. Ayurvedic medicine.mp.
11. Ayurved*.tw.
12. Traditional medicine.mp.
13. Complementary medicine.mp. [mp=title, subject heading word, registry word, abstract, trade name/generic name]
14. ((Plant* or herb* or medicin* or drug* or therap* or intervention* or extract* or formulation* or preparation* or supplement*) adj6 (Ayurved* or Hindu or Indian)).tw.
15. Medicinal plant.mp. [mp=title, subject heading word, registry word, abstract, trade name/generic name]

16. Plant extracts.mp.
17. Plants.mp.
18. ((Plant* or herb*) adj6 (medicin* or drug* or therap* or intervention* or extract* or formulation* or preparation* or supplement*)).tw.
19. Ethnobotany.mp.
20. Ethnopharmacology.mp.
21. (Ethnobotan* or ethno botan* or ethnopharmacolog* or ethno pharmacolog*).tw.
22. Phytotherapy.mp.
23. (Phytotherap* or phyto therap*).tw.
24. Acanthaceae.mp.
25. Aegle.mp.
26. Aloe.mp.
27. Artocarpus.mp.
28. Azadirachta.mp.
29. Butea.mp.
30. Cassia.mp.
31. Cinnamomum.mp.
32. Cinnamomum aromaticum.mp. [mp=title, subject heading word, registry word, abstract, trade name/generic name]
33. Cinnamomum zeylanicum.mp.
34. Clerodendrum.mp.
35. Cucurbitaceae.mp.
36. Curcuma.mp.
37. Cyamopsis.mp. [mp=title, subject heading word, registry word, abstract, trade name/generic name]
38. Ficus.mp.
39. Garlic.mp.
40. Ginger.mp.
41. Gymnema.mp.
42. Gymnema sylvestre.mp. [mp=title, subject heading word, registry word, abstract, trade name/generic name]
43. Ipomoea batatas.mp.
44. Momordica charantia.mp.
45. Murraya.mp.
46. Nigella sativa.mp.
47. Ocimum sanctum.mp.
48. Onions.mp.
49. Phyllanthus.mp. [mp=title, subject heading word, registry word, abstract, trade name/generic name]
50. Phyllanthus emblica.mp. [mp=title, subject heading word, registry word, abstract, trade name/generic name]
51. Piper.mp.
52. Plantago.mp.
53. Psyllium.mp.
54. Pterocarpus.mp.
55. Punicaceae.mp. [mp=title, subject heading word, registry word, abstract, trade name/generic name]
56. Salacia.mp.
57. Solanum.mp.
58. Syzygium.mp.
59. Tamarindus.mp.
60. Tinospora.mp.
61. Trigonella.mp.
62. Lead.mp.
63. Tin.mp.

64. (Aegle marmelos or Crateva marmelos or Allium cepa or Allium sativum or Aloe vera or Artocarpus heterophyllus or Azadirachta indica or Melia azadirachta or Butea monosperma or Cassia auriculata or Catharanthus roseus or Vinca rosea or Cinnamomum aromaticum or Cinnamomum cassia or Cinnamomum tamala or Cinnamomum verum or Cinnamomum zeylanicum or Clerodendrum phlomides or Coccinia grandis or Coccinia cordifolia or Coccinia indica or Curcuma longa or Cyamopsis tetragonoloba or Emblica officinalis or Phyllanthus emblica or Enicostemma axillare or Enicostemma littorale or Ficus carica or Gymnema lactiferum or Gymnema sylvestre or Hygrophila schulli or Asteracantha longifolia or Ipomoea batatas or Momordica charantia or Murraya koenigii or Nigella sativa or Ocimum tenuiflorum or Ocimum sanctum or Phyllanthus amarus or Phyllanthus nirurii or Piper longum or Plantago arenaria or Plantago psyllium or Pterocarpus marsupium or Pterocarpus santalinus or Punica granatum or Salacia chinensis or Salacia reticulate or Solanum torvum or Syzygium cumini or Eugenia jambolana or Syzygium jambolanum or Syzygium jambos or Eugenia jambos or Tamarindus indica or Tinospora cordifolia or Trigonella foenum-greacum or Zingiber officinale).tw.
65. (Lead or Tin or Black bitumen or Black asphalt*).tw.
66. (Arogyavardhini* or Arogyvardhini* or Ayaskriti or Chandrakant* or Chandraprabh* or Devdarvarisht* or Devdarvarist* or Dhanvantar* or Gokshuradi* or Goksuradi* or Jambudyarisht*or Jambudyarist* or Katakakhadiradi* or Kathakakhadiradi* or Kshirabal* or Ksirabal* or Lodhrasav* or Mamajjak* or Nag* or Naag* or Nimbadi* or Nimadi* or Nishamalak* or Nisamalak* or Nishamlak* or Nisamlak* or Nishakathakadi* or Nisakathakadi* or Phalatrikadi* or Falatrikadi* or Rajanyamalakadi* or Rajanyamlakadi* or Saptamrit* or Shaptamrit* or Saptargangyadi* or Shilajit* or Silajit* or Shilajat* or Silajat* or Shilajeet* or Silajeet* or Siva* or Shiva* or Somnath* or Shomnath* or Triphal* or Trifal* or Trivang* or Tribang* or Vang* or Bang* or Vasant Kusumakar* or Vashant Kushumakar* or Vasanta Kusumakar* or Vashanta Kushumakar* or Vasantkusumakar* or Vashantkushumakar* or Vasantakusumakar* or Vashantakushumakar* or Basant Kusumakar* or Bashant Kushumakar* or Basanta Kusumakar* or Bashanta Kushumakar* or Basantkusumakar* or Bashantkushumakar* or Basantakusumakar* or Bashantakushumakar* or Vijayasaradi* or Vijayasharadi* or Vijaysaradi* or Vijaysharadi* or Bijayasaradi* or Bijayasharadi* or Bijaysaradi* or Bijaysharadi* or Vyoshadi* or Vyosadi* or Byoshadi* or Byosadi*).tw.
67. (Ayush 82 or Ayush-82 or Cogent DB or Diabecon or D 400 or D-400 or GS4 or Gurmar or Hyponidd or Inolter or M 93 or M-93 or MA 471 or MA-471 or Nosulin or Pancreas Tonic).tw.
68. 10 or 11 or 12 or 13 or 14 or 15 or 16 or 17 or 18 or 19 or 20 or 21 or 22 or 23 or 24 or 25 or 26 or 27 or 28 or 29 or 30 or 31 or 32 or 33 or 34 or 35 or 36 or 37 or 38 or 39 or 40 or 41 or 42 or 43 or 44 or 45 or 46 or 47 or 48 or 49 or 50 or 51 or 52 or 53 or 54 or 55 or 56 or 57 or 58 or 59 or 60 or 61 or 62 or 63 or 64 or 65 or 66 or 67
69. Randomised controlled trial.mp.
70. Random allocation.mp.
71. Double-blind method.mp.
72. Single-blind method.mp.
73. Cross-over studies.mp.
74. ((Random$ or control$ or clinical$) adj3 (trial$ or stud$)).tw.
75. (Random$ adj3 allocat$).tw.
76. Placebo$.tw.
77. ((Singl$ or doubl$ or trebl$ or tripl$) adj (blind$ or mask$)).tw.
78. (Crossover$ or (cross adj over$)).tw.
79. 69 or 70 or 71 or 72 or 73 or 74 or 75 or 76 or 77 or 78
80. 9 and 68 and 79

Turning Research Into Practice (TRIP) (1997-16 January 2021): 186 records
(Diabet* OR NIDDM OR T2DM OR T2D OR Prameh* OR Madhumeh*) AND Ayurved*

AYUSH Research Portal (16 January 2021): 292 records

Searched with AYUSH terminology -> Selected medical system - Ayurveda -> Selected disease from the list –
Madhumeha (283 records)
Madhumeha – (9 records)

Digital Helpline for Ayurveda Research Articles (DHARA) (16 January 2021): 1230 records
Single search terms were used.
Diabetes (662 records)
Diabetic (436 records)
Diabetics (30 records)
NIDDM (15 records)
T2DM (3 records)
T2D (3 records)
Madhumeha (31 records)
Prameha (50 records)

A Bibliography of Indian medicine (ABIM) (16 January 2021): 3015 records
Single search terms were used.
Diabetes (1404 records)
Diabetic (1404 records)
Diabetics (23 records)
NIDDM (29 records)
T2DM (0 records)
T2D (0 records)
Madhumeha (113 records)
Prameha (42 records)

CAM-QUEST (16 January 2021): 29 records
Selected disease pattern - metabolic disorders -> Selected disease - diabetes mellitus -> Selected therapy - Ayurveda

Directory of Open Access Journals (16 January 2021): 191 records
Three different searches were run.
Diabet* AND Ayurved* [All fields] (171 records)
Prameha [All fields] (11 records)
Madhumeha [All fields] (9 records)

EthOS (16 January 2021): 18 records
Ayurveda [any word] OR Ayurvedic [any word]

OpenGrey (16 January 2021): 22 records
Ayurved*

ProQuest Dissertations and Theses (16 January 2021): 400 records
Three different searches were run.
Ti(Diabet* OR NIDDM OR T2DM OR T2D) AND Ayurved* (111 records)
Prameh* (165 records)

Madhumeh* (124 records)

Appendix S2: Excluded studies with reasons for exclusion.

Ineligible participants (includes impossible to extract data on T2DM participants) (n=1)

1. Hsu P, Pan FF, Hsieh C. mcIRBP-19 of bitter melon peptide effectively regulates diabetes mellitus (DM) patients' blood sugar levels. *Nutrients*. 2020;12(5):1252.

Ineligible interventions (includes unclear interventions) (n=8)

1. Mohan V. Evaluation of D-400 (Diabecon) as anti-diabetic agent: A double blind placebo controlled trial in NIDDM patients with secondary failure to oral drugs. *Indian Journal of Clinical Practice*. 1998;8(9):18-21.
2. Elder C, Aickin M, Bauer V, Cairns J, Vuckovic N. Randomized trial of a whole-system Ayurvedic protocol for type 2 diabetes. *Altern Ther Health Med*. 2006;12(5):24-30.
3. Yin J, Xing H, Ye J. Efficacy of berberine in patients with type 2 diabetes mellitus. *Metabolism*. 2008;57(5):712-7.
4. Sharma RK, Patki PS. Double-blind, placebo-controlled clinical evaluation of an Ayurvedic formulation (GlucoCare capsules) in non-insulin dependent diabetes mellitus. *J Ayurveda Integr Med*. 2010;1(1):45-51.
5. Agrawal RP, Goyal S, Chopra A, Jain S, Agarwal V, Khatri MP, *et al*. A randomized placebo controlled trial of herbal medicine (Sugaradik) in the treatment of type 2 diabetes. *Indian Medical Gazette*. 2014;148(4):142-8.
6. Kurian GA, Manjusha V, Nair SS, Varghese T, Padikkala J. Short-term effect of G-400, polyherbal formulation in the management of hyperglycemia and hyperlipidemia conditions in patients with type 2 diabetes mellitus. *Nutrition*. 2014;30(10):1158-64.
7. Mirfeizi M, Tourzani ZM, Mirfeizi SZ, Jafarabadi MA, Rezvani HR, Afzali M. Controlling type 2 diabetes mellitus with herbal medicines: A triple-blind randomized clinical trial of efficacy and safety. *J Diabetes*. 2016;8(5):647-56.
8. Venkatesan H, Karthi SA. Hypoglycaemic effect of alcoholic extracts of the leaves of *Abroma augusta* and *Gymnema sylvestre* plants in type II diabetes mellitus patients. *Indian Journal of Public Health Research and Development*. 2020;11(7):293-9.

Ineligible outcomes (includes inadequate data and/or timings) (n=19)

1. Khan AK, Akhtar S, Mahtab H. Treatment of diabetes mellitus with *Coccinia indica*. *Br Med J*. 1980;280(6220):1044.
2. Niemi MK, Keinänen-Kiukaanniemi SM, Salmela PI. Long-term effects of guar gum and microcrystalline cellulose on glycaemic control and serum lipids in type 2 diabetes. *Eur J Clin Pharmacol*. 1988;34(4):427-9.
3. Lalor BC, Bhatnagar D, Winocour PH, Ishola M, Arrol S, Brading M, *et al*. Placebo-controlled trial of the effects of guar gum and metformin on fasting blood glucose and serum lipids in obese, type 2 diabetic patients. *Diabet Med*. 1990;7(3):242-5.
4. Agrawal P, Rai V, Singh RB. Randomized placebo-controlled, single blind trial of holy basil leaves in patients with noninsulin-dependent diabetes mellitus. *Int J Clin Pharmacol Ther*. 1996;34(9):406-9.
5. Anderson JW, Allgood LD, Turner J, Oeltgen PR, Daggy BP. Effects of psyllium on glucose and serum lipid responses in men with type 2 diabetes and hypercholesterolemia. *Am J Clin Nutr*. 1999;70(4):466-73.
6. Yadav RK, Mishra R, Chhipa RP, Audichya KC. Clinical trial of an indigenous compound drug Nishaamalki in the management of madhumeha vis-a-vis diabetes mellitus. *Anc Sci Life*. 2001;21(1):18-24.

7. Arora D, Kumar M, Dubey SD, Sings U. Immunomodulating effects of rasayana drugs in diabetics: A clinical study. *Anc Sci Life*. 2002;22(2):42-8.
8. Ludvik BH, Mahdjoobian K, Waldhaeusl W, Hofer A, Prager R, Kautzky-Willer A, *et al*. The effect of *Ipomoea batatas* (caiapo) on glucose metabolism and serum cholesterol in patients with type 2 diabetes: A randomized study. *Diabetes Care*. 2002;25(1):239-40.
9. John AJ, Cherian R, Subhash HS, Cherian AM. Evaluation of the efficacy of bitter gourd (*Momordica charantia*) as an oral hypoglycemic agent: A randomized controlled clinical trial. *Indian J Physiol Pharmacol*. 2003;47(3):363-5.
10. Jayawardena MH, de Alwis NM, Hettigoda V, Fernando DJ. A double blind randomized placebo controlled cross over study of a herbal preparation containing *Salacia reticulata* in the treatment of type 2 diabetes. *J Ethnopharmacol*. 2005;97(2):215-8.
11. Ryu OH, Lee J, Lee KW, Kim HY, Seo JA, Kim SG, *et al*. Effects of green tea consumption on inflammation, insulin resistance and pulse wave velocity in type 2 diabetes patients. *Diabetes Res Clin Pract*. 2006;71(3):356-8.
12. Inayat-ur-Rahman, Malik SA, Bashir M, Khan R, Iqbal M. Serum sialic acid changes in non-insulin-dependant diabetes mellitus (NIDDM) patients following bitter melon (*Momordica charantia*) and rosiglitazone (Avandia) treatment. *Phytomedicine*. 2009;16(5):401-5.
13. Ahmed F, Hudeda S, Urooj A. Antihyperglycemic activity of *Ficus racemosa* bark extract in type 2 diabetic individuals. *J Diabetes*. 2011;3(4):318-9.
14. Amarnath S, Shukla HM. Single blind placebo controlled clinical evaluation of Mamajjaka Ghanvati in prameha with special reference to diabetes mellitus. *Journal of Ayurveda and Holistic Medicine*. 2013;1(5):11-9.
15. Jani DK. Clinical effect of supplementation of *Solanum nigrum* in diabetic individuals. *International Journal of Pharmacy and Integrated Life Sciences*. 2014;2(4):76-84.
16. Behzadi AA, Kalalian-Moghaddam H, Ahmadi AH. Effects of *Urtica dioica* supplementation on blood lipids, hepatic enzymes and nitric oxide levels in type 2 diabetic patients: A double blind, randomized clinical trial. *Avicenna J Phytomed*. 2016;6(6):686-95.
17. Pachaghare M. Randomized open clinical study of Charakokta Mutrasangrahniya Mahakashay with special reference to madhumeh i.e. diabetes mellitus. *Deerghayu International*. 2016;32-02(126):254-8.
18. Sharma SD, Masand S, Sharma GD, Chaudhary V, Sharma SD. A randomized clinical study to evaluate the efficacy of an Ayurvedic formulation in the management of madhumeha with special reference to diabetes mellitus. *International Journal of Research in Ayurveda and Pharmacy*. 2016;7(6):33-8.
19. Hadi S, Mirmiran P, Daryabeygi-Khotbesara R, Hadi V. Effect of *Nigella sativa* oil extract on inflammatory cytokine response and oxidative stress among people with type 2 diabetes mellitus: A randomized, double-blind, placebo controlled trial. *Progress in Nutrition*. 2018;20(1-S):127-33.

Ineligible study designs (n=34)

1. Singh TN, Upadhyay BN, Tewari CM, Tripathi SN. Management of diabetes mellitus (prameha) with *Inula racemosa* and *Cinnamomum tamala*. *Anc Sci Life*. 1985;5(1):9-16.
2. Kohli KR, Naik S, Tripathi R, Zarapkar M, Kenkare D. Karnim: A herbal formulation in non-insulin dependent diabetes mellitus. *The Journal of General Medicine*. 1993;6(1):47-52.
3. Kohli KR, Singh RH. A clinical trial of jambu (*Eugenia jambolana*) in non-insulin dependant diabetes mellitus. *Journal of Research in Ayurveda and Siddha*. 1993;14(3-4):89-97.
4. Kamble SM, Jyotishi GS, Kamlakar PL, Vaidya SM. Efficacy of *Coccinia indica* W. and A. in diabetes mellitus. *Journal of Research in Ayurveda and Siddha*. 1996;17(1-2):77-84.

5. Sharma RD, Sarkar A, Hazra DK, Mishra B, Singh JB, Sharma SK, *et al*. Use of fenugreek seed powder in the management of non-insulin dependent diabetes mellitus. *Nutr Res*. 1996;16(8):1331-9.
6. Sharma RD, Sarkar A, Hazra DK, Misra B, Singh JB, Maheshwari BB, *et al*. Hypolipidaemic effect of fenugreek seeds: A chronic study in non-insulin dependent diabetic patients. *Phytother Res*. 1996;10(4):332-4.
7. Bordia A, Verma SK, Srivastava KC. Effect of ginger (*Zingiber officinale* Rosc.) and fenugreek (*Trigonella foenumgraecum* L.) on blood lipids, blood sugar and platelet aggregation in patients with coronary artery disease. *Prostaglandins Leukot Essent Fatty Acids*. 1997;56(5):379-84.
8. Chandra V, Aruna K. Glycaemic and cholesterolaemic effect of sprouted fenugreek on diabetic subjects. *The Indian Journal of Nutrition and Dietetics*. 1997;34(10):246-51.
9. Mehra PS, Singh RH. Clinical evaluation of the effect of Amrita-Pippali-Nimba Yoga in diabetes mellitus with special reference to the role of agni and ojas. *Journal of Research in Ayurveda and Siddha*. 2001;21(3-4):183-97.
10. Adhikari KS, Ojha JK, Dwivedi KN. A clinical study of Shilajatu Bijaka Yoga in ojomeha patients. *Journal of Research in Ayurveda and Siddha*. 2003;24(1-2):62-70.
11. Pandey AK, Singh RH. A study of the immune status in patients of diabetes mellitus and their management with certain naimittika rasayana drugs. *Journal of Research in Ayurveda and Siddha*. 2003;24(3-4):48-61.
12. Islas IL, Nuevo JJ, Saab NG, Guerra AF. Onion consumption as an adjuvant in glycemic control of type 2 diabetes. *Medicina Interna de Mexico*. 2005;21(1):44-7.
13. Andallu B, Ramya V. Anti-hyperglycemic, cholesterol-lowering and HDL-raising effects of cumin (*Cuminum cyminum*) seeds in type 2 diabetes. *Journal of Natural Remedies*. 2007;7(1):142-9.
14. Joshi A. Studies on the impact of phytochemical rich fruit supplementation amla *Embilica officinalis* in the management of diabetes mellitus and efficacy of interpersonal counselling on the knowledge and lifestyle related risk factors in type 2 diabetes mellitus [dissertation on the internet]. Baroda: Maharaja Sayajirao University of Baroda; 2008.
15. Mitra A. Preparation and effects of cheap salad oil in the management of type 2 rural Indian diabetics. *Journal of Human Ecology*. 2008;23(1):27-38.
16. Kochhar A, Sharma N, Sachdeva R. Effect of supplementation of tulsi (*Ocimum sanctum*) and neem (*Azadirachta indica*) leaf powder on diabetic symptoms, anthropometric parameters and blood pressure of non insulin dependent male diabetics. *Studies on Ethno-Medicine*. 2009;3(1):5-9.
17. Radha R, Amrithaveni M. Role of medicinal plant *Salacia reticulata* in the management of type II diabetic subjects. *Anc Sci Life*. 2009;29(1):14-6.
18. Sartore G, Reitano R, Barison A, Magnanini P, Cosma C, Burlina S, *et al*. The effects of psyllium on lipoproteins in type II diabetic patients. *Eur J Clin Nutr*. 2009;63(10):1269-71.
19. Kumar SN, Mani UV, Mani I. An open label study on the supplementation of *Gymnema sylvestre* in type 2 diabetics. *J Diet Suppl*. 2010;7(3):273-82.
20. Venugopal S, Iyer UM. Management of diabetic dyslipidemia with subatmospheric dehydrated barley grass powder. *International Journal of Green Pharmacy*. 2010;4(4):251-6.
21. Anas M, Mohsin M, Siddiqui M, Mannan A. Therapeutic evaluation of a polyherbal formulation in type 2 diabetes mellitus. *Indian Journal of Traditional Knowledge*. 2011;10(4):711-5.
22. Nath P, Dubey SD, Ojha JK. Study on ojah kshaya WSR to madhumeha hara dravyas in Samhitas. *Journal of Research in Ayurveda and Siddha*. 2011;32(3-4):87-100.
23. Tanna I, Chandola HM, Joshi JR. Clinical efficacy of Mehamudgara Vati in type 2 diabetes mellitus. *Ayu*. 2011;32(1):30-9.

24. Upadhyay BN, Gupta V. A clinical study on the effect of Rishyagandha (*Withania coagulans*) in the management of prameha (type II diabetes mellitus). *Ayu*. 2011;32(4):507-11.
25. Hoehn AN, Stockert AL. The effects of *Cinnamomum cassia* on blood glucose values are greater than those of dietary changes alone. *Nutr Metab Insights*. 2012;5:77–83.
26. Singh U, Kochhar A. Effect of supplementation of bael (*Aegle marmelos* L.) and nutrition counseling on blood glucose, lipid profile and blood pressure of non-insulin dependent diabetics. *Journal of Pharma Research*. 2012;1(2):1-6.
27. Kim HM, Kim J. The effects of green tea on obesity and type 2 diabetes. *Diabetes Metab J*. 2013;37(3):173-5.
28. Sharma P, Sharma AK, Dwivedi KN. Clinical study on antidiabetic activity of karanja (*Pongamia pinnata*). *International Journal of Pharmaceutical Research and Bio-Science*. 2013;2(5):438-51.
29. Tayyab F, Lal SS. Antidiabetic hypolipidemic and antioxidant activity of *Momordica charantia* on type-II diabetic patient in Allahabad India. *International Journal of Pharma and Bio Sciences*. 2013;4(4):932-40.
30. Choudhary M, Kochhar A, Sangha J. Hypoglycemic and hypolipidemic effect of *Aloe vera* L. in non-insulin dependent diabetics. *J Food Sci Technol*. 2014;51(1):90-6.
31. Mathew R, Radhakrishnan VN, Babu NS. Effect of Katakakhadiradi Kasaya in type 2 diabetes mellitus associated with hypercholesterolemia. *Aryavaidyan*. 2014;28(1):27-30.
32. Balavenkata KS, Bulusu S. A critical study of pramehahara effect of Dhaatri Nishe WSR to diabetes mellitus. *International Journal of Ayurvedic Medicine*. 2018;9(3):196-201.
33. Boston C, Wong N, Ganga T, Chandradatt K, Rosales J, Singh J, *et al*. Comparison and effectiveness of complementary and alternative medicine as against conventional medicine in the treatment and management of type 2 diabetes. *Journal of Complementary and Alternative Medical Research*. 2019;7(2):1-8.
34. Sehgal S, Huddar VG, Rao MV. Efficacy of prakriti specific herbal tea in diabetes: A randomized controlled trial. *International Journal of Ayurvedic Medicine*. 2020;11(3):524-35.

Ineligible participants (includes impossible to extract data on T2DM participants)+ineligible study designs (n=7)

1. Rajasekharan S, Tuli SN. Vijaysar (*Pterocarpus marsupium*) in the treatment of madhumeha (diabetes mellitus): A clinical trial. *Journal of Research Indian Medicine, Yoga and Homoeopathy*. 1976;11:9-14.
2. Shaw BP, Tripathy PC. Evaluation of Svarnamakshika (copper pyrites) as an oral hypoglycaemic agent (clinical trials). *Nagarjun*. 1980;23(1-9):195-7.
3. Mishra SK, Pandey SK, Sharma SH, Singh GD. Effect of Amalaki-Haridra on madhumeha. *Nagarjun*. 1983;26(1-5):105-7.
4. Chaube A, Nagraja TN, Dixit SK, Agrawal JK, Kumar M, Prakash B. A novel Ayurvedic anti diabetic medicine. *Anc Sci Life*. 1995;15(2):153-5.
5. Rajagopalan K, Sasidharan K. Evaluation of the clinical management of prameha roga (diabetes mellitus). *Aryavaidyan*. 2000;14(1):33-43.
6. Kanthi VG, Sarashetti RS, Bidari RC. Management of madhumeha (diabetes mellitus) with herbomineral compound preparations. *Journal of Research in Ayurveda and Siddha*. 2004;25(3-4):47-56.
7. Gaikwad S, Kadam DB, Pawar PS. Role of Ayurvedic treatment in the management of diabetes. *Aryavaidyan*. 2009;22(4):202-5.

Ineligible interventions (includes unclear interventions)+ineligible study designs (n=4)

1. Maji D, Singh AK. Clinical trial of D-400, a herbomineral preparation in diabetes mellitus. *Journal of the Diabetic Association of India*. 1995;35(1):1-4.

2. Mitra SK, Hemavathi TS, Mohan AR, Rajesha. Anti-diabetic effect of D-400, an Ayurvedic herbal preparation in non-insulin dependent diabetes mellitus patients. *The Antiseptic*. 1995;92(9):342-5.
3. Mitra SK, Seshaiah V, Agarwal JK, Maji D, Yajnik VH, Prasanna Kumar KM, *et al*. Multicentric trial of Diabecon (D-400): A herbomineral preparation on lipid profile in diabetes mellitus. *Int J Diabetes Dev Ctries*. 1996;16:87-9.
4. Geetha B, Shivananda N, Manjula S. Management of newly diagnosed type 2 diabetes by *Trigonella foenum-graecum*. *International Journal of Research in Ayurveda and Pharmacy*. 2011;2(4):1231-4.

Ineligible outcomes (includes inadequate data and/or timings)+ineligible study designs (n=11)

1. Ojha JK, Bajpai HS, Sharma PV. Hypoglycemic effect of *Pterocarpus marsupium* Roxb (vijaysar). *Journal of Research Indian Medicine, Yoga and Homoeopathy*. 1978;13(4):12-6.
2. Chandola HM, Tripathi SN, Udupa KN. Hypoglycaemic response of *C. tamala* in patients of maturity onset (insulin independent) diabetes. *Journal of Research in Ayurveda and Siddha*. 1980;1:275-90.
3. Chandola HM, Tripathi SN, Udupa KN. Effect of *C. tamala* on plasma insulin vis-à-vis blood sugar in patients of diabetes mellitus. *Journal of Research in Ayurveda and Siddha*. 1980;1(3):345-57.
4. Jain AK, Shaw BP. Effect of herbal compound on maturity onset diabetes. *Anc Sci Life*. 1987;7(1):12-6.
5. Kumar N, Kumar A. A clinical trial of M-93 compound in the management of madhumeha (diabetes mellitus). *Journal of Research in Ayurveda and Siddha*. 1995;16(3-4):102-7.
6. Nanda GC, Chopra KK, Sahu DP, Padhi MM. Nishamalaki in madhumeha (NIDDM): A clinical study. *Journal of Research in Ayurveda and Siddha*. 1998;19(1-2):34-40.
7. Kumar N, Kumar A, Sharma ML. Clinical evaluation of single and herbo-mineral compound drugs in the management of madhumeha. *Journal of Research in Ayurveda and Siddha*. 1999;20(1-2):1-9.
8. Mudra M, Ercan-Fang N, Zhong L, Furne J, Levitt M. Influence of mulberry leaf extract on the blood glucose and breath hydrogen response to ingestion of 75 g sucrose by type 2 diabetic and control subjects. *Diabetes Care*. 2007;30(5):1272-4.
9. Date KA, Dwivedi LK, Sharma RP, Rao KS. Comparative study of Ganesayagokta Havanasamagribhasma and Silabhrarasa with Madhumeharicurna with special reference to its anti-hyperglycemic effect. *Aryavaidyan*. 2014;28(1):47-51.
10. Kumari M, Jain S, Dave R. Babul (*Acacia nilotica*): A potential source of tannin and its suitability in management of type II diabetes. *Nutrition and Food Science*. 2014;44(2):119-26.
11. Kapoor S. Impact of indigenous food (Methi dana, Kale til and Kali jiri) on blood glucose level of middle aged NIDDM patients [dissertation on the internet]. Indore: Devi Ahilya Vishwavidyalaya; 2015.

Ineligible participants (includes impossible to extract data on T2DM participants)+ineligible outcomes (includes inadequate data and/or timings)+ineligible study designs (n=3)

1. Atique A, Iqbal M, Ghouse AK. Use of *Annona squamosa* and *Piper nigrum* against diabetes. *Fitoterapia*. 1985;56(3):190-2.
2. Fernando MR, Wickramasinghe SM, Thabrew MI, Ariyananda PL, Karunanayake EH. Effect of *Artocarpus heterophyllus* and *Asteracanthus longifolia* on glucose tolerance in normal human subjects and in maturity-onset diabetic patients. *J Ethnopharmacol*. 1991;31(3):277-82.

3. Karnick CR. A clinical trial of a composite herbal drugs in the treatment of diabetic mellitus (madhumeha). *Aryavaidyan*. 1991;5(1):36-46.

Ineligible interventions (includes unclear interventions)+ineligible outcomes (includes inadequate data and/or timings)+ineligible study designs (n=1)

1. Khan SK. South Asian and Chinese medical systems: Ayurveda and traditional Chinese medicine treatments for diabetes mellitus, type 2 [dissertation on the internet]. New York: City University of New York; 2006.

Ineligible participants (includes impossible to extract data on T2DM participants)+ineligible comparators+ineligible outcomes (includes inadequate data and/or timings)+ineligible study designs (n=1)

1. Shaw BP, Gupta S. A clinical study of bimbi (*Coccinia indica*) in the treatment of madhumeha (diabetes mellitus). *Nagarjun*. 1981;14(6):24-6.

Full text unavailable (n=1)

1. Bhimani S. Clinical study of efficacy of Glycoban cap in patients of non-insulin dependent diabetes (NIDDM) mellitus. *The Antiseptic*. 2005;102(2):71-5.

Appendix S3: Potentially eligible ongoing RCTs.

Trial registry number	Scientific study title	Country
CTRI/2015/03/005660	A comparative clinical study to evaluate the efficacy of Phalendradhi Yogam with and without Yashada Bhasma in the management of madhumeha (type 2 DM)	India
CTRI/2015/06/005891	A randomised, prospective single blind clinical trial of anti diabetic polyherbal formulation (DiaKure)	India
CTRI/2017/05/008451	Pharmaceutical development of trinshati and shashti puti Naga Bhasma and their comparative clinical efficacy in the management of madhumeha (type 2 diabetes)	India
CTRI/2017/05/008461	Open labelled, controlled, randomized evaluation of the effect of Dhatri Nisha Yoga in management of prameha vis a vis diabetes mellitus (type II)	India
CTRI/2017/08/009575	Role of Vijaysaradi capsule in the management of insulin dependent type 2 diabetes: An open labelled standard controlled clinical trial	India
CTRI/2018/04/013415	Clinical evaluation of the effect of Phalatrikadi Kwath and Madhumeha-har Vati Kalpit Yoga in madhumeh (diabetes mellitus II)	India
CTRI/2018/09/015829	A comparative pharmaceutico-analytical and clinical study of Indravati and Vangeshwara Rasa in madhumeha	India
CTRI/2019/02/017474	Efficacy and safety of *Curcuma longa* as an add-on therapy in type 2 diabetes mellitus: A randomized, double blind, placebo controlled trial	India
CTRI/2019/02/017500	A randomised control trial for the mechanism of action of yoga and Ayurveda with yoga in type-2 diabetes mellitus	India
CTRI/2019/02/017609	Randomized controlled clinical study on the efficacy of Vidangadi Lauha on sthula madhumeha with special reference to type II diabetes mellitus	India
CTRI/2019/02/017755	A clinical study to evaluate the efficacy of pathya, shodhana, shamana and rasayana chikitsa in the management of madhumeha WSR to type II diabetes mellitus	India

CTRI/2019/04/018729	A comparative clinical study to evaluate the efficacy of Nyagrodhadi Churna and B-S Patra Ghana Vati in the management of madhumeha WSR type-2 diabetes mellitus	India
CTRI/2019/05/019370	An open label double arm clinical trial to determine the efficacy of D 4 combination along with ongoing conventional medicine in managing diabetes mellitus (type 2)	India
CTRI/2019/05/019452	A randomized, multi-center, open labeled, comparative, prospective clinical study to evaluate efficacy and safety of Yesaka liquid as an add-on therapy to oral hypoglycemic agent (OHA) in type 2 diabetic patients	India
CTRI/2019/06/019636	An open-label, prospective, observational, multiple-dose, controlled, two armed, relative study of bio active liquid curcumin 5mg/50ml (5 drops in 50 ml of water) of oral dose (TDS dose) along with conventional pharmaceutical treatment (only prescribed diabetic medicine) in diabetic, adult, human patients to evaluate the safety and efficacy	India
CTRI/2020/01/023027	A double blind randomised placebo controlled evaluation of adjuvant effect of Kimshukatvagadi Ghana capsule in prameha with special reference to diabetes mellitus type II	India
CTRI/2020/02/023118	A comparative control clinico-biochemical study of Ayurvedic formulation in the patients of type 2 diabetes mellitus WSR madhumeha	India
CTRI/2020/03/023876	An open label randomised clinical trial to determine the efficacy of Rohitakadi Churna along with Triphaladi granules in the management of madhumeha with special reference to diabetes mellitus (type 2)	India
CTRI/2020/06/025922	Efficacy of vamana, virechana and basti followed by shamana in the management of sthula pramehi WSR to type 2 diabetes: An open labelled randomized standard controlled clinical trial	India
CTRI/2020/07/026465	Association study between sharir prakriti and type 2 diabetes (madhumeha) and its Ayurvedic management along with lifestyle modifications	India
CTRI/2020/10/028201	An open labelled randomized controlled clinical study to evaluate the anti-hyperglycemic activity of novel Ayurvedic Choorna in the management of prameha vis-a-vis type 2 diabetes mellitus	India
CTRI/2020/12/029806	Study the effect of Nisha-amalaki (as an adjuvant drug) in uncontrolled type II diabetes mellitus: A pragmatic, randomized controlled trial	India
IRCT2016042717254N5	The effects of green cardamom supplementation on blood glucose, lipids profile, oxidative stress, sirtuin-1 and irisin in type 2 diabetic patients: A study protocol for a randomized placebo-controlled clinical trial<br>The effects of green cardamom supplementation on blood pressure and endothelium function in type 2 diabetic patients: A study protocol for a randomized controlled clinical trial	Iran
NCT03151837	A randomized, double-blind, placebo-controlled trial to evaluate the hypoglycemic efficacy of greenyn *Momordica charantia* extracts in diabetic subjects	Taiwan
NCT04023539	Evaluation of the effect of *Cinnamomum zeylanicum* on glycemic levels of Mexican adult patients with type 2 diabetes at 3 months: Randomized clinical trial	Mexico
NCT04259606	Effect of Cassia Cinnamon on arterial stiffness parameters in patients with type 2 diabetes mellitus	Mexico

RBR-2KKB6D	Analysis of the effectiveness of cinnamon (*Cinnamomum verum*) in the reduction of glycemic and lipidic levels of adults with type 2 diabetes: A study protocol	Brazil
SLCTR/2017/010	Effectiveness of *Cinnamomum zeylanicum* (Ceylon cinnamon) in lowering blood glucose in type 2 diabetes mellitus: A randomized double blind placebo controlled clinical trial	Sri Lanka

Appendix S4: Characteristics of included studies.

Author and year	Country	T2DM patients (duration of T2DM if existing cases [mean, years])	Comorbidities/ complications of T2DM	Participants /eligible arm (n)	Age (mean, years)	Females (n)	HbA1c (mean, %)	FBG (mean, mg/dL)	Any commercial funding/other support
**Ayurvedic medicine- single plant- or mineral-origin ingredient**									
Abelmoschus esculentus									
Moradi 2020	Iran	Existing cases (4.3)/on OAD		A1=30, A2=30	53.8	44	7.8	183.0	Unclear
Acacia Senegal									
Babiker 2017, Babiker 2018	Sudan	Existing cases (5.1Ω)/on OAD		A1=50, A2=50	50.1Ω	73Ω	9.4Ω	182.1Ω	Unclear
Aegle marmelos									
Sankhla 2009	India¥	Existing cases (NS)/on OAD		A1=10, A2=10	NS	0	NS	135.0Ω	Unclear
Sharma 2013	India	Newly diagnosed/treatment naïve		A1=100✪, A2=50	NS	88	8.7	225.0	Unclear
Nigam 2019	India	Existing cases (6.6Ω)/on OAD		A1=31, A2=34	51.4Ω	35Ω	9.2Ω	171.5Ω	No?
Allium sativum									
Ashraf 2005	Pakistan	Existing cases (NS)/on OAD	Dyslipidemia	A1=35, A2=35	59.0	38	NS	NS	Unclear
Ashraf 2011a	Pakistan ¥	Existing cases (2.8)/on OAD?		A1=150✪, A2=30, A3=30	45.0	88	6.6	127.4	Unclear
Ashraf 2011b	Pakistan ¥	Newly diagnosed/treatment naïve		A1=30, A2=30	37.5	27	NS	120.6	Unclear
Kumar 2013	India	Existing cases (NS)/on OAD	Obesity	A1=30, A2=30	53.3	NS	7.5	158.7	Unclear
Aloe vera									
Arora 2009	India	Existing cases (NS)/on OAD		A1=14, A2=12	NS	NS	8.7Ω	180.4Ω	Unclear
Huseini 2012a, Huseini 2012b	Iran	Existing cases (9.9?)/on OAD		A1=35?, A2=35?	54.0?	38?	7.2?	181.1?	No
Zarrintan 2015	Iran	Existing cases (NS)/on OAD		A1=22, A2=22	52.6	19	8.6	175.4	Unclear
Anethum graveolens									
Mobasseri 2014	Iran	Existing cases (NS)/on OAD		A1=30, A2=30	53.1Ω	28Ω	NS	141.2Ω	No
Haidari 2020	Iran	Existing cases (8.3Ω)/on OAD	Gastrointestinal symptoms	A1=24, A2=24	50.5Ω	30Ω	NS	147.2Ω	No
Azadirachta indica									
Usharani 2020	India	Existing cases (NS)/on OAD		A1=60✪, A2=20	53.6	34	7.4Ω	119.3Ω	Yes
Berberis aristata									

Author and year	Country	T2DM patients (duration of T2DM if existing cases [mean, years])	Comorbidities/ complications of T2DM	Participants /eligible arm (n)	Age (mean, years)	Females (n)	HbA1c (mean, %)	FBG (mean, mg/dL)	Any commercial funding/other support
Sharma 2017	India	Existing cases (NS)/on OAD	Dyslipidemia	A1=68?✪, A2=35?	NS	NS	NS	163.4Ω	Unclear
Boswellia serrata									
Azadmehr 2014	Iran	Existing cases (5.7)/on OAD		A1=37, A2=34	51.1	29	7.3	156.5	No
Mehrzadi 2018	Iran	Existing cases (11.1)/on OAD		A1=27, A2=29	54.9	30	8.2	167.0	No
Camellia sinensis									
MacKenzie 2007	US	Existing cases (7.0Ω)/on OAD		A1=36✪, A2=18✪	65.4Ω	28Ω	7.1Ω	NS	Yes?
Mirzaei 2009	Iran	Existing cases (5.7)/on OAD		A1=26, A2=46	54.6	58?	7.7	170.4	No
Hsu 2011	Taiwan	Existing cases (4.2Ω)/on OAD	Obesity	A1=40, A2=40	51.4Ω	44Ω	8.4Ω	172.8Ω	No
Lasaite 2014	Lithuania	Existing cases (8.1Ω)/on OAD and/or insulin	Diabetic retinopathy, nephropathy, or neuropathy?	A1=83?, A2=84?	57.0Ω	NS	8.0Ω	NS	Yes?
Liu 2014	Taiwan	Existing cases (4.7Ω)/on OAD	Dyslipidemia	A1=46, A2=46	54.3Ω	45Ω	7.6Ω	145.7Ω	No
Quezada-Fernandez 2019	Mexico	Existing cases (6.0Ω)/on OAD		A1=12, A2=13	53.2Ω	10Ω	7.5Ω	169.0Ω	Unclear
Capparis spinosa									
Huseini 2013	Iran	Existing cases (6.9Ω)/on OAD		A1=30, A2=30	53.9Ω	NS	8.3Ω	180.2Ω	No
Cichorium intybus									
Chandra 2020	India	Existing cases (4.6Ω)/on OAD		A1=56, A2=55	47.0Ω	45Ω	8.6Ω	197.0Ω	Yes
Cinnamomum aromaticum									
Mang 2006	Germany	Existing cases (7.0Ω)/on OAD		A1=40?, A2=39?	63.3Ω	21Ω	6.8Ω	162.0Ω	Yes
Suppapitiporn 2006	Thailand	Existing cases (4.5)/on OAD		A1=20, A2=40	59.2	32	8.1	149.3	Unclear
Blevins 2007	US	Existing cases (NS)/on OAD		A1=30, A2=30	60.8Ω	29Ω	7.2Ω	138.8Ω	No
Crawford 2009	US¥	Existing cases (NS)/on OAD and/or insulin		A1=55, A2=54	60.2	45	8.4	NS	No?
Akilen 2010	UK¥	Existing cases (5.8)/on OAD		A1=30, A2=28	54.7	32	8.4	158.4	Yes
Wainstein 2011	Israel	Existing cases (8.2)/on OAD		A1=29, A2=30	63.1	24	7.6	155.0	Unclear
Lu 2012	China	Existing cases (NS)/on OAD?		A1=46?✪, A2=23?	60.4Ω	44	8.9Ω	174.6Ω	Unclear

Author and year	Country	T2DM patients (duration of T2DM if existing cases [mean, years])	Comorbidities/ complications of T2DM	Participants /eligible arm (n)	Age (mean, years)	Females (n)	HbA1c (mean, %)	FBG (mean, mg/dL)	Any commercial funding/other support
Sharma 2012	India	Newly diagnosed/treatment naïve		A1=100✪, A2=50	NS	56	8.4	223.5	Unclear
Hasanzade 2013	Iran	Newly diagnosed/treatment naïve?		A1=35, A2=36	54.2	47	8.7	168.0	No
Tangvarasittichai 2015, Sengsuk 2016	Thailand	Existing cases (8.6Ω)/on OAD		A1=54?, A2=60?	57.2?Ω	74?Ω	7.9?▼Ω	145.8?▼Ω	No
Cinnamomum verum									
Vafa 2012	Iran	Existing cases (6.5Ω)/on OAD		A1=22, A2=22	54.9Ω	24Ω	7.3Ω	137.8Ω	No
Zahmatkesh 2012¤	Iran	Both (7.5)		A1=31, A2=30	54.7	45	9.1Ω	185.0Ω	Unclear
Talaei 2017	Iran	Existing cases (NS)/on OAD		A1=22, A2=22	57.6Ω	24Ω	10.2Ω	187.2Ω	No
Zare 2019	Iran	Existing cases (5.4Ω)/on OAD		A1=70✪, A2=70✪	52.7Ω	63Ω	7.4Ω	161.0Ω	Unclear
Mirmiranpour 2020	Iran	Existing cases (NS)/on OAD		A1=34, A2=34	58.5Ω	31Ω	NS	NS	No?
Citrullus colocynthis									
Huseini 2009	Iran	Existing cases (4.9)/on OAD		A1=25, A2=25	54.2	38	9.8	180.6	No
Barghamdi 2016	Iran	Existing cases (7.5)/on OAD		A1=35, A2=35	53.0	40	8.1	164.9	No
Coccinia grandis									
Kuriyan 2008, Kurpad 2008	India	Newly diagnosed/treatment naïve		A1=30, A2=30	47.1Ω	26Ω	6.6Ω	128.7Ω	Yes
Quamri 2017	India	Existing cases (NS)/on OAD	Dyslipidemia	A1=20, A2=20	44.1	21	NS	NS	No
Wasana 2021	Sri Lanka	Newly diagnosed/treatment naïve		A1=79, A2=79	48.6	96	6.4	133.7	Unclear
Convolvulus prostratus									
Patel 2012	India	Existing cases (NS)/on OAD		A1=45, A2=48	NS	NS	NS	182.4Ω	Unclear
Crocus sativus									
Milajerdi 2018	Iran	Existing cases (NS)/on OAD	Anxiety and depression?	A1=27, A2=27	54.6	42	6.6Ω	162.0Ω	Yes
Aleali 2019	Iran	Existing cases (6.3Ω)/on OAD		A1=38, A2=38	53.0Ω	45Ω	8.9Ω	175.2Ω	No
Ebrahimi 2019	Iran	Existing cases (7.2Ω)/on OAD		A1=45, A2=45	54.1Ω	44Ω	7.7Ω	163.8Ω	Unclear
Mobasseri 2020	Iran	Existing cases (NS)/on OAD		A1=30, A2=30	51.1Ω	NS	NS	135.1Ω	No
Cuminum cyminum									

Author and year	Country	T2DM patients (duration of T2DM if existing cases [mean, years])	Comorbidities/ complications of T2DM	Participants /eligible arm (n)	Age (mean, years)	Females (n)	HbA1c (mean, %)	FBG (mean, mg/dL)	Any commercial funding/other support
Jafari 2017	Iran	Existing cases (NS)/on OAD		A1=66✪, A2=33	47.3Ω	62Ω	7.0Ω	166.6Ω	Unclear
Hendre 2020	India	Existing cases (NS)/on OAD		A1=100, A2=100	NS	NS	NS	151	No
Curcuma longa									
Usharani 2008	India	Existing cases (NS)/on OAD		A1=24?, A2=24?	52.6Ω	21Ω	7.9Ω	158.1Ω	Yes
Na 2013	China	Existing cases (7.7Ω)/on OAD and/or insulin	Obesity	A1=55, A2=54	55.1Ω	51Ω	7.7Ω	153.0Ω	Unclear
Chuengsamarn 2014	Thailand	Newly diagnosed/treatment naïve		A1=120, A2=120	59.4Ω	116Ω	7.0Ω	141.5Ω	No
Panahi 2017, Panahi 2018	Iran	Both (NS)		A1=61, A2=57	42.0Ω	49Ω	7.5Ω	168.5Ω	Yes
Adab 2019	Iran	Existing cases (NS)/on OAD	Dyslipidemia	A1=40, A2=40	55.2Ω	39Ω	6.9Ω	131.9Ω	No
Adibian 2019	Iran	Existing cases (8.0Ω)/on OAD		A1=25, A2=28	59.0Ω	22Ω	NS	NS	Unclear
Srinivasan 2019	India	Existing cases (3.9Ω)/on OAD		A1=69, A2=67	50.6Ω	85Ω	NS	NS	Yes
de Sousa 2020	Brazil¥	Existing cases (NS)/on OAD		A1=47, A2=46	62.6Ω	47Ω	7.9Ω	171.4Ω	No
Cyamopsis tetragonoloba									
Uusitupa 1984	Finland	Newly diagnosed/treatment naïve		A1=10, A2=9	62.0Ω	18	NS	174.8Ω	Unclear
Uusitupa 1989	Finland	Existing cases (9.4)/on OAD		A1=20, A2=19	60.1	26	9.1	225.0	Unclear
Eclipta prostrata									
Sazia 2015a, Sazia 2015b	India	Newly diagnosed/treatment naïve		A1=24, A2=24, A3=24	NS	NS	NS	127.1Ω	No?
Elettaria cardamomum									
Aghasi 2019	Iran	Existing cases (7.7)/on OAD		A1=41, A2=42	53.6	39	8.0	157.0	No?
Emblica officinalis									
Usharani 2013	India	Existing cases (NS)/on OAD		A1=44?✪, A2=22?	57.4Ω	20Ω	7.7Ω	NS	Yes
Enicostemma axillare									
Shankarrao 2017	India	Newly diagnosed/treatment naïve	Obesity	A1=20, A2=20	NS	NS	NS	NS	Unclear
Gynostemma pentaphyllum									
Huyen 2010	Vietnam ¥	Newly diagnosed/treatment naïve		A1=12, A2=12	60.4	7	8.8	174.6	No
Huyen 2012	Vietnam ¥	Newly diagnosed/treatment naïve		A1=12, A2=13	55.0	10	8.9	167.4	No

Author and year	Country	T2DM patients (duration of T2DM if existing cases [mean, years])	Comorbidities/ complications of T2DM	Participants /eligible arm (n)	Age (mean, years)	Females (n)	HbA1c (mean, %)	FBG (mean, mg/dL)	Any commercial funding/other support
Hibiscus sabdariffa									
Sarbini 2019	Indonesia¥	Both (7.4Ω)		A1=30, A2=30	55.1Ω	40Ω	NS	150.6Ω	Unclear
Ipomoea batatas									
Ludvik 2004	Switzerland¥	Both (NS)?		A1=30, A2=31	55.4	27	7.1	144.0	Yes
Ludvik 2008	Austria?, Germany?¥	Both (3.9Ω)?		A1=44?, A2=44?	59.2Ω	29Ω	6.4Ω	138.5Ω	Yes
Juglans regia									
Hosseini 2014a	Iran	Existing cases (5.7Ω)/on OAD		A1=31, A2=31	57.2Ω	37	8.4Ω	166.5Ω	No
Hosseini 2014b	Iran	Existing cases (6.4)/on OAD		A1=32, A2=29	55.1	33	8.4	166.5	No
Abdoli 2017	Iran	Existing cases (7.1Ω)/on OAD		A1=25, A2=25	55.8Ω	25Ω	8.0Ω	160.8Ω	No
Zibaeenezhad 2016, Zibaeenezhad 2017	Iran	Existing cases (NS)/on OAD	Dyslipidemia?	A1=50?, A2=50?	54.8Ω	47Ω	7.0Ω	156.2Ω	No
Rabiei 2018	Iran	Existing cases (NS)/on OAD		A1=25, A2=25	50.2	37	9.7	200.1	No
Linum usitatissimum									
Barre 2008	Canada ¥	Existing cases (NS)/on OAD?		A1=20, A2=20	60.1Ω	20	7.1Ω	145.0Ω	Yes
Hashemzadeh 2017	Iran	Existing cases (NS)/on OAD?	Coronary heart disease	A1=30, A2=30	59.6	NS	NS	NS	Yes
Momordica charantia									
Dans 2007	Philippine	Both (NS)		A1=20, A2=20	59.2	25	8.0	149.4	Yes
Zänker 2012	Germany	Both (NS)?		A1=44, A2=45	62.5Ω	18Ω	6.5Ω	NS	Yes
Trakoon-osot 2013	Thailand	Existing cases (7.5)/on OAD		A1=19, A2=19	58.0	27	7.4	118.0	No
Rahman 2015	Pakistan	Newly diagnosed/treatment naïve		A1=65✪, A2=30	52.0Ω	31Ω	NS	143.7Ω	Unclear
Suthar 2016a	India¥	Existing cases (NS)/on OAD		A1=64, A2=21	41.3Ω	30Ω	7.9Ω	149.4Ω	Yes
Suthar 2016b	India¥	Both (NS)		A1=83, A2=40	48.6	54	8.2	151.5	Yes
Cortez-Navarrete 2018	Mexico	Newly diagnosed/treatment naïve?		A1=12, A2=12	48.6	16	7.7	138.6	Unclear

Author and year	Country	T2DM patients (duration of T2DM if existing cases [mean, years])	Comorbidities/ complications of T2DM	Participants /eligible arm (n)	Age (mean, years)	Females (n)	HbA1c (mean, %)	FBG (mean, mg/dL)	Any commercial funding/other support
Kumari 2018	India	Existing cases (NS)/on OAD		A1=50✪, A2=25	NS	NS	7.3	152.9	Unclear
Amini 2020¤	Iran	Existing cases (9.3Ω)/on OAD		A1=12, A2=12	51.8Ω	0	NS	190.6Ω	Unclear
Kim 2020	Republic of Korea	Existing cases (NS)/on OAD		A1=66, A2=30	59.2Ω	40Ω	7.0Ω	138.5Ω	Unclear
Nigella sativa									
Najmi 2012	India	Newly diagnosed/treatment naïve	Metabolic syndrome	A1=40, A2=40	NS	38	7.9	154.9	Unclear
Hosseini 2013	Iran	Existing cases (6.2)/on OAD		A1=35, A2=35	49.7	40	8.8	180.0	No
Hadi 2015	Iran	Existing cases (5.3Ω)/on OAD		A1=25, A2=25	53.7Ω	23Ω	NS	172.1Ω	Unclear
Heshmati 2015	Iran	Existing cases (7.0)/on OAD		A1=36, A2=36	46.4	38	8.3	192.6	Unclear
Kaatabi 2015	Saudi Arabia	Existing cases (6.6)/on OAD		A1=57, A2=57	46.5	51	8.4	188.4	No?
Moustafa 2019	Egypt	Newly diagnosed/treatment naïve		A1=29, A2=36?	NS	47	7.5Ω	154.4Ω	Unclear
Kooshki 2020	Iran	Existing cases (7.5)/on OAD		A1=27, A2=23	54.1	34	NS	195.8	Unclear
Jangjo-Borazjani 2021	Iran	Existing cases (NS)/on OAD		A1=20✪, A2=20✪	43.6	40	NS	135.9	Unclear
Plantago ovata									
Ziai 2005	Iran	Existing cases (NS)/on OAD		A1=27, A2=22	52.8Ω	NS	9.8Ω	193.7Ω	Unclear
Feinglos 2013	US¥	Both (NS)		A1=29✪, A2=8	61.0	12	7.5	200.1	Yes
Abutair 2016	Palestine¥	Existing cases (NS)/on OAD		A1=20, A2=20	NS	20	8.5Ω	160.0Ω	No
Portulaca oleracea									
El-Sayed 2011	Yemen	Unclear	Obesity, dyslipidemia?, liver dysfunction?	A1=15, A2=15	40.0	10	NS	226.8	No?
Farzanegi 2014	Iran	Existing cases (NS)/on OAD		A1=14✪, A2=14✪	51.1	28	NS	174.1	Unclear
Dehghan 2016	Iran	Existing cases (8.0?)/on OAD		A1=16?✪, A2=16?✪	55.6	32?	NS	167.0	No
Wainstein 2016	Israel	Existing cases (NS)/on OAD?		A1=31, A2=32	55.4	22	7.5	174.0	Yes
Pterocarpus marsupium									

Author and year	Country	T2DM patients (duration of T2DM if existing cases [mean, years])	Comorbidities/ complications of T2DM	Participants /eligible arm (n)	Age (mean, years)	Females (n)	HbA1c (mean, %)	FBG (mean, mg/dL)	Any commercial funding/other support
Hariharan 2005	India¥	Newly diagnosed/treatment naïve		A1=182, A2=183	46.0Ω	150Ω	10.5Ω	169.2Ω	No
Punica granatum									
Faghihimani 2016	Iran	Existing cases (7.7Ω)/on OAD		A1=40, A2=40	50.0Ω	52Ω	6.8Ω	152.5Ω	Unclear
Babaeian 2013¤	Iran	Existing cases (4.2Ω)/on OAD		A1=25, A2=25	47.0Ω	23Ω	8.0Ω	174.6Ω	Unclear
Sohrab 2014, Sohrab 2015	Iran	Existing cases (6.2Ω)/on OAD		A1=25, A2=25	56.0Ω	21Ω	8.0Ω	154.5Ω	Unclear
Khajebishak 2019a, Khajebishak 2019b	Iran¥	Existing cases (5.9Ω)/on OAD	Obesity	A1=30, A2=30	44.8Ω	30Ω	7.6Ω	159.0Ω	Unclear
Grabež 2020	Republic of Srpska, Bosnia and Herzegovina	Existing cases (6.2)/on OAD		A1=19, A2=18	57.3	20	7.5	159.2	No
Hashemi 2020	Iran	Existing cases (5.7Ω)/on OAD		A1=33, A2=32	62.1Ω	31	7.2Ω	145.7Ω	No
Sesamum indicum									
Shahi 2017	Iran	Existing cases (NS)/on OAD		A1=24, A2=24	50.9Ω	NS	8.0Ω	159.0Ω	No
Aslam 2018	Pakistan	Existing cases (NS)/on OAD		A1=30?, A2=30?	NS	NS	7.6Ω	187.1Ω	No
**Shilajit**									
Narasimha Raju 2016	India	Both (NS)?		A1=56, A2=54	NS	NS	7.6Ω	193.4Ω	Unclear
Niranjan 2016	India	Existing cases (NS)/on OAD		A1=20, A2=20	55.3	10	7.7	NS	Yes
Syzygium cumini									
Sahana 2010	India	Newly diagnosed/treatment naïve		A1=15, A2=5, A3=10	56.2	14	8.3	150.8	Yes
Sidana 2016, Sidana 2017	India	Existing cases (NS)/on OAD		A1=58, A2=55	NS	NS	8.8Ω	142.0Ω	No
Terminalia chebula									
Usharani 2020	India	Existing cases (NS)/on OAD	Endothelial dysfunction	A1=40✪, A2=20	54.3	21	7.4	121.5	Yes
Tinospora cordifolia									
Mishra 2015	India	Existing cases (NS)/on OAD		A1=50?, A2=50?	49.9	NS	8.2	149.8	No

Author and year	Country	T2DM patients (duration of T2DM if existing cases [mean, years])	Comorbidities/ complications of T2DM	Participants /eligible arm (n)	Age (mean, years)	Females (n)	HbA1c (mean, %)	FBG (mean, mg/dL)	Any commercial funding/other support
Roy 2015	India	Existing cases (NS)/on OAD	Dyslipidemia	A1=30?, A2=30	NS	NS	7.8Ω	NS	No
Tribulus terrestris									
Samani 2016	Iran¥	Existing cases (8.2Ω)/on OAD		A1=50, A2=50	52.5Ω	100	7.7Ω	158.0Ω	No
Trigonella foenum-graecum									
Gupta 2001	India	Both (NS)?		A1=12, A2=13	51.0	6	8.3	142.9	Yes
Lu 2008	China	Existing cases (10.8)/on OAD		A1=46, A2=23	54.3	31	8.3	153.0	Unclear
Ansari 2011	Pakistan	Existing cases (NS)/on OAD		A1=102, A2=108	61.0Ω	82Ω	9.2Ω?	NS	Unclear
Rafraf 2014	Iran	Existing cases (5.9Ω)/on OAD		A1=45, A2=45	40.5Ω	78Ω	8.8Ω	179.5Ω	No
Suchitra 2015	India	Existing cases (7.1)/on OAD		A1=30, A2=30	50.2	23	7.9	NS	Unclear
Kaur 2016	India	Newly diagnosed/treatment naïve		A1=30, A2=30	53.1	24	8.1	163.1	No
Singh 2016	India	Both (NS)		A1=20, A2=20, A3=20	56.5	31	8.0	170.2	No
Verma 2016	India¥	Existing cases (NS)/on OAD		A1=77?, A2=77?	NS	46	NS	152.1	Yes
Ranade 2017	India	Existing cases (NS)/on OAD and/or insulin		A1=30, A2=30	47.1	15	7.6	157.2	No
Gholaman 2018	Iran	Existing cases (NS)/on OAD	Obesity	A1=10, A2=10	NS	20	NS	207.7	Unclear
Kandhare 2018	India¥	Existing cases (NS)/on OAD		A1=60, A2=59	51.5	66	8.5	180.2	Yes
Hassani 2019	Iran¥	Existing cases (6.6Ω)/on OAD		A1=72✪, A2=72✪	51.4Ω	71Ω	8.1Ω	160.5Ω	Unclear
Hota 2019$	India	Existing cases (NS)/on OAD		A1=102, A2=102	52.6	65	8.1	144.8	Yes
Najdi 2019	Saudi Arabia	Existing cases (4.8)/on OAD		A1=6, A2=6	51.2	NS	8.2Ω	172.3Ω	Unclear
Rashid 2019	Pakistan	Newly diagnosed/treatment naïve		A1=32, A2=32	47.1	29	7.3	135.0	Unclear
Hadi 2020	Iran	Existing cases (4.7Ω)/on OAD		A1=25, A2=25	47.6Ω	25Ω	NS	141.7Ω	No
Urtica dioica									
Namazi 2011, Esfanjani 2012a¤,	Iran	Existing cases (8.5?Ω)/on OAD		A1=25, A2=25	53.5Ω	21?Ω	7.4Ω	135.5Ω	Unclear

Author and year	Country	T2DM patients (duration of T2DM if existing cases [mean, years])	Comorbidities/ complications of T2DM	Participants /eligible arm (n)	Age (mean, years)	Females (n)	HbA1c (mean, %)	FBG (mean, mg/dL)	Any commercial funding/other support
Esfanjani 2012b¤									
Kianbakht 2013	Iran	Existing cases (12.8Ω)/on OAD		A1=52, A2=55	55.3Ω	75Ω	10.1Ω	255.3Ω	No
Khajeh-Mehrizi 2014	Iran	Existing cases (NS)/on OAD		A1=30, A2=30	54.7Ω	28Ω	NS	175.4Ω	Unclear
Dabagh 2016	Iran	Existing cases (3.0)/on OAD		A1=20✪, A2=20✪	42.9	0	NS	147.5	No
Hassani 2016¤	Iran	Existing cases (NS)/on OAD		A1=15?, A2=13?	NS	28?	NS	177.9Ω	Unclear
Dadvar 2017¤	Iran	Existing cases (2.9)/on OAD		A1=20✪, A2=20✪	45.6	40	NS	156.0	Unclear
Ghalavand 2017	Iran	Existing cases (2.7)/on OAD		A1=20✪, A2=20✪	42.1	0	NS	155.2	Unclear
Korani 2017	Iran	Existing cases (2.5Ω)/on OAD		A1=30, A2=30	49.1Ω	36Ω	8.8Ω	142.5Ω	Yes
Mohammadnia 2017¤	Iran	Existing cases (NS)/on OAD		A1=15, A2=13	55.8Ω	28	NS	NS	Unclear
Vernonia cinerea									
Sayeed 2013	Bangladesh	Existing cases (4.9)/on OAD		A1=24, A2=24	54.2	26	10.1	188.5	Unclear
Withania coagulans									
Hemalatha 2018	India	Existing cases (NS)/on OAD		A1=12, A2=12	NS	15	NS	185.0	Unclear
Withania somnifera									
Usharani 2014	India	Existing cases (NS)/on OAD		A1=44?✪, A2=22?	56.7Ω	21Ω	NS	NS	Yes
Zingiber officinale									
Mahluji 2013	Iran	Existing cases (NS)/on OAD		A1=32, A2=32	51.2Ω	24Ω	7.0Ω	147.5Ω	No
Arablou 2014a, Arablou 2014b¤	Iran	Existing cases (3.9Ω)/on OAD		A1=35, A2=35	52.3Ω	48Ω	8.3Ω	130.0Ω	No
Mozaffari-Khosravi 2014, Talaei 2017, Talaei 2018	Iran	Existing cases (NS)/on OAD		A1=44, A2=44	50.4Ω	50Ω	7.6Ω	153.7Ω	No?
Shidfar 2015	Iran	Existing cases (5.3Ω)/on OAD		A1=25, A2=25	46.2Ω	NS	7.4Ω	158.5Ω	No?

Author and year	Country	T2DM patients (duration of T2DM if existing cases [mean, years])	Comorbidities/ complications of T2DM	Participants /eligible arm (n)	Age (mean, years)	Female s (n)	HbA1c (mean, %)	FBG (mean, mg/dL)	Any commercial funding/other support
Arzati 2017, Zarezadeh 2018	Iran	Existing cases (NS)/on OAD		A1=25?, A2=25?	50.7Ω	34Ω	7.4Ω	165.5Ω	No
Mohammadi 2017¤, Mohammadi 2019¤	Iran	Existing cases (NS)/on OAD		A1=22✪, A2=22✪	53.4	NS	8.9	NS	Unclear
Carvalho 2020	Brazil¥	Existing cases (NS)/on OAD		A1=72, A2=72	58.6Ω	72Ω	8.4Ω	194.4Ω	Unclear
Gholinezhad 2020	Iran¥	Existing cases (NS)/on OAD	Periodontitis	A1=25, A2=25	52.2Ω	23Ω	8.5Ω	180.0Ω	No
Ziziphus mauritiana									
Yazdanpanah 2017	Iran	Existing cases (3.6Ω)/on OAD		A1=58, A2=58	49.8Ω	69Ω	8.0Ω	148.5Ω	No
Acalypha indica/Allium cepa/Allium sativum/Azadirachta indica/Mangifera indica/Murraya koenigii/Musa sapientum/Ocimum tenuiflorum/Phyllanthus amarus/Tinospora cordifolia									
Balasubramaniam 2010	India	Newly diagnosed/treatment naïve		A1=20/group (total 200), A2=20/group (total 200)	50.8	184	NS	NS	Unclear
Allium sativum*; *Cuminum cyminum									
Mansouri 2018	Iran	Existing cases (NS)/on OAD	Hypertension	A1=25, A2=25, A3=25	52.9	41?	11.8	NS	Unclear
Aloe vera*; *Pterocarpus marsupium*; *Aloe vera+Pterocarpus marsupium									
Maurya 2017	India	Newly diagnosed/treatment naïve		A1=35, A2=35, A3=35, A4=35	51.7Ω	64Ω	7.0Ω	154.7Ω	No
Cinnamomum verum*; *Crocus sativus*; *Elettaria cardamomum*; *Zingiber officinale									
Azimi 2014, Azimi 2016	Iran	Existing cases (NS)/on OAD		A1=42?, A2=42?, A3=42?, A4=42?, A5=40?	54.3Ω	125Ω	7.8Ω	360.2Ω	No
***Enicostemma axillare*; Shilajit**									
Kumar 2014	India	Both (NS)		A1=33?, A2=31?, A3=31?	NS	NS	8.1Ω	143.0Ω	Unclear
Enicostemma axillare+Emblica officinalis+Tinospora cordifolia									
Sharma 2019	India¥	Existing cases (NS)/on OAD		A1=6071, A2=43	NS	NS	NS	146.2Ω	No?
Linum usitatissimum*; *Plantago ovata									

Author and year	Country	T2DM patients (duration of T2DM if existing cases [mean, years])	Comorbidities/ complications of T2DM	Participants /eligible arm (n)	Age (mean, years)	Females (n)	HbA1c (mean, %)	FBG (mean, mg/dL)	Any commercial funding/other support
Ricklefs-Johnson 2017	US	Existing cases (NS)/on OAD		A1=10?, A2=9?	59.1Ω	8Ω	6.9Ω	125.1Ω	Yes
Syzygium cumini+Withania coagulans									
Siddiqui 2017	India	Unclear		A1=35?, A2=35?	NS	37Ω	8.5Ω	162.3Ω	Unclear
Trigonella foenum-graecum*; *Aegle marmelos*; *Trigonella foenum-graecum+Aegle marmelos									
Yaheya 2009	Oman?	Existing cases (NS)/on OAD		A1=20, A2=20, A3=20, A4=20	NS	NS	NS	NS	Unclear
**Ayurvedic medicine- combination of plant- and/or mineral-origin ingredients§**									
**AYUBES**									
Godatwar 2019$	India¥	Existing cases (NS)/on OAD		A1=39?, A2=39?	56.5Ω	NS	7.6Ω	160.1Ω	Yes
**BGR-34**									
Gupta 2018	India	Both (NS)?		A1=32, A2=32	48.8Ω	26Ω	9.7Ω	191.6Ω	Unclear
**Bilvadi Churna; Kiratadi Churna**									
Kumari 2016	India	Existing cases (NS)/on OAD	Stress	A1=20, A2=20, A3=20	NS	21	8.0	183.3	Unclear
**CardiPro**									
Fatima 2012	India	Existing cases (NS)/on OAD		A1=30, A2=30	52.2Ω	21Ω	NS	NS	Yes
**Cogent db**									
Shekhar 2002	Malaysia ¥	Existing cases (9.0)/on OAD		A1=39, A2=40	49.0Ω	27	9.9Ω	208.8Ω	Yes
**DCBT 2345**									
Mohan 2001	India	Existing cases (NS)/on OAD		A1=22?, A2=22?	55.0Ω	19Ω	9.4Ω	189.0Ω	Yes
**Diabetea tea**									
Mahmoud 2016	Kuwait	Existing cases (5.7Ω)/on OAD	Hypertension?	A1=30, A2=30	52.9Ω	22Ω	9.1Ω	156.6Ω	Unclear
Emblica officinalis*; *Withania somnifera*; *Emblica officinalis, Withania somnifera									
Usharani 2014	India	Existing cases (NS)/on OAD		A1=14?, A2=14?, A3=14?	58.7Ω	4Ω	NS	NS	Yes
**Herbal combination**									
Shokoohi 2017	Iran	Existing cases (6.2)/on OAD	Dyslipidemia	A1=43, A2=43	49.7	86	8.0	189.8	No
**Hyponidd**									
Poongothai 2002	India	Existing cases (NS)/on OAD		A1=20, A2=20	53.5Ω	11Ω	8.7Ω	177.5Ω	Yes
**Inolter**									

Author and year	Country	T2DM patients (duration of T2DM if existing cases [mean, years])	Comorbidities/ complications of T2DM	Participants /eligible arm (n)	Age (mean, years)	Females (n)	HbA1c (mean, %)	FBG (mean, mg/dL)	Any commercial funding/other support
Agrawal 2002	India	Newly diagnosed/treatment naïve		A1=30, A2=30	46.1	14	10.1	218.5	Yes
**Kalpit**									
Agarwal 2013	India	Unclear	Obesity	A1=30, A2=30, A3=30	NS	NS	NS	168.4	Unclear
**Khadira-Kramuka Kashaya Ghanavati; Nishamalaki, Shilajit; Khadira-Kramuka Kashaya Ghanavati+Nishamalaki, Shilajit**									
Paliwal 2018$	India	Both (NS)		A1=20?, A2=20?, A3=20?	NS	NS	8.0	160.3	Unclear
**Lodhradi Kashaya Ghana Vati**									
Bramhankar 2017	India	Newly diagnosed/treatment naïve?		A1=15, A2=15, A3=15	NS	NS	NS	176.8Ω	Unclear
**Madhumeha Nashini Gutika; Darvyadi Kwatha; Madhumeha Nashini Gutika+Darvyadi Kwatha**									
Bhawana 2015	India	Unclear		A1=20, A2=20, A3=20	NS	NS	NS	159.7	Unclear
**Mamajjaka Ghana Vati; Tejashiladi Vati**									
Bhagat 2017$	India	Both (NS)		A1=46, A2=44	NS	44	NS	209.7Ω	Unclear
**Mamajjaka Ghana Vati; Trikatu Gutika**									
Kataria 2017$	India	Both (NS)		A1=15, A2=15	NS	12	NS	170.6	Unclear
**Mehagni**									
Gopalakrishna 2017	India	Existing cases (NS)/on OAD		A1=20, A2=20	43.4	NS	9.4	156.3	Yes
**Mustadi Kwatha Ghana Vati**									
Kushwaha 2017	India	Existing cases (8.9)/on OAD		A1=50, A2=50	NS	50	NS	180.8	Unclear
**Naga Bhasma, Nishamalaki; Nishamalaki, *Hordeum vulgare***									
Desale 2018$	India	Both (NS)		A1=80✪, A2=40	NS	56	NS	179.4Ω	Unclear
Nigella sativa*, *Trigonella foenum-graecum									
Memon 2010a, Memon 2010b, Memon 2012	Pakistan ¥	Existing cases (NS)/on OAD		A1=50, A2=50	49.5	60	NS	189.0	Unclear
**Nisha Katakadi Kashaya; Yashad Bhasma; Nisha Katakadi Kashaya+Yashad Bhasma**									
Srinivas 2018	India¥	Both (NS)?		A1=65?, A2=64?, A3=64?	NS	100Ω	7.1Ω	145.0Ω	Unclear
**Pancreas tonic**									

Author and year	Country	T2DM patients (duration of T2DM if existing cases [mean, years])	Comorbidities/ complications of T2DM	Participants /eligible arm (n)	Age (mean, years)	Females (n)	HbA1c (mean, %)	FBG (mean, mg/dL)	Any commercial funding/other support
Hsia 2004	US¥	Existing cases (4.1Ω~)/on OAD		A1=31, A2=16	47.5Ω~	26Ω~	9.9Ω~	219.0Ω~	Unclear
**Polyherbal formulation**									
Awasthi 2015	India	Newly diagnosed/treatment naïve		A1=48, A2=45	45.6Ω	33Ω	7.6Ω	163.3Ω	No?
**Salasaradi Kashaya+Shilajit+Trivanga Bhasma+*Tinospora cordifolia*, *Azadirachta indica*; Hyponidd**									
Bhat 2012$	India	Both (NS)		A1=25?, A2=25?	NS	8Ω	NS	137.8Ω	Yes?
**Shilajit; Asanadi Ghana Vati**									
Gupta 2016	India	Existing cases (NS)/on OAD	Diabetic neuropathy?	A1=45?, A2=45?	NS	29Ω	7.7Ω	168.5Ω	No
**Swarnamakshika Bhasma**									
Taviad 2016$	India	Both (NS)		A1=54, A2=56	NS	65	NS	180.2Ω	Unclear
**Talapotaka Churna**									
Nille 2018	India	Unclear		A1=16?✪, A2=12?, A3=8?	NS	NS	8.5Ω	150.7Ω	Unclear
Trigonella foenum-graecum*, *Ocimum tenuiflorum									
Mitra 2006	India	Newly diagnosed/treatment naïve	Dyslipidemia	A1=64✪, A2=16	46.7	38	NS	183.6	Unclear
***Triticum aestivum*; Nishamalaki; *Triticum aestivum*+Nishamalaki**									
Samagandi 2012	India	Existing cases (NS)/on OAD		A1=10, A2=10, A3=10	NS	NS	NS	172.6	Unclear
**Vidangadi Yoga**									
Deshpande 2018	India	Both (NS)		A1=31, A2=30	49.2Ω	32Ω	7.0Ω	149.4Ω	No
**Vijaysaradi Ghana Vati; Madhumehari Vati**									
Sharma 2018$	India	Existing cases (NS)/on OAD		A1=29, A2=31	NS	19	8.1Ω	164.3Ω	Yes

A1, A2, A3, A4, A5=Eligible study arms, FBG=Fasting blood glucose, HbA1c=Glycated hemoglobin, NS=Not specified, OAD=Oral antidiabetic drug, T2DM=Type 2 diabetes mellitus
$Not a journal publication.
¤In Persian language.
¥Multicenter/site recruitment.
✪Two or more study arms were pooled together.
▼Median.
ΩPer-protocol analysis.
~Prior to randomization (and not at the point of randomization).
§Some of the Ayurvedic medicines contain single plant- or mineral-origin ingredient.

Appendix S5: Details of the interventions.

Author and year	Arm 1	Arm 2	Arm 3	Arm 4	Arm 5	Form and timing of administration of Ayurvedic medicine	Dose (g/day or otherwise mentioned) X duration (weeks) of Ayurvedic medicine	Anupan of Ayurvedic medicine
**Ayurvedic medicine- single plant- or mineral-origin ingredient**								
Abelmoschus esculentus								
Moradi 2020	*Abelmoschus esculentus* (fruit)	Placebo				A1=Powder, BD	A1=10 X 8	A1=Yogurt
Acacia Senegal								
Babiker 2017, Babiker 2018	*Acacia senegal* (gum)	Placebo				A1=Powder, OD	A1=30 X 13	A1=Water
Aegle marmelos								
Sankhla 2009	*Aegle marmelos* (leaf, leaf extract)	Placebo (green gram)				A1=Powder, BD	A1=4 X 8	
Sharma 2013	*Aegle marmelos* (leaf extract)✪	Placebo				A1=Capsule, OD✪	A1=0.25, 0.6 X 13✪	
Nigam 2019	*Aegle marmelos* (leaf)	No additional medicine?				A1=Juice, OD	A1=100(ml) X 8	
Allium sativum								
Ashraf 2005	*Allium sativum* (clove)	Placebo				A1=Tablet, BD	A1=0.6 X 12	
Ashraf 2011a	*Allium sativum* (clove)✪	OAD	Placebo			A1=Tablet, Unclear✪	A1=0.3, 0.6, 0.9, 1.2, 1.5 X 24✪	
Ashraf 2011b	*Allium sativum* (clove)	Placebo				A1=Tablet, TDS	A1=0.9 X 24	
Kumar 2013	*Allium sativum* (clove)	No additional medicine				A1=Capsule, BD	A1=0.5 X 12	
Aloe vera								
Arora 2009	*Aloe vera* (leaf?)	No additional medicine				A1=Juice, OD	A1=150(ml) X 13	
Huseini 2012a, Huseini 2012b	*Aloe vera* (leaf gel)	Placebo				A1=Capsule, BD	A1=0.6 X 9	

Author and year	Arm 1	Arm 2	Arm 3	Arm 4	Arm 5	Form and timing of administration of Ayurvedic medicine	Dose (g/day or otherwise mentioned) X duration (weeks) of Ayurvedic medicine	Anupan of Ayurvedic medicine
Zarrintan 2015	*Aloe vera* (leaf? extract)	Placebo				A1=Tablet, OD	A1=1 X 9	
Anethum graveolens								
Mobasseri 2014	*Anethum graveolens* (leaf, stem)	Placebo				A1=Tablet, TDS	A1=3.3 X 8	
Haidari 2020	*Anethum graveolens* (leaf)	Placebo				A1=Capsule, TDS	A1=3 X 8	
Azadirachta indica								
Usharani 2020	*Azadirachta indica* (leaf+twig extract)✪	Placebo				A1=Capsule, BD✪	A1=0.25, 0.5, 1 X 12✪	A1=Water✪
Berberis aristata								
Sharma 2017	*Berberis aristata* (stem)✪	No additional medicine				A1=Powder, BD✪	A1=1.5, 3 X 39✪	
Boswellia serrata								
Azadmehr 2014	*Boswellia serrata* (gum resin)	Placebo				A1=Capsule, BD	A1=0.8 X 12	
Mehrzadi 2018	*Boswellia serrata* (gum resin)	Placebo				A1=Capsule, BD	A1=0.5 X 8	
Camellia sinensis								
MacKenzie 2007	*Camellia sinensis* (green and black tea; leaf extract; decaffeinated)✪	Placebo✪				A1=Capsule, OD✪	A1=0.375, 0.75 X 12✪	
Mirzaei 2009	*Camellia sinensis* (green tea; leaf extract)¶	Placebo				A1=Capsule, TDS	A1=1.5 X 8	
Hsu 2011	*Camellia sinensis* (green tea; leaf extract; decaffeinated)	Placebo				A1=Capsule, TDS	A1=1.5 X 16	
Lasaite 2014	*Camellia sinensis* (green tea; leaf extract)	Placebo				A1=Capsule, BD to TDS	A1=0.4 to 0.6 X 78	
Liu 2014	*Camellia sinensis* (green tea; leaf extract; decaffeinated)	Placebo				A1=Capsule, TDS	A1=1.5 X 16	
Quezada-Fernandez 2019	*Camellia sinensis* (green tea; leaf extract; decaffeinated)	Placebo				A1=Capsule, Unclear	A1=0.4 X 12	
Capparis spinosa								
Huseini 2013	*Capparis spinosa* (fruit extract)¶	Placebo				A1=Capsule, TDS	A1=1.2 X 9	

Author and year	Arm 1	Arm 2	Arm 3	Arm 4	Arm 5	Form and timing of administration of Ayurvedic medicine	Dose (g/day or otherwise mentioned) X duration (weeks) of Ayurvedic medicine	Anupan of Ayurvedic medicine
Cichorium intybus								
Chandra 2020	*Cichorium intybus* (seed extract)¶	Placebo				A1=Capsule, BD	A1=0.541 X 12	
Cinnamomum aromaticum								
Mang 2006	*Cinnamomum aromaticum* (bark extract)	Placebo				A1=Capsule, TDS	A1=0.336 X 17	
Suppapitiporn 2006	*Cinnamomum aromaticum* (bark)	Placebo				A1=Capsule, TDS	A1=4.5? X 12	
Blevins 2007	*Cinnamomum aromaticum* (bark)	Placebo (wheat)				A1=Capsule, BD	A1=1 X 13	
Crawford 2009	*Cinnamomum aromaticum* (bark)	No additional medicine				A1=Capsule, OD?	A1=1 X 13	
Akilen 2010	*Cinnamomum aromaticum* (bark)¶	Placebo				A1=Capsule, TDS	A1=2 X 12	
Wainstein 2011	*Cinnamomum aromaticum* (bark)	Placebo				A1=Capsule, TDS	A1=2.4 to 1.2 X 12	
Lu 2012	*Cinnamomum aromaticum* (bark extract)¶✪	Placebo				A1=Tablet, OD✪	A1=0.12, 0.36 X 13✪	
Sharma 2012	*Cinnamomum aromaticum*? (bark)✪	Placebo				A1=Capsule, TDS✪	A1=3, 6 X 13✪	
Hasanzade 2013	*Cinnamomum aromaticum* (bark)	Placebo				A1=Capsule, BD	A1=1 X 9	
Tangvarasittichai 2015, Sengsuk 2016	*Cinnamomum aromaticum* (bark)	Placebo				A1=Capsule, TDS	A1=1.5 X 9	
Cinnamomum verum								
Vafa 2012	*Cinnamomum verum* (bark)	Placebo (wheat)				A1=Capsule, TDS	A1=3 X 8	
Zahmatkesh 2012	*Cinnamomum verum* (bark)	Placebo				A1=Capsule, BD	A1=1 X 8?	
Talaei 2017	*Cinnamomum verum*? (bark)	Placebo				A1=Capsule, TDS	A1=3 X 8	
Zare 2019	*Cinnamomum verum*? (bark)¶✪	Placebo✪				A1=Capsule, BD✪	A1=1 X 13✪	

Author and year	Arm 1	Arm 2	Arm 3	Arm 4	Arm 5	Form and timing of administration of Ayurvedic medicine	Dose (g/day or otherwise mentioned) X duration (weeks) of Ayurvedic medicine	Anupan of Ayurvedic medicine
Mirmiranpour 2020	*Cinnamomum verum* (bark)	Placebo (rice)				A1=Capsule, OD	A1=0.5 X 13	
Citrullus colocynthis								
Huseini 2009	*Citrullus colocynthis* (fruit)	Placebo				A1=Capsule, TDS	A1=0.3 X 9	
Barghamdi 2016	*Citrullus colocynthis* (fruit)	Placebo				A1=Capsule, OD	A1=0.125 X 9	
Coccinia grandis								
Kuriyan 2008, Kurpad 2008	*Coccinia grandis* (aerial parts extract)	Placebo				A1=Capsule, OD?	A1=1 X 13	
Quamri 2017	*Coccinia grandis* (leaf)	OAD				A1=Powder, BD	A1=15 X 8	
Wasana 2021	*Coccinia grandis* (leaf extract)¶	Placebo (corn)				A1=Capsule, OD	A1=0.5 X 13	
Convolvulus prostratus								
Patel 2012	*Convolvulus prostratus* (whole plant)	No additional medicine				A1=Capsule, TDS	A1=1.5 X 8	
Crocus sativus								
Milajerdi 2018	*Crocus sativus* (stigma extract)	Placebo				A1=Capsule, BD	A1=0.03 X 8	
Aleali 2019	*Crocus sativus* (stigma extract)	Placebo				A1=Capsule, Unclear	A1=0.03 X 12	
Ebrahimi 2019	*Crocus sativus* (stigma)	Placebo				A1=Tablet, BD	A1=0.1 X 12	A1=Water
Mobasseri 2020	*Crocus sativus* (stigma)	Placebo				A1=Capsule, OD	A1=0.1 X 8	
Cuminum cyminum								
Jafari 2017	*Cuminum cyminum* (seed oil)✪	Placebo				A1=Capsule, Unclear✪	A1=0.05, 0.1 X 8✪	
Hendre 2020	*Cuminum cyminum* (seed)	No additional medicine				A1=Capsule, OD	A1=0.5 X 13	
Curcuma longa								
Usharani 2008	*Curcuma longa* (rhizome extract)	Placebo				A1=Capsule, BD	A1=0.6 X 8	

Author and year	Arm 1	Arm 2	Arm 3	Arm 4	Arm 5	Form and timing of administration of Ayurvedic medicine	Dose (g/day or otherwise mentioned) X duration (weeks) of Ayurvedic medicine	Anupan of Ayurvedic medicine
Na 2013	*Curcuma longa* (rhizome extract)	Placebo				A1=Capsule, BD	A1=0.3 X 13	
Chuengsamarn 2014	*Curcuma longa* (rhizome extract)	Placebo				A1=Capsule, BD	A1=1.5 X 26	
Panahi 2017, Panahi 2018	*Curcuma longa* (rhizome extract)	Placebo				A1=Capsule, Unclear	A1=1 X 12	
Adab 2019	*Curcuma longa* (rhizome)¶	Placebo (corn)				A1=Capsule, TDS	A1=2.1 X 8	
Adibian 2019	*Curcuma longa* (rhizome extract)	Placebo (rice)				A1=Capsule, TDS	A1=1.5 X 10	
Srinivasan 2019	*Curcuma longa* (rhizome)	Placebo				A1=Capsule, TDS	A1=1.2 X 12	
de Sousa 2020	*Curcuma longa* (rhizome)	Placebo				A1=Capsule, OD	A1=0.5 X 17	
Cyamopsis tetragonoloba								
Uusitupa 1984	*Cyamopsis tetragonoloba* (seed)	Placebo (wheat)				A1=Granule, TDS	A1=7.5 to 15 to 21 X 18	
Uusitupa 1989	*Cyamopsis tetragonoloba* (seed)	Placebo (wheat)				A1=Granule, TDS	A1=15 X 13	A1=Juice, milk, or water
Eclipta prostrata								
Sazia 2015a, Sazia 2015b	*Eclipta prostrata* (extract)	OAD	No medicine			A1=Capsule, TDS	A1=2.4 X 13	
Elettaria cardamomum								
Aghasi 2019	*Elettaria cardamomum* (seedpod?)¶	Placebo				A1=Capsule, TDS	A1=3 X 10	
Emblica officinalis								
Usharani 2013	*Emblica officinalis* (fruit extract)✪	Placebo				A1=Capsule, BD✪	A1=0.5, 1 X 12✪	
Enicostemma axillare								
Shankarrao 2017	*Enicostemma axillare*	OAD				A1=Powder, BD	A1=10 X 12	A1=Lukewarm water
Gynostemma pentaphyllum								

Author and year	Arm 1	Arm 2	Arm 3	Arm 4	Arm 5	Form and timing of administration of Ayurvedic medicine	Dose (g/day or otherwise mentioned) X duration (weeks) of Ayurvedic medicine	Anupan of Ayurvedic medicine
Huyen 2010	*Gynostemma pentaphyllum* (whole plant extract)¶	Placebo (green tea)				A1=Powder, BD	A1=6 X 12	A1=Water
Huyen 2012	*Gynostemma pentaphyllum* (whole plant extract)¶	Placebo (green tea)				A1=Powder, BD	A1=6 X 8	A1=Water
Hibiscus sabdariffa								
Sarbini 2019	*Hibiscus sabdariffa*¶	Placebo				A1=Capsule, BD	A1=1 X 8	
Ipomoea batatas								
Ludvik 2004	*Ipomoea batatas* (white variety; tuber skin extract)	Placebo				A1=Tablet?, OD	A1=4 X 12	
Ludvik 2008	*Ipomoea batatas* (white variety; tuber skin extract)	Placebo				A1=Tablet, OD	A1=4 X 22	
Juglans regia								
Hosseini 2014a	*Juglans regia* (leaf extract)	Placebo				A1=Tablet, BD	A1=0.4 X 9	
Hosseini 2014b	*Juglans regia* (leaf extract)¶	Placebo				A1=Capsule, BD	A1=0.2 X 13	
Abdoli 2017	*Juglans regia* (leaf extract)¶	Placebo				A1=Capsule, TDS	A1=0.75 X 13	
Zibaeenezhad 2016, Zibaeenezhad 2017	*Juglans regia* (seed oil)	Placebo?				A1=Capsule, TDS	A1=15 X 13	
Rabiei 2018	*Juglans regia* (leaf extract)¶	Placebo				A1=Capsule, OD to BD	A1=0.1 to 0.2 X 8	
Linum usitatissimum								
Barre 2008	*Linum usitatissimum* (seed oil)	Placebo (safflower oil)				A1=Capsule, Unclear	A1=10 X 13	
Hashemzadeh 2017	*Linum usitatissimum* (seed oil)	Placebo				A1=Capsule, BD	A1=2 X 12	
Momordica charantia								
Dans 2007	*Momordica charantia* (fruit+seed extract)	Placebo				A1=Capsule, TDS	A1=3? X 13	
Zänker 2012	*Momordica charantia* (fruit? extract)	Placebo				A1=Capsule, BD	A1=1 X 17	
Trakoon-osot 2013	*Momordica charantia* (fruit pulp)¶	Placebo				A1=Capsule, TDS	A1=6 X 16	
Rahman 2015	*Momordica charantia* (fruit pulp)✪	OAD				A1=Capsule, Unclear✪	A1=2, 4 X 10✪	

Author and year	Arm 1	Arm 2	Arm 3	Arm 4	Arm 5	Form and timing of administration of Ayurvedic medicine	Dose (g/day or otherwise mentioned) X duration (weeks) of Ayurvedic medicine	Anupan of Ayurvedic medicine
Suthar 2016a	*Momordica charantia* (fruit)	Placebo				A1=Capsule, BD	A1=1.2 X 13	
Suthar 2016b	*Momordica charantia* (fruit)	OAD				A1=Capsule, BD	A1=0.8 to 1.2 X 15	
Cortez-Navarrete 2018	*Momordica charantia* (fruit pulp)	Placebo				A1=Capsule, BD	A1=2 X 12	
Kumari 2018	*Momordica charantia* (fruit?)✪	Placebo				A1=Tablet, Unclear✪	A1=1, 1.5 X 8✪	
Amini 2020	*Momordica charantia* (fruit)	Placebo				A1=Capsule, BD	A1=2 X 8	
Kim 2020	*Momordica charantia* (fruit extract)¶	Placebo				A1=Capsule, BD	A1=2.38 X 12	
Nigella sativa								
Najmi 2012	*Nigella sativa* (seed)	No additional medicine				A1=Capsule, OD	A1=0.5 X 8	
Hosseini 2013	*Nigella sativa* (seed oil)	Placebo				A1=Oil, BD	A1=5(ml) X 13	
Hadi 2015	*Nigella sativa* (seed oil)	Placebo				A1=Capsule, BD	A1=1 X 8	
Heshmati 2015	*Nigella sativa* (seed oil)	Placebo (sunflower oil)				A1=Capsule, TDS	A1=3 X 12	
Kaatabi 2015	*Nigella sativa* (seed)	Placebo				A1=Capsule, BD	A1=2 X 52	
Moustafa 2019	*Nigella sativa* (seed oil)	OAD				A1=Capsule, TDS	A1=1.35 X 12	
Kooshki 2020	*Nigella sativa* (seed oil)	Placebo				A1=Capsule, BD	A1=1 X 8	
Jangjo-Borazjani 2021	*Nigella sativa* (seed)✪	Placebo✪				A1=Capsule, BD✪	A1=2 X 8✪	
Plantago ovata								
Ziai 2005	*Plantago ovata* (seed husk)	Placebo				A1=Fiber, BD	A1=10.2 X 8	A1=Water
Feinglos 2013	*Plantago ovata* (seed husk)✪	Placebo				A1=Fiber, BD✪	A1=6.8, 13.6 X 12✪	

Author and year	Arm 1	Arm 2	Arm 3	Arm 4	Arm 5	Form and timing of administration of Ayurvedic medicine	Dose (g/day or otherwise mentioned) X duration (weeks) of Ayurvedic medicine	Anupan of Ayurvedic medicine
Abutair 2016	*Plantago ovata* (seed husk)	No additional medicine?				A1=Fiber, BD	A1=10.5 X 8	A1=Water
Portulaca oleracea								
El-Sayed 2011	*Portulaca oleracea* (seed)¶	OAD				A1=Powder, BD	A1=10 X 8	A1=Skimmed yogurt
Farzanegi 2014	*Portulaca oleracea* (seed)✪	Placebo✪				A1=Powder, BD✪	A1=7.5 X 8✪	A1=Skimmed yogurt✪
Dehghan 2016	*Portulaca oleracea* (seed)¶✪	Placebo✪				A1=Capsule, BD✪	A1=7.5 X 16✪	
Wainstein 2016	*Portulaca oleracea* (whole plant extract)	Placebo				A1=Capsule, OD?	A1=0.18 X 12	
Pterocarpus marsupium								
Hariharan 2005	*Pterocarpus marsupium* (heartwood extract)	OAD				A1=Capsule, BD	A1=2 to 4 X 36	
Punica granatum								
Faghihimani 2016	*Punica granatum* (seed oil)	Placebo				A1=Capsule, BD	A1=2 X 8	
Babaeian 2013	*Punica granatum* (fruit)	Placebo?				A1=Juice, OD?	A1=240(ml) X 8	
Sohrab 2014, Sohrab 2015	*Punica granatum* (fruit)	Placebo				A1=Juice, OD?	A1=250(ml) X 12	
Khajebishak 2019a, Khajebishak 2019b	*Punica granatum* (seed oil)	Placebo				A1=Capsule, TDS	A1=3 X 8	
Grabež 2020	*Punica granatum* (peel extract)	Placebo				A1=Capsule, BD	A1=0.5 X 8	
Hashemi 2020	*Punica granatum* (seed)	Placebo				A1=Infusion, BD	A1=10 X 8	A1=Hot water (infused in)
Sesamum indicum								
Shahi 2017	*Sesamum indicum* (seed extract)	Placebo				A1=Capsule, OD	A1=0.2 X 8	A1=Water
Aslam 2018	*Sesamum indicum* (seed oil)	Placebo				A1=Oil, Unclear	A1=30(ml) X 13	

Author and year	Arm 1	Arm 2	Arm 3	Arm 4	Arm 5	Form and timing of administration of Ayurvedic medicine	Dose (g/day or otherwise mentioned) X duration (weeks) of Ayurvedic medicine	Anupan of Ayurvedic medicine
**Shilajit**								
Narasimha Raju 2016	Shilajit (*Asphaltum punjabianum*)	Placebo				A1=Capsule, BD	A1=2 X 9	A1=Salasar adi Gana Kashaya (*Acacia catechu* [resin], *Areca catechu* [fruit], *Gymnema sylvestre* [leaf], *Albizia lebbeck* [bark], *Terminalia arjuna* [bark], *Pongamia pinnata* [seed])
Niranjan 2016	Shilajit (*Asphaltum punjabianum*; extract)	Placebo				A1=Capsule, BD	A1=0.5 X 12	A1=Water
Syzygium cumini								
Sahana 2010	*Syzygium cumini* (seed)¶	OAD	No medicine			A1=Powder, BD	A1=10 X 26	
Sidana 2016, Sidana 2017	*Syzygium cumini* (seed)	Placebo				A1=Powder, BD	A1=10 X 13	
Terminalia chebula								
Usharani 2020	*Terminalia chebula* (fruit extract)¶✪	Placebo				A1=Capsule, BD✪	A1=0.5, 1 X 12✪	
Tinospora cordifolia								
Mishra 2015	*Tinospora cordifolia* (extract)	No additional medicine				A1=Tablet, TDS	A1=1.5 X 26	
Roy 2015	*Tinospora cordifolia* (stem extract)	No additional medicine				A1=Capsule, BD	A1=0.5 X 9	
Tribulus terrestris								

Author and year	Arm 1	Arm 2	Arm 3	Arm 4	Arm 5	Form and timing of administration of Ayurvedic medicine	Dose (g/day or otherwise mentioned) X duration (weeks) of Ayurvedic medicine	Anupan of Ayurvedic medicine
Samani 2016	*Tribulus terrestris* (extract)¶	Placebo				A1=Capsule, BD	A1=1 X 13	
Trigonella foenum-graecum								
Gupta 2001	*Trigonella foenum-graecum* (seed extract)	Placebo				A1=Capsule, BD	A1=1 X 9	
Lu 2008	*Trigonella foenum-graecum* (seed extract)¶	Placebo				A1=Capsule, TDS	A1=6.3 X 12	
Ansari 2011	*Trigonella foenum-graecum* (seed)	No additional medicine				A1=Powder, BD	A1=100 X 13	
Rafraf 2014	*Trigonella foenum-graecum* (seed)	Placebo (wheat)				A1=Powder, BD	A1=10 X 8	A1=Water
Suchitra 2015	*Trigonella foenum-graecum* (seed)	No additional medicine				A1=Seed, TDS	A1=30 X 8	
Kaur 2016	*Trigonella foenum-graecum* (seed)	No additional medicine				A1=Capsule, TDS	A1=3 X 12	
Singh 2016	*Trigonella foenum-graecum* (seed extract)	*Trigonella foenum-graecum* (seed extract)+OAD	OAD			A1=Capsule, OD, A2=Capsule, BD	A1=0.5 X 12, A2=1 X 12	
Verma 2016	*Trigonella foenum-graecum* (seed extract)¶	Placebo				A1=Capsule, BD	A1=1 X 13	
Ranade 2017	*Trigonella foenum-graecum* (seed)	No additional medicine				A1=Seed, OD	A1=10 X 26	A1=Hot water (soaked in)
Gholaman 2018	*Trigonella foenum-graecum* (seed)	Placebo				A1=Powder, TDS	A1=15 X 8	A1=Yogurt
Kandhare 2018	*Trigonella foenum-graecum* (seed extract)	Placebo				A1=Capsule, TDS	A1=2.1 X 12	
Hassani 2019	*Trigonella foenum-graecum* (seed?)¶✪	Placebo (wheat)✪				A1=Powder, BD✪	A1=10 X 8✪	
Hota 2019	*Trigonella foenum-graecum* (seed extract)¶	No additional medicine				A1=Capsule, BD	A1=1 X 12	
Najdi 2019	*Trigonella foenum-graecum* (seed)	OAD				A1=Capsule, TDS	A1=2 X 12	
Rashid 2019	*Trigonella foenum-graecum* (seed extract)¶	Placebo				A1=Capsule, OD	A1=1 X 12	
Hadi 2020	*Trigonella foenum-graecum* (seed)	No additional medicine				A1=Powder, TDS	A1=15 X 8	A1=Water
Urtica dioica								

Author and year	Arm 1	Arm 2	Arm 3	Arm 4	Arm 5	Form and timing of administration of Ayurvedic medicine	Dose (g/day or otherwise mentioned) X duration (weeks) of Ayurvedic medicine	Anupan of Ayurvedic medicine
Namazi 2011, Esfanjani 2012a, Esfanjani 2012b	*Urtica dioica* (aerial parts extract)	Placebo				A1=Powder, TDS	A1=0.1(/kg) X 8	A1=Lukewarm water
Kianbakht 2013	*Urtica dioica* (leaf extract)¶	Placebo				A1=Capsule, TDS	A1=1.5 X 13	
Khajeh-Mehrizi 2014	*Urtica dioica* (extract)	Placebo				A1=Unclear, TDS	A1=0.1(/kg) X 8	
Dabagh 2016	*Urtica dioica* (leaf)✪	No additional medicine✪				A1=Powder, OD✪	A1=10 X 8✪	A1=Yogurt✪
Hassani 2016	*Urtica dioica* (extract)	Placebo				A1=Liquid, TDS	A1=6(ml) X 8	A1=Water
Dadvar 2017	*Urtica dioica*✪	No additional medicine✪				A1=Powder, OD	A1=10 X 8	A1=Yogurt
Ghalavand 2017	*Urtica dioica*✪	No additional medicine✪				A1=Unclear, TDS✪	A1=10 X 8✪	
Korani 2017	*Urtica dioica* (extract)	Placebo				A1=Unclear, TDS	A1=0.02(/kg) X 8	
Mohammadnia 2017	*Urtica dioica* (extract)	Placebo				A1=Liquid, TDS	A1=6(ml) X 8	A1=Water
Vernonia cinerea								
Sayeed 2013	*Vernonia cinerea* (root)	Placebo				A1=Tablet, TDS	A1=6 X 13	
Withania coagulans								
Hemalatha 2018	*Withania coagulans* (fruit)	Placebo				A1=Infusion, OD	A1=2-2.5 X 9	A1=Water (infused in)
Withania somnifera								
Usharani 2014	*Withania somnifera* (root extract)✪	Placebo				A1=Capsule, BD✪	A1=0.5, 1 X 12✪	
Zingiber officinale								
Mahluji 2013	*Zingiber officinale* (rhizome)	Placebo (corn)				A1=Tablet, BD	A1=2 X 8	
Arablou 2014a,	*Zingiber officinale* (rhizome)	Placebo (wheat)				A1=Capsule, BD	A1=1.6 X 12	

Author and year	Arm 1	Arm 2	Arm 3	Arm 4	Arm 5	Form and timing of administration of Ayurvedic medicine	Dose (g/day or otherwise mentioned) X duration (weeks) of Ayurvedic medicine	Anupan of Ayurvedic medicine
Arablou 2014b								
Mozaffari-Khosravi 2014, Talaei 2017, Talaei 2018	*Zingiber officinale* (rhizome)	Placebo				A1=Capsule, TDS	A1=3 X 8	
Shidfar 2015	*Zingiber officinale* (rhizome)	Placebo				A1=Capsule, TDS?	A1=3 X 12	
Arzati 2017, Zarezadeh 2018	*Zingiber officinale* (rhizome)	Placebo (wheat)				A1=Capsule, BD	A1=2 X 10	
Mohammadi 2017, Mohammadi 2019	*Zingiber officinale* (rhizome)✪	Placebo✪				A1=Capsule, QDS?✪	A1=1 X 8✪	
Carvalho 2020	*Zingiber officinale* (rhizome extract)	Placebo				A1=Capsule, BD	A1=1.2 X 13	
Gholinezhad 2020	*Zingiber officinale* (rhizome)	Placebo (pea)				A1=Tablet, BD	A1=2 X 8	
Ziziphus mauritiana								
Yazdanpanah 2017	*Ziziphus mauritiana* (fruit pulp+peel)	No additional medicine				A1=Infusion, TDS	A1=30 X 12	A1=Hot water
Acalypha indica/Allium cepa/Allium sativum/Azadirachta indica/Mangifera indica/Murraya koenigii/Musa sapientum/Ocimum tenuiflorum/Phyllanthus amarus/Tinospora cordifolia								
Balasubramaniam 2010	*Acalypha indica* (whole plant extract)/*Allium cepa* (bulb extract)/*Allium sativum* (bulb extract)/*Azadirachta indica* (leaf extract)/*Mangifera indica* (stem bark extract)/*Murraya koenigii* (leaf extract)/*Musa sapientum* (flower extract)/*Ocimum tenuiflorum* (leaf extract)/*Phyllanthus amarus* (whole plant extract)/*Tinospora cordifolia* (leaf extract)¶	Placebo				A1=Decoction, OD?	A1=5(ml) X 9	
Allium sativum*; *Cuminum cyminum								
Mansouri 2018	*Allium sativum* (clove)	*Cuminum cyminum* (seed)	Placebo			A1=Capsule, TDS, A2=Capsule, BD	A1=0.9 X 9, A2=0.2 X 9	
Aloe vera*; *Pterocarpus marsupium*; *Aloe vera+Pterocarpus marsupium								
Maurya 2017	*Aloe vera* (leaf?)	*Pterocarpus marsupium* (heartwood?)	*Aloe vera+Pter*	OAD		A1=Capsule, BD,	A1=0.6 X 13, A2=2	

Author and year	Arm 1	Arm 2	Arm 3	Arm 4	Arm 5	Form and timing of administration of Ayurvedic medicine	Dose (g/day or otherwise mentioned) X duration (weeks) of Ayurvedic medicine	Anupan of Ayurvedic medicine
			ocarpus marsupium			A2=Capsule, BD, A3=Capsule, OD+Capsule, BD	to 4 X 13, A3=0.3+1 to 3 X 13	
Cinnamomum verum*; *Crocus sativus*; *Elettaria cardamomum*; *Zingiber officinale								
Azimi 2014, Azimi 2016	*Cinnamomum verum* (bark)	*Crocus sativus* (flower stigma)	*Elettaria cardamomum* (seedpod)	*Zingiber officinale* (rhizome)	Placebo (black tea)	A1=Powder, TDS, A2=Powder, TDS, A3=Powder, TDS, A4=Powder, TDS	A1=3 X 8, A2=1 X 8, A3=3 X 8, A4=3 X 8	A1=Black tea, A2=Black tea, A3=Black tea, A4=Black tea
***Enicostemma axillare*; Shilajit**								
Kumar 2014	*Enicostemma axillare* (whole plant extract)	Shilajit (*Asphaltum punjabianum*)	OAD			A1=Capsule, BD, A2=Capsule, BD	A1=1 X 13, A2=1 X 13	A1=Water, A2=Water
Enicostemma axillare+Emblica officinalis+Tinospora cordifolia								
Sharma 2019	*Enicostemma axillare* (whole plant)+*Emblica officinalis* (fruit)+*Tinospora cordifolia* (stem)	No additional medicine				A1=Capsule, BD+Powder, BD+Powder, BD	A1=2+6+6 X 26	A1=Lukewarm water
Linum usitatissimum*; *Plantago ovata								
Ricklefs-Johnson 2017	*Linum usitatissimum* (seed)	*Plantago ovata* (seed husk)				A1=Powder, Unclear, A2=Fiber, Unclear	A1=28 X 8, A2=9 X 8	
Syzygium cumini+Withania coagulans								
Siddiqui 2017	*Syzygium cumini* (seed?)+*Withania coagulans* (fruit)	OAD				A1=Powder, BD+Infusion, BD	A1=12+Unclear (10 pieces) X 13	A1=Water (infused in)
Trigonella foenum-graecum*; *Aegle marmelos*; *Trigonella foenum-graecum+Aegle marmelos								
Yaheya 2009	*Trigonella foenum-graecum* (seed)	*Aegle marmelos* (leaf)	*Trigonella foenum-graecum+ Aegle marmelos*	No additional medicine		A1=Powder, OD, A2=Decoction, OD, A3=Powder,	A1=20 X 16, A2=5 X 16, A3=20+5 X 16	

Author and year	Arm 1	Arm 2	Arm 3	Arm 4	Arm 5	Form and timing of administration of Ayurvedic medicine	Dose (g/day or otherwise mentioned) X duration (weeks) of Ayurvedic medicine	Anupan of Ayurvedic medicine
						OD+Decoction, OD		
**Ayurvedic medicine- combination of plant- and/or mineral-origin ingredients§**								
**AYUBES**								
Godatwar 2019	AYUBES (*Gymnema sylvestre*, *Momordica charantia*, *Pterocarpus marsupium*, *Andrographis paniculata*, *Aegle marmelos*, *Syzygium cumini*, *Enicostemma axillare*, *Picrorhiza kurroa*, *Azadirachta indica*, *Cyperus rotundus*)	Placebo				A1=Capsule, BD	A1=Unclear (4 capsules) X 13	A1=Lukewarm water
**BGR-34**								
Gupta 2018	BGR-34 (*Berberis aristata* [stem], *Pterocarpus marsupium* [heartwood], *Gymnema sylvestre* [leaf], *Rubia cordifolia* [root], *Trigonella foenum-graecum* [seed], *Tinospora cordifolia* [stem])	Placebo (Triphala)				A1=Tablet, BD	A1=1.24 X 16	
**Bilvadi Churna; Kiratadi Churna**								
Kumari 2016	Bilvadi Churna (*Aegle marmelos* [root], *Bacopa monnieri* [whole plant], *Tinospora cordifolia* [stem], *Withania somnifera* [root], *Piper longum* [fruit])	Kiratadi Churna (*Swertia chirayita* [whole plant], *Picrorhiza kurroa* [rhizome], *Trigonella foenum-graecum* [seed], *Tribulus terrestris* [fruit], *Azadirachta indica* [bark])	Placebo (barley)			A1=Powder, TDS, A2=Powder, TDS	A1=15 X 9, A2=15 X 9	A1=Lukewarm water, A2=Lukewarm water
**CardiPro**								
Fatima 2012	CardiPro (*Terminalia arjuna*, *Emblica officinalis* [fruit], *Withania somnifera*, *Boerhavia diffusa*, *Ocimum tenuiflorum*)	Placebo				A1=Capsule, TDS	A1=1.2 X 12	
**Cogent db**								
Shekhar 2002	Cogent db (*Azadirachta indica*, *Emblica officinalis* [fruit], *Curcuma longa* [rhizome], *Trigonella foenum-graecum*, *Syzygium cumini*, *Tribulus terrestris*, *Terminalia bellirica* [fruit], *Terminalia chebula* [fruit], *Rotula aquatica*)	No additional medicine				A1=Tablet, TDS	A1=60 X 13	
**DCBT 2345**								
Mohan 2001	DCBT 2345 (extracts of *Gymnema sylvestre*, *Syzygium cumini*, *Coccinia grandis*)	Placebo				A1=Tablet, TDS	A1=Unclear (6 tablets) X 26	
**Diabetea tea**								

Author and year	Arm 1	Arm 2	Arm 3	Arm 4	Arm 5	Form and timing of administration of Ayurvedic medicine	Dose (g/day or otherwise mentioned) X duration (weeks) of Ayurvedic medicine	Anupan of Ayurvedic medicine
Mahmoud 2016	Diabetea tea (extracts of *Camellia sinensis* [black tea; leaf], *Cassia tora* [leaf, seed], *Ficus racemosa* [bark, fruit], *Ficus bengalensis* [bark, fruit], *Syzygium cumini* [bark], *Terminalia arjuna* [bark], *Terminalia chebula* [fruit], *Terminalia bellirica* [fruit], *Emblica officinalis* [fruit], *Tribulus terrestris* [fruit], *Trigonella foenum-graecum* [seed], *Cardiospermum halicacabum* [leaf], *Cinnamomum verum* [bark], *Azadirachta indica* [leaf, seed])	Placebo (black tea)				A1=Infusion, TDS	A1=7.5 X 12	A1=Hot water (infused in)
Emblica officinalis*; *Withania somnifera*; *Emblica officinalis*, *Withania somnifera								
Usharani 2014	*Emblica officinalis* (fruit extract)	*Withania somnifera* (root+leaf extract)	*Emblica officinalis*, *Withania somnifera*			A1=Capsule, BD, A2=Capsule, BD, A3=Capsule, BD	A1=1 X 12, A2=1 X 12, A3=1 X 12	
**Herbal combination**								
Shokoohi 2017	Herbal combination (*Terminalia chebula* [fruit extract], *Commiphora wightii* [oleo-gum resin], *Commiphora myrrha* [oleo-gum resin])	Placebo				A1=Capsule, TDS	A1=1.8 X 13	
**Hyponidd**								
Poongothai 2002	Hyponidd (Shilajit [*Asphaltum punjabianum*], Yashad Bhasma [zinc preparation], *Momordica charantia* [fruit], *Curcuma longa* [rhizome extract], *Cassia auriculata* [seed extract], *Emblica officinalis* [fruit extract], *Syzygium cumini* [seed extract], *Gymnema sylvestre* [leaf extract], *Enicostemma axillare* [whole plant extract], *Azadirachta indica* [leaf extract], *Pterocarpus marsupium* [bark extract], *Tinospora cordifolia* [stem extract], *Swertia chirayita* [whole plant extract])	Placebo				A1=Tablet, TDS	A1=8.262 ? X 12	
**Inolter**								
Agrawal 2002	Inolter (*Momordica charantia* [fruit+seed+leaf extract], *Trigonella foenum-graecum* [seed+pod+leaf extract], Shilajit [*Asphaltum punjabianum*], *Gymnema sylvestre* [root, leaf], *Syzygium cumini* [seed, fruit, leaf, bark])	Placebo				A1=Capsule, OD?	A1=0.5? X 13	
**Kalpit**								
Agarwal 2013	Kalpit (*Holoptelea integrifolia* [bark], *Berberis aristata* [rhizome], *Mangifera indica* [seed], *Syzygium cumini* [seed], *Momordica charantia* [fruit], *Azadirachta indica*, *Aegle marmelos* [leaf], *Gymnema sylvestre* [leaf],	Kalpit+OAD	OAD			A1=Powder, BD, A2=Powder, BD	A1=10 X 9, A2=10 X 9	A1=Lukewarm water, A2=Lukewarm water

Author and year	Arm 1	Arm 2	Arm 3	Arm 4	Arm 5	Form and timing of administration of Ayurvedic medicine	Dose (g/day or otherwise mentioned) X duration (weeks) of Ayurvedic medicine	Anupan of Ayurvedic medicine
	Curcuma longa [rhizome], *Trigonella foenum-graecum* [seed], *Emblica officinalis* [fruit], *Terminalia bellirica* [fruit], *Terminalia chebula* [fruit])							
**Khadira-Kramuka Kashaya Ghanavati; Nishamalaki, Shilajit; Khadira-Kramuka Kashaya Ghanavati+Nishamalaki, Shilajit**								
Paliwal 2018	Khadira-Kramuka Kashaya Ghanavati (*Acacia catechu* [extract], *Areca catechu* [fruit])	Nishamalaki (*Curcuma longa* [rhizome], *Emblica officinalis* [fruit]), Shilajit (*Asphaltum punjabianum*)	Khadira-Kramuka Kashaya Ghanavati +Nishamalaki, Shilajit			A1=Tablet, BD, A2=Powder, BD, A3=Tablet, BD+Powder, BD	A1=1 X 13, A2=13 X 13, A3=1+13 X 13	
**Lodhradi Kashaya Ghana Vati**								
Bramhankar 2017	Lodhradi Kashaya Ghana Vati (extracts of *Symplocos racemosa*, *Terminalia chebula* [fruit], *Cyperus rotundus*, *Myrica esculenta*)	Lodhradi Kashaya Ghana Vati+OAD	OAD			A1=Tablet?, TDS, A2=Tablet?, TDS	A1=1.5 X 13, A2=1.5 X 13	A1=Warm water, A2=Warm water
**Madhumeha Nashini Gutika; Darvyadi Kwatha; Madhumeha Nashini Gutika+Darvyadi Kwatha**								
Bhawana 2015	Madhumeha Nashini Gutika (Trivanga Bhasma [tin, lead, zinc preparation], *Gymnema sylvestre* [leaf], *Azadirachta indica* [leaf], Shilajit [*Asphaltum punjabianum*])	Darvyadi Kwatha (*Berberis aristata*, *Cedrus deodara*, *Terminalia chebula* [fruit], *Terminalia bellirica* [fruit], *Emblica officinalis* [fruit], *Cyperus rotundus*)	Madhumeha Nashini Gutika+Darvyadi Kwatha			A1=Tablet, TDS, A2=Decoction, BD, A3=Tablet, TDS+Decoction, BD	A1=1.5 X 13, A2=40(ml) X 13, A3=1.5+40(ml) X 13	A1=Lukewarm water, A3=Lukewarm water (for Madhumeha Nashini Gutika)
**Mamajjaka Ghana Vati; Tejashiladi Vati**								
Bhagat 2017	Mamajjaka Ghana Vati (*Enicostemma axillare* [leaf], *Aconitum heterophyllum* [root], *Picrorhiza kurroa* [rhizome], *Piper longum* [fruit])	Tejashiladi Vati (*Cinnamomum tamala* [leaf], Shilajit [*Asphaltum punjabianum*], Yashad Bhasma [zinc preparation])				A1=Tablet, BD, A2=Tablet, BD	A1=1 X 8, A2=1 X 8	A1=Lukewarm water, A2=Lukewarm water
**Mamajjaka Ghana Vati; Trikatu Gutika**								
Kataria 2017	Mamajjaka Ghana Vati (*Enicostemma axillare* [leaf], *Aconitum heterophyllum* [root], *Picrorhiza kurroa* [rhizome], *Piper longum* [fruit])	Trikatu Gutika (*Piper longum* [fruit], *Zingiber officinale* [rhizome], *Piper nigrum* [fruit], *Terminalia chebula* [fruit], *Terminalia bellirica* [fruit], *Emblica officinalis* [fruit], *Commiphora wightii*				A1=Tablet, TDS, A2=Tablet, TDS	A1=6 X 8, A2=6 X 8	A1=Lukewarm water, A2=Lukewarm water

Author and year	Arm 1	Arm 2	Arm 3	Arm 4	Arm 5	Form and timing of administration of Ayurvedic medicine	Dose (g/day or otherwise mentioned) X duration (weeks) of Ayurvedic medicine	Anupan of Ayurvedic medicine
		[oleo-gum resin], *Tribulus terrestris* [root])						
**Mehagni**								
Gopalakrishna 2017	Mehagni (*Curcuma longa* [rhizome], *Emblica officinalis* [fruit], *Gymnema sylvestre*, *Salacia reticulata*)	No additional medicine				A1=Tablet, BD	A1=2 X 12	
**Mustadi Kwatha Ghana Vati**								
Kushwaha 2017	Mustadi Kwatha Ghana Vati (*Cyperus rotundus* [tuber], *Terminalia bellirica* [fruit rind], *Citrullus colocynthis* [root], *Marsdenia tenacissima* [root bark], *Cedrus deodara* [inner wood, oil], *Terminalia chebula* [fruit], *Curcuma longa* [rhizome], *Symplocos racemosa* [stem bark], *Emblica officinalis* [fruit])	Placebo (Godanti Bhasma)				A1=Tablet, BD	A1=1 X 9	A1=Lukewarm water
**Naga Bhasma, Nishamalaki; Nishamalaki, *Hordeum vulgare***								
Desale 2018	Naga Bhasma (lead preparation), Nishamalaki (*Curcuma longa* [rhizome], *Emblica officinalis* [fruit])✪	Nishamalaki (*Curcuma longa* [rhizome], *Emblica officinalis* [fruit]), *Hordeum vulgare* (seed)				A1=Capsule, OD✪, A2=Capsule, OD	A1=0.375 X 13✪, A2=0.5 X 13	A1=Honey✪, A2=Honey
Nigella sativa*, *Trigonella foenum-graecum								
Memon 2010a, Memon 2010b, Memon 2012	*Nigella sativa* (seed), *Trigonella foenum-graecum* (seed)	No additional medicine				A1=Capsule, BD?	A1=1 to 2 X 13	
**Nisha Katakadi Kashaya; Yashad Bhasma; Nisha Katakadi Kashaya+Yashad Bhasma**								
Srinivas 2018	Nisha Katakadi Kashaya (*Curcuma longa* [rhizome], *Strychnos potatorum*, *Acacia catechu*, *Emblica officinalis* [fruit], *Salacia chinensis*, *Berberis aristata*, *Mimosa pudica*, *Salix caprea*, *Cissampelos pareira*, *Mangifera indica*, *Terminalia chebula* [fruit], *Cyperus rotundus*)	Yashad Bhasma (zinc preparation)	Nisha Katakadi Kashaya+ Yashad Bhasma			A1=Decoction, BD, A2=Capsule, TDS, A3=Decoction, BD+Capsule, TDS	A1=30(ml) X 12, A2=0.375 X 12, A3=30(ml) +0.375 X 12	A1=Lukewarm water (diluted with), A2=Lukewarm water, A3=Lukewarm water (diluted with)+Lukewarm water
**Pancreas tonic**								

Author and year	Arm 1	Arm 2	Arm 3	Arm 4	Arm 5	Form and timing of administration of Ayurvedic medicine	Dose (g/day or otherwise mentioned) X duration (weeks) of Ayurvedic medicine	Anupan of Ayurvedic medicine
Hsia 2004	Pancreas tonic (extracts of *Aegle marmelos* [leaf], *Pterocarpus marsupium* [heartwood], *Syzygium cumini* [fruit], *Momordica charantia* [seed], *Gymnema sylvestre* [leaf], *Trigonella foenum-graecum* [seed], *Azadirachta indica* [seed], *Ficus racemosa*, *Tinospora cordifolia* [stem], *Cinnamomum tamala* [leaf])	Placebo				A1=Capsule, TDS	A1=Unclear (6 capsules) X 12	
**Polyherbal formulation**								
Awasthi 2015	Polyherbal formulation (extracts of *Berberis aristata*, *Cyperus rotundus*, *Cedrus deodara*, *Emblica officinalis* [fruit], *Terminalia chebula* [fruit], *Terminalia bellirica* [fruit])	OAD				A1=Capsule, Unclear	A1=0.5 to 3 X 24	
**Salasaradi Kashaya+Shilajit+Trivanga Bhasma+*Tinospora cordifolia*, *Azadirachta indica*; Hyponidd**								
Bhat 2012	Salasaradi Kashaya (*Shorea robusta* [bark], *Acacia catechu* [bark extract?], *Acacia polyacantha* [bark], *Areca catechu* [fruit], *Betula utilis* [bark], *Gymnema sylvestre* [leaf], *Ougeinia oojeinensis* [bark+stem extract?], *Santalum album* [stem extract], *Pterocarpus santalinus* [stem extract], *Dalbergia sissoo* [bark extract?], *Albizia lebbeck* [bark, seed], *Pterocarpus marsupium* [stem extract], *Anogeissus latifolia* [bark], *Terminalia arjuna* [bark], *Borassus flabellifer* [fruit, root], *Tectona grandis* [stem extract?], *Pongamia pinnata* [bark, leaf], *Holoptelea integrifolia* [bark], *Dipterocarpus turbinatus* [bark], *Aquilaria malaccensis* [stem extract])+Shilajit (*Asphaltum punjabianum*)+Trivanga Bhasma (tin, lead, zinc preparation)+*Tinospora cordifolia*, *Azadirachta indica* (bark)	Hyponidd (Shilajit [*Asphaltum punjabianum*], Yashad Bhasma [zinc preparation], *Momordica charantia* [fruit], *Curcuma longa* [rhizome extract], *Cassia auriculata* [seed extract], *Emblica officinalis* [fruit extract], *Syzygium cumini* [seed extract], *Gymnema sylvestre* [leaf extract], *Enicostemma axillare* [whole plant extract], *Azadirachta indica* [leaf extract], *Pterocarpus marsupium* [bark extract], *Tinospora cordifolia* [stem extract], *Swertia chirayita* [whole plant extract])				A1=Decoction, TDS+Capsule, BD+Powder?, OD+Powder, TDS, A2=Tablet, BD	A1=60(ml) +1+0.125+ 12 X 13, A2=5.508 ? X 13	A1=Water+ Water+Water+Lukewarm water, A2=Water
**Shilajit; Asanadi Ghana Vati**								
Gupta 2016	Shilajit (*Asphaltum punjabianum*)	Asanadi Ghana Vati (extracts of *Pterocarpus marsupium*, *Ougeinia oojeinensis*, *Betula utilis*, *Terminalia arjuna*, *Holoptelea integrifolia*, *Acacia catechu*, *Acacia*				A1=Capsule, BD, A2=Tablet, BD	A1=1 X 13, A2=2 X 13	

Author and year	Arm 1	Arm 2	Arm 3	Arm 4	Arm 5	Form and timing of administration of Ayurvedic medicine	Dose (g/day or otherwise mentioned) X duration (weeks) of Ayurvedic medicine	Anupan of Ayurvedic medicine
		polyacantha, *Albizia lebbeck*, *Dalbergia sissoo*, *Gymnema sylvestre*, *Santalum album*, *Pterocarpus santalinus*, *Berberis aristata*, *Borassus flabellifer*, *Butea monosperma*, *Aquilaria malaccensis*, *Tectona grandis*, *Shorea robusta*, *Anogeissus latifolia*, *Areca catechu*, *Holarrhena pubescens*, *Vateria indica*, *Dipterocarpus alatus*)						
**Swarnamakshika Bhasma**								
Taviad 2016	Swarnamakshika Bhasma (chalcopyrite preparation)	Placebo				A1=Capsule, BD	A1=1 X 8	A1=Honey
**Talapotaka Churna**								
Nille 2018	Talapotaka Churna (*Cassia auriculata*, *Emblica officinalis* [fruit], *Curcuma longa* [rhizome], *Berberis aristata*)✪	Talapotaka Churna+OAD	OAD			A1=Powder, TDS✪, A2=Powder, TDS	A1=12 X 9✪, A2=12 X 9	A1=Takra (butter milk) or warm water✪, A2=Warm water
Trigonella foenum-graecum*, *Ocimum tenuiflorum								
Mitra 2006	*Trigonella foenum-graecum* (seed), *Ocimum tenuiflorum* (leaf)✪	No medicine				A1=Powder, BD✪	A1=27.5, 52.5, 77.5, 102.5 X 9✪	A1=Water✪
***Triticum aestivum*; Nishamalaki; *Triticum aestivum*+Nishamalaki**								
Samagandi 2012	*Triticum aestivum* (grass)	Nishamalaki (*Curcuma longa* [rhizome], *Emblica officinalis* [fruit])	*Triticum aestivum*+ Nishamalaki			A1=Juice, BD, A2=Tablet, BD, A3=Juice, BD+Tablet, BD	A1=100(ml) X 9, A2=2 X 9, A3=100(ml)+2 X 9	
**Vidangadi Yoga**								
Deshpande 2018	Vidangadi Yoga (extracts of *Embelia ribes* [fruit], *Shorea robusta* [bark], *Terminalia arjuna* [bark], *Myrica esculenta* [bark], *Neolamarckia cadamba* [bark], *Symplocos*	OAD				A1=Tablet, TDS	A1=1.5 X 12	A1=Water

Author and year	Arm 1	Arm 2	Arm 3	Arm 4	Arm 5	Form and timing of administration of Ayurvedic medicine	Dose (g/day or otherwise mentioned) X duration (weeks) of Ayurvedic medicine	Anupan of Ayurvedic medicine
	racemosa [bark], *Pterocarpus marsupium* [bark], *Holarrhena pubescens* [bark])							
**Vijaysaradi Ghana Vati; Madhumehari Vati**								
Sharma 2018	Vijaysaradi Ghana Vati (*Pterocarpus marsupium* [heartwood], *Terminalia chebula* [fruit], *Emblica officinalis* [fruit], *Terminalia bellirica* [fruit], *Swertia chirayita* [whole plant], *Trichosanthes dioica* [leaf], *Picrorhiza kurroa* [rhizome], *Tribulus terrestris* [fruit], *Cyperus rotundus* [root], *Santalum album* [heartwood], *Berberis aristata* [stem, root], *Vetiveria zizanioides* [root])	Madhumehari Vati (*Enicostemma axillare* [whole plant], *Gymnema sylvestre* [leaf], *Caesalpinia bonduc* [nut], *Picrorhiza kurroa* [rhizome], *Piper longum* [fruit], *Capsicum frutescens* [fruit], *Citrullus colocynthis* [fruit])				A1=Tablet, TDS, A2=Tablet, TDS	A1=3 X 8, A2=3 X 8	A1=Water, A2=Water

A1, A2, A3, A4, A5=Eligible study arms (Ayurvedic detoxifying and purifying therapies [e.g., Panchakarma] are not mentioned but only Ayurvedic medicines [and their active ingredients] are mentioned), BD=Twice a day, OAD=Oral antidiabetic drug, OD=Once a day, QDS=Four times a day, TDS=Thrice a day

✪Two or more study arms were pooled together.

¶Voucher specimen number/other reference number provided for Ayurvedic medicines.

§Some of the Ayurvedic medicines contain single plant- or mineral-origin ingredient.

Appendix S6: Assessment of methodological quality of included studies.

Author and year	Q1	Q2	Q3	Q4	Q5	Q6	Q7	Q8	Q9	Q10	Q11	Q12	Q13	% of Y
**Ayurvedic medicine- single plant- or mineral-origin ingredient**														
Abelmoschus esculentus														
Moradi 2020	Y	U	N	U	U	U	U	N	U	Y	U	N	Y	23
Acacia Senegal														
Babiker 2017, Babiker 2018	U	U	N	U	Y	U	Y	N	N	Y	U	N	Y	31
Aegle marmelos														
Sankhla 2009	U	U	N	U	U	U	U	N	N	Y	U	N	Y	15
Sharma 2013	U	U	Y	Y	U	Y	Y	Y	Y	Y	U	N	Y	62
Nigam 2019	U	U	N	U	U	U	U	N	N	Y	U	N	Y	15
Allium sativum														
Ashraf 2005	U	U	Y	Y	U	U	U	N	N	Y	U	N	Y	31
Ashraf 2011a	U	U	Y	U	U	U	U	N	N	Y	U	N	Y	23
Ashraf 2011b	U	U	N	U	U	U	Y	N	N	U	U	N	Y	15

Author and year	Q1	Q2	Q3	Q4	Q5	Q6	Q7	Q8	Q9	Q10	Q11	Q12	Q13	% of Y
Kumar 2013	U	U	U	N	N	N	Y	U	U	Y	U	N	Y	23
Aloe vera														
Arora 2009	U	U	N	U	U	U	Y	N	N	U	U	N	Y	15
Huseini 2012a, Huseini 2012b	U	U	N	Y	U	U	Y	N	N	Y	U	N	Y	31
Zarrintan 2015	Y	Y	N	Y	Y	Y	Y	N	N	Y	U	N	Y	62
Anethum graveolens														
Mobasseri 2014	Y	U	N	Y	U	U	Y	N	N	Y	U	N	Y	38
Haidari 2020	Y	U	N	Y	U	U	Y	N	N	Y	U	N	Y	38
Azadirachta indica														
Usharani 2020	Y	U	N	Y	Y	U	Y	N	N	U	U	N	Y	38
Berberis aristata														
Sharma 2017	U	U	N	N	N	N	Y	N	N	Y	U	N	Y	23
Boswellia serrata														
Azadmehr 2014	U	U	Y	Y	U	U	Y	Y	Y	Y	U	N	Y	54
Mehrzadi 2018	Y	U	N	Y	Y	U	Y	N	Y	Y	U	N	Y	54
Camellia sinensis														
MacKenzie 2007	Y	U	N	U	Y	U	Y	N	N	U	U	N	Y	31
Mirzaei 2009	U	U	N	U	U	U	U	U	Y	Y	U	N	Y	23
Hsu 2011	Y	U	Y	Y	Y	U	Y	N	N	Y	U	N	Y	54
Lasaite 2014	U	U	Y	U	U	U	U	N	N	Y	U	N	Y	23
Liu 2014	Y	Y	N	Y	Y	U	Y	N	N	Y	U	N	Y	54
Quezada-Fernandez 2019	U	U	N	Y	U	U	Y	N	N	Y	U	N	Y	31
Capparis spinosa														
Huseini 2013	U	U	N	Y	U	Y	Y	N	N	Y	U	N	Y	38
Cichorium intybus														
Chandra 2020	U	U	Y	Y	U	Y	Y	N	N	Y	U	N	Y	46
Cinnamomum aromaticum														
Mang 2006	U	U	N	Y	U	U	U	N	N	Y	U	N	Y	23
Suppapitiporn 2006	U	U	N	U	U	U	Y	Y	Y	U	U	N	Y	31
Blevins 2007	U	U	N	U	U	U	U	N	N	U	U	N	Y	8
Crawford 2009	Y	U	Y	N	U	Y	Y	N	Y	Y	U	N	Y	54
Akilen 2010	Y	Y	Y	Y	Y	U	U	N	Y	Y	U	N	Y	62
Wainstein 2011	U	U	N	Y	U	U	U	U	Y	U	U	N	Y	23
Lu 2012	U	U	N	Y	Y	U	Y	N	N	Y	U	N	Y	38
Sharma 2012	U	U	N	Y	U	Y	Y	U	Y	U	U	N	Y	38
Hasanzade 2013	U	U	N	Y	U	U	Y	U	Y	Y	U	N	Y	38
Tangvarasittichai 2015, Sengsuk 2016	Y	Y	N	Y	U	U	Y	N	N	Y	U	N	Y	46
Cinnamomum verum														
Vafa 2012	U	U	N	Y	U	U	Y	N	N	Y	U	N	Y	31
Zahmatkesh 2012	U	U	N	U	U	U	U	N	N	U	U	N	Y	8
Talaei 2017	U	U	Y	Y	U	U	Y	N	N	Y	U	N	Y	38
Zare 2019	Y	Y	N	Y	Y	U	U	N	N	Y	U	N	Y	46
Mirmiranpour 2020	Y	U	Y	U	U	U	U	N	N	Y	U	N	Y	31
Citrullus colocynthis														
Huseini 2009	Y	Y	N	Y	U	Y	Y	Y	Y	Y	U	N	Y	69

Author and year	Q1	Q2	Q3	Q4	Q5	Q6	Q7	Q8	Q9	Q10	Q11	Q12	Q13	% of Y
Barghamdi 2016	U	U	N	Y	U	U	Y	Y	Y	U	U	N	Y	38
Coccinia grandis														
Kuriyan 2008, Kurpad 2008	U	U	N	U	U	U	Y	N	N	Y	U	N	Y	23
Quamri 2017	Y	U	Y	U	U	U	U	U	U	U	U	N	Y	23
Wasana 2021	Y	U	N	Y	U	U	Y	N	Y	Y	U	N	Y	46
Convolvulus prostratus														
Patel 2012	U	U	N	U	U	U	U	N	N	U	U	N	Y	8
Crocus sativus														
Milajerdi 2018	Y	N	N	Y	U	U	Y	N	N	Y	U	N	Y	38
Aleali 2019	U	U	N	Y	U	U	Y	N	N	Y	U	N	Y	31
Ebrahimi 2019	Y	U	N	Y	U	U	Y	N	N	Y	U	N	Y	38
Mobasseri 2020	Y	U	N	U	U	U	U	N	N	Y	U	N	Y	23
Cuminum cyminum														
Jafari 2017	U	U	N	Y	U	U	Y	N	N	Y	U	N	Y	31
Hendre 2020	U	U	U	U	U	U	Y	U	U	U	U	U	Y	15
Curcuma longa														
Usharani 2008	U	U	Y	U	U	U	U	N	N	U	U	N	Y	15
Na 2013	Y	U	Y	Y	U	Y	Y	N	N	Y	U	N	Y	54
Chuengsamarn 2014	Y	Y	Y	Y	U	U	Y	N	N	U	U	Y	Y	54
Panahi 2017, Panahi 2018	U	U	N	Y	U	U	Y	N	N	U	U	N	Y	23
Adab 2019	U	U	N	Y	U	U	Y	N	N	Y	U	N	Y	31
Adibian 2019	U	U	N	Y	U	U	Y	N	N	U	U	N	Y	23
Srinivasan 2019	Y	U	N	Y	U	U	Y	N	N	Y	U	N	Y	38
de Sousa 2020	U	U	N	U	U	U	U	N	N	U	U	N	Y	8
Cyamopsis tetragonoloba														
Uusitupa 1984	U	U	N	U	U	U	Y	N	N	Y	U	N	Y	23
Uusitupa 1989	U	U	N	U	U	U	Y	Y	Y	Y	U	N	Y	38
Eclipta prostrata														
Sazia 2015a, Sazia 2015b	Y	U	U	U	U	U	Y	N	N	Y	U	N	Y	31
Elettaria cardamomum														
Aghasi 2019	U	U	N	Y	U	U	Y	N	Y	Y	U	N	Y	38
Emblica officinalis														
Usharani 2013	U	U	N	Y	U	U	U	U	U	U	U	N	Y	15
Enicostemma axillare														
Shankarrao 2017	U	U	U	N	N	N	Y	U	U	U	U	N	Y	15
Gynostemma pentaphyllum														
Huyen 2010	U	U	N	U	U	U	Y	Y	N	Y	U	N	Y	31
Huyen 2012	U	U	N	U	U	U	Y	Y	Y	Y	U	N	Y	38
Hibiscus sabdariffa														
Sarbini 2019	Y	U	N	Y	U	Y	Y	N	N	Y	U	N	Y	46
Ipomoea batatas														
Ludvik 2004	U	U	N	U	U	U	Y	U	U	U	U	N	Y	15
Ludvik 2008	U	U	N	U	U	U	Y	N	N	Y	U	N	Y	23
Juglans regia														
Hosseini 2014a	U	U	Y	Y	U	Y	Y	N	N	Y	U	N	Y	46

Author and year	Q1	Q2	Q3	Q4	Q5	Q6	Q7	Q8	Q9	Q10	Q11	Q12	Q13	% of Y
Hosseini 2014b	U	U	N	Y	U	U	Y	Y	Y	Y	U	N	Y	46
Abdoli 2017	Y	U	N	Y	U	U	Y	N	N	Y	U	N	Y	38
Zibaeenezhad 2016, Zibaeenezhad 2017	Y	U	N	U	U	Y	Y	N	N	Y	U	N	Y	38
Rabiei 2018	U	U	N	U	U	U	Y	N	N	U	U	N	Y	15
Linum usitatissimum														
Barre 2008	U	U	N	U	U	U	U	N	N	Y	U	N	Y	15
Hashemzadeh 2017	Y	U	Y	Y	U	U	U	Y	Y	Y	U	N	Y	54
Momordica charantia														
Dans 2007	Y	U	Y	Y	U	U	Y	N	Y	U	U	N	Y	46
Zänker 2012	U	U	Y	Y	U	U	Y	N	N	U	U	N	Y	31
Trakoon-osot 2013	U	U	Y	U	U	U	Y	Y	Y	Y	U	N	Y	46
Rahman 2015	U	U	N	U	U	U	Y	N	N	Y	U	N	Y	23
Suthar 2016a	U	U	N	N	N	N	U	N	N	U	U	N	Y	8
Suthar 2016b	U	U	N	N	N	N	Y	N	N	U	U	N	Y	15
Cortez-Navarrete 2018	U	U	N	Y	U	U	Y	N	N	Y	U	N	Y	31
Kumari 2018	Y	U	N	U	U	U	Y	Y	Y	Y	U	N	Y	46
Amini 2020	U	U	Y	Y	U	U	U	N	N	Y	U	N	Y	31
Kim 2020	U	U	N	U	U	U	U	N	N	U	U	N	Y	8
Nigella sativa														
Najmi 2012	U	U	N	N	N	N	Y	U	Y	U	U	N	Y	23
Hosseini 2013	U	U	Y	U	U	U	Y	Y	Y	Y	U	N	Y	46
Hadi 2015	U	U	N	U	U	U	Y	N	N	U	U	N	Y	15
Heshmati 2015	Y	U	N	Y	U	U	U	N	Y	Y	U	N	Y	38
Kaatabi 2015	Y	U	N	Y	U	U	Y	N	N	Y	U	N	Y	38
Moustafa 2019	Y	U	N	N	N	N	Y	N	N	U	U	N	Y	23
Kooshki 2020	U	U	N	Y	U	U	Y	U	U	U	U	N	Y	23
Jangjo-Borazjani 2021	U	U	N	Y	U	U	U	U	Y	Y	U	N	Y	31
Plantago ovata														
Ziai 2005	U	U	N	U	U	U	Y	N	N	Y	U	N	Y	23
Feinglos 2013	U	U	N	U	U	U	Y	N	U	U	U	N	Y	15
Abutair 2016	U	U	N	U	U	U	U	N	N	Y	U	N	Y	15
Portulaca oleracea														
El-Sayed 2011	U	U	N	U	U	U	U	U	U	Y	U	N	Y	15
Farzanegi 2014	U	U	N	U	U	U	U	U	U	Y	U	N	U	8
Dehghan 2016	U	U	N	U	U	U	U	U	U	Y	U	N	Y	15
Wainstein 2016	Y	U	N	Y	U	U	U	N	Y	Y	U	N	Y	38
Pterocarpus marsupium														
Hariharan 2005	U	U	Y	Y	Y	U	Y	N	N	Y	U	U	Y	46
Punica granatum														
Faghihimani 2016	U	U	N	U	U	U	Y	N	N	Y	U	N	Y	23
Babaeian 2013	U	U	Y	U	U	U	Y	N	N	Y	U	N	Y	31
Sohrab 2014, Sohrab 2015	U	U	N	U	U	U	Y	N	N	Y	U	N	Y	23
Khajebishak 2019a, Khajebishak 2019b	U	U	N	Y	U	U	U	N	N	Y	U	N	Y	23
Grabež 2020	U	U	N	Y	U	U	Y	Y	Y	Y	U	N	Y	46
Hashemi 2020	Y	U	N	U	Y	U	Y	N	N	U	U	N	Y	31

Author and year	Q1	Q2	Q3	Q4	Q5	Q6	Q7	Q8	Q9	Q10	Q11	Q12	Q13	% of Y
Sesamum indicum														
Shahi 2017	U	U	N	U	U	U	U	N	N	Y	U	N	Y	15
Aslam 2018	U	U	N	U	U	U	Y	N	N	Y	U	N	Y	23
**Shilajit**														
Narasimha Raju 2016	U	U	N	U	U	U	U	N	N	U	U	N	Y	8
Niranjan 2016	U	U	Y	U	U	U	U	Y	Y	U	U	N	Y	31
Syzygium cumini														
Sahana 2010	Y	U	N	N	N	N	Y	N	U	U	U	N	Y	23
Sidana 2016, Sidana 2017	U	U	N	U	U	U	Y	N	N	U	U	N	Y	15
Terminalia chebula														
Usharani 2020	Y	U	N	Y	Y	U	U	Y	Y	U	U	N	Y	46
Tinospora cordifolia														
Mishra 2015	U	U	N	U	U	U	Y	U	U	Y	U	N	Y	23
Roy 2015	U	U	N	N	N	N	Y	U	U	U	U	N	Y	15
Tribulus terrestris														
Samani 2016	U	U	N	Y	Y	U	Y	N	N	Y	U	N	Y	38
Trigonella foenum-graecum														
Gupta 2001	U	U	N	U	U	U	U	U	Y	U	U	N	Y	15
Lu 2008	Y	U	N	Y	U	U	Y	Y	Y	Y	U	N	Y	54
Ansari 2011	Y	U	U	U	U	U	U	N	N	U	U	U	Y	15
Rafraf 2014	Y	U	N	U	U	U	Y	N	N	Y	U	N	Y	31
Suchitra 2015	U	U	Y	U	U	U	U	Y	Y	U	U	N	Y	31
Kaur 2016	Y	U	U	N	N	N	Y	Y	Y	U	U	N	Y	38
Singh 2016	U	U	N	N	N	N	U	Y	Y	Y	U	N	Y	31
Verma 2016	Y	U	U	U	U	U	Y	U	U	Y	U	N	Y	31
Ranade 2017	Y	U	Y	N	U	U	Y	U	U	U	U	N	Y	31
Gholaman 2018	U	U	N	U	U	U	Y	Y	Y	U	U	N	U	23
Kandhare 2018	Y	U	N	Y	U	U	Y	N	N	U	U	N	Y	31
Hassani 2019	U	U	N	U	U	U	Y	N	N	Y	U	N	Y	23
Hota 2019	U	U	Y	N	N	N	Y	U	U	U	U	U	Y	23
Najdi 2019	Y	Y	N	N	N	N	Y	N	N	U	U	N	Y	31
Rashid 2019	U	U	N	U	U	U	Y	Y	Y	U	U	N	Y	31
Hadi 2020	U	U	N	U	U	Y	Y	N	N	Y	U	N	Y	31
Urtica dioica														
Namazi 2011, Esfanjani 2012a, Esfanjani 2012b	U	U	N	U	U	U	Y	N	N	Y	U	N	Y	23
Kianbakht 2013	Y	Y	N	Y	Y	N	Y	N	N	Y	U	N	Y	54
Khajeh-Mehrizi 2014	U	U	N	U	U	U	Y	N	N	Y	U	N	Y	23
Dabagh 2016	U	U	Y	U	U	U	U	U	U	Y	U	N	U	15
Hassani 2016	U	U	N	U	U	U	U	N	N	Y	U	N	Y	15
Dadvar 2017	U	U	N	U	U	U	U	U	U	Y	U	N	U	8
Ghalavand 2017	U	U	Y	U	U	U	U	U	U	Y	U	N	U	15
Korani 2017	U	U	N	U	U	U	U	N	N	Y	U	N	Y	15
Mohammadnia 2017	U	U	U	U	U	U	U	N	N	Y	U	N	U	8
Vernonia cinerea														
Sayeed 2013	Y	U	N	Y	U	U	Y	U	U	Y	U	N	Y	38

Author and year	Q1	Q2	Q3	Q4	Q5	Q6	Q7	Q8	Q9	Q10	Q11	Q12	Q13	% of Y
Withania coagulans														
Hemalatha 2018	U	U	U	U	U	U	Y	U	U	Y	U	N	Y	23
Withania somnifera														
Usharani 2014	U	U	N	Y	U	U	U	N	N	U	U	N	Y	15
Zingiber officinale														
Mahluji 2013	U	U	N	Y	U	U	Y	N	N	Y	U	N	Y	31
Arablou 2014a, Arablou 2014b	U	U	N	Y	U	U	Y	N	N	Y	U	N	Y	31
Mozaffari-Khosravi 2014, Talaei 2017, Talaei 2018	Y	U	N	Y	U	U	Y	N	N	U	U	N	Y	31
Shidfar 2015	U	U	N	Y	U	U	Y	N	N	Y	U	N	Y	31
Arzati 2017, Zarezadeh 2018	U	U	N	Y	U	U	Y	N	N	Y	U	N	Y	31
Mohammadi 2017, Mohammadi 2019	U	U	N	U	U	U	U	U	Y	Y	U	N	U	15
Carvalho 2020	Y	U	N	Y	U	U	Y	N	N	Y	U	N	Y	38
Gholinezhad 2020	U	U	N	Y	U	U	U	N	N	Y	U	N	Y	23
Ziziphus mauritiana														
Yazdanpanah 2017	Y	U	N	U	U	Y	Y	N	N	Y	U	N	Y	38
Acalypha indica/Allium cepa/Allium sativum/Azadirachta indica/Mangifera indica/Murraya koenigii/Musa sapientum/Ocimum tenuiflorum/Phyllanthus amarus/Tinospora cordifolia														
Balasubramaniam 2010	Y	U	U	U	U	U	Y	Y	Y	Y	U	N	Y	46
Allium sativum*; *Cuminum cyminum														
Mansouri 2018	U	U	N	U	U	U	U	U	U	U	U	N	Y	8
Aloe vera*; *Pterocarpus marsupium*; *Aloe vera+Pterocarpus marsupium														
Maurya 2017	N	U	N	U	N	N	Y	N	N	U	U	N	N	8
Cinnamomum verum*; *Crocus sativus*; *Elettaria cardamomum*; *Zingiber officinale														
Azimi 2014, Azimi 2016	U	U	N	U	U	U	U	N	N	Y	U	N	Y	15
***Enicostemma axillare*; Shilajit**														
Kumar 2014	U	U	N	U	U	U	U	N	N	U	U	N	U	0
Enicostemma axillare+Emblica officinalis+Tinospora cordifolia														
Sharma 2019	U	U	N	N	N	N	Y	N	N	U	U	N	Y	15
Linum usitatissimum*; *Plantago ovata														
Ricklefs-Johnson 2017	Y	U	N	U	U	U	Y	N	N	Y	U	N	Y	31
Syzygium cumini+Withania coagulans														
Siddiqui 2017	Y	U	Y	N	N	N	U	N	N	U	U	N	Y	23
Trigonella foenum-graecum*; *Aegle marmelos*; *Trigonella foenum-graecum+Aegle marmelos														
Yaheya 2009	U	U	U	U	U	U	U	Y	Y	Y	U	N	Y	31
**Ayurvedic medicine- combination of plant- and/or mineral-origin ingredients§**														
**AYUBES**														
Godatwar 2019	Y	U	N	Y	U	U	N	U	N	U	U	N	Y	23
**BGR-34**														
Gupta 2018	U	U	N	U	U	U	U	N	N	U	U	N	Y	8
**Bilvadi Churna; Kiratadi Churna**														
Kumari 2016	U	U	N	U	U	U	U	U	U	U	U	N	Y	8
**CardiPro**														
Fatima 2012	U	U	N	Y	U	U	U	N	N	U	U	N	Y	15
**Cogent db**														
Shekhar 2002	U	U	N	U	U	U	Y	N	N	U	U	N	U	8
**DCBT 2345**														

Author and year	Q1	Q2	Q3	Q4	Q5	Q6	Q7	Q8	Q9	Q10	Q11	Q12	Q13	% of Y
Mohan 2001	U	U	N	Y	U	U	Y	N	N	Y	U	N	Y	31
**Diabetea tea**														
Mahmoud 2016	U	U	N	U	U	U	Y	N	N	Y	U	N	Y	23
Emblica officinalis*; *Withania somnifera*; *Emblica officinalis*, *Withania somnifera														
Usharani 2014	U	U	Y	U	U	U	U	N	N	U	U	N	Y	15
**Herbal combination**														
Shokoohi 2017	U	U	N	Y	Y	U	Y	N	Y	Y	U	N	Y	46
**Hyponidd**														
Poongothai 2002	U	U	N	Y	U	U	Y	N	N	Y	U	N	Y	31
**Inolter**														
Agrawal 2002	U	U	Y	Y	U	U	Y	Y	Y	Y	U	N	Y	54
**Kalpit**														
Agarwal 2013	U	U	N	U	U	U	U	U	Y	U	U	N	Y	15
**Khadira-Kramuka Kashaya Ghanavati; Nishamalaki, Shilajit; Khadira-Kramuka Kashaya Ghanavati+Nishamalaki, Shilajit**														
Paliwal 2018	U	N	N	U	U	U	U	U	U	U	U	N	Y	8
**Lodhradi Kashaya Ghana Vati**														
Bramhankar 2017	U	U	N	U	U	U	Y	N	N	U	U	N	Y	15
**Madhumeha Nashini Gutika; Darvyadi Kwatha; Madhumeha Nashini Gutika+Darvyadi Kwatha**														
Bhawana 2015	U	U	N	U	U	U	U	U	U	U	U	N	Y	8
**Mamajjaka Ghana Vati; Tejashiladi Vati**														
Bhagat 2017	Y	U	N	N	N	N	Y	N	N	U	U	N	Y	23
**Mamajjaka Ghana Vati; Trikatu Gutika**														
Kataria 2017	U	U	N	U	U	U	Y	Y	Y	U	U	N	Y	31
**Mehagni**														
Gopalakrishna 2017	U	U	N	N	N	N	N	Y	Y	Y	U	N	Y	31
**Mustadi Kwatha Ghana Vati**														
Kushwaha 2017	U	U	N	N	N	N	U	U	U	Y	U	N	Y	15
**Naga Bhasma, Nishamalaki; Nishamalaki, *Hordeum vulgare***														
Desale 2018	Y	U	N	U	U	U	Y	N	N	U	U	N	Y	23
Nigella sativa*, *Trigonella foenum-graecum														
Memon 2010a, Memon 2010b, Memon 2012	U	U	N	N	N	N	Y	Y	Y	Y	U	N	Y	38
**Nisha Katakadi Kashaya; Yashad Bhasma; Nisha Katakadi Kashaya+Yashad Bhasma**														
Srinivas 2018	Y	U	Y	N	N	N	U	N	N	U	U	N	Y	23
**Pancreas tonic**														
Hsia 2004	U	U	N	Y	U	U	Y	N	N	Y	U	N	Y	31
**Polyherbal formulation**														
Awasthi 2015	Y	U	N	N	N	N	Y	N	N	Y	U	N	Y	31
**Salasaradi Kashaya+Shilajit+Trivanga Bhasma+*Tinospora cordifolia*, *Azadirachta indica*; Hyponidd**														
Bhat 2012	U	U	N	U	U	U	Y	N	N	U	U	N	Y	15
**Shilajit; Asanadi Ghana Vati**														
Gupta 2016^	U	U	U	U	U	U	U	N	N	U	U	N	Y	8
**Swarnamakshika Bhasma**														
Taviad 2016	Y	U	N	U	U	U	U	N	N	U	U	N	Y	15
**Talapotaka Churna**														
Nille 2018	U	U	N	U	U	U	U	N	N	U	U	N	Y	8

Author and year	Q1	Q2	Q3	Q4	Q5	Q6	Q7	Q8	Q9	Q10	Q11	Q12	Q13	% of Y
Trigonella foenum-graecum*, *Ocimum tenuiflorum														
Mitra 2006	U	U	U	U	U	U	Y	U	U	Y	U	N	Y	23
***Triticum aestivum*; Nishamalaki; *Triticum aestivum*+Nishamalaki**														
Samagandi 2012	U	U	N	U	U	U	U	Y	Y	U	U	N	Y	23
**Vidangadi Yoga**														
Deshpande 2018	Y	Y	N	N	N	N	U	N	N	U	U	N	Y	23
**Vijaysaradi Ghana Vati; Madhumehari Vati**														
Sharma 2018	Y	U	N	N	N	N	U	N	N	U	U	N	Y	15

Y=Yes, N=No, U=Unclear; JBI critical appraisal checklist for RCTs: 1. Was true randomization used for assignment of participants to treatment groups? 2. Was allocation to treatment groups concealed? 3. Were treatment groups similar at the baseline? 4. Were participants blind to treatment assignment? 5. Were those delivering treatment blind to treatment assignment? 6. Were outcomes assessors blind to treatment assignment? 7. Were treatment groups treated identically other than the intervention of interest? 8. Was follow up complete and if not, were differences between groups in terms of their follow up adequately described and analyzed? 9. Were participants analyzed in the groups to which they were randomized? 10. Were outcomes measured in the same way for treatment groups? 11. Were outcomes measured in a reliable way? 12. Was appropriate statistical analysis used? 13. Was the trial design appropriate, and any deviations from the standard RCT design (individual randomization, parallel groups) accounted for in the conduct and analysis of the trial?

§Some of the Ayurvedic medicines contain single plant- or mineral-origin ingredient.

^Extracted only adverse event data.

Appendix S7: 89 comparisons of Ayurvedic medicines which could not be included in meta-analyses.

Abelmoschus esculentus (Moradi 2020)

Outcomes	Number of studies	Mean Diff. with 95% CI	Heterogeneity (%)
HbA1c	1	0.36 [-0.20, 0.92]	N/A
FBG	1	-13.03 [-37.49, 11.43]	N/A
Fasting insulin*	1	-0.27 [-2.98, 2.44]	N/A
Insulin resistance	1	-0.78 [-2.40, 0.84]	N/A
Body weight	1	-1.80 [-6.85, 3.25]	N/A
BMI	1	-0.92 [-2.69, 0.85]	N/A
SBP	1	-2.76 [-7.50, 1.98]	N/A
DBP	1	1.89 [-1.78, 5.56]	N/A
TC	1	-21.00 [-40.11, -1.89]	N/A
HDL-C	1	1.51 [-2.00, 5.02]	N/A
LDL-C	1	-9.04 [-27.92, 9.84]	N/A
TG	1	-36.10 [-61.71, -10.49]	N/A

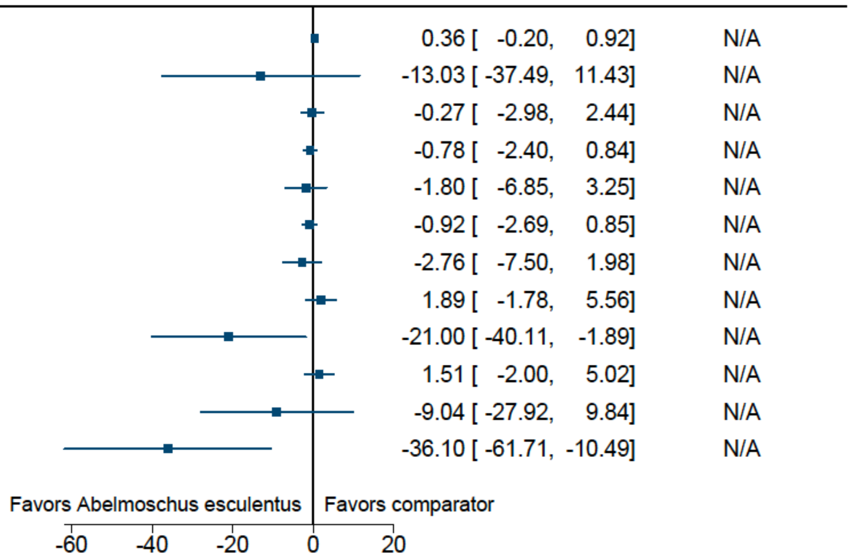

*Favors intervention/comparator as insulin sensitizer

Acacia senegal (Babiker 2017, Babiker 2018)

Outcomes	Number of studies	Mean Diff. with 95% CI	Heterogeneity (%)
HbA1c	1	-1.00 [-1.91, -0.09]	N/A
FBG	1	-21.39 [-36.74, -6.04]	N/A
Body weight	1	-5.68 [-11.89, 0.53]	N/A
BMI	1	-2.87 [-5.34, -0.40]	N/A
Waist circumference	1	1.22 [-4.39, 6.83]	N/A
SBP	1	-3.37 [-7.89, 1.15]	N/A
DBP	1	-0.83 [-4.32, 2.66]	N/A
TC	1	-2.58 [-17.52, 12.36]	N/A
HDL-C	1	-7.29 [-15.42, 0.84]	N/A
LDL-C	1	-14.60 [-32.22, 3.02]	N/A
TG	1	23.53 [3.22, 43.84]	N/A

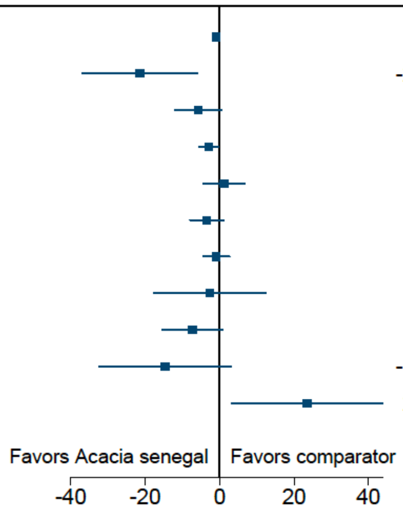

Acalypha indica (Balasubramaniam 2010)

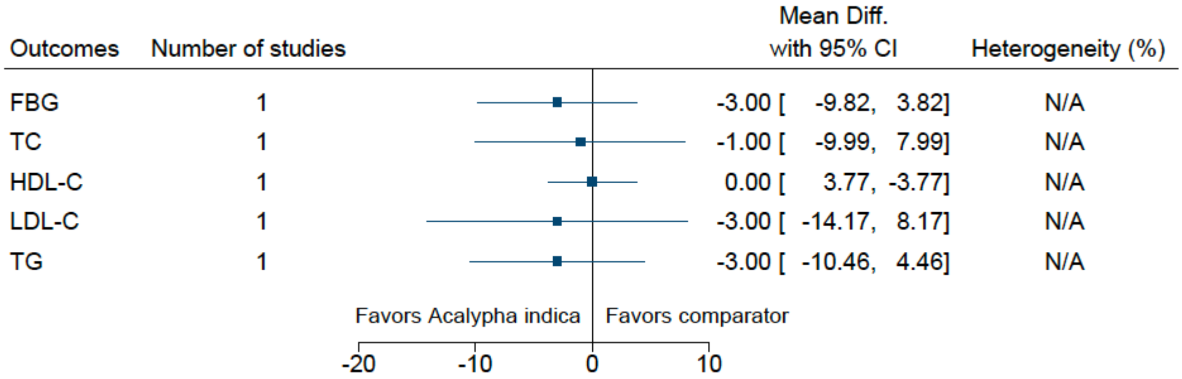

Allium cepa (Balasubramaniam 2010)

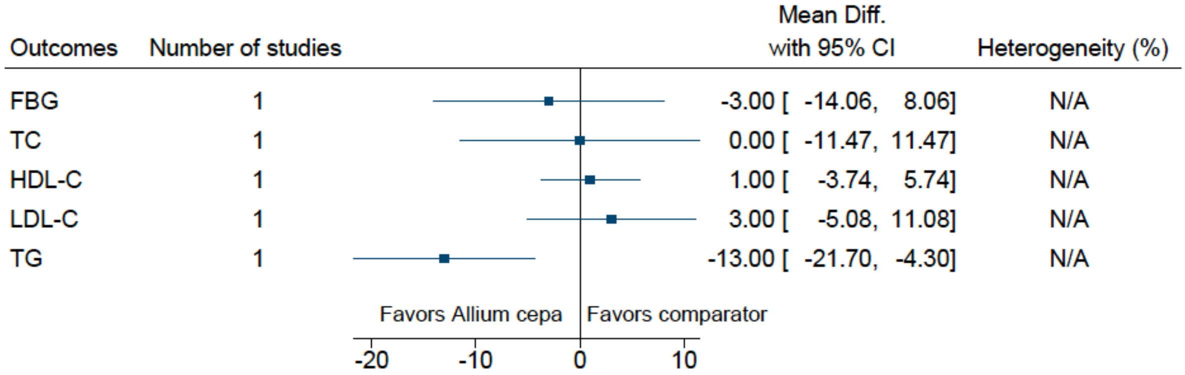

Allium sativum (versus OAD) (Ashraf 2011a)

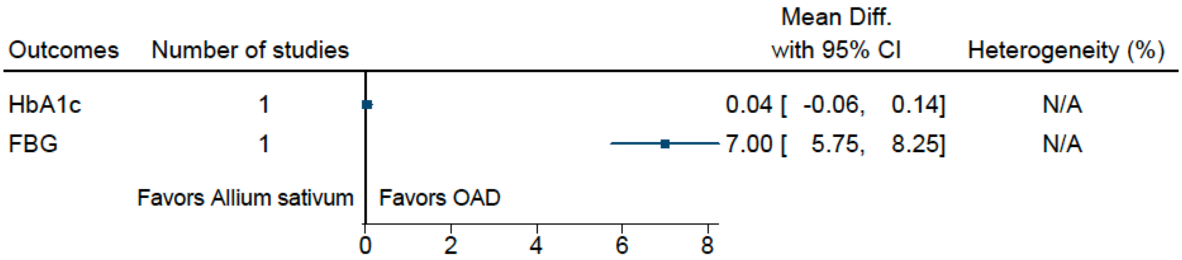

Allium sativum versus *Cuminum cyminum* (Mansouri 2018)

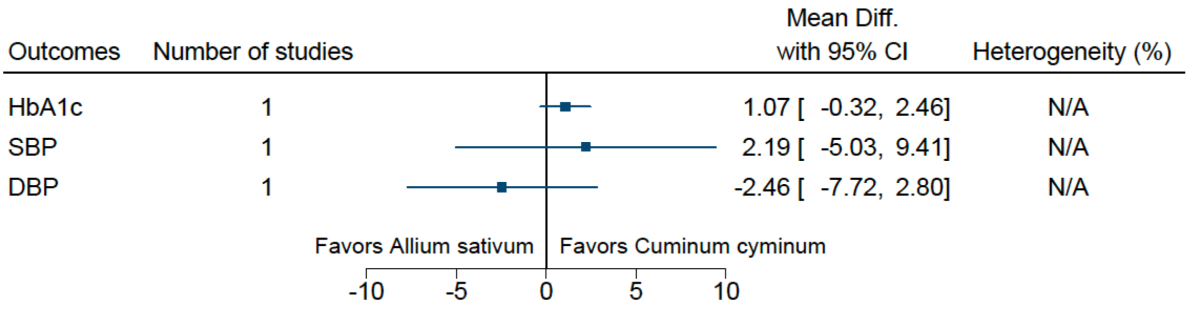

AYUBES (Godatwar 2019)

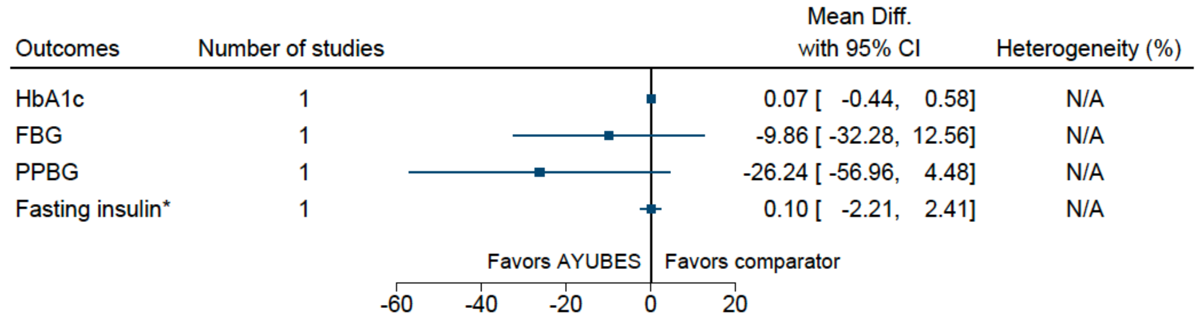

*Favors intervention/comparator as insulin sensitizer

Berberis aristata (Sharma 2017)

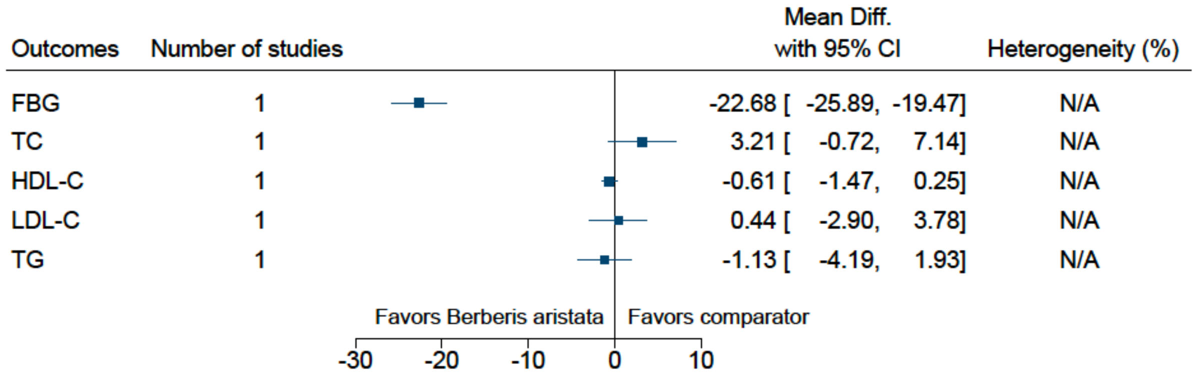

BGR-34 (Gupta 2018)

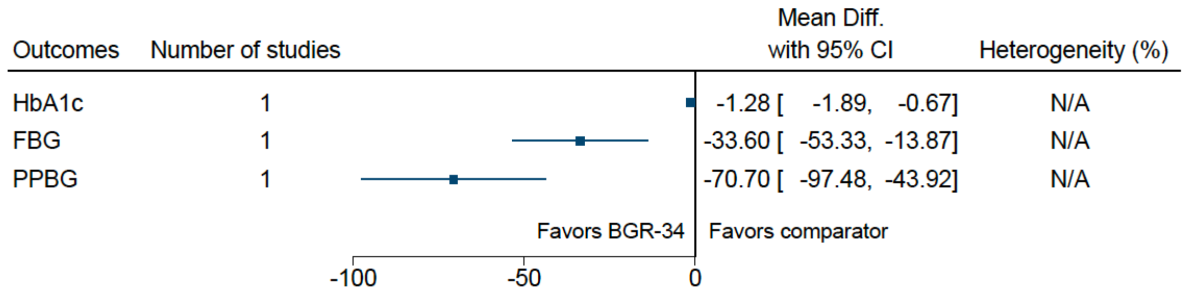

Bilvadi Churna (Kumari 2016)

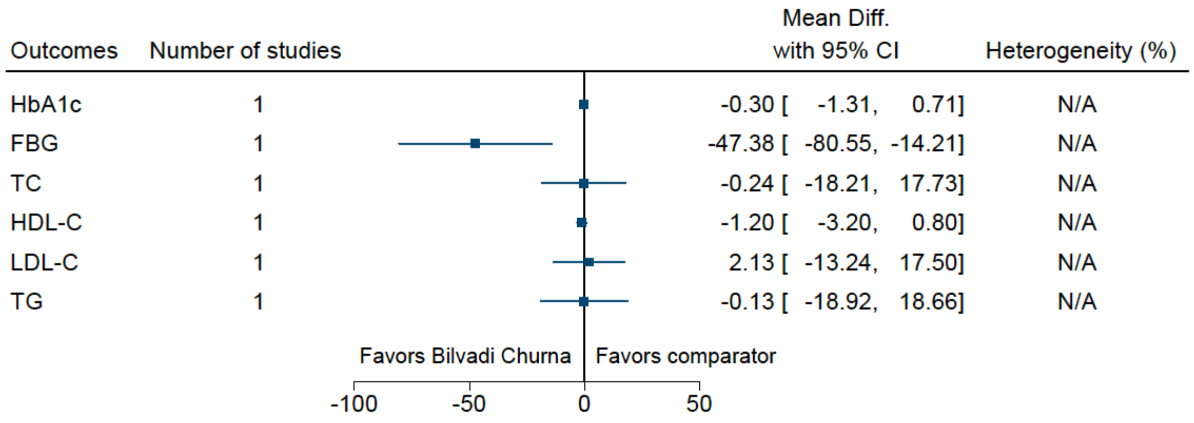

Bilvadi Churna versus Kiratadi Churna (Kumari 2016)

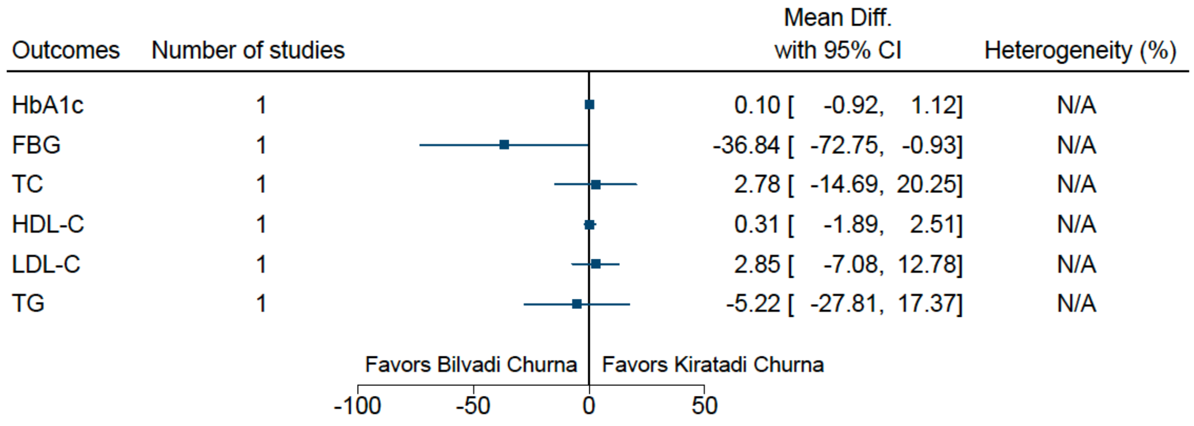

Capparis spinosa **(Huseini 2013)**

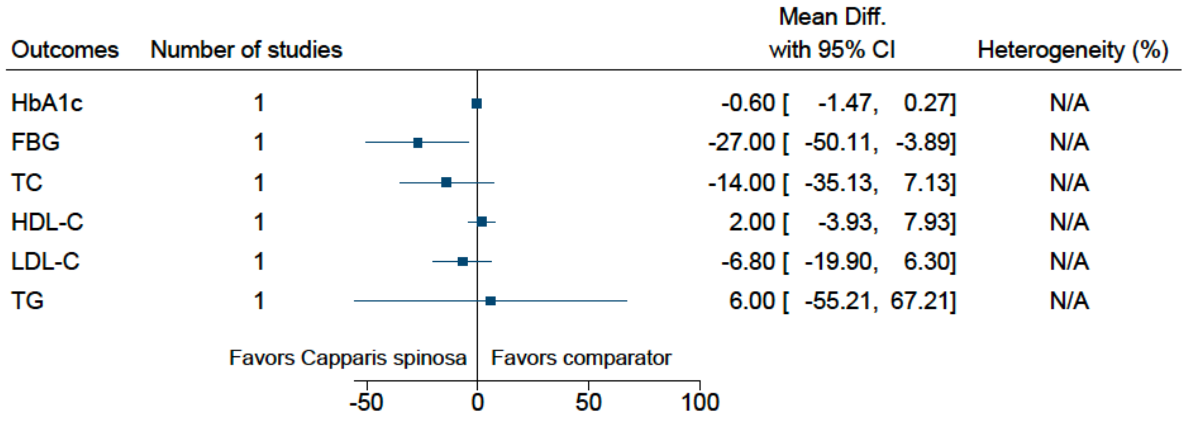

CardiPro (Fatima 2012)

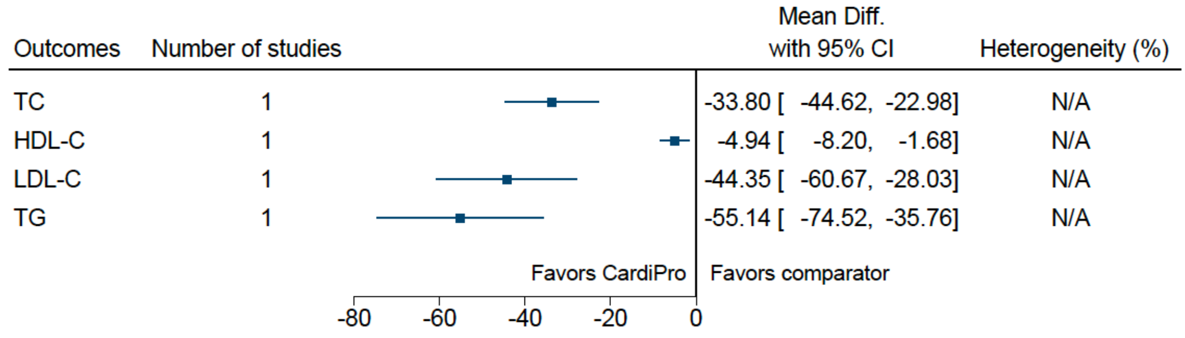

Cichorium intybus (Chandra 2020)

Outcomes	Number of studies	Mean Diff. with 95% CI	Heterogeneity (%)
TC	1	-4.60 [-25.29, 16.09]	N/A
HDL-C	1	-0.90 [-5.33, 3.53]	N/A
LDL-C	1	2.50 [-8.62, 13.62]	N/A
TG	1	-29.80 [-56.12, -3.48]	N/A

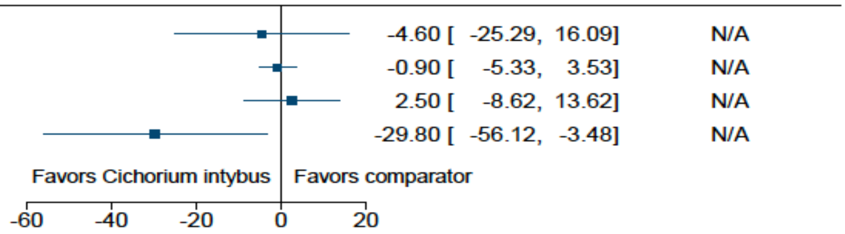

Cinnamomum verum versus *Crocus sativus* (Azimi 2014, Azimi 2016)

Outcomes	Number of studies	Mean Diff. with 95% CI	Heterogeneity (%)
HbA1c	1	0.13 [-0.09, 0.35]	N/A
FBG	1	1.59 [-20.99, 24.17]	N/A
Fasting insulin*	1	0.25 [-0.20, 0.70]	N/A
Body weight	1	-6.58 [-9.62, -3.54]	N/A
BMI	1	-0.16 [-0.71, 0.39]	N/A
Waist circumference	1	0.43 [-3.73, 4.59]	N/A
SBP	1	-6.00 [-6.43, -5.57]	N/A
DBP	1	-7.42 [-7.70, -7.14]	N/A
TC	1	2.70 [-5.26, 10.66]	N/A
HDL-C	1	-3.34 [-5.69, -0.99]	N/A
LDL-C	1	6.07 [-1.98, 14.12]	N/A
TG	1	43.74 [24.86, 62.62]	N/A

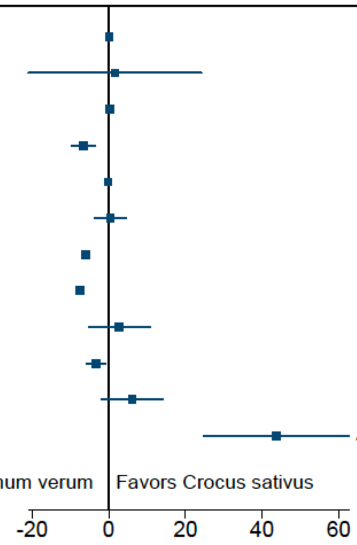

*Favors intervention/comparator as insulin sensitizer

Cinnamomum verum versus *Elettaria cardamomum* (Azimi 2014, Azimi 2016)

Outcomes	Number of studies	Mean Diff. with 95% CI	Heterogeneity (%)
HbA1c	1	0.00 [-0.27, 0.27]	N/A
FBG	1	-1.13 [-33.02, 30.76]	N/A
Fasting insulin*	1	0.08 [-0.44, 0.60]	N/A
Body weight	1	-3.18 [-6.51, 0.15]	N/A
BMI	1	-0.21 [-0.76, 0.34]	N/A
Waist circumference	1	1.38 [-2.64, 5.40]	N/A
SBP	1	-10.60 [-11.15, -10.05]	N/A
DBP	1	-8.90 [-9.18, -8.62]	N/A
TC	1	27.81 [10.07, 45.55]	N/A
HDL-C	1	-3.25 [-6.48, -0.02]	N/A
LDL-C	1	10.35 [-1.82, 22.52]	N/A
TG	1	32.36 [0.98, 63.74]	N/A

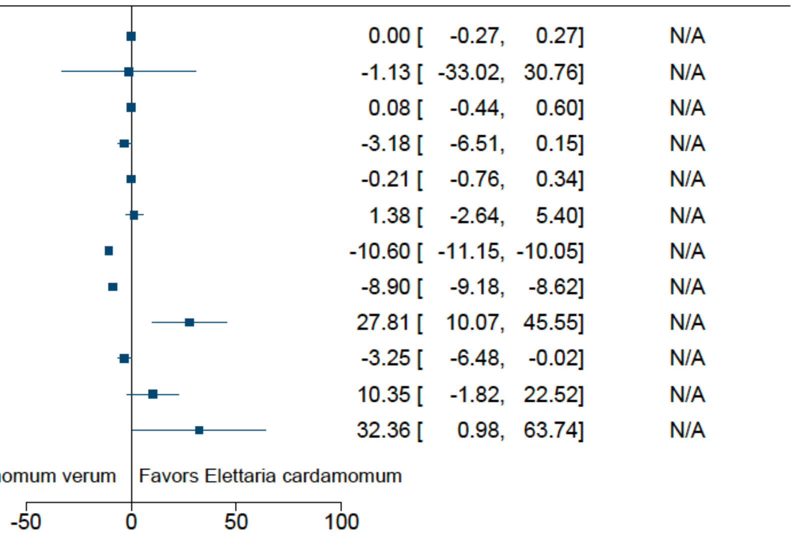

*Favors intervention/comparator as insulin sensitizer

Cinnamomum verum versus *Zingiber officinale* (Azimi 2014, Azimi 2016)

Outcomes	Number of studies	Mean Diff. with 95% CI	Heterogeneity (%)
HbA1c	1	-0.03 [-0.25, 0.19]	N/A
FBG	1	-8.09 [-34.59, 18.41]	N/A
Fasting insulin*	1	-0.36 [-0.86, 0.14]	N/A
Body weight	1	-3.89 [-6.89, -0.89]	N/A
BMI	1	-0.40 [-0.95, 0.15]	N/A
Waist circumference	1	-1.72 [-5.60, 2.16]	N/A
SBP	1	-9.07 [-9.62, -8.52]	N/A
DBP	1	-10.49 [-10.77, -10.21]	N/A
TC	1	0.76 [-7.85, 9.37]	N/A
HDL-C	1	-0.01 [-2.40, 2.38]	N/A
LDL-C	1	8.45 [-0.22, 17.12]	N/A
TG	1	36.76 [13.04, 60.48]	N/A

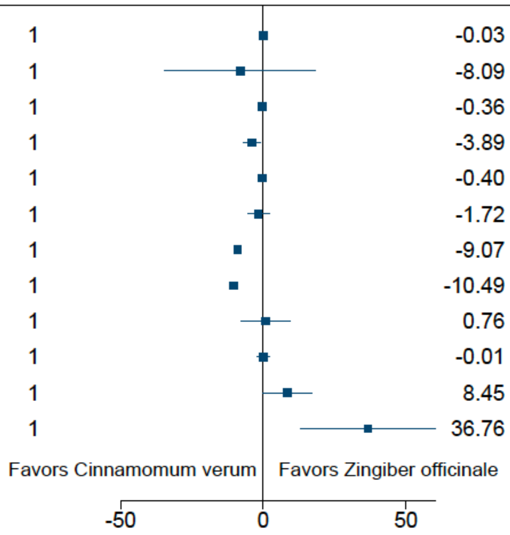

*Favors intervention/comparator as insulin sensitizer

Coccinia grandis (versus OAD) (Quamri 2017)

Outcomes	Number of studies	Mean Diff. with 95% CI	Heterogeneity (%)
TC	1	8.22 [-17.90, 34.34]	N/A
HDL-C	1	-1.83 [-7.61, 3.95]	N/A
LDL-C	1	65.53 [44.78, 86.28]	N/A
TG	1	5.33 [-36.55, 47.21]	N/A

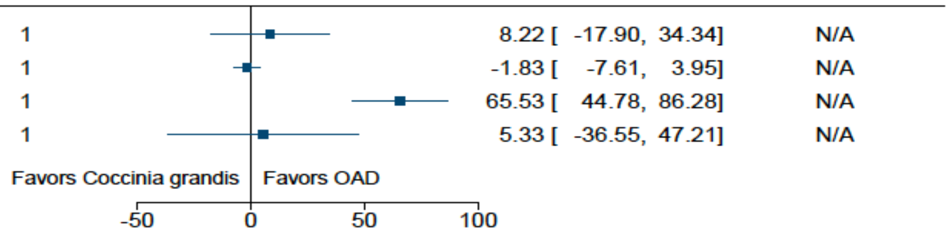

Cogent db (Shekhar 2002)

Outcomes	Number of studies	Mean Diff. with 95% CI	Heterogeneity (%)
HbA1c	1	-2.10 [-3.25, -0.95]	N/A
FBG	1	-63.06 [-97.10, -29.02]	N/A
PPBG	1	-115.30 [-155.69, -74.91]	N/A
Fasting insulin*	1	-2.90 [-7.56, 1.76]	N/A
Stimulated insulin*	1	-16.90 [-44.92, 11.12]	N/A
Fasting C-peptide*	1	-0.20 [-1.28, 0.88]	N/A
Stimulated C-peptide*	1	0.80 [-1.50, 3.10]	N/A
TC	1	-27.07 [-48.72, -5.41]	N/A
HDL-C	1	0.00 [-5.87, 5.87]	N/A
LDL-C	1	-11.60 [-36.34, 13.15]	N/A
TG	1	-44.25 [-89.26, 0.76]	N/A

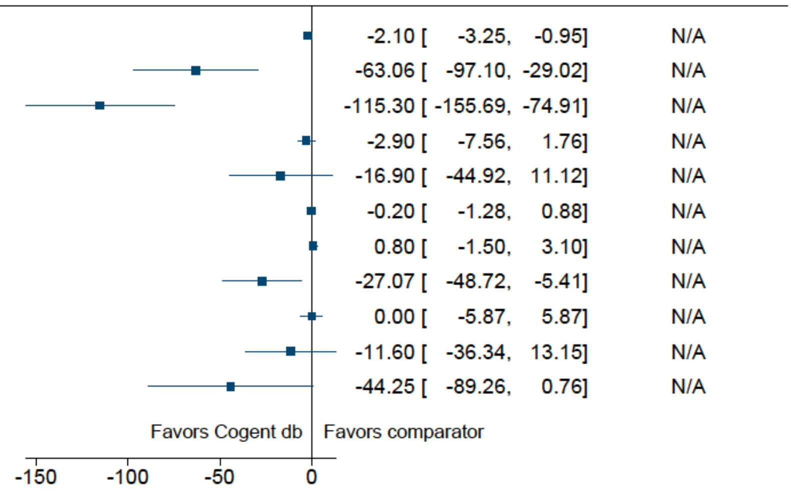

*Favors intervention/comparator as insulin sensitizer

Convolvulus prostratus (Patel 2012)

Outcomes	Number of studies	Mean Diff. with 95% CI	Heterogeneity (%)
FBG	1	30.02 [12.49, 47.55]	N/A
PPBG	1	53.15 [20.94, 85.36]	N/A

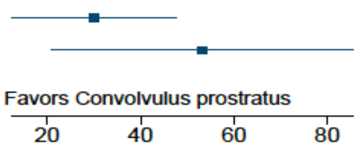

Crocus sativus versus *Elettaria cardamomum* (Azimi 2014, Azimi 2016)

Outcomes	Number of studies	Mean Diff. with 95% CI	Heterogeneity (%)
HbA1c	1	-0.13 [-0.37, 0.11]	N/A
FBG	1	-2.72 [-27.84, 22.40]	N/A
Fasting insulin*	1	-0.17 [-0.66, 0.32]	N/A
Body weight	1	3.40 [0.35, 6.45]	N/A
BMI	1	-0.05 [-0.61, 0.51]	N/A
Waist circumference	1	0.95 [-3.07, 4.97]	N/A
SBP	1	-4.60 [-5.04, -4.16]	N/A
DBP	1	-1.48 [-1.76, -1.20]	N/A
TC	1	25.11 [8.46, 41.76]	N/A
HDL-C	1	0.09 [-2.89, 3.07]	N/A
LDL-C	1	4.28 [-7.23, 15.79]	N/A
TG	1	-11.38 [-38.03, 15.27]	N/A

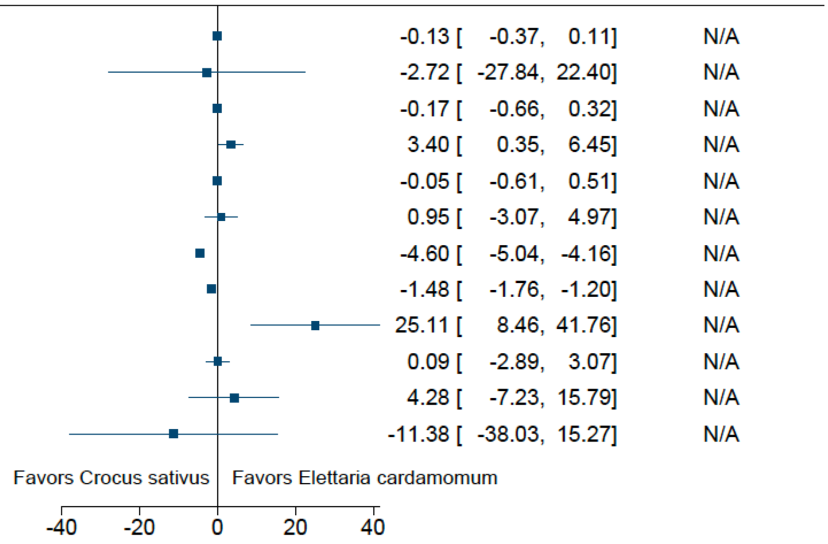

*Favors intervention/comparator as insulin sensitizer

Crocus sativus versus *Zingiber officinale* (Azimi 2014, Azimi 2016)

Outcomes	Number of studies	Mean Diff. with 95% CI	Heterogeneity (%)
HbA1c	1	-0.16 [-0.35, 0.03]	N/A
FBG	1	-9.68 [-27.65, 8.29]	N/A
Fasting insulin*	1	-0.61 [-1.08, -0.14]	N/A
Body weight	1	2.69 [-0.02, 5.40]	N/A
BMI	1	-0.24 [-0.80, 0.32]	N/A
Waist circumference	1	-2.15 [-6.05, 1.75]	N/A
SBP	1	-3.07 [-3.51, -2.63]	N/A
DBP	1	-3.07 [-3.35, -2.79]	N/A
TC	1	-1.94 [-8.83, 4.95]	N/A
HDL-C	1	3.33 [1.24, 5.42]	N/A
LDL-C	1	2.38 [-5.54, 10.30]	N/A
TG	1	-6.98 [-24.38, 10.42]	N/A

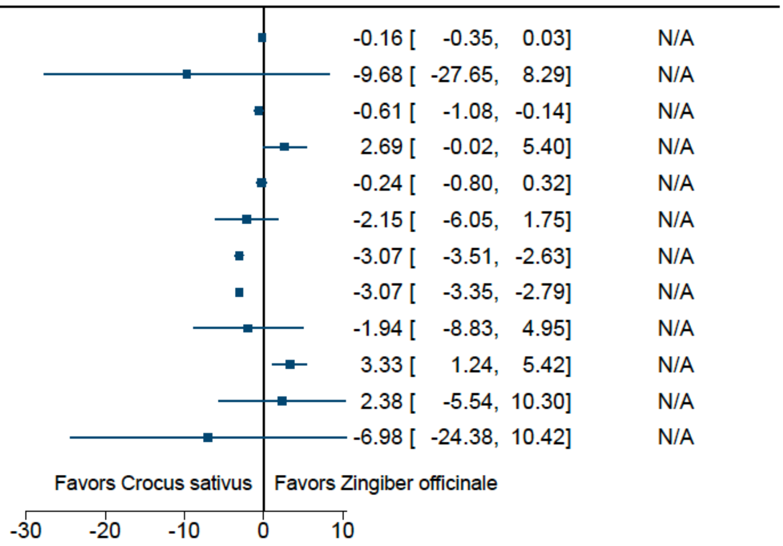

*Favors intervention/comparator as insulin sensitizer

Darvyadi Kwatha (Bhawana 2015)

Outcomes	Number of studies	Mean Diff. with 95% CI	Heterogeneity (%)
FBG	1	18.61 [-1.46, 38.68]	N/A
PPBG	1	17.13 [-12.88, 47.14]	N/A

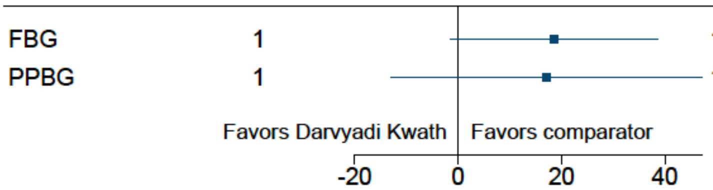

DCBT 2345 (Mohan 2001)

Outcomes	Number of studies	Mean Diff. with 95% CI	Heterogeneity (%)
HbA1c	1	0.10 [-1.11, 1.31]	N/A
FBG	1	4.00 [-32.27, 40.27]	N/A
PPBG	1	6.00 [-51.55, 63.55]	N/A
Fasting insulin*	1	4.40 [-1.03, 9.83]	N/A
Stimulated insulin*	1	8.00 [-9.60, 25.60]	N/A
Fasting C-peptide*	1	0.30 [-0.26, 0.86]	N/A
Stimulated C-peptide*	1	0.30 [-1.09, 1.69]	N/A

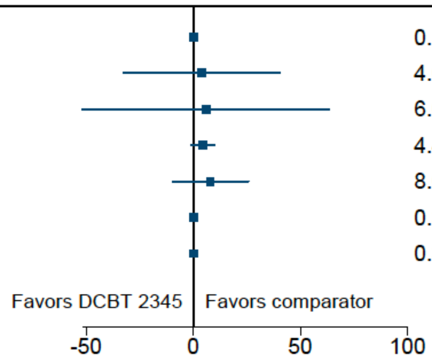

*Favors intervention/comparator as insulin sensitizer

Diabetea tea (Mahmoud 2016)

Outcomes	Number of studies	Mean Diff. with 95% CI	Heterogeneity (%)
HbA1c	1	0.56 [0.40, 0.72]	N/A
FBG	1	10.45 [5.71, 15.19]	N/A
TC	1	1.55 [-6.33, 9.43]	N/A
HDL-C	1	-2.32 [-4.70, 0.06]	N/A
LDL-C	1	3.48 [0.22, 6.74]	N/A
TG	1	30.97 [20.37, 41.57]	N/A

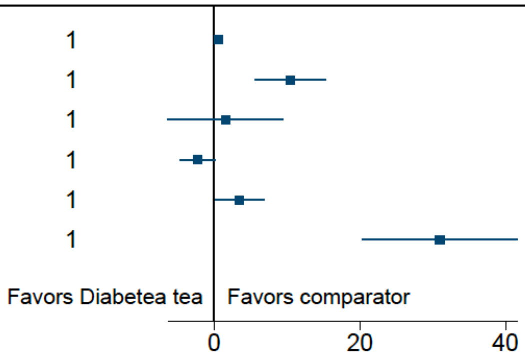

Eclipta prostrata (Sazia 2015a, Sazia 2015b)

Outcomes	Number of studies	Mean Diff. with 95% CI	Heterogeneity (%)
FBG	1	-4.65 [-17.28, 7.98]	N/A
PPBG	1	-8.06 [-28.13, 12.01]	N/A

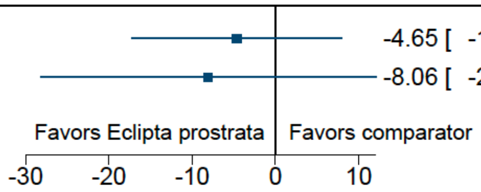

***Eclipta prostrata* (versus OAD) (Sazia 2015a, Sazia 2015b)**

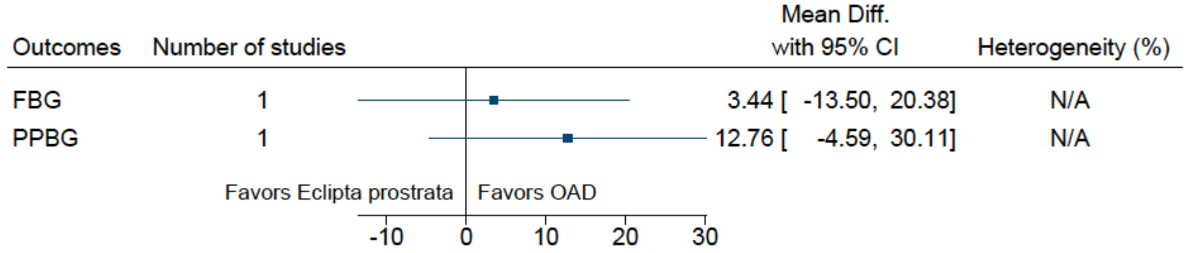

***Elettaria cardamomum* versus *Zingiber officinale* (Azimi 2014, Azimi 2016)**

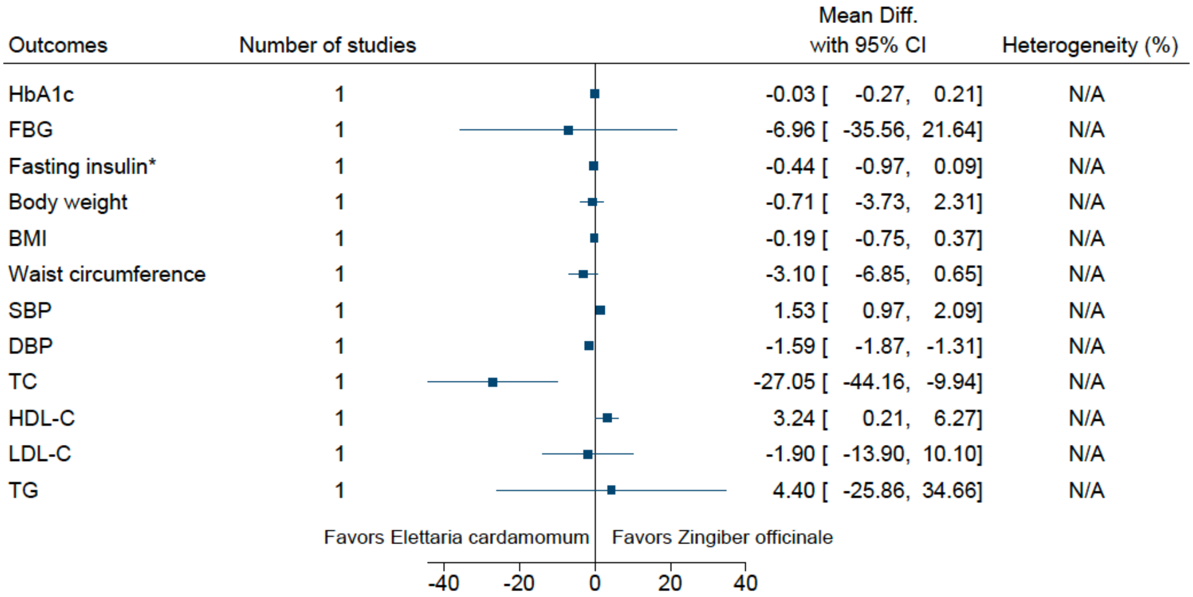

*Favors intervention/comparator as insulin sensitizer

***Emblica officinalis* (Usharani 2013)**

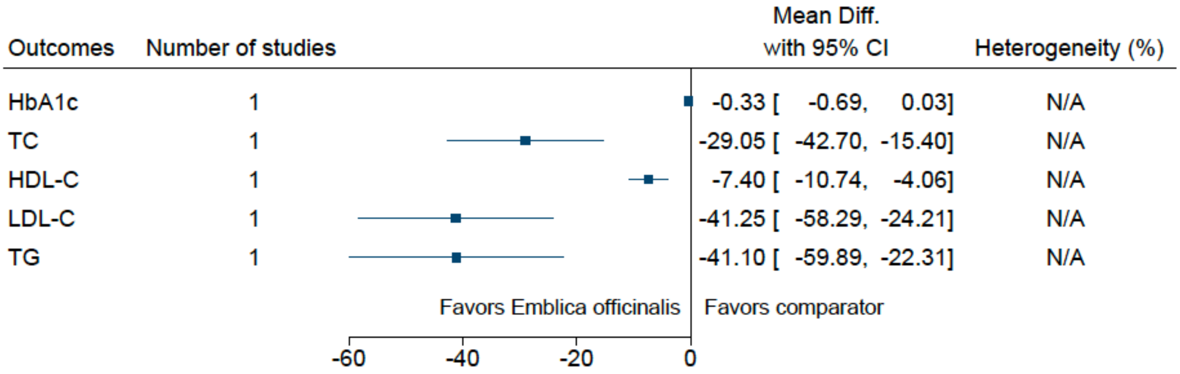

Emblica officinalis versus *Withania somnifera* (Usharani 2014)

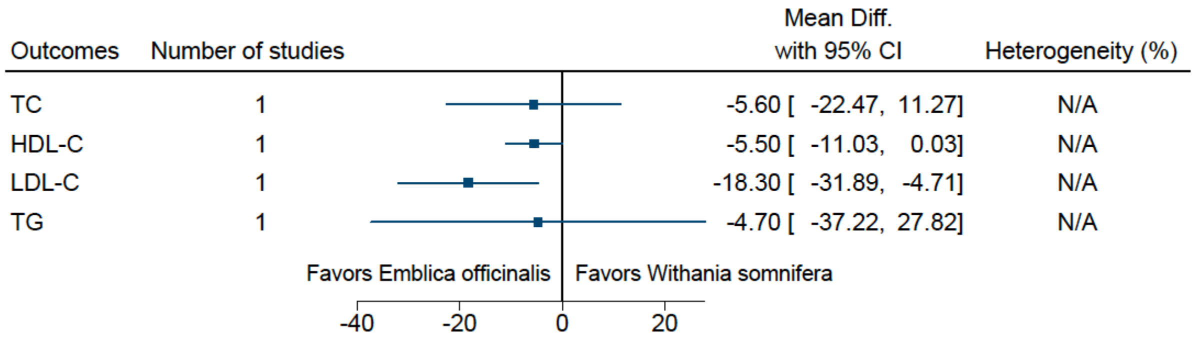

Emblica officinalis, *Withania somnifera* versus *Emblica officinalis* (Usharani 2014)

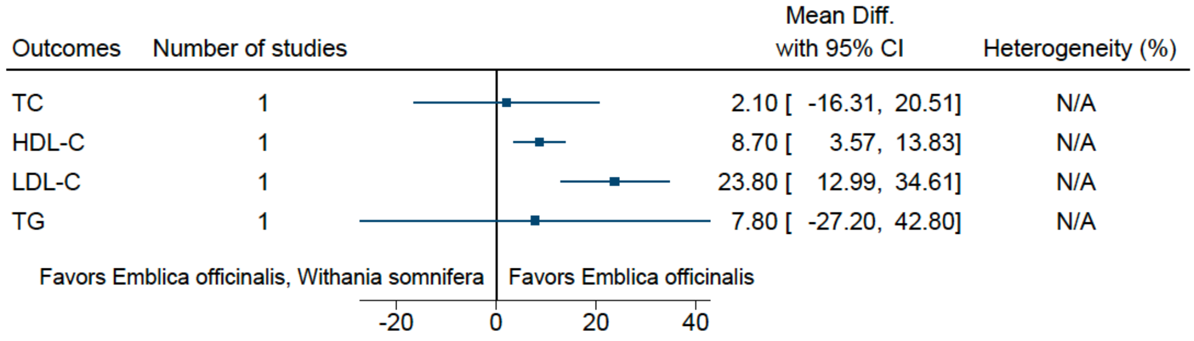

Emblica officinalis, *Withania somnifera* versus *Withania somnifera* (Usharani 2014)

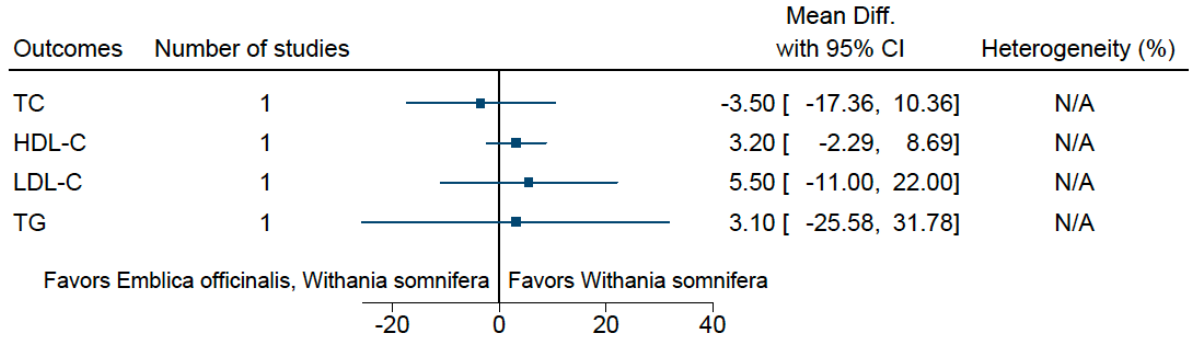

Enicostemma axillare versus Shilajit (Kumar 2014)

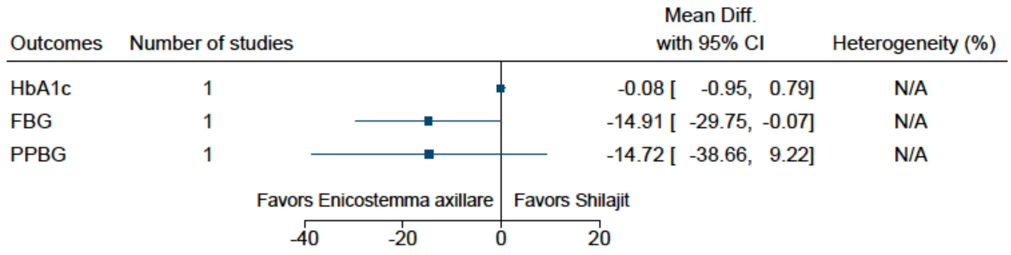

Enicostemma axillare+Emblica officinalis+Tinospora cordifolia (Sharma 2019)

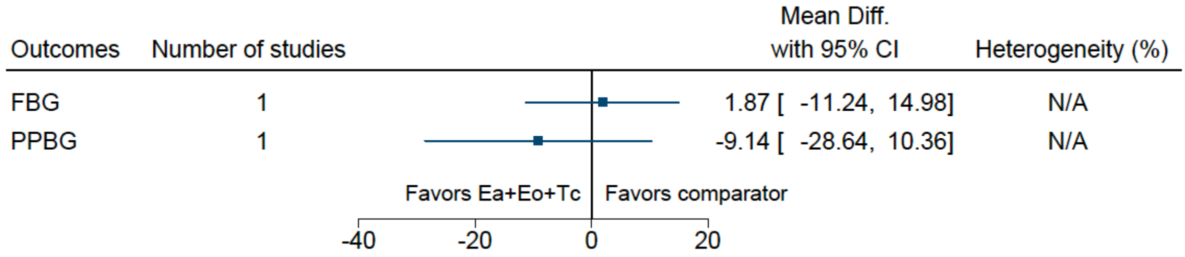

Herbal combination (Shokoohi 2017)

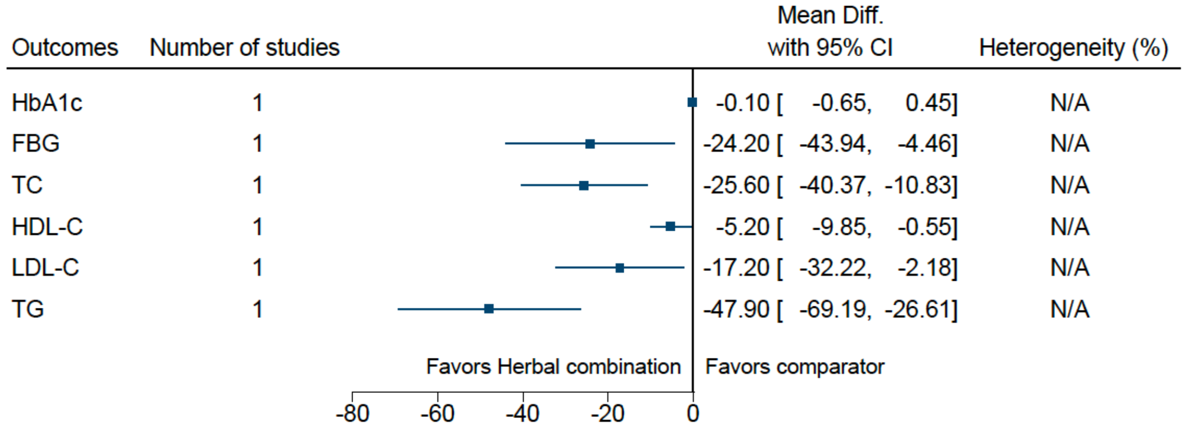

Hibiscus sabdariffa (Sarbini 2019)

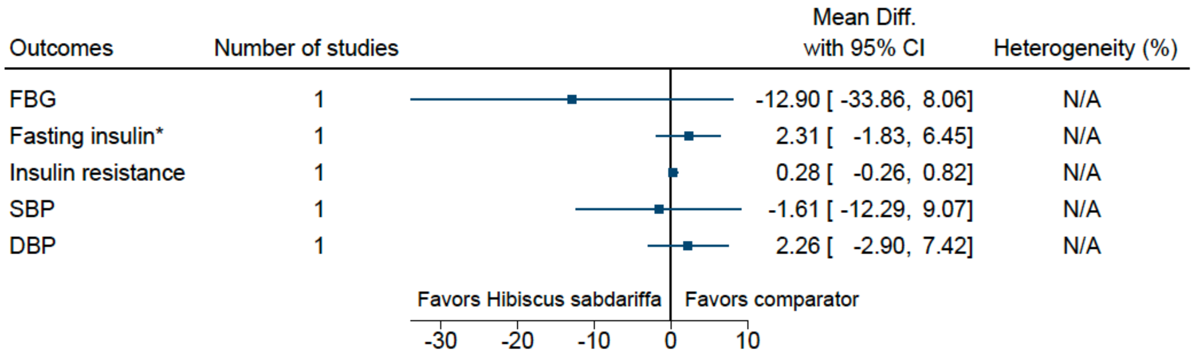

*Favors Intervention/comparator as insulin sensitizer

Hyponidd (Poongothai 2002)

Outcomes	Number of studies	Mean Diff. with 95% CI	Heterogeneity (%)
HbA1c	1	-0.50 [-1.54, 0.54]	N/A
FBG	1	-12.00 [-52.89, 28.89]	N/A
PPBG	1	-8.00 [-62.96, 46.96]	N/A
Fasting insulin*	1	1.10 [-3.29, 5.49]	N/A
Stimulated insulin*	1	3.30 [-8.39, 14.99]	N/A
Fasting C-peptide*	1	0.30 [-0.12, 0.72]	N/A
Stimulated C-peptide*	1	0.30 [-1.11, 1.71]	N/A
TC	1	-1.00 [-30.64, 28.64]	N/A
HDL-C	1	-2.00 [-7.27, 3.27]	N/A
LDL-C	1	6.00 [-18.20, 30.20]	N/A
TG	1	-32.00 [-149.32, 85.32]	N/A

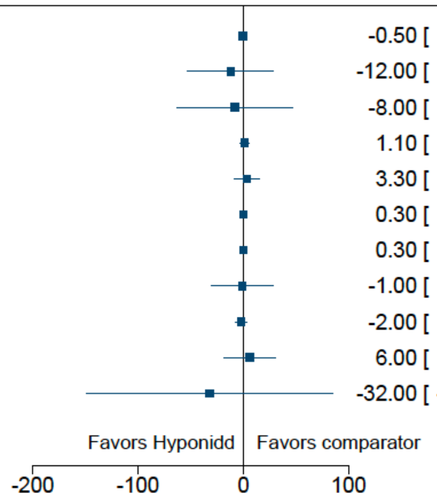

*Favors intervention/comparator as insulin sensitizer

Inolter (Agrawal 2002)

Outcomes	Number of studies	Mean Diff. with 95% CI	Heterogeneity (%)
HbA1c	1	-0.80 [-0.93, -0.67]	N/A
FBG	1	-26.50 [-62.48, 9.48]	N/A
TC	1	2.10 [-8.96, 13.16]	N/A
HDL-C	1	-3.90 [-5.71, -2.09]	N/A
LDL-C	1	-8.40 [-10.82, -5.98]	N/A
TG	1	-16.80 [-22.63, -10.97]	N/A

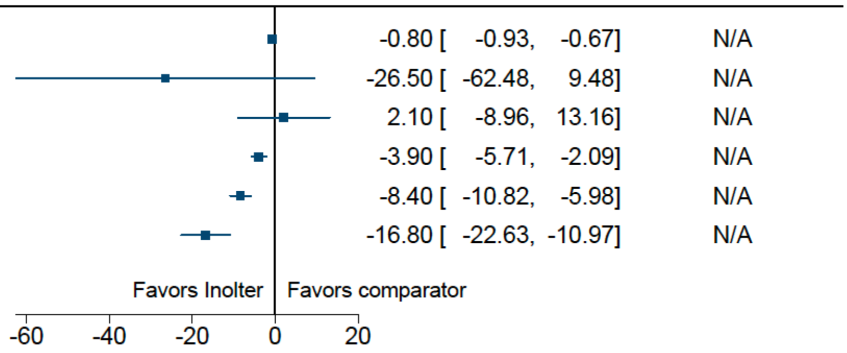

Kalpit (Agarwal 2013)

Outcomes	Number of studies	Mean Diff. with 95% CI	Heterogeneity (%)
FBG	1	-17.33 [-27.31, -7.35]	N/A
PPBG	1	-19.30 [-32.13, -6.47]	N/A
Body weight	1	-1.44 [-2.13, -0.75]	N/A
BMI	1	-0.75 [-1.07, -0.43]	N/A
Waist circumference	1	-3.98 [-5.20, -2.77]	N/A
TC	1	-26.20 [-35.82, -16.58]	N/A
HDL-C	1	-0.28 [-3.03, 2.47]	N/A
LDL-C	1	-21.31 [-30.77, -11.85]	N/A
TG	1	-34.49 [-44.51, -24.47]	N/A

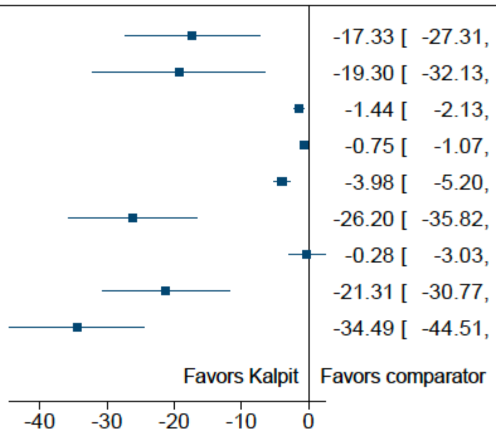

Kalpit (versus OAD) (Agarwal 2013)

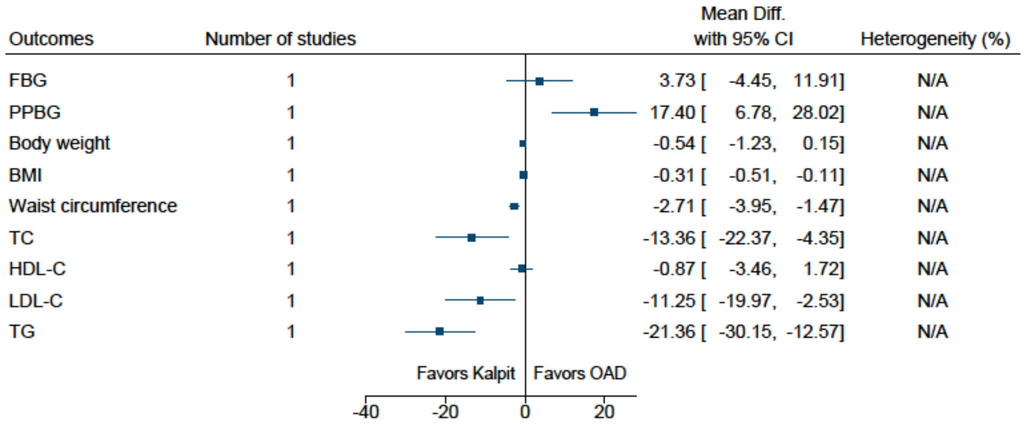

Khadira-Kramuka Kashaya Ghanavati (Paliwal 2018)

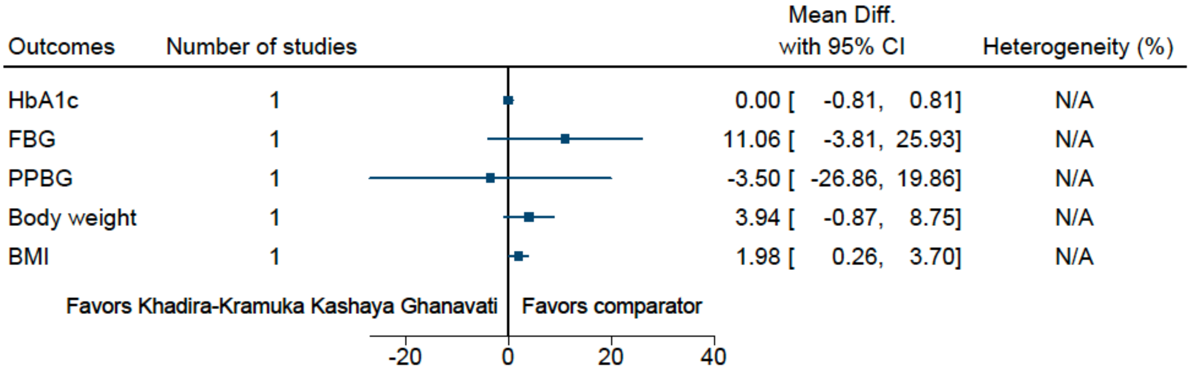

Khadira-Kramuka Kashaya Ghanavati versus Nishamalaki, Shilajit (Paliwal 2018)

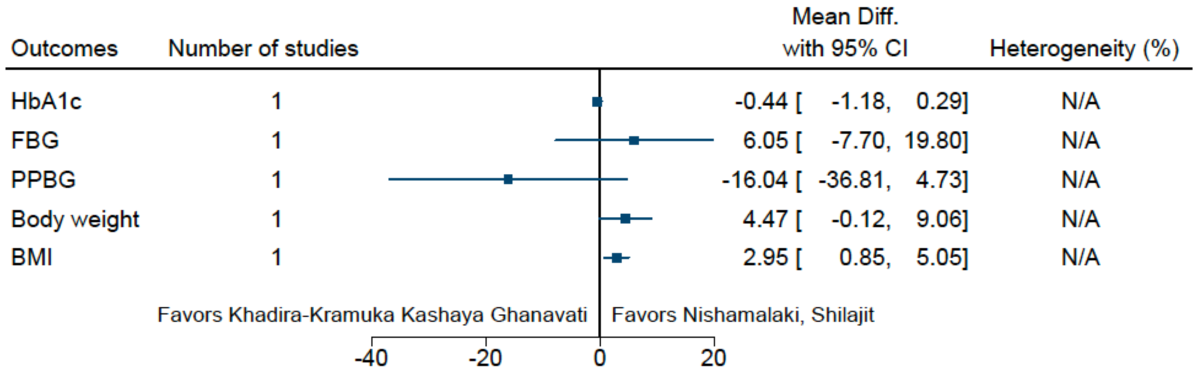

Kiratadi Churna (Kumari 2016)

Outcomes	Number of studies	Mean Diff. with 95% CI	Heterogeneity (%)
HbA1c	1	-0.40 [-1.62, 0.82]	N/A
FBG	1	-10.54 [-35.48, 14.40]	N/A
TC	1	-3.02 [-23.31, 17.27]	N/A
HDL-C	1	-1.51 [-3.92, 0.90]	N/A
LDL-C	1	-0.72 [-19.01, 17.57]	N/A
TG	1	5.09 [-16.78, 26.96]	N/A

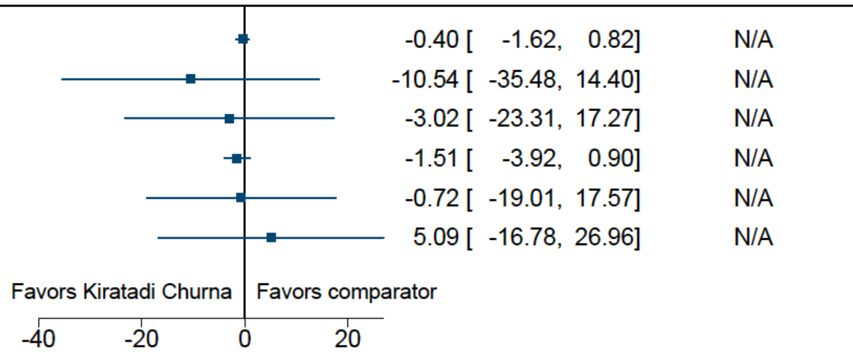

Linum usitatissimum (Barre 2008, Hashemzadeh 2017)

Outcomes	Number of studies	Mean Diff. with 95% CI	Heterogeneity (%)
HbA1c	1	0.00 [-1.13, 1.13]	N/A
FBG	1	9.01 [-42.87, 60.89]	N/A
Fasting insulin*	1	10.70 [-1.31, 22.71]	N/A
Body weight	1	-3.30 [-8.73, 2.13]	N/A
BMI	1	-1.00 [-2.88, 0.88]	N/A

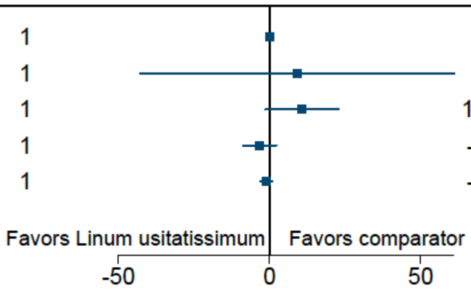

*Favors intervention/comparator as insulin sensitizer

Linum usitatissimum versus *Plantago ovata* (Ricklefs-Johnson 2017)

Outcomes	Number of studies	Mean Diff. with 95% CI	Heterogeneity (%)
HbA1c	1	-0.20 [-2.06, 1.66]	N/A
FBG	1	5.80 [-53.27, 64.87]	N/A
Fasting insulin*	1	-1.70 [-12.02, 8.62]	N/A
Insulin resistance	1	-0.40 [-5.55, 4.75]	N/A
Body weight	1	7.70 [-9.57, 24.97]	N/A
BMI	1	2.40 [-2.75, 7.55]	N/A
Waist circumference	1	3.38 [-12.24, 19.00]	N/A
SBP	1	0.80 [-10.66, 12.26]	N/A
DBP	1	-2.00 [-6.86, 2.86]	N/A
TC	1	-7.60 [-40.87, 25.67]	N/A
HDL-C	1	7.80 [-20.63, 36.23]	N/A
LDL-C	1	3.50 [-29.38, 36.38]	N/A
TG	1	13.00 [-91.27, 117.27]	N/A

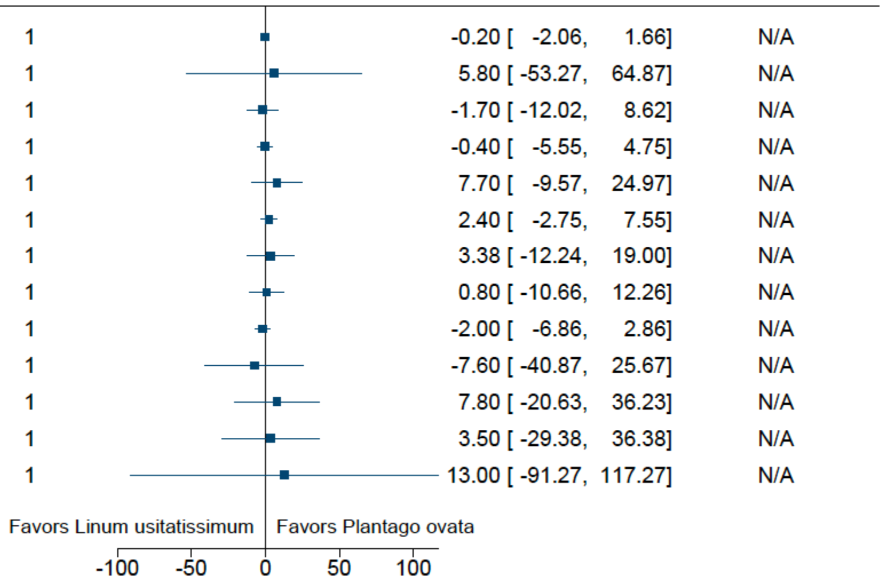

*Favors intervention/comparator as insulin sensitizer

Lodhradi Kashaya Ghana Vati (Bramhankar 2017)

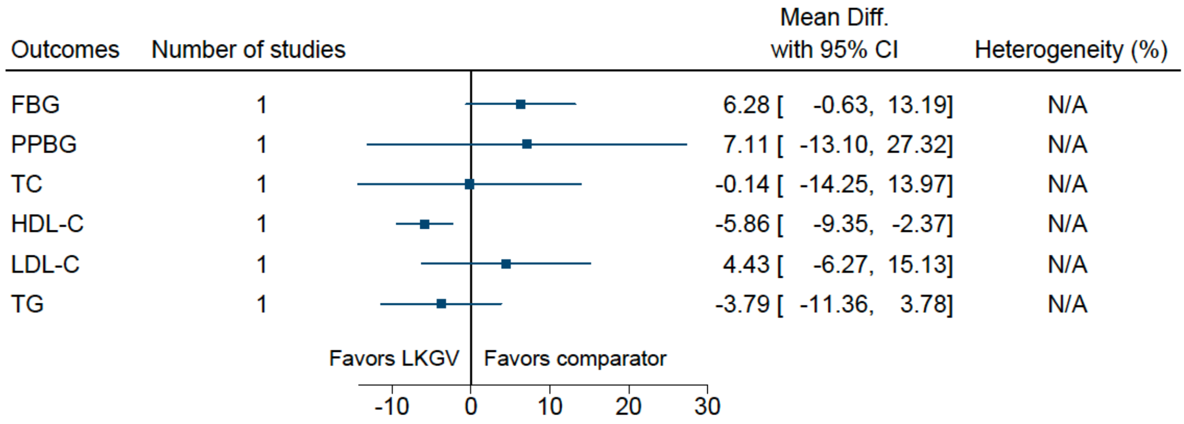

Lodhradi Kashaya Ghana Vati (versus OAD) (Bramhankar 2017)

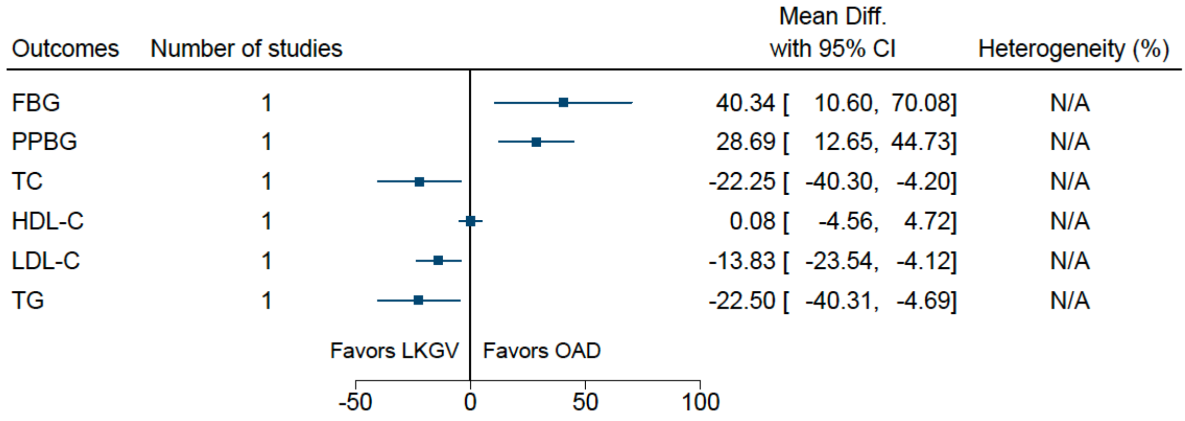

Madhumeha Nashini Gutika (Bhawana 2015)

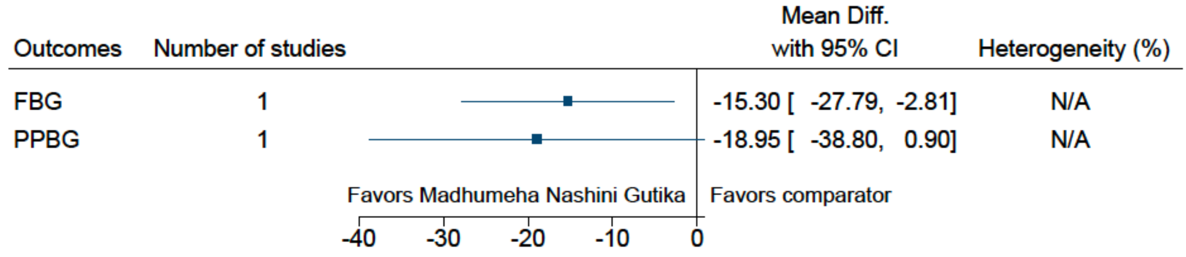

Madhumeha Nashini Gutika versus Darvyadi Kwatha (Bhawana 2015)

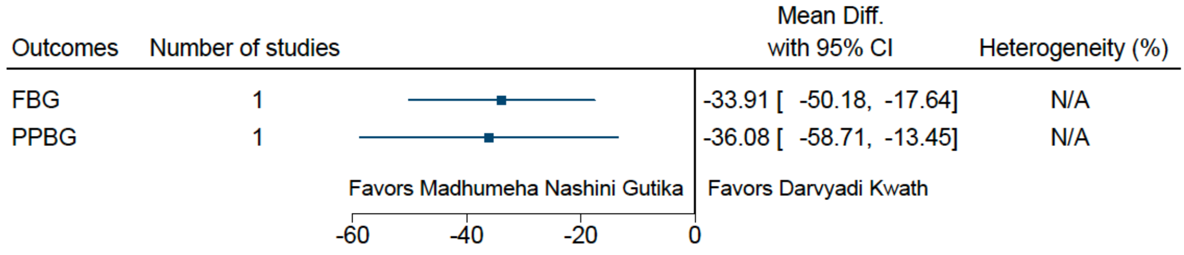

Mamajjaka Ghana Vati versus Tejashiladi Vati (Bhagat 2017)

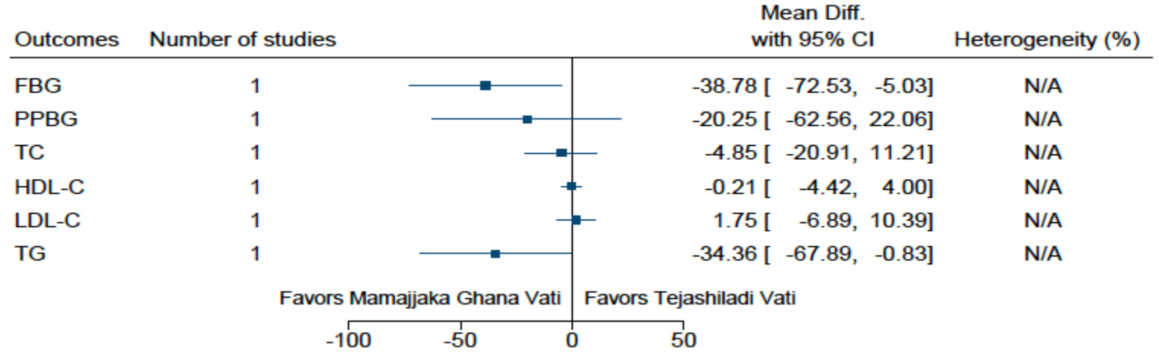

Mamajjaka Ghana Vati versus Trikatu Gutika (Kataria 2017)

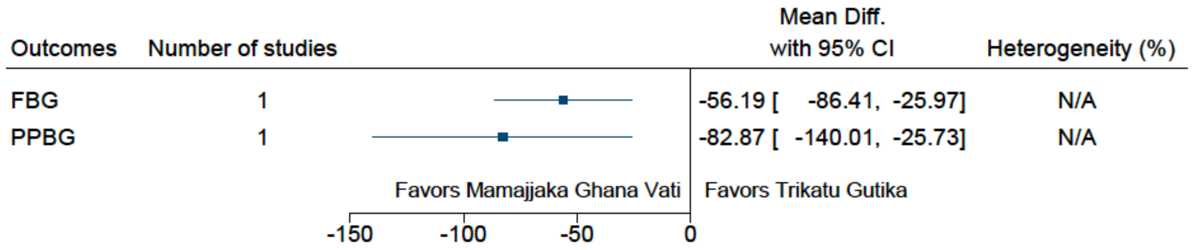

Mangifera indica (Balasubramaniam 2010)

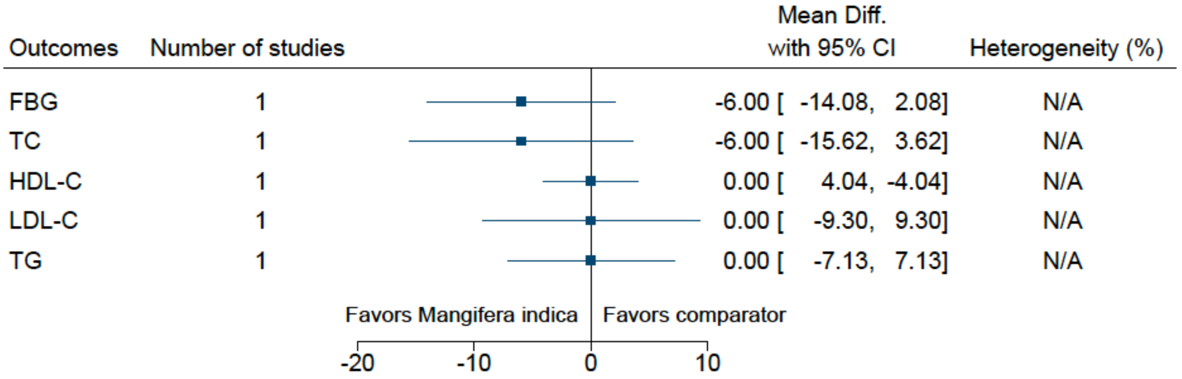

Mehagni (Gopalakrishna 2017)

Outcomes	Number of studies	Mean Diff. with 95% CI	Heterogeneity (%)
HbA1c	1	-0.35 [-1.25, 0.55]	N/A
FBG	1	-12.55 [-28.68, 3.58]	N/A
PPBG	1	-15.92 [-41.41, 9.57]	N/A
Body weight	1	-7.05 [-9.02, -5.08]	N/A
BMI	1	0.19 [-1.59, 1.97]	N/A
TC	1	-10.19 [-11.98, -8.40]	N/A
HDL-C	1	12.74 [9.30, 16.18]	N/A
LDL-C	1	-5.78 [-8.82, -2.74]	N/A
TG	1	-35.15 [-37.69, -32.61]	N/A

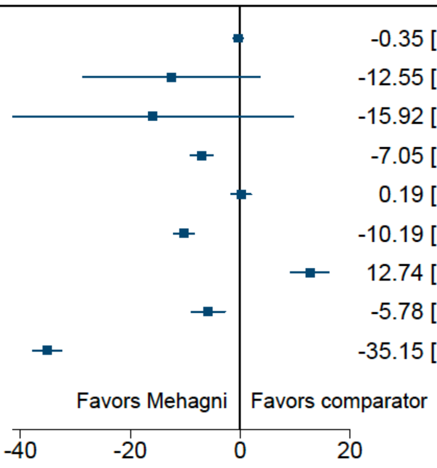

***Murraya koenigii* (Balasubramaniam 2010)**

Outcomes	Number of studies	Mean Diff. with 95% CI	Heterogeneity (%)
FBG	1	-5.00 [-10.90, 0.90]	N/A
TC	1	-2.00 [-10.99, 6.99]	N/A
HDL-C	1	-1.00 [-6.28, 4.28]	N/A
LDL-C	1	-3.00 [-11.37, 5.37]	N/A
TG	1	0.00 [-5.28, 5.28]	N/A

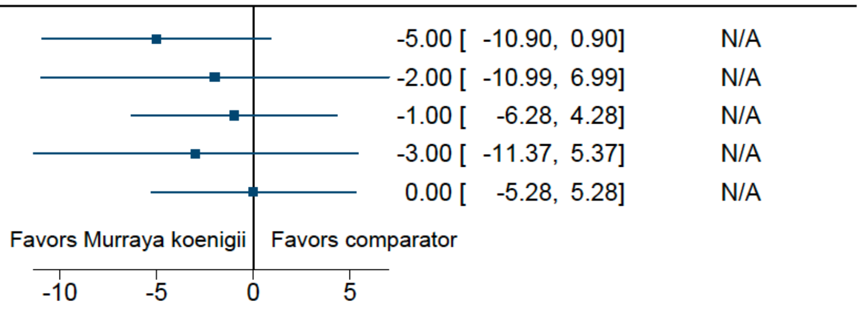

***Musa sapientum* (Balasubramaniam 2010)**

Outcomes	Number of studies	Mean Diff. with 95% CI	Heterogeneity (%)
FBG	1	-3.00 [-14.06, 8.06]	N/A
TC	1	0.00 [-8.99, 8.99]	N/A
HDL-C	1	0.00 [2.81, -2.81]	N/A
LDL-C	1	0.00 [-4.66, 4.66]	N/A
TG	1	2.00 [-5.80, 9.80]	N/A

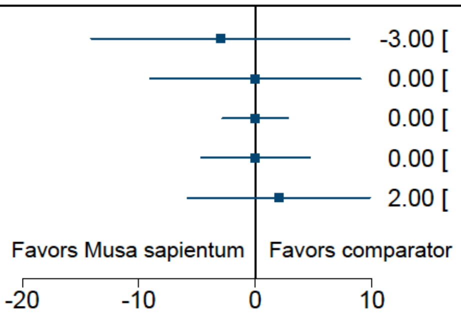

Mustadi Kwatha Ghana Vati (Kushwaha 2017)

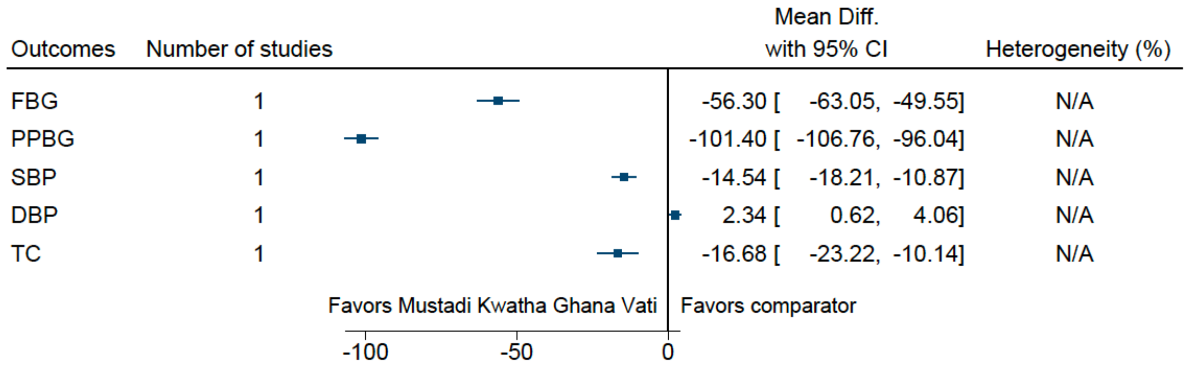

Naga Bhasma, Nishamalaki versus Nishamalaki, *Hordeum vulgare* (Desale 2018)

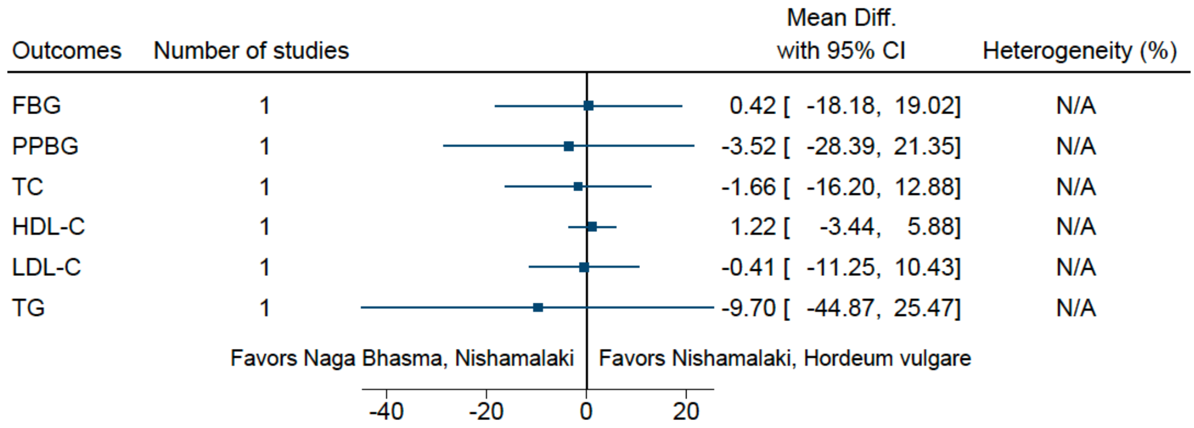

Nigella sativa (versus OAD) (Moustafa 2019)

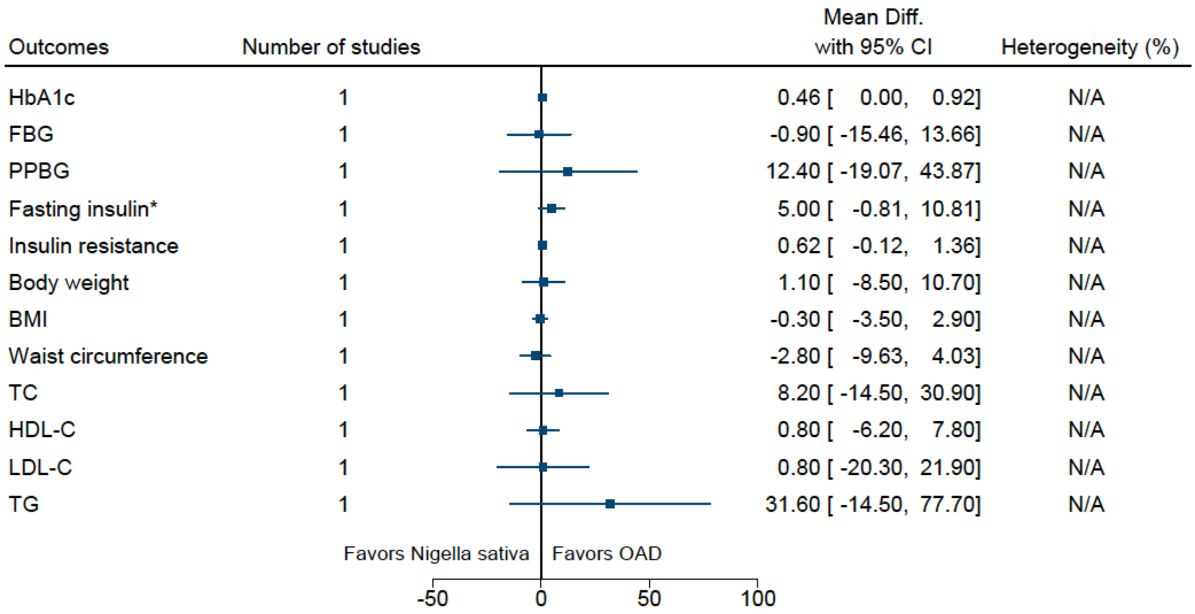

*Favors intervention/comparator as insulin sensitizer

***Nigella sativa*, *Trigonella foenum-graecum* (Memon 2010a, Memon 2010b, Memon 2012)**

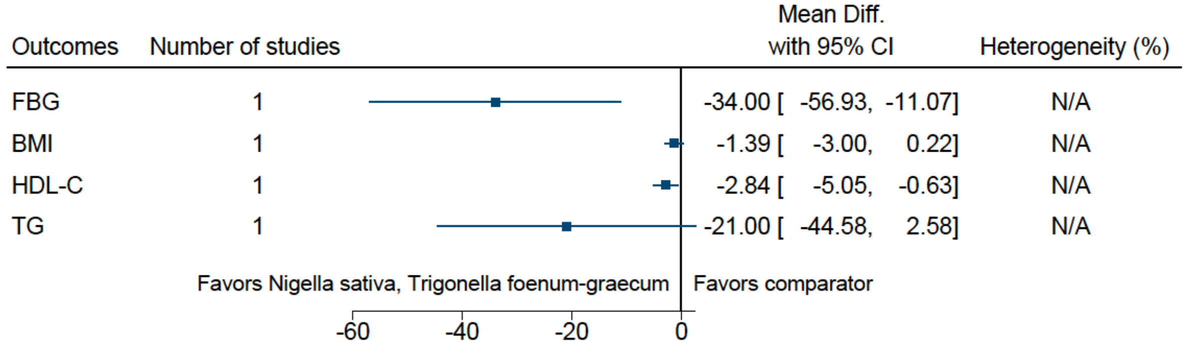

Nisha Katakadi Kashaya (Srinivas 2018)

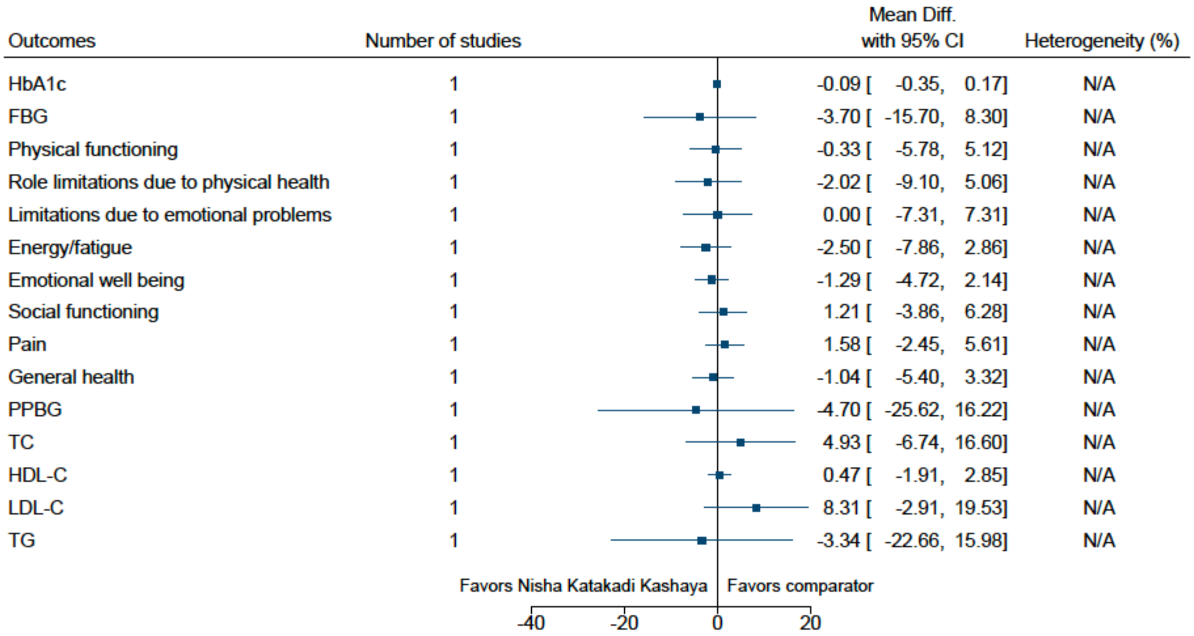

Nisha Katakadi Kashaya versus Yashad Bhasma (Srinivas 2018)

Outcomes	Number of studies	Mean Diff. with 95% CI	Heterogeneity (%)
HbA1c	1	1.06 [0.78, 1.34]	N/A
FBG	1	-0.60 [-12.93, 11.73]	N/A
Physical functioning	1	-0.80 [-6.32, 4.72]	N/A
Role limitations due to physical health	1	0.50 [-1.04, 2.04]	N/A
Limitations due to emotional problems	1	-3.54 [-10.81, 3.73]	N/A
Energy/fatigue	1	0.14 [-5.09, 5.37]	N/A
Emotional well being	1	-2.63 [-6.29, 1.03]	N/A
Social functioning	1	-1.95 [-4.59, 0.69]	N/A
Pain	1	1.70 [-2.61, 6.01]	N/A
General health	1	2.07 [-2.39, 6.53]	N/A
PPBG	1	-3.50 [-26.46, 19.46]	N/A
TC	1	15.74 [3.82, 27.66]	N/A
HDL-C	1	-1.40 [-4.06, 1.26]	N/A
LDL-C	1	13.35 [2.80, 23.90]	N/A
TG	1	0.21 [-21.44, 21.86]	N/A

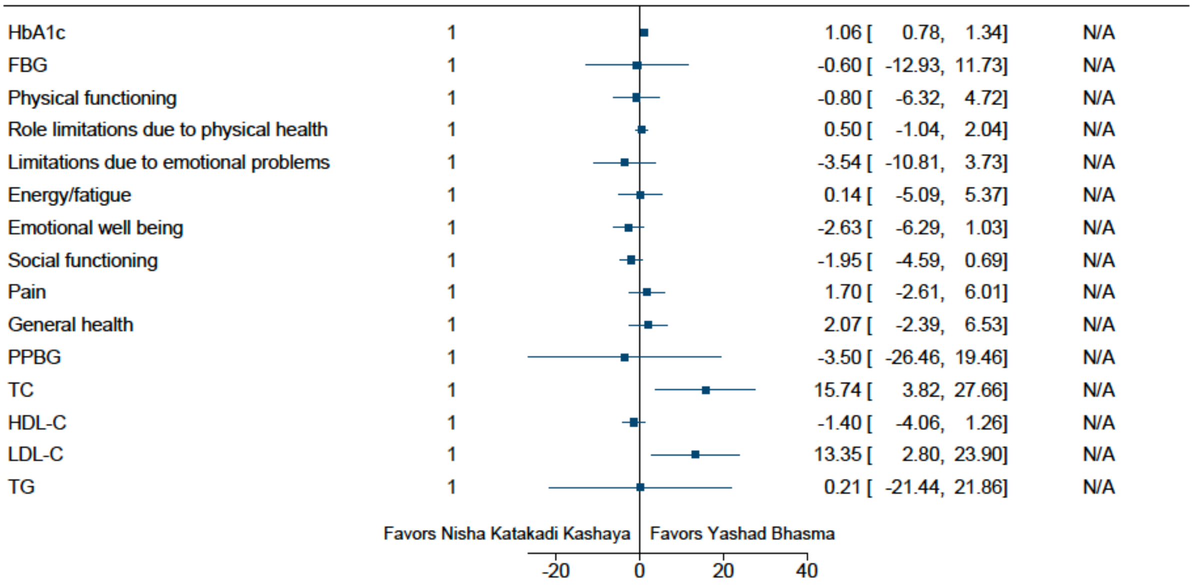

Nishamalaki (Samagandi 2012)

Outcomes	Number of studies	Mean Diff. with 95% CI	Heterogeneity (%)
FBG	1	-18.20 [-31.93, -4.47]	N/A
PPBG	1	-25.40 [-47.22, -3.58]	N/A

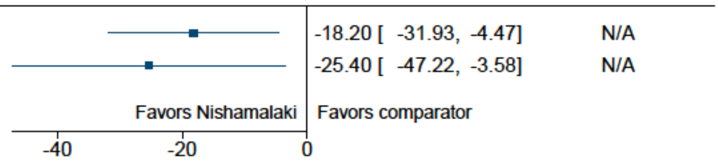

Nishamalaki, Shilajit (Paliwal 2018)

Outcomes	Number of studies	Mean Diff. with 95% CI	Heterogeneity (%)
HbA1c	1	0.44 [-0.39, 1.28]	N/A
FBG	1	5.01 [-12.04, 22.06]	N/A
PPBG	1	12.54 [-14.43, 39.51]	N/A
Body weight	1	-0.53 [-6.30, 5.24]	N/A
BMI	1	-0.97 [-3.51, 1.57]	N/A

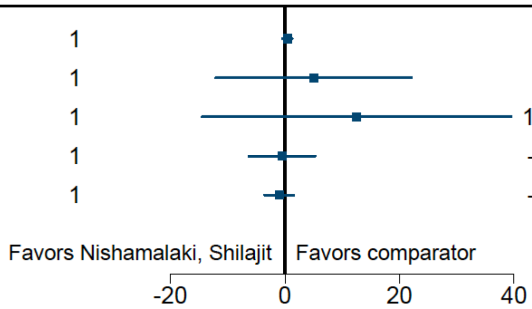

Ocimum tenuiflorum (Balasubramaniam 2010)

Outcomes	Number of studies	Mean Diff. with 95% CI	Heterogeneity (%)
FBG	1	-3.00 [-13.34, 7.34]	N/A
TC	1	-5.00 [-13.99, 3.99]	N/A
HDL-C	1	-2.00 [-4.19, 0.19]	N/A
LDL-C	1	-6.00 [-16.34, 4.34]	N/A
TG	1	-3.00 [-14.31, 8.31]	N/A

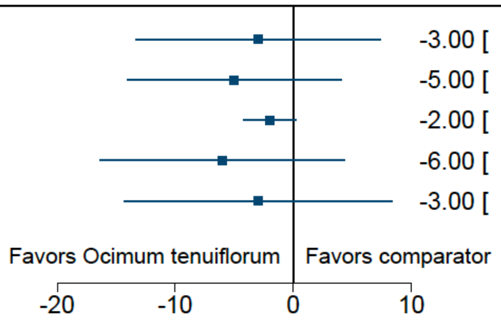

Pancreas tonic (Hsia 2004)

Outcomes	Number of studies	Mean Diff. with 95% CI	Heterogeneity (%)
HbA1c	1	-0.36 [-1.65, 0.93]	N/A

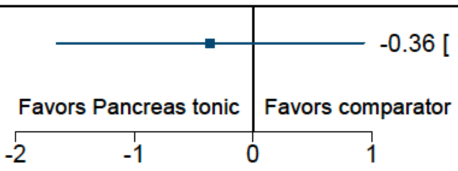

Phyllanthus amarus (Balasubramaniam 2010)

Outcomes	Number of studies	Mean Diff. with 95% CI	Heterogeneity (%)
FBG	1	-6.00 [-12.85, 0.85]	N/A
TC	1	-7.00 [-18.16, 4.16]	N/A
HDL-C	1	0.00 [2.81, -2.81]	N/A
LDL-C	1	0.00 [-9.38, 9.38]	N/A
TG	1	-8.00 [-16.70, 0.70]	N/A

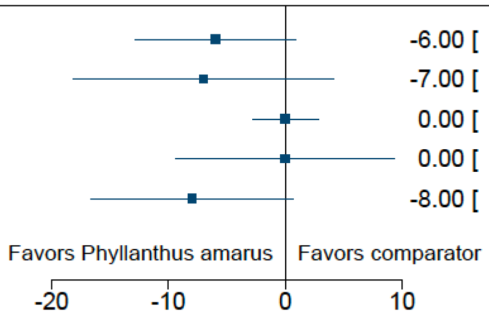

Polyherbal formulation (Awasthi 2015)

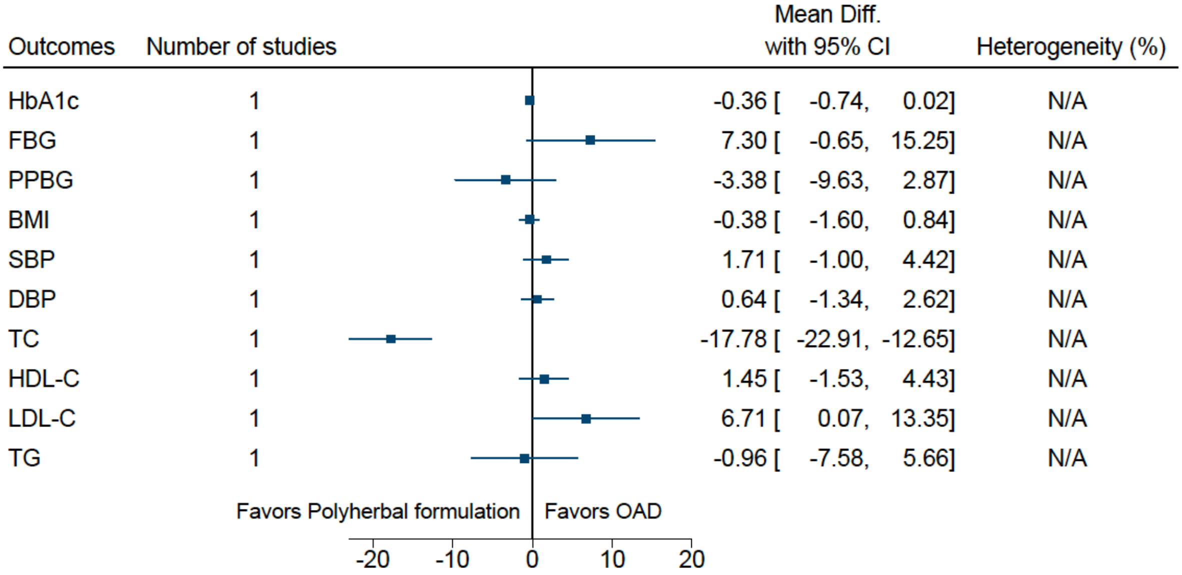

Portulaca oleracea **(versus OAD) (El-Sayed 2011)**

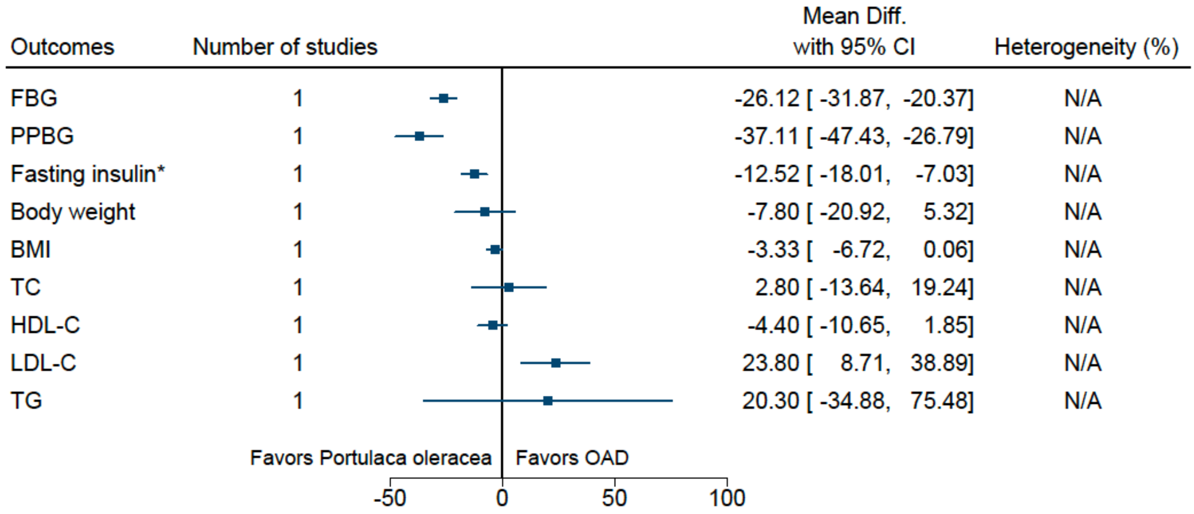

*Favors intervention/comparator as insulin sensitizer

Salasaradi Kashaya+Shilajit+Trivanga Bhasma+*Tinospora cordifolia*, *Azadirachta indica* versus Hyponidd (Bhat 2012)

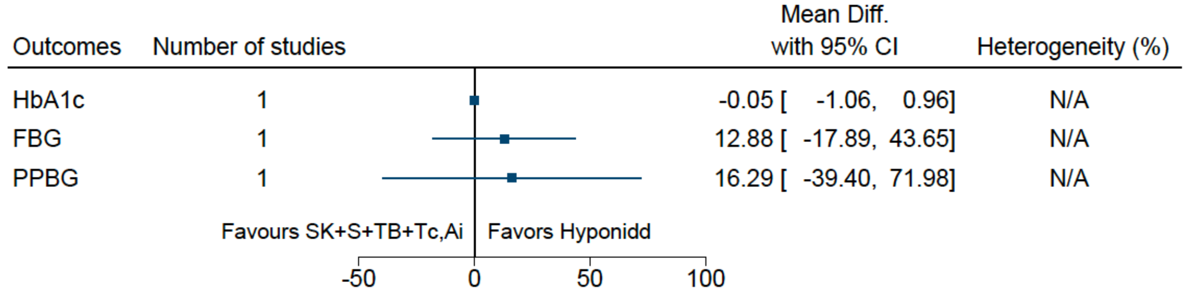

Shilajit (versus OAD) (Kumar 2014)

Outcomes	Number of studies	Mean Diff. with 95% CI	Heterogeneity (%)
HbA1c	1	-0.35 [-1.43, 0.73]	N/A
FBG	1	16.34 [3.95, 28.73]	N/A
PPBG	1	46.45 [26.47, 66.43]	N/A

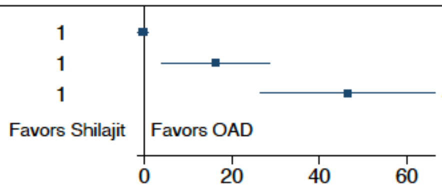

Swarnamakshika Bhasma (Taviad 2016)

Outcomes	Number of studies	Mean Diff. with 95% CI	Heterogeneity (%)
FBG	1	-4.22 [-20.29, 11.85]	N/A
PPBG	1	-13.94 [-40.96, 13.08]	N/A
TC	1	-8.50 [-22.25, 5.25]	N/A
HDL-C	1	1.77 [-1.89, 5.43]	N/A
TG	1	-23.40 [-59.49, 12.69]	N/A

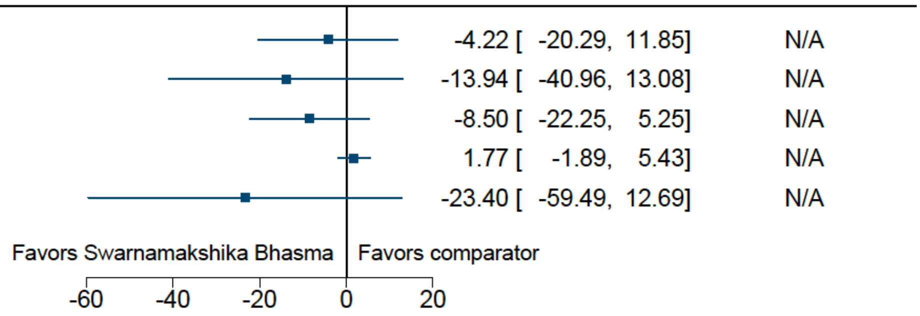

***Syzygium cumini* (versus OAD) (Sahana 2010)**

Outcomes	Number of studies	Mean Diff. with 95% CI	Heterogeneity (%)
HbA1c	1	0.20 [-1.24, 1.64]	N/A
FBG	1	11.90 [-13.11, 36.91]	N/A
PPBG	1	31.60 [-18.96, 82.16]	N/A
Insulin resistance	1	0.90 [-2.80, 4.60]	N/A
HDL-C	1	3.80 [-3.15, 10.75]	N/A

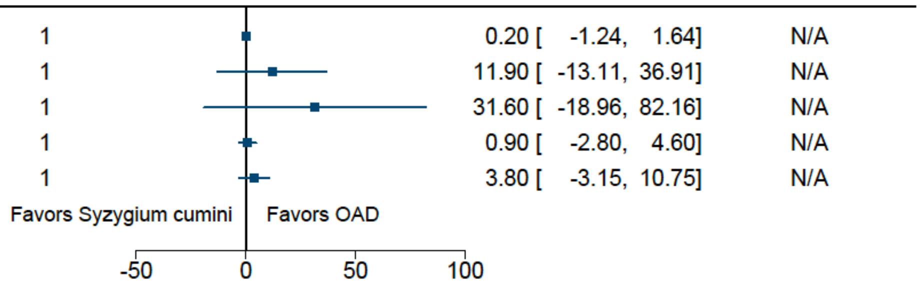

***Syzygium cumini+Withania coagulans* (Siddiqui 2017)**

Outcomes	Number of studies	Mean Diff. with 95% CI	Heterogeneity (%)
HbA1c	1	-0.23 [-0.61, 0.15]	N/A
FBG	1	4.43 [-7.07, 15.93]	N/A
PPBG	1	-5.63 [-26.97, 15.71]	N/A
TC	1	-1.47 [-20.91, 17.97]	N/A
TG	1	5.00 [-6.93, 16.93]	N/A

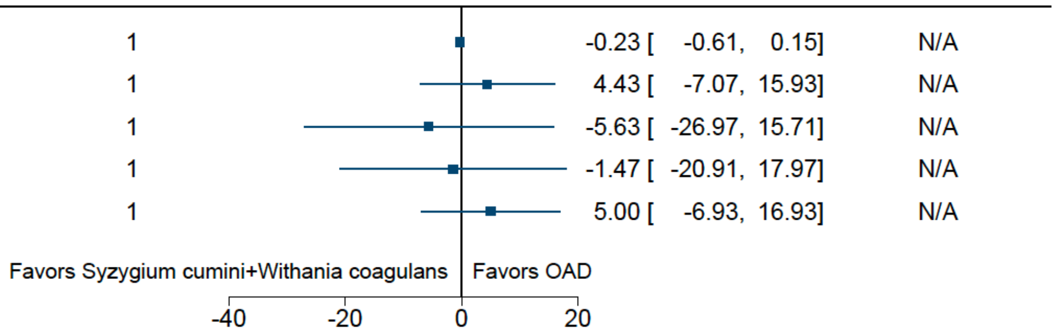

Talapotaka Churna (Nille 2018)

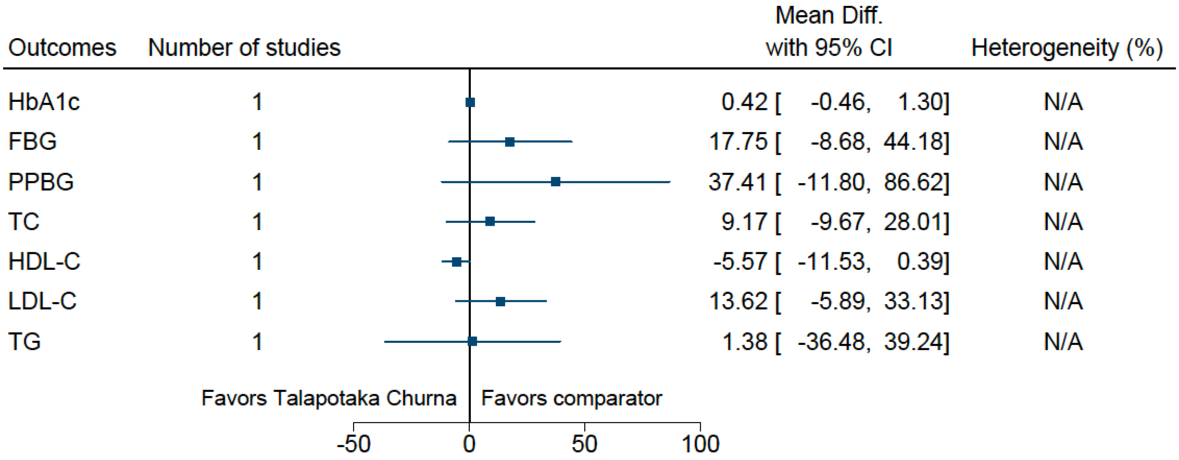

Talapotaka Churna (versus OAD) (Nille 2018)

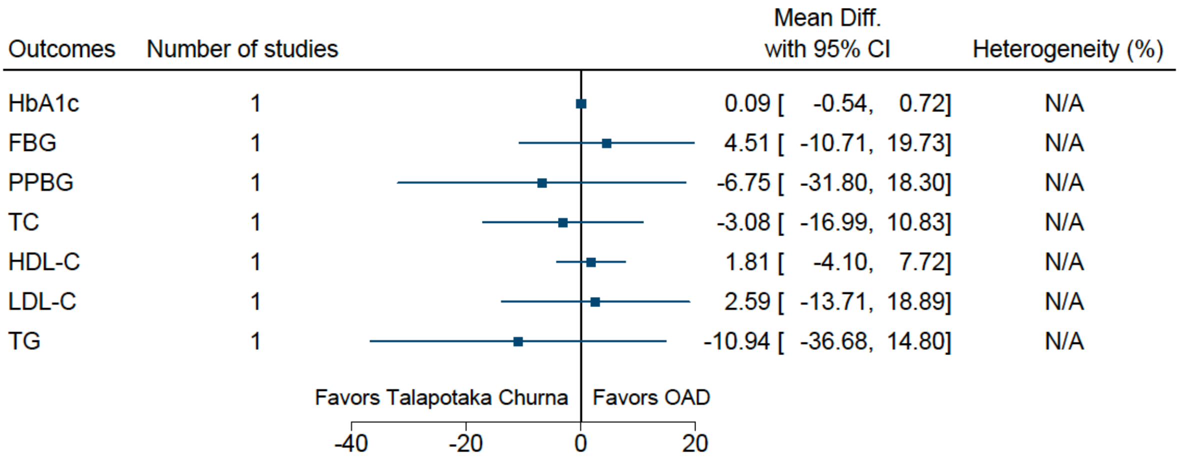

***Terminalia chebula* (Usharani 2020)**

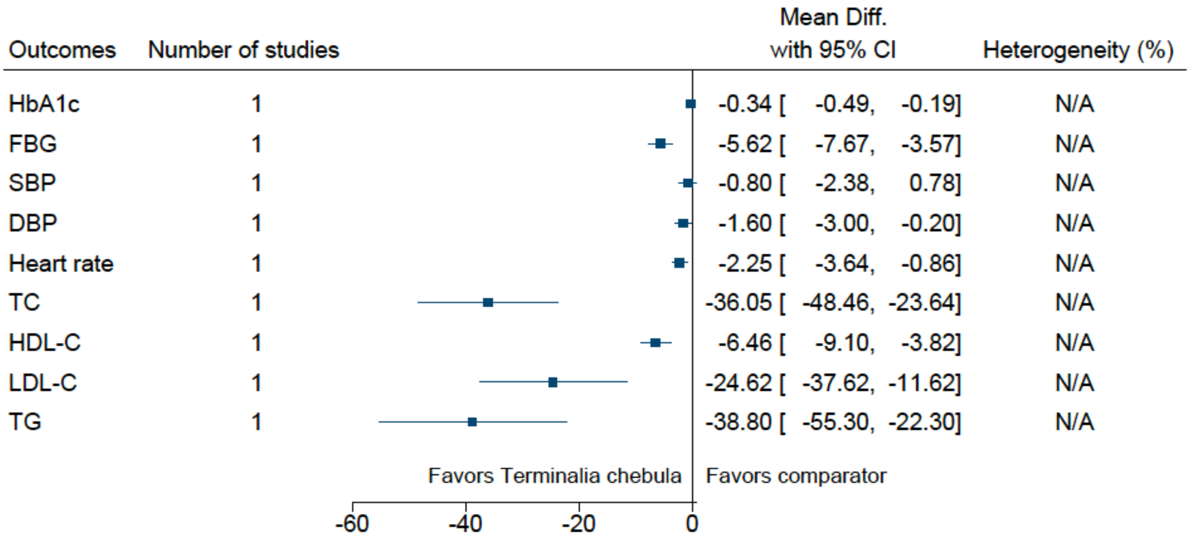

***Tribulus terrestris* (Samani 2016)**

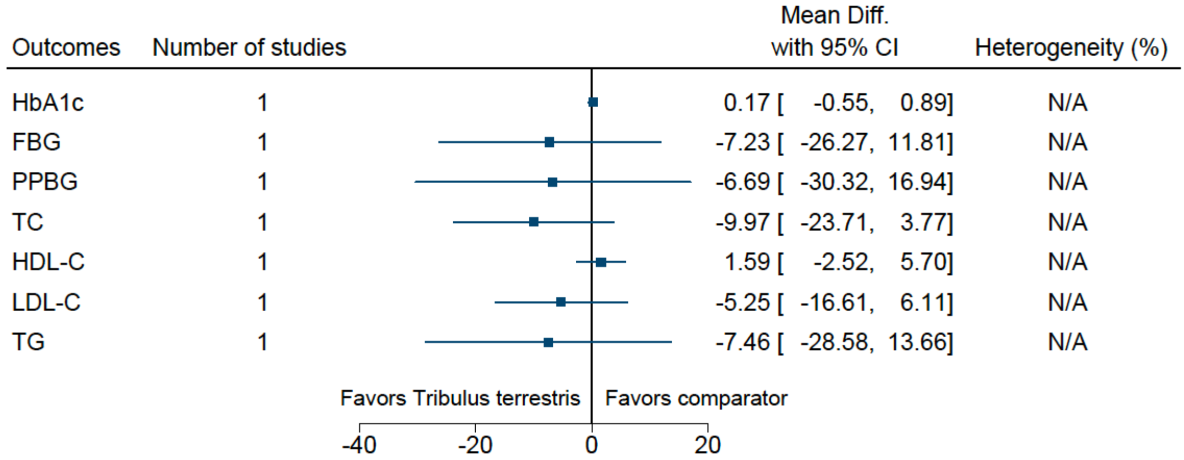

***Trigonella foenum-graecum*, *Ocimum tenuiflorum* (Mitra 2006)**

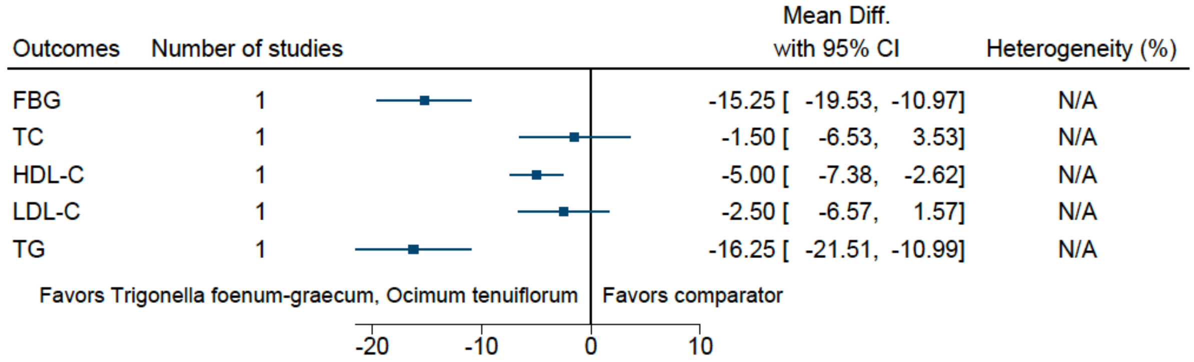

***Trigonella foenum-graecum* versus *Aegle marmelos* (Yaheya 2009)**

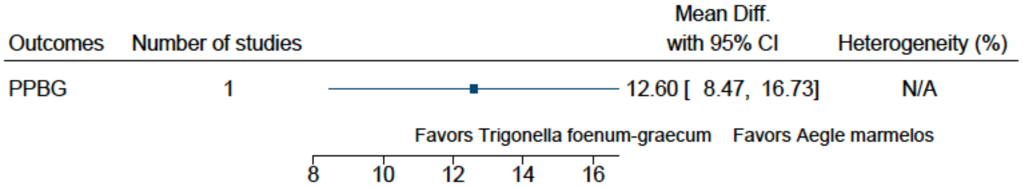

***Trigonella foenum-graecum+Aegle marmelos* (Yaheya 2009)**

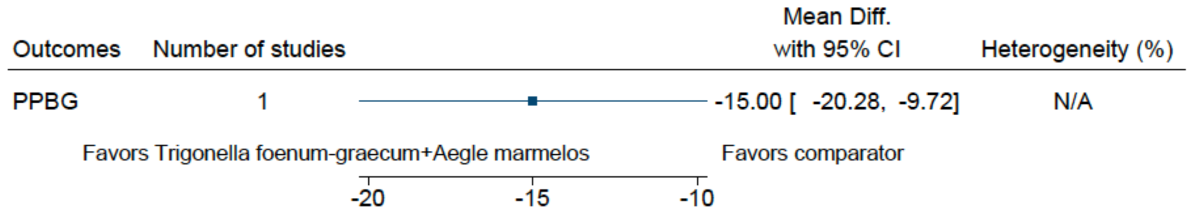

Triticum aestivum (Samagandi 2012)

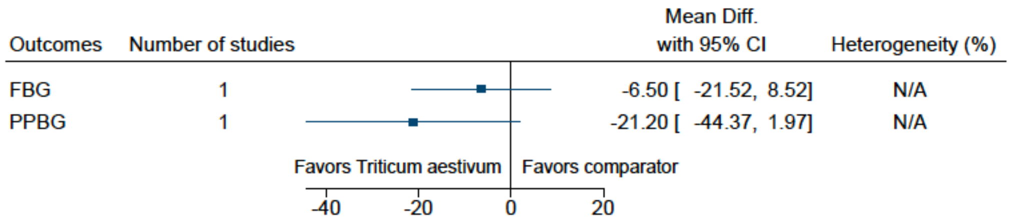

Triticum aestivum versus Nishamalaki (Samagandi 2012)

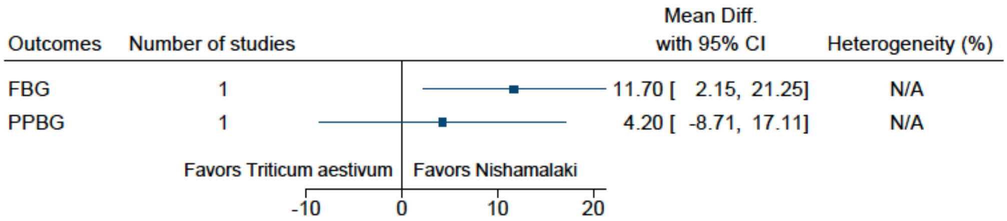

Vernonia cinerea (Sayeed 2013)

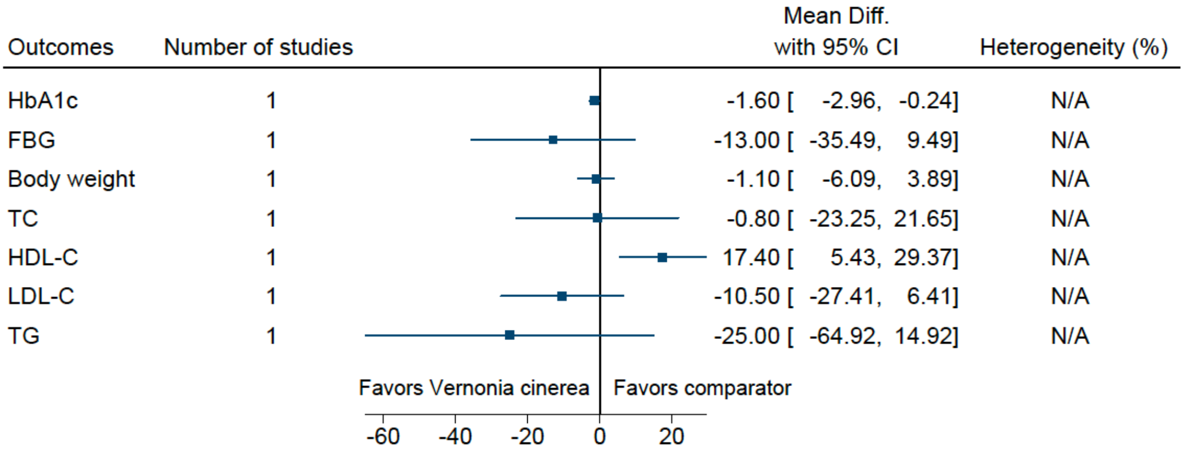

Vidangadi Yoga (versus OAD) (Deshpande 2018)

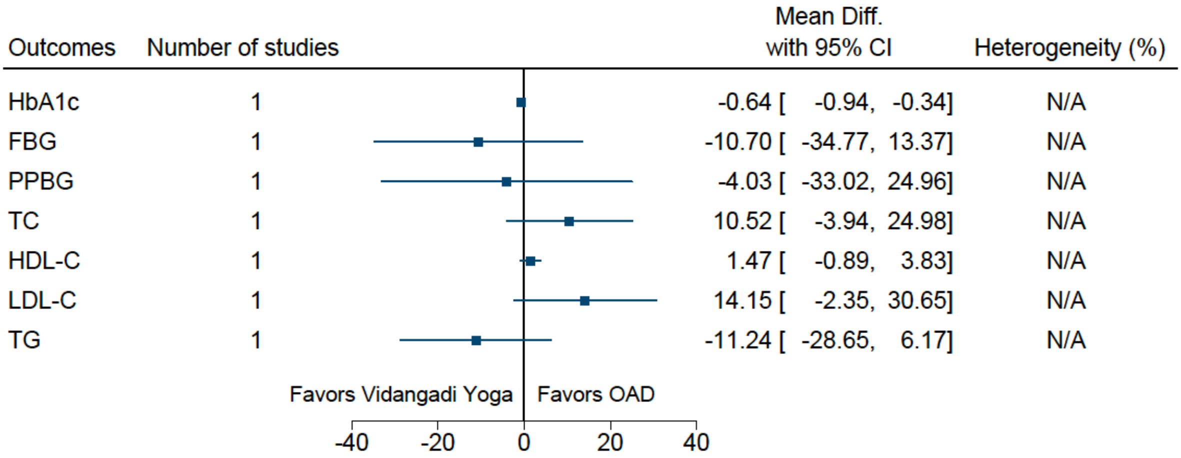

Vijaysaradi Ghana Vati versus Madhumehari Vati (Sharma 2018)

Outcomes	Number of studies	Mean Diff. with 95% CI	Heterogeneity (%)
HbA1c	1	0.75 [0.16, 1.34]	N/A
FBG	1	1.66 [-18.21, 21.53]	N/A
PPBG	1	15.68 [-12.16, 43.52]	N/A

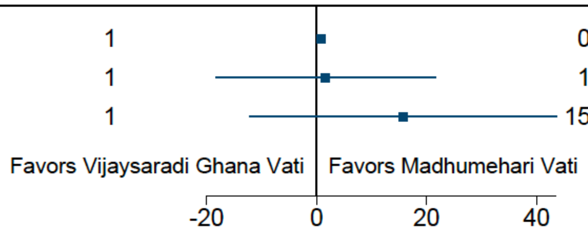

Withania coagulans (Hemalatha 2018)

Outcomes	Number of studies	Mean Diff. with 95% CI	Heterogeneity (%)
FBG	1	-73.17 [-113.67, -32.67]	N/A

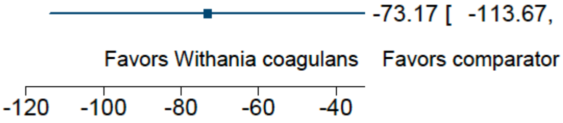

Withania somnifera (Usharani 2014)

Outcomes	Number of studies	Mean Diff. with 95% CI	Heterogeneity (%)
TC	1	-31.15 [-44.44, -17.86]	N/A
HDL-C	1	-5.50 [-8.51, -2.49]	N/A
LDL-C	1	-33.07 [-50.78, -15.36]	N/A
TG	1	-30.70 [-48.95, -12.45]	N/A

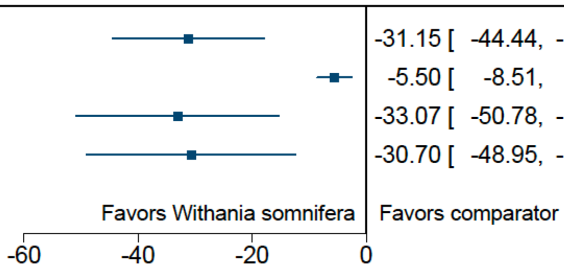

Yashad Bhasma (Srinivas 2018)

Outcomes	Number of studies	Mean Diff. with 95% CI	Heterogeneity (%)
HbA1c	1	-1.15 [-1.44, -0.86]	N/A
FBG	1	-3.10 [-14.66, 8.46]	N/A
Physical functioning	1	0.47 [-5.32, 6.26]	N/A
Role limitations due to physical health	1	-2.52 [-9.26, 4.22]	N/A
Limitations due to emotional problems	1	3.54 [-6.34, 13.42]	N/A
Energy/fatigue	1	-2.64 [-7.61, 2.33]	N/A
Emotional well being	1	1.34 [-3.49, 6.17]	N/A
Social functioning	1	3.16 [-1.99, 8.31]	N/A
Pain	1	-0.12 [-5.81, 5.57]	N/A
General health	1	-3.11 [-9.27, 3.05]	N/A
PPBG	1	-1.20 [-23.47, 21.07]	N/A
TC	1	-10.81 [-24.15, 2.53]	N/A
HDL-C	1	1.87 [-0.52, 4.26]	N/A
LDL-C	1	-5.04 [-17.17, 7.09]	N/A
TG	1	-3.55 [-22.60, 15.50]	N/A

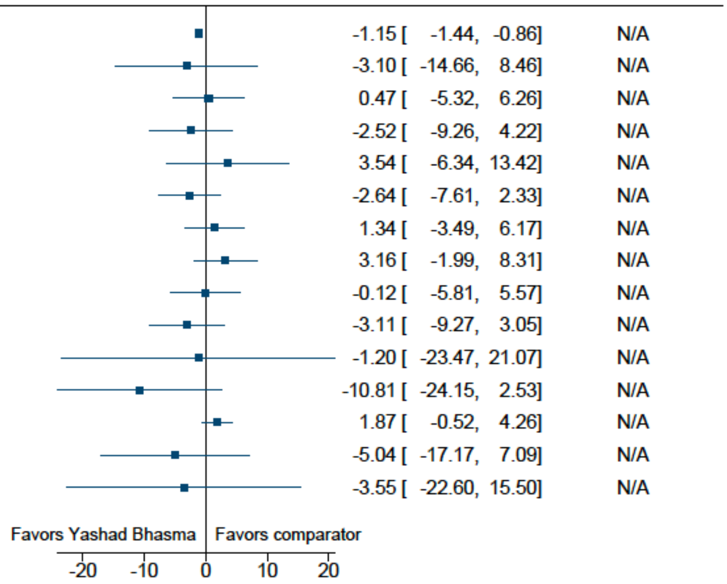

***Ziziphus mauritiana* (Yazdanpanah 2017)**

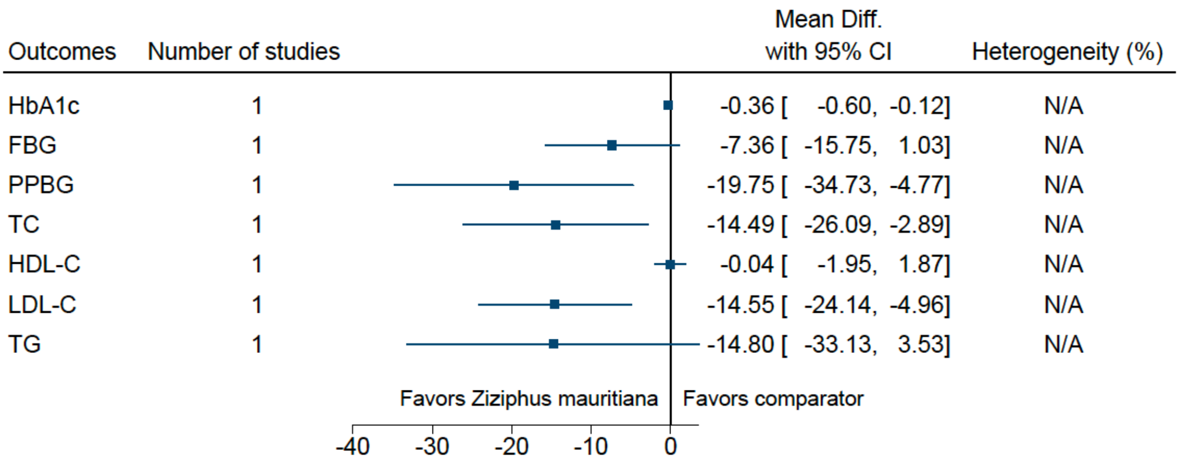

Appendix S8: Adverse events and dropouts/withdrawals/discontinued interventions due to adverse events in the included studies.

Author and year	Study arms	Adverse events (including serious adverse events)	Dropouts/withdrawals/discontinued interventions due to adverse events (if mentioned)
**Ayurvedic medicine- single plant- or mineral-origin ingredient**			
Abelmoschus esculentus			
Moradi 2020	A1=*Abelmoschus esculentus*	0	
	A2=Placebo		
Acacia Senegal			
Babiker 2017, Babiker 2018	A1=*Acacia senegal*	Abdominal boating-6, Diarrhea-11, Discomfort with viscous sensation-32, Nausea-8	
	A2=Placebo		
Aegle marmelos			
Sankhla 2009	A1=*Aegle marmelos*		
	A2=Placebo		
Sharma 2013	A1=*Aegle marmelos*	0	
	A2=Placebo	0	
Nigam 2019	A1=*Aegle marmelos*	Exact names and numbers not provided	
	A2=No additional medicine?		
Allium sativum			
Ashraf 2005	A1=*Allium sativum*		Heart burn-1
	A2=Placebo		
Ashraf 2011a	A1=*Allium sativum*	Gastric discomfort-1	Heart burn-3
	A2=OAD		Vague abdominal discomfort-2, Weight loss-1
	A3=Placebo		
Ashraf 2011b	A1=*Allium sativum*	Gastric discomfort-1	Heart burn-1
	A2=Placebo		
Kumar 2013	A1=*Allium sativum*		
	A2=No additional medicine		
Aloe vera			
Arora 2009	A1=*Aloe vera*		Diarrhea-1, Vomiting-1
	A2=No additional medicine		
Huseini 2012a, Huseini 2012b	A1=*Aloe vera*	0	
	A2=Placebo	0	
Zarrintan 2015	A1=*Aloe vera*		
	A2=Placebo		
Anethum graveolens			
Mobasseri 2014	A1=*Anethum graveolens*		
	A2=Placebo		
Haidari 2020	A1=*Anethum graveolens*		
	A2=Placebo		
Azadirachta indica			
Usharani 2020	A1=*Azadirachta indica*	Mild gastrointestinal disturbances-2	
	A2=Placebo		

Author and year	Study arms	Adverse events (including serious adverse events)	Dropouts/withdrawals/discontinued interventions due to adverse events (if mentioned)
**Ayurvedic medicine- single plant- or mineral-origin ingredient**			
Berberis aristata			
Sharma 2017	A1=*Berberis aristata*	Nausea-2.2%, Flatulence-1.1%, Diarrhea-3.3%, Constipation- 1.1%, Rash-2.2%, Headache-4.4%, Abdominal pain-4.4%, Metallic taste-3.3%	
	A2=No additional medicine		
Boswellia serrata			
Azadmehr 2014	A1=*Boswellia serrata*	0	
	A2=Placebo		
Mehrzadi 2018	A1=*Boswellia serrata*	0	
	A2=Placebo	0	
Camellia sinensis			
MacKenzie 2007	A1=*Camellia sinensis*	Profuse sweating after ingestion of a single dose of the medicine-1, Systemic rash-1	Profuse sweating after ingestion of a single dose of the medicine-1, Systemic rash-1
	A2=Placebo	0	
Mirzaei 2009	A1=*Camellia sinensis*		
	A2=Placebo		
Hsu 2011	A1=*Camellia sinensis*	Hypoglycemia-1, Mild constipation-2, Abdominal discomfort-2	Hypoglycemia-1
	A2=Placebo	Mild constipation-1, Abdominal discomfort-1	
Lasaite 2014	A1=*Camellia sinensis*		
	A2=Placebo		
Liu 2014	A1=*Camellia sinensis*	Epigastric dullness-1, Mild constipation-2	
	A2=Placebo	Abdominal discomfort-1	
Quezada-Fernández 2019	A1=*Camellia sinensis*		Insulin therapy-1
	A2=Placebo		Insulin therapy-1
Capparis spinosa			
Huseini 2013	A1=*Capparis spinosa*	0	
	A2=Placebo	0	
Cichorium intybus			
Chandra 2020	A1=*Cichorium intybus*		
	A2=Placebo		
Cinnamomum aromaticum			
Mang 2006	A1=*Cinnamomum aromaticum*		Weight change ≥5%-2, Serious disease (exact names not provided)-4
	A2=Placebo		
Suppapitiporn 2006	A1=*Cinnamomum aromaticum*	0	
	A2=Placebo	0	
Blevins 2007	A1=*Cinnamomum aromaticum*		OAD or lipid-lowering drug therapy amendment-5
	A2=Placebo		
Crawford 2009	A1=*Cinnamomum aromaticum*	Rash-1	Rash-1
	A2=No additional medicine	0	
Akilen 2010	A1=*Cinnamomum aromaticum*	0	
	A2=Placebo	Mild gastric upset-1	Insulin therapy-2
Wainstein 2011	A1=*Cinnamomum aromaticum*		

Author and year	Study arms	Adverse events (including serious adverse events)	Dropouts/withdrawals/discontinued interventions due to adverse events (if mentioned)
**Ayurvedic medicine- single plant- or mineral-origin ingredient**			
	A2=Placebo		
Lu 2012	A1=*Cinnamomum aromaticum*		
	A2=Placebo		
Sharma 2012	A1=*Cinnamomum aromaticum?*	0	
	A2=Placebo	0	
Hasanzade 2013	A1=*Cinnamomum aromaticum*		
	A2=Placebo		
Tangvarasittichai 2015, Sengsuk 2016	A1=*Cinnamomum aromaticum*	0	
	A2=Placebo		
Cinnamomum verum			
Vafa 2012	A1=*Cinnamomum verum*	0	
	A2=Placebo	0	
Zahmatkesh 2012	A1=*Cinnamomum verum*	0	
	A2=Placebo	0	
Talaei 2017	A1=*Cinnamomum verum?*		
	A2=Placebo		
Zare 2019	A1=*Cinnamomum verum?*	0	
	A2=Placebo	Some allergic reaction-1	Some allergic reaction-1
Mirmiranpour 2020	A1=*Cinnamomum verum*	0	
	A2=Placebo	0	
Citrullus colocynthis			
Huseini 2009	A1=*Citrullus colocynthis*	Mild diarrhea-3	
	A2=Placebo		
Barghamdi 2016	A1=*Citrullus colocynthis*	0	
	A2=Placebo		
Coccinia grandis			
Kuriyan 2008, Kurpad 2008	A1=*Coccinia grandis*	Gastrointestinal tract symptoms (such as abdominal distention, flatulence, constipation, gastritis)-7, Mild hypoglycemic symptoms (such as perspiration, excessive hunger, slight dizziness) once or twice postprandially (midmorning)-17	
	A2=Placebo	Gastrointestinal tract symptoms (such as abdominal distention, flatulence, constipation, gastritis)-8	
Quamri 2017	A1=*Coccinia grandis*	0	
	A2=OAD	0	
Wasana 2021	A1=*Coccinia grandis*	0	
	A2=Placebo	0	
Convolvulus prostratus			
Patel 2012	A1=*Convolvulus prostratus*		
	A2=No additional medicine		
Crocus sativus			
Milajerdi 2018	A1=*Crocus sativus*		Headache-1

Author and year	Study arms	Adverse events (including serious adverse events)	Dropouts/withdrawals/discontinued interventions due to adverse events (if mentioned)
**Ayurvedic medicine- single plant- or mineral-origin ingredient**			
	A2=Placebo		Headache-1
Aleali 2019	A1=*Crocus sativus*		Allergy to medicine-2
	A2=Placebo		Allergy to medicine-2, Insulin therapy-2
Ebrahimi 2019	A1=*Crocus sativus*		Stomach problem-1
	A2=Placebo		Insulin therapy-2
Mobasseri 2020	A1=*Crocus sativus*		
	A2=Placebo		
Cuminum cyminum			
Jafari 2017	A1=*Cuminum cyminum*		Medicine amendment-1, Gastrointestinal complaint-1
	A2=Placebo		Medicine amendment-1
Hendre 2020	A1=*Cuminum cyminum*		
	A2=No additional medicine		
Curcuma longa			
Usharani 2008	A1=*Curcuma longa*	Mild diarrhea-2	
	A2=Placebo		
Na 2013	A1=*Curcuma longa*		
	A2=Placebo		
Chuengsamarn 2014	A1=*Curcuma longa*	Hot flash-1, Constipation-2, Nausea-1	
	A2=Placebo	Hot flash-1, Constipation-1, Vertigo-1, Iching-1	
Panahi 2017, Panahi 2018	A1=*Curcuma longa*	0	
	A2=Placebo	0	
Adab 2019	A1=*Curcuma longa*		Medicine dose amendment-1, Insulin therapy-1
	A2=Placebo	0	
Adibian 2019	A1=*Curcuma longa*		Stomach discomfort-1
	A2=Placebo		Medicine amendment-1
Srinivasan 2019	A1=*Curcuma longa*	Increased frequency of stools-1, Upper abdominal pain-1	
	A2=Placebo	Fatigue-1, Sleepiness-2	Fatigue-1
de Sousa 2020	A1=*Curcuma longa*	0	
	A2=Placebo	0	
Cyamopsis tetragonoloba			
Uusitupa 1984	A1=*Cyamopsis tetragonoloba*	Exact names not provided-2 (related)	Exact names not provided-2 (related)
	A2=Placebo		
Uusitupa 1989	A1=*Cyamopsis tetragonoloba*	Flatulence-7, Heartburn-1	
	A2=Placebo	Flatulence-1, Diarrhea-1	
Eclipta prostrata			
Sazia 2015, Sazia 2015	A1=*Eclipta prostrata*		
	A2=OAD		
	A3=No medicine		
Elettaria cardamomum			
Aghasi 2019	A1=*Elettaria cardamomum*	Dysuria-1, Mild skin inflammation-1	Medicine amendment-3

Author and year	Study arms	Adverse events (including serious adverse events)	Dropouts/withdrawals/discontinued interventions due to adverse events (if mentioned)
**Ayurvedic medicine- single plant- or mineral-origin ingredient**			
	A2=Placebo	0	Sudden increase in blood glucose level-1
Emblica officinalis			
Usharani 2013	A1=*Emblica officinalis*	Dyspepsia-3	
	A2=Placebo	0	
Enicostemma axillare			
Shankarrao 2017	A1=*Enicostemma axillare*		
	A2=OAD		
Gynostemma pentaphyllum			
Huyen 2010	A1=*Gynostemma pentaphyllum*	0	
	A2=Placebo	0	
Huyen 2012	A1=*Gynostemma pentaphyllum*	0	
	A2=Placebo	0	
Hibiscus sabdariffa			
Sarbini 2019	A1=*Hibiscus sabdariffa*		OAD dose/type amendment-1, Hospitalization due to illness-1?
	A2=Placebo		
Ipomoea batatas			
Ludvik 2004	A1=*Ipomoea batatas*	A1=Exact names not provided-16 (relation could not be excluded-6); A2=Exact names not provided-14 (relation could not be excluded-2); Most common in A1 and A2=Mild gastrointestinal issues (constipation, gastric pain, meteorism)	
	A2=Placebo		
Ludvik 2008	A1=*Ipomoea batatas*	A1=Exact names not provided-96; A2=Exact names not provided-120; Most common in A1 and A2=Nausea-3, Tympanites or diarrhea-3	
	A2=Placebo		
Juglans regia			
Hosseini 2014a	A1=*Juglans regia*	0	
	A2=Placebo	0	
Hosseini 2014b	A1=*Juglans regia*	Mild adverse events (such as diarrhea, vertigo, anxiety)-11	
	A2=Placebo	Mild adverse events (such as diarrhea, vertigo, anxiety)-4	
Abdoli 2017	A1=*Juglans regia*	0	
	A2=Placebo		
Zibaeenezhad 2016, Zibaeenezhad 2017	A1=*Juglans regia*	Gastrointestinal oil intolerance-2	Gastrointestinal oil intolerance-2?
	A2=Placebo?	0	
Rabiei 2018	A1=*Juglans regia*		OAD amendment and other reasons-5
	A2=Placebo		OAD amendment and other reasons-6
Linum usitatissimum			
Barre 2008	A1=*Linum usitatissimum*		
	A2=Placebo		
Hashemzadeh 2017	A1=*Linum usitatissimum*	0	
	A2=Placebo		

Author and year	Study arms	Adverse events (including serious adverse events)	Dropouts/withdrawals/discontinued interventions due to adverse events (if mentioned)
**Ayurvedic medicine- single plant- or mineral-origin ingredient**			
Momordica charantia			
Dans 2007	A1=*Momordica charantia*	Diarrhea and epigastric pain-1, Diarrhea-2, Hospitalization due to gastroenteritis-1, Hospitalization due to cholecystolithiasis-1 (unrelated), Chest pain-1, Urinary incontinence-1, Fever-1	Diarrhea and epigastric pain-1
	A2=Placebo	Diarrhea-1	
Zänker 2012	A1=*Momordica charantia*		OAD amendment-6, Insulin therapy-2, OAD amendment and lost body weight-1, Lipid-lowering drug therapy amendment-5
	A2=Placebo		
Trakoon-osot 2013	A1=*Momordica charantia*	Mild diarrhea-5, Flatulence-8	
	A2=Placebo	Flatulence-2	
Rahman 2015	A1=*Momordica charantia*	Hyperacidity/heart burn-8, Anorexia-10, Nausea-5, Diarrhea-3, Appetite-7, Abdominal discomfort-2, Headache-13, Dizziness-3, Skin rash-2	
	A2=OAD	Hyperacidity/heart burn-1, Anorexia-3, Nausea-1, Appetite-3, Headache-5, Dizziness-2, Skin rash-2	
Suthar 2016a	A1=*Momordica charantia*	0	
	A2=Placebo	0	
Suthar 2016b	A1=*Momordica charantia*	22 mild or moderate treatment emergent adverse events (exact names not provided)-14 (mostly unrelated)<br><br>Hypotension-1 (unrelated)	Blood glucose level >270 mg/dL-2
	A2=OAD	9 mild or moderate treatment emergent adverse events (exact names not provided)-7 (mostly unrelated)<br><br>Pyrexia-1 (unrelated)	
Cortez-Navarrete 2018	A1=*Momordica charantia*	Headache and dizziness-33.3%, Nausea-16.7%, Vomiting-8.3%, Constipation-8.3%	
	A2=Placebo	Headache-25%, Constipation-16.7%, Abdominal distension-16.7%, Dizziness-8.3%, Nausea-8.3%	
Kumari 2018	A1=*Momordica charantia*	Mild gastrointestinal discomfort such as transient dyspepsia and diarrhea-2	
	A1=Placebo	0	
Amini 2020	A1=*Momordica charantia*		
	A2=Placebo		
Kim 2020	A1=*Momordica charantia*	Anorexia/nausea-2, Abdominal discomfort-3, Diarrhea-1, Constipation-2, Foamy urine-1, Skin rash-1	Exact names not provided and other reasons-14
	A2=Placebo	Anorexia/nausea-1, Abdominal discomfort-2, Epigastric soreness-1, Foamy urine-1, Skin rash-1	Exact names not provided and other reasons-6
Nigella sativa			

Author and year	Study arms	Adverse events (including serious adverse events)	Dropouts/withdrawals/discontinued interventions due to adverse events (if mentioned)
**Ayurvedic medicine- single plant- or mineral-origin ingredient**			
Najmi 2012	A1=*Nigella sativa*	0	
	A2=No additional medicine	0	
Hosseini 2013	A1=*Nigella sativa*	Mild transient nausea-4	
	A2=Placebo	0	
Hadi 2015	A1=*Nigella sativa*		Insulin therapy-1?
	A2=Placebo		Insulin therapy-3?
Heshmati 2015	A1=*Nigella sativa*	Mild gastrointestinal problems-Exact numbers not provided	Medicine amendment-1, Stomach ache-1
	A2=Placebo		Stomach ache-2
Kaatabi 2015	A1=*Nigella sativa*	0	
	A2=Placebo		Uncomfortable with stool color-4, Nausea-2
Moustafa 2019	A1=*Nigella sativa*		Nausea-2
	A2=OAD		Gastrointestinal tract upset-3, OAD amendment-1
Kooshki 2020	A1=*Nigella sativa*	0	
	A2=Placebo	0	
Jangjo-Borazjani 2021	A1=*Nigella sativa*		
	A2=Placebo		
Plantago ovata			
Ziai 2005	A1=*Plantago ovata*	Flatulence-4, Diarrhea-1, Constipation-1, Reduced flushing-11	
	A2=Placebo	Flatulence-13, Diarrhea-1, Constipation-1	Exact names not provided-5
Feinglos 2013	A1=*Plantago ovata*	A1 and A2=Exact names not provided-Distributed approximately equally (80–88% of participants in each arm); A2=Transient symptoms consistent with episodes of hypoglycemia-2 (they were on OAD as well)	Exact names not provided-2
	A2=Placebo		
Abutair 2016	A1=*Plantago ovata*		
	A2=No additional medicine?		
Portulaca oleracea			
El-Sayed 2011	A1=*Portulaca oleracea*		
	A2=OAD		
Farzanegi 2014	A1=*Portulaca oleracea*		
	A2=Placebo		
Dehghan 2016	A1=*Portulaca oleracea*		
	A2=Placebo		
Wainstein 2016	A1=*Portulaca oleracea*	Acute bronchitis-1 (unrelated), Constipation-1 (probably related), electrocardiogram (ECG) changes-1 (not assessable), Uncontrolled hypertension-1 (not assessable), Vision disturbance-1 (unrelated)	Exact name not provided-1
	A2=Placebo	Elevated blood glucose level-1 (probably related), Influenza-1 (unrelated), Abnormal liver enzyme levels-1 (unrelated)	
Pterocarpus marsupium			

Author and year	Study arms	Adverse events (including serious adverse events)	Dropouts/withdrawals/discontinued interventions due to adverse events (if mentioned)
**Ayurvedic medicine- single plant- or mineral-origin ingredient**			
Hariharan 2005	A1=*Pterocarpus marsupium*	0	
	A2=OAD		Dizziness on fasting-1, Insulin therapy-1
Punica granatum			
Faghihimani 2016	A1=*Punica granatum*	Rash and itching-2, Gastrointestinal upset-1, Hospitalization due to upper gastrointestinal bleeding-1?	Rash and itching-2, Gastrointestinal upset-1, Hospitalization due to upper gastrointestinal bleeding-1?
	A2=Placebo	Headache-1, Mild abdominal pain-2	Headache-1, Mild abdominal pain-2
Babaeian 2013	A1=*Punica granatum*		Intolerance-2
	A2=Placebo		
Sohrab 2014, Sohrab 2015	A1=*Punica granatum*		Stomach discomfort-1
	A2=Placebo		Medicine amendment-1
Khajebishak 2019a, Khajebishak 2019b	A1=*Punica granatum*	0	
	A2=Placebo		
Grabež 2020	A1=*Punica granatum*		
	A2=Placebo		
Hashemi 2020	A1=*Punica granatum*	0	
	A2=Placebo	0	
Sesamum indicum			
Shahi 2017	A1=*Sesamum indicum*		Insulin therapy-2
	A2=Placebo		Medicine dose/type amendment-2
Aslam 2018	A1=*Sesamum indicum*		
	A2=Placebo		
**Shilajit**			
Narasimha Raju 2016	A1= Shilajit		
	A2=Placebo		
Niranjan 2016	A1=Shilajit	Mild headache-3	
	A2=Placebo	Dyspepsia-2	
Syzygium cumini			
Sahana 2010	A1=*Syzygium cumini*		Uncontrolled blood glucose level-2
	A2=OAD		
	A3=No medicine		
Sidana 2016, Sidana 2017	A1=*Syzygium cumini*		
	A2=Placebo		
Terminalia chebula			
Usharani 2020	A1=*Terminalia chebula*	Dyspepsia-3	
	A2=Placebo	Mild headache-3	
Tinospora cordifolia			
Mishra 2015	A1=*Tinospora cordifolia*	0	
	A2=No additional medicine		
Roy 2015	A1=*Tinospora cordifolia*	0	
	A2=No additional medicine	0	
Tribulus terrestris			

Author and year	Study arms	Adverse events (including serious adverse events)	Dropouts/withdrawals/discontinued interventions due to adverse events (if mentioned)
**Ayurvedic medicine- single plant- or mineral-origin ingredient**			
Samani 2016	A1=*Tribulus terrestris*	0	
	A2=Placebo	0	
Trigonella foenum-graecum			
Gupta 2001	A1=*Trigonella foenum-graecum*	Dyspepsia and mild abdominal distension-5	
	A2=Placebo		
Lu 2008	A1=*Trigonella foenum-graecum*	Stomach discomfort and nausea-2, Diarrhea-1	
	A2=Placebo		
Ansari 2011	A1=*Trigonella foenum-graecum*		
	A2=No additional medicine		
Rafraf 2014	A1=*Trigonella foenum-graecum*	0	
	A2=Placebo	0	
Suchitra 2015	A1=*Trigonella foenum-graecum*	0	
	A2=No additional medicine	0	
Kaur 2016	A1=*Trigonella foenum-graecum*	Mild diarrhea-1, Flatulence-1, Mild nausea/vomiting-1, Mild facial swelling-1, Mild itching-1	
	A2=No additional medicine	0	
Singh 2016	A1=*Trigonella foenum-graecum*	Mild dyspepsia-2, Flatulence/mild abdominal bloating-4, Mild diarrhea-1, Mild pain abdomen and mild abdominal bloating-1	
	A2=*Trigonella foenum-graecum*+OAD	Flatulence/mild abdominal bloating-2	
	A3=OAD	Mild nausea-1, Mild hypoglycemia-1, Mild headache-2	
Verma 2016	A1=*Trigonella foenum-graecum*		
	A2=Placebo		
Ranade 2017	A1=*Trigonella foenum-graecum*		
	A2=No additional medicine		
Gholaman 2018	A1=*Trigonella foenum-graecum*		
	A2=Placebo		
Kandhare 2018	A1=*Trigonella foenum-graecum*	Musculoskeletal system disorders-4, Central and peripheral nervous system-10, Vision disorders-1, Psychiatric disorders-2, Gastrointestinal system disorders-10, Liver and biliary system-9, Metabolic and nutritional disorders-42, Cardiovascular disorders-7, Heart rate and rhythm disorders-2, Respiratory system disorders-6, Red blood cell disorders-8, White cells disorders-8, Platelet bleeding and clotting disorders-1, Urinary system disorders-4, Female reproductive disorders-1, Body as a whole (general disorders)-30, Resistance mechanism disorders-4, Total drug-related adverse events (exact names not provided)-25<br><br>Hypoglycemia-10 (related), 2 (unrelated)<br><br>Hypertriglyceridemia-1 (unrelated)	Failure of medicine and adverse event (exact name not provided)-1, Exact names not provided-3, Adverse events (exact names not provided) and other reasons-4

Author and year	Study arms	Adverse events (including serious adverse events)	Dropouts/withdrawals/discontinued interventions due to adverse events (if mentioned)
**Ayurvedic medicine- single plant- or mineral-origin ingredient**			
	A2=Placebo	Skin and appendages disorders-3, Musculoskeletal system disorders-2, Central and peripheral nervous system-8, Autonomic nervous system disorders-1, Vision disorders-1, Hearing and vestibular disorders-1, Psychiatric disorders-3, Gastrointestinal system disorders-13, Liver and biliary system-9, Metabolic and nutritional disorders-43, Cardiovascular disorders-2, Heart rate and rhythm disorders-2, Respiratory system disorders-6, Red blood cell disorders-2, White cells disorders-13, Platelet bleeding and clotting disorders-1, Urinary system disorders-3, Female reproductive disorders-1, Body as a whole (general disorders)-28, Total drug-related adverse events (exact names not provided)-19<br><br>Hypoglycemia-3 (related), 2 (unrelated)<br><br>Leucocytosis-1	Failure of medicine-1, Exact names not provided-2, Adverse events (exact names not provided) and other reasons-9
Hassani 2019	A1=*Trigonella foenum-graecum*		
	A2=Placebo		
Hota 2019	A1=*Trigonella foenum-graecum*	0	
	A2=No additional medicine	0	
Najdi 2019	A1=*Trigonella foenum-graecum*	Hypoglycemia-1	Medicine amendment and other reasons-1
	A2=OAD	0	Medicine amendment and other reasons-2
Rashid 2019	A1=*Trigonella foenum-graecum*	0	
	A2=Placebo		
Hadi 2020	A1=*Trigonella foenum-graecum*	0	
	A2=No additional medicine		OAD dose amendment-1
Urtica dioica			
Namazi 2011, Esfanjani 2012a, Esfanjani 2012b	A1=*Urtica dioica*		
	A2=Placebo		Insulin therapy-2, Nausea-1
Kianbakht 2013	A1=*Urtica dioica*	0	
	A2=Placebo	0	
Khajeh-Mehrizi 2014	A1=*Urtica dioica*		Illness (exact names not provided)-2
	A2=Placebo		Illness (exact names not provided)-4
Dabagh 2016	A1=*Urtica dioica*		
	A2=No additional medicine		
Hassani 2016	A1=*Urtica dioica*		
	A2=Placebo		
Dadvar 2017	A1=*Urtica dioica*		
	A2=No additional medicine		
Ghalavand 2017	A1=*Urtica dioica*		

Author and year	Study arms	Adverse events (including serious adverse events)	Dropouts/withdrawals/discontinued interventions due to adverse events (if mentioned)
**Ayurvedic medicine- single plant- or mineral-origin ingredient**			
	A2=No additional medicine		
Korani 2017	A1=*Urtica dioica*	Itching-1	
	A2=Placebo	0	
Mohammadnia 2017	A1=*Urtica dioica*		
	A2=Placebo		
Vernonia cinerea			
Sayeed 2013	A1=*Vernonia cinerea*	0	
	A2=Placebo		
Withania coagulans			
Hemalatha 2018	A1=*Withania coagulans*		
	A2=Placebo		
Withania somnifera			
Usharani 2014	A1=*Withania somnifera*		Abnormal laboratory reports? (exact names not provided)-4
	A2=Placebo		
Zingiber officinale			
Mahluji 2013	A1=*Zingiber officinale*	Slight heart burn-2	Medicine failure-2, Medicine amendment-1
	A2=Placebo		Medicine failure-1
Arablou 2014a, Arablou 2014b	A1=*Zingiber officinale*		Heartburn-1
	A2=Placebo		Insulin therapy-1
Mozaffari-Khosravi 2014, Talaei 2017, Talaei 2018	A1=*Zingiber officinale*	0	
	A2=Placebo	0	
Shidfar 2015	A1=*Zingiber officinale*		
	A2=Placebo		
Arzati 2017, Zarezadeh 2018	A1=*Zingiber officinale*		Insulin therapy-1
	A2=Placebo		Gastrointestinal intolerance-1
Mohammadi 2017, Mohammadi 2019	A1=*Zingiber officinale*		
	A2=Placebo		
Carvalho 2020	A1=*Zingiber officinale*	Diarrhoea-1, Gastrointestinal discomfort-1	Exact name not provided-1
	A2=Placebo		Exact name not provided-1
Gholinezhad 2020	A1=*Zingiber officinale*	0	
	A2=Placebo		
Ziziphus mauritiana			
Yazdanpanah 2017	A1=*Ziziphus mauritiana*		Hypotension-1, Hives-1
	A2=No additional medicine		Medicine amendment-1, Insulin therapy-2
Acalypha indica/Allium cepa/Allium sativum/Azadirachta indica/Mangifera indica/Murraya koenigii/Musa sapientum/Ocimum tenuiflorum/Phyllanthus amarus/Tinospora cordifolia			
Balasubramaniam 2010	A1=*Acalypha indica/Allium cepa/Allium sativum/Azadirachta indica/Mangifera indica/Murraya koenigii/Musa sapientum/Ocimum tenuiflorum/Phyllanthus amarus/Tinospora cordifolia*		

Author and year	Study arms	Adverse events (including serious adverse events)	Dropouts/withdrawals/discontinued interventions due to adverse events (if mentioned)
**Ayurvedic medicine- single plant- or mineral-origin ingredient**			
	A2=Placebo		
Allium sativum*; *Cuminum cyminum			
Mansouri 2018	A1=*Allium sativum*	0?	
	A2=*Cuminum cyminum*	0?	
	A3=Placebo		
Aloe vera*; *Pterocarpus marsupium*; *Aloe vera+Pterocarpus marsupium			
Maurya 2017	A1=*Aloe vera*	0?	
	A2=*Pterocarpus marsupium*	Diarrhea and flatulence?-1	
	A3=*Aloe vera*+*Pterocarpus marsupium*	0?	
	A4=OAD		
Cinnamomum verum*; *Crocus sativus*; *Elettaria cardamomum*; *Zingiber officinale			
Azimi 2014, Azimi 2016	A1=*Cinnamomum verum*	0	
	A2=*Crocus sativus*	0	
	A3=*Elettaria cardamomum*	0	
	A4=*Zingiber officinale*	0	
	A5=Placebo		
***Enicostemma axillare*; Shilajit**			
Kumar 2014	A1=*Enicostemma axillare*	0	
	A2=Shilajit	0	
	A3=OAD		
Enicostemma axillare+Emblica officinalis+Tinospora cordifolia			
Sharma 2019	A1=*Enicostemma axillare*+*Emblica officinalis*+*Tinospora cordifolia*		
	A2=No additional medicine		
Linum usitatissimum*; *Plantago ovata			
Ricklefs-Johnson 2017	A1=*Linum usitatissimum*	0	
	A2=*Plantago ovata*		
Syzygium cumini+Withania coagulans			
Siddiqui 2017	A1=*Syzygium cumini*+*Withania coagulans*	0	
	A2=OAD	0	
Trigonella foenum-graecum*; *Aegle marmelos*; *Trigonella foenum-graecum+Aegle marmelos			
Yaheya 2009	A1=*Trigonella foenum-graecum*	Flatulence-Exact numbers not provided, Headache-Exact numbers not provided	
	A2=*Aegle marmelos*		
	A3=*Trigonella foenum-graecum*+*Aegle marmelos*		
	A4=No additional medicine		
**Ayurvedic medicine- combination of plant- and/or mineral-origin ingredients§**			
**AYUBES**			
Godatwar 2019	A1=AYUBES	Exact names not provided-13 (mostly unrelated)	
	A2=Placebo	Exact names not provided-12 (mostly unrelated)	
**BGR-34**			
Gupta 2018	A1=BGR-34	0	

Author and year	Study arms	Adverse events (including serious adverse events)	Dropouts/withdrawals/discontinued interventions due to adverse events (if mentioned)
**Ayurvedic medicine- single plant- or mineral-origin ingredient**			
	A2=Placebo	0	
**Bilvadi Churna; Kiratadi Churna**			
Kumari 2016	A1=Bilvadi Churna		
	A2=Kiratadi Churna		
	A3=Placebo		
**CardiPro**			
Fatima 2012	A1=CardiPro	Diarrhea-2	
	A2=Placebo	Dyspepsia-1	
**Cogent db**			
Shekhar 2002	A1=Cogent db	Mild diarrhea-4	
	A2=No additional medicine	0	
**DCBT 2345**			
Mohan 2001	A1=DCBT 2345		Associated illness (vomiting, hepatitis, abdominal tuberculosis, urinary tract infection and coronary artery disease)-6, Insulin therapy due to severe hyperglycemia-6
	A2=Placebo		
**Diabetea tea**			
Mahmoud 2016	A1=Diabetea tea	0	
	A2=Placebo		
Emblica officinalis*; *Withania somnifera*; *Emblica officinalis, Withania somnifera			
Usharani 2014	A1=*Emblica officinalis*	Dyspepsia-3	
	A2=*Withania somnifera*		
	A3=*Emblica officinalis*, *Withania somnifera*		
**Herbal combination**			
Shokoohi 2017	A1=Herbal combination	0	
	A2=Placebo	0	
**Hyponidd**			
Poongothai 2002	A1=Hyponidd		Severe hyperglycemia-2, Transient increase in Serum Glutamate Oxaloacetate Transaminase (SGOT) and Serum Glutamate Pyruvate Transaminase (SGPT) levels-1
	A2=Placebo		
**Inolter**			
Agrawal 2002	A1=Inolter	0	
	A2=Placebo	0	
**Kalpit**			
Agarwal 2013	A1=Kalpit	0	
	A2=Kalpit+OAD		
	A3=OAD		
**Khadira-Kramuka Kashaya Ghanavati; Nishamalaki, Shilajit; Khadira-Kramuka Kashaya Ghanavati+Nishamalaki, Shilajit**			
Paliwal 2018	A1=Khadira-Kramuka Kashaya Ghanavati	0	
	A2=Nishamalaki, Shilajit	0	

Author and year	Study arms	Adverse events (including serious adverse events)	Dropouts/withdrawals/discontinued interventions due to adverse events (if mentioned)
**Ayurvedic medicine- single plant- or mineral-origin ingredient**			
	A3=Khadira-Kramuka Kashaya Ghanavati+Nishamalaki, Shilajit	0	
**Lodhradi Kashaya Ghana Vati**			
Bramhankar 2017	A1=Lodhradi Kashaya Ghana Vati		
	A2=Lodhradi Kashaya Ghana Vati+OAD		
	A3=OAD		
**Madhumeha Nashini Gutika; Darvyadi Kwatha; Madhumeha Nashini Gutika+Darvyadi Kwatha**			
Bhawana 2015	A1=Madhumeha Nashini Gutika	0	
	A2=Darvyadi Kwatha	0	
	A3=Madhumeha Nashini Gutika+Darvyadi Kwatha	0	
**Mamajjaka Ghana Vati; Tejashiladi Vati**			
Bhagat 2017	A1=Mamajjaka Ghana Vati	0	
	A2=Tejashiladi Vati	0	
**Mamajjaka Ghana Vati; Trikatu Gutika**			
Kataria 2017	A1=Mamajjaka Ghana Vati		
	A2=Trikatu Gutika		
**Mehagni**			
Gopalakrishna 2017	A1=Mehagni		
	A2=No additional medicine		
**Mustadi Kwatha Ghana Vati**			
Kushwaha 2017	A1=Mustadi Kwatha Ghana Vati	0	
	A2=Placebo		
**Naga Bhasma, Nishamalaki; Nishamalaki, *Hordeum vulgare***			
Desale 2018	A1=Naga Bhasma, Nishamalaki	0	Hysterectomy-1
	A2=Nishamalaki, *Hordeum vulgare*	0	
Nigella sativa*, *Trigonella foenum-graecum			
Memon 2010a, Memon 2010b, Memon 2012	A1=*Nigella sativa*, *Trigonella foenum-graecum*		
	A2=No additional medicine		
**Nisha Katakadi Kashaya; Yashad Bhasma; Nisha Katakadi Kashaya+Yashad Bhasma**			
Srinivas 2018	A1=Nisha Katakadi Kashaya		
	A2=Yashad Bhasma		
	A3=Nisha Katakadi Kashaya+Yashad Bhasma		
**Pancreas tonic**			
Hsia 2004	A1=Pancreas tonic	Sore throat, Itchy eyes, Migraine headache-4?, Exacerbation of back and leg pain-1, Mild hypoglycemia-1 (unrelated)	
	A2=Placebo	Nightmares, Dizzy spells, Abdominal pain, Flank pain, Insomnia, Leg numbness, Weakness on exertion-5?	Hospitalization due to angina exacerbation-1
**Polyherbal formulation**			
Awasthi 2015	A1=Polyherbal formulation		OAD due to uncontrolled blood glucose level-6

Author and year	Study arms	Adverse events (including serious adverse events)	Dropouts/withdrawals/discontinued interventions due to adverse events (if mentioned)
**Ayurvedic medicine- single plant- or mineral-origin ingredient**			
	A2=OAD		OAD amendment due to uncontrolled blood glucose level-5
**Salasaradi Kashaya+Shilajit+Trivanga Bhasma+*Tinospora cordifolia*, *Azadirachta indica*; Hyponidd**			
Bhat 2012	A1=Salasaradi Kashaya+Shilajit+Trivanga Bhasma+*Tinospora cordifolia*, *Azadirachta indica*		Aggravation of T2DM symptoms-Few (exact names and numbers not provided)
	A2=Hyponidd		
**Shilajit; Asanadi Ghana Vati**			
Gupta 2016	A1=Shilajit	0	
	A2=Asanadi Ghana Vati	0	
**Swarnamakshika Bhasma**			
Taviad 2016	A1=Swarnamakshika Bhasma	0	
	A2=Placebo	0	
**Talapotaka Churna**			
Nille 2018	A1=Talapotaka Churna	0	
	A2=Talapotaka Churna+OAD		
	A3=OAD		
Trigonella foenum-graecum*, *Ocimum tenuiflorum			
Mitra 2006	A1=*Trigonella foenum-graecum*, *Ocimum tenuiflorum*		
	A2=No medicine		
***Triticum aestivum*; Nishamalaki; *Triticum aestivum*+Nishamalaki**			
Samagandi 2012	A1=*Triticum aestivum*		
	A2=Nishamalaki		
	A3=*Triticum aestivum*+Nishamalaki		
**Vidangadi Yoga**			
Deshpande 2018	A1=Vidangadi Yoga	A1=4 and A2=7; Fever with chills-2, Upper respiratory tract infection-4, Hypertension-3, Knee joint pain-1, Left foot abscess-1 (all unrelated)	
	A2=OAD		
**Vijaysaradi Ghana Vati; Madhumehari Vati**			
Sharma 2018	A1=Vijaysaradi Ghana Vati		
	A2=Madhumehari Vati		

A1, A2, A3, A4, A5=Eligible study arms, OAD=Oral antidiabetic drug, T2DM=Type 2 diabetes mellitus
§Some of the Ayurvedic medicines contain single plant- or mineral-origin ingredient.
