# Supplementary figures and images for: Effectiveness and Safety of Ayurvedic Medicines in Type 2 Diabetes Mellitus Management: A Systematic Review and Meta-Analysis (part 2 of 2)
Source: Front Pharmacol. 2022 Jun 8;13:821810. doi: 10.3389/fphar.2022.821810 (PMC9213670; doi:10.3389/fphar.2022.821810)

# Allium sativum - LDL-C

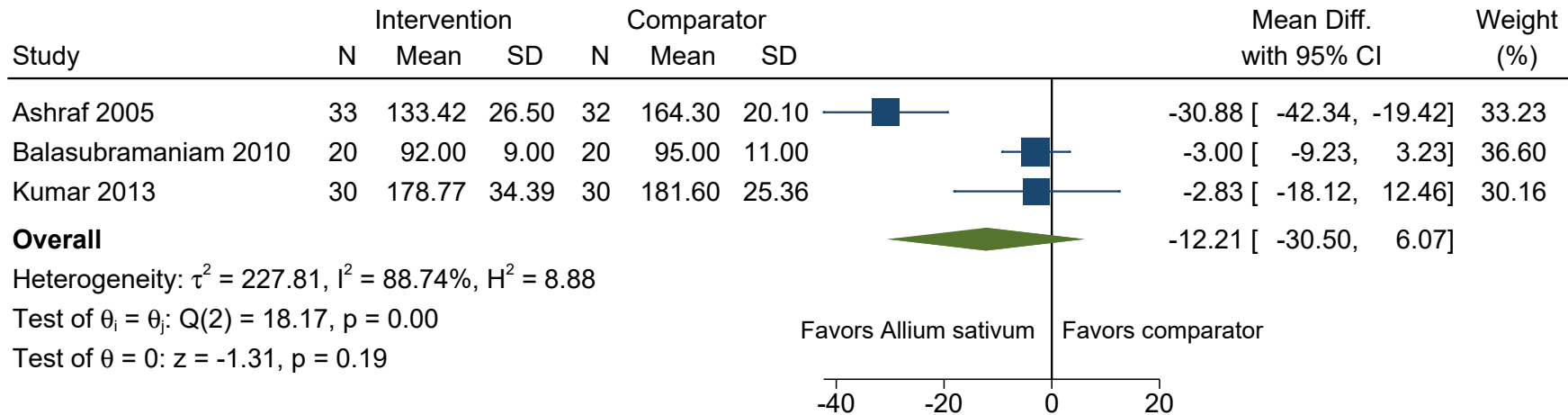

Random-effects REML model

Supplement: Supplementary file 1 [file DataSheet1.zip › Supplementary Material/Forest and Funnel Plots/Allium sativum/LDL-C.pdf]

# Elettaria cardamomum - Waist circumference

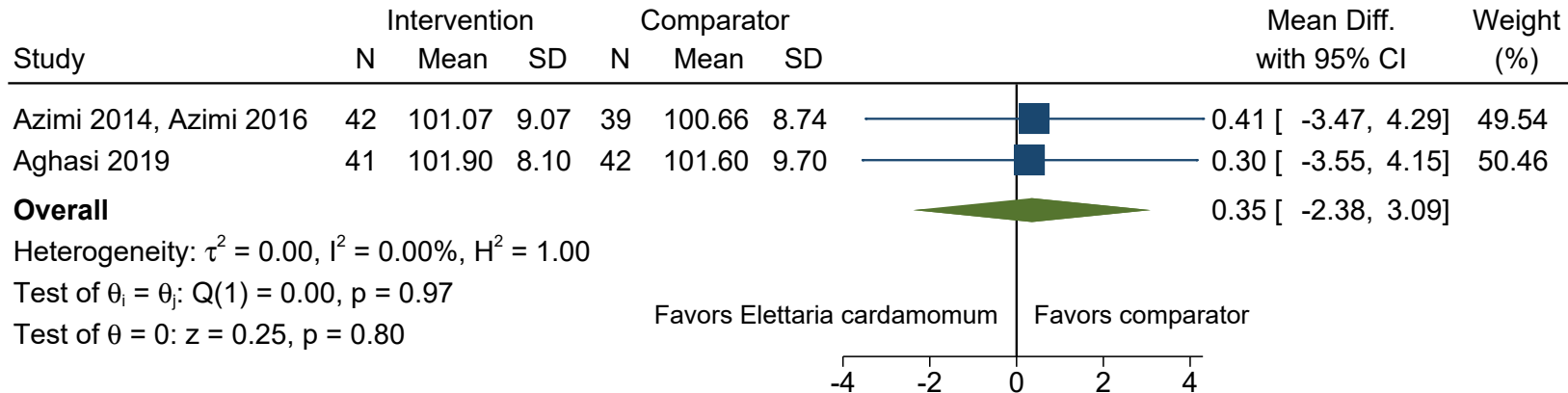

Random-effects REML model

Supplement: Supplementary file 1 [file DataSheet1.zip › Supplementary Material/Forest and Funnel Plots/Elettaria cardamomum/Waist circumference.pdf]

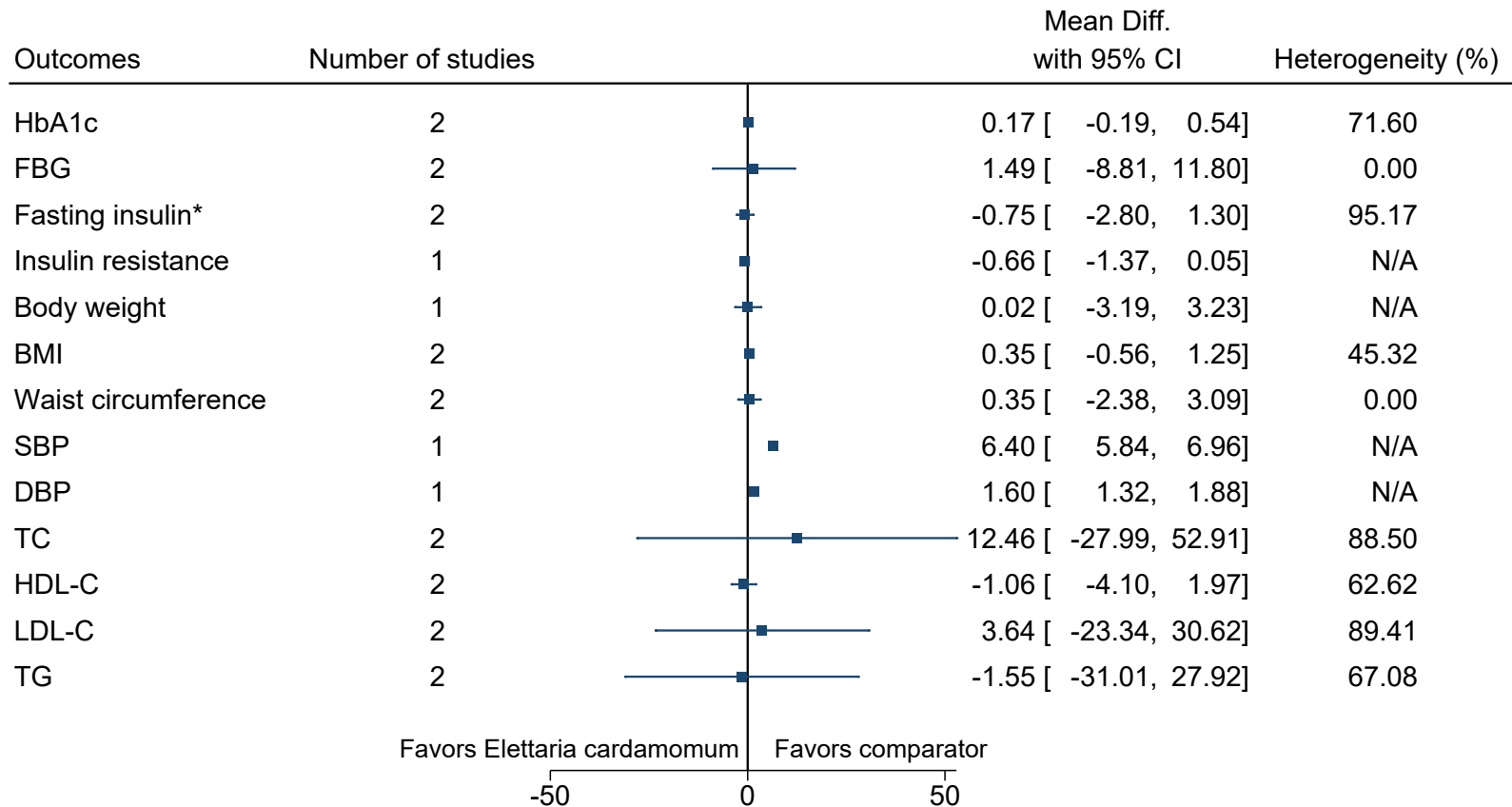

\*Favors intervention/comparator as insulin sensitizer

Supplement: Supplementary file 1 [file DataSheet1.zip › Supplementary Material/Forest and Funnel Plots/Elettaria cardamomum/Elettaria cardamomum.pdf]

# Elettaria cardamomum - TG

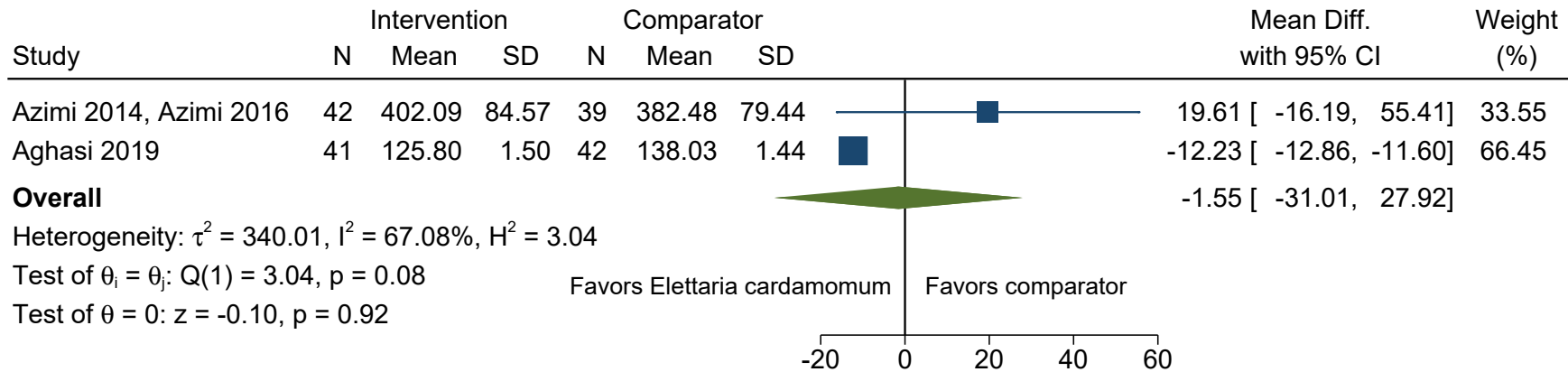

Random-effects REML model

Supplement: Supplementary file 1 [file DataSheet1.zip › Supplementary Material/Forest and Funnel Plots/Elettaria cardamomum/TG.pdf]

# Elettaria cardamomum - BMI

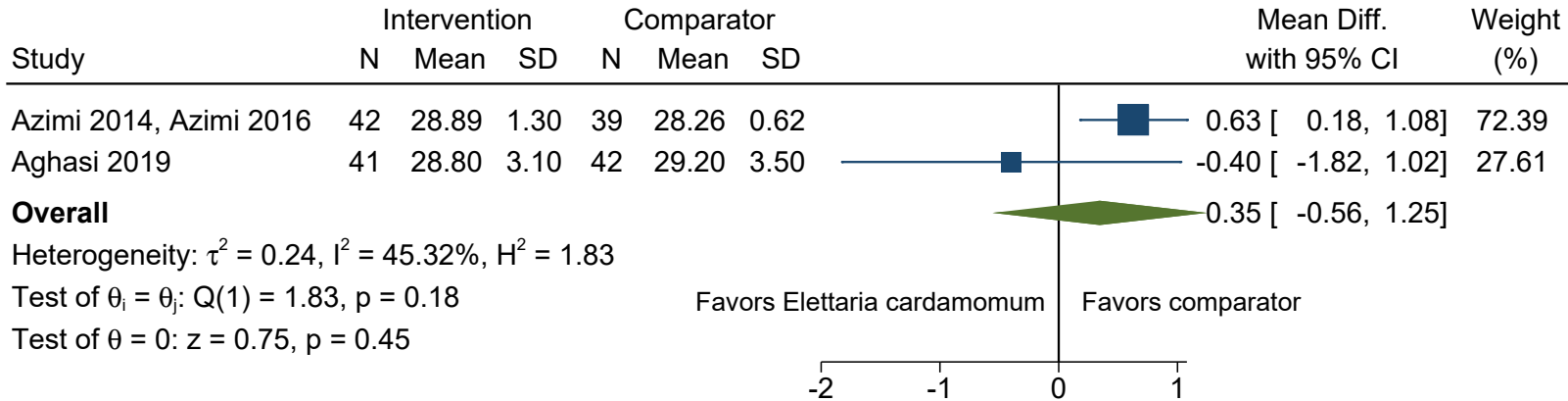

Random-effects REML model

Supplement: Supplementary file 1 [file DataSheet1.zip › Supplementary Material/Forest and Funnel Plots/Elettaria cardamomum/BMI.pdf]

# Elettaria cardamomum - TC

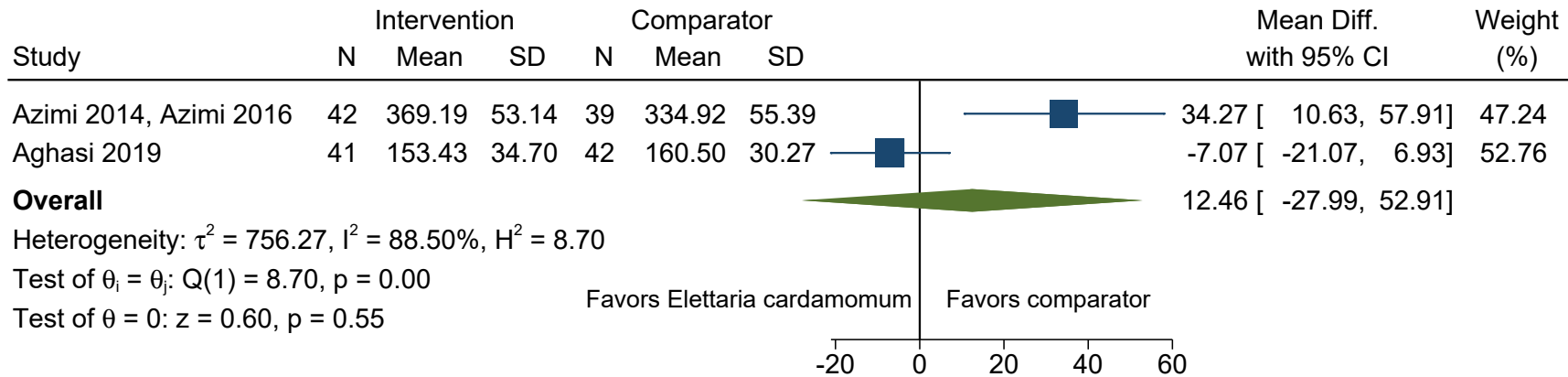

Random-effects REML model

Supplement: Supplementary file 1 [file DataSheet1.zip › Supplementary Material/Forest and Funnel Plots/Elettaria cardamomum/TC.pdf]

# Elettaria cardamomum - FBG

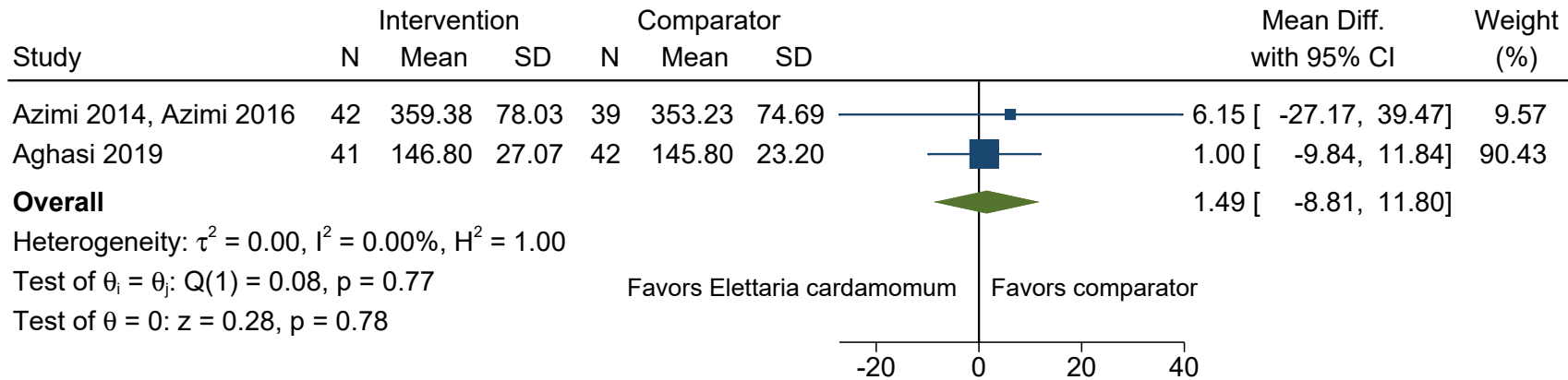

Random-effects REML model

Supplement: Supplementary file 1 [file DataSheet1.zip › Supplementary Material/Forest and Funnel Plots/Elettaria cardamomum/FBG.pdf]

# Elettaria cardamomum - HDL-C

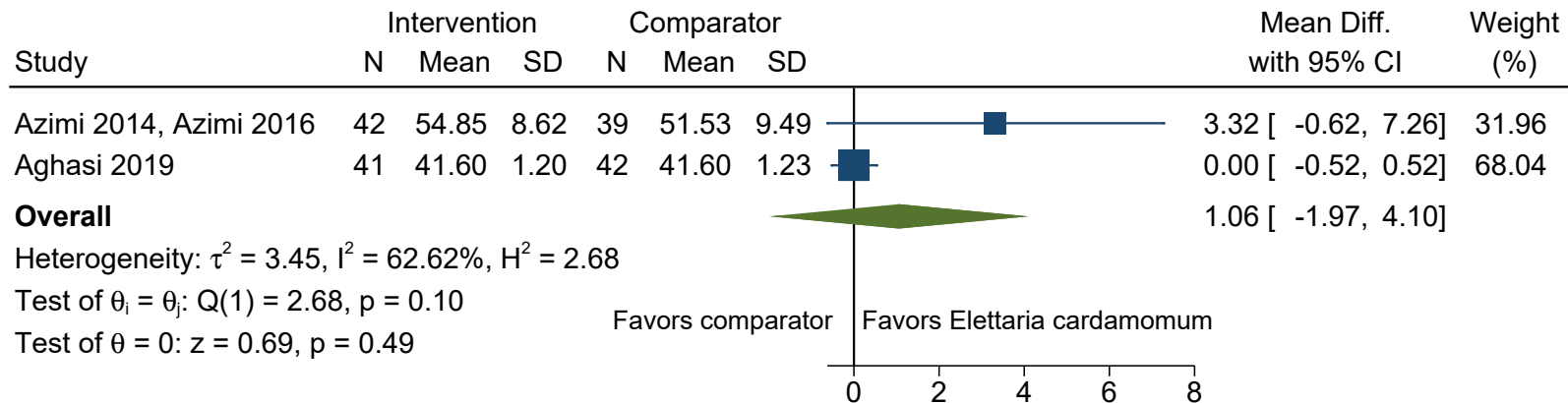

Random-effects REML model

Supplement: Supplementary file 1 [file DataSheet1.zip › Supplementary Material/Forest and Funnel Plots/Elettaria cardamomum/HDL-C.pdf]

# Elettaria cardamomum - Fasting insulin

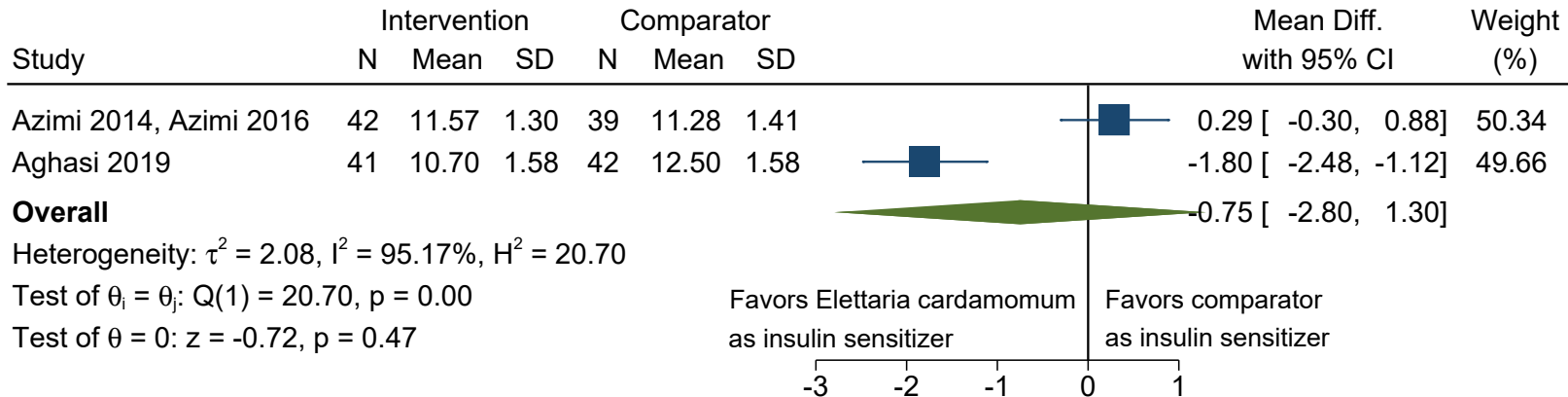

Random-effects REML model

Supplement: Supplementary file 1 [file DataSheet1.zip › Supplementary Material/Forest and Funnel Plots/Elettaria cardamomum/Fasting insulin.pdf]

# Elettaria cardamomum - HbA1c

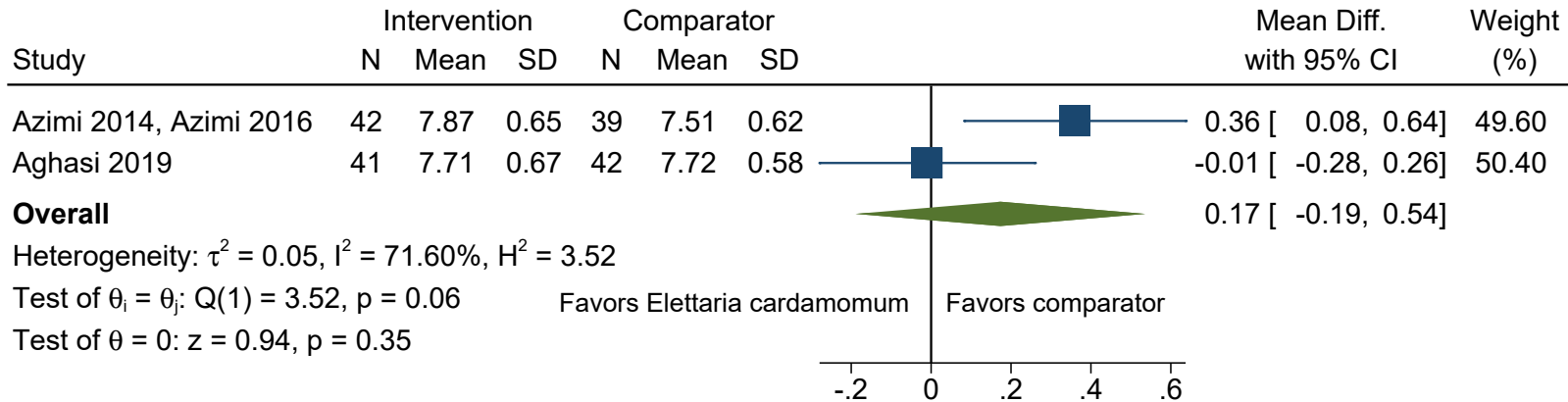

Random-effects REML model

Supplement: Supplementary file 1 [file DataSheet1.zip › Supplementary Material/Forest and Funnel Plots/Elettaria cardamomum/HbA1c.pdf]

# Elettaria cardamomum - LDL-C

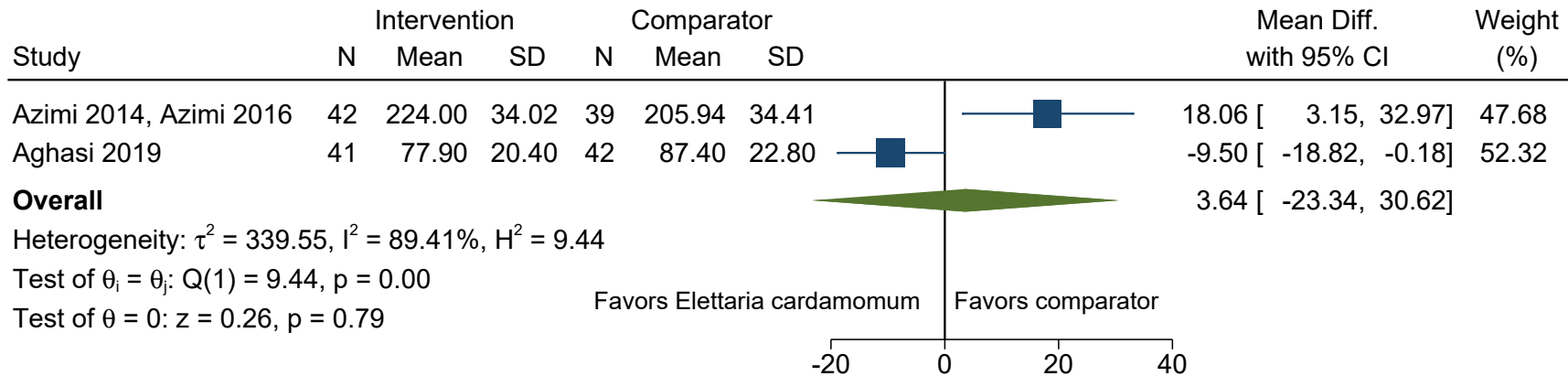

Random-effects REML model

Supplement: Supplementary file 1 [file DataSheet1.zip › Supplementary Material/Forest and Funnel Plots/Elettaria cardamomum/LDL-C.pdf]

# Cinnamomum verum - TG

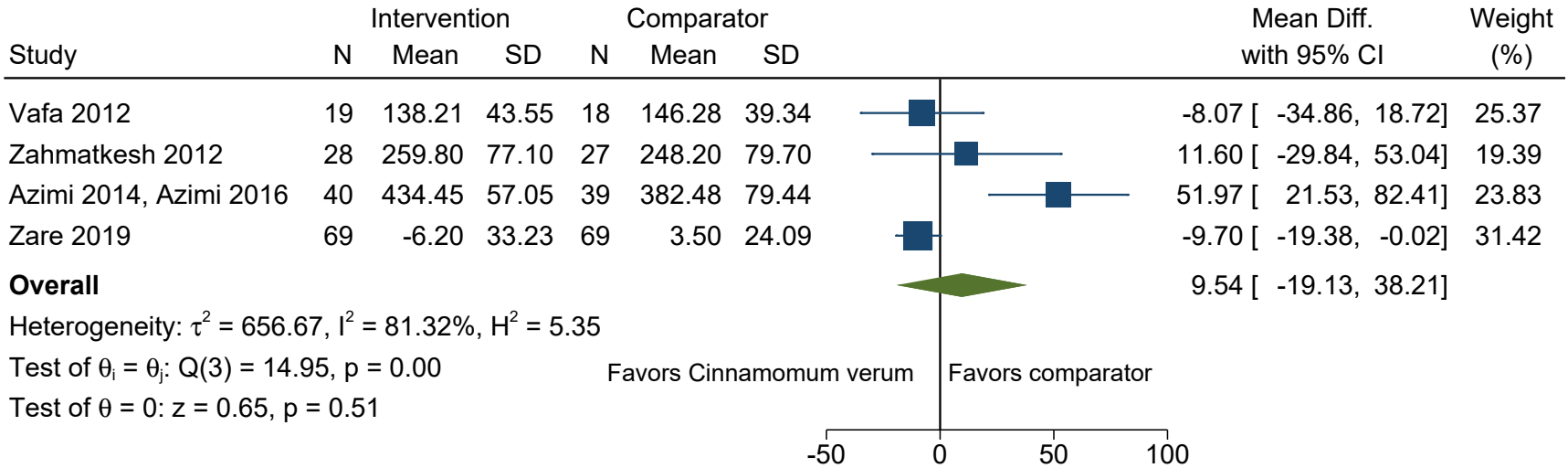

Random-effects REML model

Supplement: Supplementary file 1 [file DataSheet1.zip › Supplementary Material/Forest and Funnel Plots/Cinnamomum verum/TG.pdf]

# Cinnamomum verum - BMI

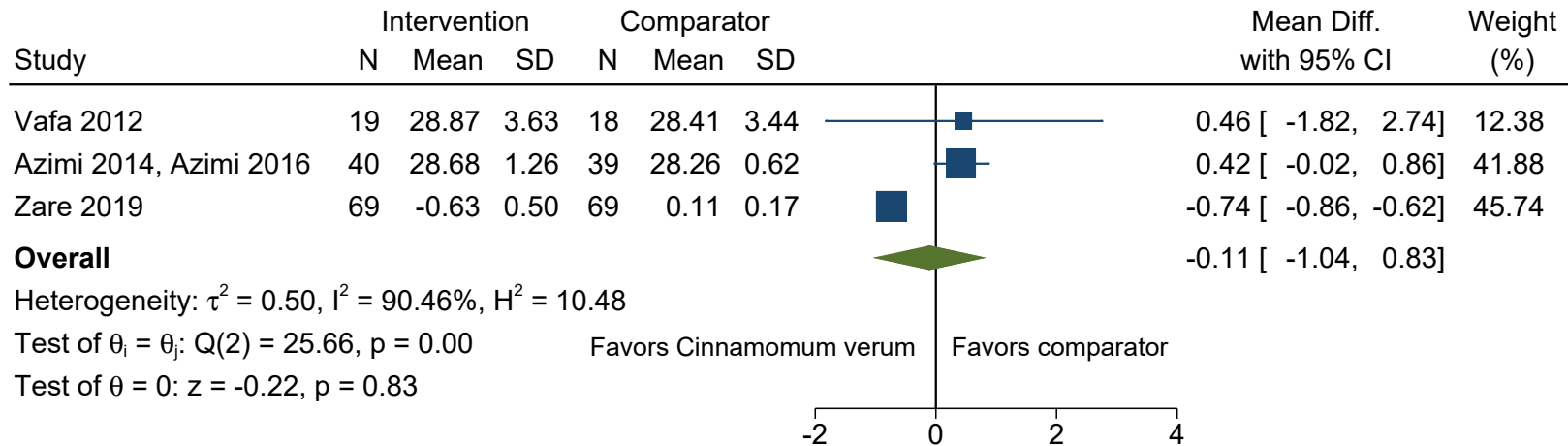

Random-effects REML model

Supplement: Supplementary file 1 [file DataSheet1.zip › Supplementary Material/Forest and Funnel Plots/Cinnamomum verum/BMI.pdf]

# Cinnamomum verum - TC

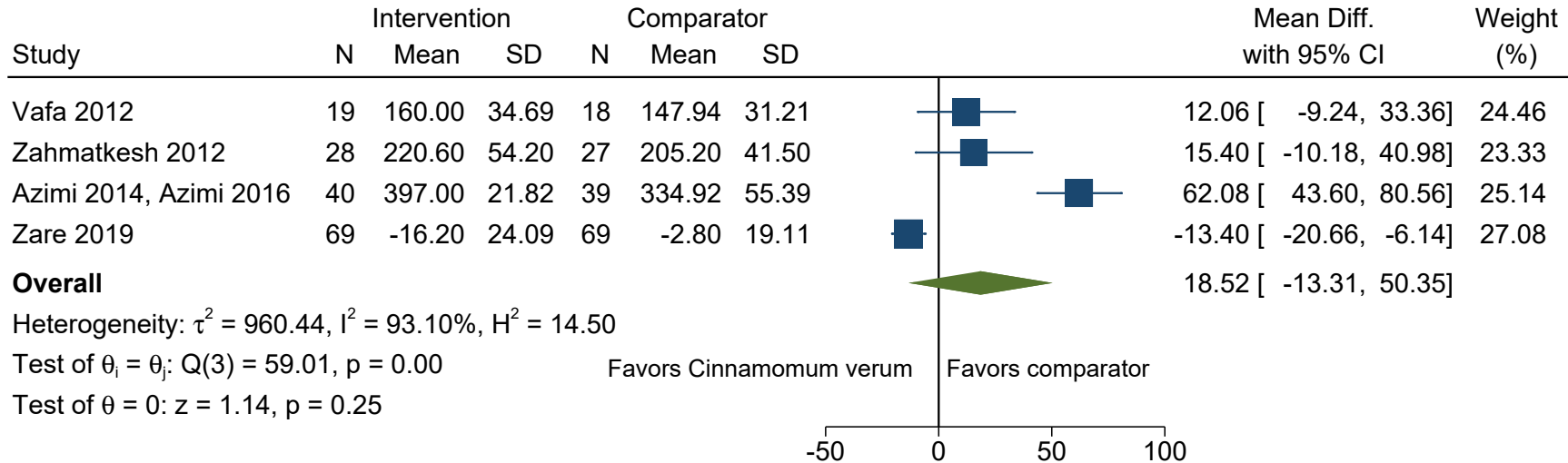

Random-effects REML model

Supplement: Supplementary file 1 [file DataSheet1.zip › Supplementary Material/Forest and Funnel Plots/Cinnamomum verum/TC.pdf]

# Cinnamomum verum - FBG

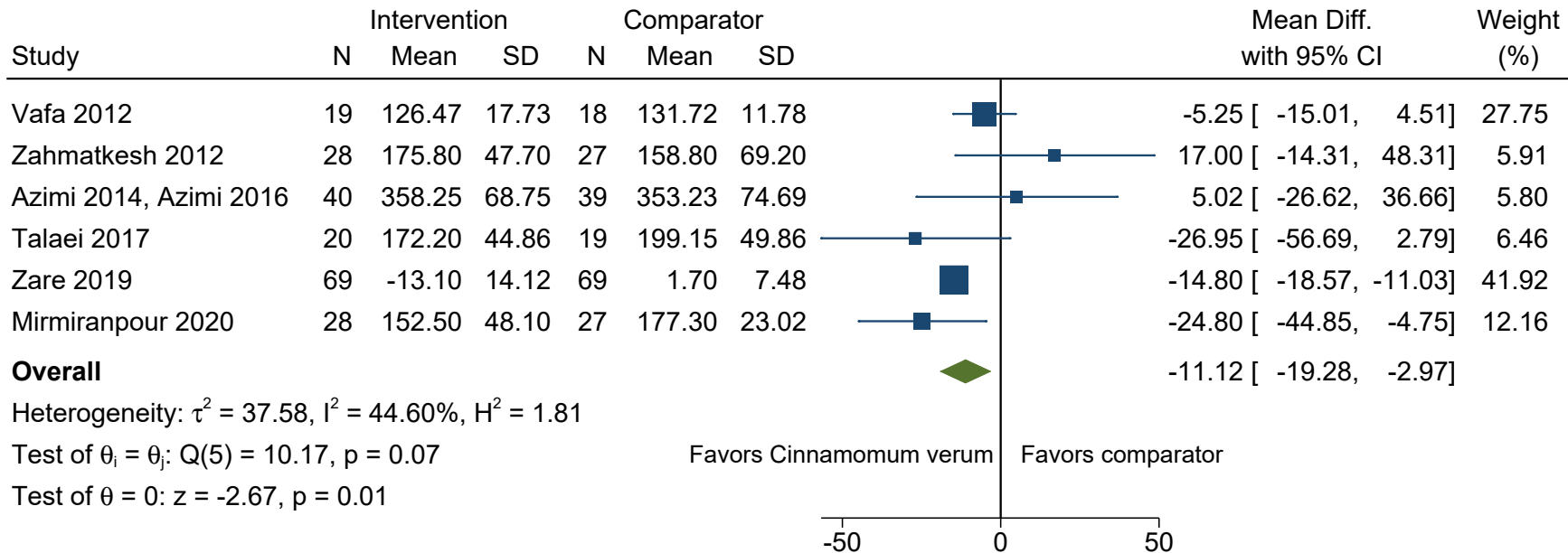

Supplement: Supplementary file 1 [file DataSheet1.zip › Supplementary Material/Forest and Funnel Plots/Cinnamomum verum/FBG.pdf]

# Cinnamomum verum - HDL-C

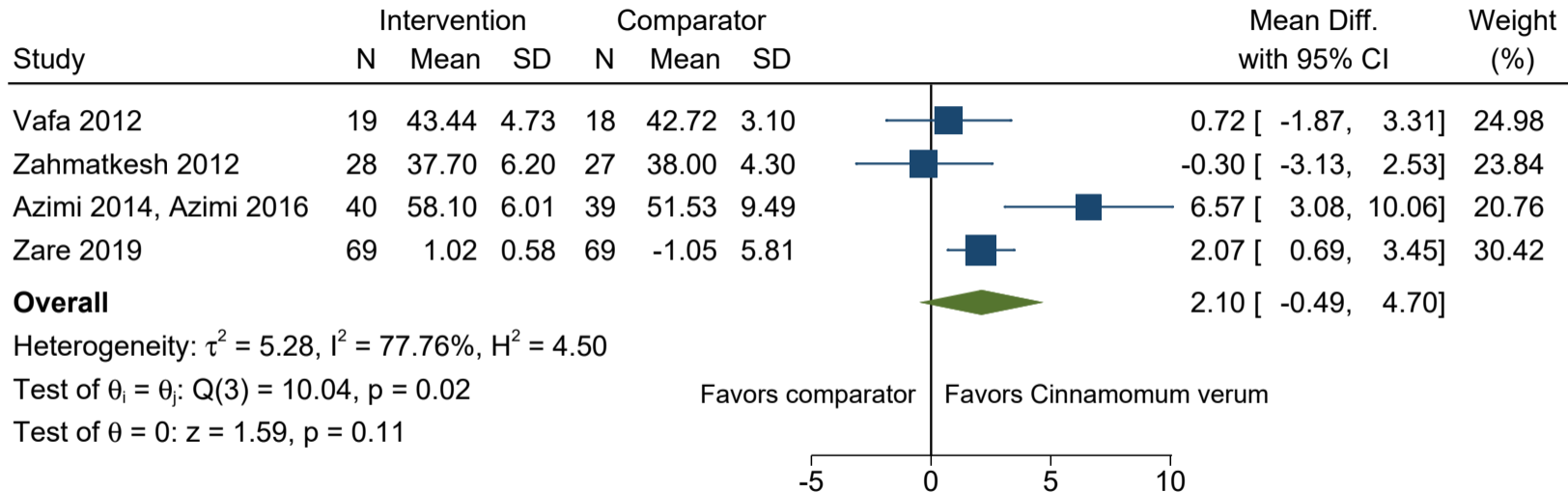

Random-effects REML model

Supplement: Supplementary file 1 [file DataSheet1.zip › Supplementary Material/Forest and Funnel Plots/Cinnamomum verum/HDL-C.pdf]

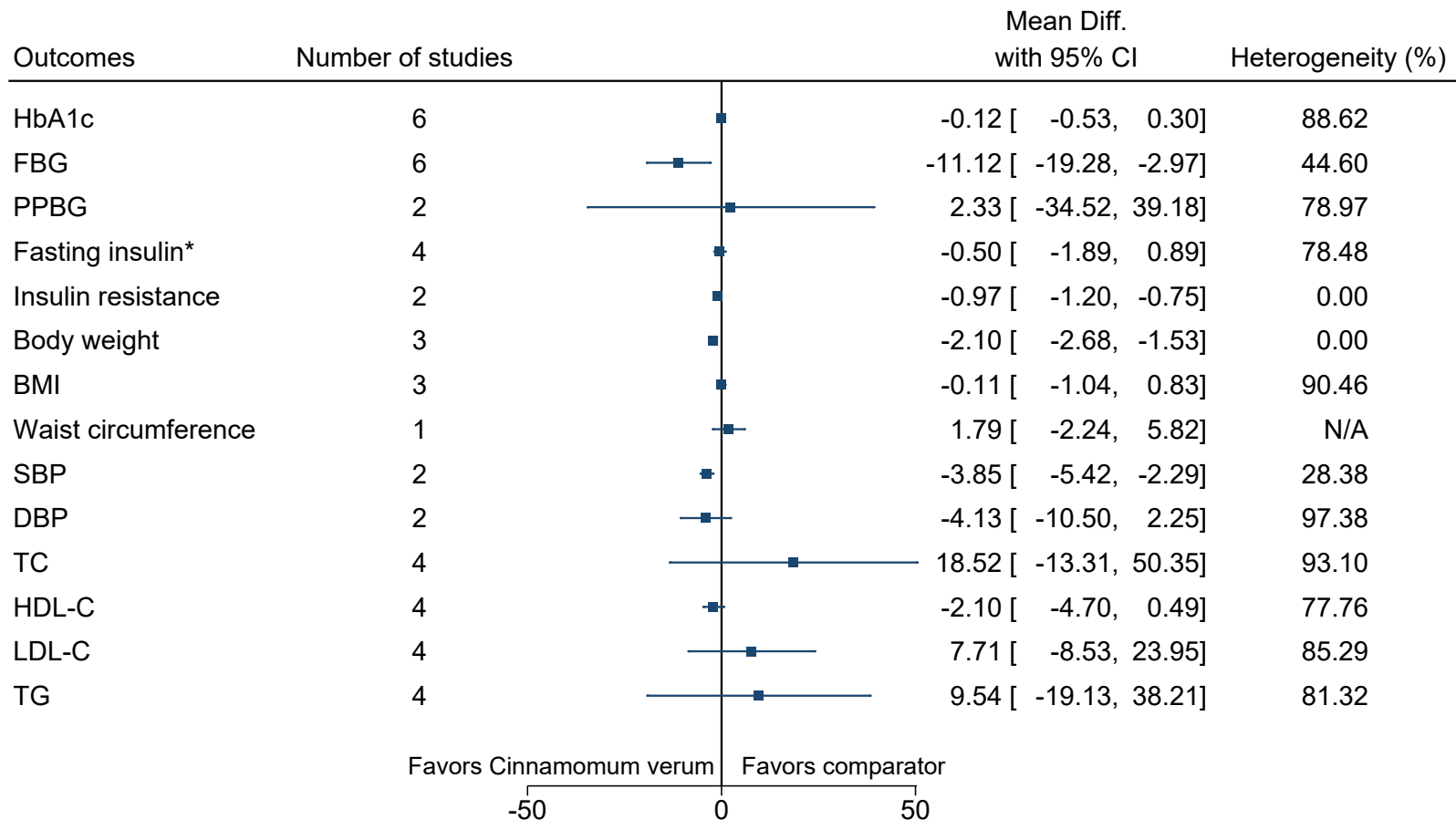

\*Favors intervention/comparator as insulin sensitizer

Supplement: Supplementary file 1 [file DataSheet1.zip › Supplementary Material/Forest and Funnel Plots/Cinnamomum verum/Cinnamomum verum.pdf]

# Cinnamomum verum - DBP

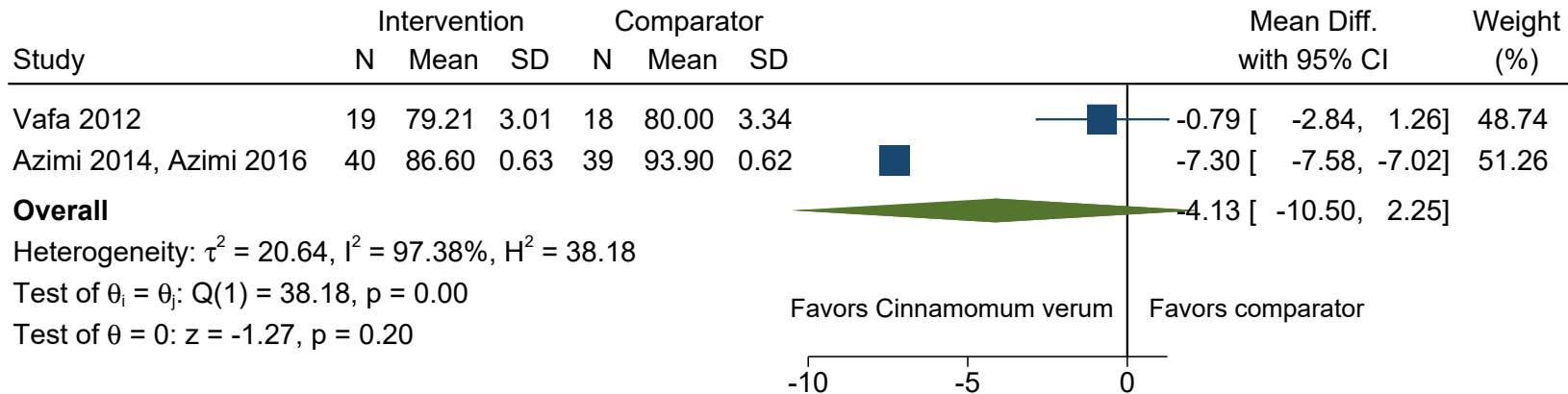

Random-effects REML model

Supplement: Supplementary file 1 [file DataSheet1.zip › Supplementary Material/Forest and Funnel Plots/Cinnamomum verum/DBP.pdf]

# Cinnamomum verum - Body weight

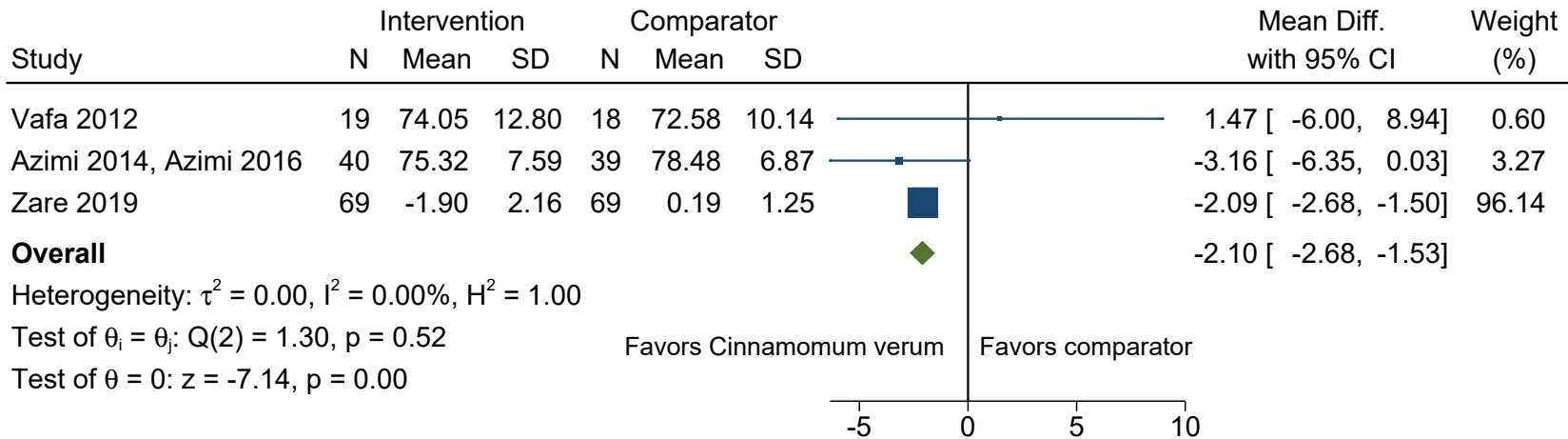

Random-effects REML model

Supplement: Supplementary file 1 [file DataSheet1.zip › Supplementary Material/Forest and Funnel Plots/Cinnamomum verum/Body weight.pdf]

# Cinnamomum verum - Insulin resistance

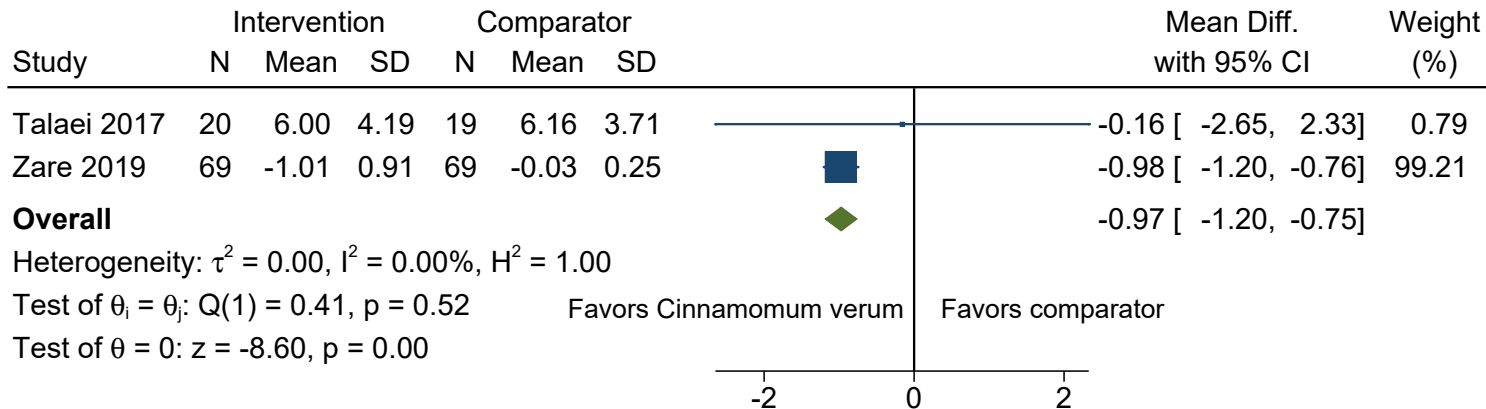

Random-effects REML model

Supplement: Supplementary file 1 [file DataSheet1.zip › Supplementary Material/Forest and Funnel Plots/Cinnamomum verum/Insulin resistance.pdf]

# Cinnamomum verum - Fasting insulin

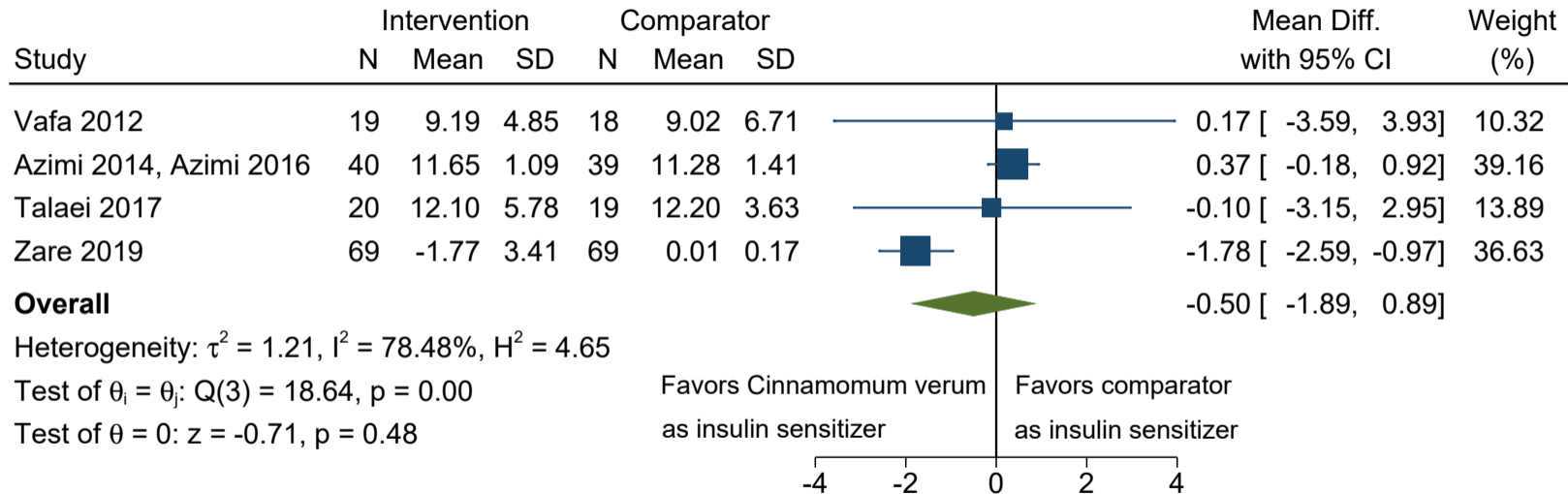

Random-effects REML model

Supplement: Supplementary file 1 [file DataSheet1.zip › Supplementary Material/Forest and Funnel Plots/Cinnamomum verum/Fasting insulin.pdf]

# Cinnamomum verum - HbA1c

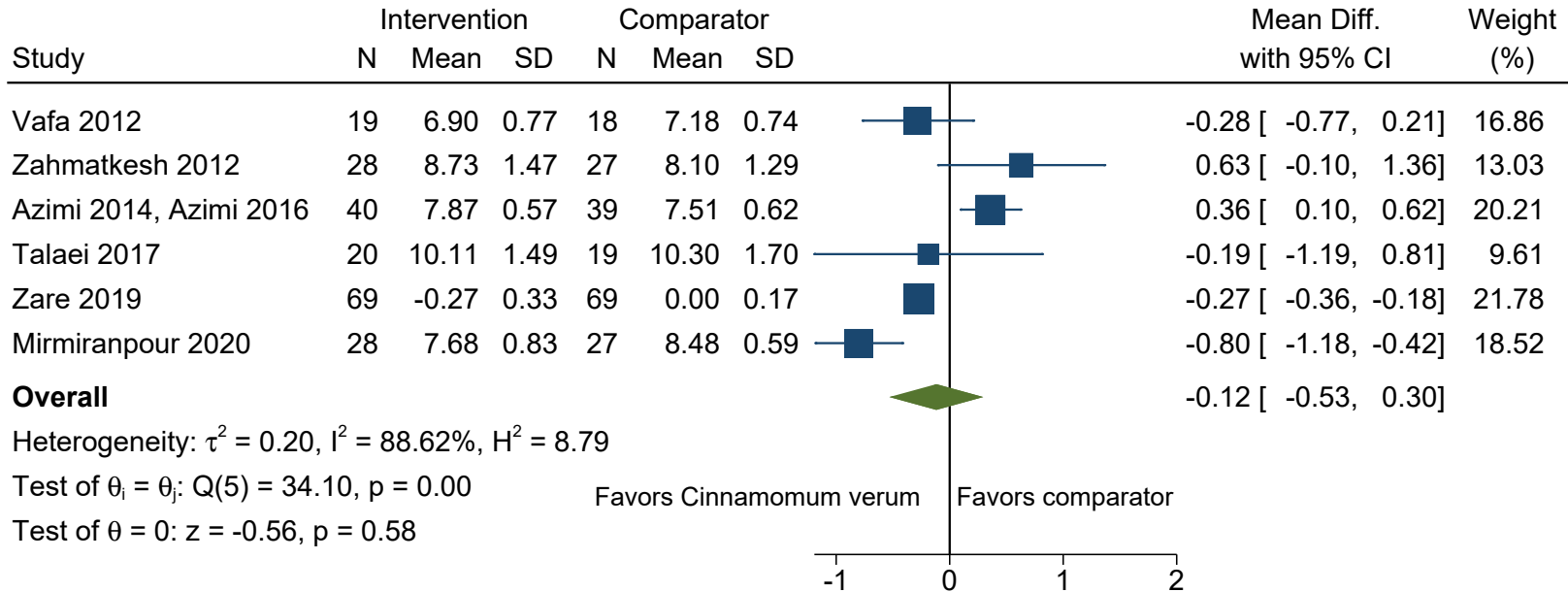

Supplement: Supplementary file 1 [file DataSheet1.zip › Supplementary Material/Forest and Funnel Plots/Cinnamomum verum/HbA1c.pdf]

# Cinnamomum verum - PPBG

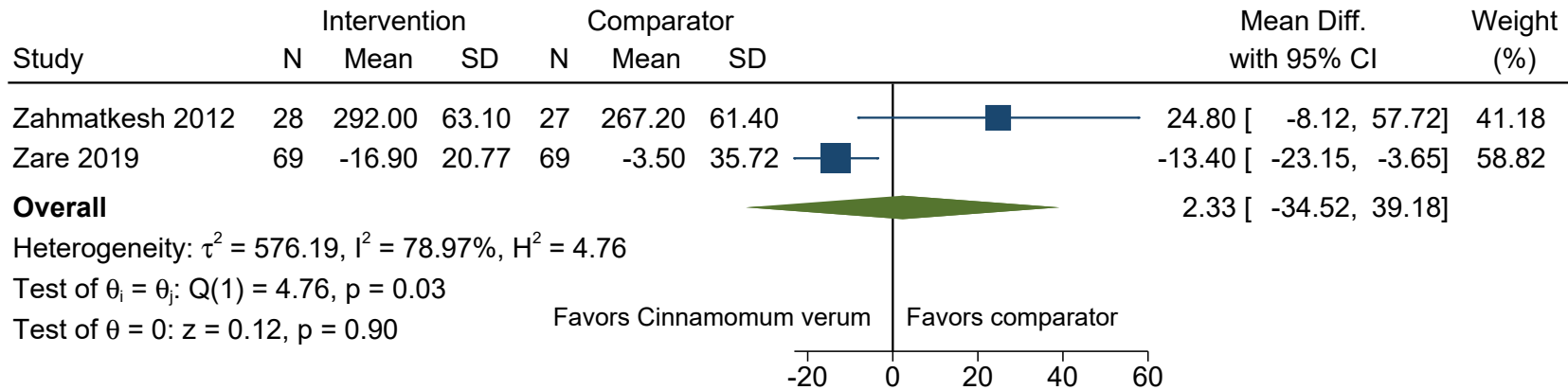

Random-effects REML model

Supplement: Supplementary file 1 [file DataSheet1.zip › Supplementary Material/Forest and Funnel Plots/Cinnamomum verum/PPBG.pdf]

# Cinnamomum verum - SBP

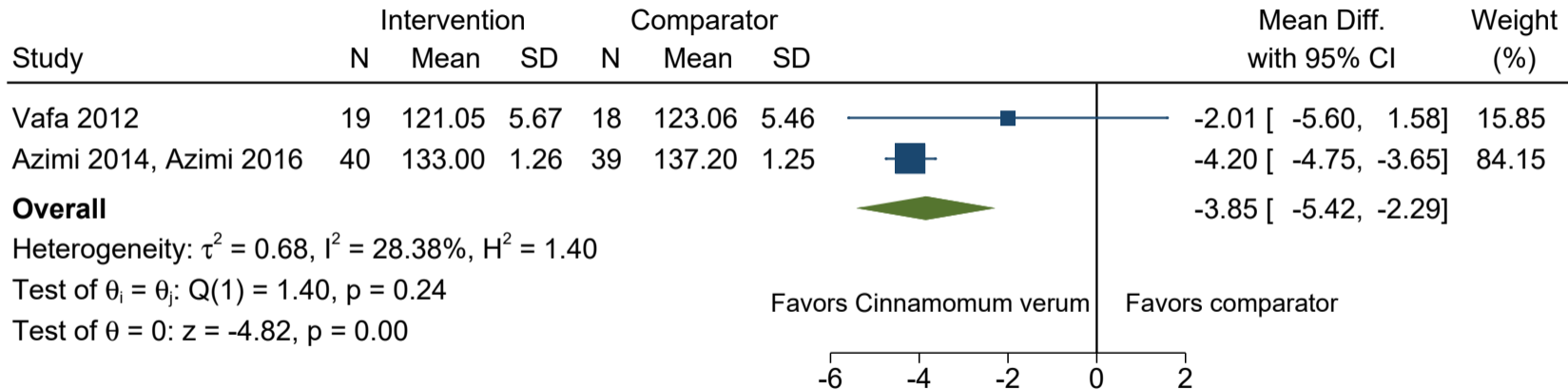

Random-effects REML model

Supplement: Supplementary file 1 [file DataSheet1.zip › Supplementary Material/Forest and Funnel Plots/Cinnamomum verum/SBP.pdf]

# Cinnamomum verum - LDL-C

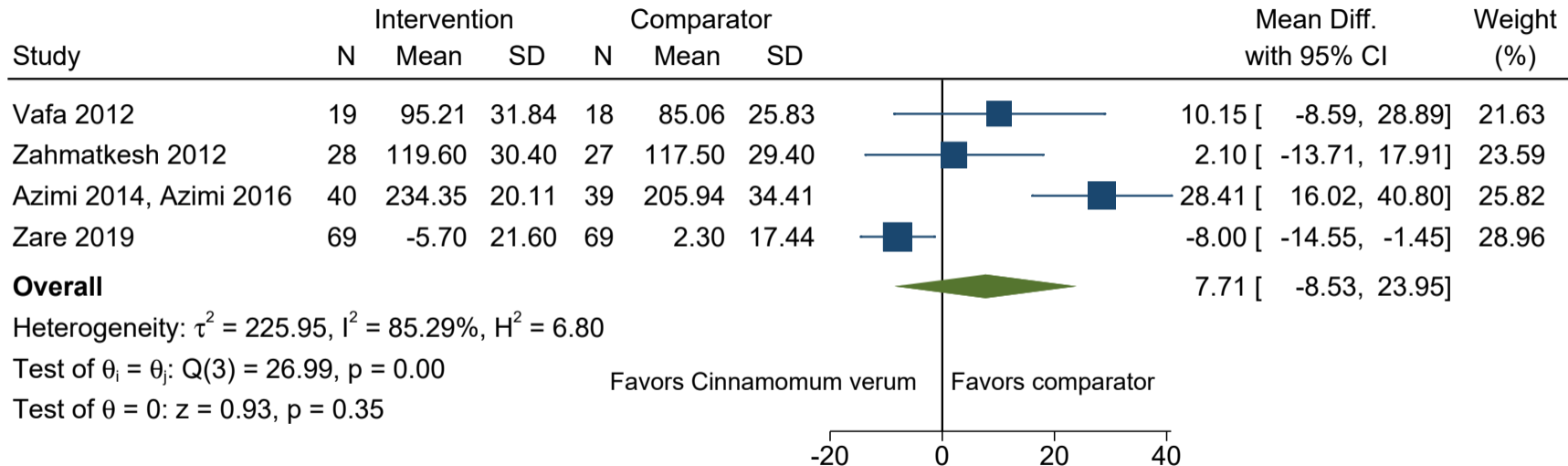

Random-effects REML model

Supplement: Supplementary file 1 [file DataSheet1.zip › Supplementary Material/Forest and Funnel Plots/Cinnamomum verum/LDL-C.pdf]

# Anethum graveolens - TG

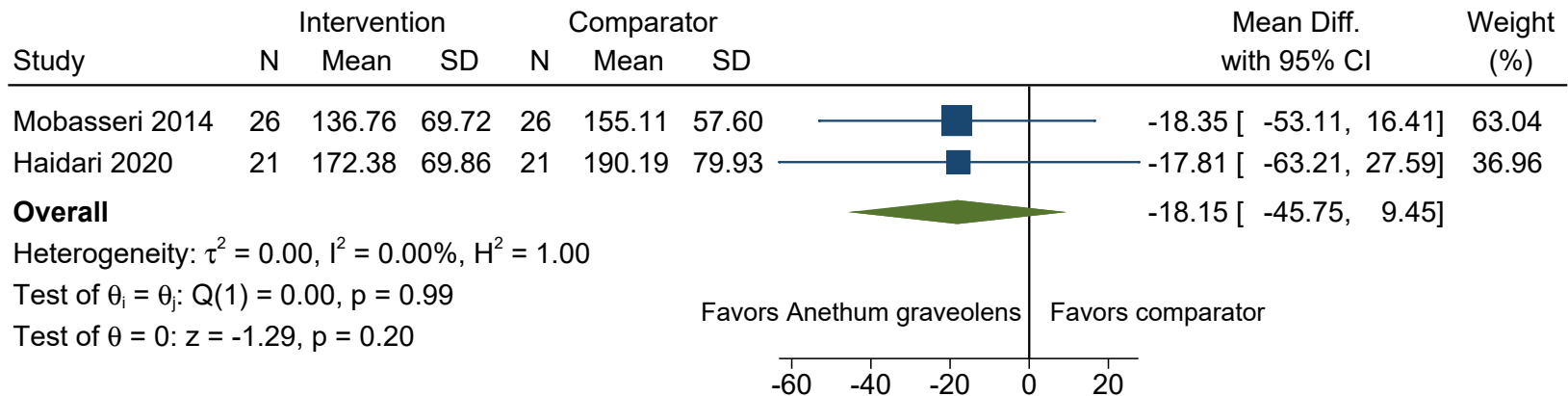

Random-effects REML model

Supplement: Supplementary file 1 [file DataSheet1.zip › Supplementary Material/Forest and Funnel Plots/Anethum graveolens/TG.pdf]

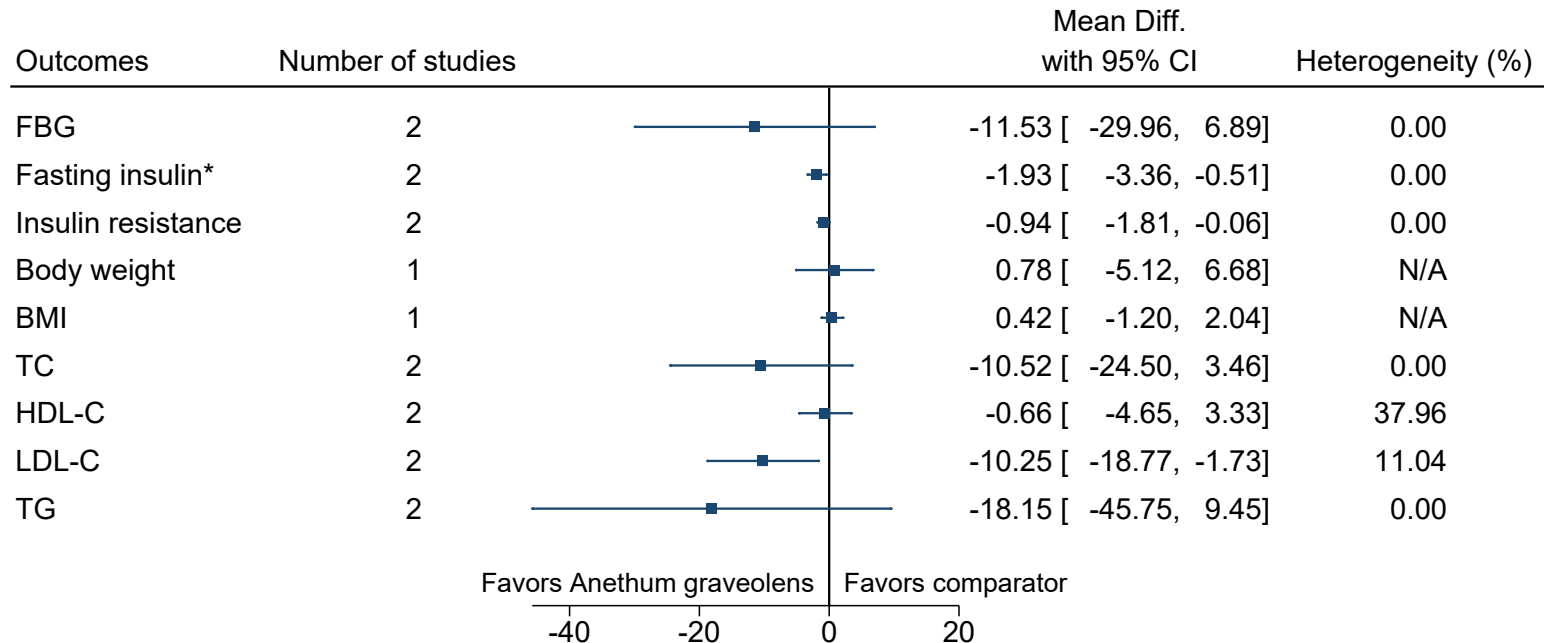

\*Favors intervention/comparator as insulin sensitizer

Supplement: Supplementary file 1 [file DataSheet1.zip › Supplementary Material/Forest and Funnel Plots/Anethum graveolens/Anethum graveolens.pdf]

# Anethum graveolens - TC

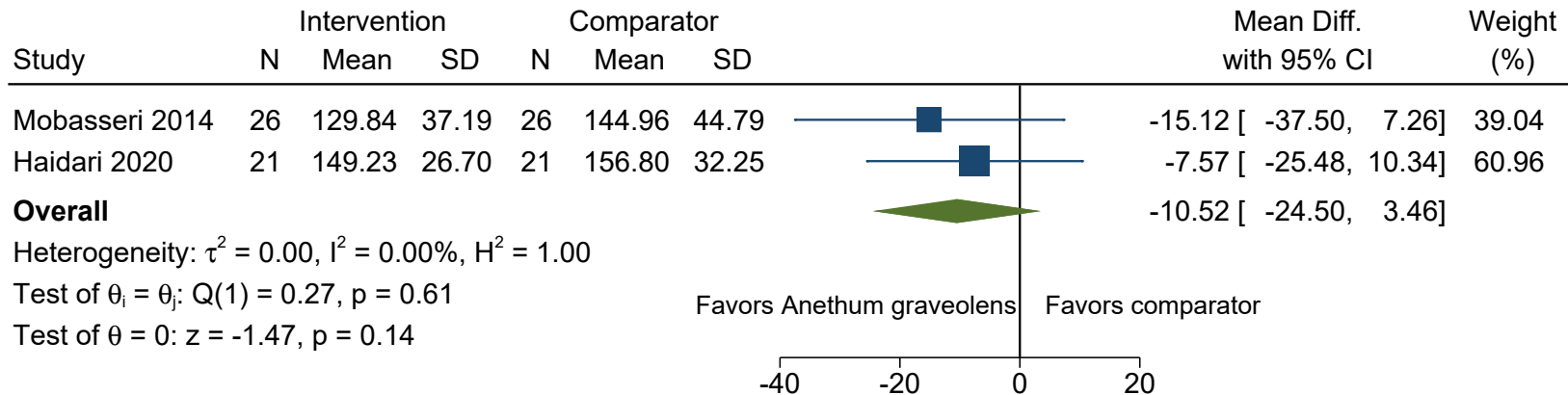

Random-effects REML model

Supplement: Supplementary file 1 [file DataSheet1.zip › Supplementary Material/Forest and Funnel Plots/Anethum graveolens/TC.pdf]

# Anethum graveolens - FBG

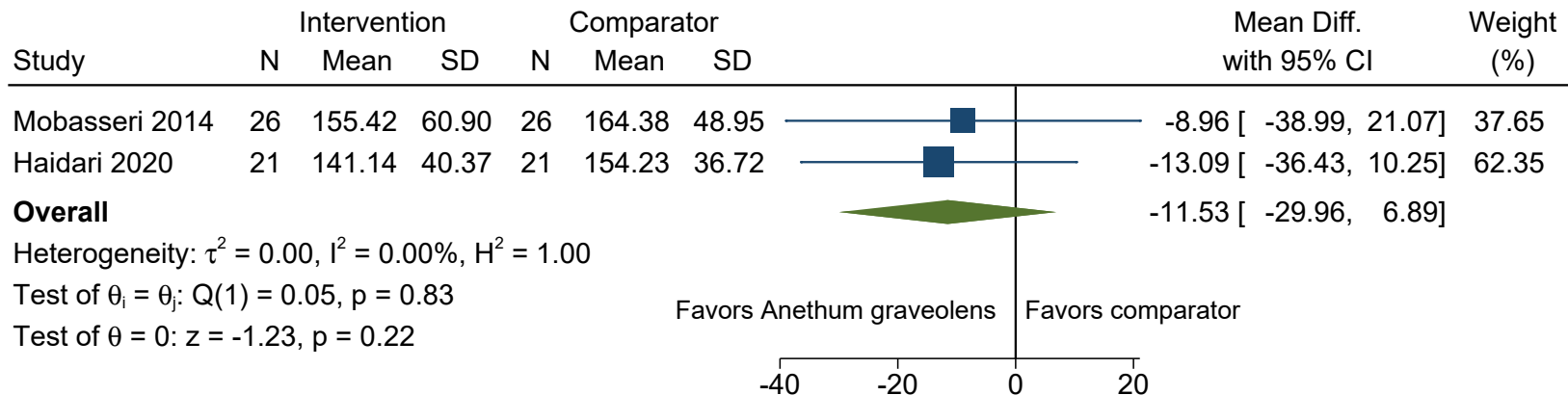

Random-effects REML model

Supplement: Supplementary file 1 [file DataSheet1.zip › Supplementary Material/Forest and Funnel Plots/Anethum graveolens/FBG.pdf]

# Anethum graveolens - HDL-C

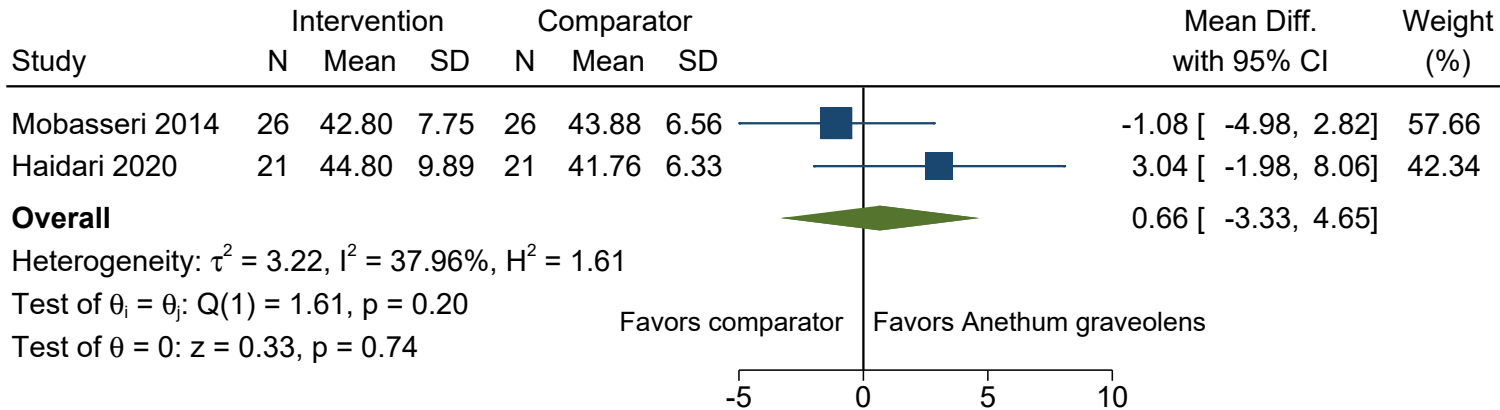

Random-effects REML model

Supplement: Supplementary file 1 [file DataSheet1.zip › Supplementary Material/Forest and Funnel Plots/Anethum graveolens/HDL-C.pdf]

# Anethum graveolens - Insulin resistance

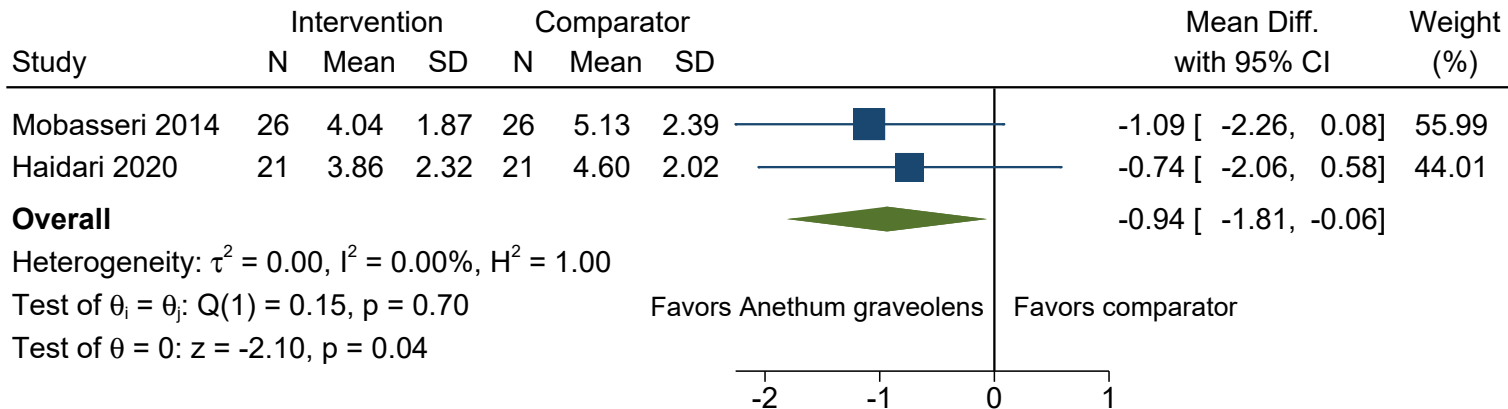

Random-effects REML model

Supplement: Supplementary file 1 [file DataSheet1.zip › Supplementary Material/Forest and Funnel Plots/Anethum graveolens/Insulin resistance.pdf]

# Anethum graveolens - Fasting insulin

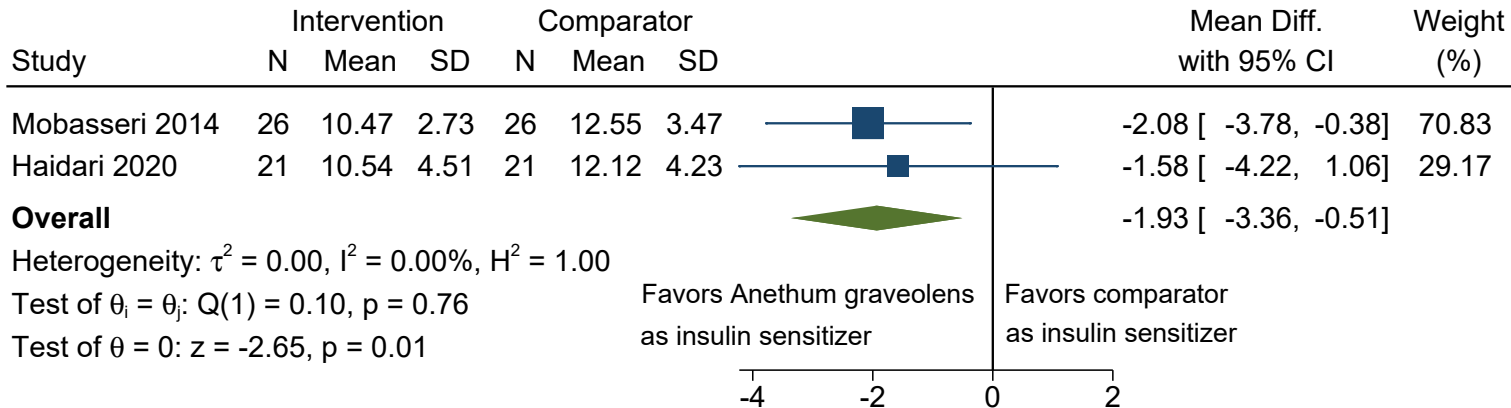

Random-effects REML model

Supplement: Supplementary file 1 [file DataSheet1.zip › Supplementary Material/Forest and Funnel Plots/Anethum graveolens/Fasting insulin.pdf]

# Anethum graveolens - LDL-C

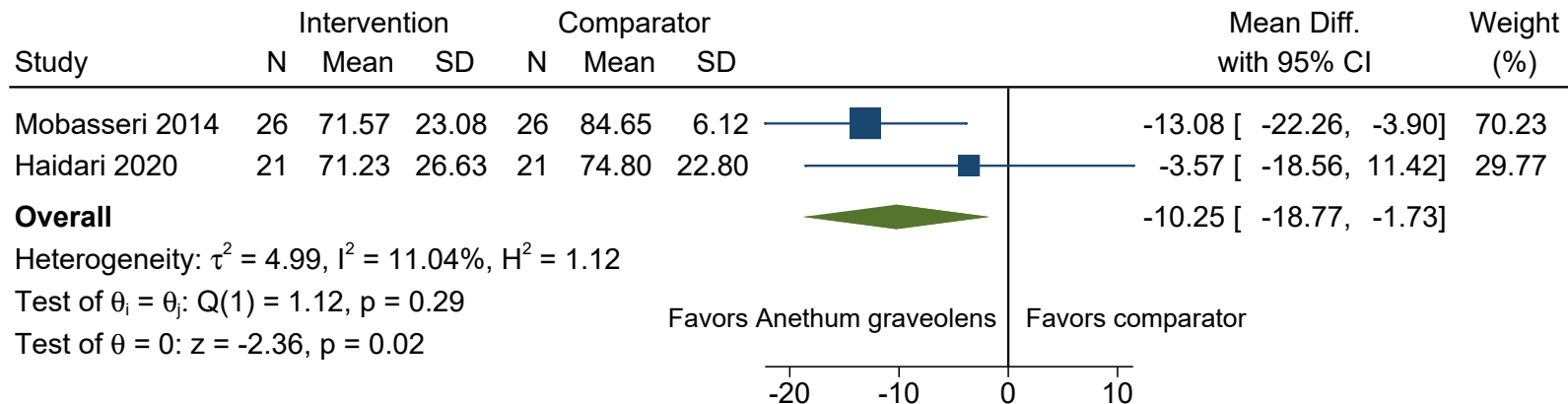

Random-effects REML model

Supplement: Supplementary file 1 [file DataSheet1.zip › Supplementary Material/Forest and Funnel Plots/Anethum graveolens/LDL-C.pdf]

# Cyamopsis tetragonoloba - TG

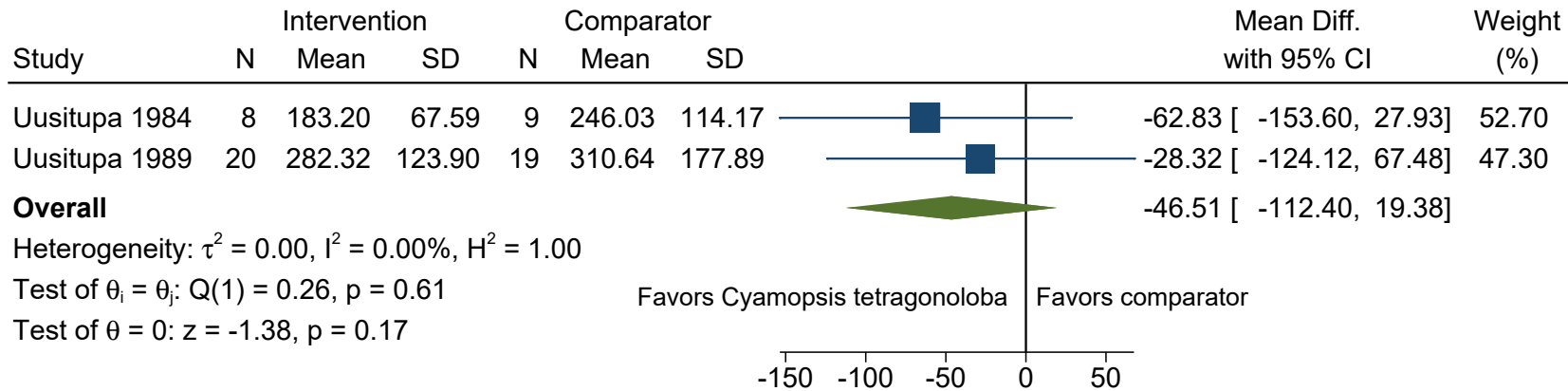

Random-effects REML model

Supplement: Supplementary file 1 [file DataSheet1.zip › Supplementary Material/Forest and Funnel Plots/Cyamopsis tetragonoloba/TG.pdf]

# Cyamopsis tetragonoloba - TC

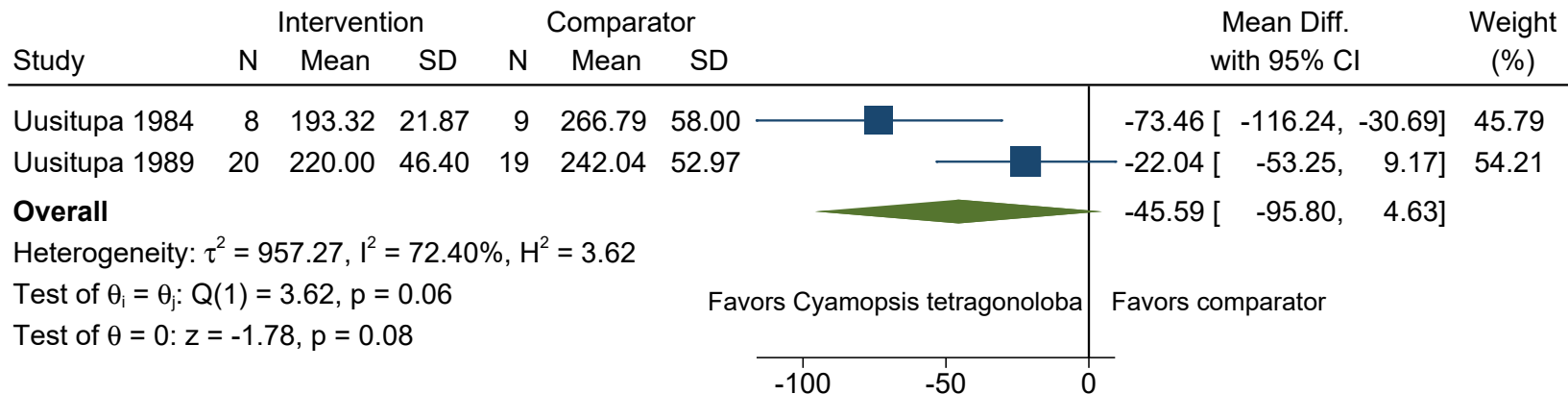

Random-effects REML model

Supplement: Supplementary file 1 [file DataSheet1.zip › Supplementary Material/Forest and Funnel Plots/Cyamopsis tetragonoloba/TC.pdf]

# Cyamopsis tetragonoloba - FBG

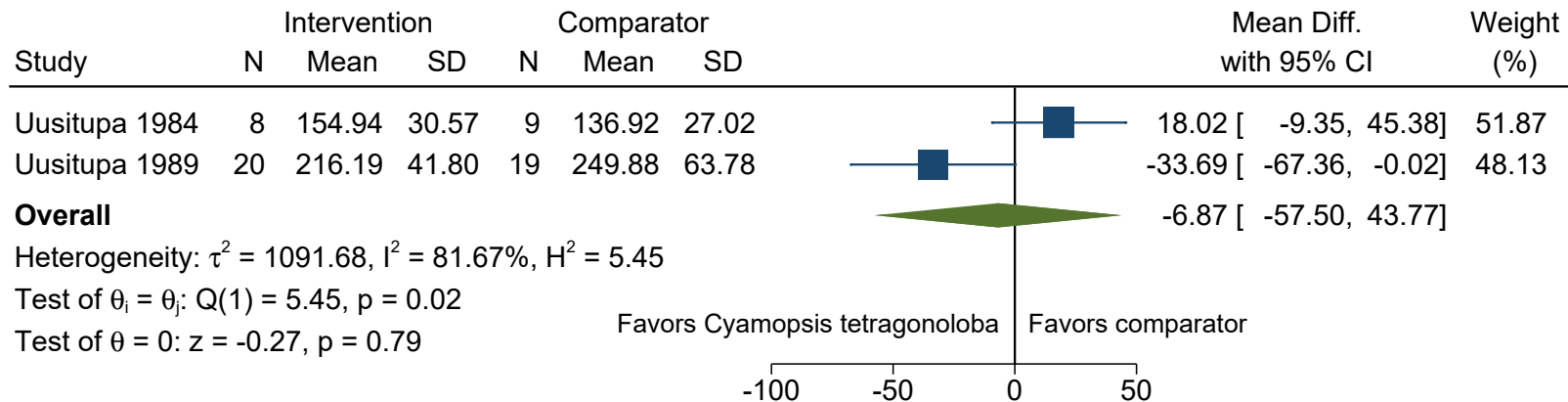

Random-effects REML model

Supplement: Supplementary file 1 [file DataSheet1.zip › Supplementary Material/Forest and Funnel Plots/Cyamopsis tetragonoloba/FBG.pdf]

# Cyamopsis tetragonoloba - HDL-C

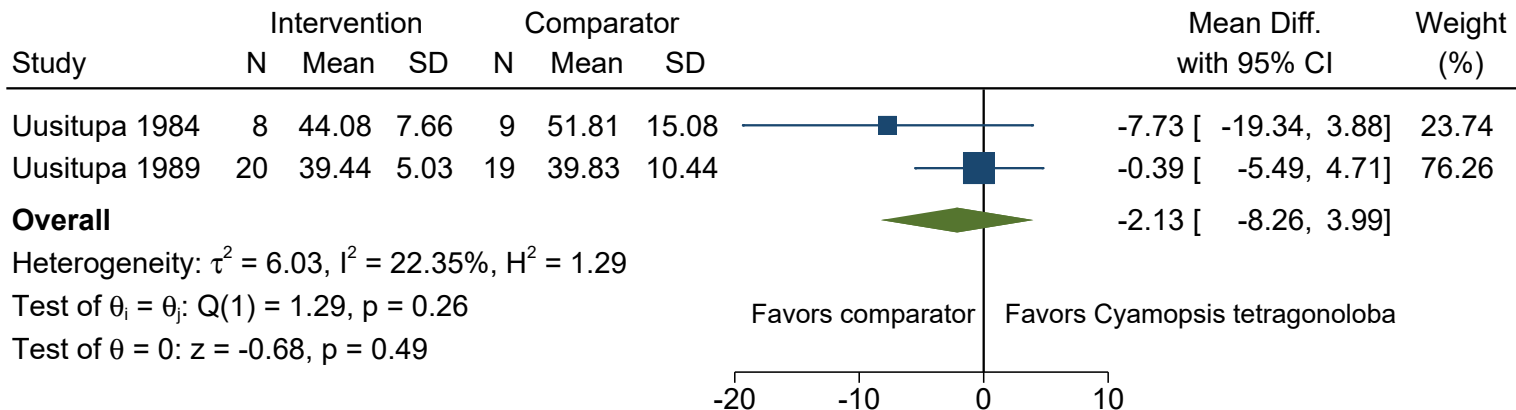

Random-effects REML model

Supplement: Supplementary file 1 [file DataSheet1.zip › Supplementary Material/Forest and Funnel Plots/Cyamopsis tetragonoloba/HDL-C.pdf]

# Cyamopsis tetragonoloba - DBP

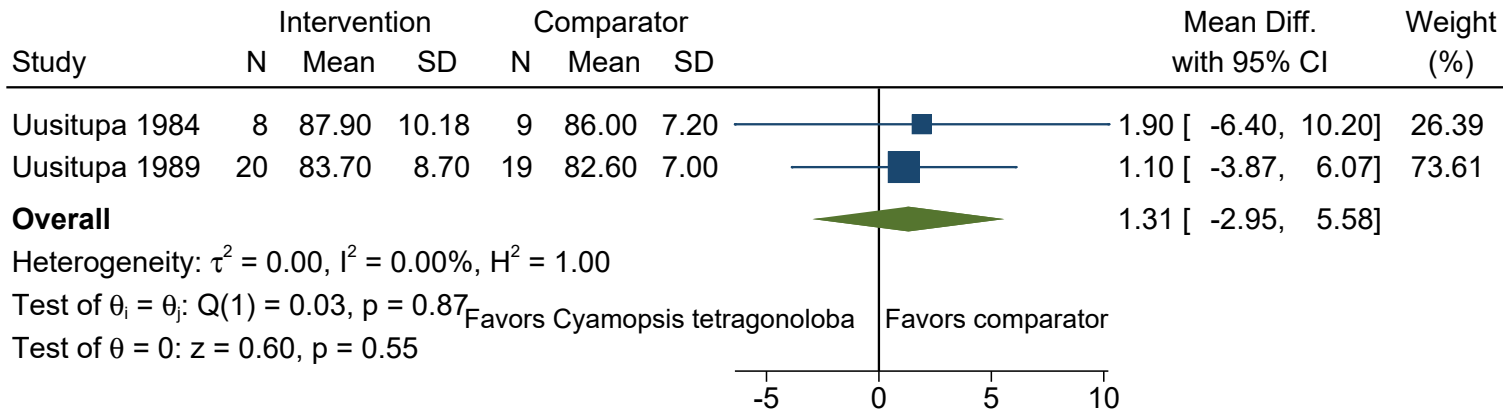

Random-effects REML model

Supplement: Supplementary file 1 [file DataSheet1.zip › Supplementary Material/Forest and Funnel Plots/Cyamopsis tetragonoloba/DBP.pdf]

# Cyamopsis tetragonoloba - Body weight

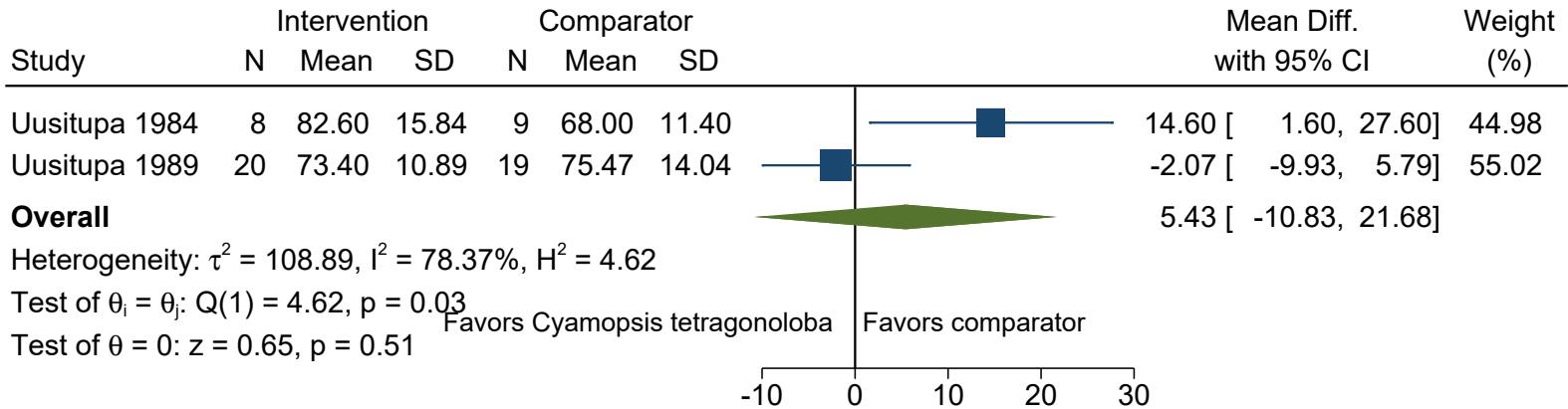

Random-effects REML model

Supplement: Supplementary file 1 [file DataSheet1.zip › Supplementary Material/Forest and Funnel Plots/Cyamopsis tetragonoloba/Body weight.pdf]

# Cyamopsis tetragonoloba - SBP

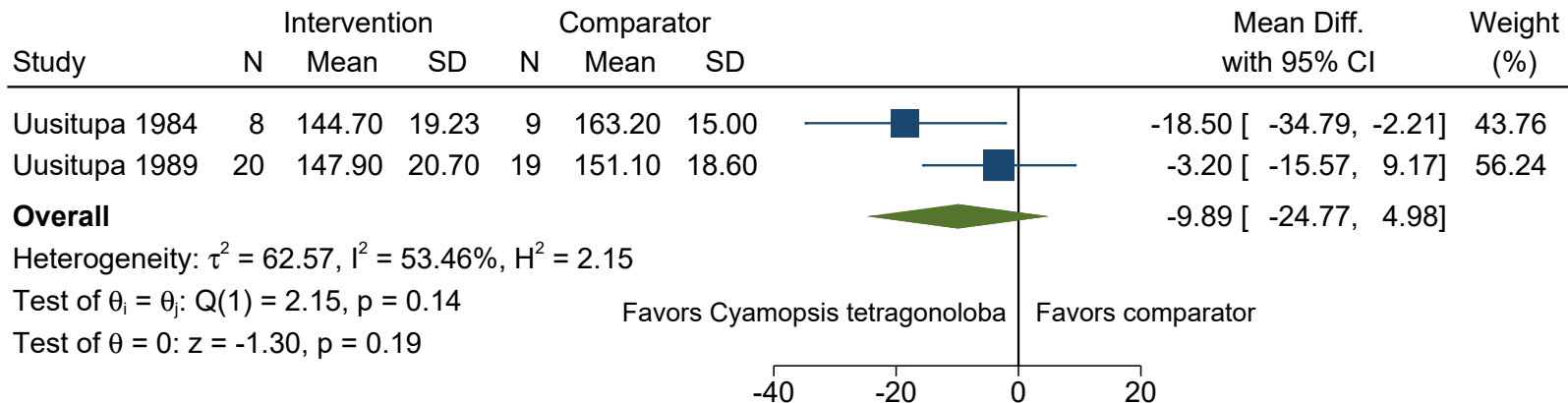

Random-effects REML model

Supplement: Supplementary file 1 [file DataSheet1.zip › Supplementary Material/Forest and Funnel Plots/Cyamopsis tetragonoloba/SBP.pdf]

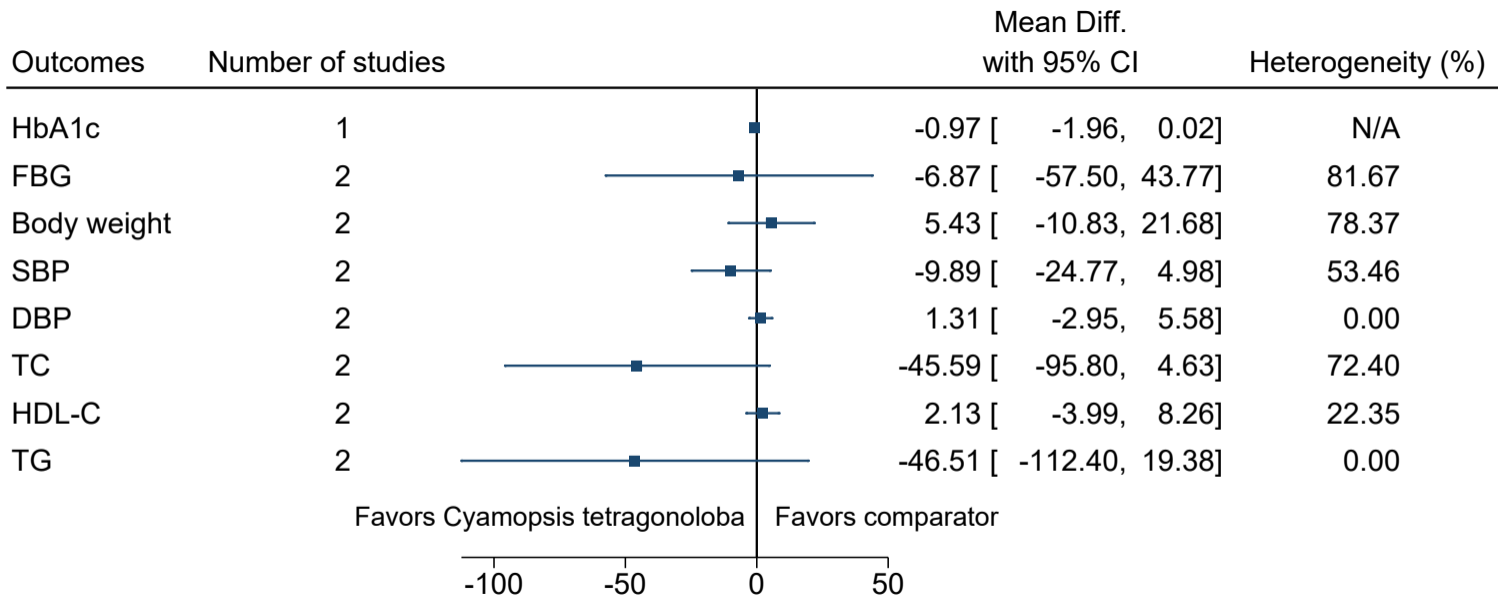

Supplement: Supplementary file 1 [file DataSheet1.zip › Supplementary Material/Forest and Funnel Plots/Cyamopsis tetragonoloba/Cyamopsis tetragonoloba.pdf]

# Momordica charantia - Waist circumference

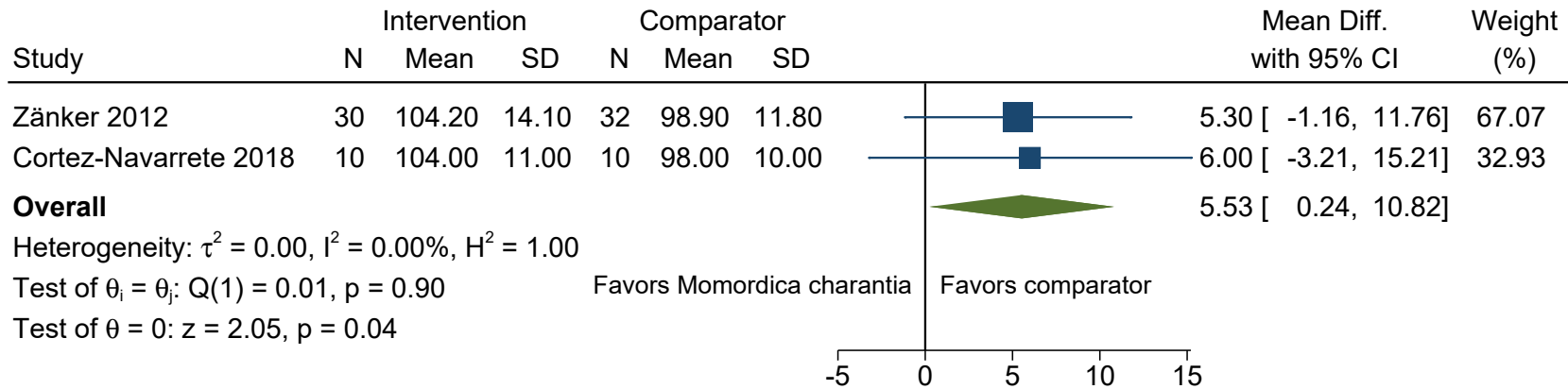

Random-effects REML model

Supplement: Supplementary file 1 [file DataSheet1.zip › Supplementary Material/Forest and Funnel Plots/Momordica charantia/Waist circumference.pdf]

# Momordica charantia - TG

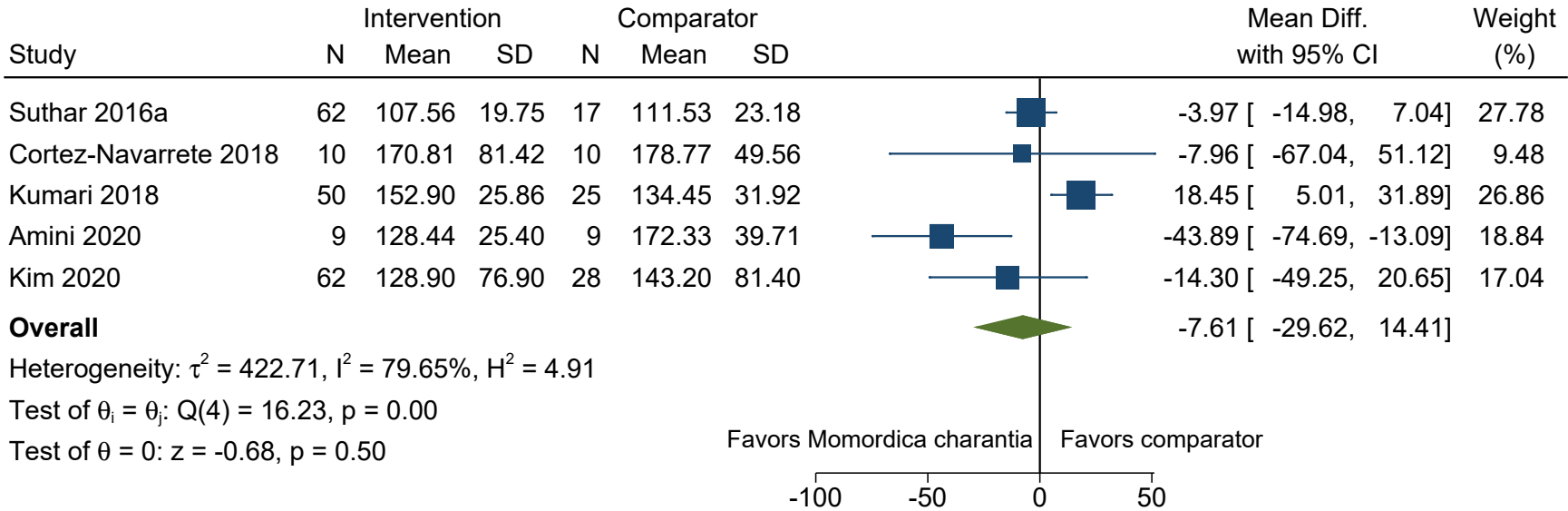

Supplement: Supplementary file 1 [file DataSheet1.zip › Supplementary Material/Forest and Funnel Plots/Momordica charantia/TG.pdf]

# Momordica charantia - BMI

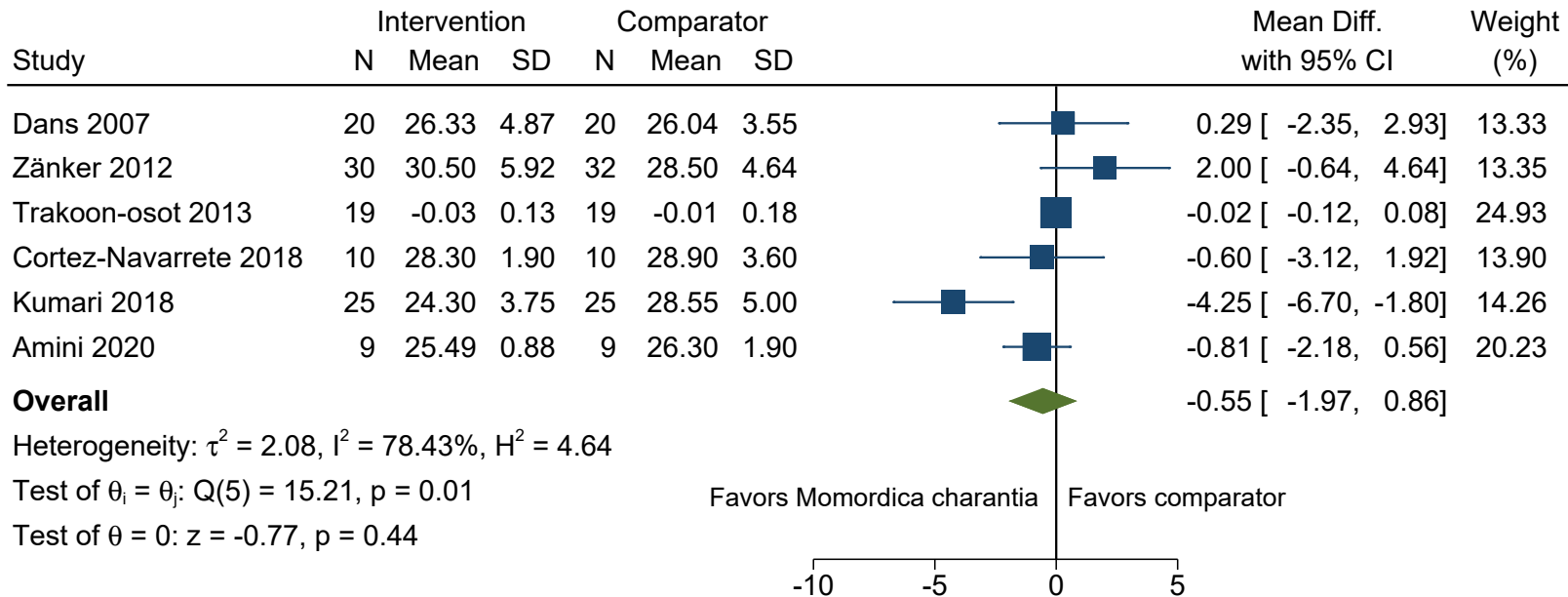

Random-effects REML model

Supplement: Supplementary file 1 [file DataSheet1.zip › Supplementary Material/Forest and Funnel Plots/Momordica charantia/BMI.pdf]

# Momordica charantia - TC

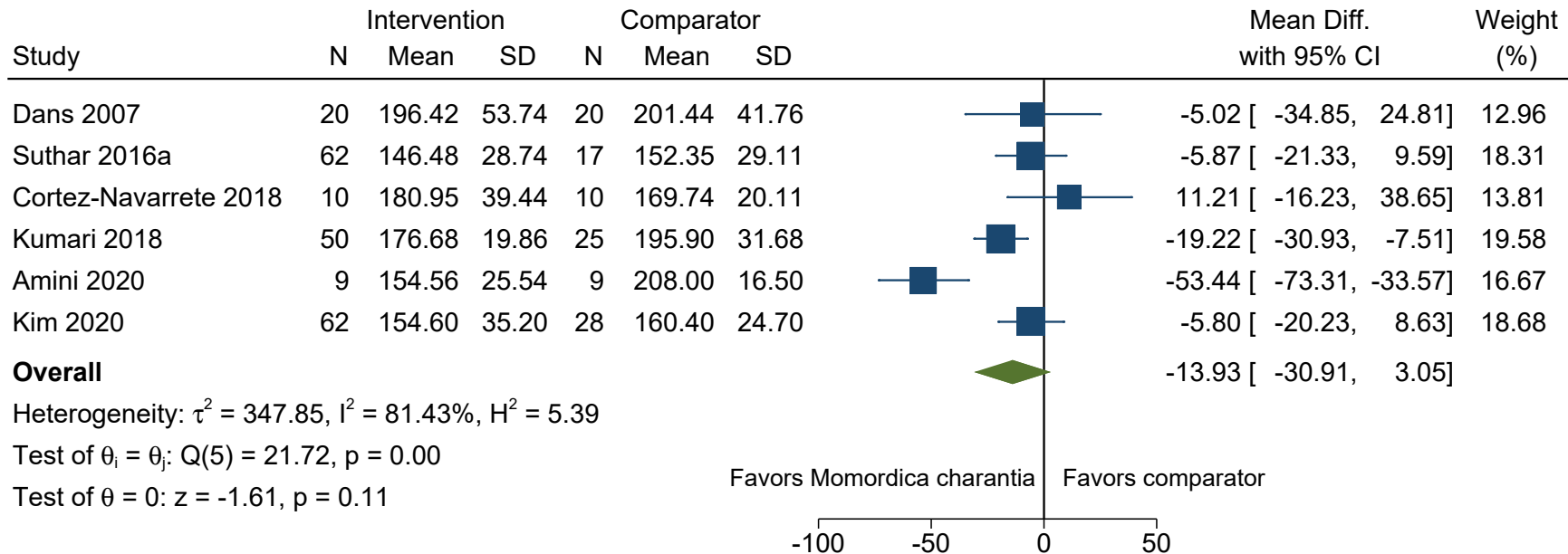

Supplement: Supplementary file 1 [file DataSheet1.zip › Supplementary Material/Forest and Funnel Plots/Momordica charantia/TC.pdf]

# Momordica charantia - FBG

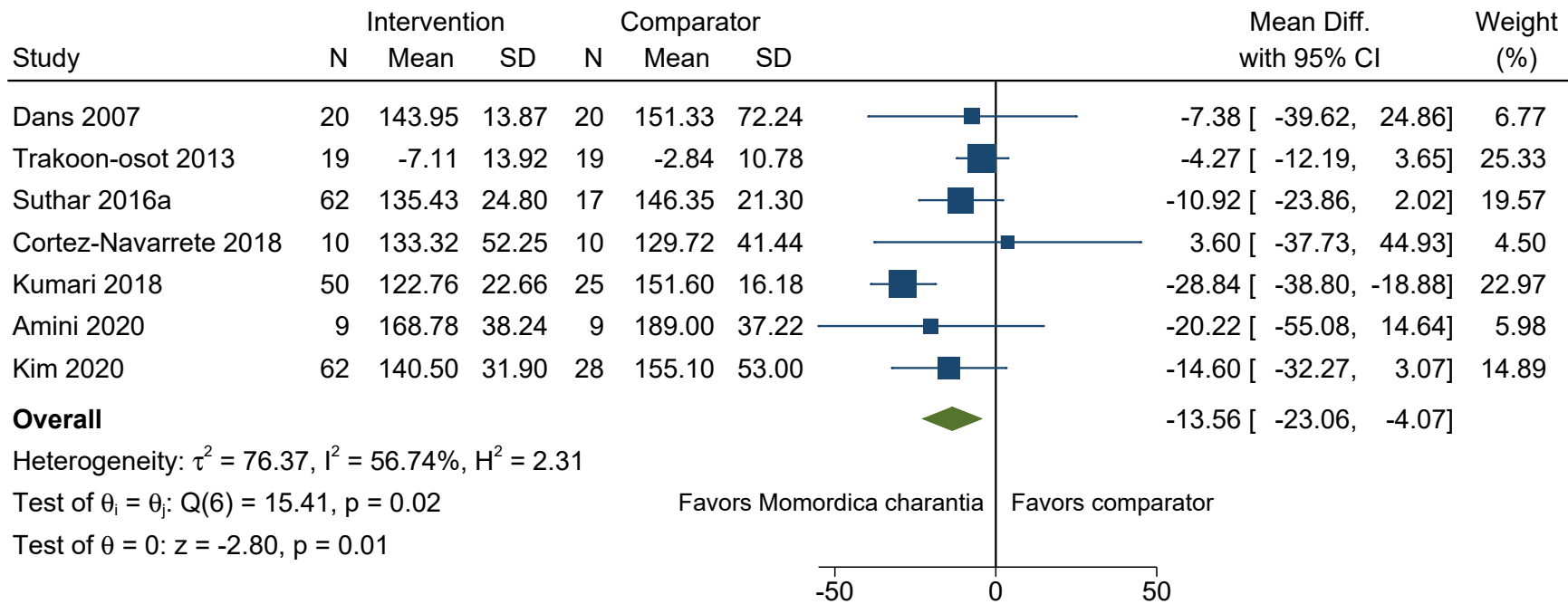

Supplement: Supplementary file 1 [file DataSheet1.zip › Supplementary Material/Forest and Funnel Plots/Momordica charantia/FBG.pdf]

# Momordica charantia - HDL-C

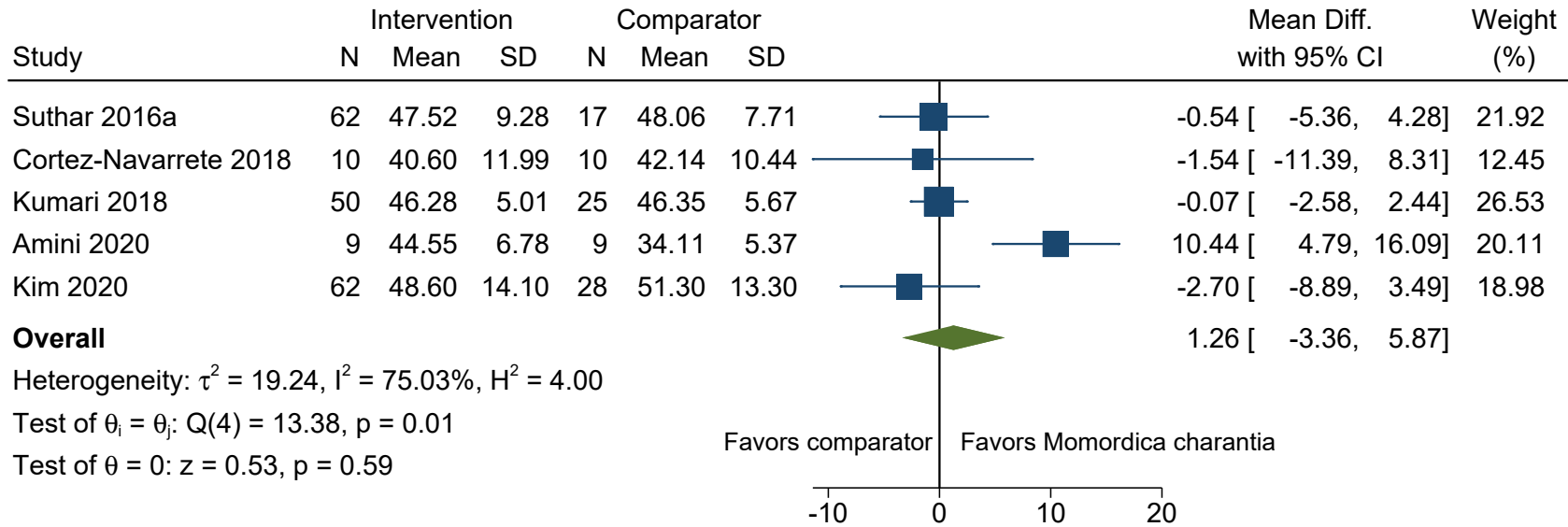

Supplement: Supplementary file 1 [file DataSheet1.zip › Supplementary Material/Forest and Funnel Plots/Momordica charantia/HDL-C.pdf]

# Momordica charantia - DBP

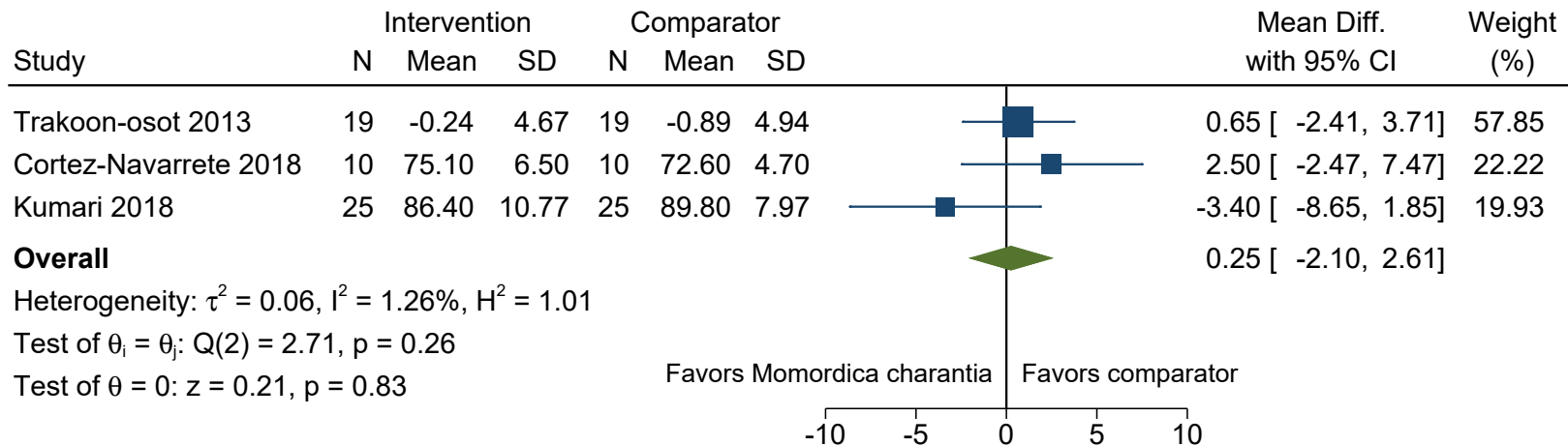

Random-effects REML model

Supplement: Supplementary file 1 [file DataSheet1.zip › Supplementary Material/Forest and Funnel Plots/Momordica charantia/DBP.pdf]

# Momordica charantia - Body weight

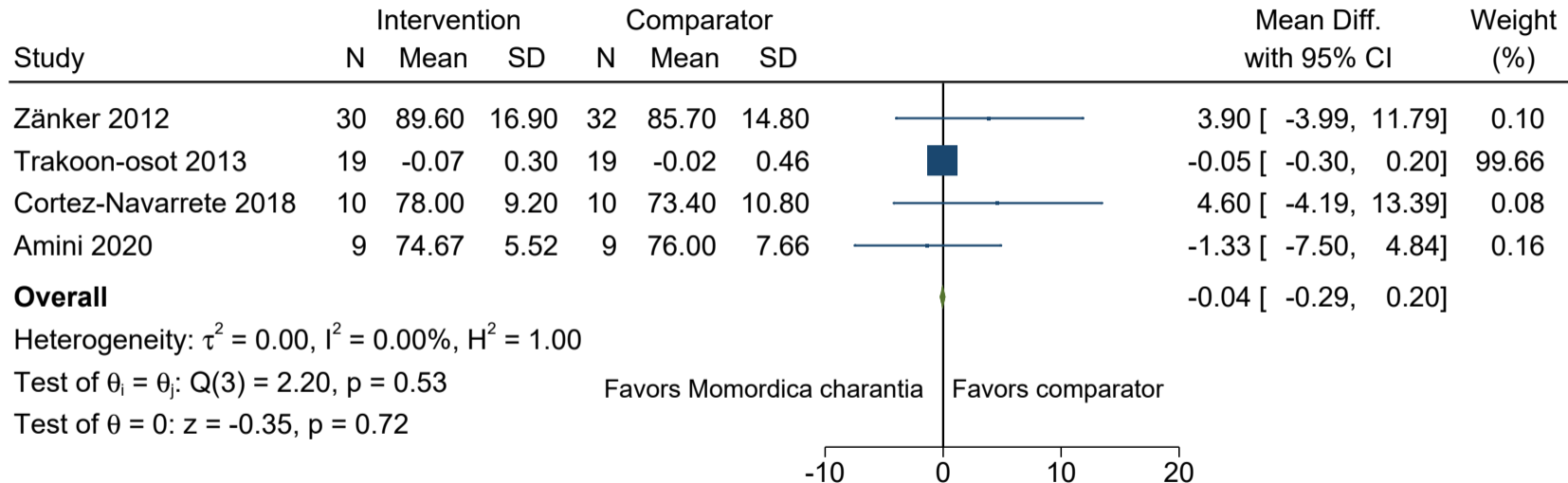

Random-effects REML model

Supplement: Supplementary file 1 [file DataSheet1.zip › Supplementary Material/Forest and Funnel Plots/Momordica charantia/Body weight.pdf]

# Momordica charantia - HbA1c

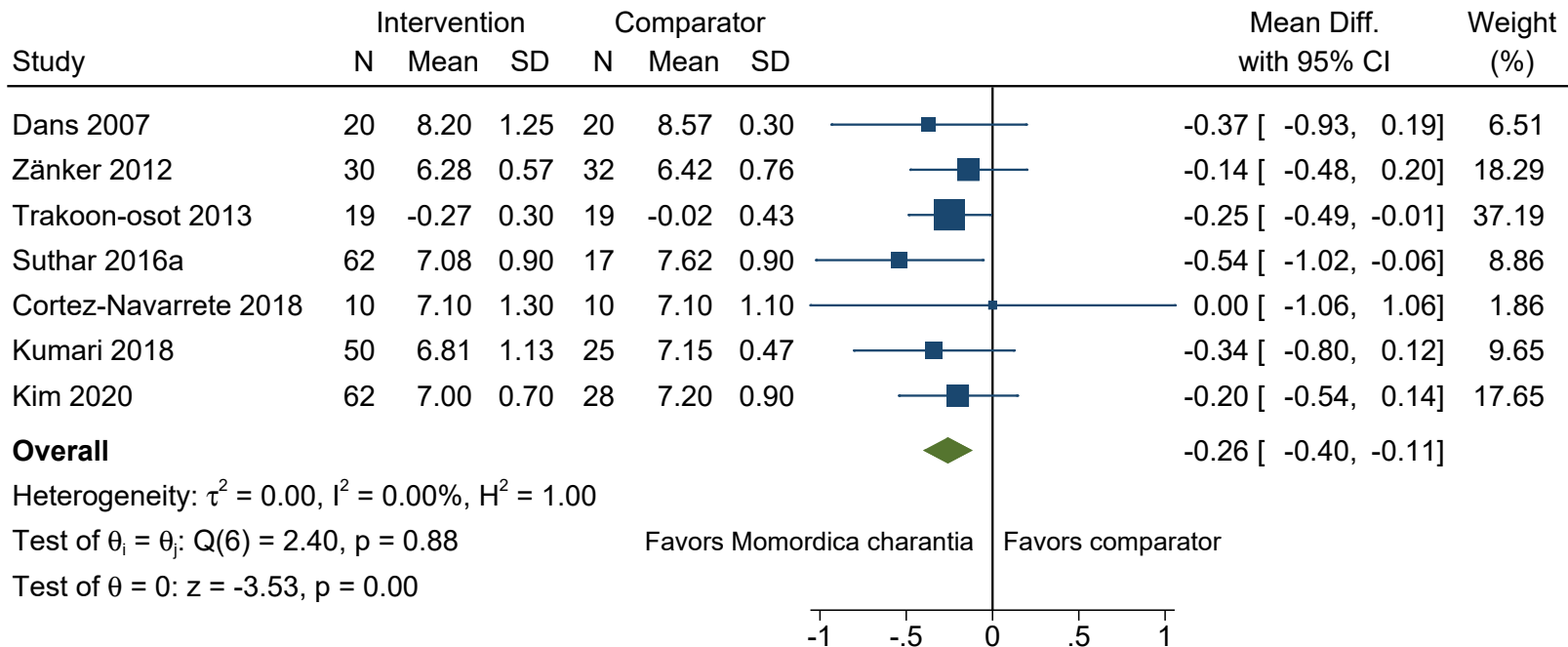

Supplement: Supplementary file 1 [file DataSheet1.zip › Supplementary Material/Forest and Funnel Plots/Momordica charantia/HbA1c.pdf]

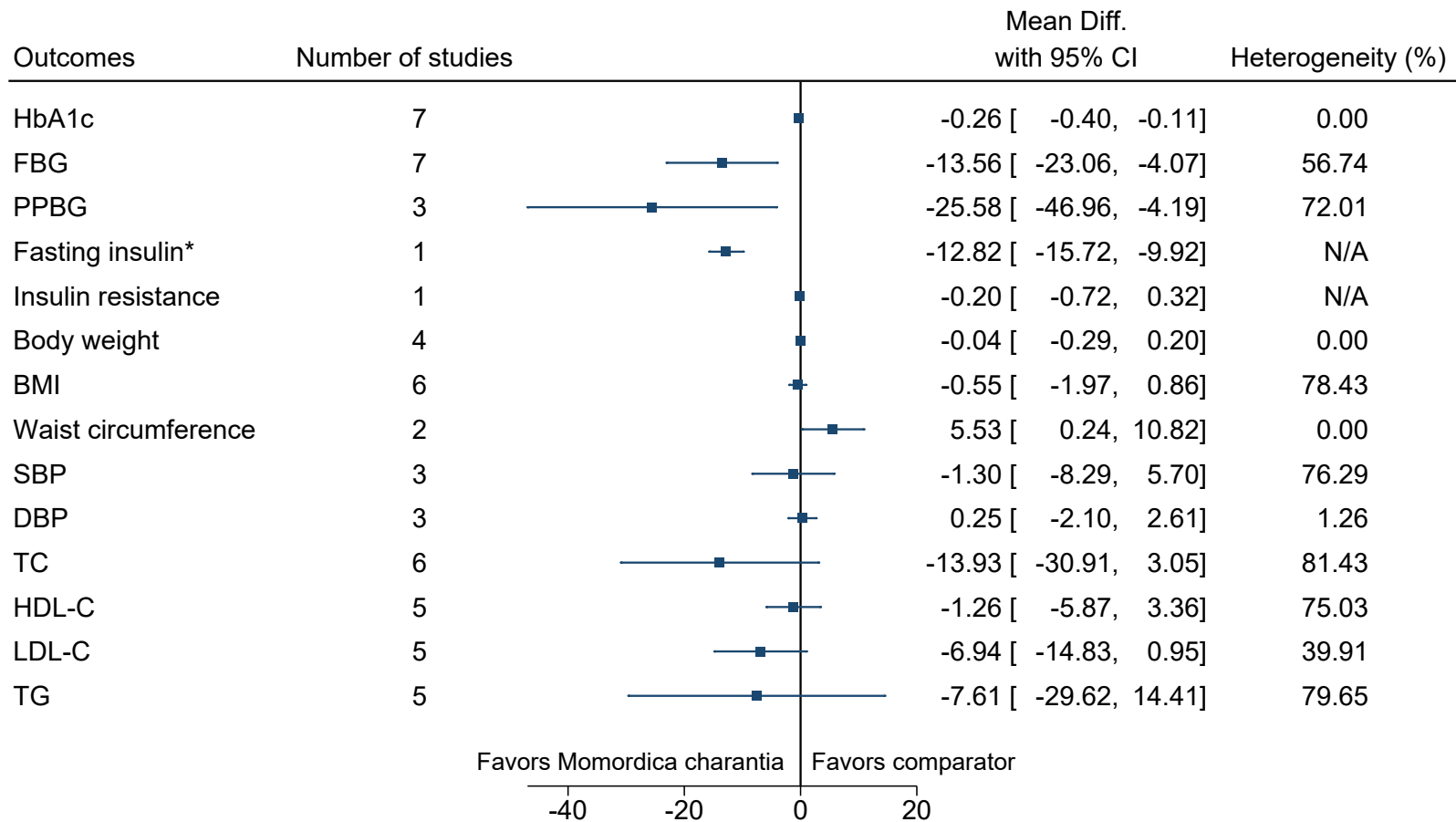

\*Favors intervention/comparator as insulin sensitizer

Supplement: Supplementary file 1 [file DataSheet1.zip › Supplementary Material/Forest and Funnel Plots/Momordica charantia/Momordica charantia.pdf]

# Momordica charantia - PPBG

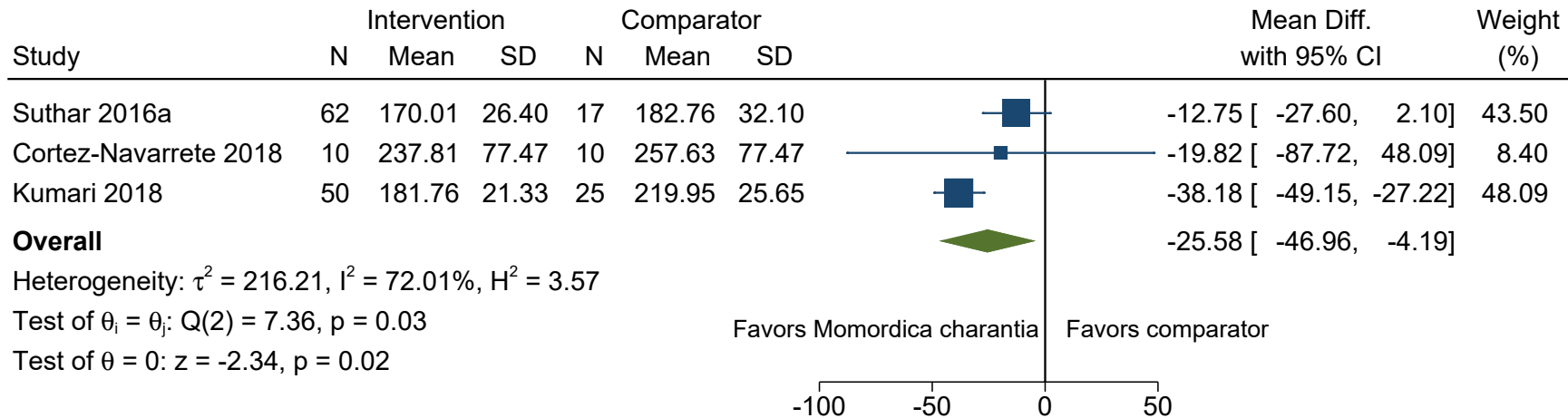

Random-effects REML model

Supplement: Supplementary file 1 [file DataSheet1.zip › Supplementary Material/Forest and Funnel Plots/Momordica charantia/PPBG.pdf]

# Momordica charantia - SBP

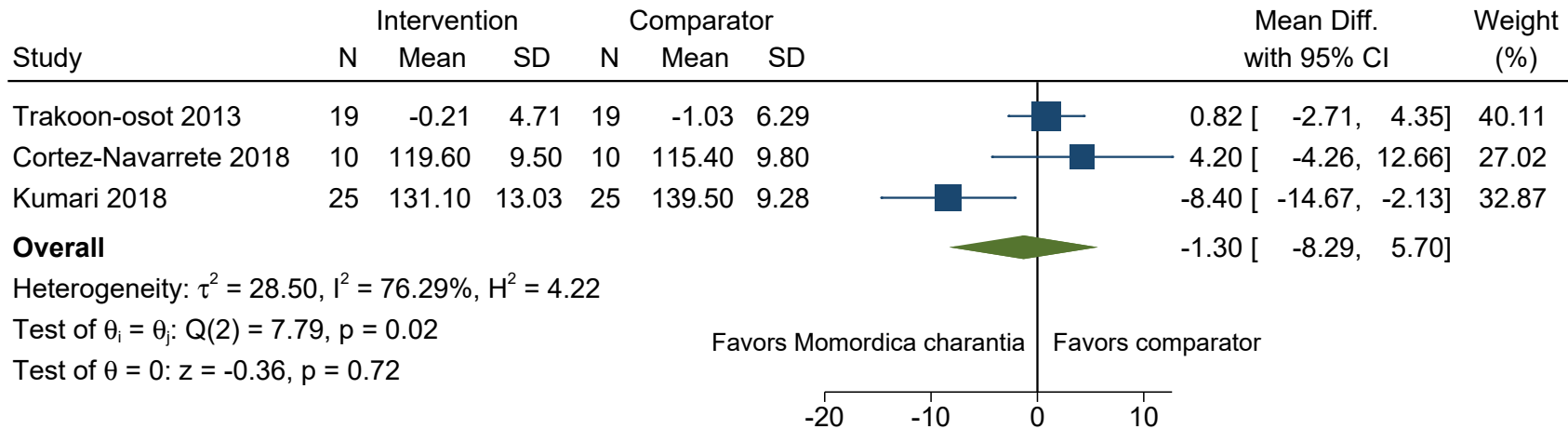

Random-effects REML model

Supplement: Supplementary file 1 [file DataSheet1.zip › Supplementary Material/Forest and Funnel Plots/Momordica charantia/SBP.pdf]

# Momordica charantia - LDL-C

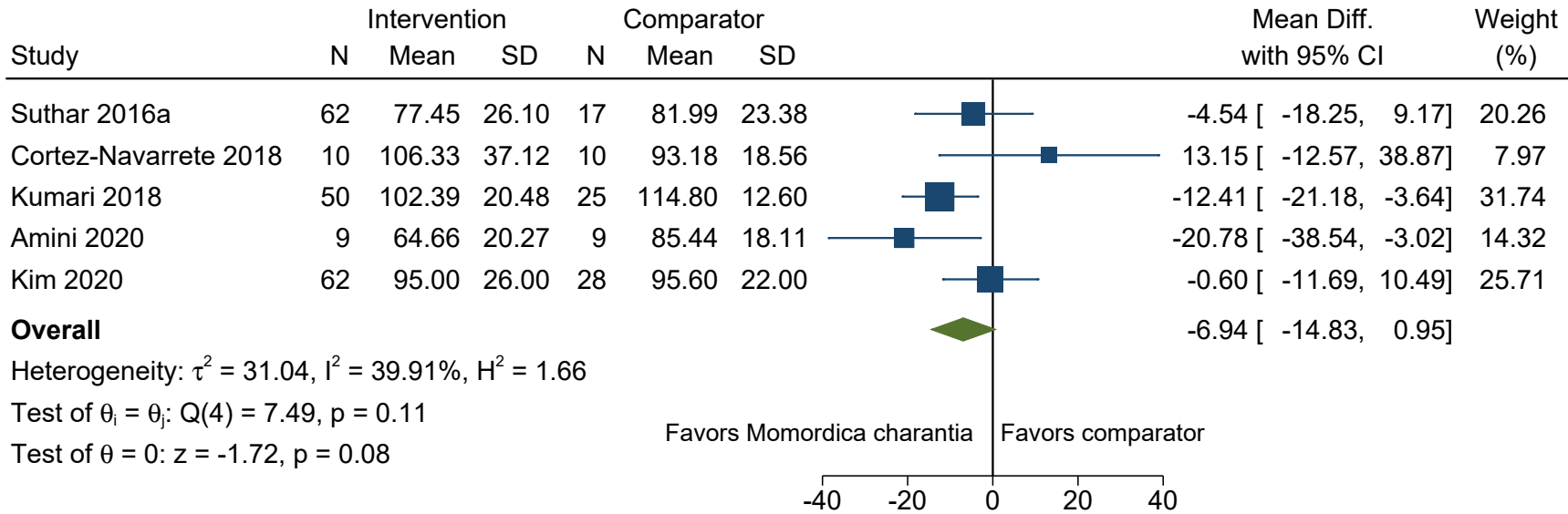

Supplement: Supplementary file 1 [file DataSheet1.zip › Supplementary Material/Forest and Funnel Plots/Momordica charantia/LDL-C.pdf]

# Citrullus colocynthis - FBG

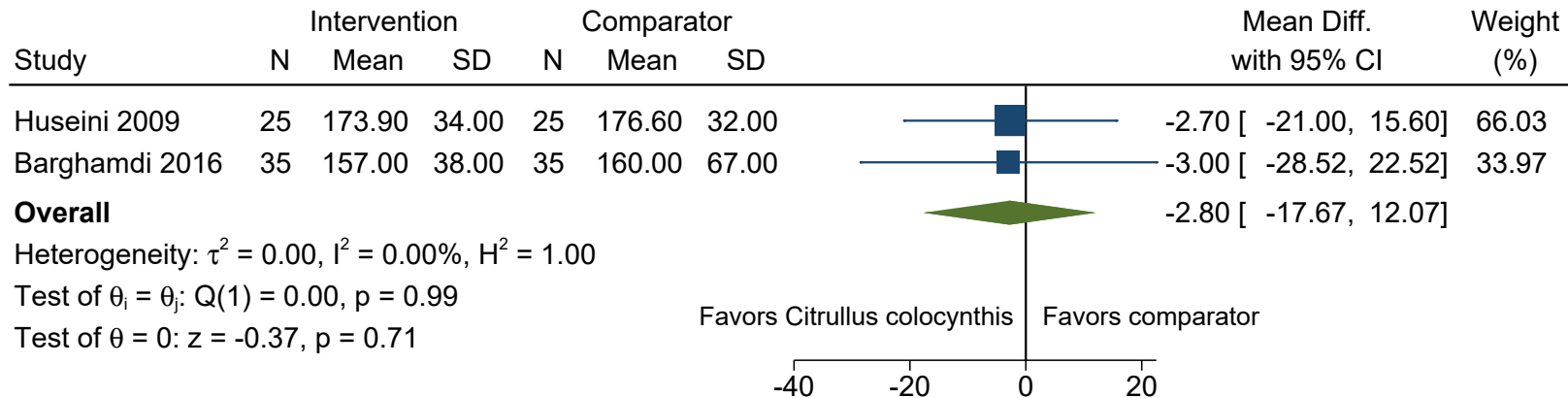

Random-effects REML model

Supplement: Supplementary file 1 [file DataSheet1.zip › Supplementary Material/Forest and Funnel Plots/Citrullus colocynthis/FBG.pdf]

# Citrullus colocynthis - HbA1c

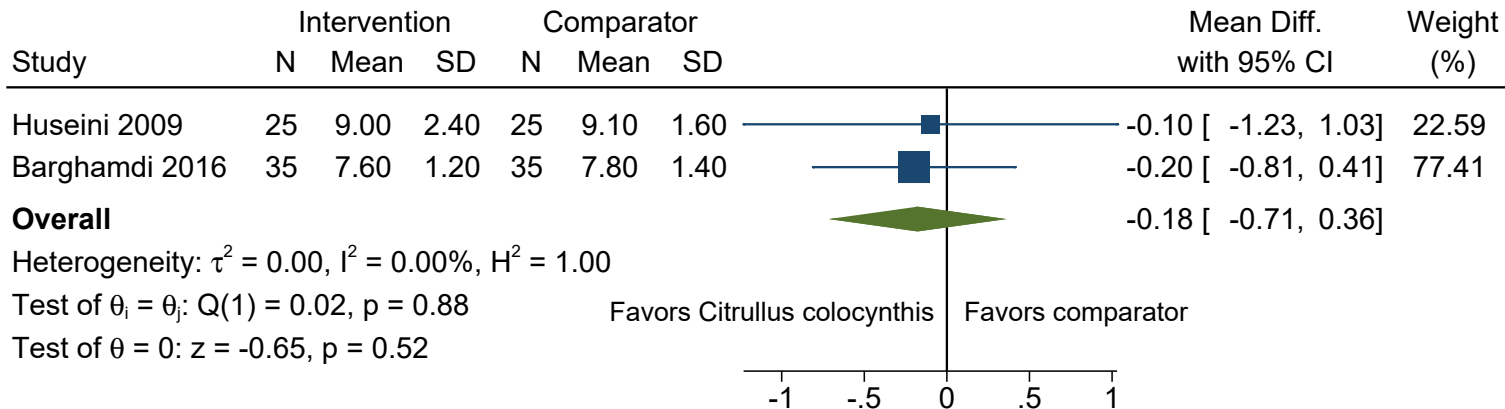

Random-effects REML model

Supplement: Supplementary file 1 [file DataSheet1.zip › Supplementary Material/Forest and Funnel Plots/Citrullus colocynthis/HbA1c.pdf]

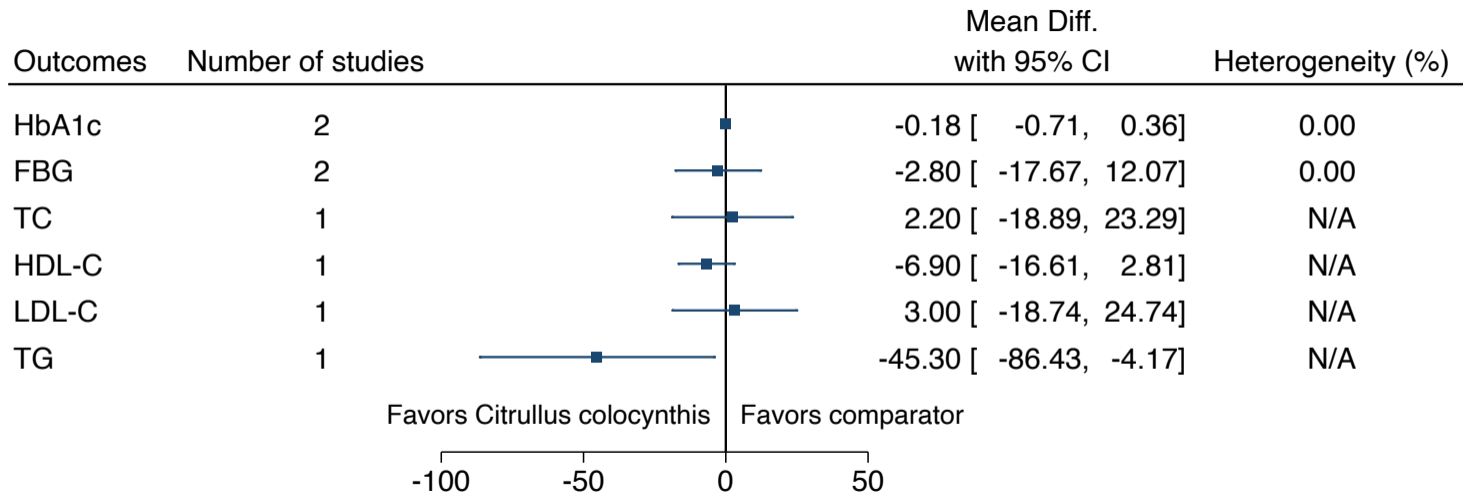

Supplement: Supplementary file 1 [file DataSheet1.zip › Supplementary Material/Forest and Funnel Plots/Citrullus colocynthis/Citrullus colocynthis.pdf]

# Crocus sativus - Waist circumference

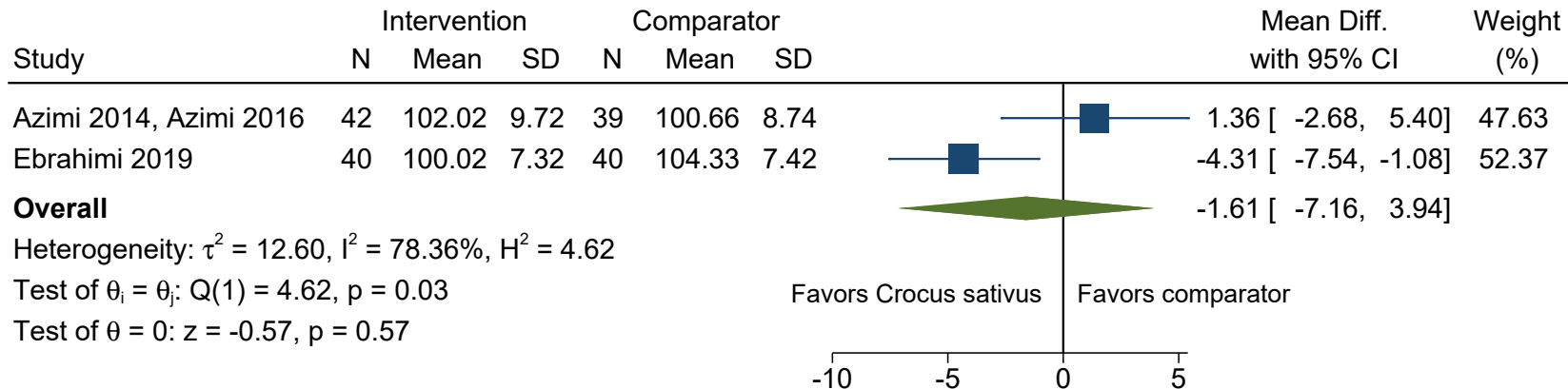

Random-effects REML model

Supplement: Supplementary file 1 [file DataSheet1.zip › Supplementary Material/Forest and Funnel Plots/Crocus sativus/Waist circumference.pdf]

## Crocus sativus - TG

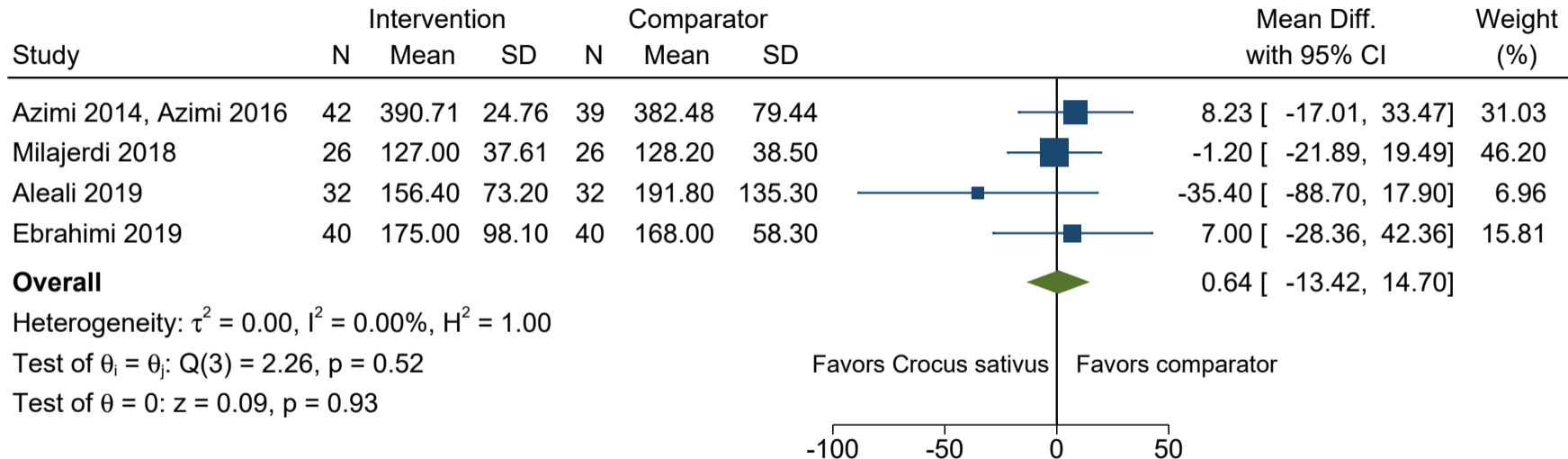

Random-effects REML model

Supplement: Supplementary file 1 [file DataSheet1.zip › Supplementary Material/Forest and Funnel Plots/Crocus sativus/TG.pdf]

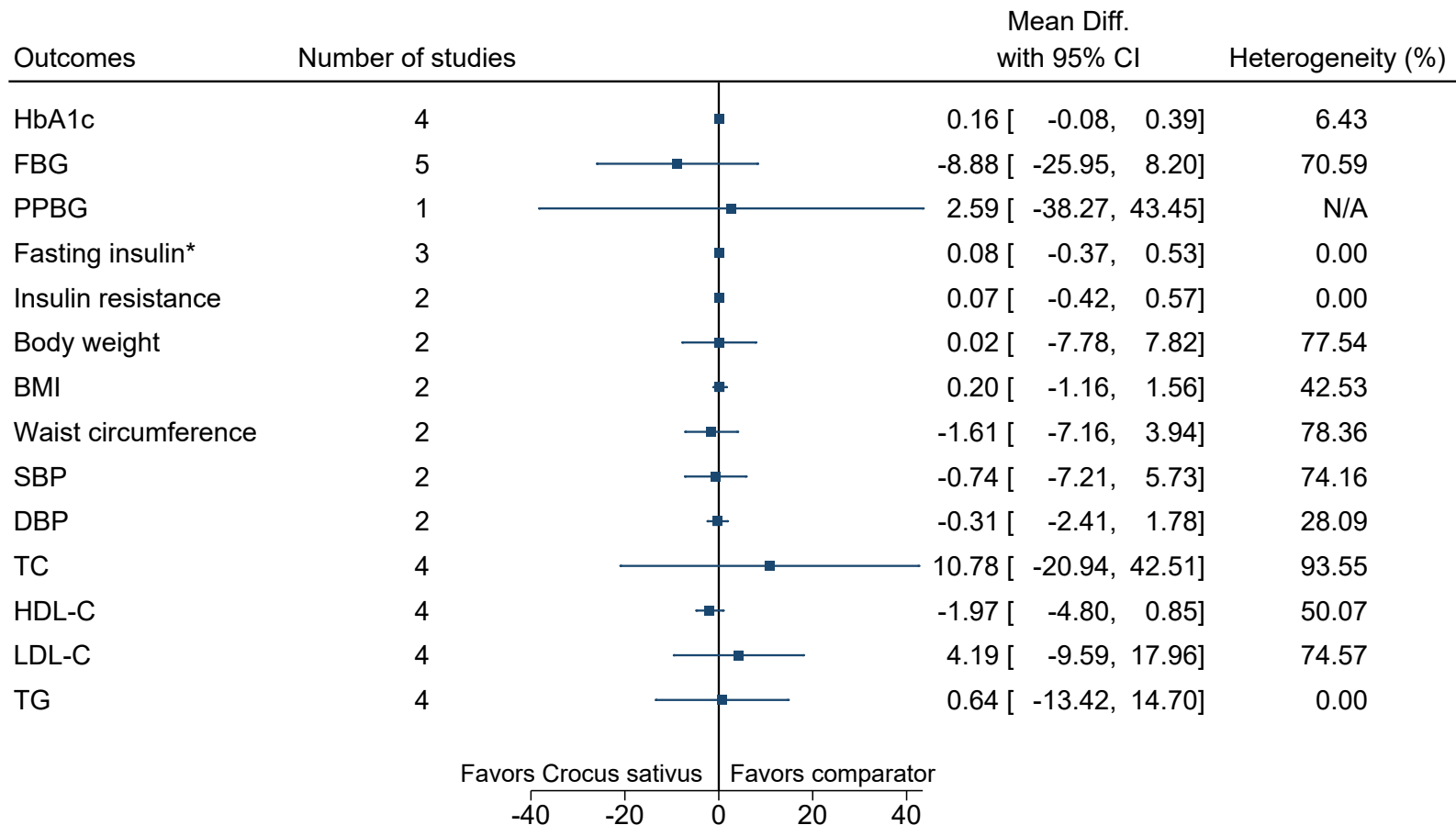

\*Favors intervention/comparator as insulin sensitizer

Supplement: Supplementary file 1 [file DataSheet1.zip › Supplementary Material/Forest and Funnel Plots/Crocus sativus/Crocus sativus.pdf]

## Crocus sativus - BMI

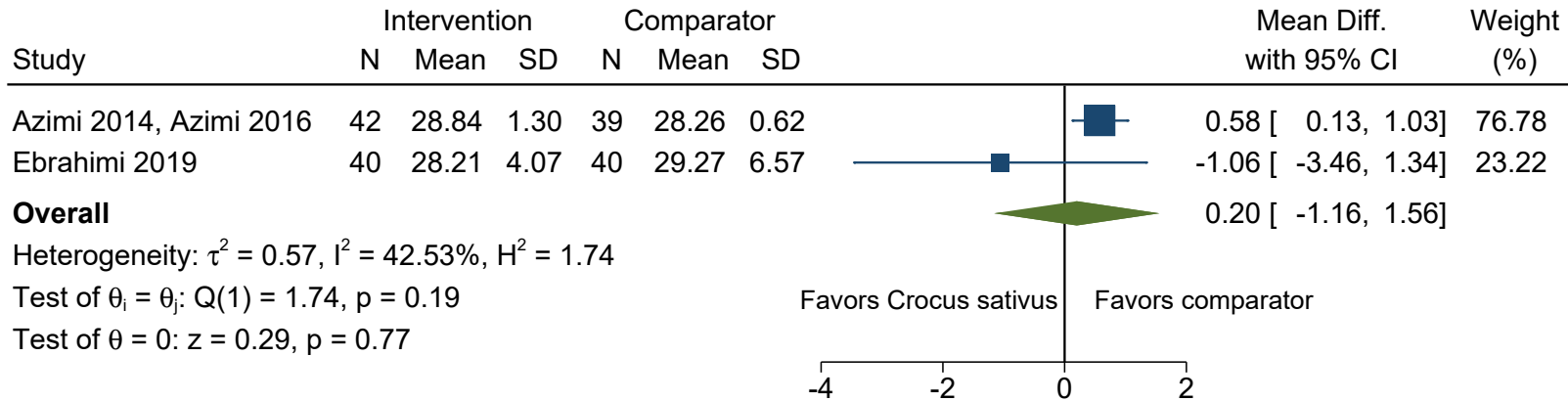

Random-effects REML model

Supplement: Supplementary file 1 [file DataSheet1.zip › Supplementary Material/Forest and Funnel Plots/Crocus sativus/BMI.pdf]

## Crocus sativus - TC

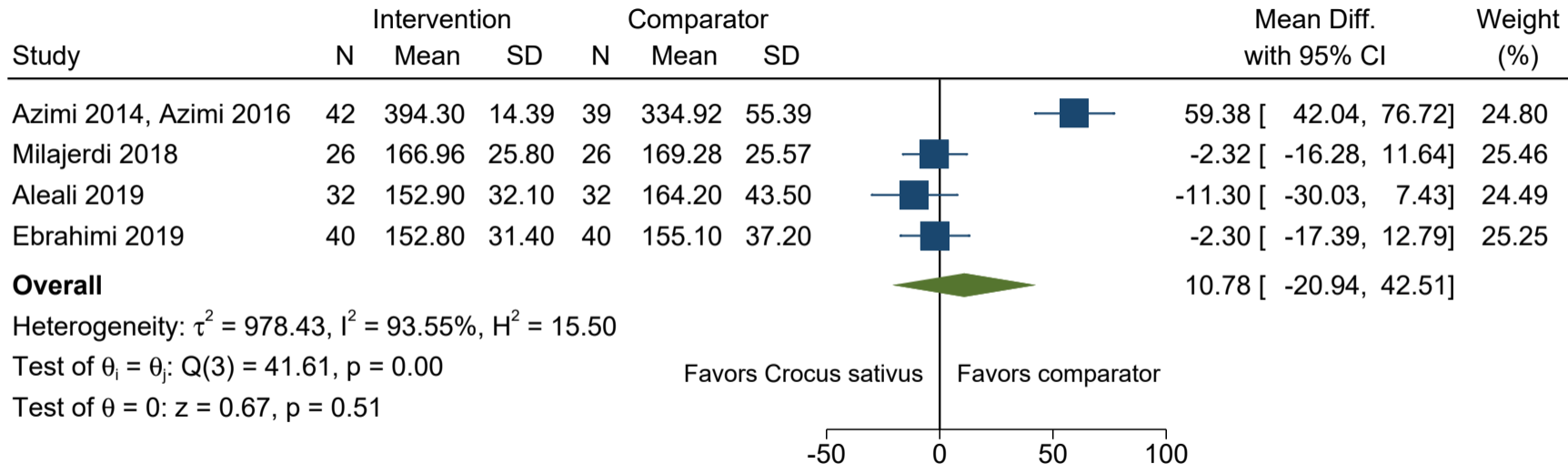

Random-effects REML model

Supplement: Supplementary file 1 [file DataSheet1.zip › Supplementary Material/Forest and Funnel Plots/Crocus sativus/TC.pdf]

# Crocus sativus - FBG

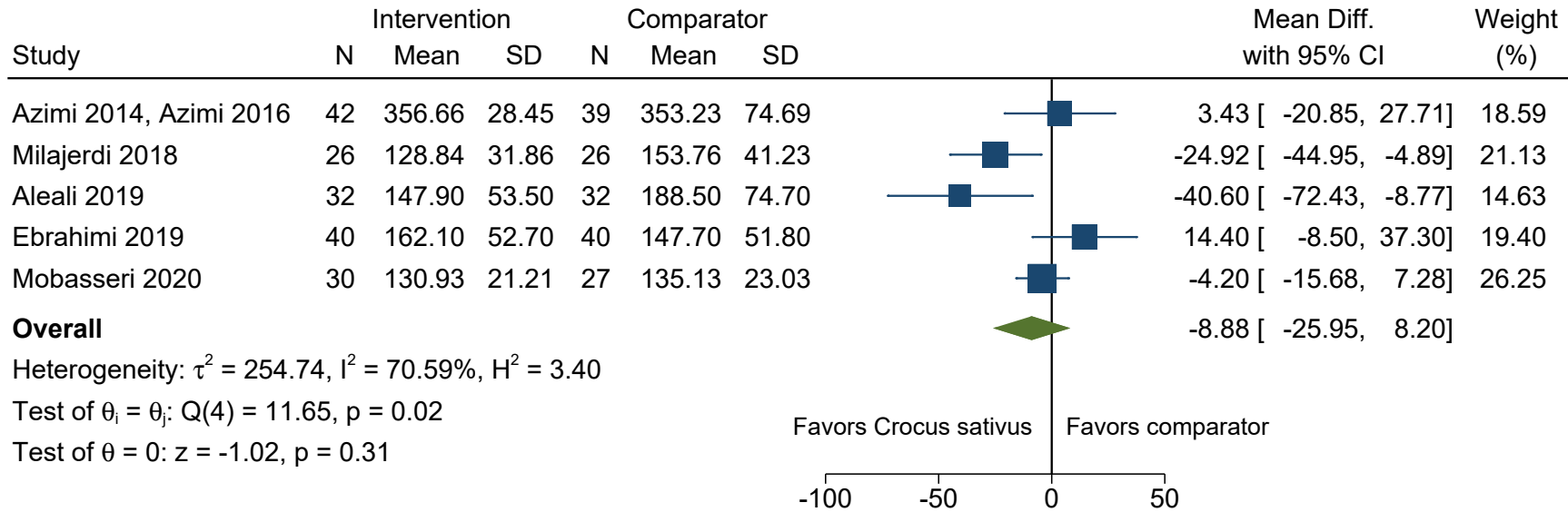

Supplement: Supplementary file 1 [file DataSheet1.zip › Supplementary Material/Forest and Funnel Plots/Crocus sativus/FBG.pdf]

# Crocus sativus - HDL-C

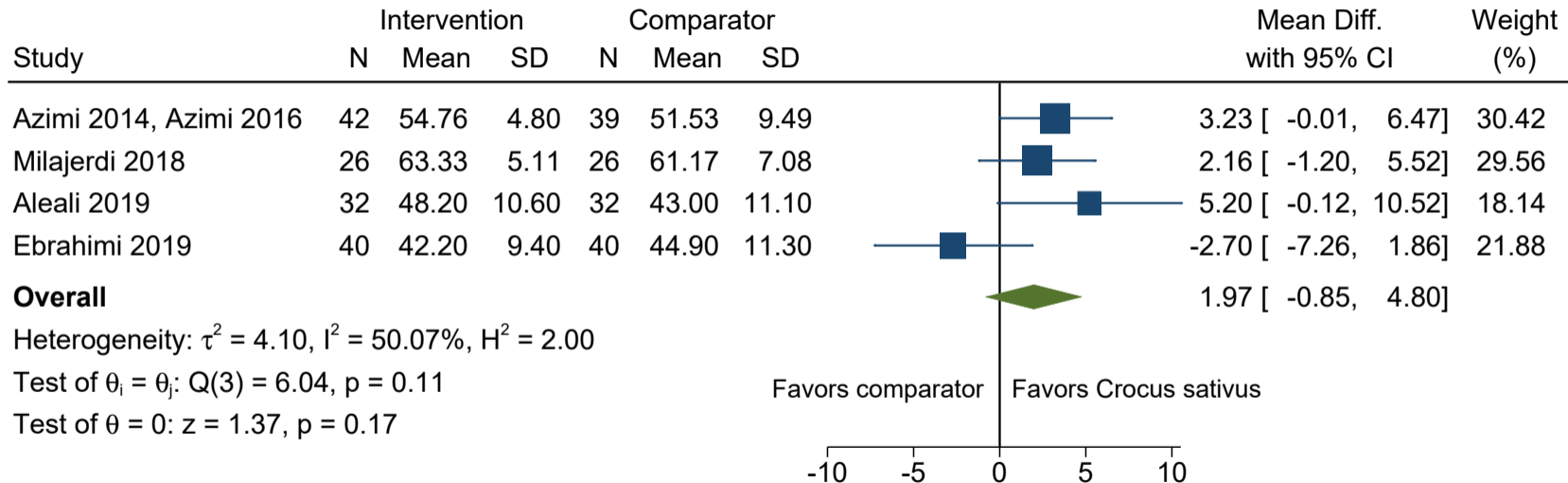

Random-effects REML model

Supplement: Supplementary file 1 [file DataSheet1.zip › Supplementary Material/Forest and Funnel Plots/Crocus sativus/HDL-C.pdf]

# Crocus sativus - DBP

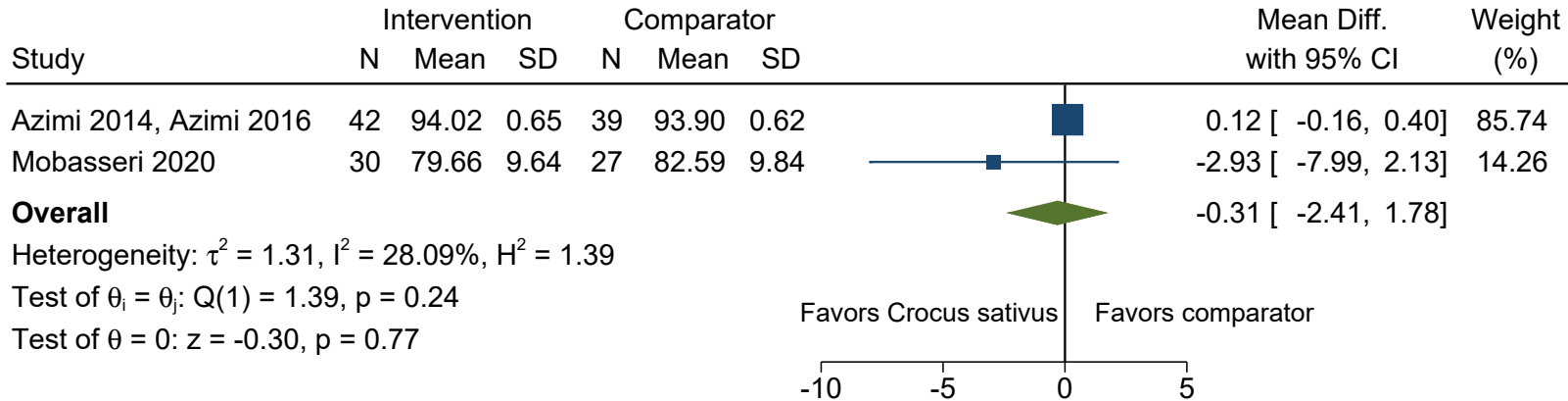

Random-effects REML model

Supplement: Supplementary file 1 [file DataSheet1.zip › Supplementary Material/Forest and Funnel Plots/Crocus sativus/DBP.pdf]

# Crocus sativus - Body weight

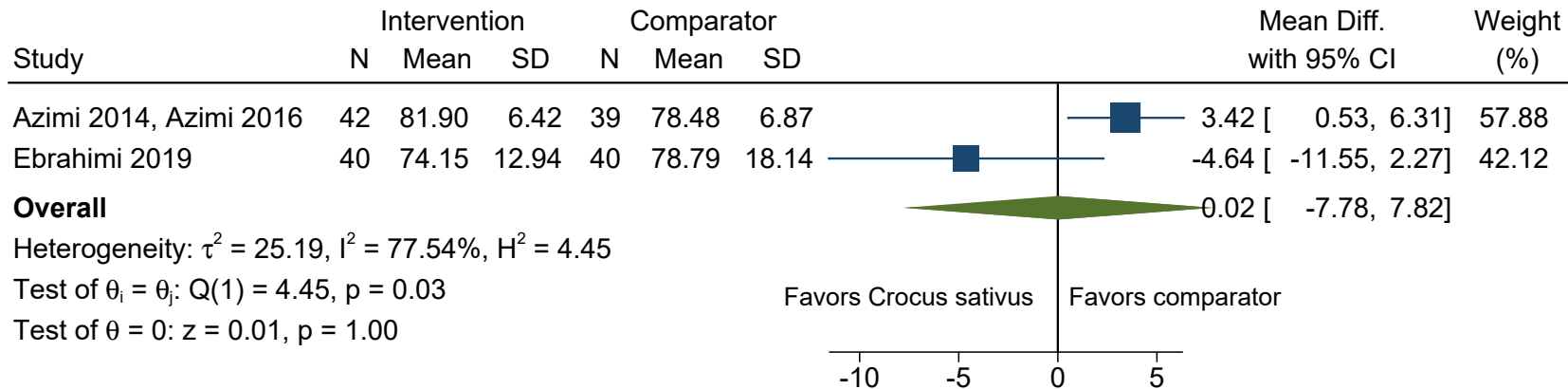

Random-effects REML model

Supplement: Supplementary file 1 [file DataSheet1.zip › Supplementary Material/Forest and Funnel Plots/Crocus sativus/Body weight.pdf]

# Crocus sativus - Insulin resistance

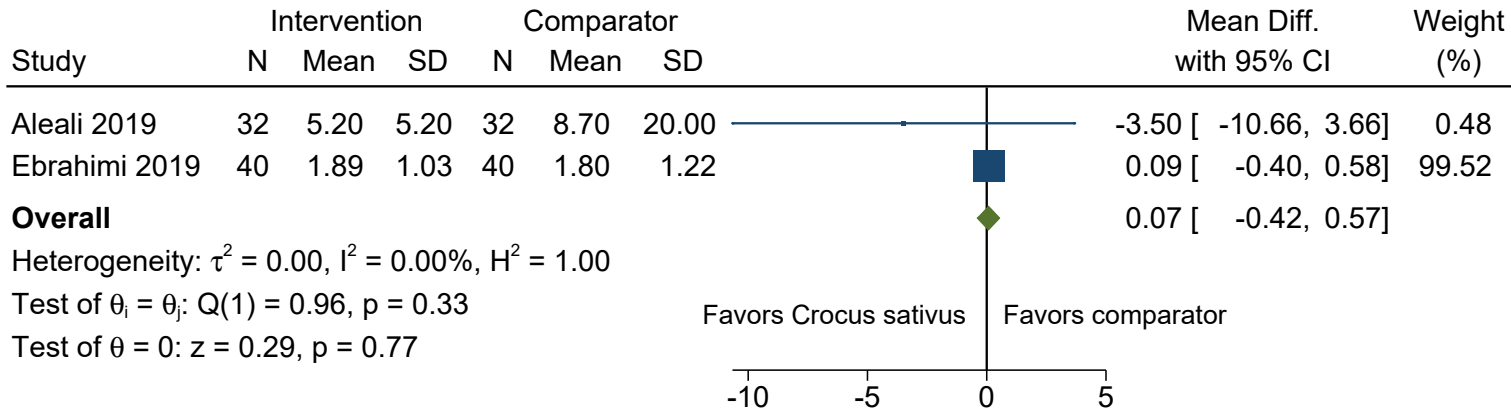

Random-effects REML model

Supplement: Supplementary file 1 [file DataSheet1.zip › Supplementary Material/Forest and Funnel Plots/Crocus sativus/Insulin resistance.pdf]

# Crocus sativus - Fasting insulin

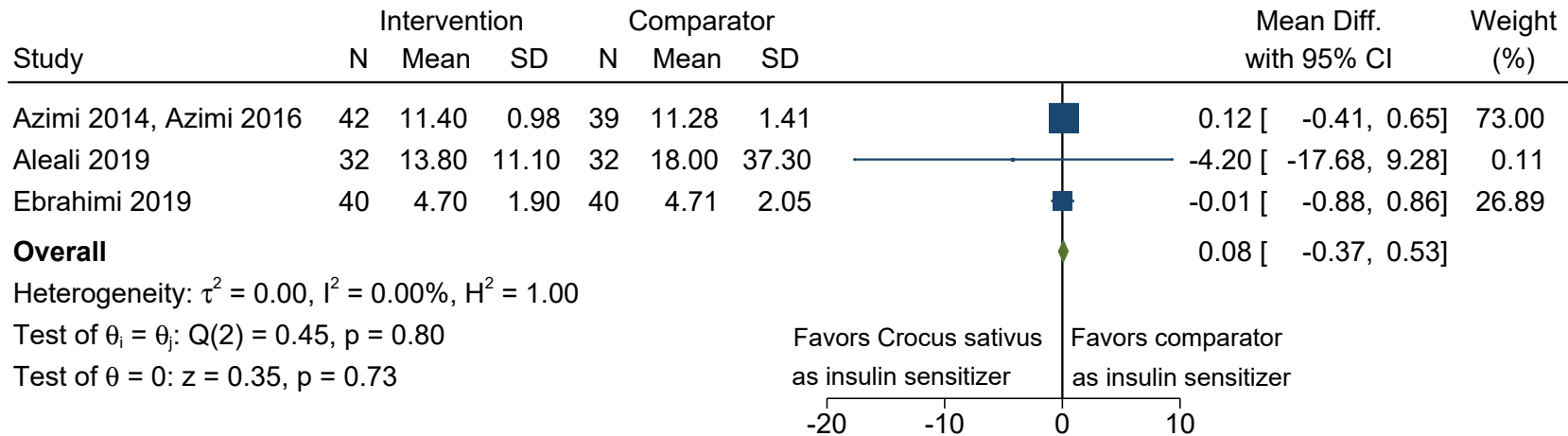

Supplement: Supplementary file 1 [file DataSheet1.zip › Supplementary Material/Forest and Funnel Plots/Crocus sativus/Fasting insulin.pdf]

# Crocus sativus - HbA1c

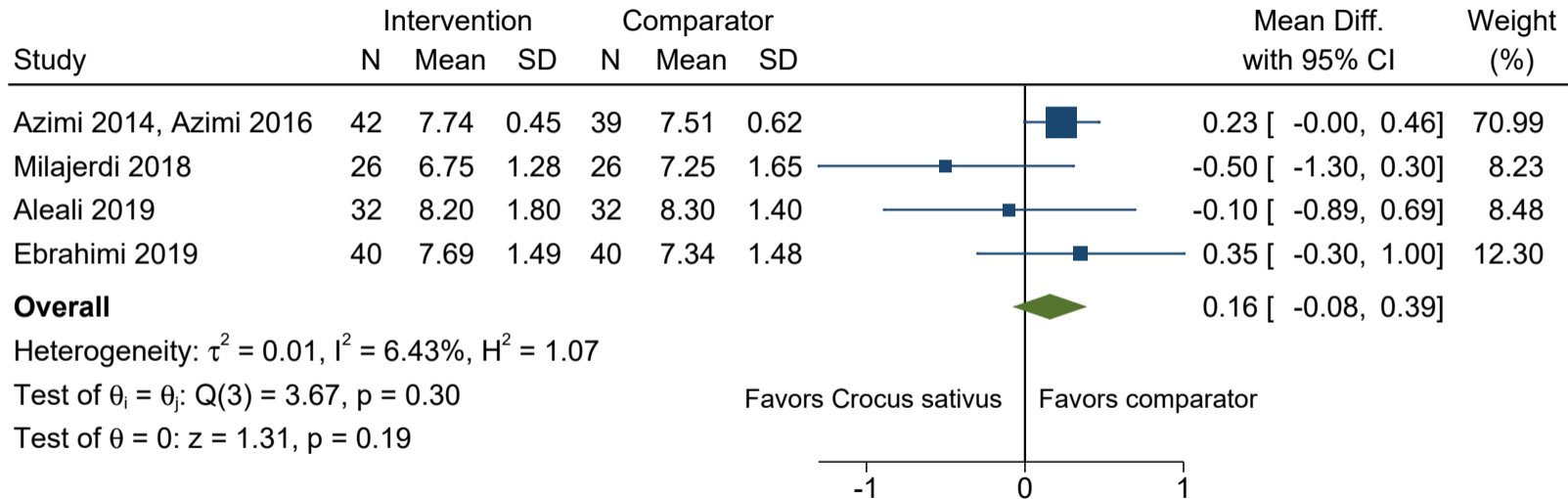

Random-effects REML model

Supplement: Supplementary file 1 [file DataSheet1.zip › Supplementary Material/Forest and Funnel Plots/Crocus sativus/HbA1c.pdf]

# Crocus sativus - SBP

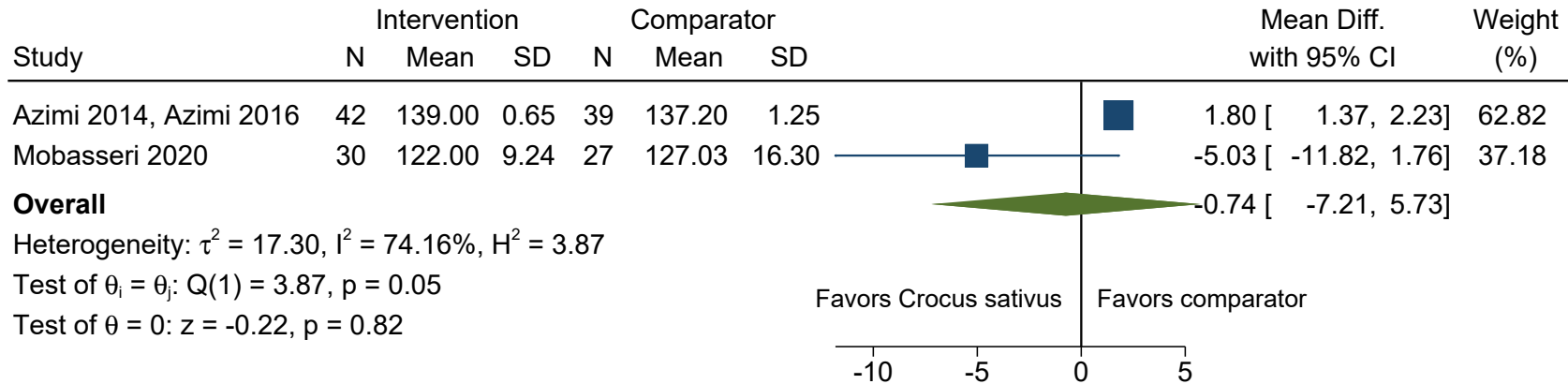

Random-effects REML model

Supplement: Supplementary file 1 [file DataSheet1.zip › Supplementary Material/Forest and Funnel Plots/Crocus sativus/SBP.pdf]

# Crocus sativus - LDL-C

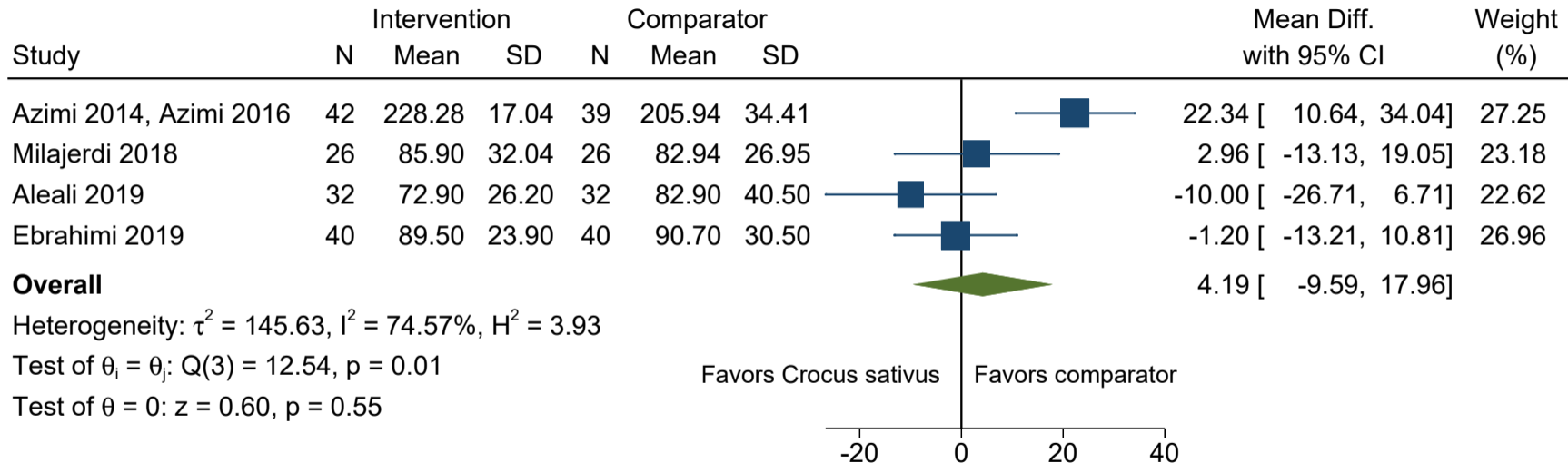

Random-effects REML model

Supplement: Supplementary file 1 [file DataSheet1.zip › Supplementary Material/Forest and Funnel Plots/Crocus sativus/LDL-C.pdf]

# Pterocarpus marsupium - FBG

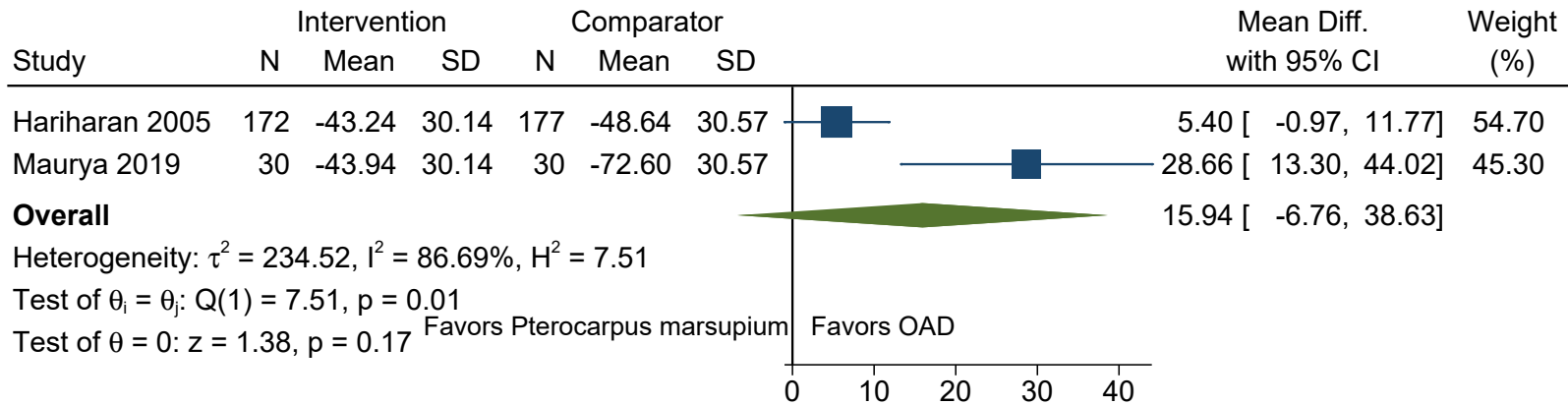

Random-effects REML model

Supplement: Supplementary file 1 [file DataSheet1.zip › Supplementary Material/Forest and Funnel Plots/Pterocarpus marsupium (versus OAD)/FBG.pdf]

# Pterocarpus marsupium - PPBG

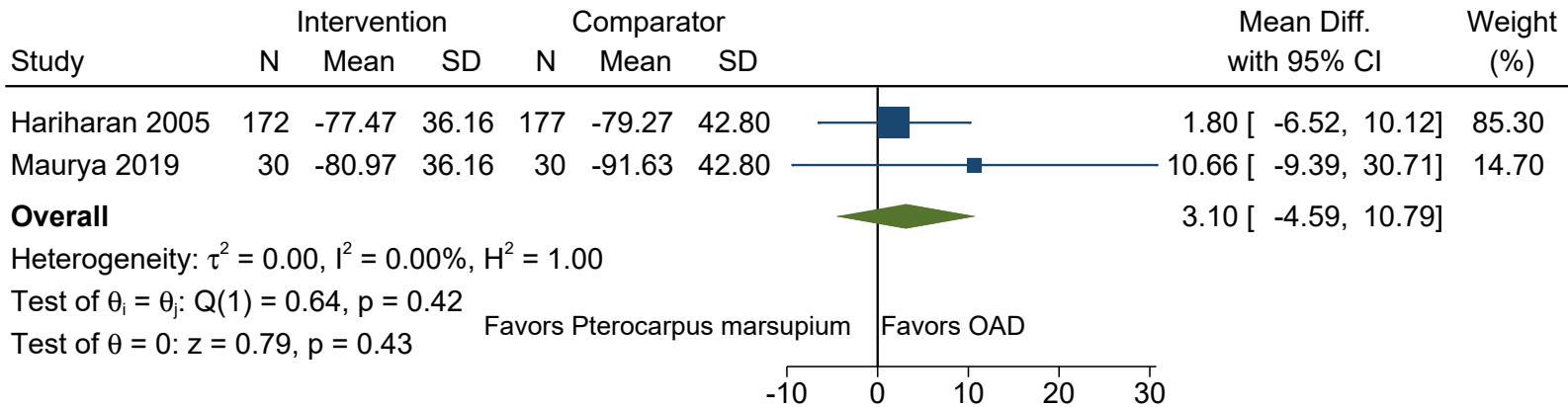

Random-effects REML model

Supplement: Supplementary file 1 [file DataSheet1.zip › Supplementary Material/Forest and Funnel Plots/Pterocarpus marsupium (versus OAD)/PPBG.pdf]

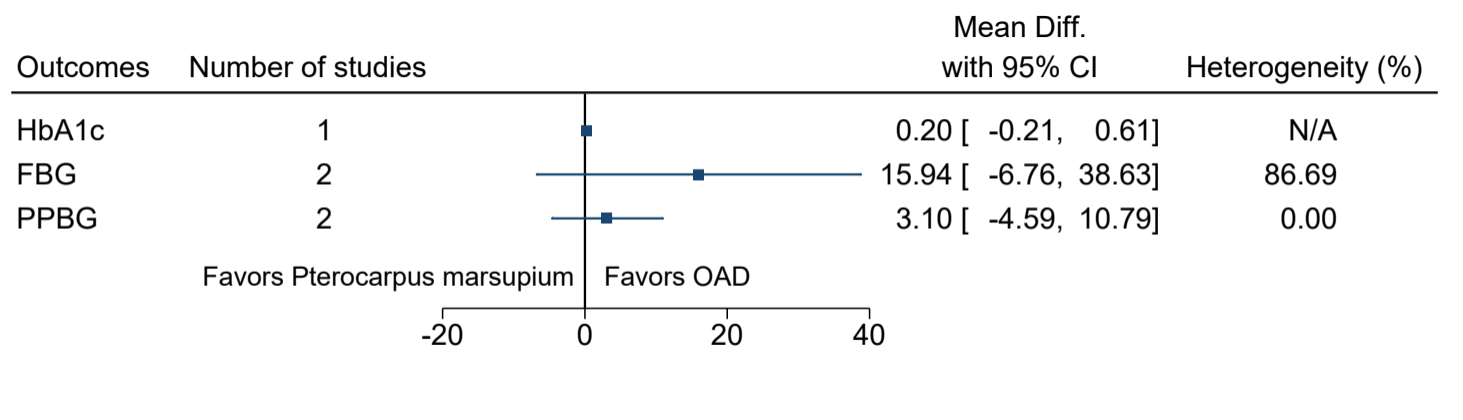

Supplement: Supplementary file 1 [file DataSheet1.zip › Supplementary Material/Forest and Funnel Plots/Pterocarpus marsupium (versus OAD)/Pterocarpus marsupium.pdf]

Juglans regia - TG

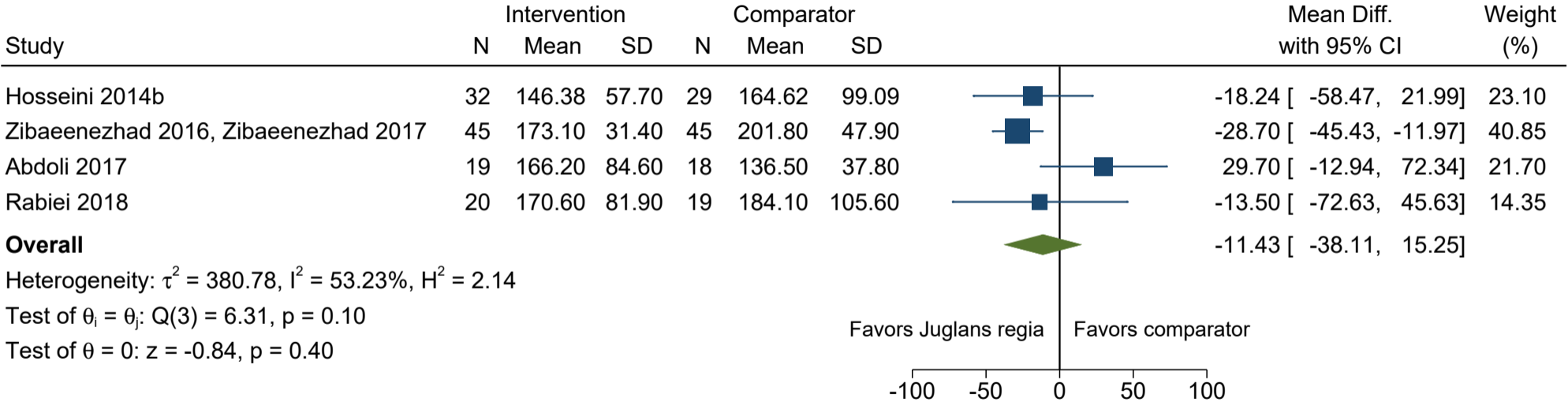

Supplement: Supplementary file 1 [file DataSheet1.zip › Supplementary Material/Forest and Funnel Plots/Juglans regia/TG.pdf]

# Juglans regia - BMI

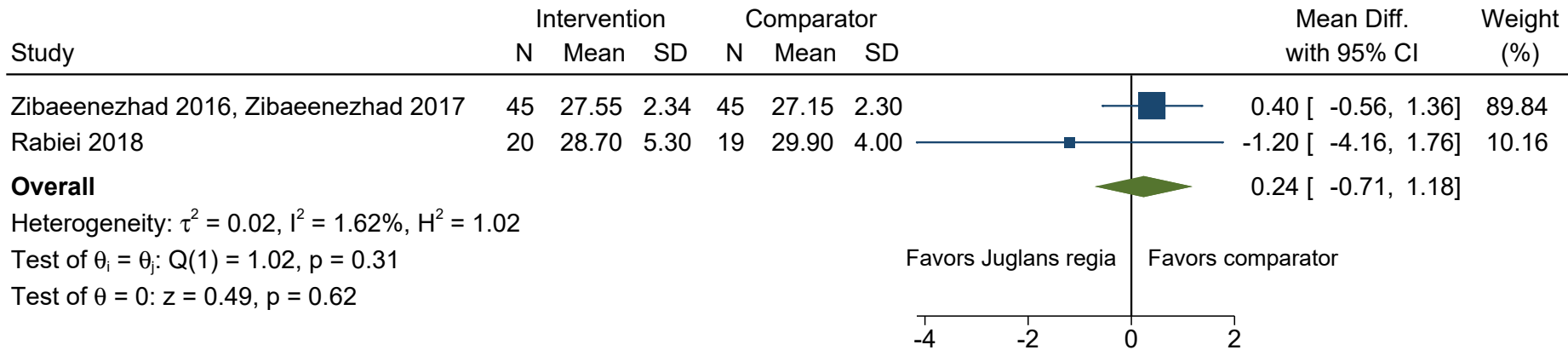

Random-effects REML model

Supplement: Supplementary file 1 [file DataSheet1.zip › Supplementary Material/Forest and Funnel Plots/Juglans regia/BMI.pdf]

# Juglans regia - TC

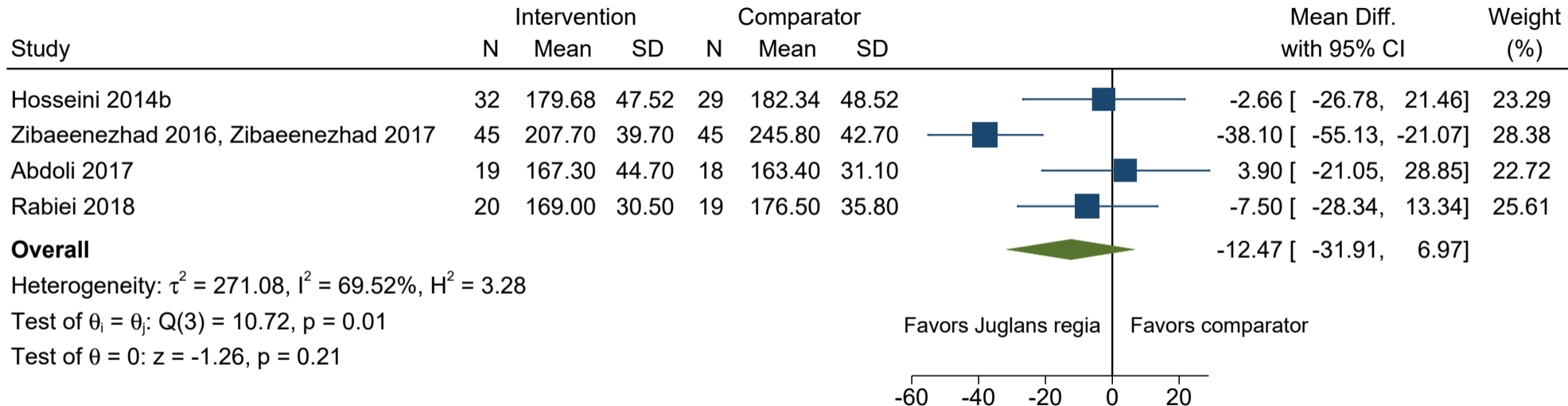

Supplement: Supplementary file 1 [file DataSheet1.zip › Supplementary Material/Forest and Funnel Plots/Juglans regia/TC.pdf]

# Juglans regia - FBG

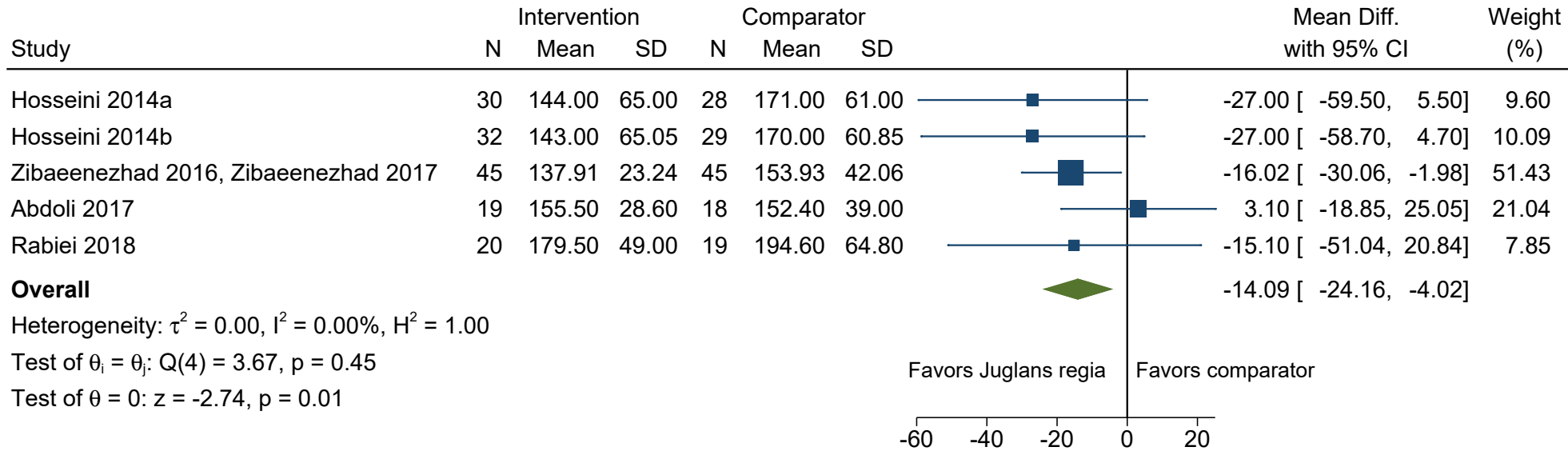

Supplement: Supplementary file 1 [file DataSheet1.zip › Supplementary Material/Forest and Funnel Plots/Juglans regia/FBG.pdf]

# Juglans regia - HDL-C

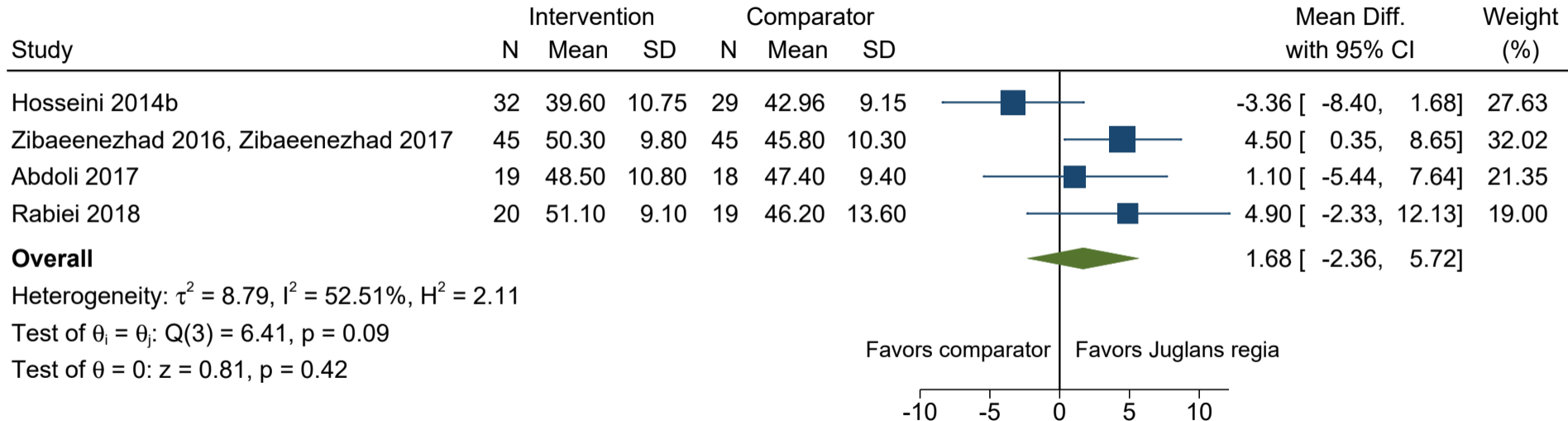

Random-effects REML model

Supplement: Supplementary file 1 [file DataSheet1.zip › Supplementary Material/Forest and Funnel Plots/Juglans regia/HDL-C.pdf]

# Juglans regia - DBP

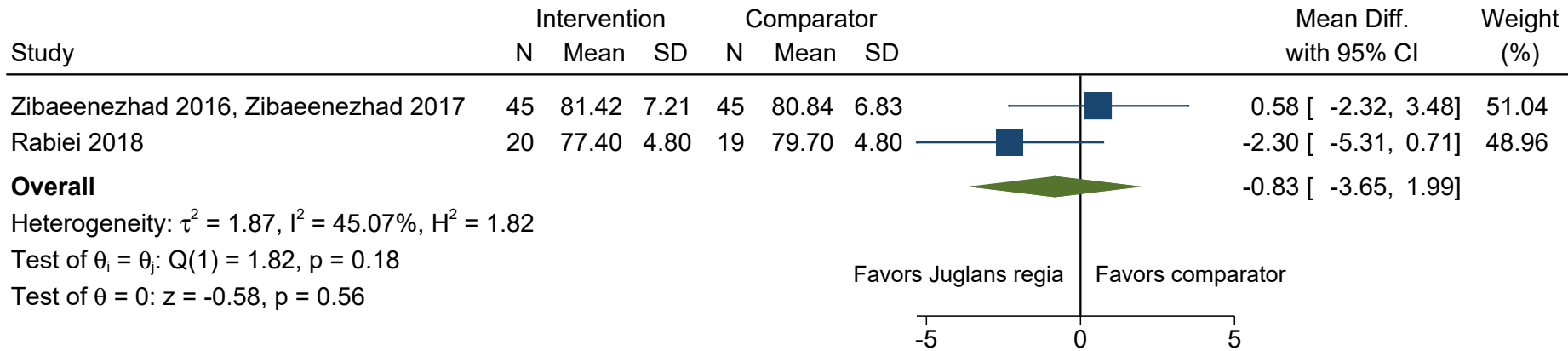

Random-effects REML model

Supplement: Supplementary file 1 [file DataSheet1.zip › Supplementary Material/Forest and Funnel Plots/Juglans regia/DBP.pdf]

# Juglans regia - Body weight

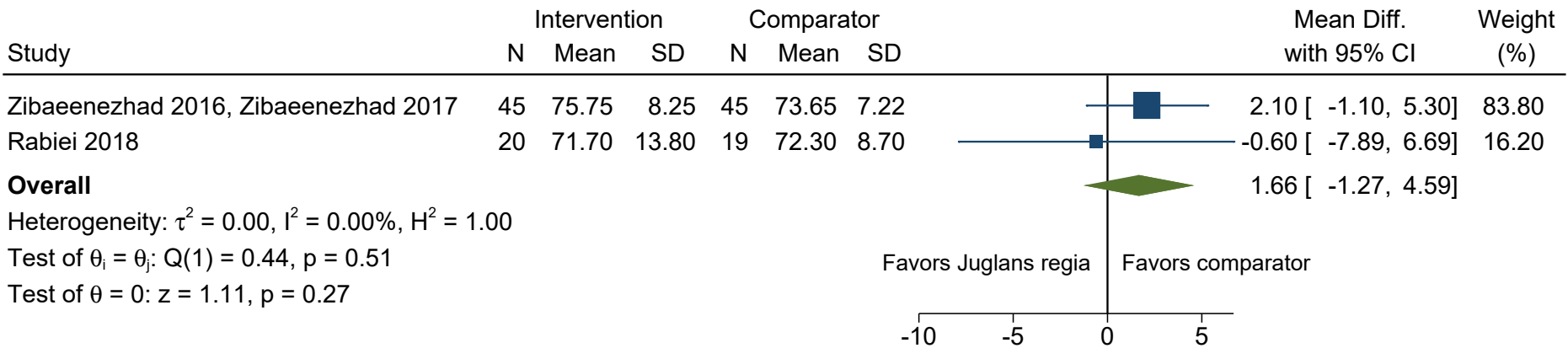

Random-effects REML model

Supplement: Supplementary file 1 [file DataSheet1.zip › Supplementary Material/Forest and Funnel Plots/Juglans regia/Body weight.pdf]

# Juglans regia - Fasting insulin

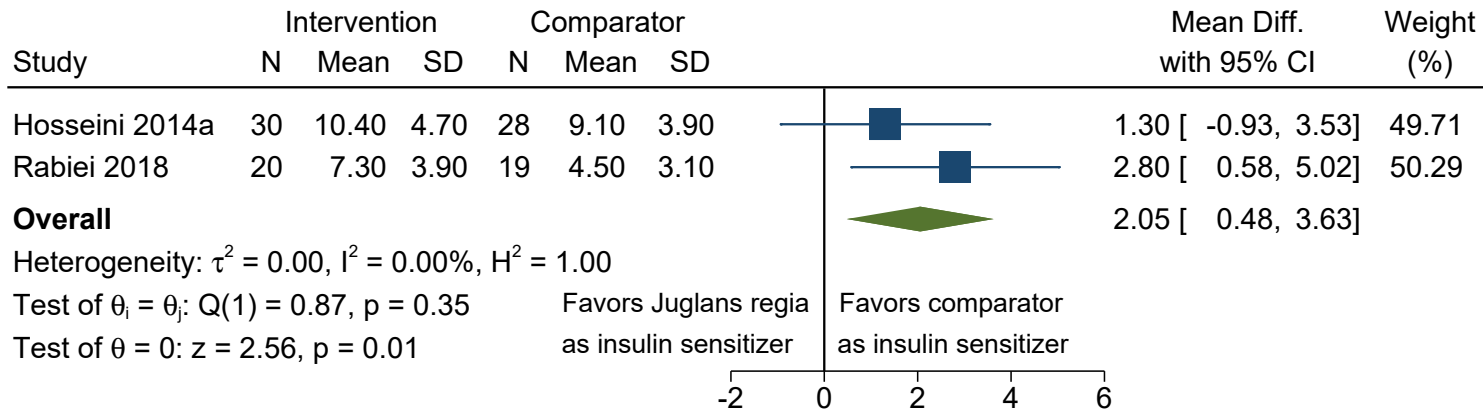

Supplement: Supplementary file 1 [file DataSheet1.zip › Supplementary Material/Forest and Funnel Plots/Juglans regia/Fasting insulin.pdf]

# Juglans regia - HbA1c

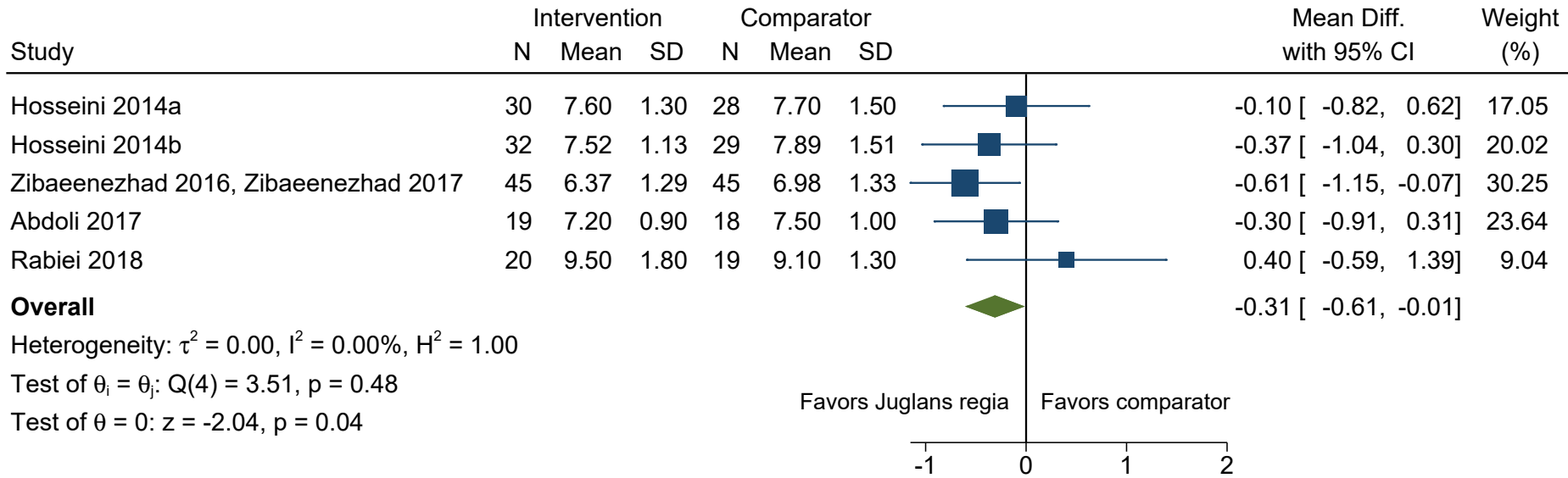

Supplement: Supplementary file 1 [file DataSheet1.zip › Supplementary Material/Forest and Funnel Plots/Juglans regia/HbA1c.pdf]

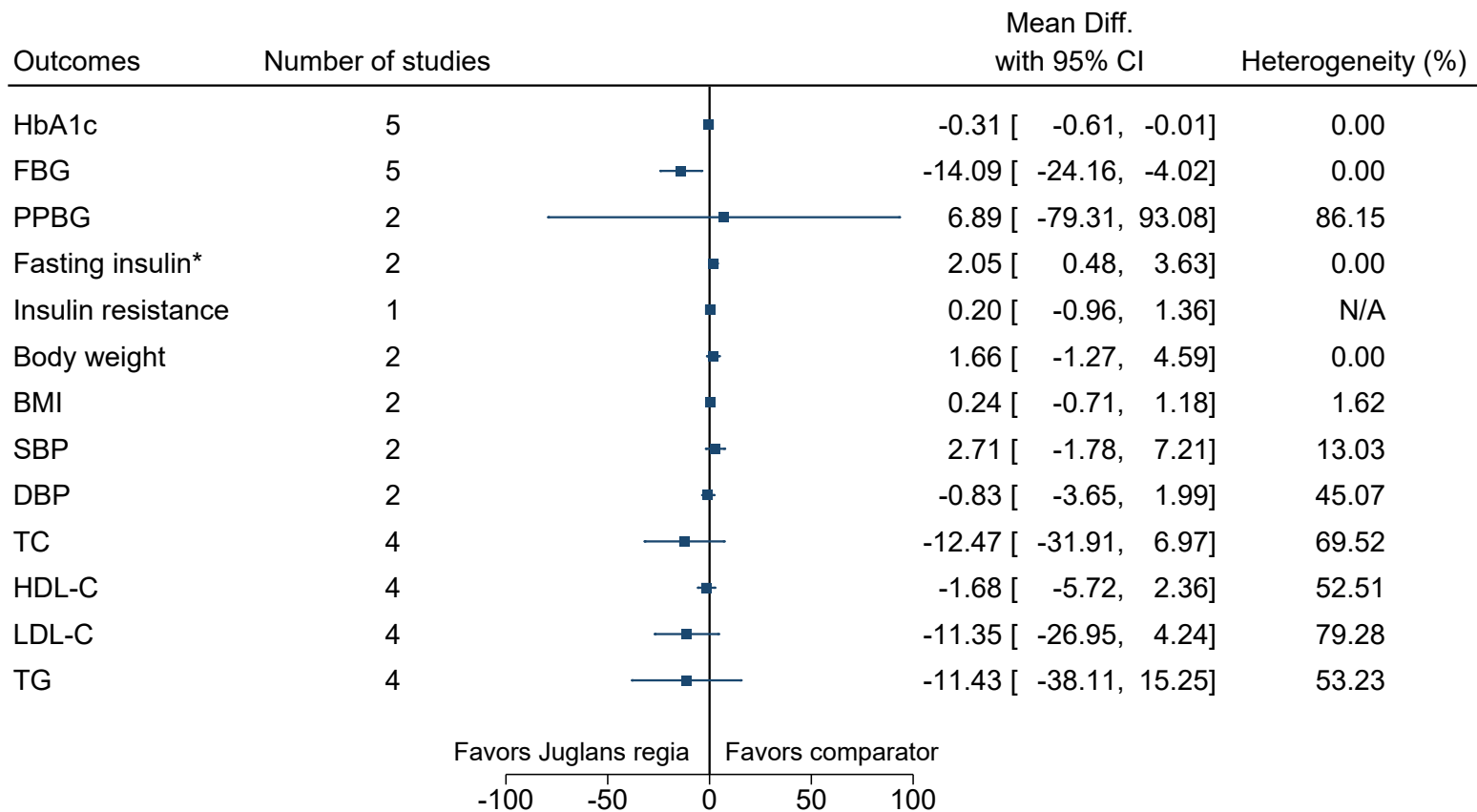

\*Favors intervention/comparator as insulin sensitizer

Supplement: Supplementary file 1 [file DataSheet1.zip › Supplementary Material/Forest and Funnel Plots/Juglans regia/Juglans regia.pdf]

# Juglans regia - PPBG

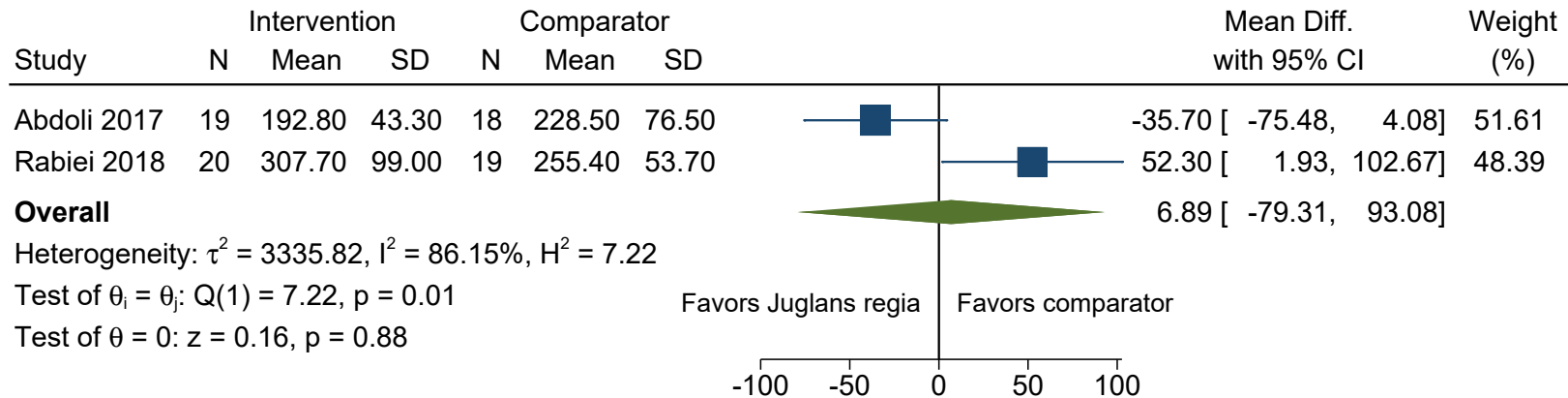

Random-effects REML model

Supplement: Supplementary file 1 [file DataSheet1.zip › Supplementary Material/Forest and Funnel Plots/Juglans regia/PPBG.pdf]

# Juglans regia - SBP

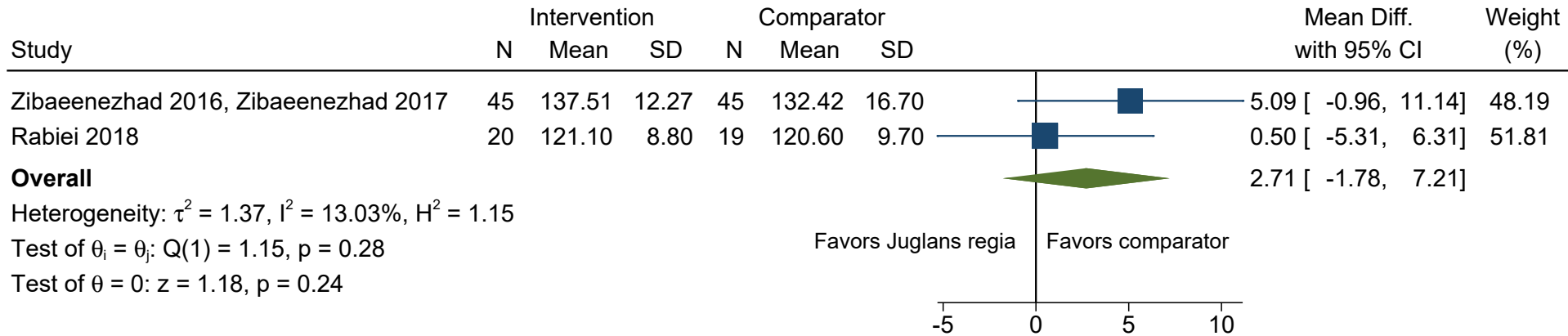

Random-effects REML model

Supplement: Supplementary file 1 [file DataSheet1.zip › Supplementary Material/Forest and Funnel Plots/Juglans regia/SBP.pdf]

# Juglans regia - LDL-C

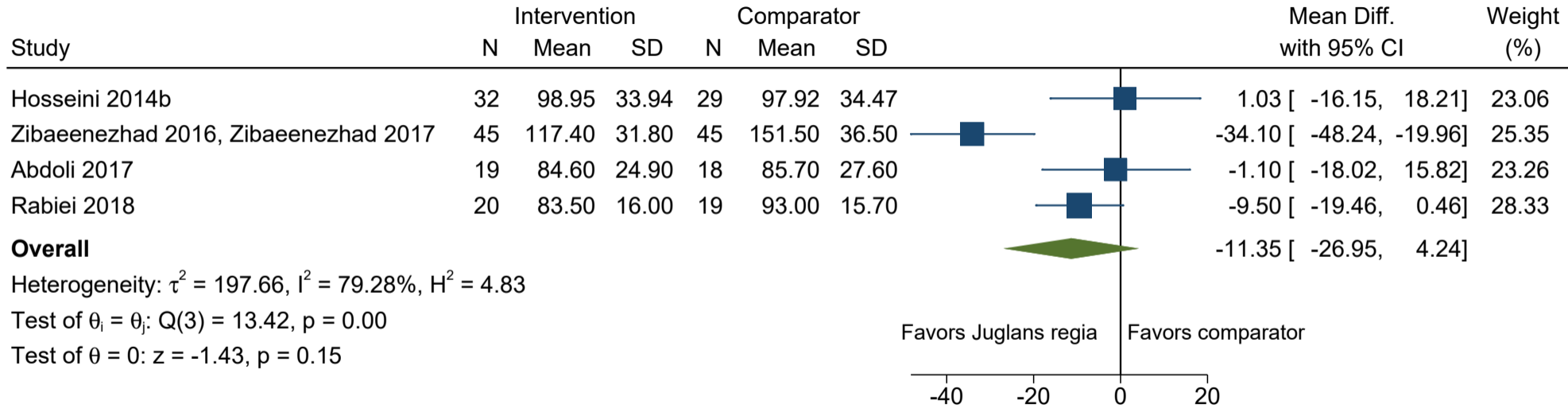

Supplement: Supplementary file 1 [file DataSheet1.zip › Supplementary Material/Forest and Funnel Plots/Juglans regia/LDL-C.pdf]

# Cinnamomum aromaticum - Waist circumference

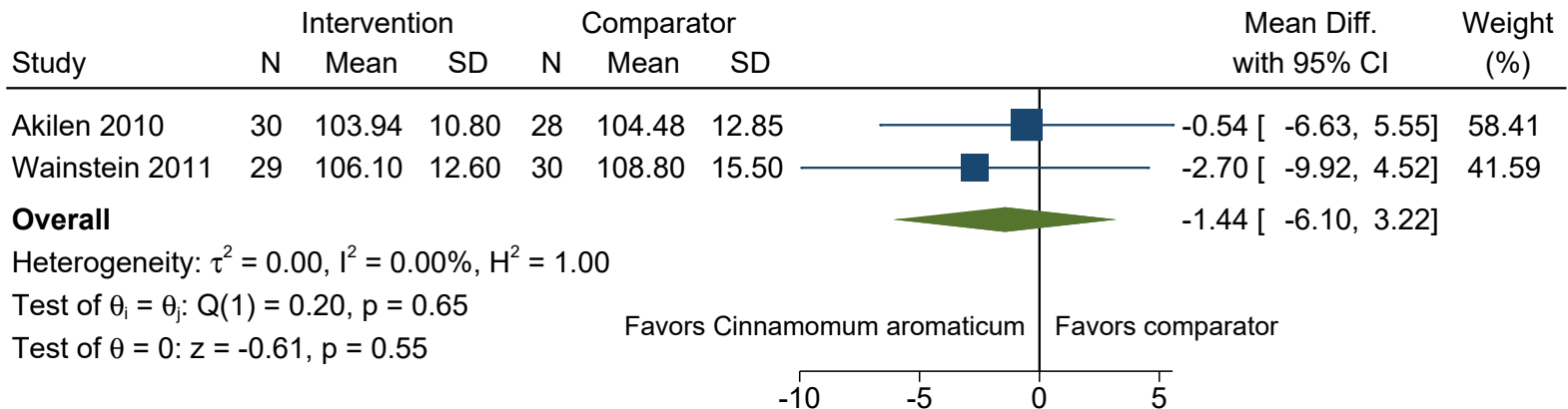

Random-effects REML model

Supplement: Supplementary file 1 [file DataSheet1.zip › Supplementary Material/Forest and Funnel Plots/Cinnamomum aromaticum/Waist circumference.pdf]

# Cinnamomum aromaticum - TG

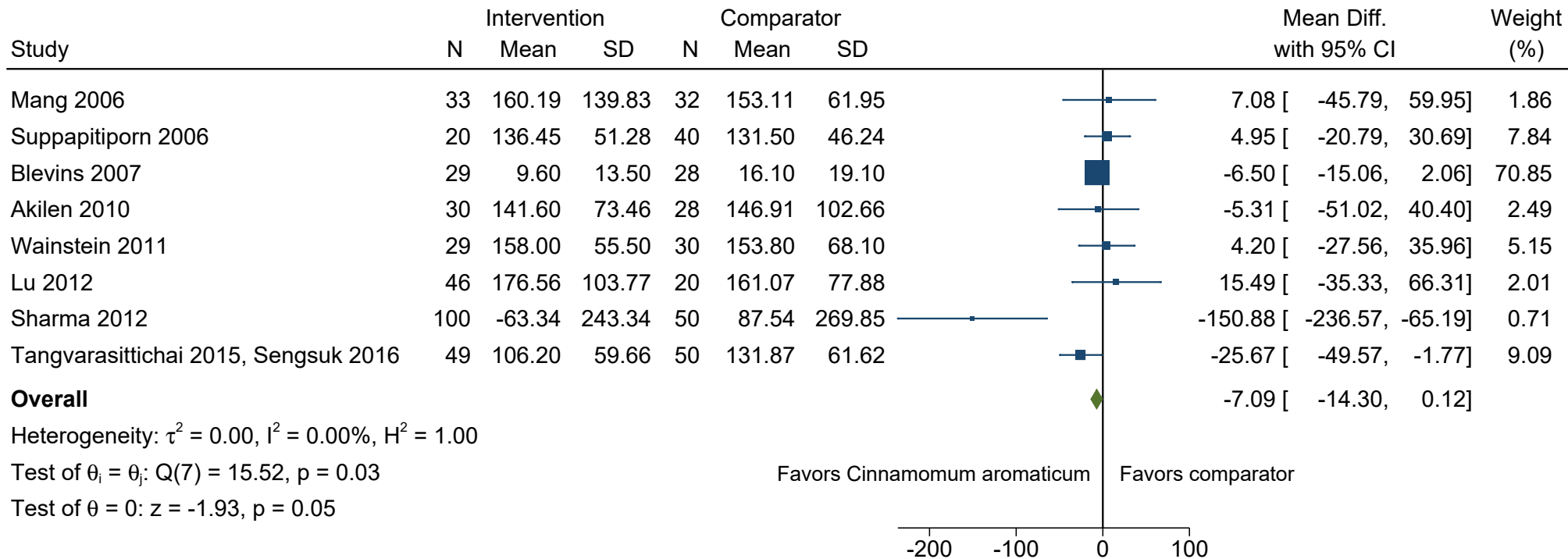

Supplement: Supplementary file 1 [file DataSheet1.zip › Supplementary Material/Forest and Funnel Plots/Cinnamomum aromaticum/TG.pdf]

# Cinnamomum aromaticum - BMI

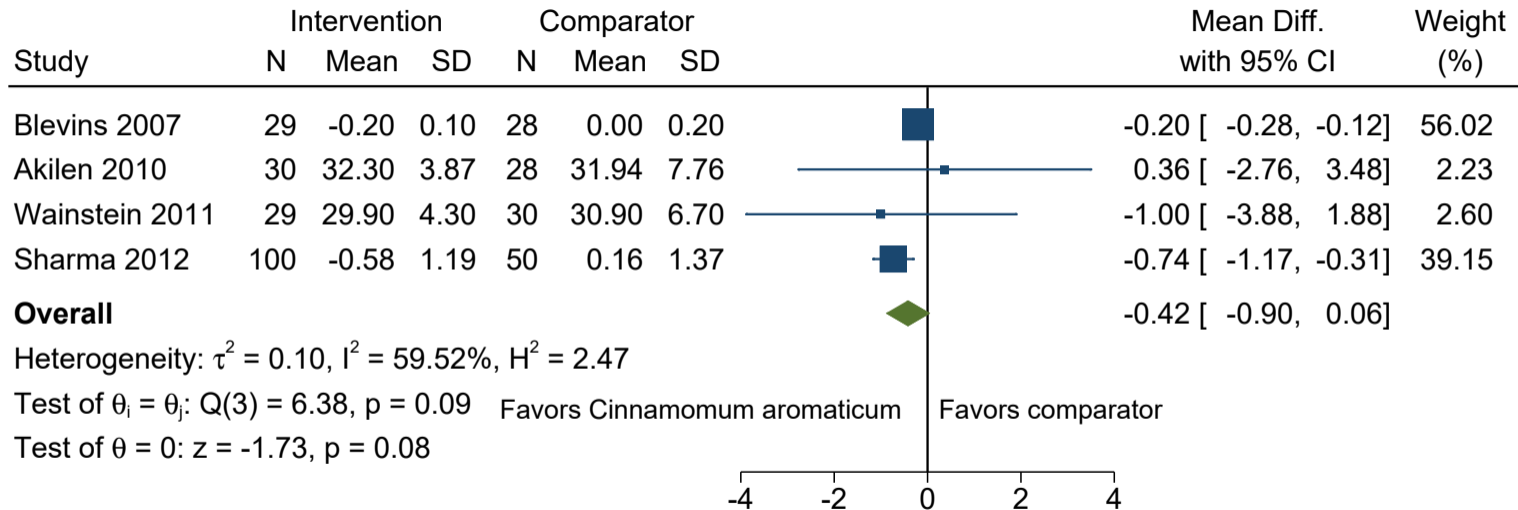

Random-effects REML model

Supplement: Supplementary file 1 [file DataSheet1.zip › Supplementary Material/Forest and Funnel Plots/Cinnamomum aromaticum/BMI.pdf]

# Cinnamomum aromaticum - TC

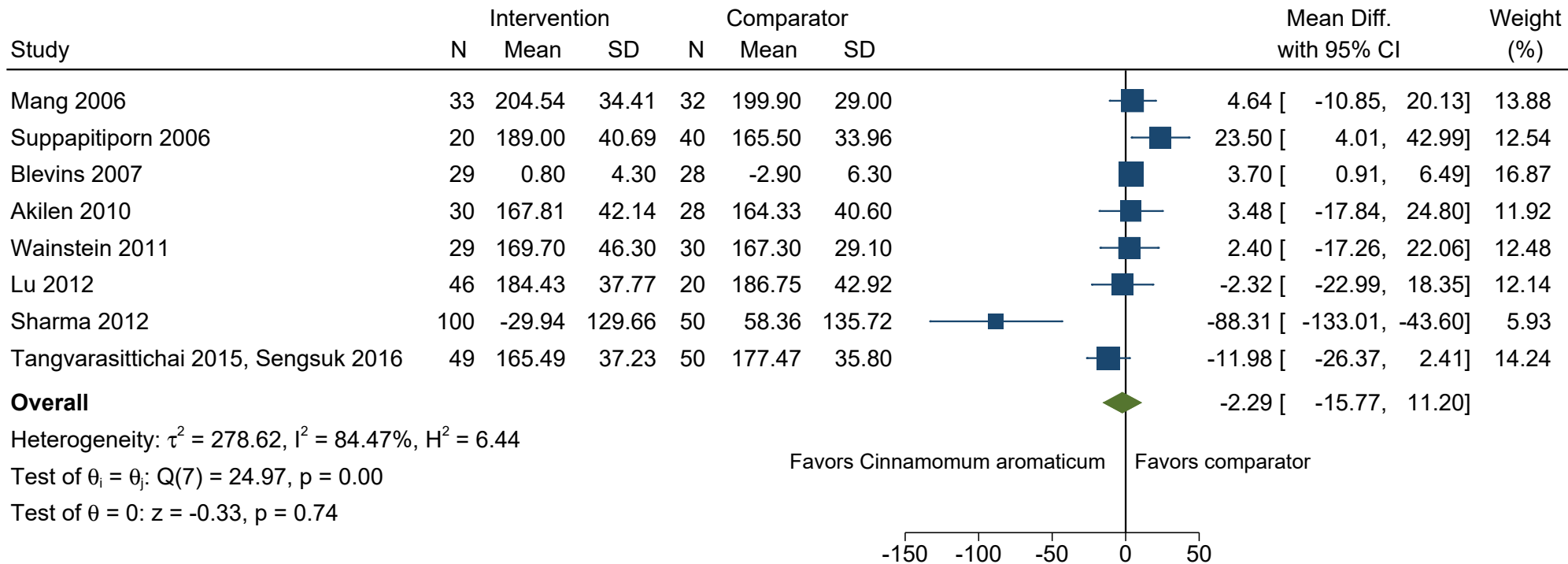

Supplement: Supplementary file 1 [file DataSheet1.zip › Supplementary Material/Forest and Funnel Plots/Cinnamomum aromaticum/TC.pdf]

# Cinnamomum aromaticum - FBG

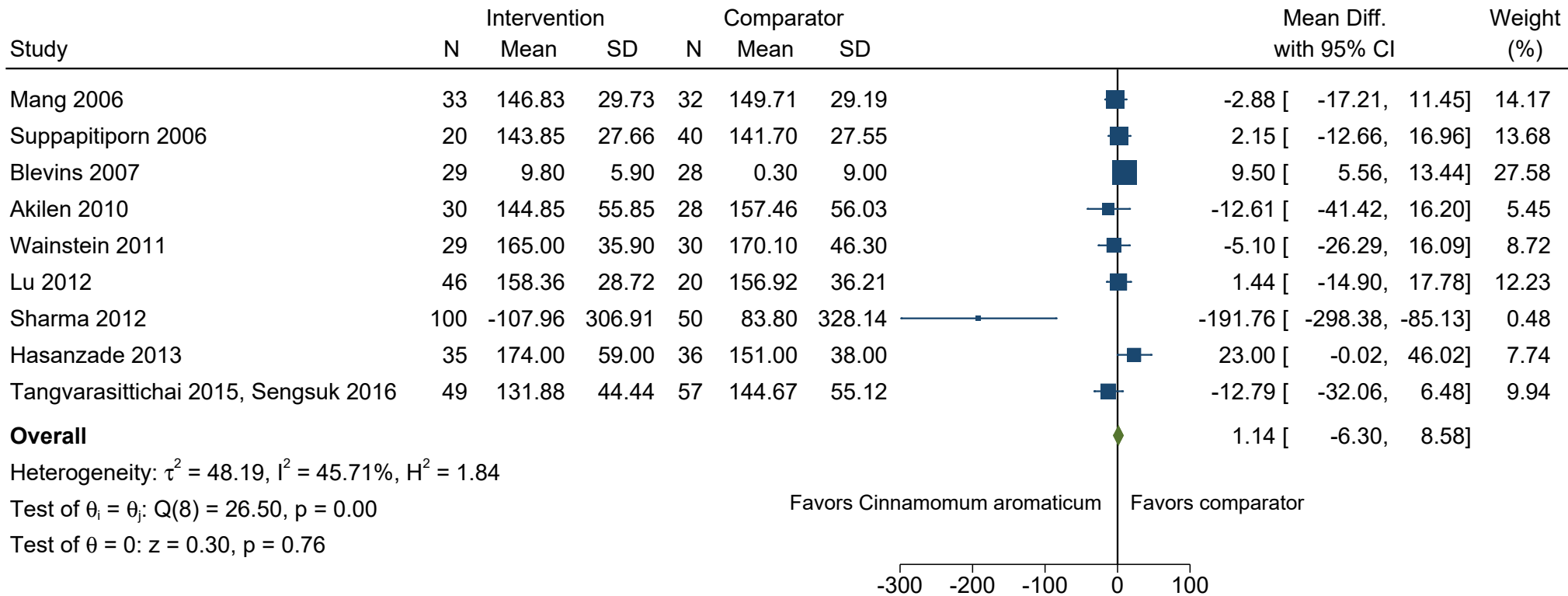

Supplement: Supplementary file 1 [file DataSheet1.zip › Supplementary Material/Forest and Funnel Plots/Cinnamomum aromaticum/FBG.pdf]

# Cinnamomum aromaticum - HDL-C

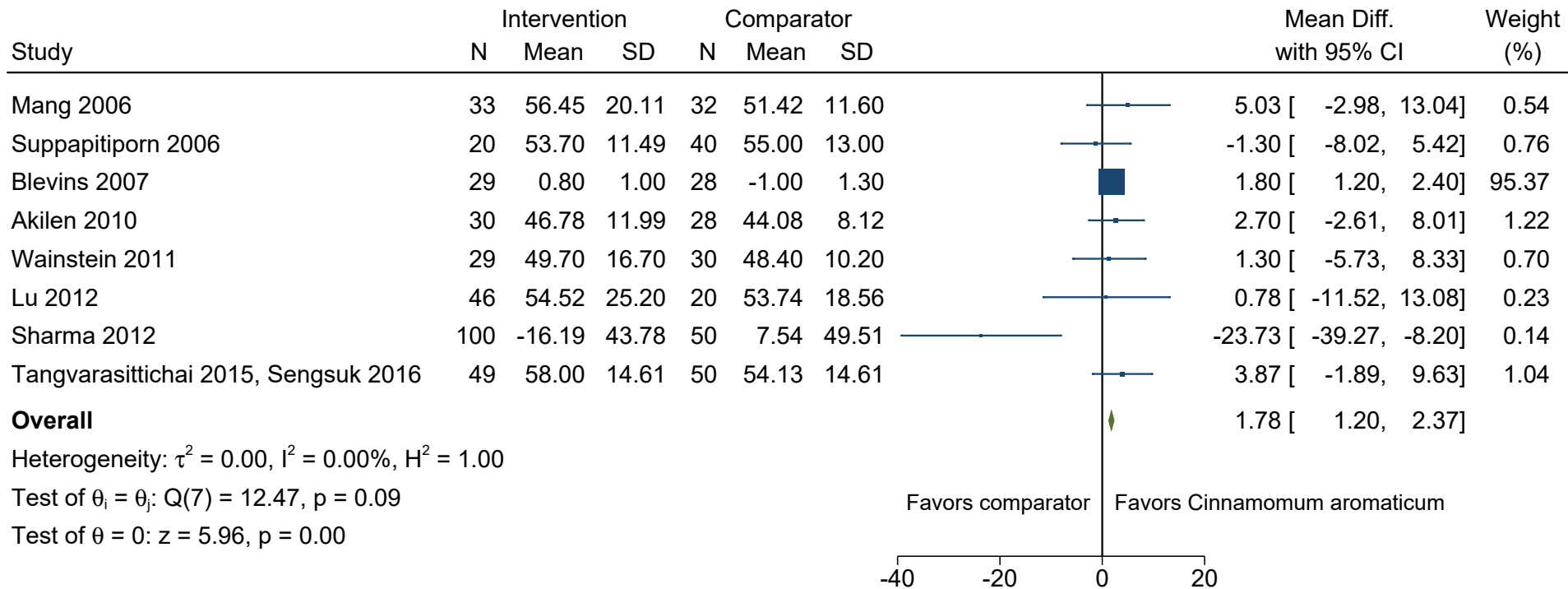

Supplement: Supplementary file 1 [file DataSheet1.zip › Supplementary Material/Forest and Funnel Plots/Cinnamomum aromaticum/HDL-C.pdf]

# Cinnamomum aromaticum - DBP

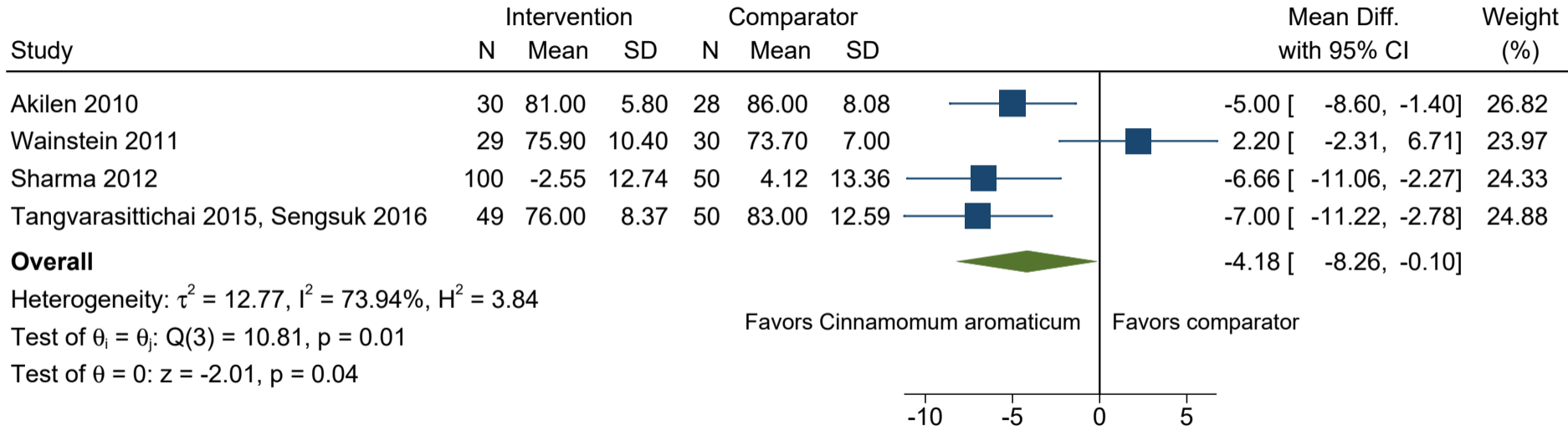

Random-effects REML model

Supplement: Supplementary file 1 [file DataSheet1.zip › Supplementary Material/Forest and Funnel Plots/Cinnamomum aromaticum/DBP.pdf]

# Cinnamomum aromaticum - Body weight

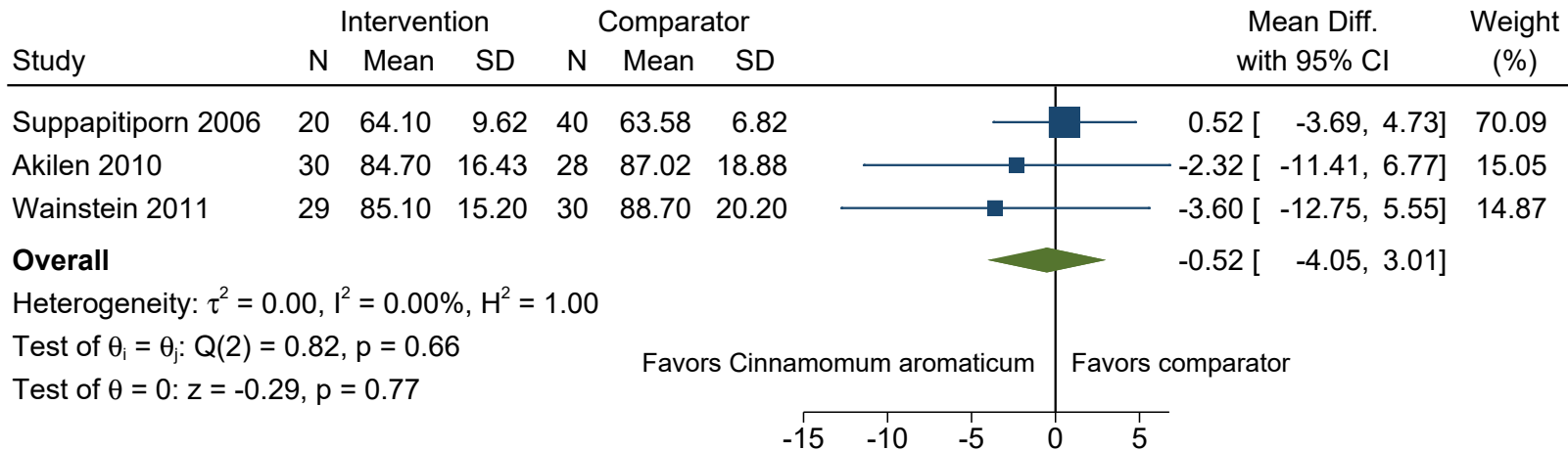

Supplement: Supplementary file 1 [file DataSheet1.zip › Supplementary Material/Forest and Funnel Plots/Cinnamomum aromaticum/Body weight.pdf]

# Cinnamomum aromaticum - Fasting insulin

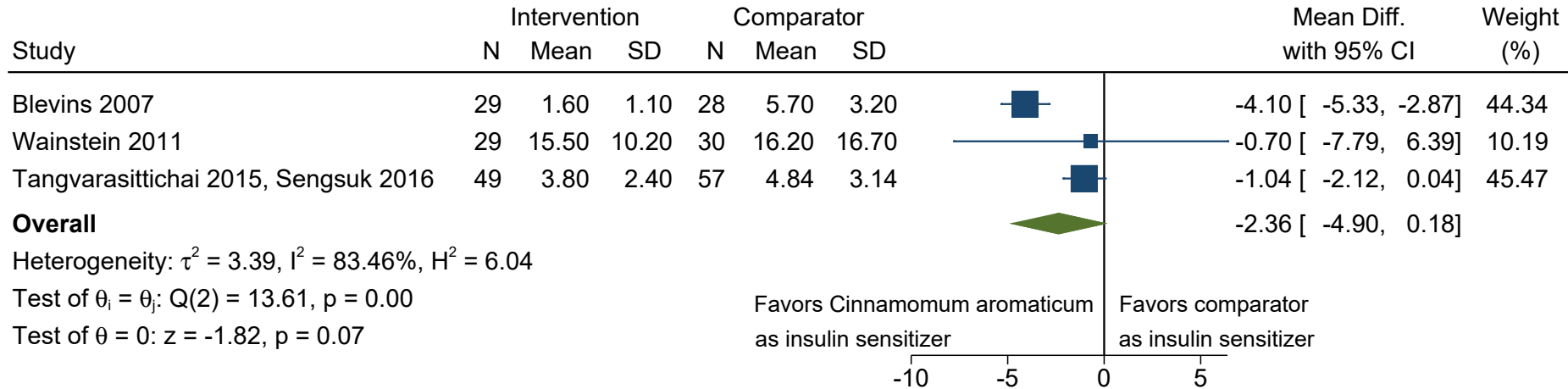

Supplement: Supplementary file 1 [file DataSheet1.zip › Supplementary Material/Forest and Funnel Plots/Cinnamomum aromaticum/Fasting insulin.pdf]

# Cinnamomum aromaticum - HbA1c

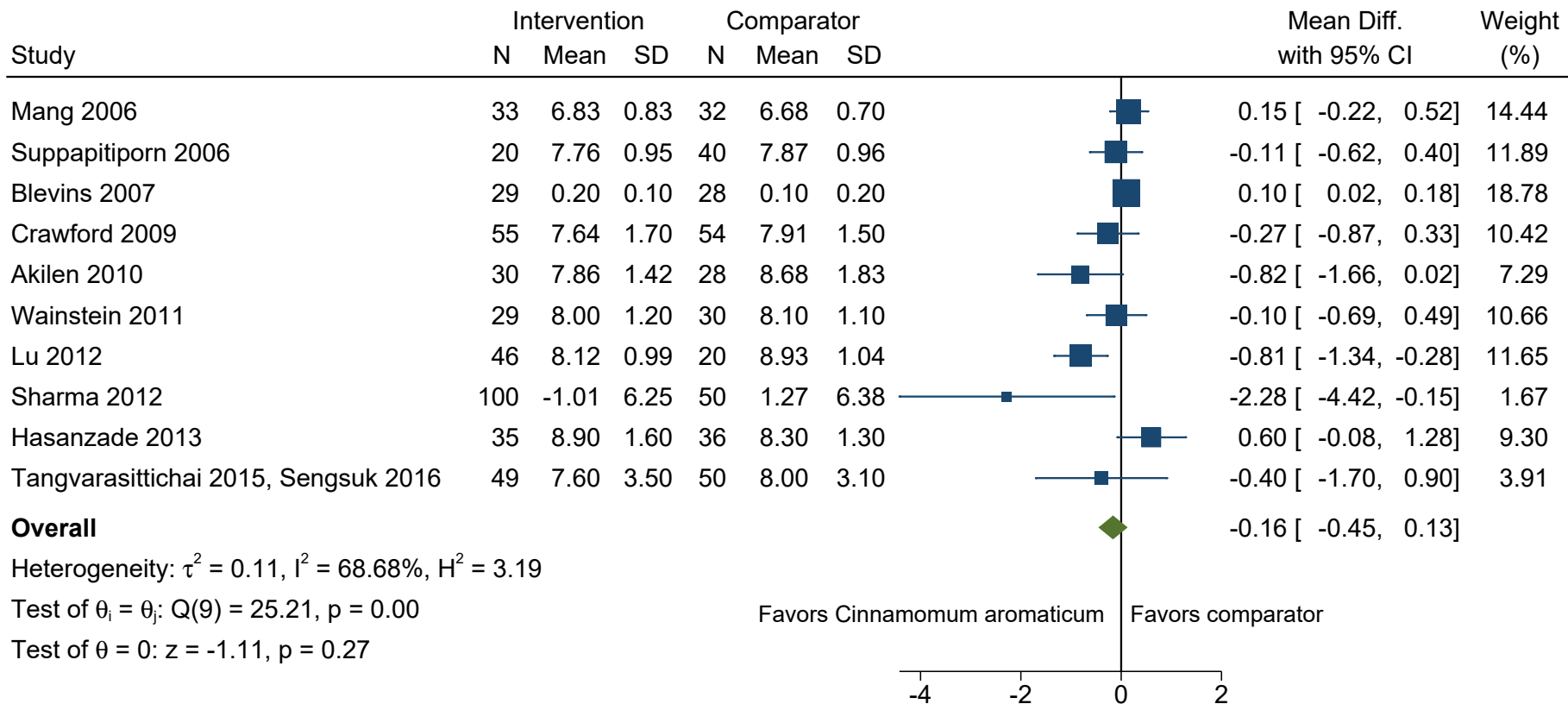

Supplement: Supplementary file 1 [file DataSheet1.zip › Supplementary Material/Forest and Funnel Plots/Cinnamomum aromaticum/HbA1c.pdf]

# Cinnamomum aromaticum - SBP

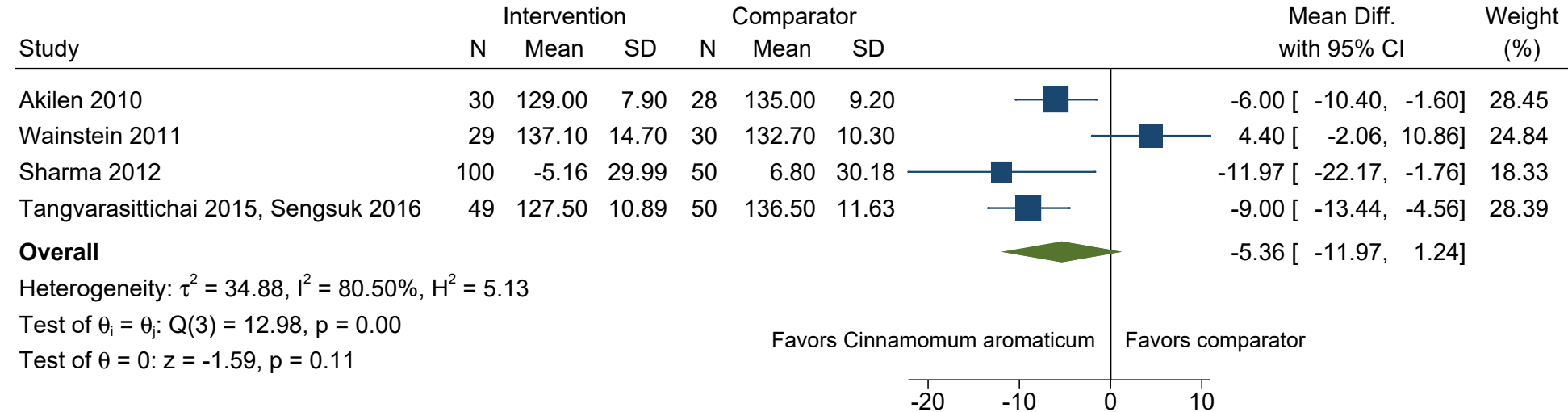

Random-effects REML model

Supplement: Supplementary file 1 [file DataSheet1.zip › Supplementary Material/Forest and Funnel Plots/Cinnamomum aromaticum/SBP.pdf]
