# Supplementary figures and images for: Effectiveness and Safety of Ayurvedic Medicines in Type 2 Diabetes Mellitus Management: A Systematic Review and Meta-Analysis (part 1 of 2)
Source: Front Pharmacol. 2022 Jun 8;13:821810. doi: 10.3389/fphar.2022.821810 (PMC9213670; doi:10.3389/fphar.2022.821810)

# Azadirachta indica - TG

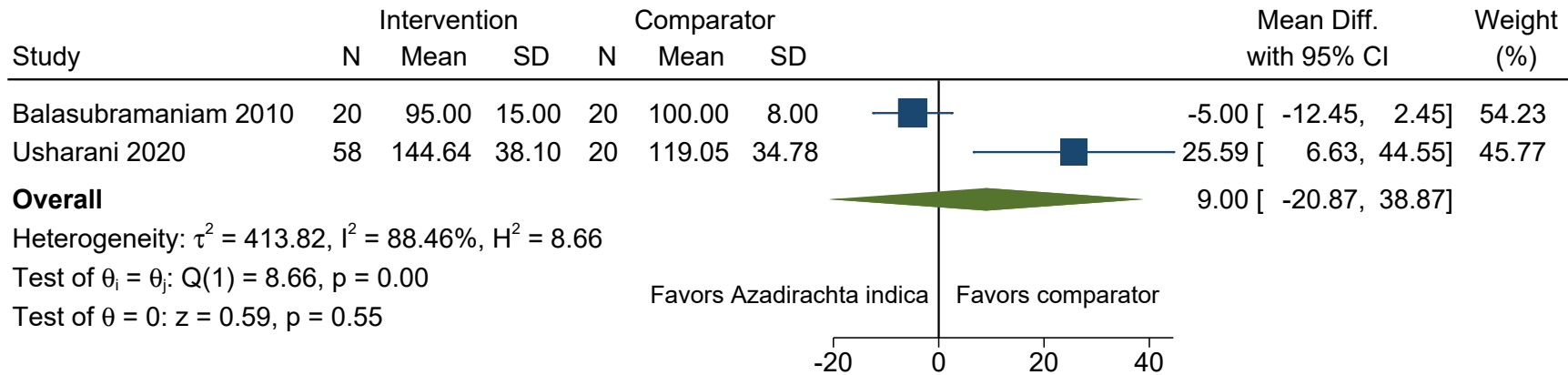

Random-effects REML model

Supplement: Supplementary file 1 [file DataSheet1.zip › Supplementary Material/Forest and Funnel Plots/Azadirachta indica/TG.pdf]

# Azadirachta indica - TC

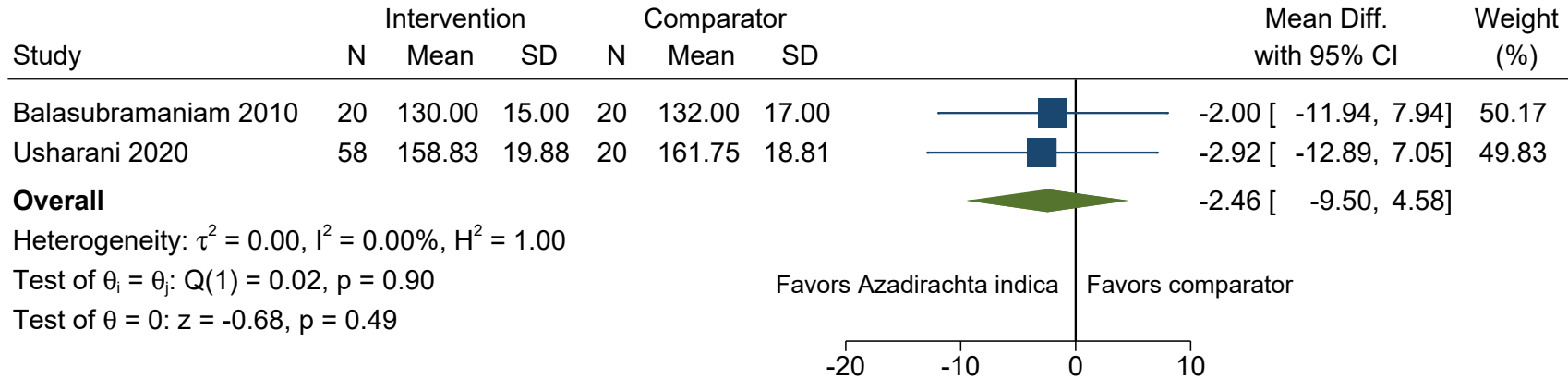

Random-effects REML model

Supplement: Supplementary file 1 [file DataSheet1.zip › Supplementary Material/Forest and Funnel Plots/Azadirachta indica/TC.pdf]

# Azadirachta indica - FBG

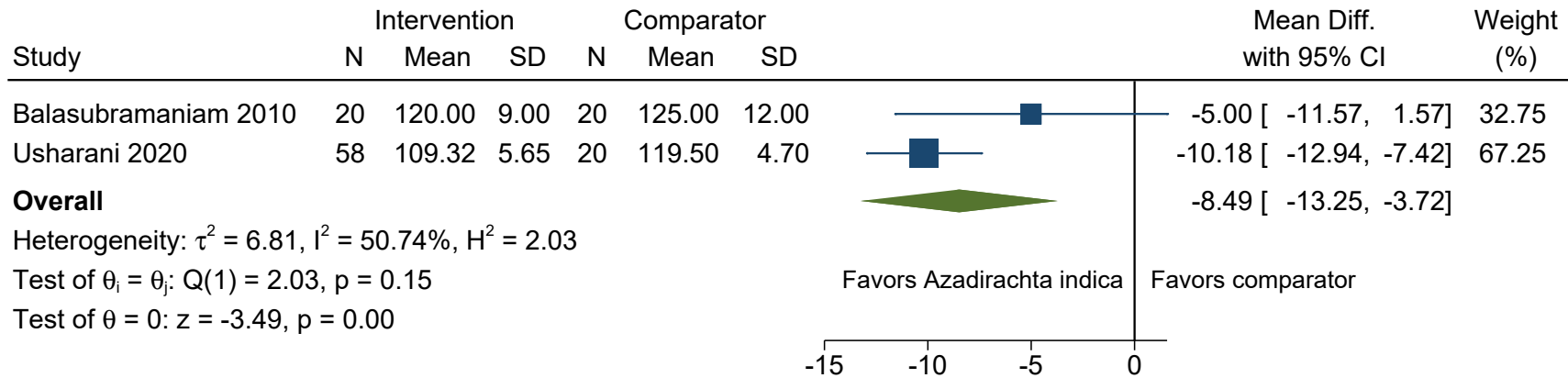

Random-effects REML model

Supplement: Supplementary file 1 [file DataSheet1.zip › Supplementary Material/Forest and Funnel Plots/Azadirachta indica/FBG.pdf]

# Azadirachta indica - HDL-C

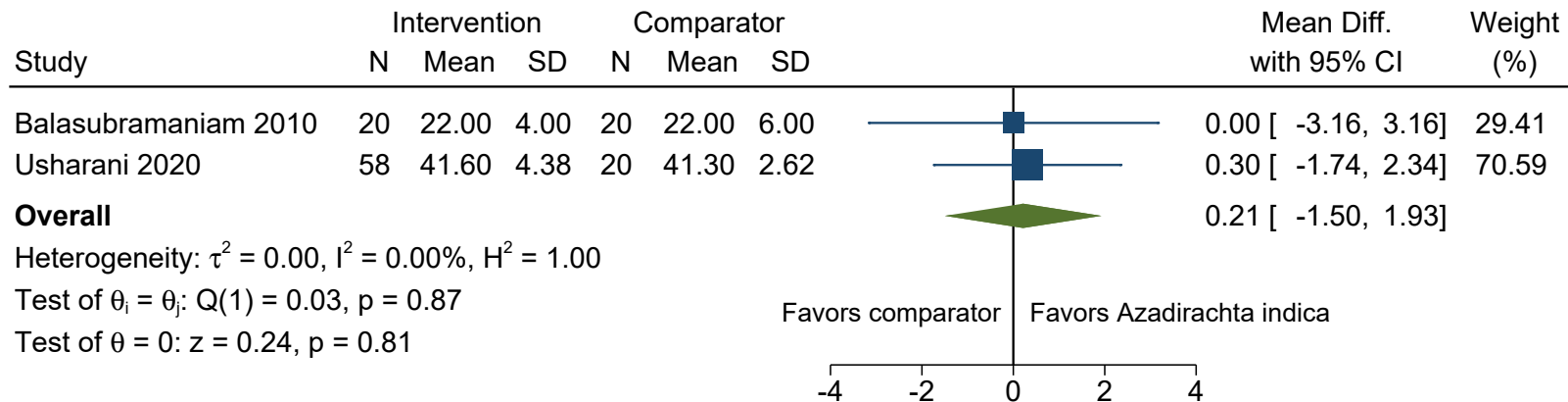

Random-effects REML model

Supplement: Supplementary file 1 [file DataSheet1.zip › Supplementary Material/Forest and Funnel Plots/Azadirachta indica/HDL-C.pdf]

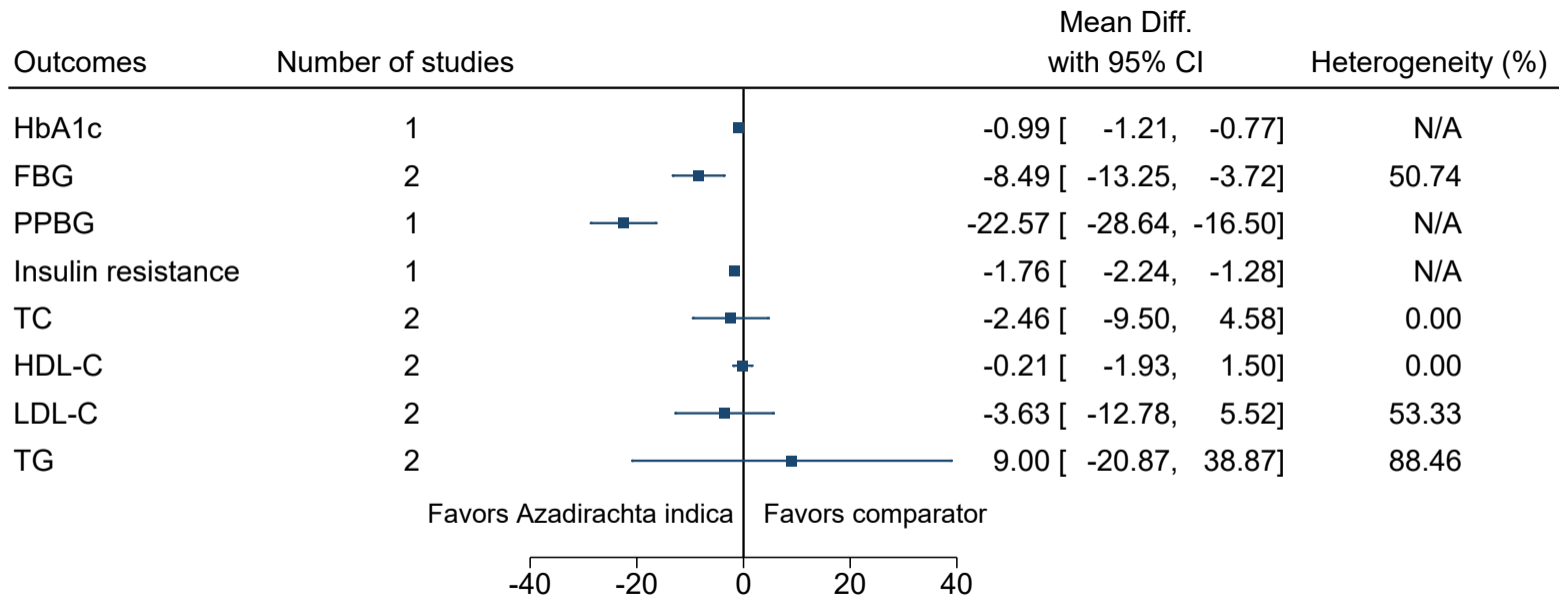

Supplement: Supplementary file 1 [file DataSheet1.zip › Supplementary Material/Forest and Funnel Plots/Azadirachta indica/Azadirachta indica.pdf]

# Azadirachta indica - LDL-C

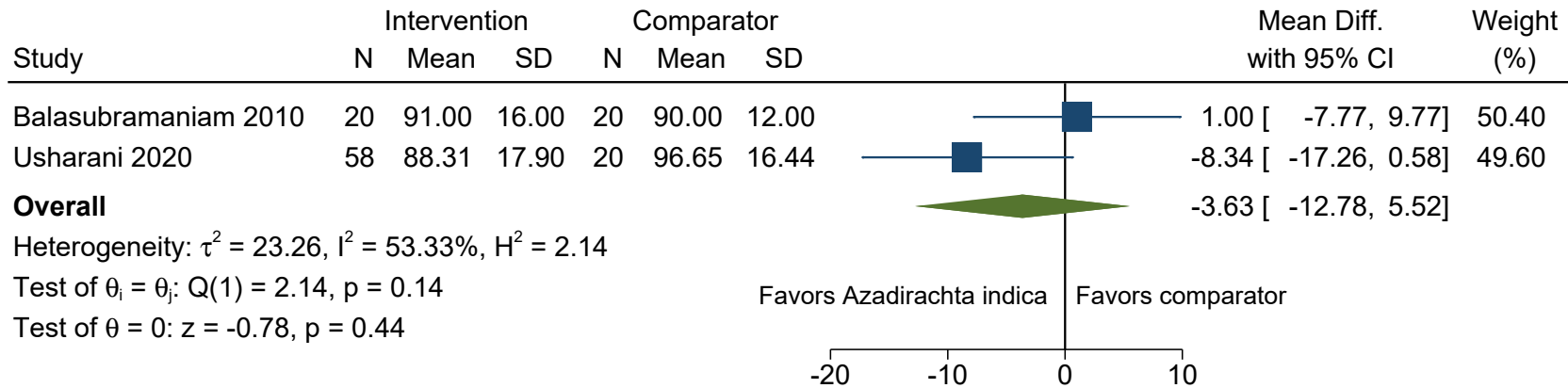

Random-effects REML model

Supplement: Supplementary file 1 [file DataSheet1.zip › Supplementary Material/Forest and Funnel Plots/Azadirachta indica/LDL-C.pdf]

# Boswellia serrata - TG

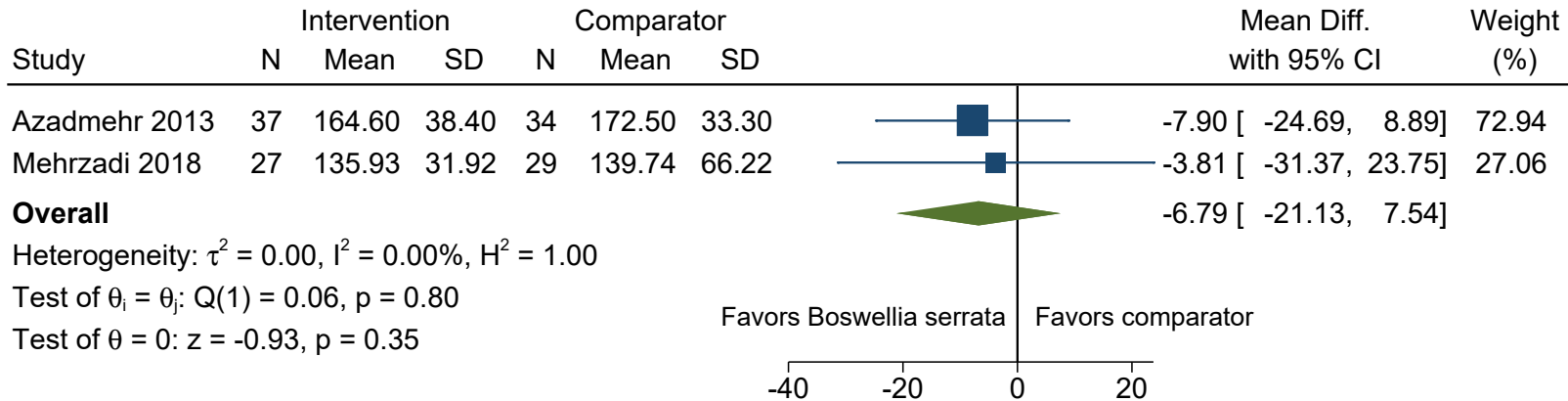

Random-effects REML model

Supplement: Supplementary file 1 [file DataSheet1.zip › Supplementary Material/Forest and Funnel Plots/Boswellia serrata/TG.pdf]

# Boswellia serrata - TC

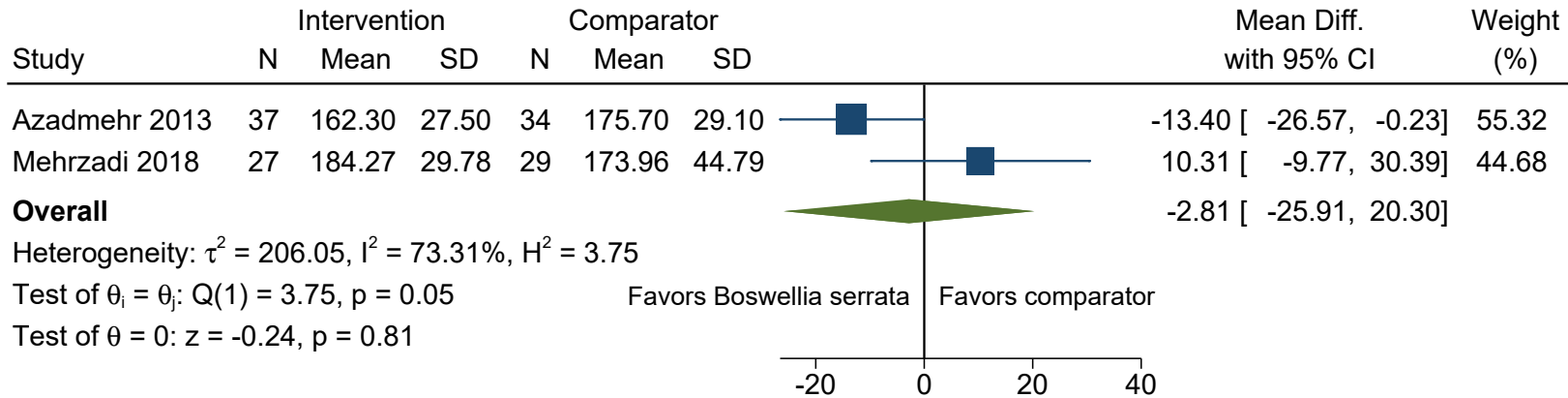

Random-effects REML model

Supplement: Supplementary file 1 [file DataSheet1.zip › Supplementary Material/Forest and Funnel Plots/Boswellia serrata/TC.pdf]

# Boswellia serrata - FBG

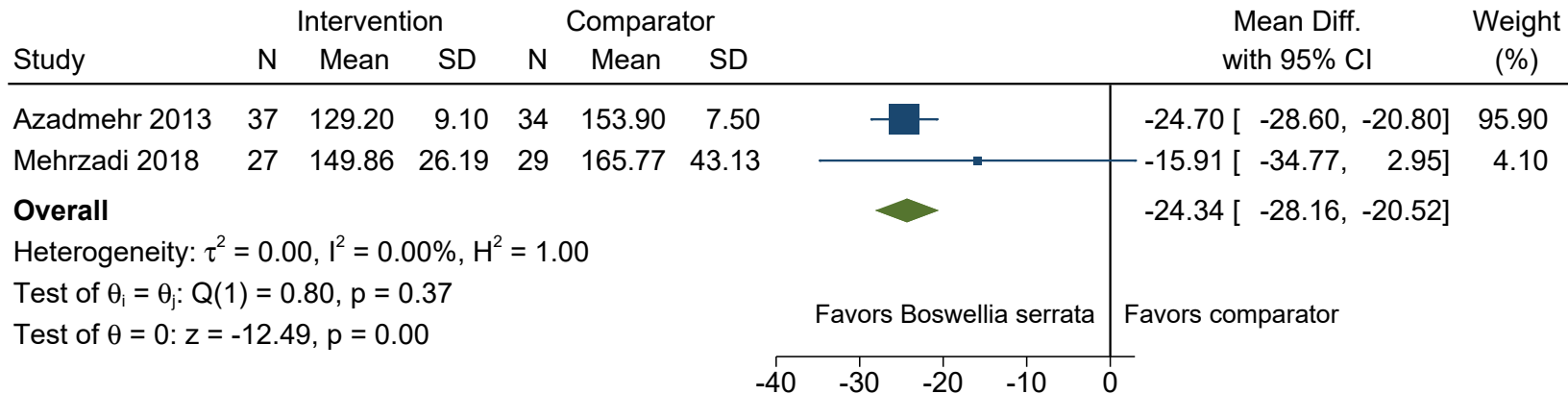

Random-effects REML model

Supplement: Supplementary file 1 [file DataSheet1.zip › Supplementary Material/Forest and Funnel Plots/Boswellia serrata/FBG.pdf]

# Boswellia serrata - Fasting insulin

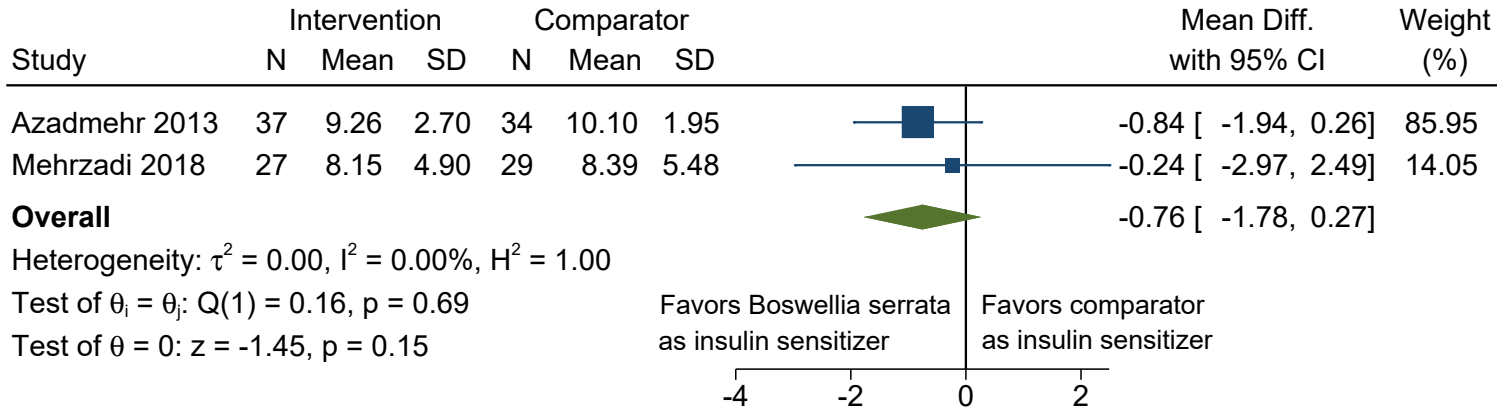

Random-effects REML model

Supplement: Supplementary file 1 [file DataSheet1.zip › Supplementary Material/Forest and Funnel Plots/Boswellia serrata/Fasting insulin.pdf]

# Boswellia serrata - HbA1c

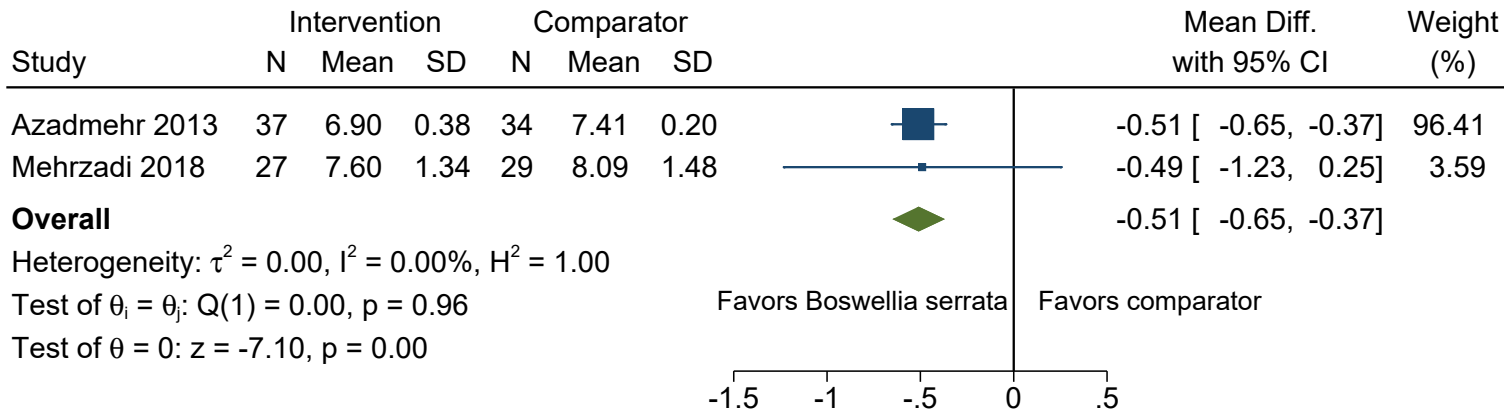

Random-effects REML model

Supplement: Supplementary file 1 [file DataSheet1.zip › Supplementary Material/Forest and Funnel Plots/Boswellia serrata/HbA1c.pdf]

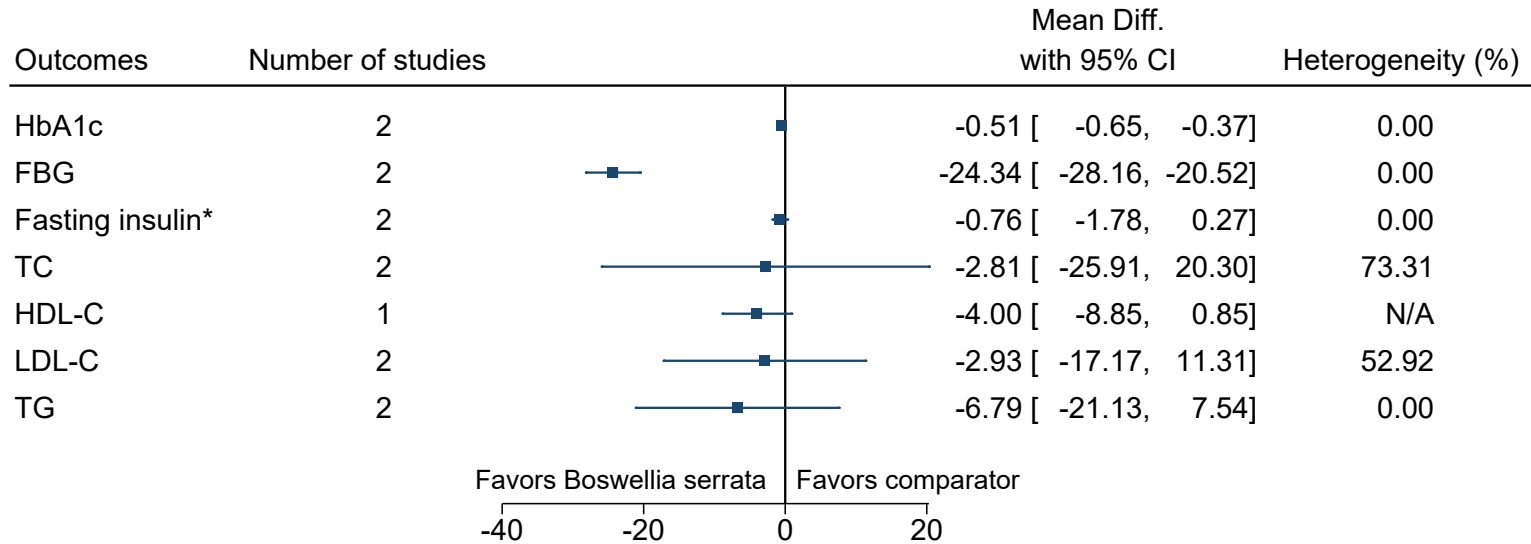

\*Favours intervention/comparator as insulin sensitizer

Supplement: Supplementary file 1 [file DataSheet1.zip › Supplementary Material/Forest and Funnel Plots/Boswellia serrata/Boswellia serrata.pdf]

# Boswellia serrata - LDL-C

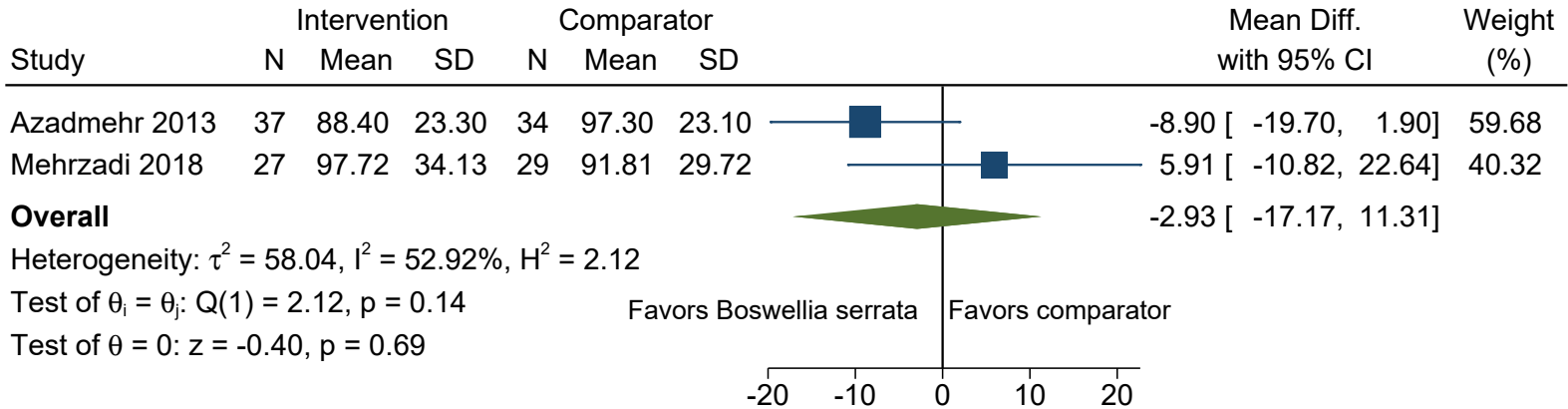

Random-effects REML model

Supplement: Supplementary file 1 [file DataSheet1.zip › Supplementary Material/Forest and Funnel Plots/Boswellia serrata/LDL-C.pdf]

# Curcuma longa - Waist circumference

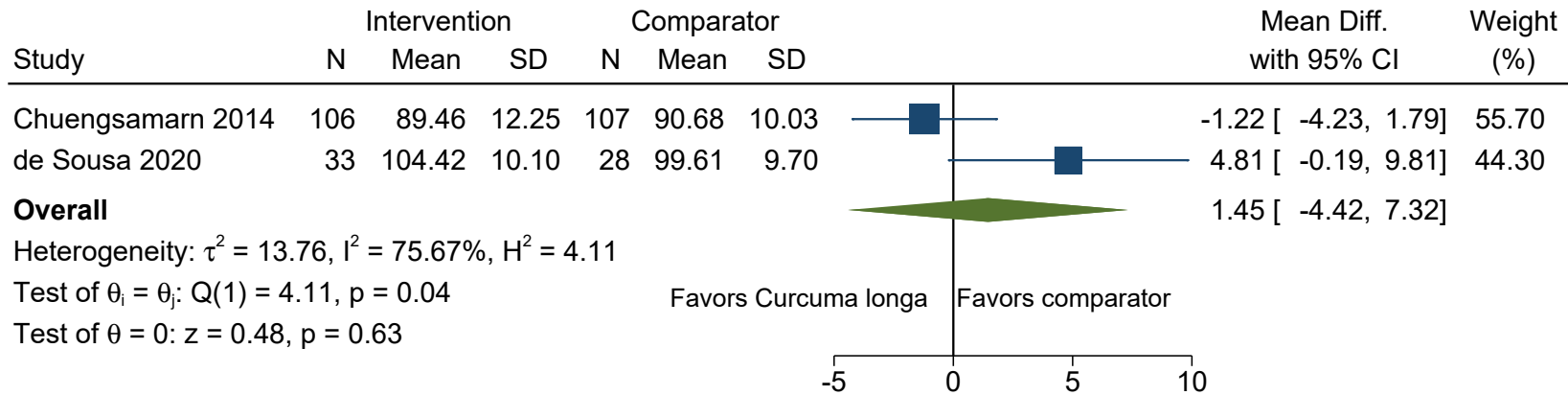

Random-effects REML model

Supplement: Supplementary file 1 [file DataSheet1.zip › Supplementary Material/Forest and Funnel Plots/Curcuma longa/Waist circumference.pdf]

# Curcuma longa - TG

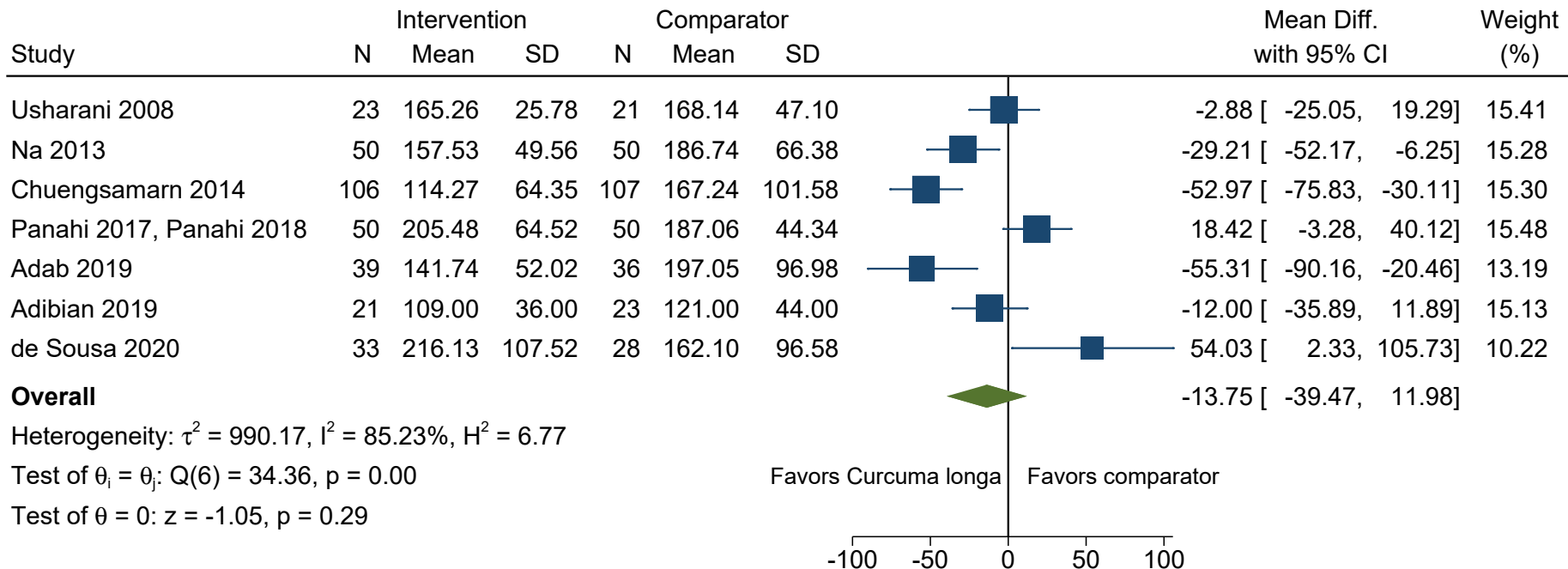

Supplement: Supplementary file 1 [file DataSheet1.zip › Supplementary Material/Forest and Funnel Plots/Curcuma longa/TG.pdf]

# Curcuma longa - BMI

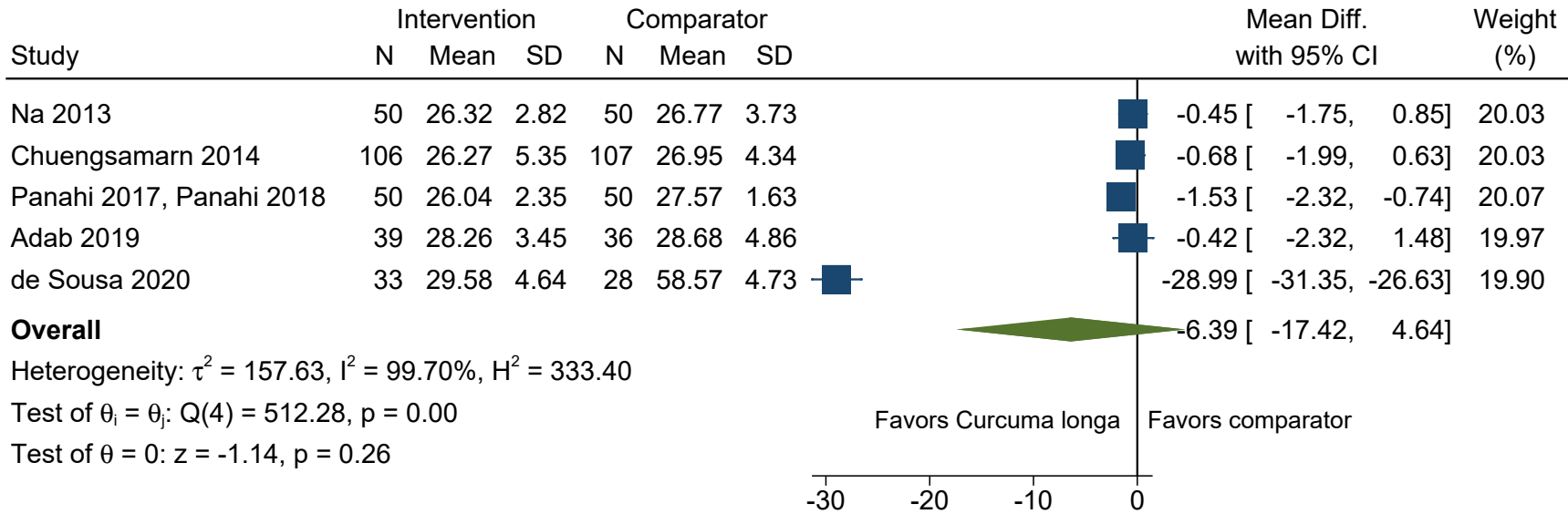

Supplement: Supplementary file 1 [file DataSheet1.zip › Supplementary Material/Forest and Funnel Plots/Curcuma longa/BMI.pdf]

# Curcuma longa - TC

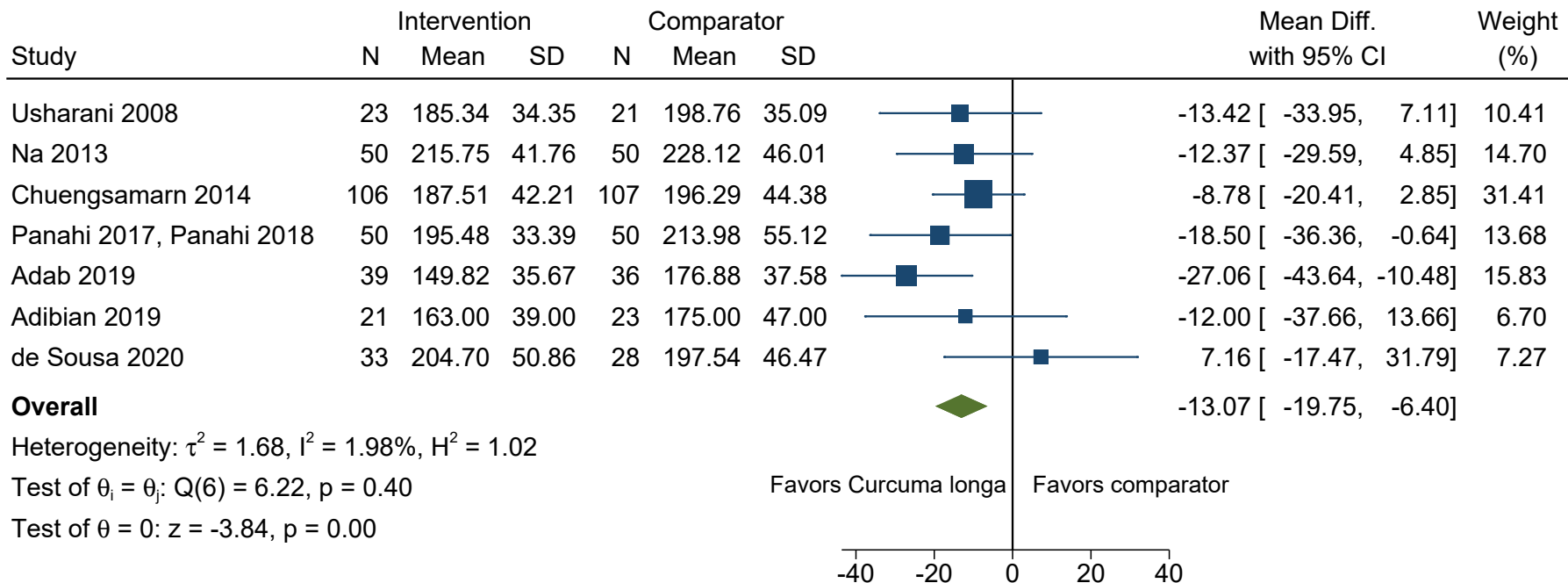

Supplement: Supplementary file 1 [file DataSheet1.zip › Supplementary Material/Forest and Funnel Plots/Curcuma longa/TC.pdf]

# Curcuma longa - FBG

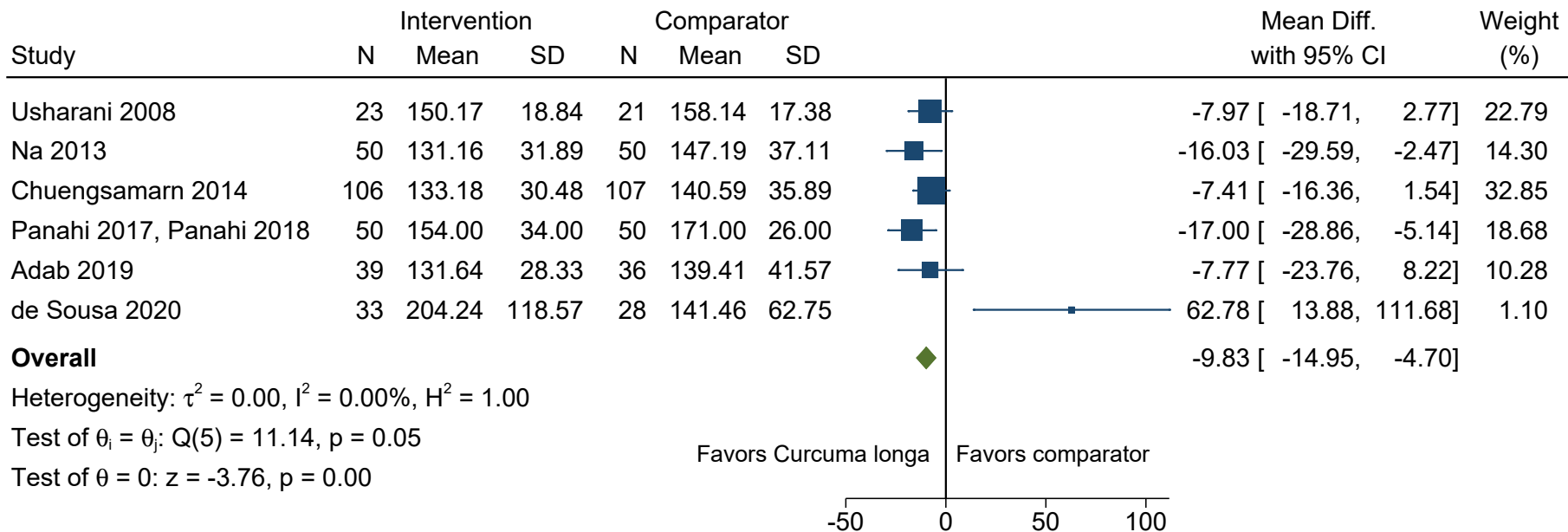

Supplement: Supplementary file 1 [file DataSheet1.zip › Supplementary Material/Forest and Funnel Plots/Curcuma longa/FBG.pdf]

# Curcuma longa - HDL-C

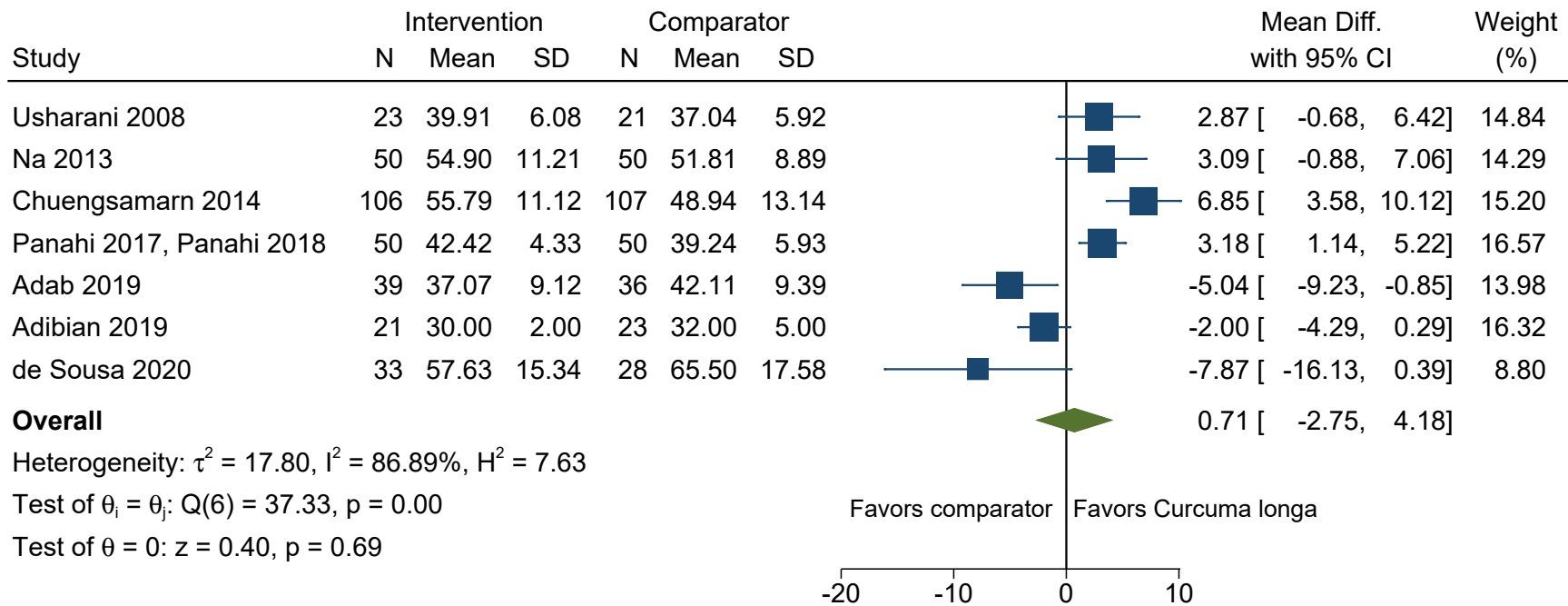

Supplement: Supplementary file 1 [file DataSheet1.zip › Supplementary Material/Forest and Funnel Plots/Curcuma longa/HDL-C.pdf]

# Curcuma longa - DBP

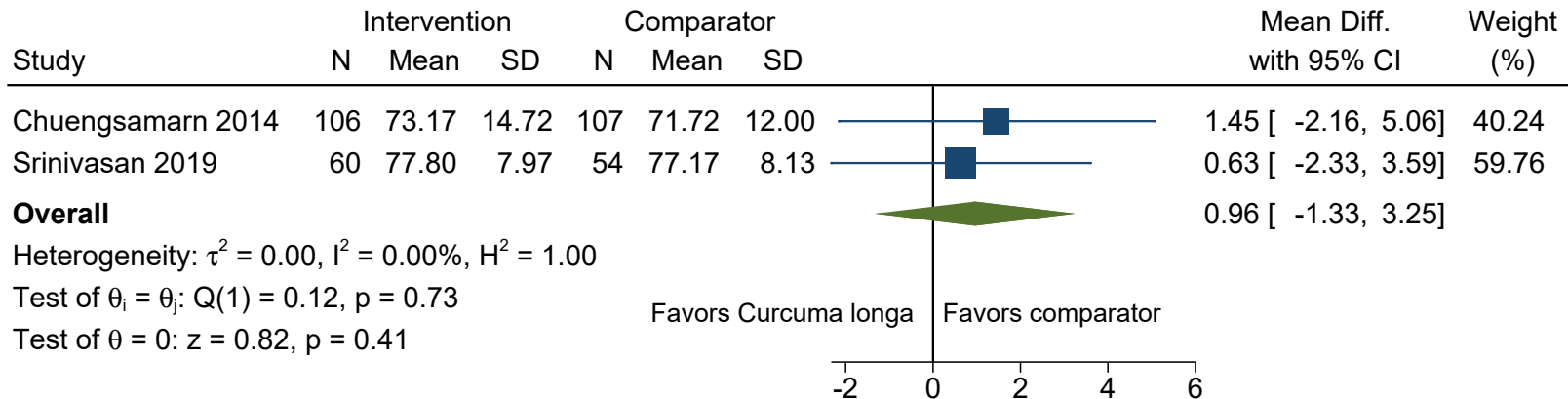

Random-effects REML model

Supplement: Supplementary file 1 [file DataSheet1.zip › Supplementary Material/Forest and Funnel Plots/Curcuma longa/DBP.pdf]

# Curcuma longa - Body weight

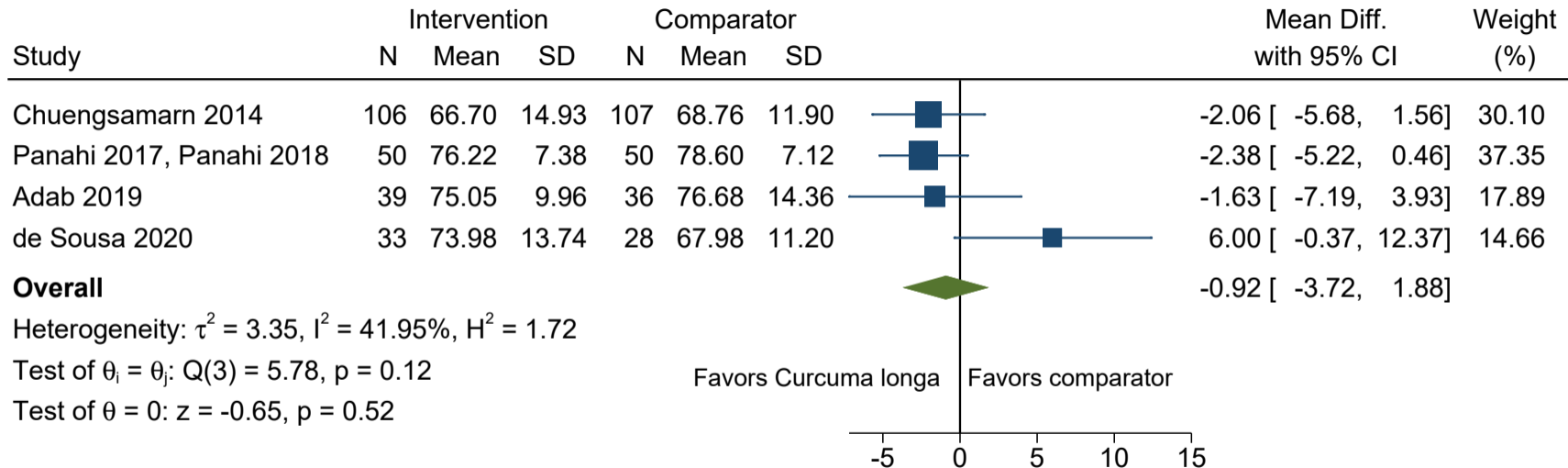

Random-effects REML model

Supplement: Supplementary file 1 [file DataSheet1.zip › Supplementary Material/Forest and Funnel Plots/Curcuma longa/Body weight.pdf]

# Curcuma longa - Insulin resistance

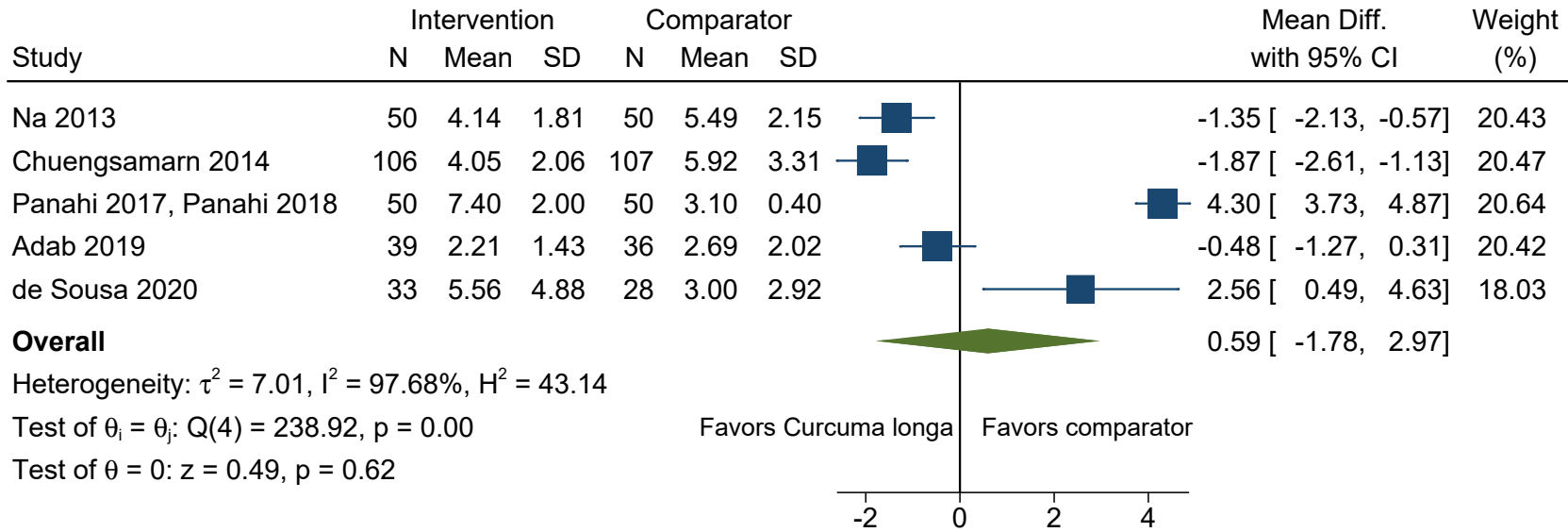

Supplement: Supplementary file 1 [file DataSheet1.zip › Supplementary Material/Forest and Funnel Plots/Curcuma longa/Insulin resistance.pdf]

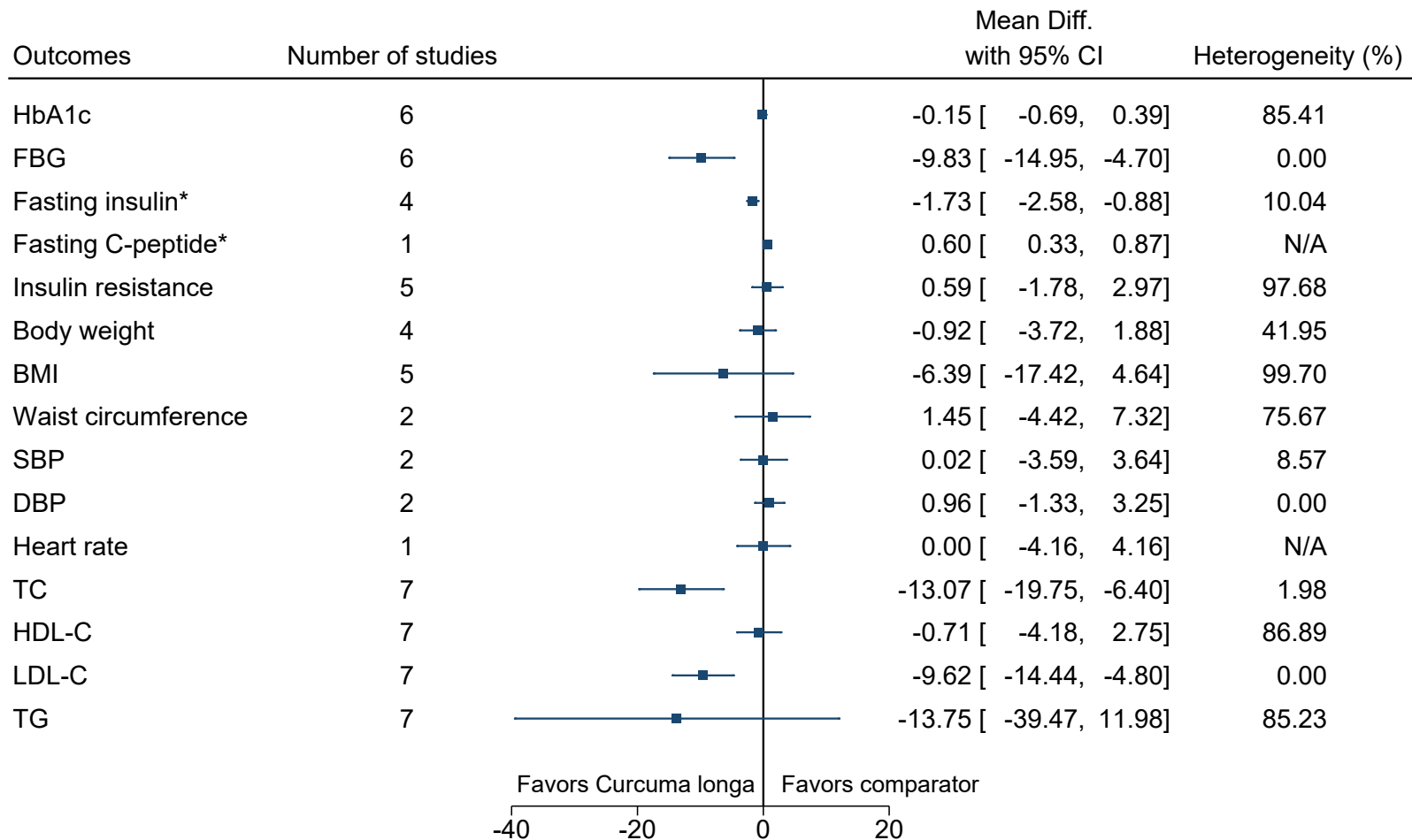

\*Favors intervention/comparator as insulin sensitizer

Supplement: Supplementary file 1 [file DataSheet1.zip › Supplementary Material/Forest and Funnel Plots/Curcuma longa/Curcuma longa.pdf]

# Curcuma longa - Fasting insulin

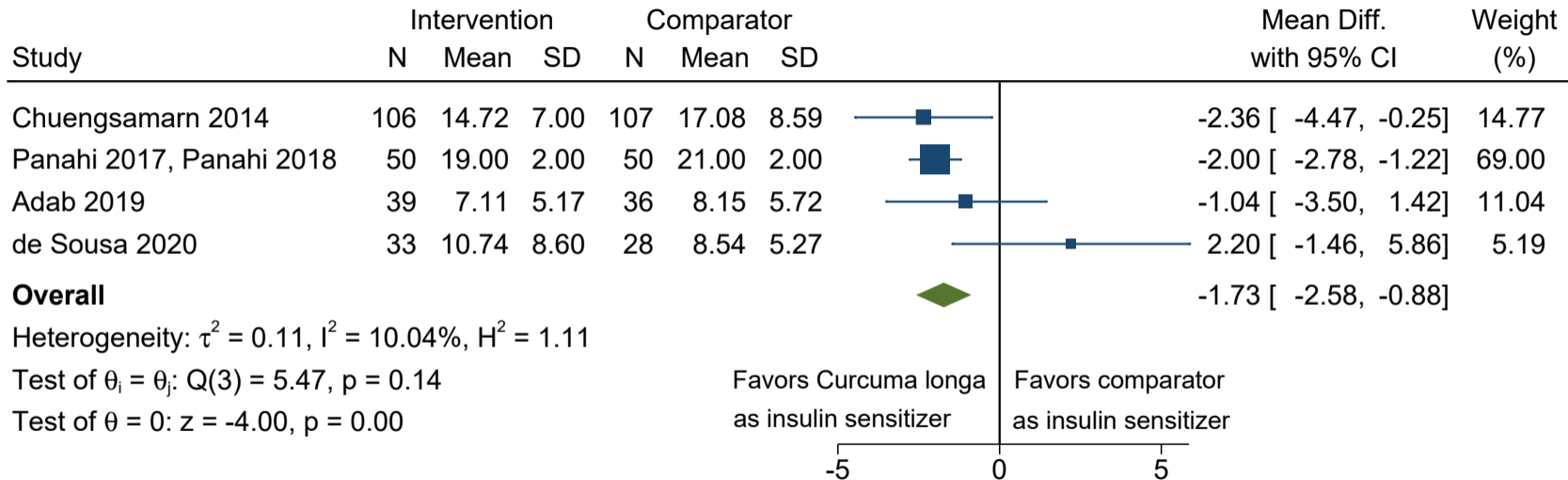

Random-effects REML model

Supplement: Supplementary file 1 [file DataSheet1.zip › Supplementary Material/Forest and Funnel Plots/Curcuma longa/Fasting insulin.pdf]

# Curcuma longa - HbA1c

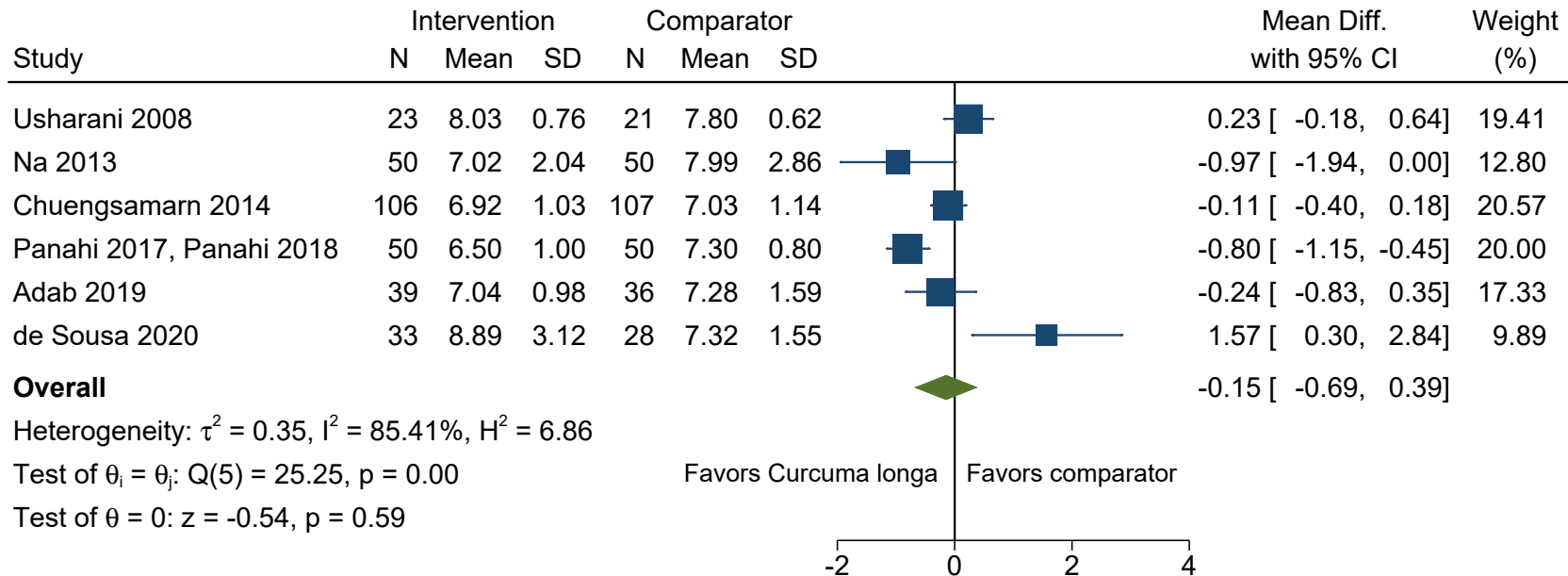

Supplement: Supplementary file 1 [file DataSheet1.zip › Supplementary Material/Forest and Funnel Plots/Curcuma longa/HbA1c.pdf]

# Curcuma longa - SBP

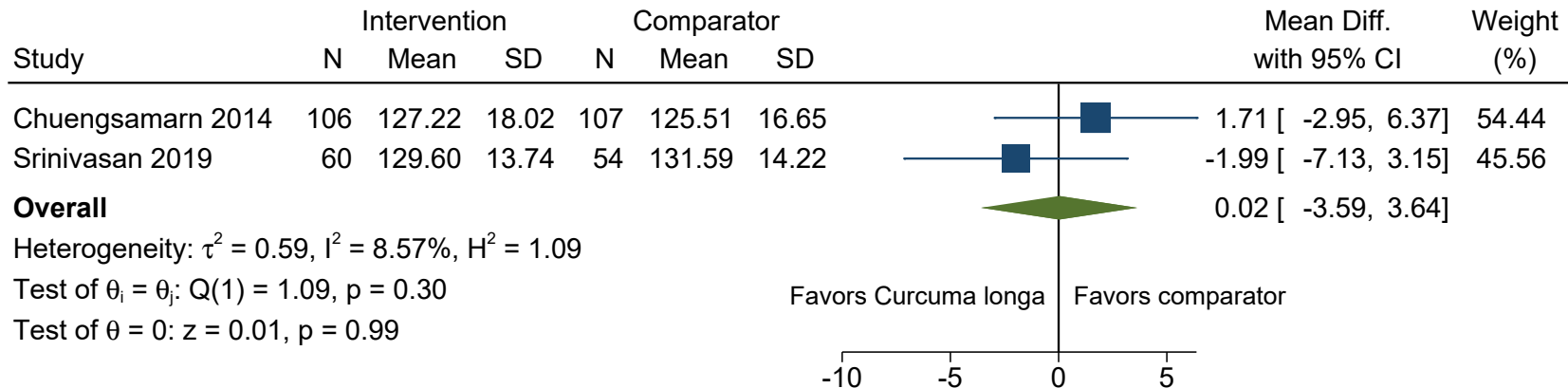

Random-effects REML model

Supplement: Supplementary file 1 [file DataSheet1.zip › Supplementary Material/Forest and Funnel Plots/Curcuma longa/SBP.pdf]

# Curcuma longa - LDL-C

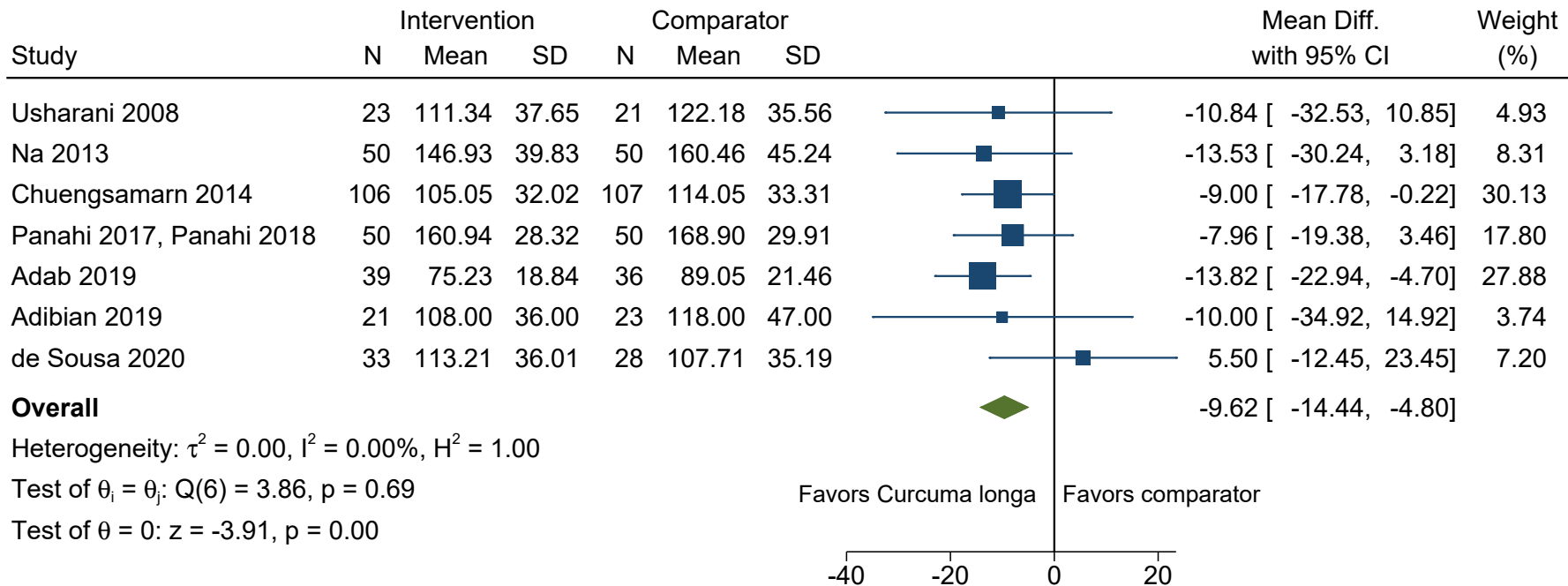

Supplement: Supplementary file 1 [file DataSheet1.zip › Supplementary Material/Forest and Funnel Plots/Curcuma longa/LDL-C.pdf]

# Camellia sinensis - Waist circumference

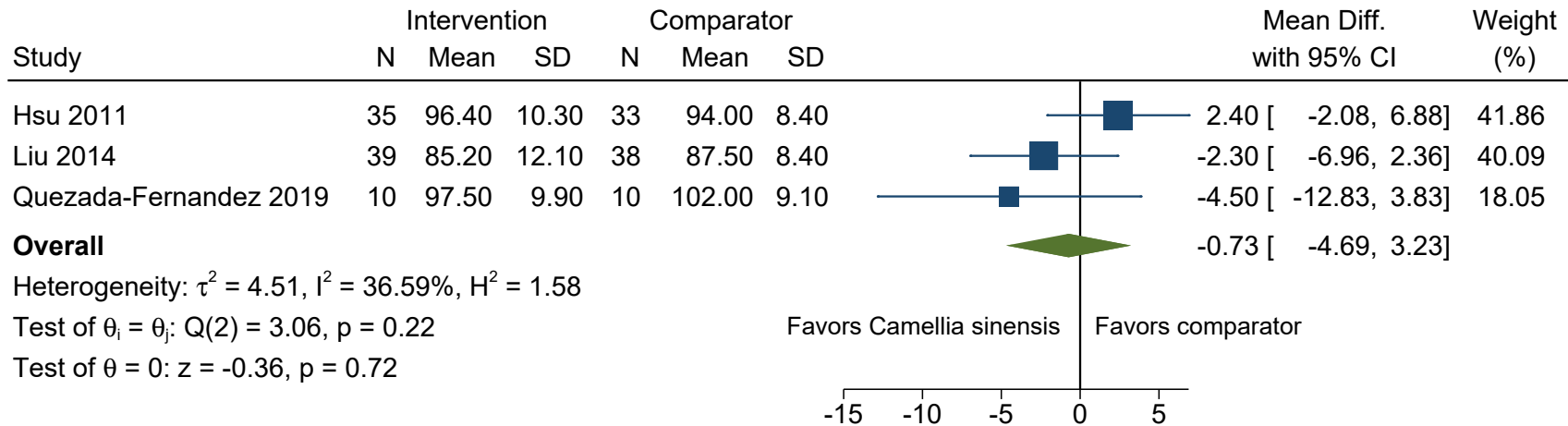

Random-effects REML model

Supplement: Supplementary file 1 [file DataSheet1.zip › Supplementary Material/Forest and Funnel Plots/Camellia sinensis/Waist circumference.pdf]

# Camellia sinensis - TG

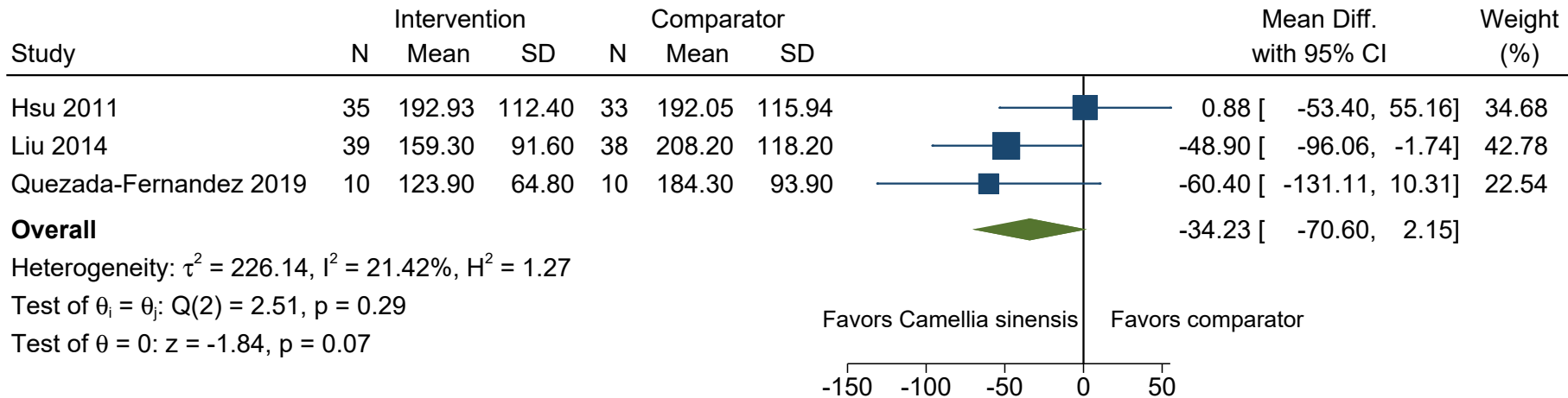

Random-effects REML model

Supplement: Supplementary file 1 [file DataSheet1.zip › Supplementary Material/Forest and Funnel Plots/Camellia sinensis/TG.pdf]

# Camellia sinensis - BMI

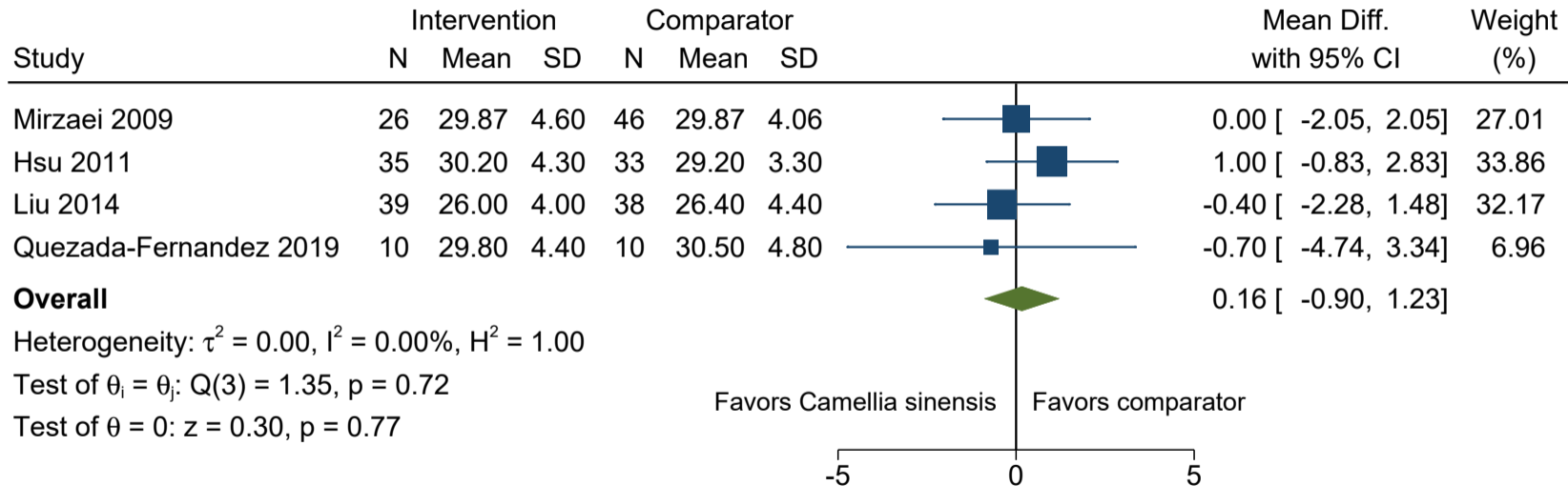

Random-effects REML model

Supplement: Supplementary file 1 [file DataSheet1.zip › Supplementary Material/Forest and Funnel Plots/Camellia sinensis/BMI.pdf]

# Camellia sinensis - TC

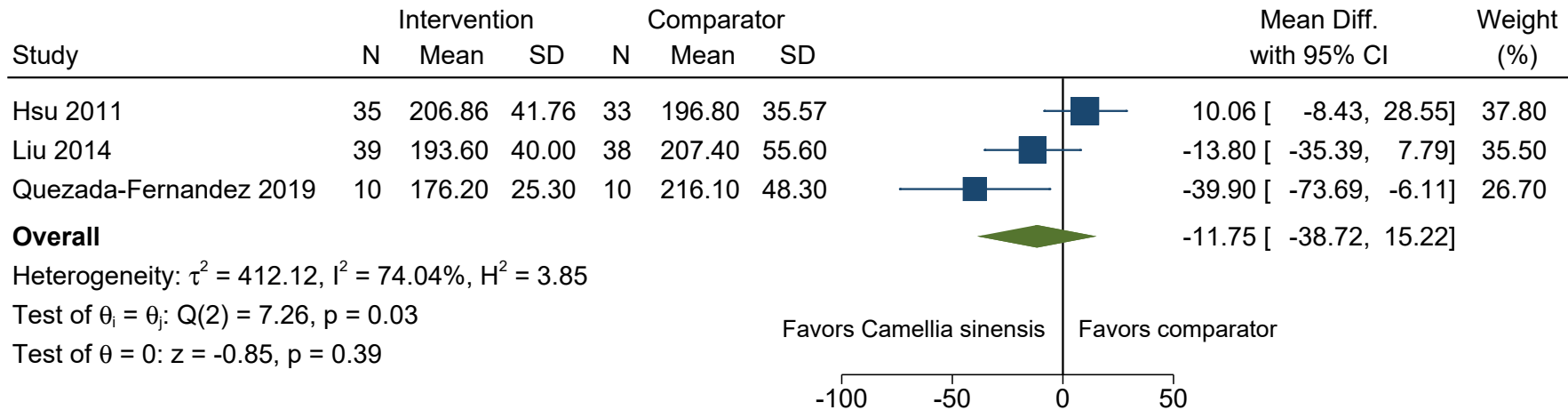

Random-effects REML model

Supplement: Supplementary file 1 [file DataSheet1.zip › Supplementary Material/Forest and Funnel Plots/Camellia sinensis/TC.pdf]

# Camellia sinensis - FBG

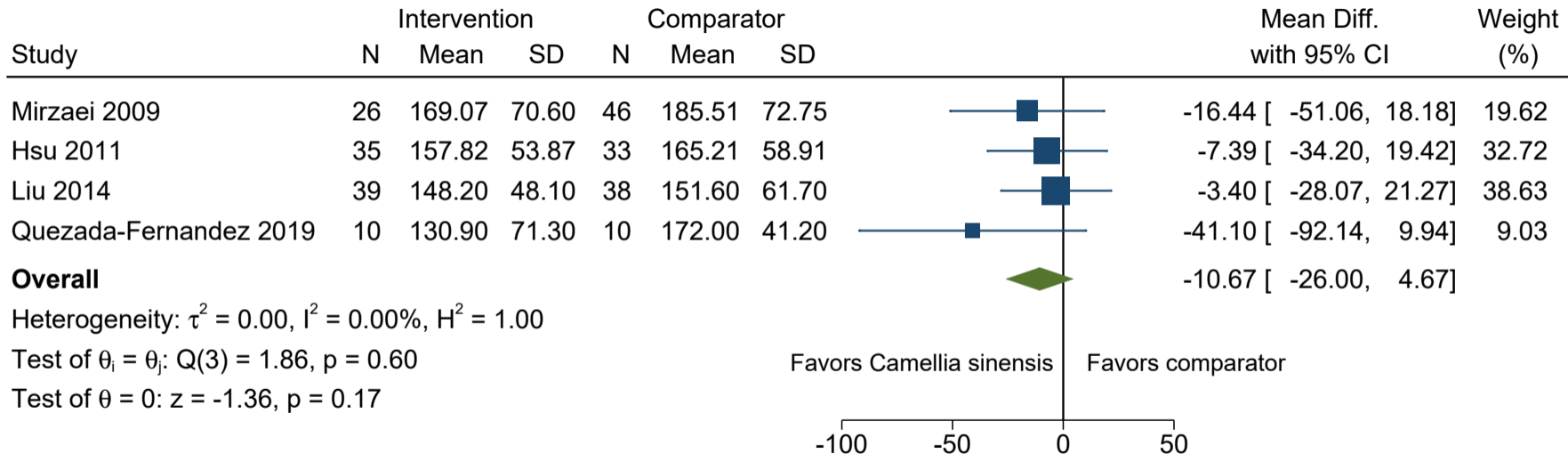

Random-effects REML model

Supplement: Supplementary file 1 [file DataSheet1.zip › Supplementary Material/Forest and Funnel Plots/Camellia sinensis/FBG.pdf]

# Camellia sinensis - HDL-C

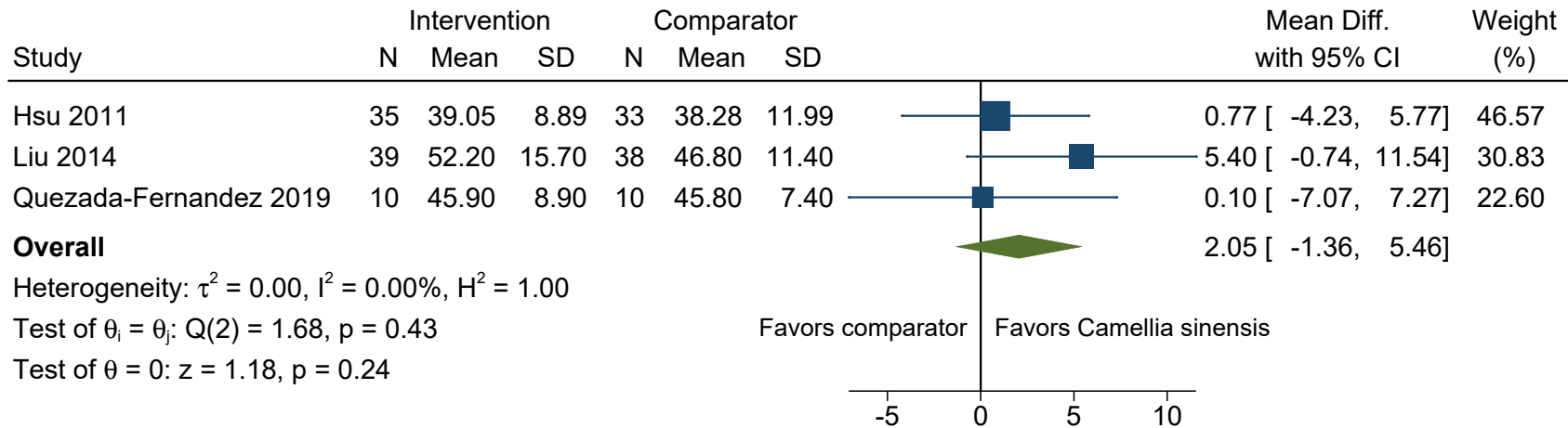

Random-effects REML model

Supplement: Supplementary file 1 [file DataSheet1.zip › Supplementary Material/Forest and Funnel Plots/Camellia sinensis/HDL-C.pdf]

# Camellia sinensis - DBP

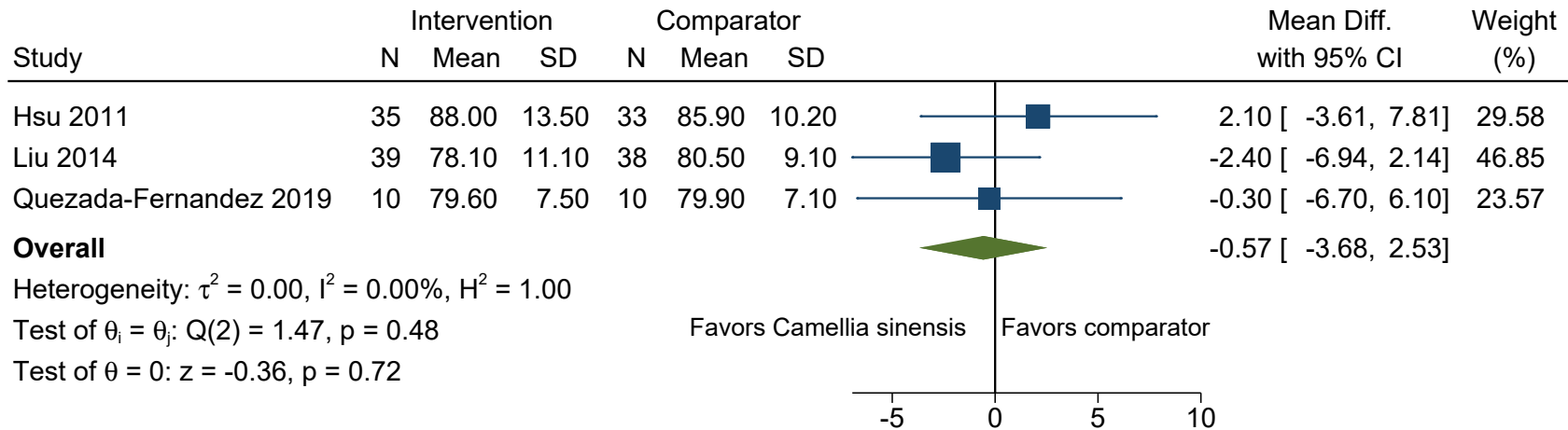

Random-effects REML model

Supplement: Supplementary file 1 [file DataSheet1.zip › Supplementary Material/Forest and Funnel Plots/Camellia sinensis/DBP.pdf]

# Camellia sinensis - Body weight

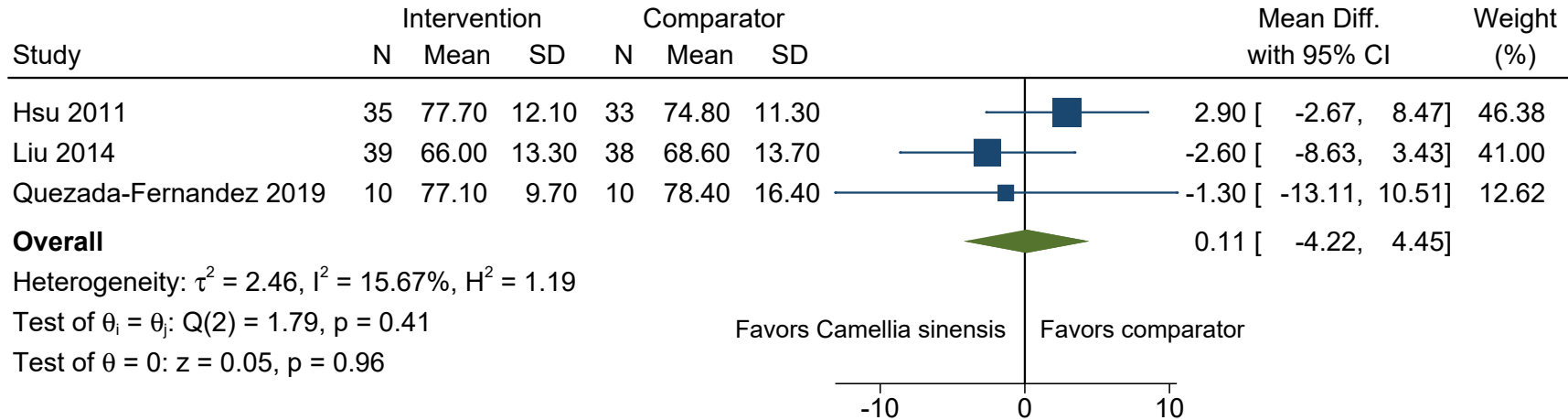

Random-effects REML model

Supplement: Supplementary file 1 [file DataSheet1.zip › Supplementary Material/Forest and Funnel Plots/Camellia sinensis/Body weight.pdf]

# Camellia sinensis - Insulin resistance

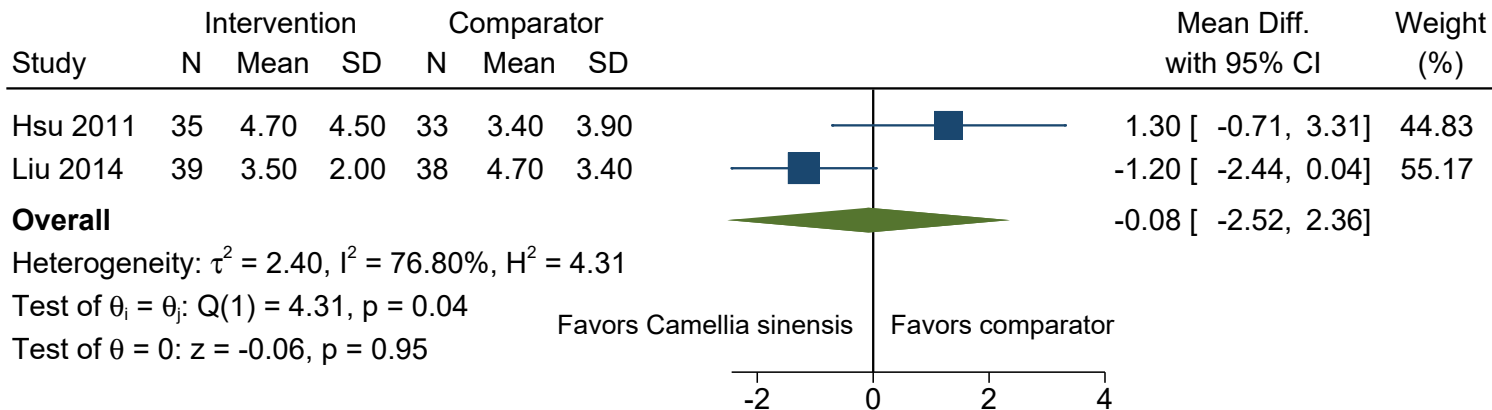

Random-effects REML model

Supplement: Supplementary file 1 [file DataSheet1.zip › Supplementary Material/Forest and Funnel Plots/Camellia sinensis/Insulin resistance.pdf]

# Camellia sinensis - Fasting insulin

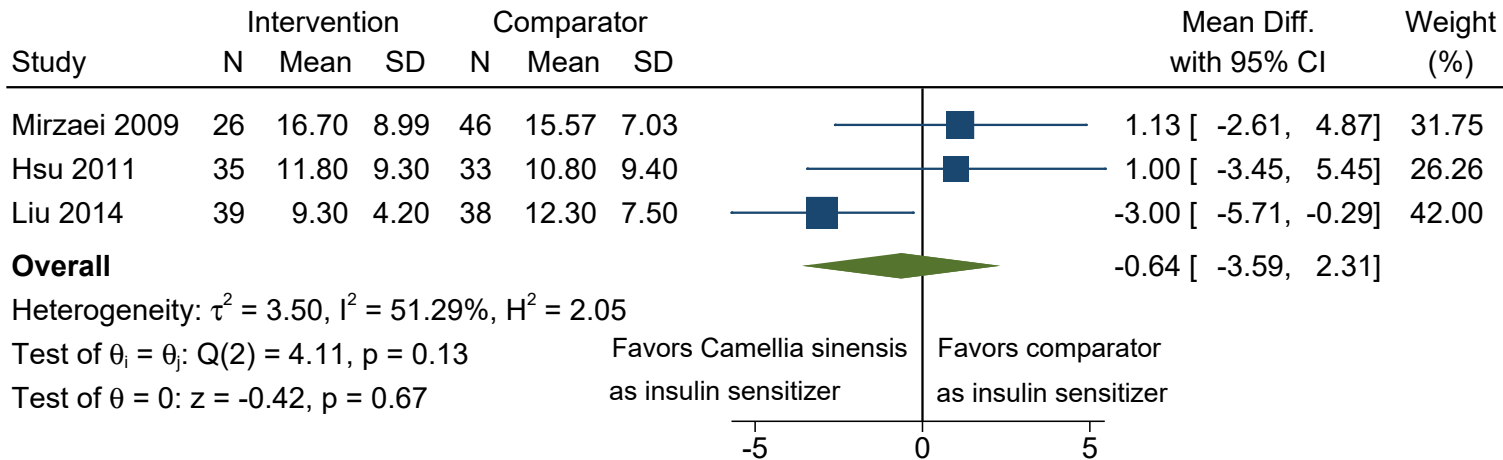

Supplement: Supplementary file 1 [file DataSheet1.zip › Supplementary Material/Forest and Funnel Plots/Camellia sinensis/Fasting insulin.pdf]

# Camellia sinensis - HbA1c

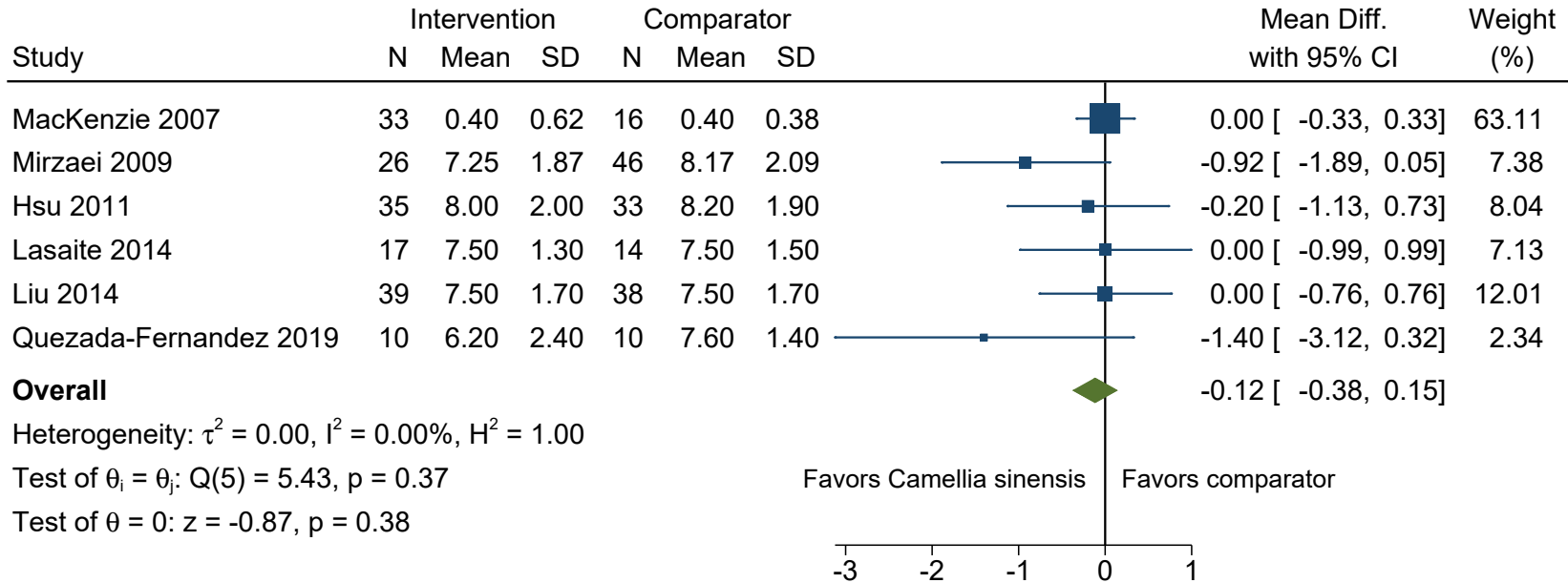

Random-effects REML model

Supplement: Supplementary file 1 [file DataSheet1.zip › Supplementary Material/Forest and Funnel Plots/Camellia sinensis/HbA1c.pdf]

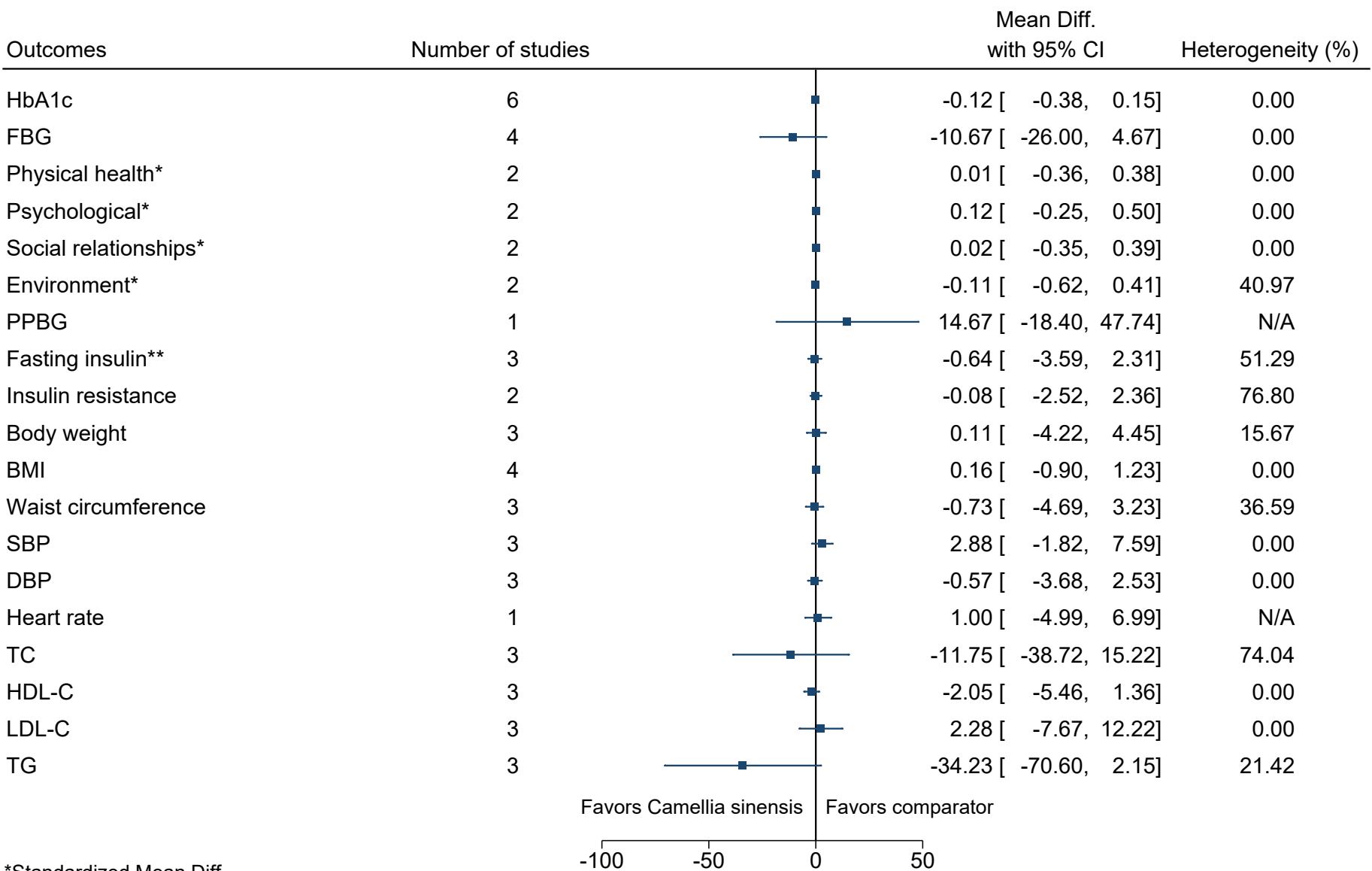

\*Standardized Mean Diff.  
\*\*Favors intervention/comparator as insulin sensitizer

Supplement: Supplementary file 1 [file DataSheet1.zip › Supplementary Material/Forest and Funnel Plots/Camellia sinensis/Camellia sinensis.pdf]

# Camellia sinensis - SBP

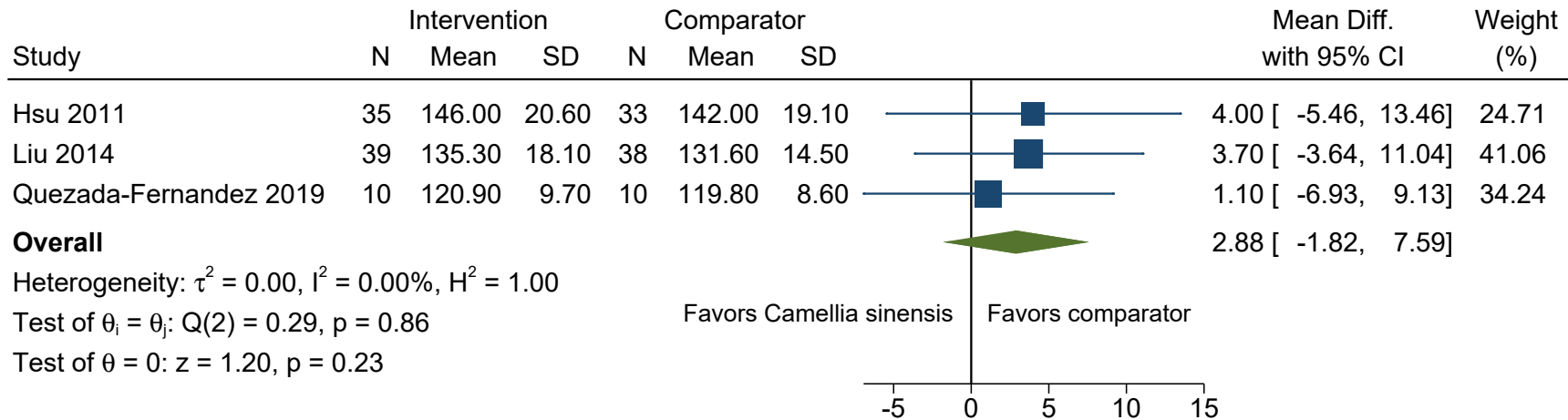

Random-effects REML model

Supplement: Supplementary file 1 [file DataSheet1.zip › Supplementary Material/Forest and Funnel Plots/Camellia sinensis/SBP.pdf]

# Camellia sinensis - HRQoL

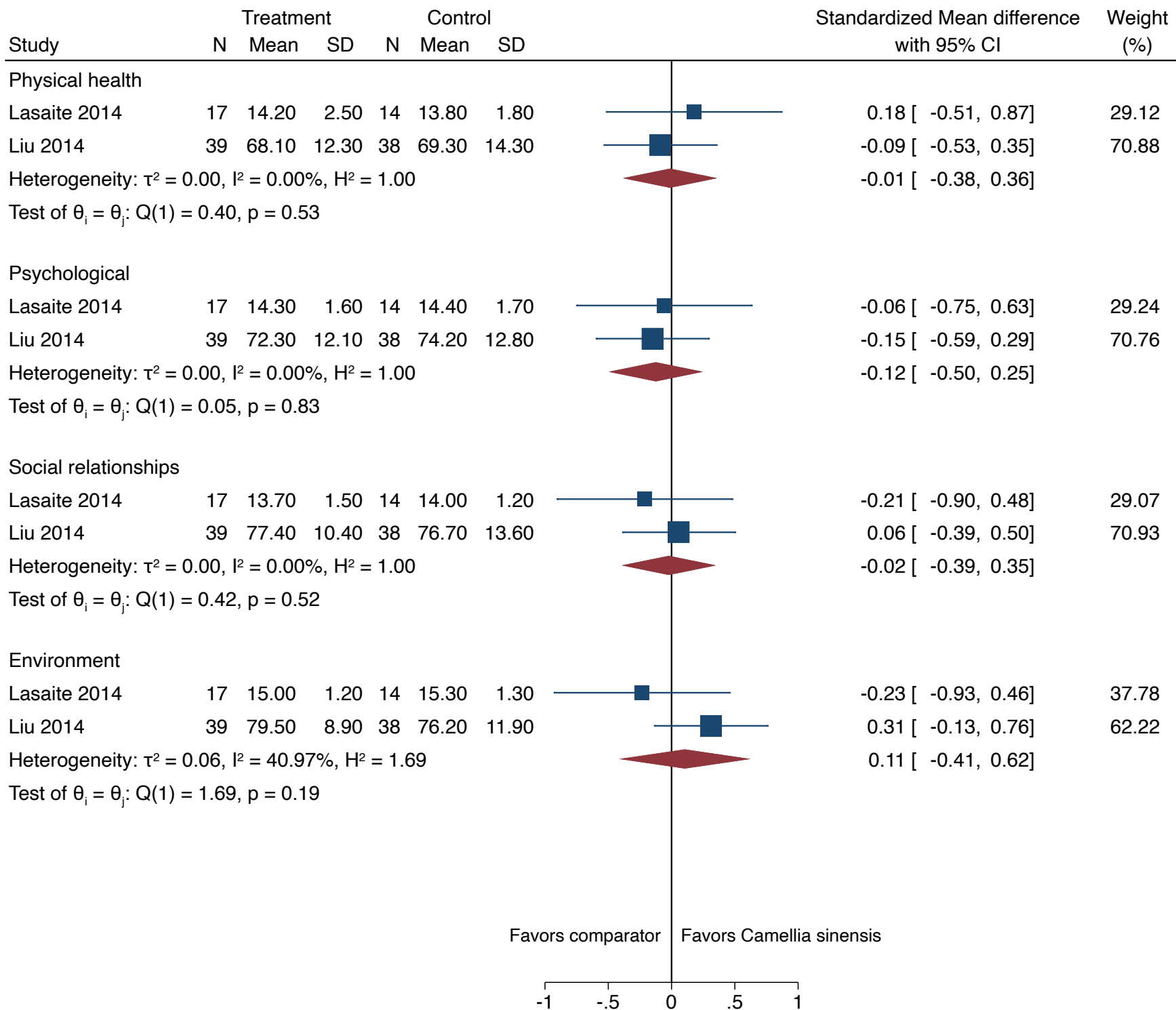

Supplement: Supplementary file 1 [file DataSheet1.zip › Supplementary Material/Forest and Funnel Plots/Camellia sinensis/HRQoL.pdf]

# Camellia sinensis - LDL-C

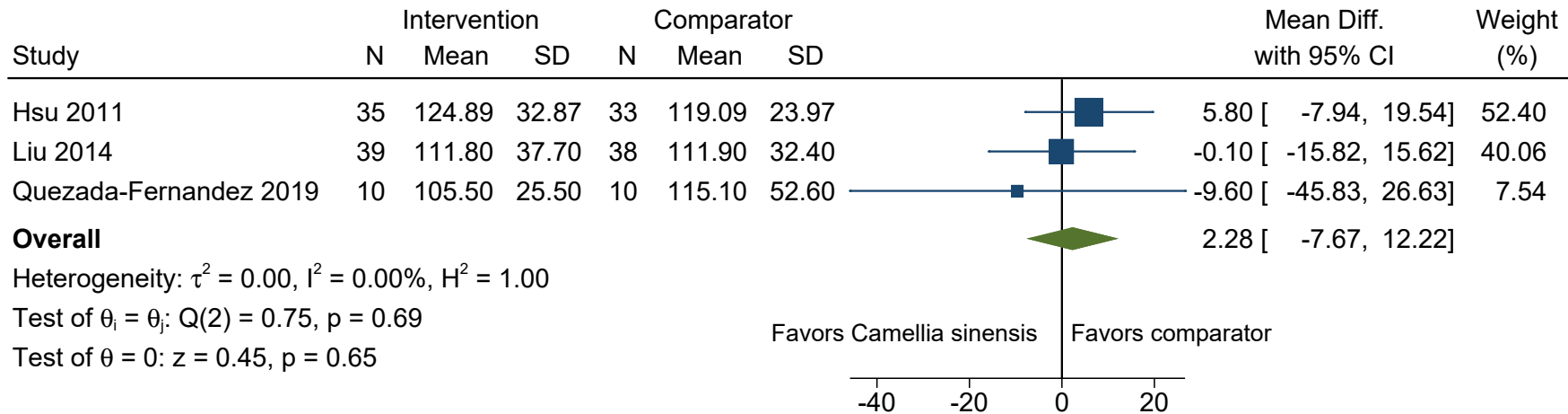

Random-effects REML model

Supplement: Supplementary file 1 [file DataSheet1.zip › Supplementary Material/Forest and Funnel Plots/Camellia sinensis/LDL-C.pdf]

# Aloe vera - TG

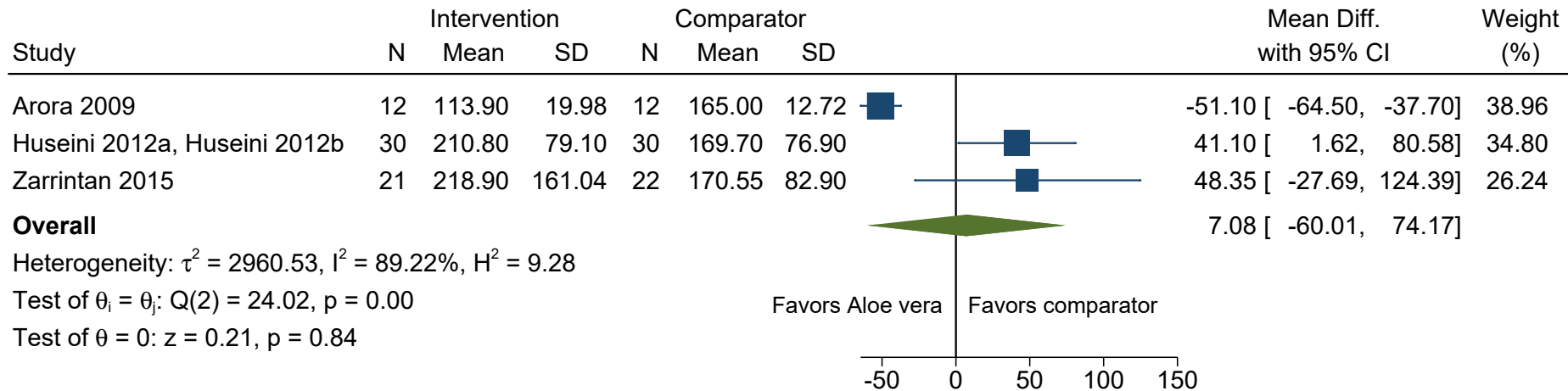

Random-effects REML model

Supplement: Supplementary file 1 [file DataSheet1.zip › Supplementary Material/Forest and Funnel Plots/Aloe vera/TG.pdf]

# Aloe vera - TC

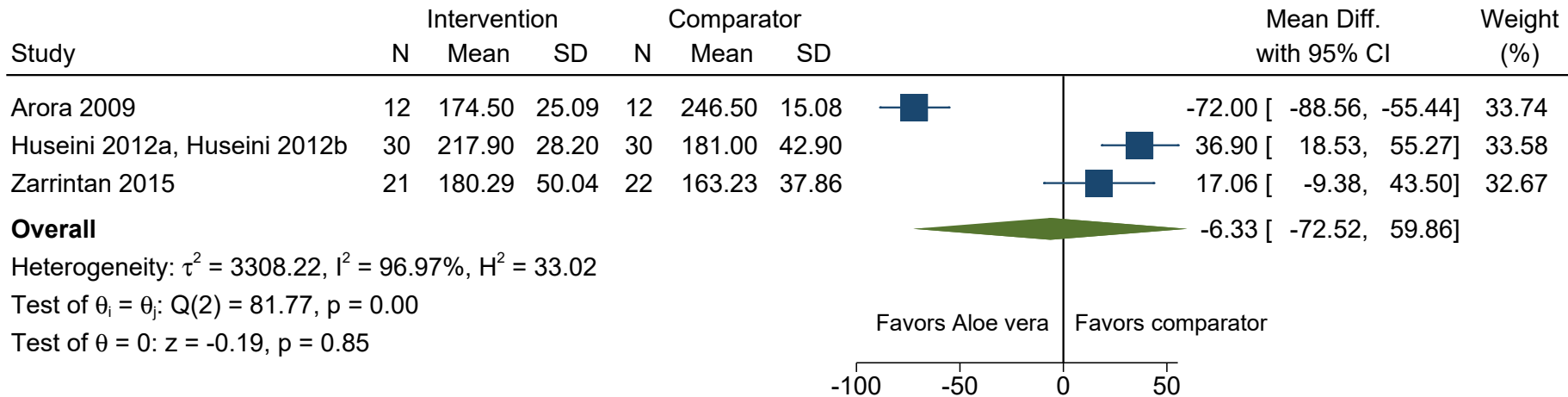

Random-effects REML model

Supplement: Supplementary file 1 [file DataSheet1.zip › Supplementary Material/Forest and Funnel Plots/Aloe vera/TC.pdf]

# Aloe vera - FBG

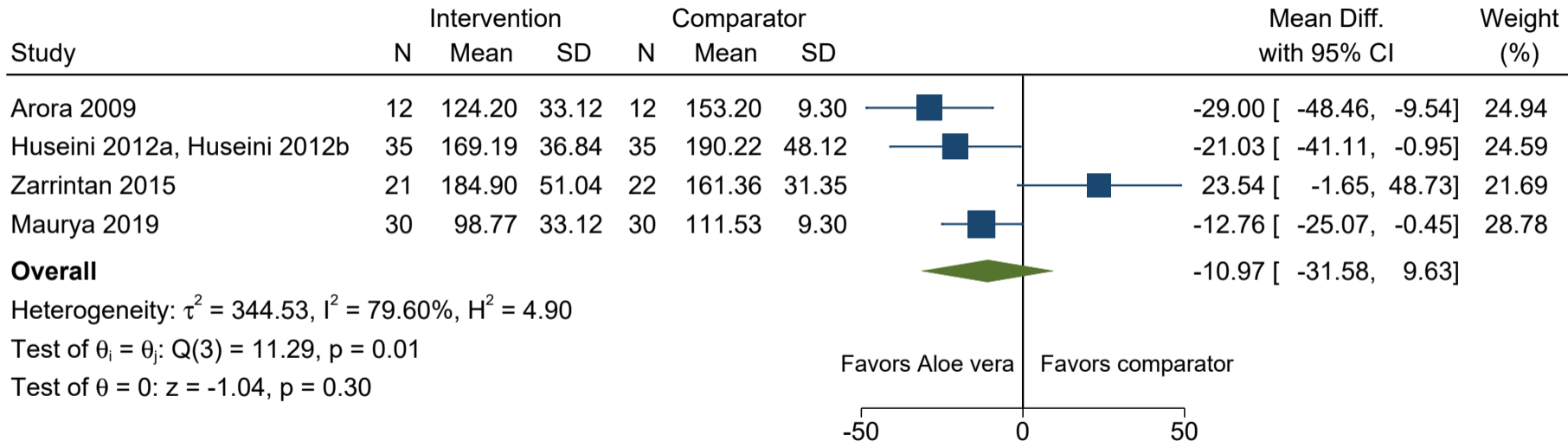

Random-effects REML model

Supplement: Supplementary file 1 [file DataSheet1.zip › Supplementary Material/Forest and Funnel Plots/Aloe vera/FBG.pdf]

# Aloe vera - HDL-C

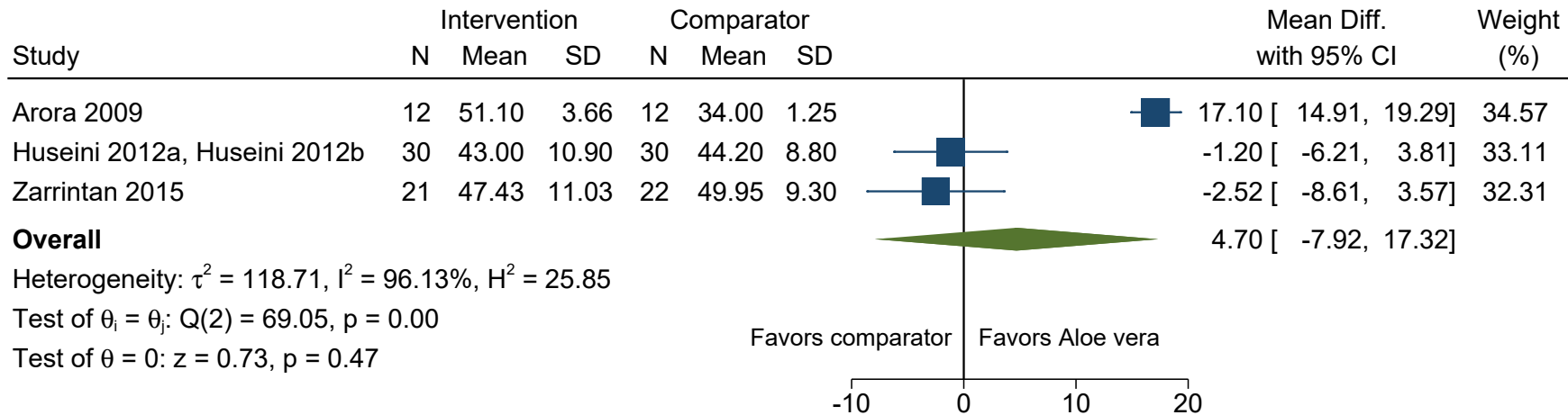

Random-effects REML model

Supplement: Supplementary file 1 [file DataSheet1.zip › Supplementary Material/Forest and Funnel Plots/Aloe vera/HDL-C.pdf]

# Aloe vera - HbA1c

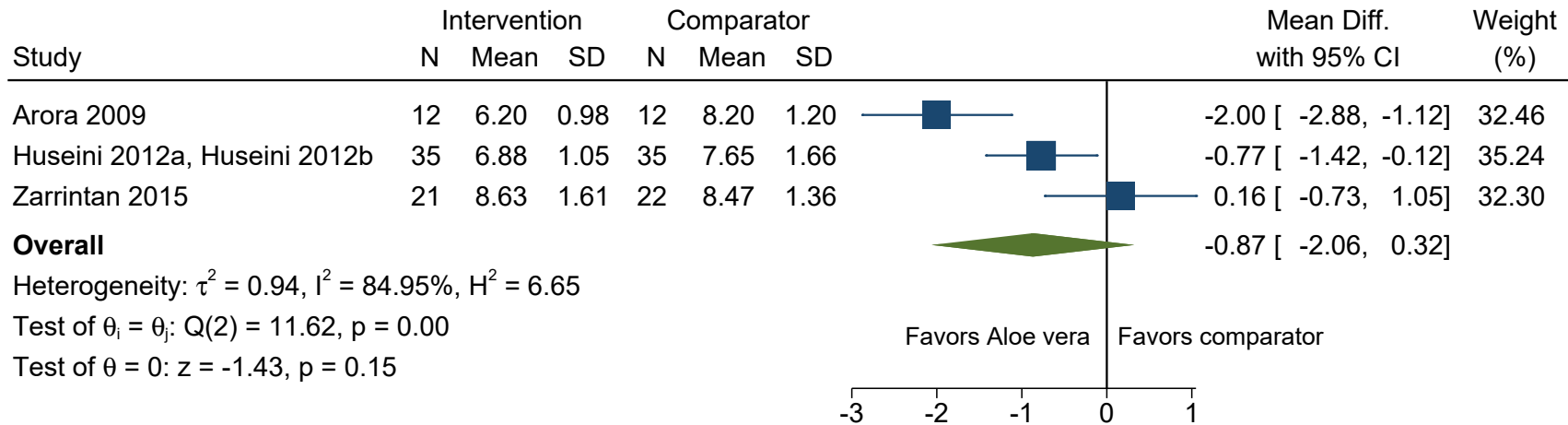

Random-effects REML model

Supplement: Supplementary file 1 [file DataSheet1.zip › Supplementary Material/Forest and Funnel Plots/Aloe vera/HbA1c.pdf]

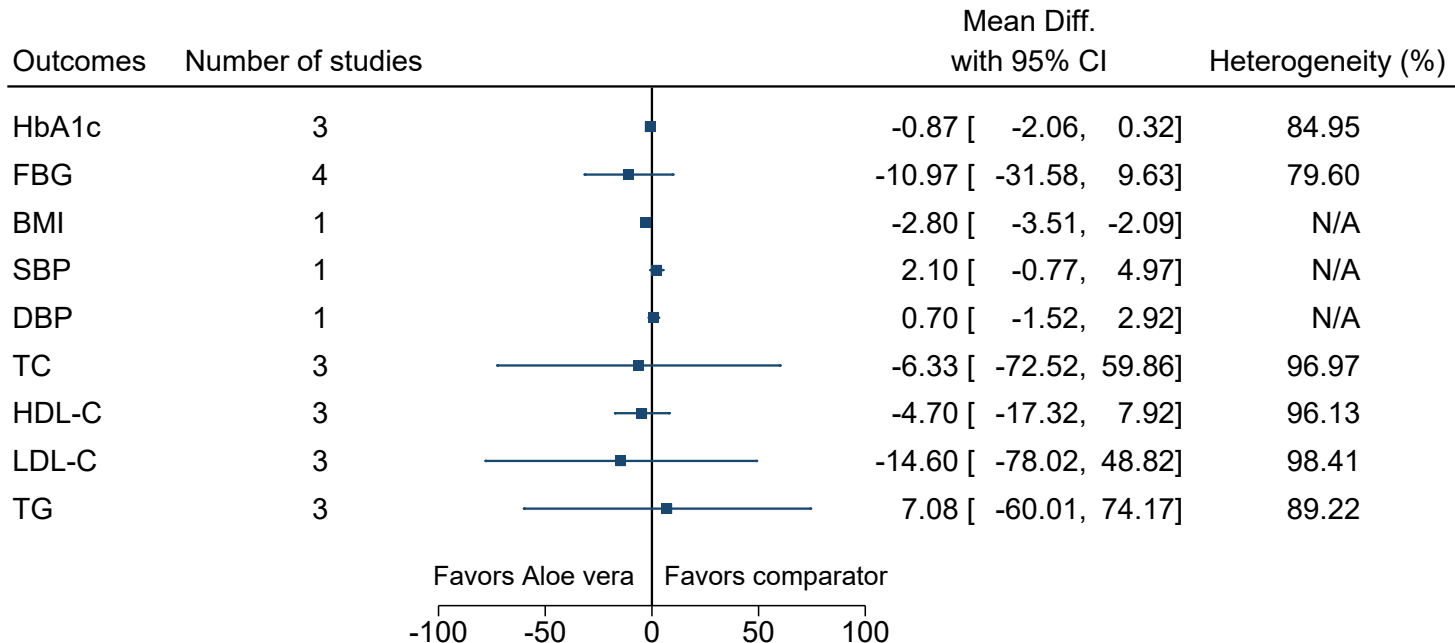

Supplement: Supplementary file 1 [file DataSheet1.zip › Supplementary Material/Forest and Funnel Plots/Aloe vera/Aloe vera.pdf]

# Aloe vera - LDL-C

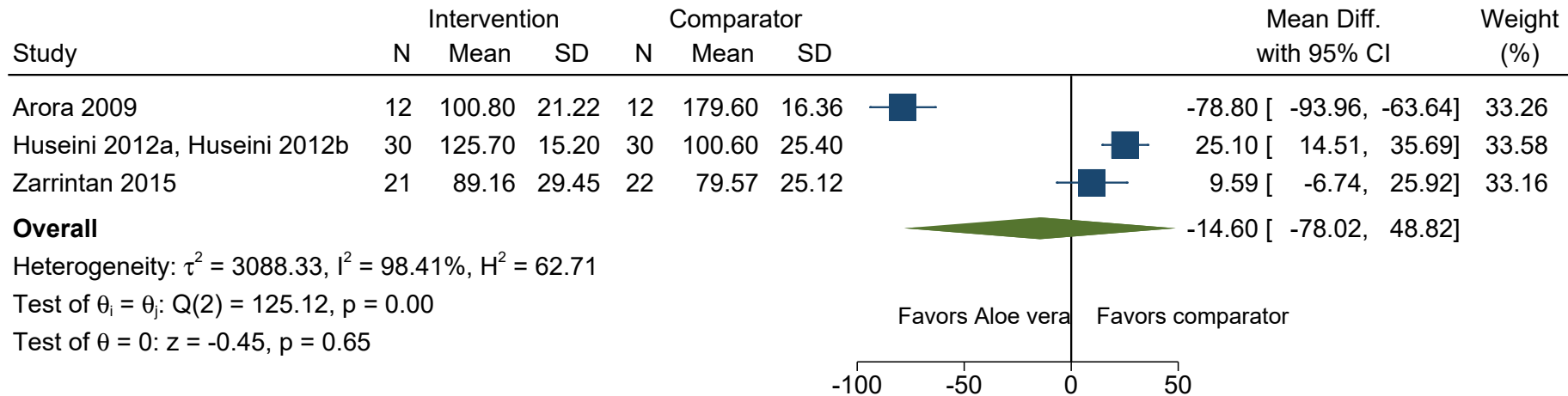

Supplement: Supplementary file 1 [file DataSheet1.zip › Supplementary Material/Forest and Funnel Plots/Aloe vera/LDL-C.pdf]

# Cuminum cyminum - FBG

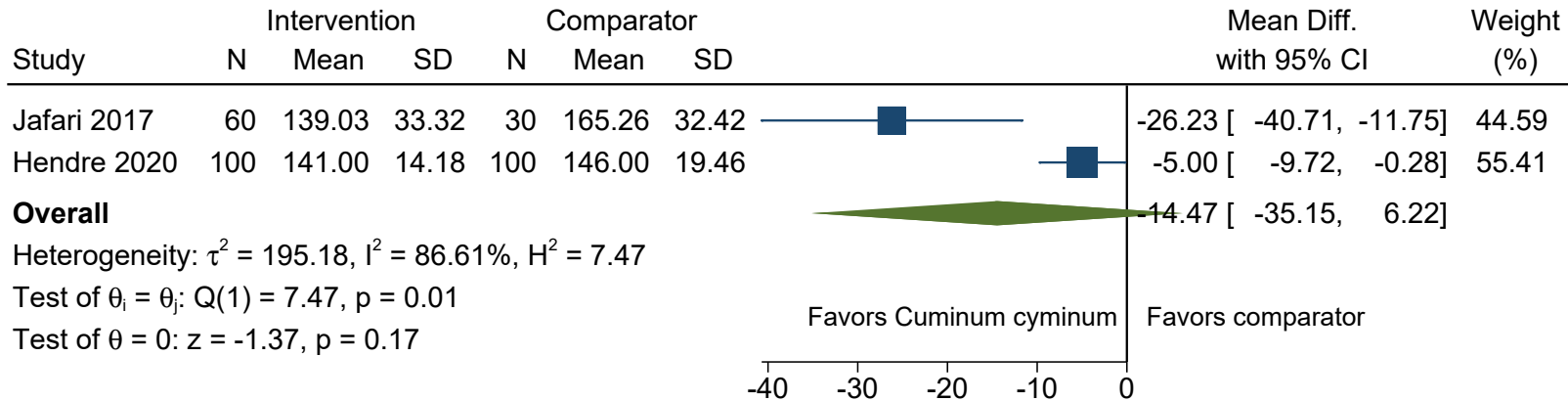

Random-effects REML model

Supplement: Supplementary file 1 [file DataSheet1.zip › Supplementary Material/Forest and Funnel Plots/Cuminum cyminum/FBG.pdf]

# Cuminum cyminum - HbA1c

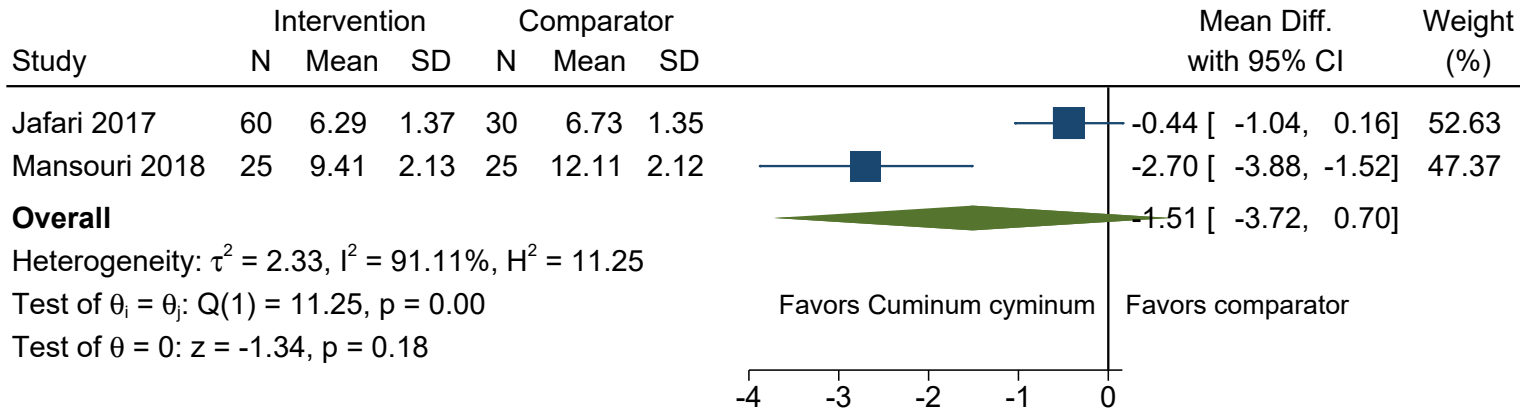

Random-effects REML model

Supplement: Supplementary file 1 [file DataSheet1.zip › Supplementary Material/Forest and Funnel Plots/Cuminum cyminum/HbA1c.pdf]

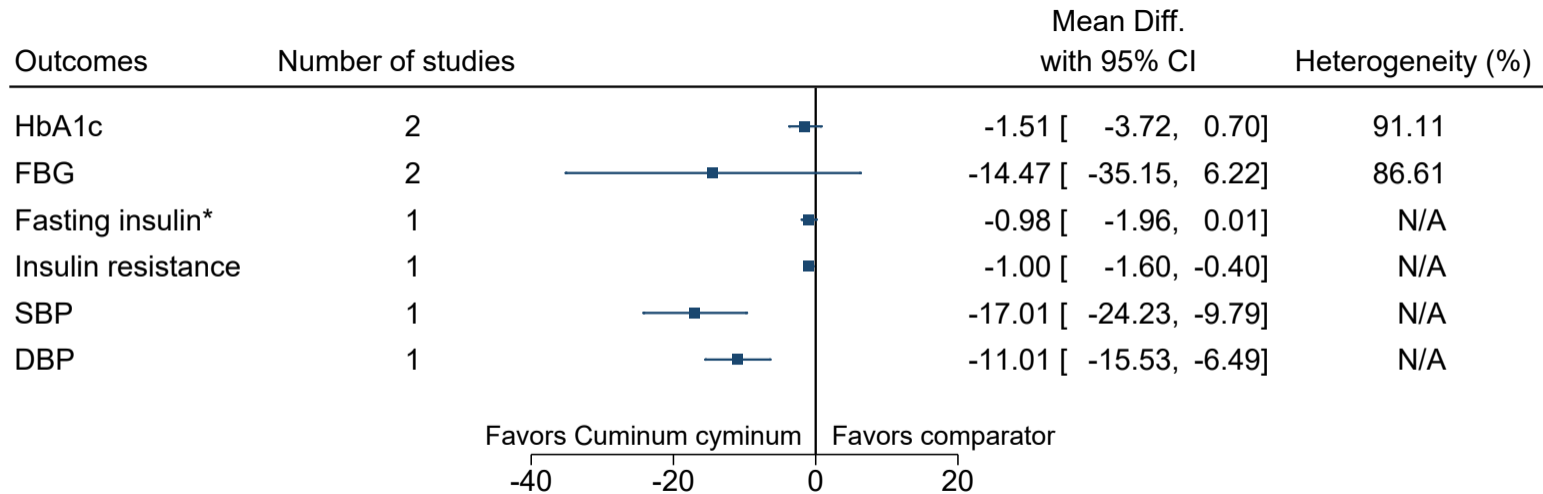

\*Favors intervention/comparator as insulin sensitizer

Supplement: Supplementary file 1 [file DataSheet1.zip › Supplementary Material/Forest and Funnel Plots/Cuminum cyminum/Cuminum cyminum.pdf]

# Aegle marmelos - BMI

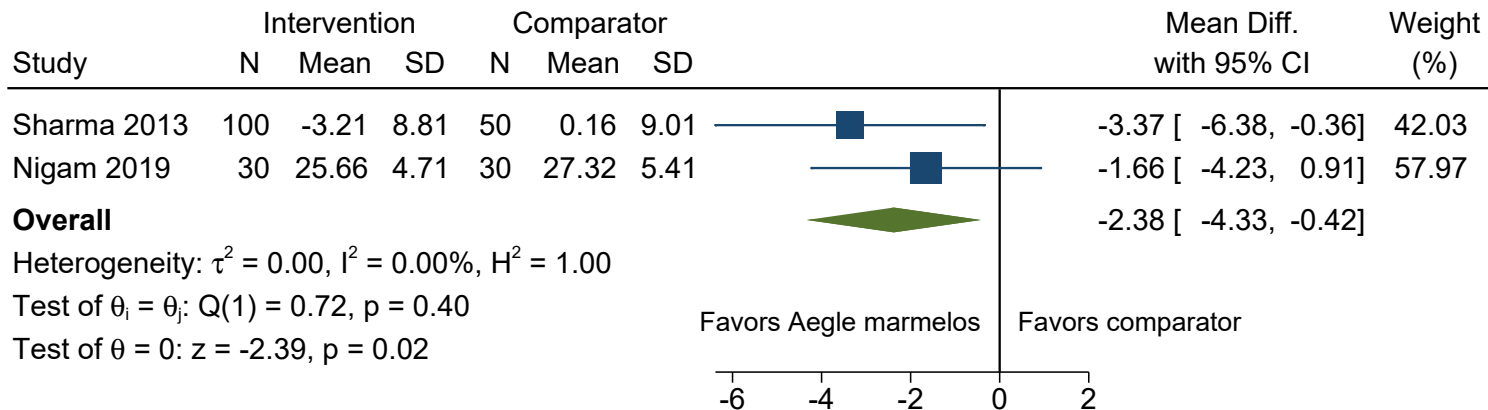

Random-effects REML model

Supplement: Supplementary file 1 [file DataSheet1.zip › Supplementary Material/Forest and Funnel Plots/Aegle marmelos/BMI.pdf]

# Aegle marmelos - FBG

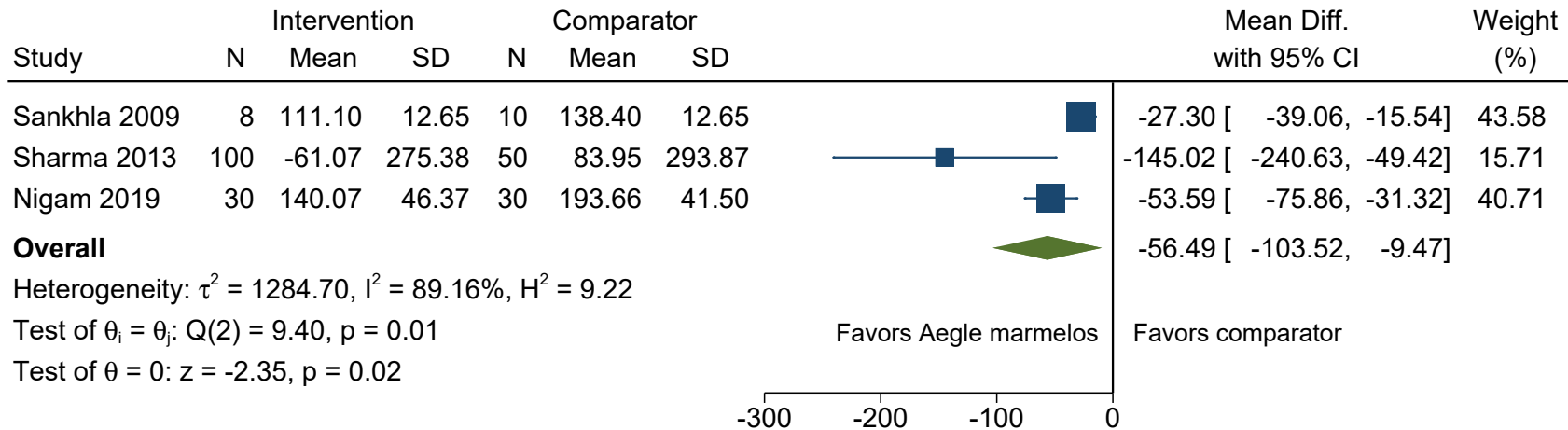

Supplement: Supplementary file 1 [file DataSheet1.zip › Supplementary Material/Forest and Funnel Plots/Aegle marmelos/FBG.pdf]

# Aegle marmelos - DBP

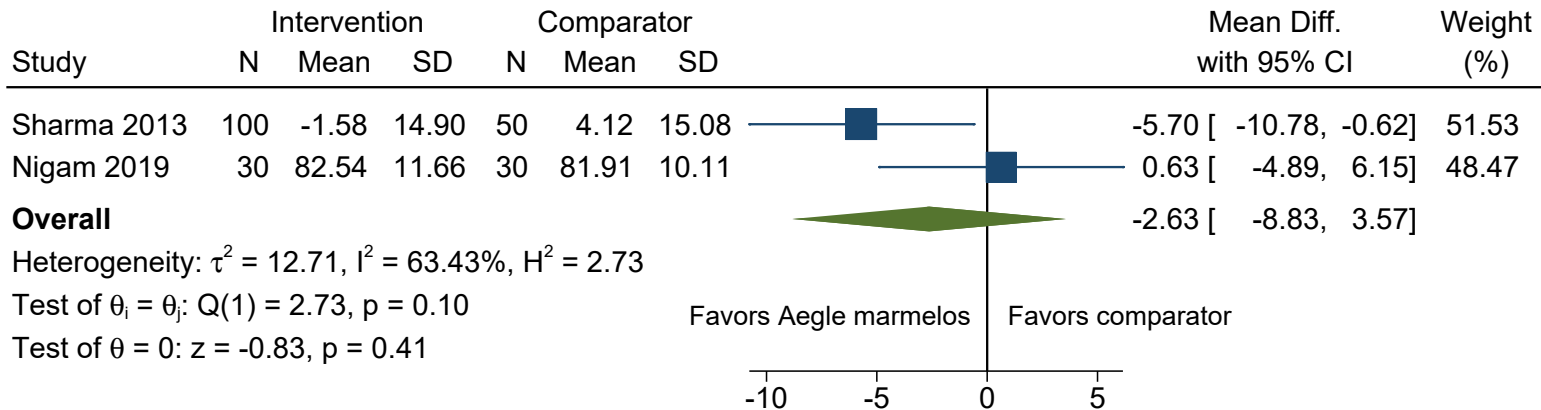

Random-effects REML model

Supplement: Supplementary file 1 [file DataSheet1.zip › Supplementary Material/Forest and Funnel Plots/Aegle marmelos/DBP.pdf]

# Aegle marmelos - HbA1c

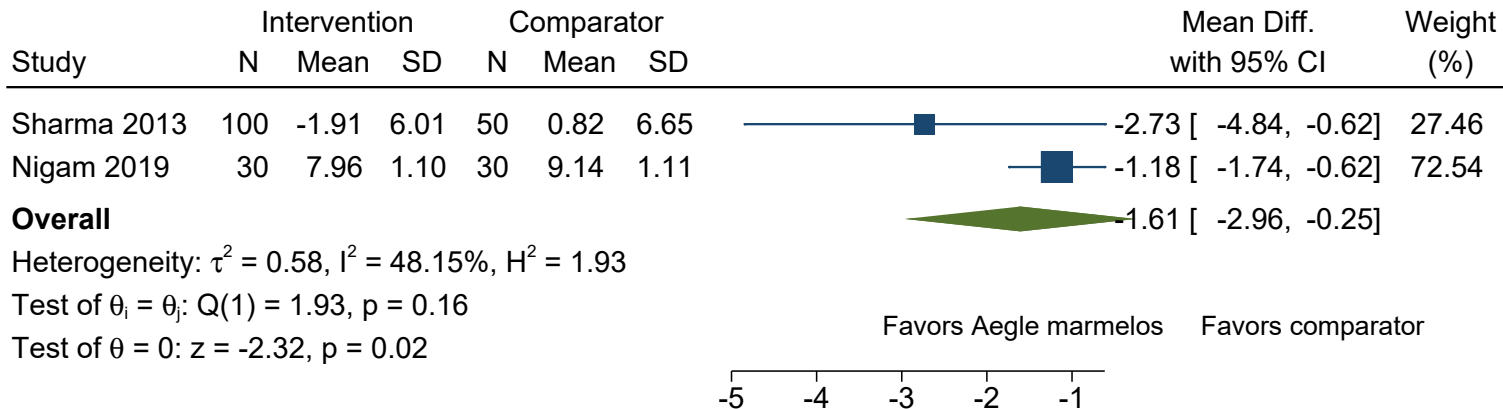

Random-effects REML model

Supplement: Supplementary file 1 [file DataSheet1.zip › Supplementary Material/Forest and Funnel Plots/Aegle marmelos/HbA1c.pdf]

# Aegle marmelos - PPBG

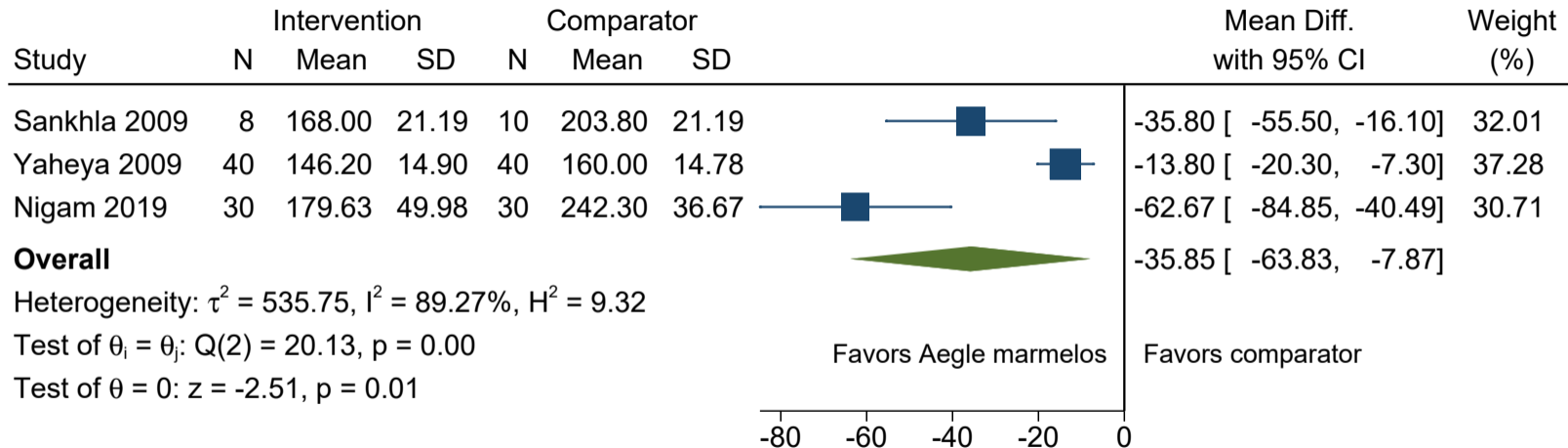

Random-effects REML model

Supplement: Supplementary file 1 [file DataSheet1.zip › Supplementary Material/Forest and Funnel Plots/Aegle marmelos/PPBG.pdf]

# Aegle marmelos - SBP

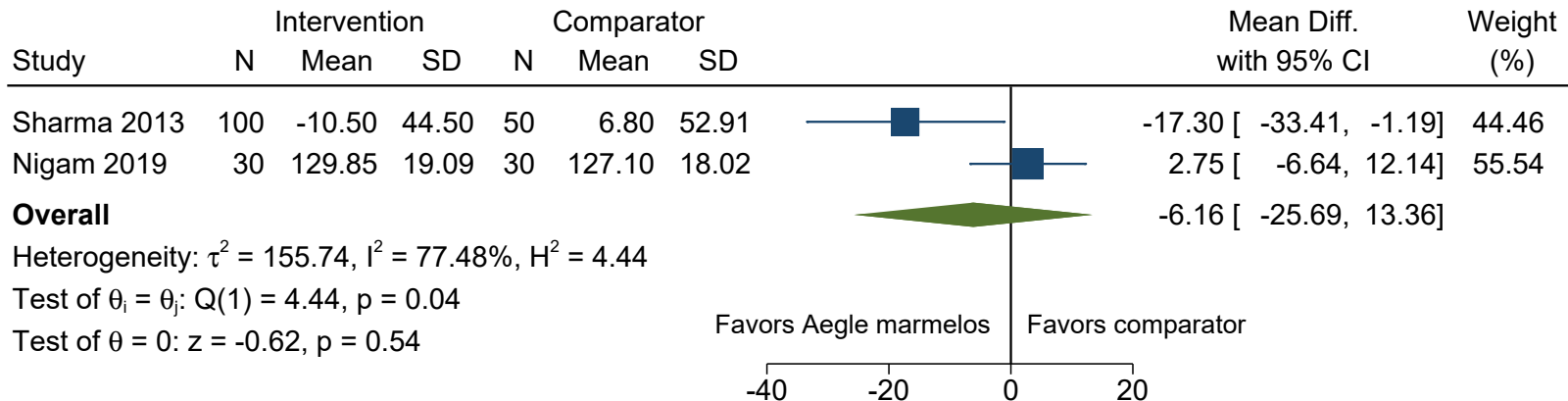

Random-effects REML model

Supplement: Supplementary file 1 [file DataSheet1.zip › Supplementary Material/Forest and Funnel Plots/Aegle marmelos/SBP.pdf]

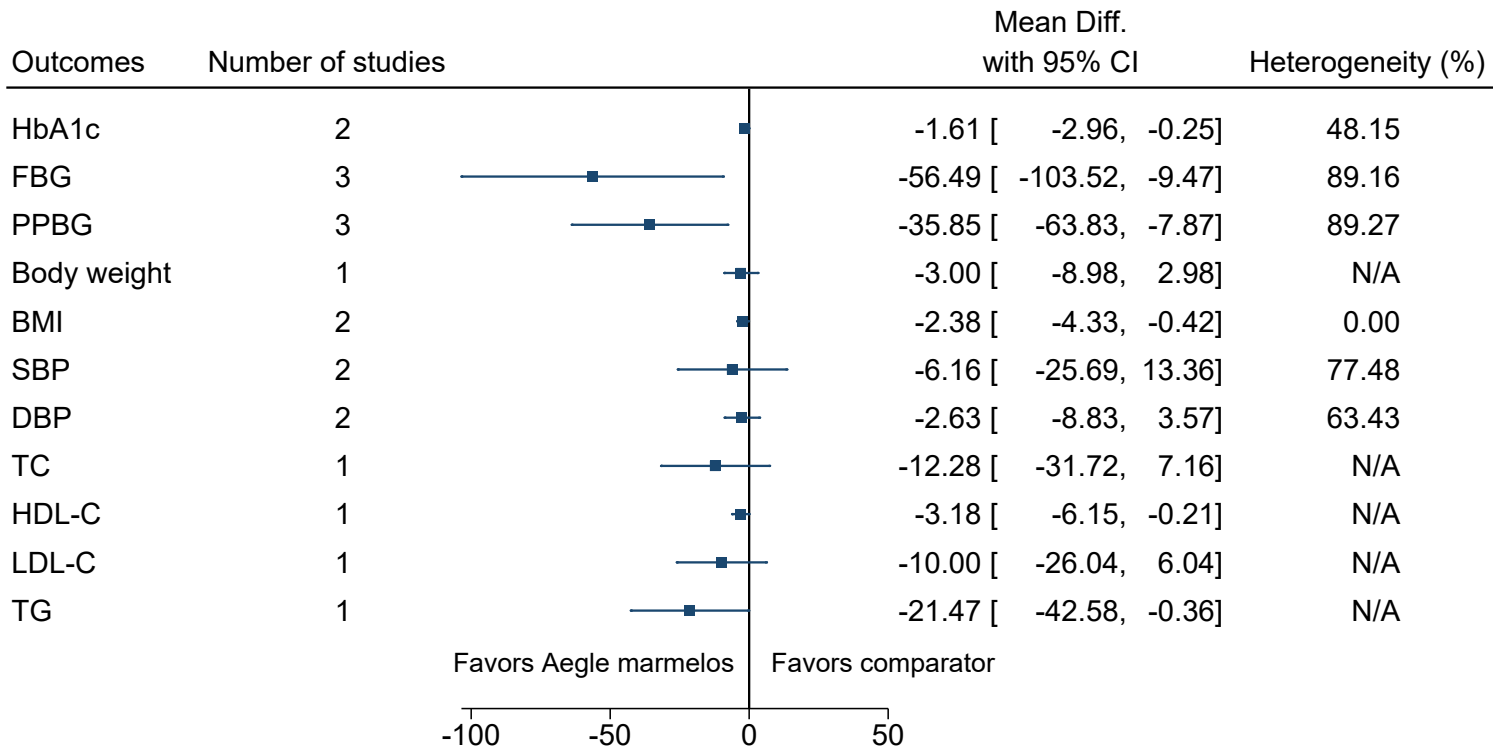

Supplement: Supplementary file 1 [file DataSheet1.zip › Supplementary Material/Forest and Funnel Plots/Aegle marmelos/Aegle marmelos.pdf]

# Coccinia grandis - Waist circumference

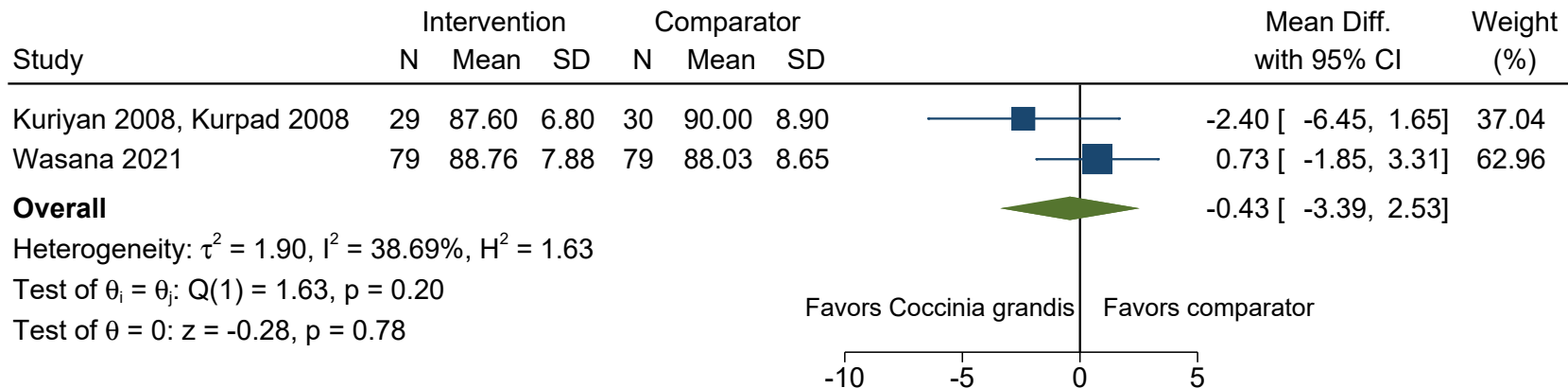

Random-effects REML model

Supplement: Supplementary file 1 [file DataSheet1.zip › Supplementary Material/Forest and Funnel Plots/Coccinia grandis/Waist circumference.pdf]

# Coccinia grandis - TG

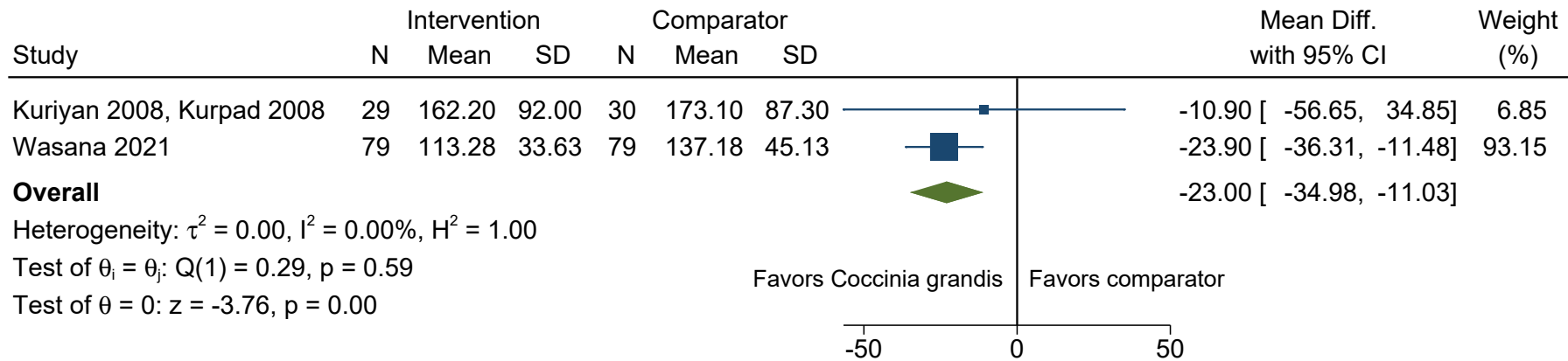

Random-effects REML model

Supplement: Supplementary file 1 [file DataSheet1.zip › Supplementary Material/Forest and Funnel Plots/Coccinia grandis/TG.pdf]

# Coccinia grandis - BMI

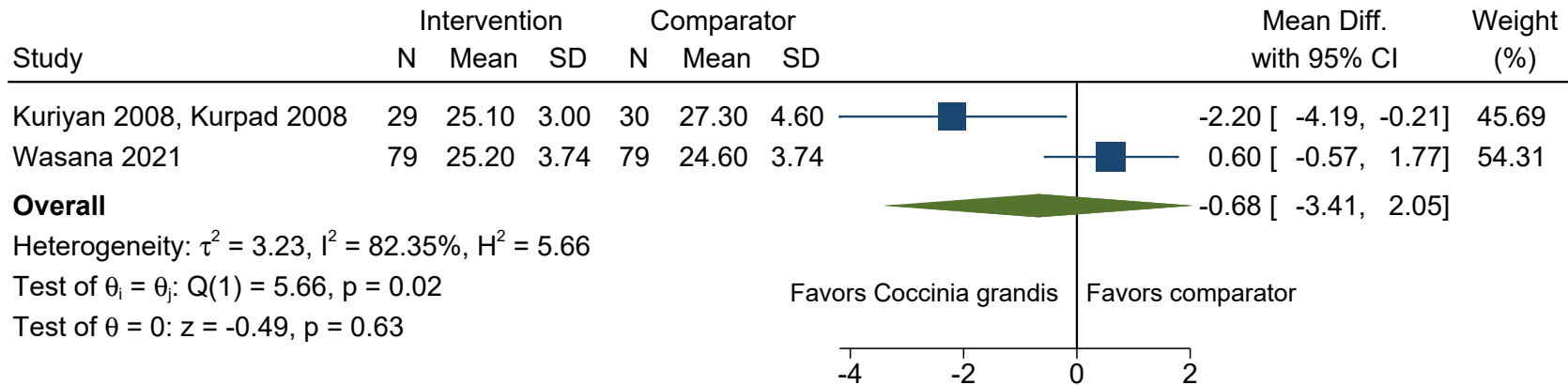

Random-effects REML model

Supplement: Supplementary file 1 [file DataSheet1.zip › Supplementary Material/Forest and Funnel Plots/Coccinia grandis/BMI.pdf]

# Coccinia grandis - TC

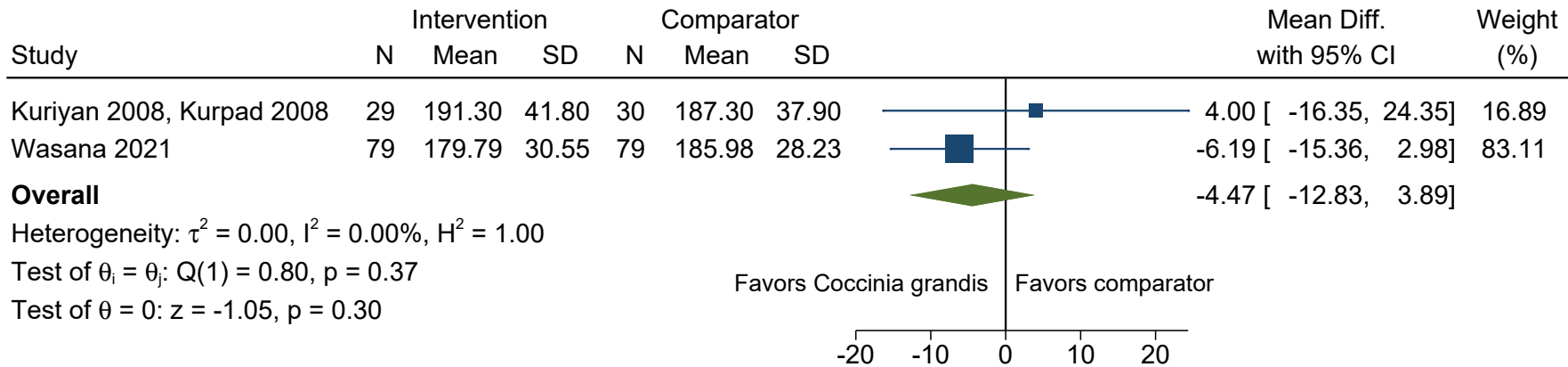

Random-effects REML model

Supplement: Supplementary file 1 [file DataSheet1.zip › Supplementary Material/Forest and Funnel Plots/Coccinia grandis/TC.pdf]

# Coccinia grandis - FBG

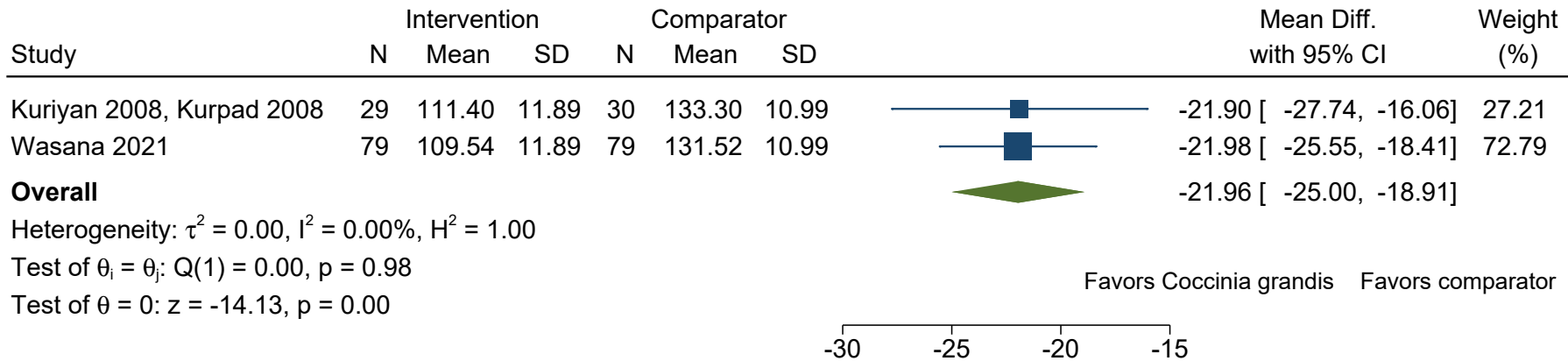

Random-effects REML model

Supplement: Supplementary file 1 [file DataSheet1.zip › Supplementary Material/Forest and Funnel Plots/Coccinia grandis/FBG.pdf]

# Coccinia grandis - HDL-C

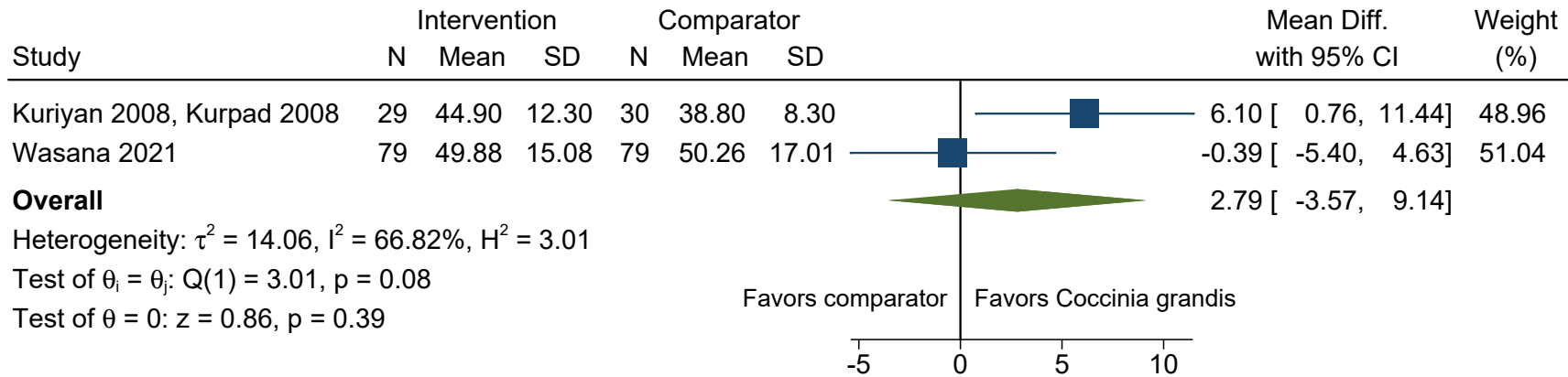

Random-effects REML model

Supplement: Supplementary file 1 [file DataSheet1.zip › Supplementary Material/Forest and Funnel Plots/Coccinia grandis/HDL-C.pdf]

# Coccinia grandis - HbA1c

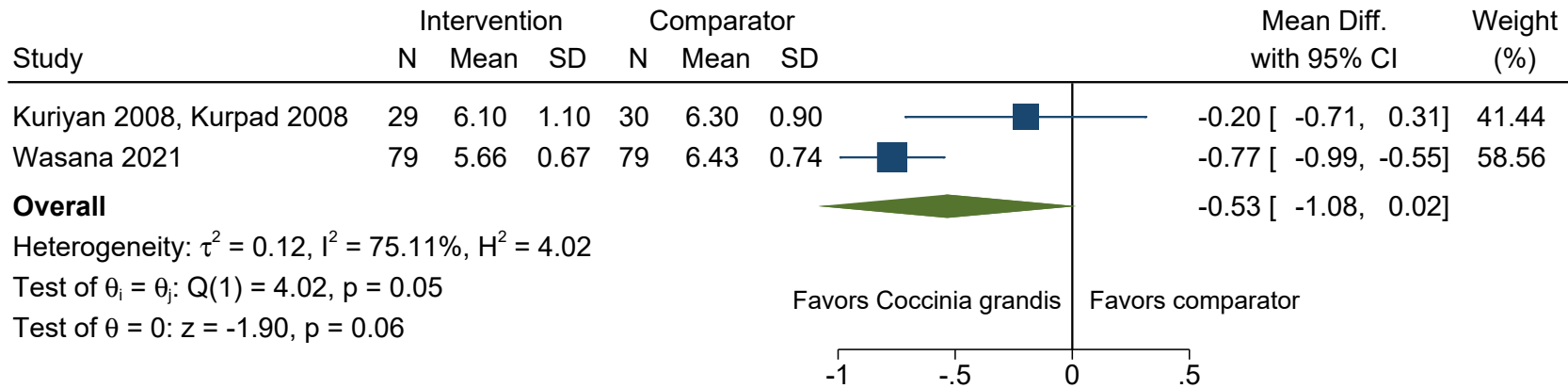

Random-effects REML model

Supplement: Supplementary file 1 [file DataSheet1.zip › Supplementary Material/Forest and Funnel Plots/Coccinia grandis/HbA1c.pdf]

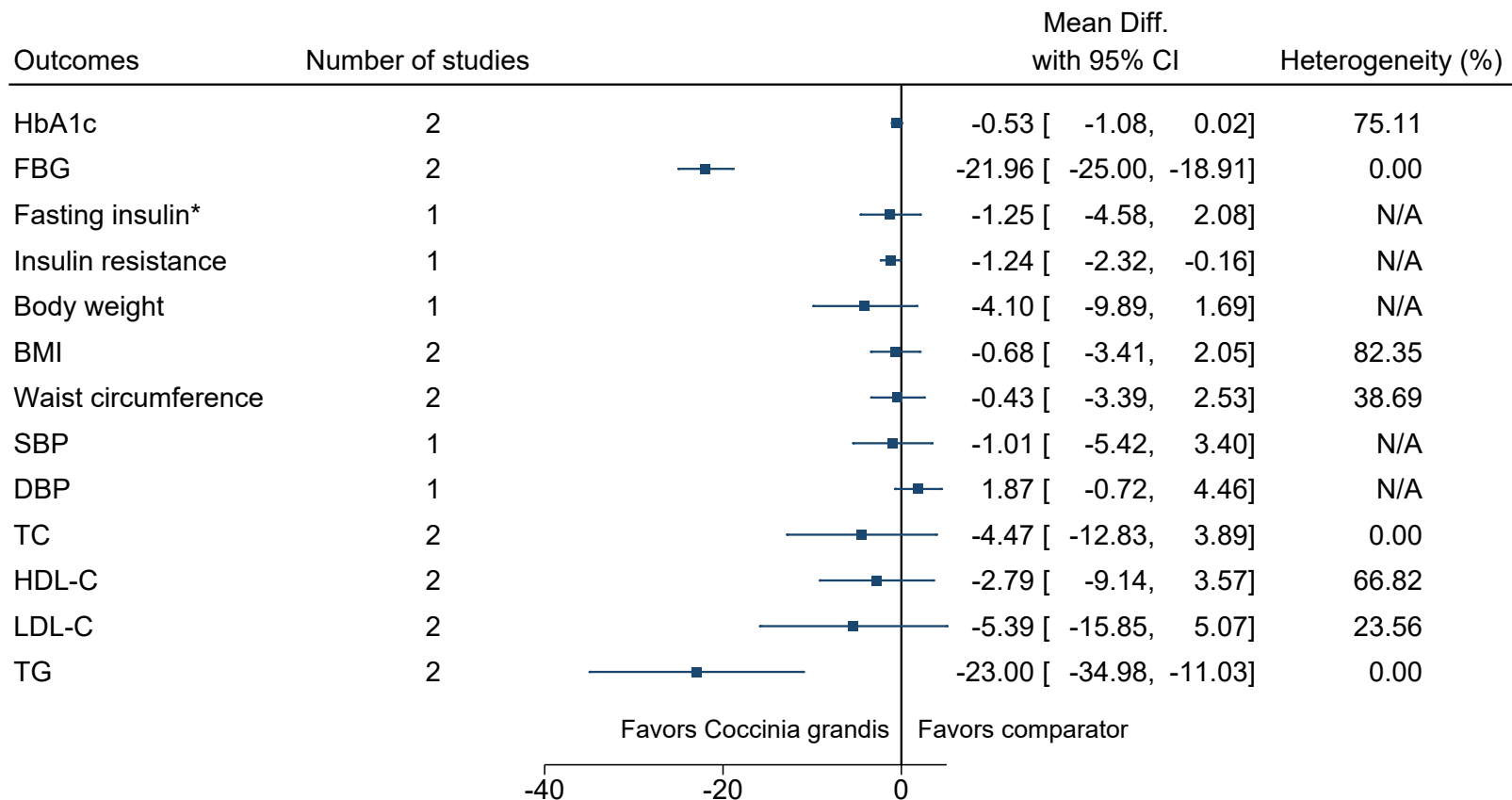

\*Favors intervention/comparator as insulin sensitizer

Supplement: Supplementary file 1 [file DataSheet1.zip › Supplementary Material/Forest and Funnel Plots/Coccinia grandis/Coccinia grandis.pdf]

# Coccinia grandis - LDL-C

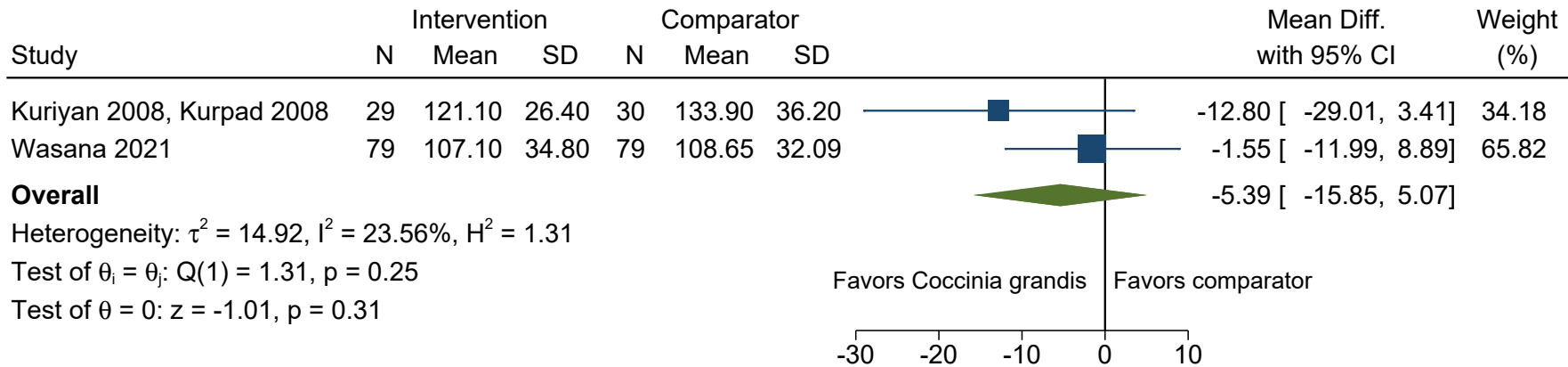

Random-effects REML model

Supplement: Supplementary file 1 [file DataSheet1.zip › Supplementary Material/Forest and Funnel Plots/Coccinia grandis/LDL-C.pdf]

# Ipomoea batatas -TG

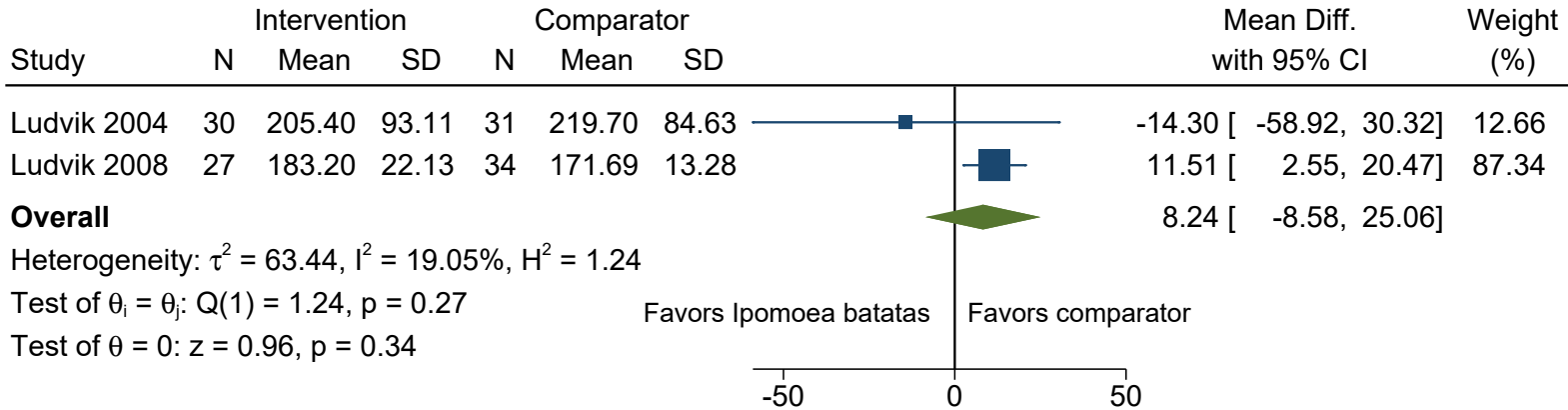

Random-effects REML model

Supplement: Supplementary file 1 [file DataSheet1.zip › Supplementary Material/Forest and Funnel Plots/Ipomoea batatas/TG.pdf]

# Ipomoea batatas -TC

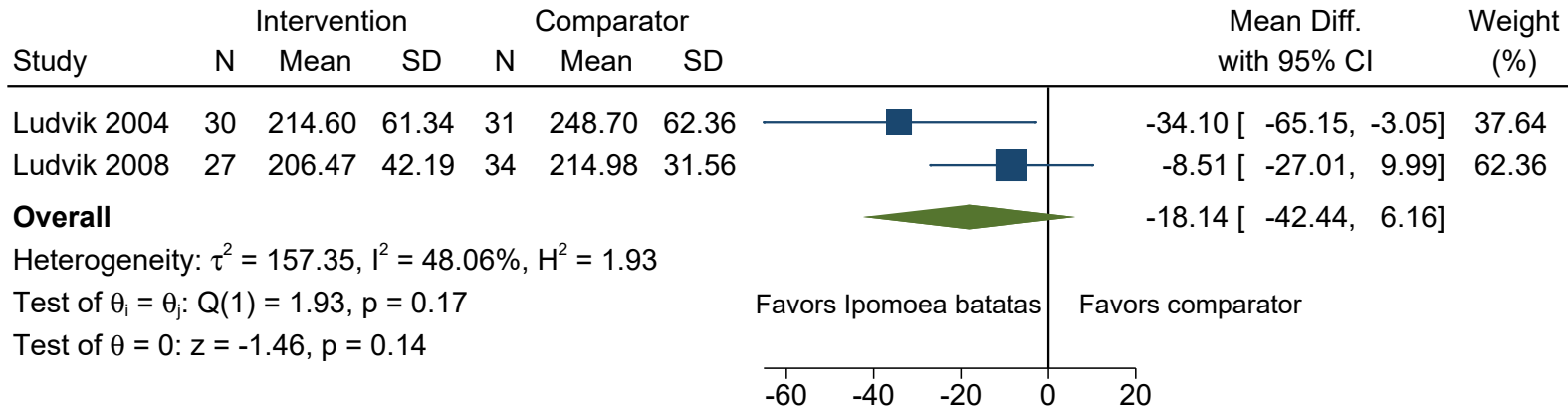

Random-effects REML model

Supplement: Supplementary file 1 [file DataSheet1.zip › Supplementary Material/Forest and Funnel Plots/Ipomoea batatas/TC.pdf]

# Ipomoea batatas - FBG

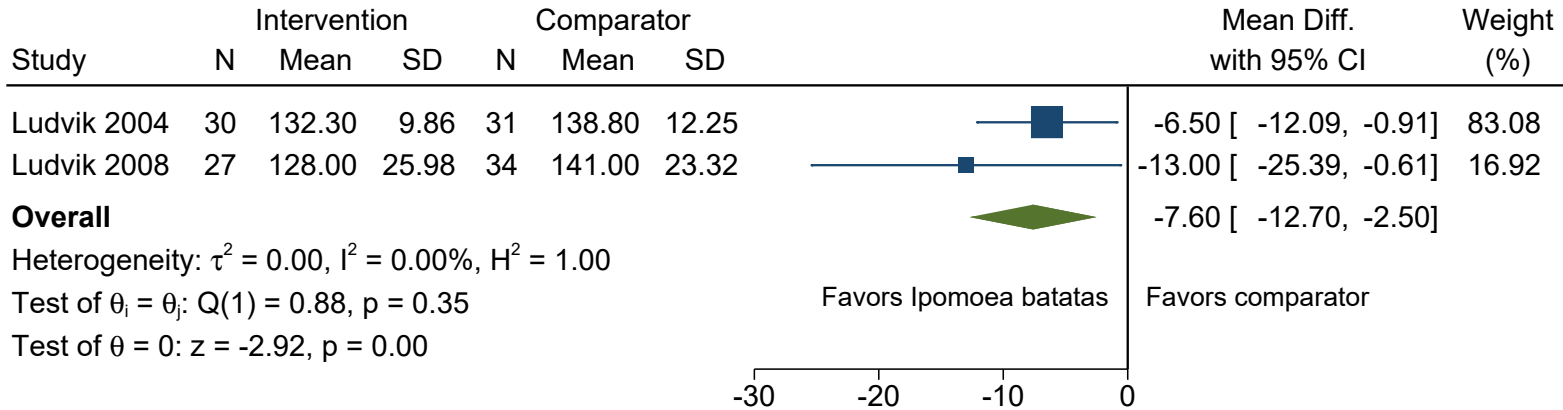

Random-effects REML model

Supplement: Supplementary file 1 [file DataSheet1.zip › Supplementary Material/Forest and Funnel Plots/Ipomoea batatas/FBG.pdf]

# Ipomoea batatas - HbA1c

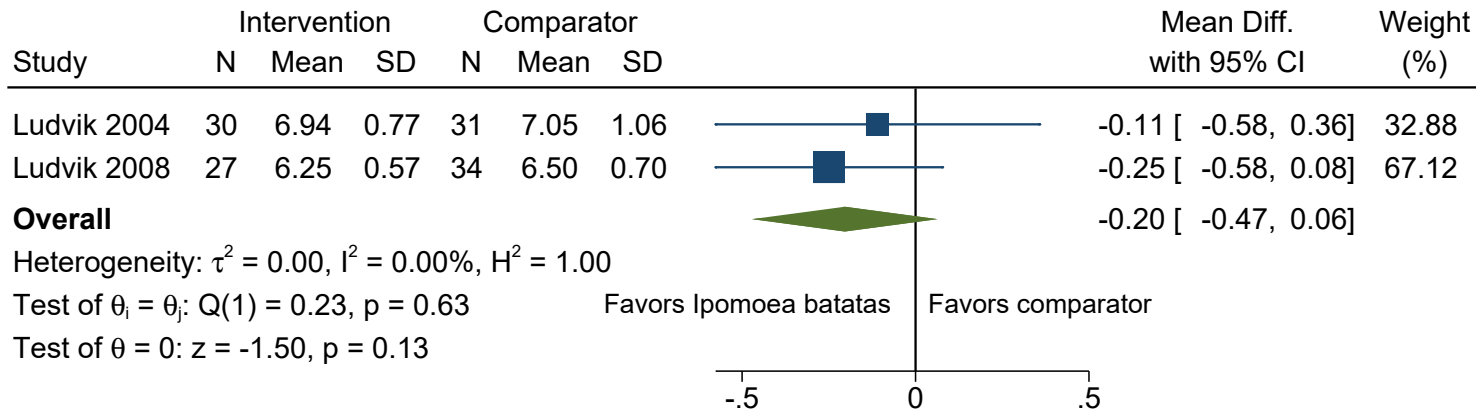

Random-effects REML model

Supplement: Supplementary file 1 [file DataSheet1.zip › Supplementary Material/Forest and Funnel Plots/Ipomoea batatas/HbA1c.pdf]

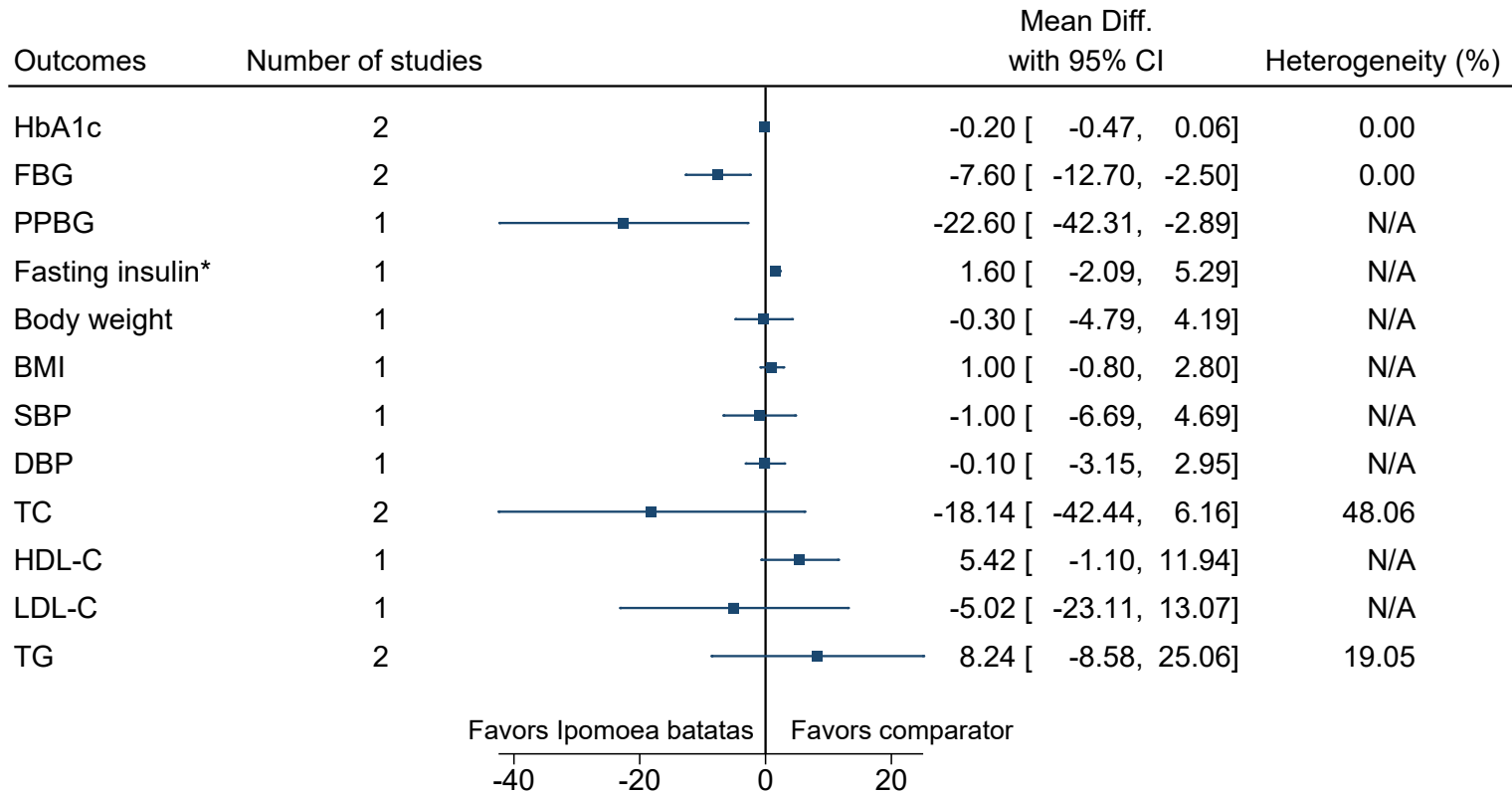

\*Favors intervention/comparator as insulin sensitizer

Supplement: Supplementary file 1 [file DataSheet1.zip › Supplementary Material/Forest and Funnel Plots/Ipomoea batatas/Ipomoea batatas.pdf]

# Urtica dioica - TG

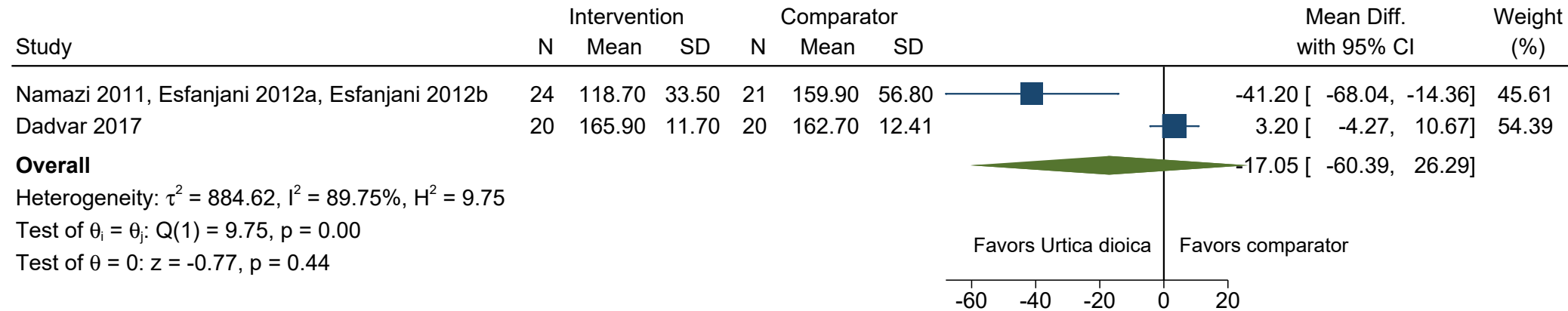

Random-effects REML model

Supplement: Supplementary file 1 [file DataSheet1.zip › Supplementary Material/Forest and Funnel Plots/Urtica dioica/TG.pdf]

# Urtica dioica - BMI

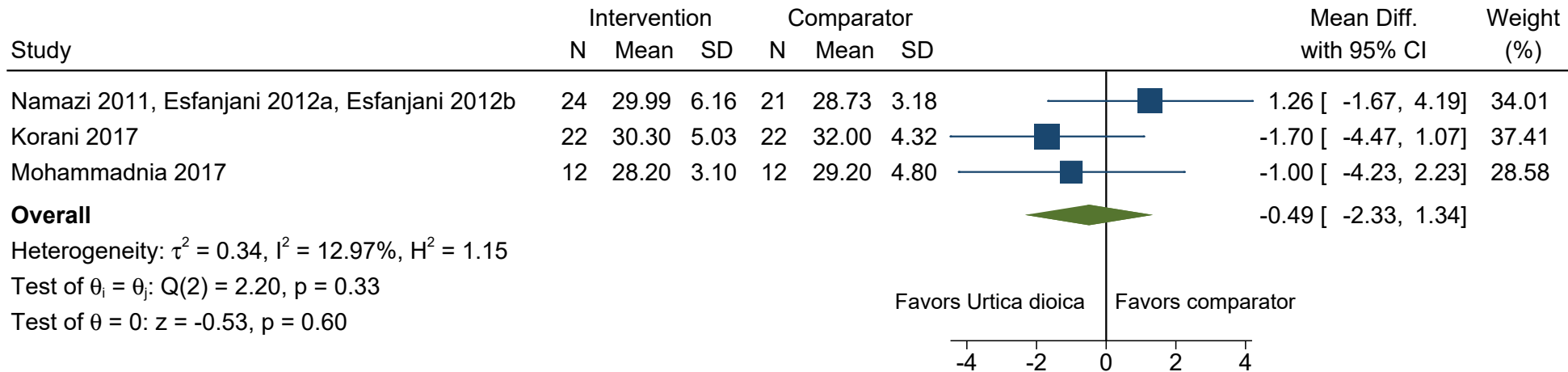

Random-effects REML model

Supplement: Supplementary file 1 [file DataSheet1.zip › Supplementary Material/Forest and Funnel Plots/Urtica dioica/BMI.pdf]

# Urtica dioica - TC

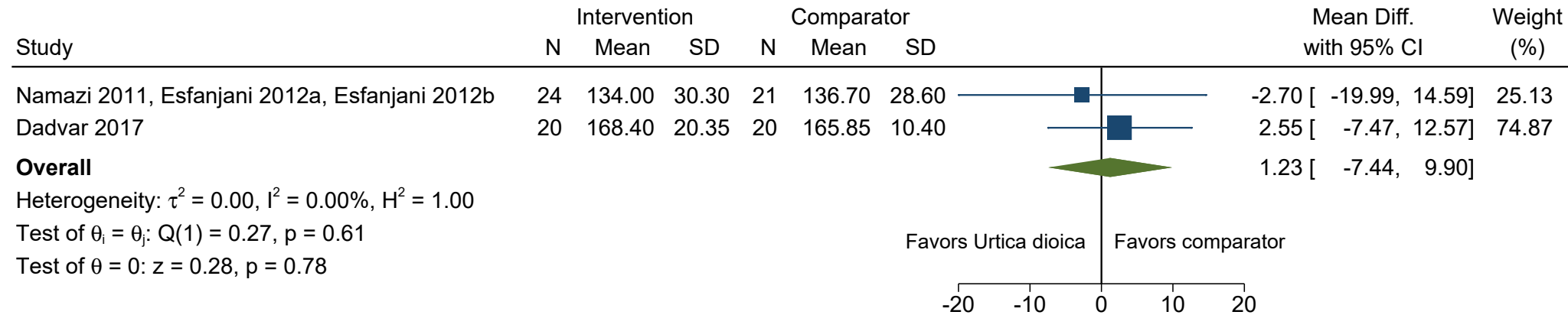

Random-effects REML model

Supplement: Supplementary file 1 [file DataSheet1.zip › Supplementary Material/Forest and Funnel Plots/Urtica dioica/TC.pdf]

# Urtica dioica - FBG

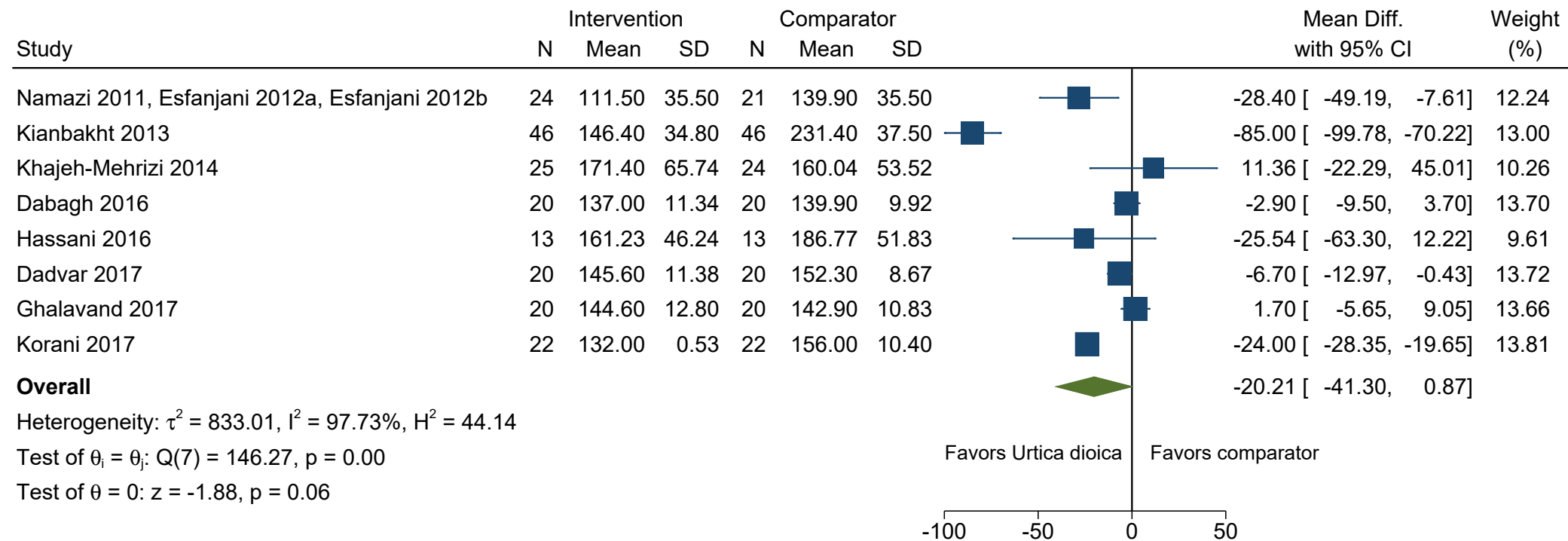

Supplement: Supplementary file 1 [file DataSheet1.zip › Supplementary Material/Forest and Funnel Plots/Urtica dioica/FBG.pdf]

# Urtica dioica - HDL-C

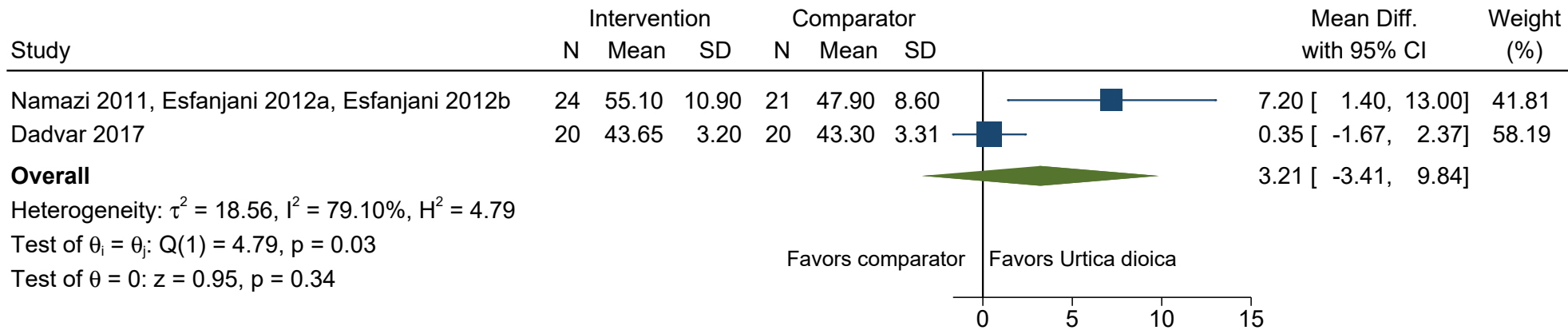

Random-effects REML model

Supplement: Supplementary file 1 [file DataSheet1.zip › Supplementary Material/Forest and Funnel Plots/Urtica dioica/HDL-C.pdf]

# Urtica dioica - DBP

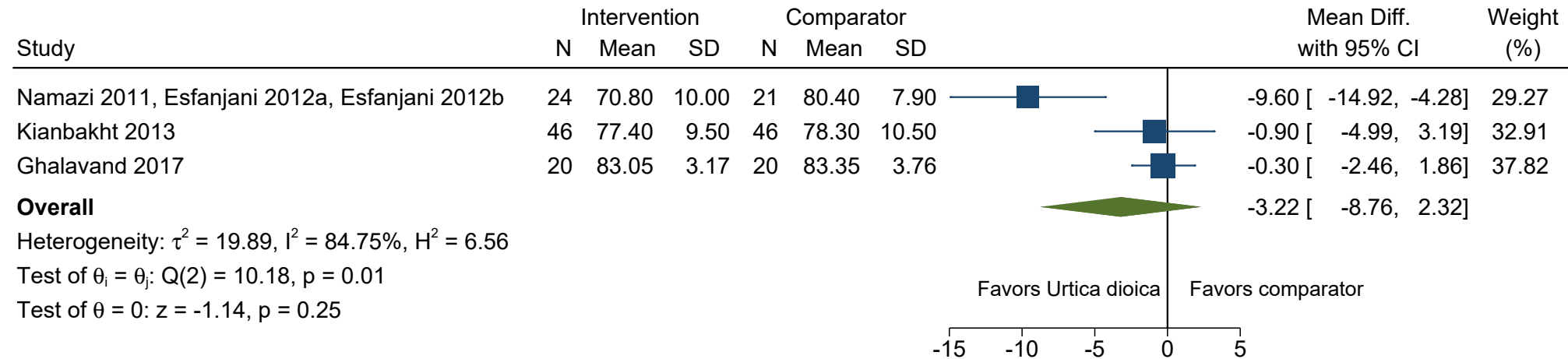

Supplement: Supplementary file 1 [file DataSheet1.zip › Supplementary Material/Forest and Funnel Plots/Urtica dioica/DBP.pdf]

# Urtica dioica - Insulin resistance

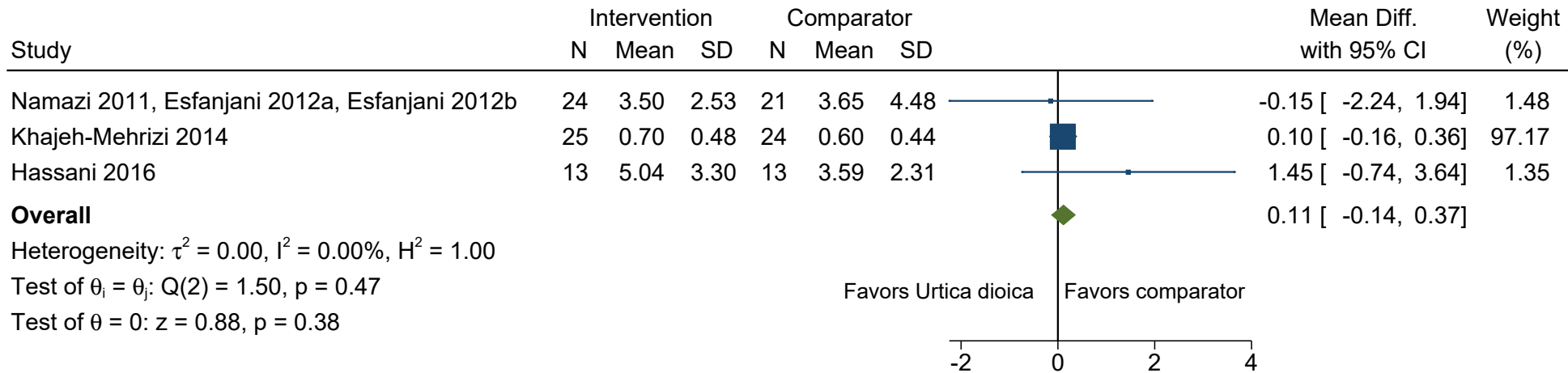

Random-effects REML model

Supplement: Supplementary file 1 [file DataSheet1.zip › Supplementary Material/Forest and Funnel Plots/Urtica dioica/Insulin resistance.pdf]

# Urtica dioica - Fasting insulin

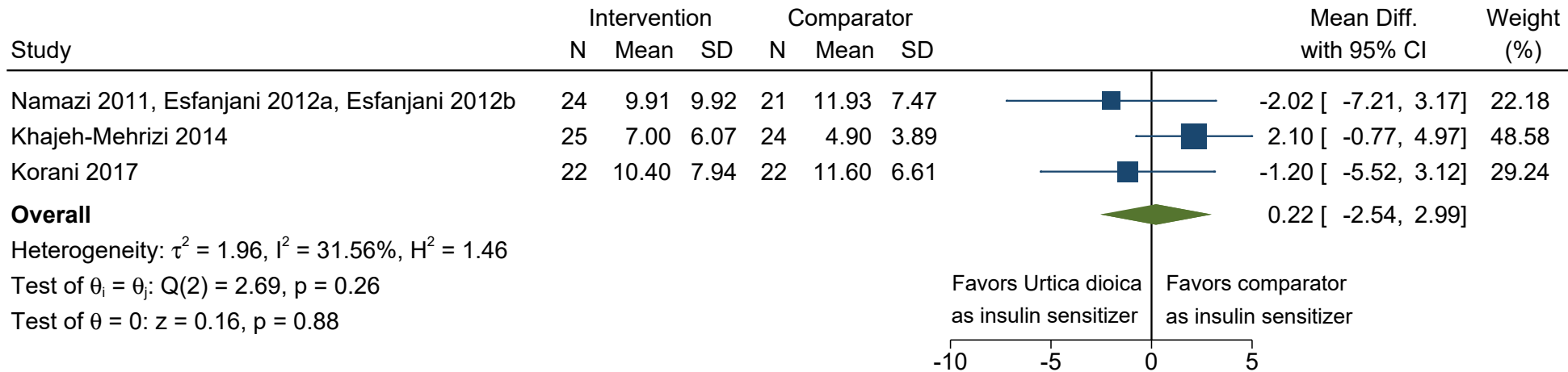

Random-effects REML model

Supplement: Supplementary file 1 [file DataSheet1.zip › Supplementary Material/Forest and Funnel Plots/Urtica dioica/Fasting insulin.pdf]

# Urtica dioica - HbA1c

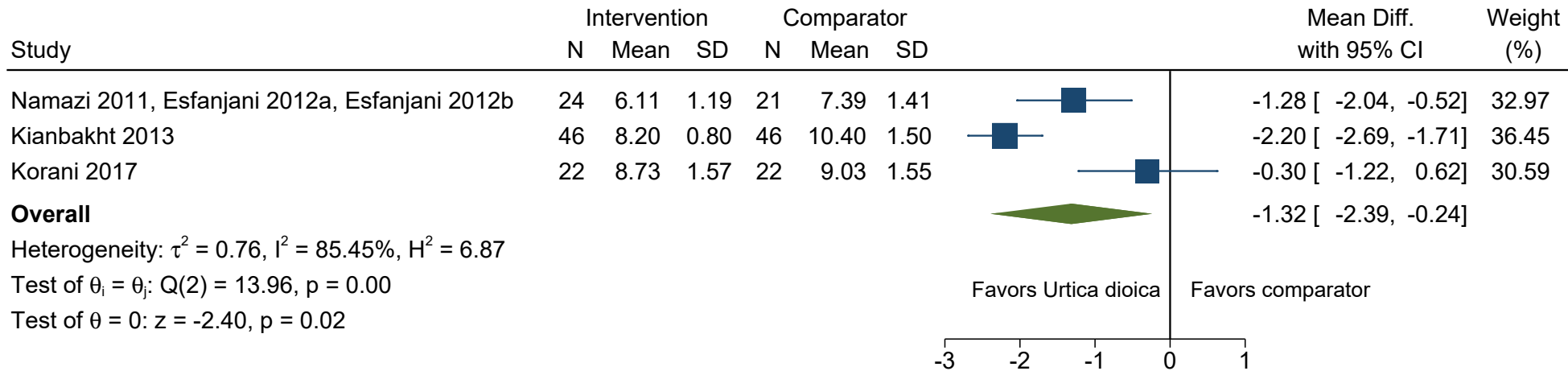

Supplement: Supplementary file 1 [file DataSheet1.zip › Supplementary Material/Forest and Funnel Plots/Urtica dioica/HbA1c.pdf]

Urtica dioica - SBP

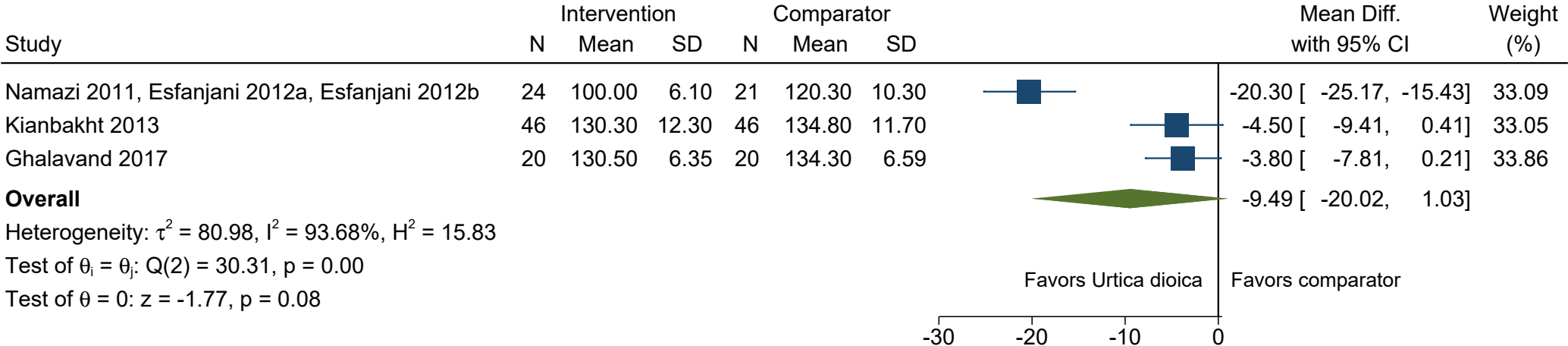

Random-effects REML model

Supplement: Supplementary file 1 [file DataSheet1.zip › Supplementary Material/Forest and Funnel Plots/Urtica dioica/SBP.pdf]

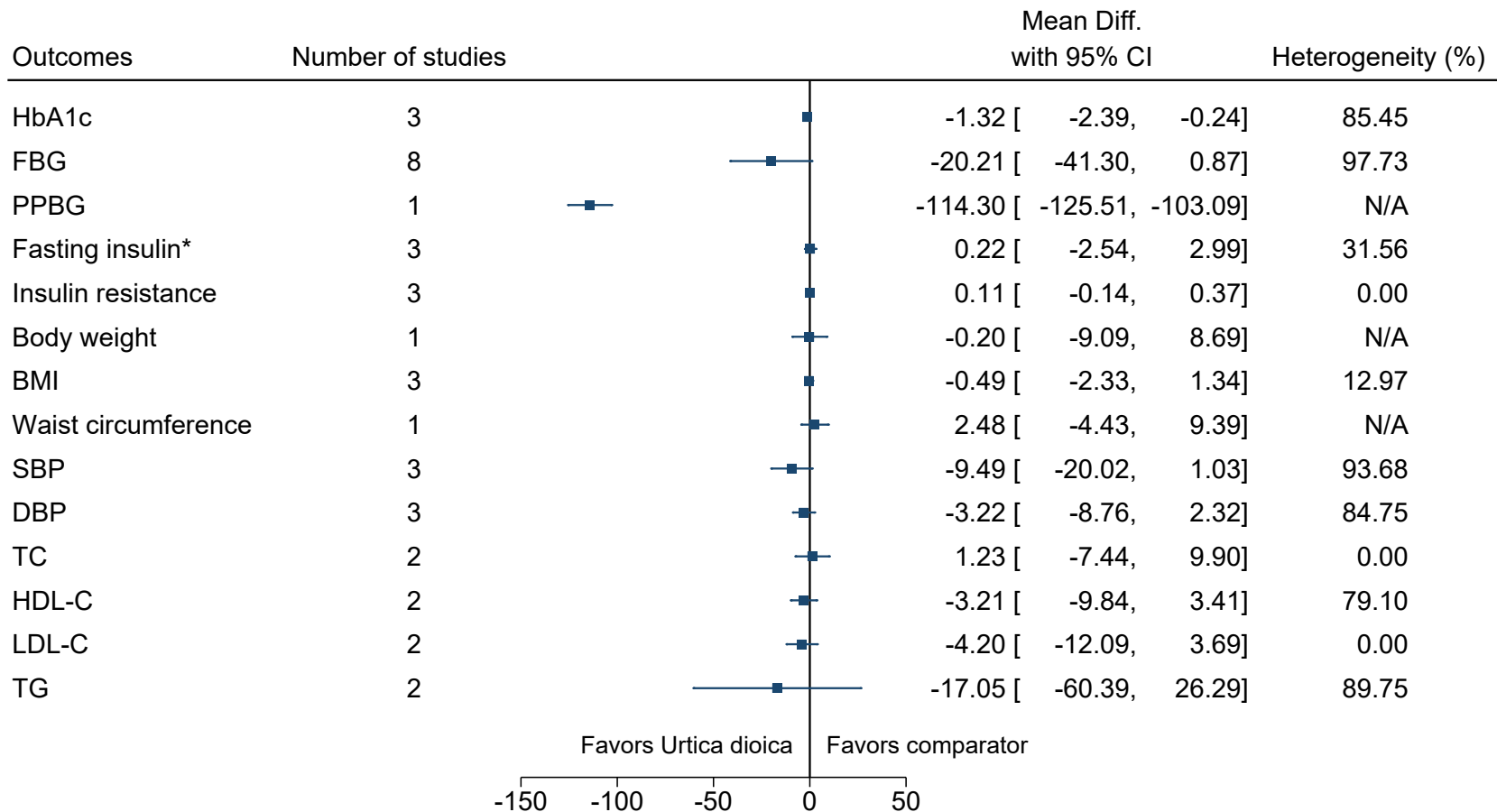

\*Favors intervention/comparator as insulin sensitizer

Supplement: Supplementary file 1 [file DataSheet1.zip › Supplementary Material/Forest and Funnel Plots/Urtica dioica/Urtica dioica.pdf]

# Urtica dioica - LDL-C

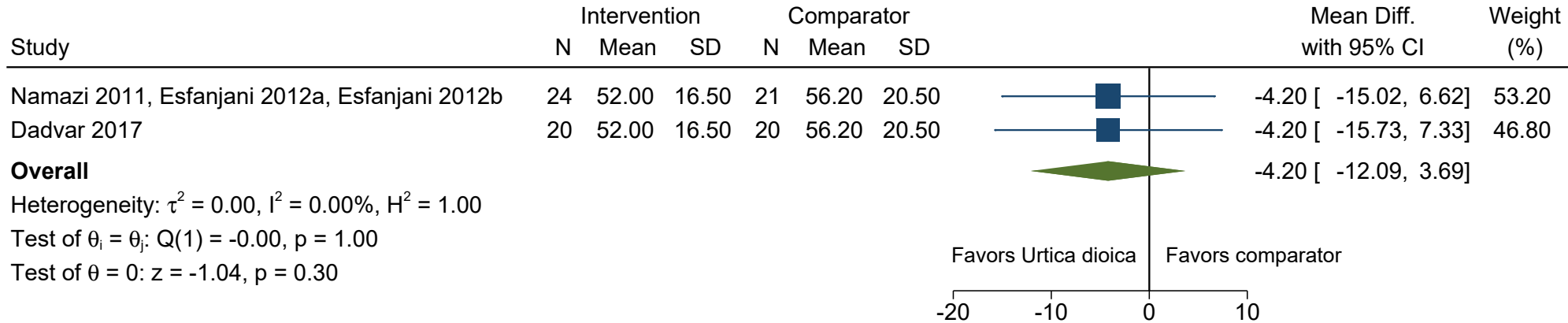

Random-effects REML model

Supplement: Supplementary file 1 [file DataSheet1.zip › Supplementary Material/Forest and Funnel Plots/Urtica dioica/LDL-C.pdf]

# Momordica charantia - FBG

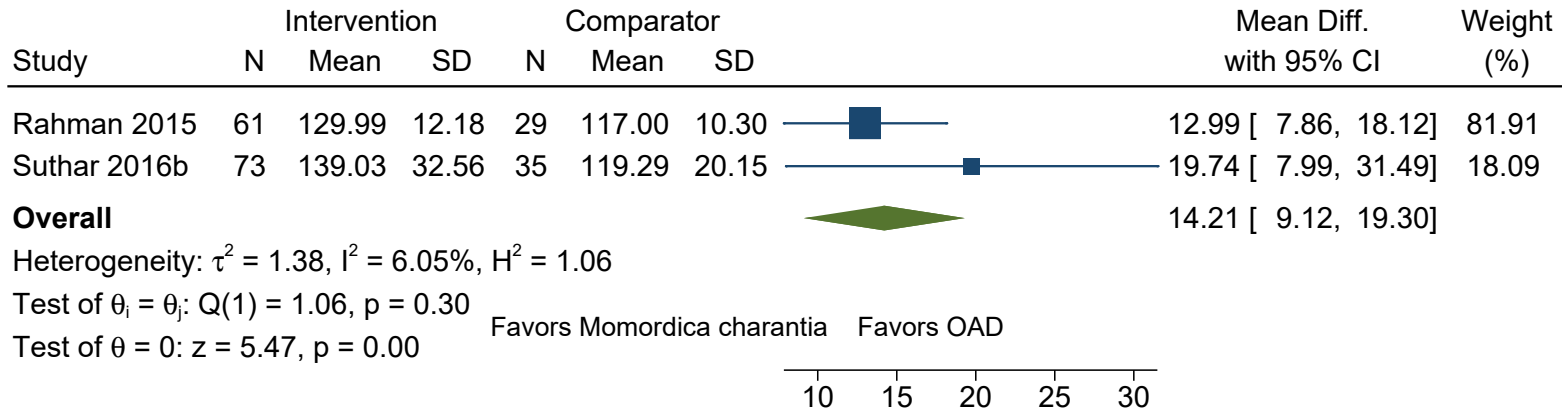

Random-effects REML model

Supplement: Supplementary file 1 [file DataSheet1.zip › Supplementary Material/Forest and Funnel Plots/Momordica charantia (versus OAD)/FBG.pdf]

# Momordica charantia - HbA1c

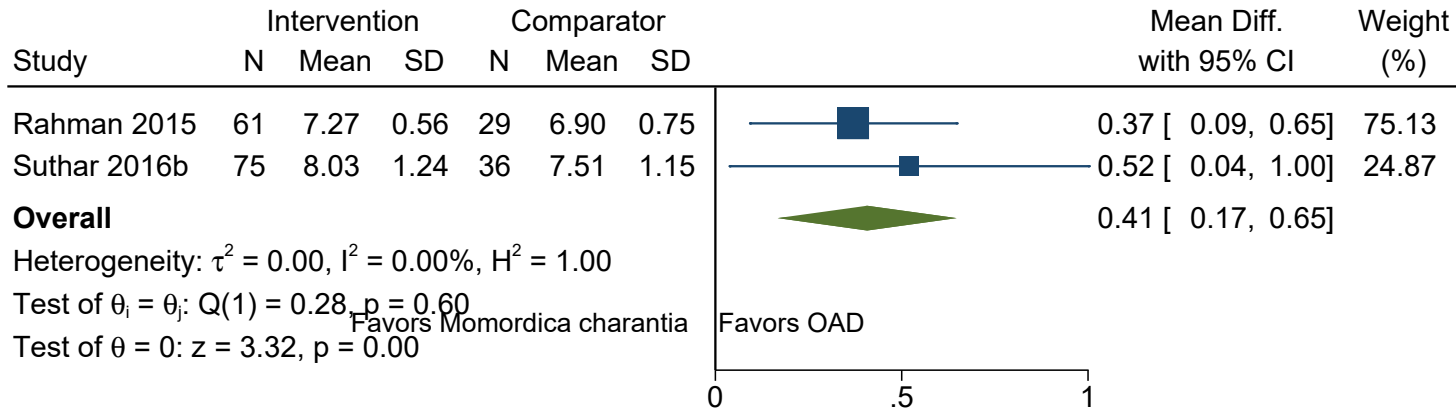

Random-effects REML model

Supplement: Supplementary file 1 [file DataSheet1.zip › Supplementary Material/Forest and Funnel Plots/Momordica charantia (versus OAD)/HbA1c.pdf]

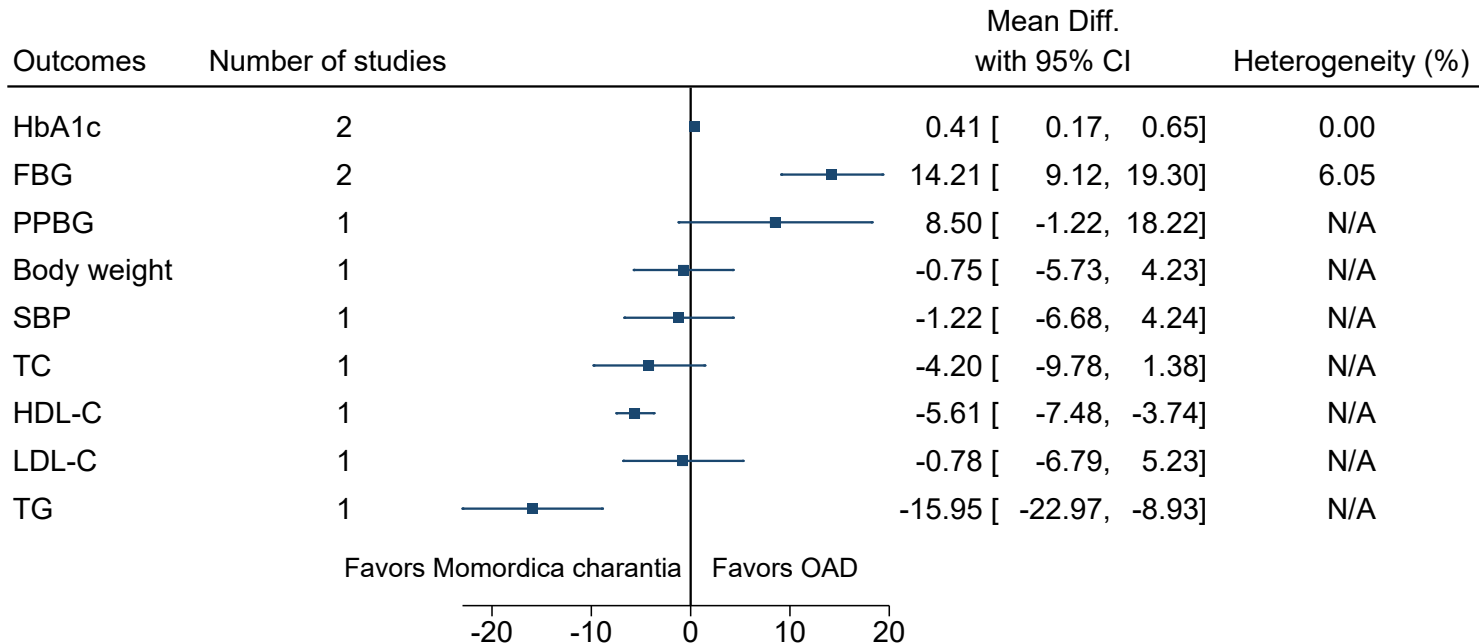

Supplement: Supplementary file 1 [file DataSheet1.zip › Supplementary Material/Forest and Funnel Plots/Momordica charantia (versus OAD)/Momordica charantia.pdf]

# Shilajit - TG

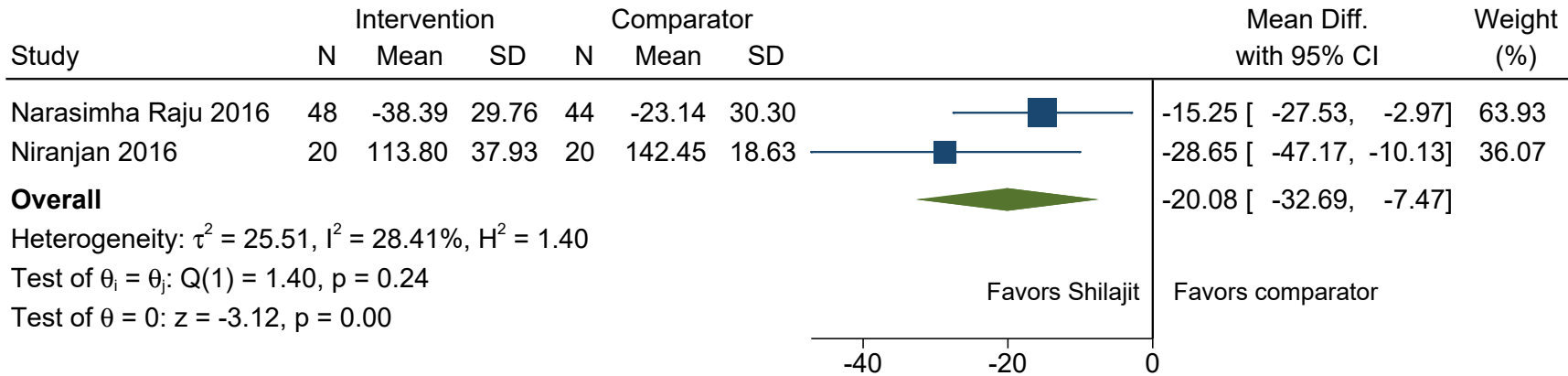

Random-effects REML model

Supplement: Supplementary file 1 [file DataSheet1.zip › Supplementary Material/Forest and Funnel Plots/Shilajit/TG.pdf]

# Shilajit - TC

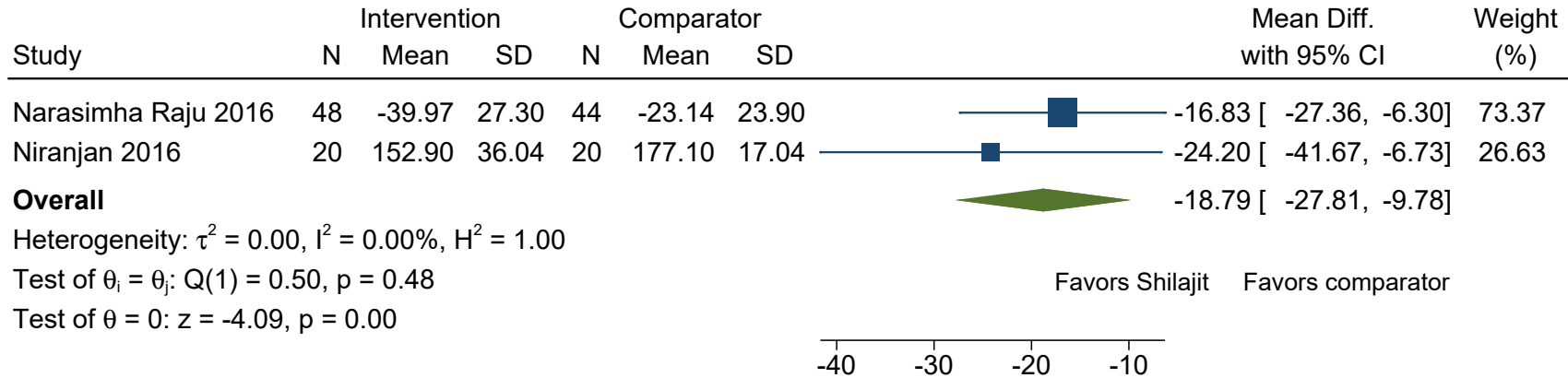

Random-effects REML model

Supplement: Supplementary file 1 [file DataSheet1.zip › Supplementary Material/Forest and Funnel Plots/Shilajit/TC.pdf]

# Shilajit - HDL-C

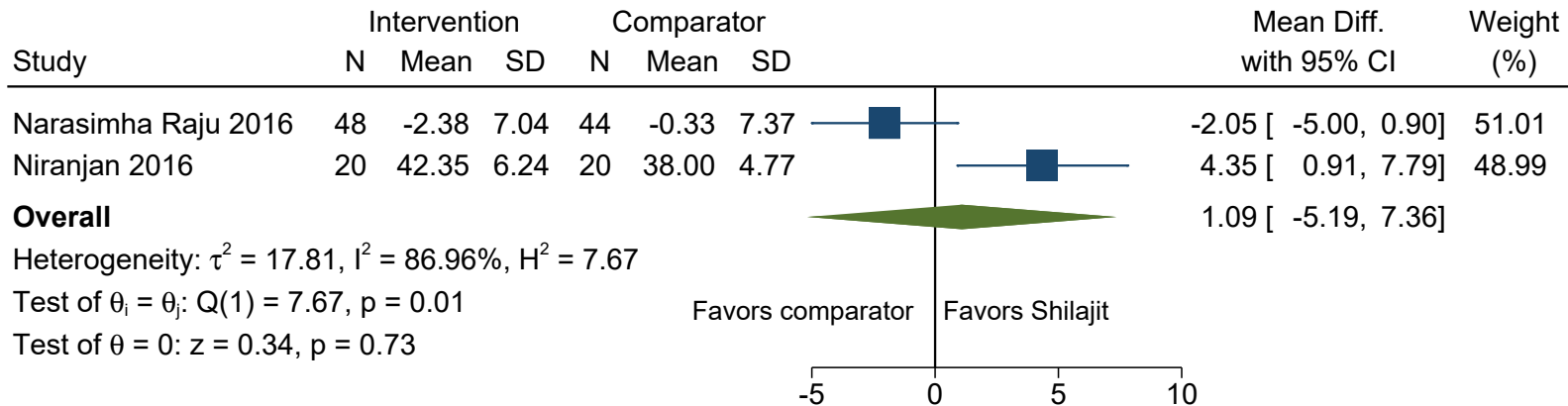

Random-effects REML model

Supplement: Supplementary file 1 [file DataSheet1.zip › Supplementary Material/Forest and Funnel Plots/Shilajit/HDL-C.pdf]

# Shilajit - HbA1c

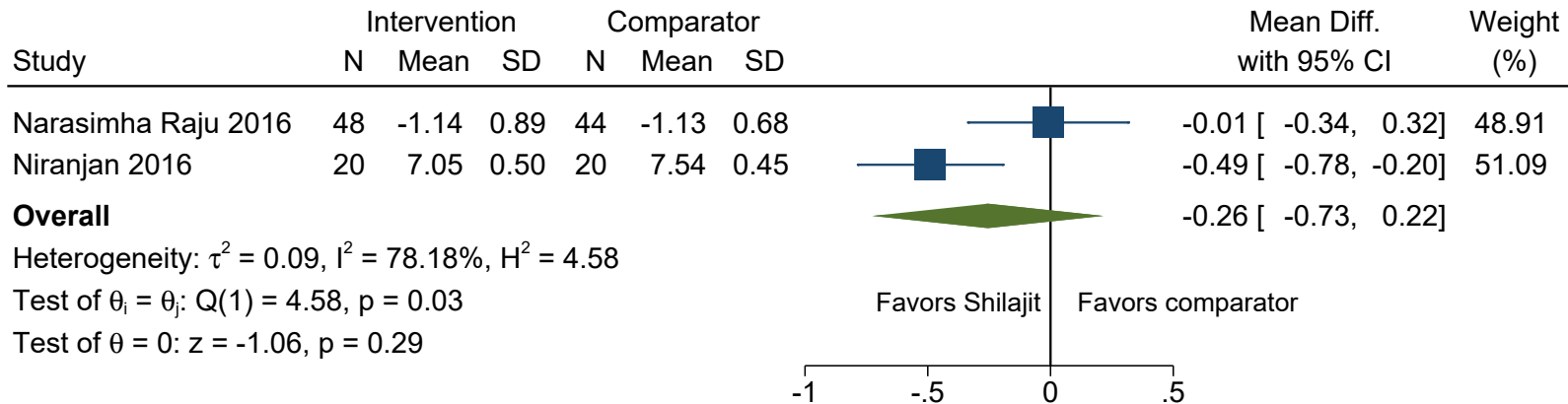

Random-effects REML model

Supplement: Supplementary file 1 [file DataSheet1.zip › Supplementary Material/Forest and Funnel Plots/Shilajit/HbA1c.pdf]

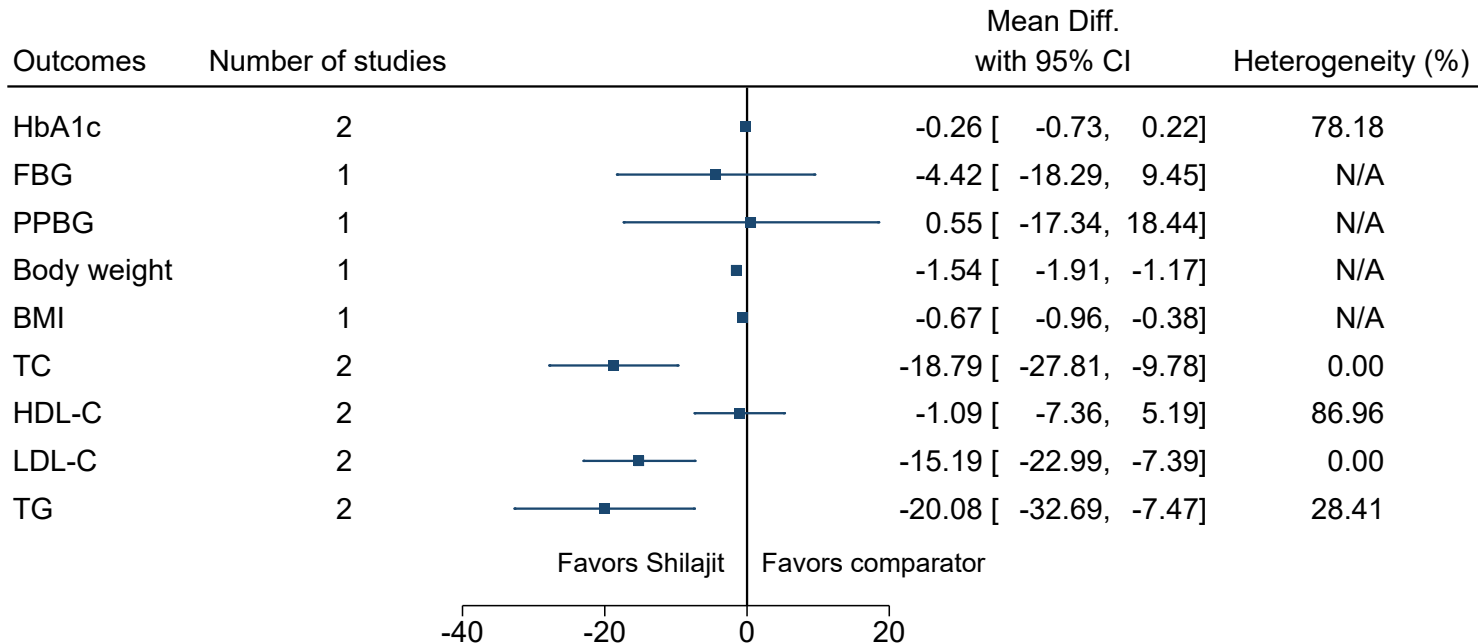

Supplement: Supplementary file 1 [file DataSheet1.zip › Supplementary Material/Forest and Funnel Plots/Shilajit/Shilajit.pdf]

# Shilajit - LDL-C

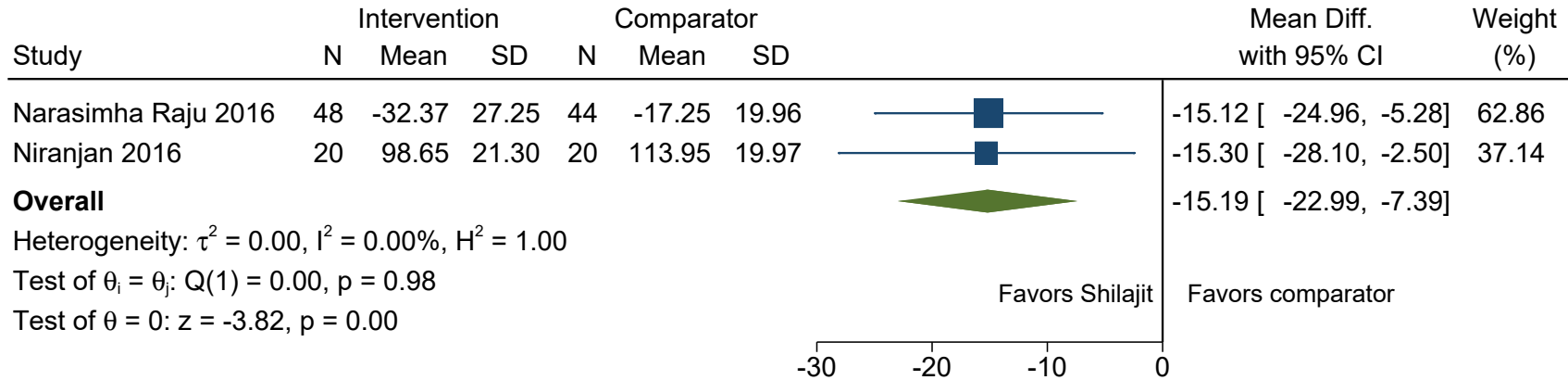

Random-effects REML model

Supplement: Supplementary file 1 [file DataSheet1.zip › Supplementary Material/Forest and Funnel Plots/Shilajit/LDL-C.pdf]

# Allium sativum - TG

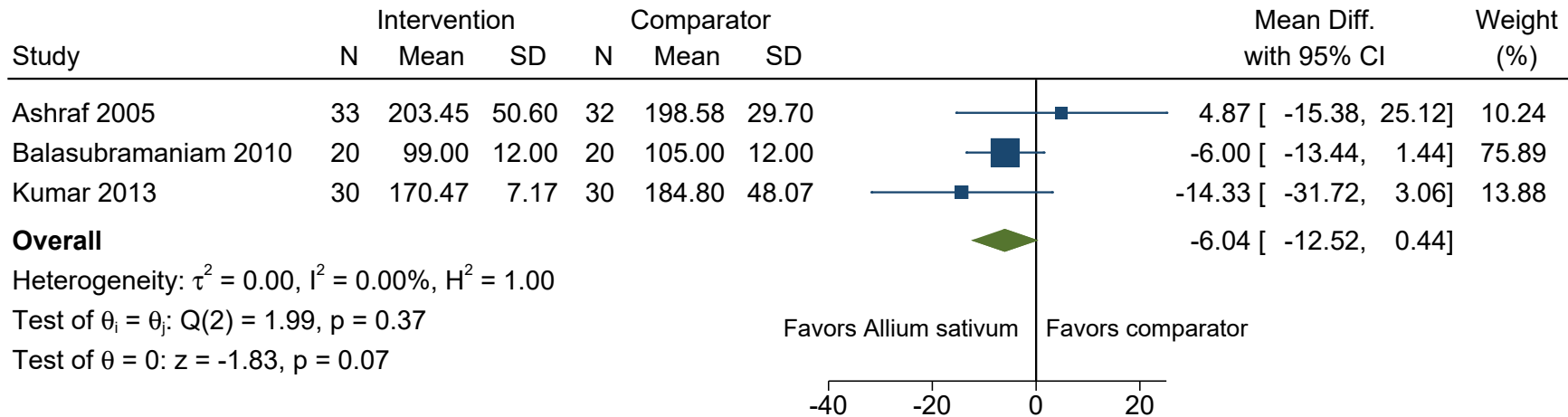

Random-effects REML model

Supplement: Supplementary file 1 [file DataSheet1.zip › Supplementary Material/Forest and Funnel Plots/Allium sativum/TG.pdf]

# Allium sativum - TC

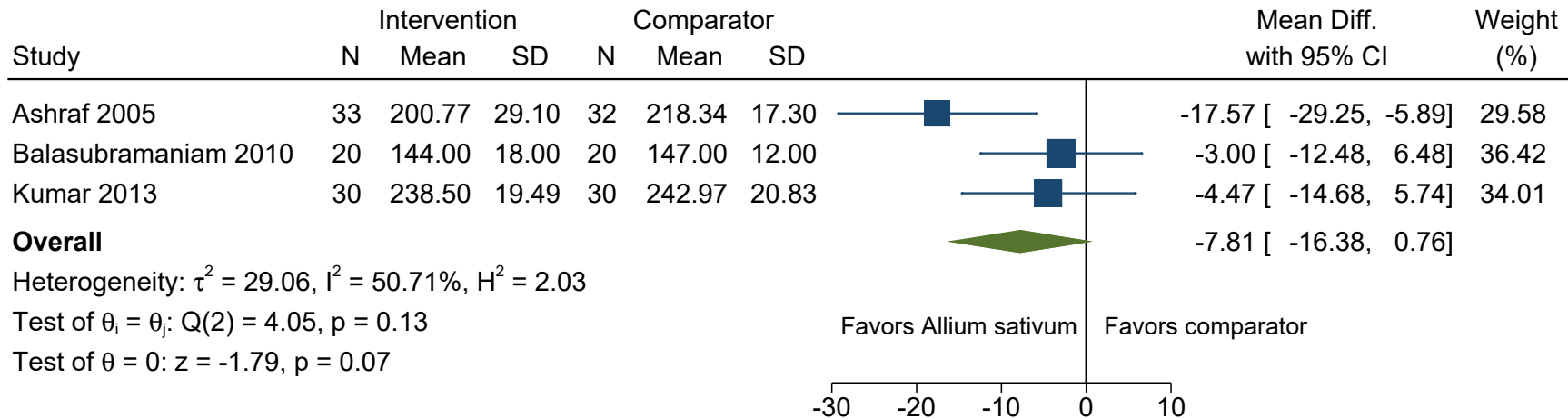

Random-effects REML model

Supplement: Supplementary file 1 [file DataSheet1.zip › Supplementary Material/Forest and Funnel Plots/Allium sativum/TC.pdf]

# Allium sativum - FBG

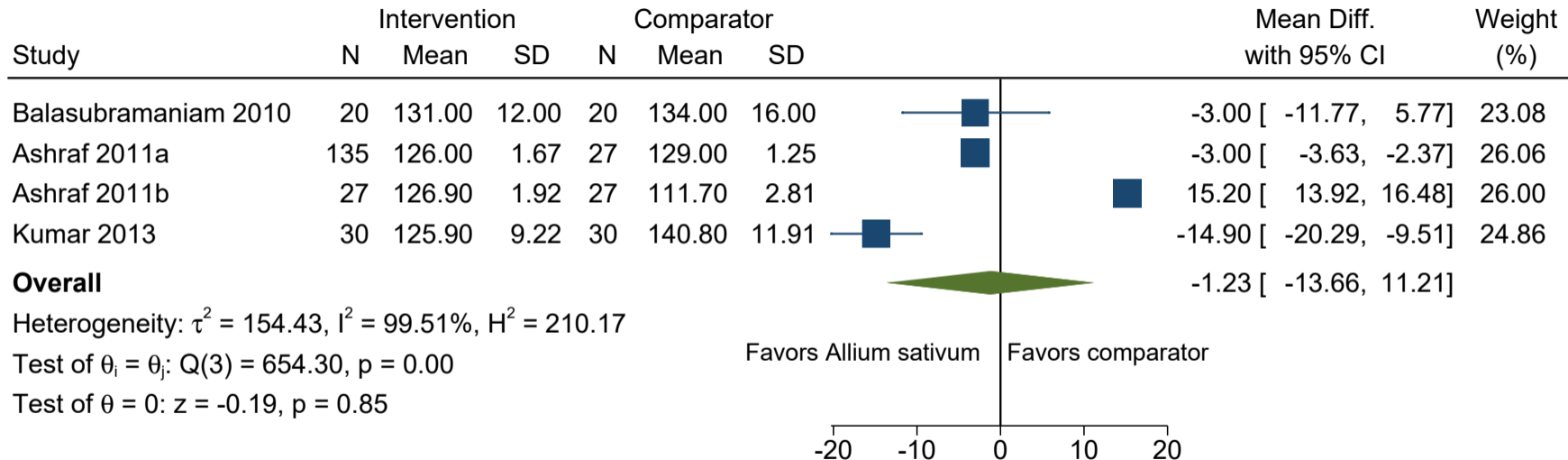

Random-effects REML model

Supplement: Supplementary file 1 [file DataSheet1.zip › Supplementary Material/Forest and Funnel Plots/Allium sativum/FBG.pdf]

# Allium sativum - HDL-C

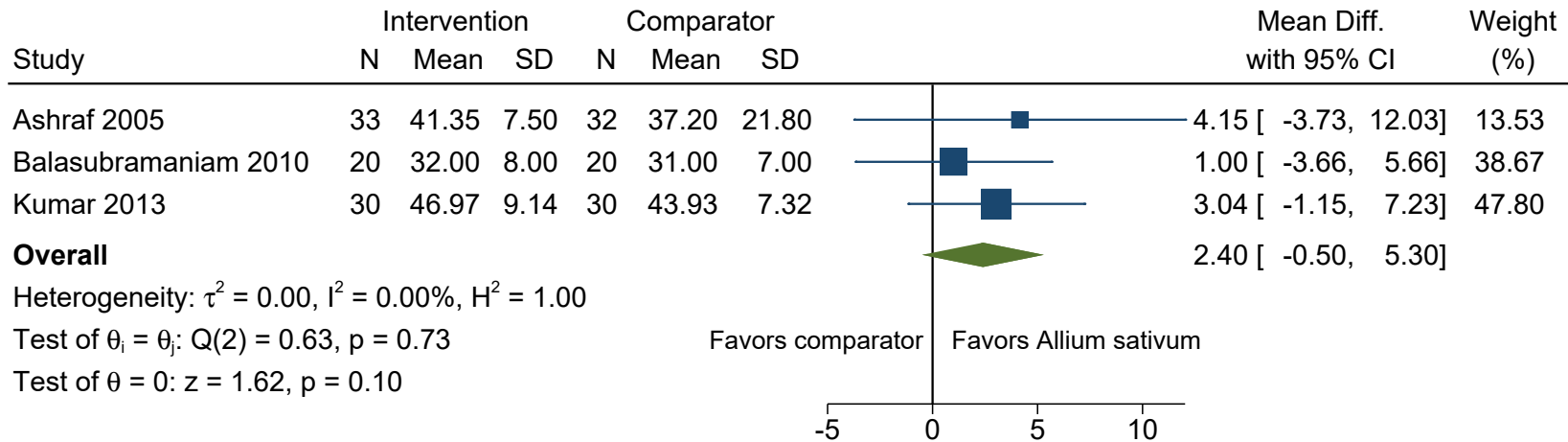

Random-effects REML model

Supplement: Supplementary file 1 [file DataSheet1.zip › Supplementary Material/Forest and Funnel Plots/Allium sativum/HDL-C.pdf]

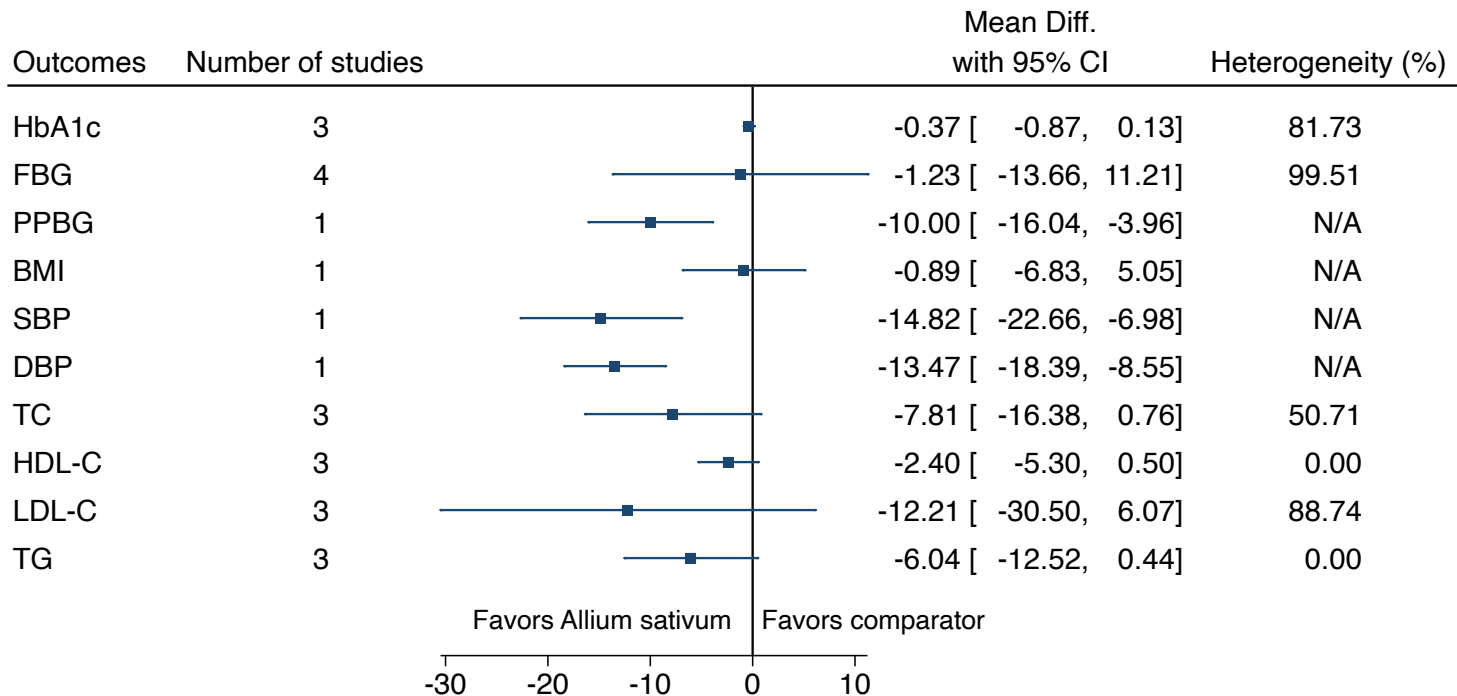

Supplement: Supplementary file 1 [file DataSheet1.zip › Supplementary Material/Forest and Funnel Plots/Allium sativum/Allium sativum.pdf]

# Allium sativum - HbA1c

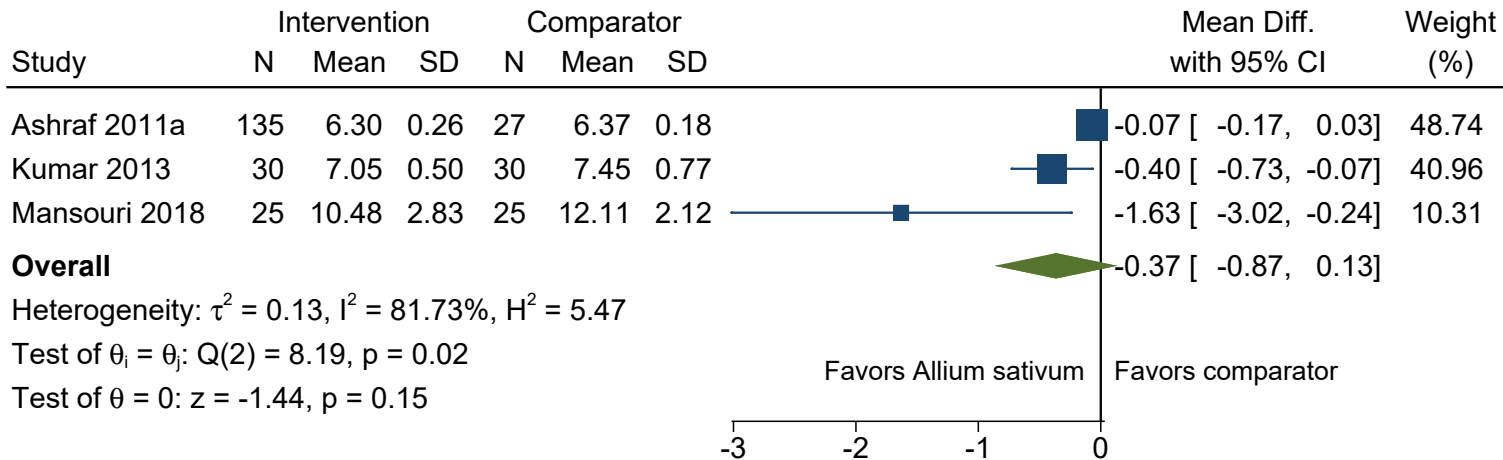

Supplement: Supplementary file 1 [file DataSheet1.zip › Supplementary Material/Forest and Funnel Plots/Allium sativum/HbA1c.pdf]
